# Supplementary material for: Frequency and nature of potentially harmful preventable problems in primary care from the patient’s perspective with clinician review: a population-level survey in Great Britain
Source: BMJ Open. 2018 Jun 13;8(6):e020952. doi: 10.1136/bmjopen-2017-020952 (PMC6009615; doi:10.1136/bmjopen-2017-020952)
Supplement: Supplementary file 1 [file bmjopen-2017-020952supp001.pdf]

Appendix 1. Supplementary methods and results

SJ Stocks et al. BMJ Open 2018: The frequency and nature of potentially-harmful preventable-problems in primary care from the patient's perspective with clinician review – a population level survey in Great Britain

Survey administered as part of the Ipsos MORI GB Face to Face Omnibus between 8th and 21st April 2016

We'd now like you to think about the last time you personally had an appointment for yourself, with a GP.

Q1. Did you have confidence and trust in the GP you saw or spoke to at your last appointment?
1. Yes, definitely 2. Yes, to some extent 3. No, not at all 4. Don't know / can't say

INTERVIEWER INSTRUCTION: READ OUT AND DISPLAY ON SCREEN.

The next few questions are about primary care.

Primary Care is the local healthcare that we receive at our GP or dental surgery, NHS walk-in centres, pharmacists (or high street chemist) and optometrists. This also could include all non-hospital care, for example, healthcare service provided by out of hours care, community (or district) nursing, ambulance, physiotherapy or other types of therapy or tests based at a GP surgery, learning disability services and any other non-hospital medical care.

We understand that this is a highly sensitive topic and would therefore like to remind you that any information you give is strictly confidential and will be used for research purposes only. You will not be identifiable as an individual from the responses you give.

At each question, if you do not wish to answer, you can refuse.

For the next question, we'd like you to think about the occasions when you have personally used primary care for yourself.

Q2a. Have you experienced a situation with a primary care service where your health has ACTUALLY been made worse by a problem or error that could have been prevented?
1. Yes 2. No 3. Don't Know

Q2b. And have you experienced a situation with a primary care service where you SUSPECTED your health has been made worse by a problem or error that could have been prevented?
1. Yes 2. No 3. Don't Know

Q2c. And have you experienced a situation with a primary care service where your health could have been made worse had someone not NOTICED a problem or error?
1. Yes 2. No 3. Don't Know

Q2d. And have you experienced a situation with a primary care service where there was a problem or error that could have been prevented but it did not make your health worse?
1. Yes 2. No 3. Don't Know

IF 2 OR MORE SCENARIOS AT Q2a to Q2e ARE CODED 1 THEN ASK Q2e

Q2e. You mentioned you have experienced the following situation(s) with a primary care service. Which of the following did you experience most recently?

1. 'My health was made worse'
2 'I suspect health was made worse'
3 'My health could have been made worse if the problem or error had not been noticed'
4 'There was no effect on my health'

ASK ALL WHO CODE 1 AT Q2
Q3. Thinking about the most recent occasion where you experienced a preventable problem or error caused by the primary care service, when did this occur?
1. In the last 12 months
2. 1 year up to 2 years ago
3. 2 years up to 3 years ago
4. 3 or more years ago

ASK ALL CODING 1 AT Q2 OR 1 AT Q11
Q4. Thinking about the most recent occasion, which primary care service were you using when the problem or error occurred?
1. GP surgery
2. Out of hours care
3. Walk in clinic
4. Dental surgery
5. Pharmacy
6. Community or district nursing
7. Ambulance
8. Opticians
9. Other (please specify)

INTERVIEWER INSTRUCTION: For the next five questions, please record enough information so that somebody else reading the description can understand what happened.

ASK ALL CODING 1 AT Q2 OR 1 AT Q11
Q5. Thinking about the most recent problem or error you experienced, can you briefly describe what it was and how it happened?

Q6 In your opinion, how, if at all, could the problem or error have been avoided?

Q7. Were you able to talk about the problem or error with anybody WORKING IN THE PRIMARY CARE SERVICE?
1. Yes 2. No

INTERVIEWER INSTRUCTION: if prompted, this can be anyone in the primary care service, including for example, the receptionist at a GP surgery or another nurse/doctor who wasn't working directly in their care.

ASK ALL CODING 1 AT Q7
Q8. You said you were able to discuss the problem or error with somebody working in primary care. Please describe their job or role and their response.

ASK ALL CODING 2 AT Q7

Q9. Which of the following reasons, if any, best describes why you were unable to talk about the problem or error with somebody working in the primary care service?

1. I had the opportunity but did not feel comfortable discussing the problem or error
2. I could not find anybody with whom I could discuss the problem or error
3. I was not concerned about the problem or error
4. I did not notice the problem or error
5. I was too distressed to discuss the problem or error
6. Other (please specify)

ASK IF (Q2 '2 OR DK OR REF')

Q10. In the last 12 months, have any of the following happened to you **while** using primary care, or not? 1. Yes 2. No

IF YES AT Q11, REDIRECT TO Q4

(RANDOMISE 1-16(KEEP 2&3 TOGETHER, KEEP 6&7 TOGETHER, KEEP 9&10 TOGETHER), ALLOW DK AND REF)

1. Received a wrong or late diagnosis
2. Was not referred for further investigation when requested by you as a patient
3. Was not referred for further investigation in error by healthcare practitioner (for example, they forgot to refer you onwards)
4. Test results lost or mixed up
5. Received the wrong medicine or wrong dose
6. Should not have been prescribed medicine because of another health problem
7. Should not have been prescribed medicine because of another medication already being taken
8. Poor communication leading to misunderstanding of diagnosis or treatment
9. Not referred to a specialist when needed when requested by you as a patient
10. Not referred to a specialist when needed in error by healthcare practitioner (for example, they forgot to refer you onwards)
11. Received unclear instructions about treatment
12. Not offered access to prevention or screening programmes e.g. CVD/stroke prevention clinics
13. A medical professional failed to recognise or act on vulnerable people's needs e.g. child abuse, suicide risk or mental health problems
14. Mistake with a procedure e.g. dental treatment, injection, ear syringing, physiotherapy
15. Not notified about recommended vaccinations e.g. flu, HPV
16. A medical professional practicing poor hygiene

ASK ALL CODING 1 AT Q2 OR 1 AT Q11

Q11. Do you, personally, work as a Healthcare Professional in any capacity? For example, a doctor/nurse/therapist/pharmacist/other NHS staff, etc.

1. Yes 2. No

Table A. Coding of patient-reported potentially-unsafe scenarios in primary care

**1. Errors in the process of the healthcare delivery system**	
Makeham 2002, Dovey 2002	Common threads reported in this study
1.1. Errors in the process of conducting an administrative task	A1. Administrative problem not otherwise specified
1.1.1. Information filed in wrong place or wrong time	
1.1.2. Unavailability of information that should have been in patients charts<br>1.1.2.1. Entire chart or part of chart could not be accessed when needed<br>1.1.2.2. Care provided was not documented<br>1.1.2.3. Item(s) of information missing from chart	A2. Incorrect notes/inadequate notes/notes not kept up to date
1.1.3. Errors in patient's movement through the healthcare delivery system	A3. Intended referral was not sent or delayed<br>A4. Patient not reminded, informed or assisted to attend regular check-ups or other necessary routine treatments
1.1.4. Errors in the taking and distributing of messages	
1.1.5. Errors in managing appointments for healthcare	A5. Unable to get an appointment/other problems with making appointment<br>A6. Ambulance delayed or did not arrive
**1.2. Errors in the process of investigating a patient's condition**	
1.2.1. Laboratory errors<br>1.2.1.1. Wrong test ordered or test not ordered when appropriate<br>1.2.1.2. Errors in the process of obtaining or processing a laboratory specimen<br>1.2.1.3. Error in the process of physician receiving accurate laboratory results in a timely fashion<br>1.2.1.4. Inappropriate response to an abnormal laboratory result	B1. Test results lost or other problem with investigation or paperwork<br>B2. Incorrect interpretation of tests or other investigation results<br>B3. Clinician did not consider patient history sufficiently/did not use patient's notes adequately<br>B4. Investigation not thorough enough<br>B5. Not referred when patient felt was needed
1.2.3. Errors in the processes of other investigations<br>1.2.3.1. Wrong test ordered or test not ordered when appropriate<br>1.2.3.2. Errors in the process of obtaining or processing of other diagnostic investigation<br>1.2.3.3. Error in the process of physician receiving accurate test results of other investigation in a timely fashion<br>1.2.3.4. Inappropriate response to an abnormal result of other investigation	
**1.3. Errors in the process of treating a patient's condition**	
1.3.1. Errors in the process of treating with medications<br>1.3.1.1. Wrong medication or wrong dose of medication ordered or medication not ordered by physician when appropriate<br>1.3.1.2. Error in the process of delivering a medication order or inappropriate medication order by a provider working under physician supervision<br>1.3.1.3. Error in the process of dispensing medication as ordered	C1. Medication problem<br><br>C2. Not provided with medical devices needed to manage long term conditions

1.3.2. Errors in other treatments	C3. Problem with dental treatment or diagnosis
**1.4. Errors in the process of communication**	
1.4.1. Errors in communication between primary healthcare provider and patients	D1. Clinician seemed to lack interest in the patient's health problem or did not listen carefully enough<br>D2. Information about the patient's health had not been passed on to the patient who felt it should have been<br>D3. Communication problem between patient and primary care staff
1.4.2. Errors in communication between healthcare providers	D4. Problem with communication between primary care and other types of care including secondary care<br>D5. Disagreement between 2 clinicians
**2. Errors arising from lack of clinical knowledge or skills**	
2.1. Errors in the execution of a clinical task<br>2.1.1. Non-clinical staff made the wrong clinical decision<br>2.1.2. Failed to follow standard practice<br>2.1.3. Lacked needed experience or expertise in a clinical task	E1. Administrative staff seemed to make clinical decisions<br>E2. Procedure was not carried out correctly<br>E3. Incorrect advice/no advice given by clinician
**2.2. Errors in diagnosis**<br>2.2.1. Wrong or delayed diagnosis	F1. Wrong/late/missed/delayed diagnosis
**2.3. Wrong treatment decision**	G1. Wrong treatment decision
	H. Other
	X. Not a problem/ insufficient information/refused/don't know

Table B. Level 4 coding of patient-reported potentially-unsafe medication scenarios

Common threads reported in this study grouped as described by Makeham 2002, Dovey 2002
C1 Medication error not otherwise specified /other problem
• **1.3.1.1. Ordering medications (prescribing)**
C1.1.1 Prescribed wrong or inappropriate drug
C1.1.2 Started new prescription or changed prescription without sufficient discussion, follow up or checks
C1.1.3 Long term or continued prescribing without review or consideration of long term or side effects
C1.1.4 Prescribed drug when should have known contra-indicated *e.g.* patient had informed clinician of allergy, adverse reaction or it was in the records
C1.1.5 Repeat prescription unintentionally changed
C1.1.6 Out of date repeat prescription mistakenly re-issued
• **1.3.1.2./1.3.1.3. Implementing or receiving medications (dispensing or issuing)**
C1.2.1 Medication not dispensed or administered as intended or prescribed
• **1.3.1.1/1.3.1.2./1.3.1.3. Ordering, implementing or receiving medications**
C1.3.1 Wrong dose or drug or delivery method
C1.3.2 Being given another patient's drugs or prescription
C1.3.3 Wrong or inadequate advice about drug effects or how to use
C1.3.4 Delay or failure in prescription processing

Table C. Scoring for likelihood that the patient-reported scenario is potentially-unsafe

Score	How likely do you think it is the patient was correct in thinking that their health might be worsened, or actually was made worse, because of a mistake or a problem in primary care that could have been prevented? Choose from the options below.
5	Very likely or certain (75-100% confident is a potentially unsafe scenario)
4	Probably (50-74% confident is a potentially unsafe scenario)
3	Possibly (25-49% confident is a potentially unsafe scenario)
2	Unlikely (bottom 25% confident is a potentially unsafe scenario)
1	Definitely not a potentially unsafe event (0% chance is a potentially unsafe scenario)
-	Insufficient information
-	Don't know
-	Other - add text at end of row

Supplementary results

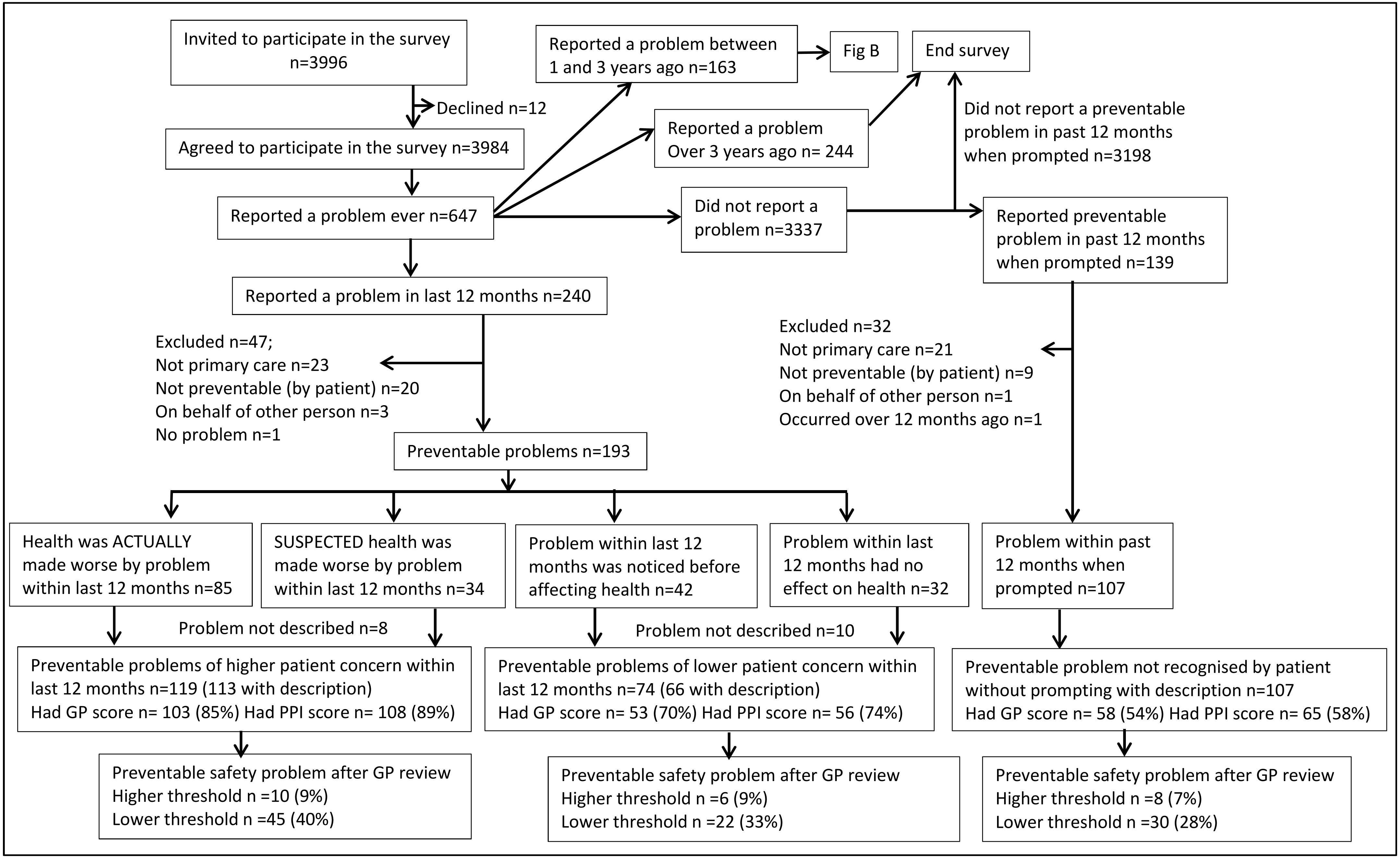

Fig A. Flow chart of participants reporting a potential-harmful preventable-problem within the last 12 months

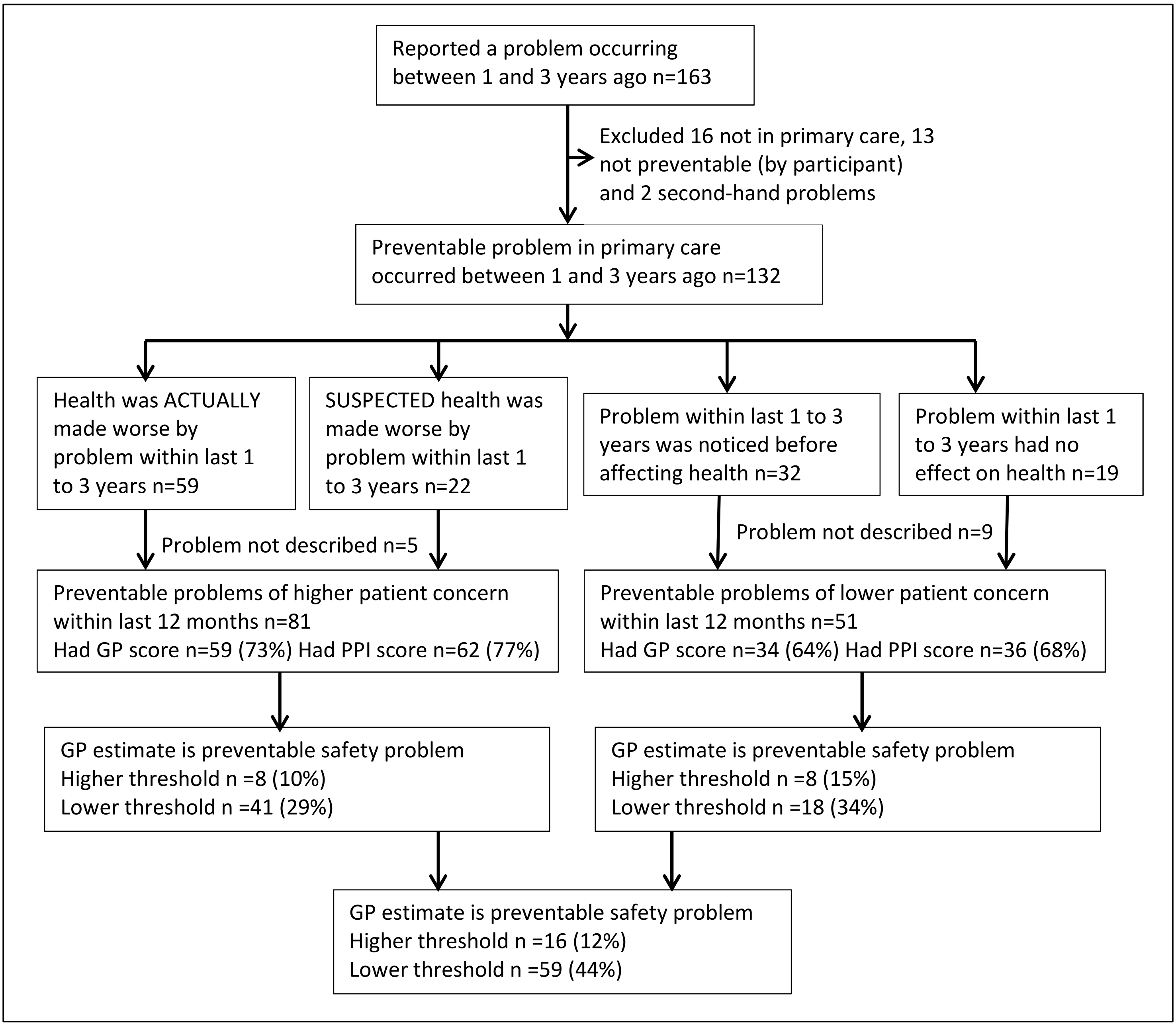

Fig B. Flow chart of participants reporting a potential-harmful preventable-problem within the last 1 to 3 years

Table D. Demographics of responders to Ipsos MORI GB Face to Face Omnibus April 2016

	Number of participants (%) n=3984	Population level estimates for comparison	Population comparator source; $P(\chi^2)$= probability survey population differs from population comparator
**Confidence and trust in GP at last appointment?**			
Yes definitely	3031 (76%)	523498 (63%)	GP patient survey in England mid-2015(25) $P(\chi^2)<0.0001$
Yes, to some extent	611 (15%)	235760 (29%)	
No, not at all	311 (8%)	37743 (5%)	
Don't know /can't say	31 (1%)	28866 (3%)	
**Gender (1 missing)**			
Male	1950 (49%)	32074400 (49%)	ONS mid-2015 estimates[1] $P(\chi^2)=0.7$
Female	2033 (51%)	33035600 (51%)	
**Age**			
15 to 24	533 (13%)	8118600 (15%)	ONS mid-2015 estimates[1] $P(\chi^2)<0.0001$
25 to 34	573 (14%)	8822700 (16%)	
35 to 44	528 (13%)	8378300 (16%)	
45 to 54	629 (16%)	9196000 (17%)	
55 to 64	654 (16%)	7452400 (13%)	
65 to 74	609 (15%)	6339800 (11%)	
75 or older	458 (12%)	5271400 (10%)	
**Ethnicity (18 missing)**			
White	3491 (88%)	48209395 (86%)	England & Wales census (2011)[2] $P(\chi^2)<0.0001$
Other ethnicity	475 (12%)	7866517 (14%)	
**Social Grade[3]**			
A/B	1054 (26%)	8081619 (23%)	England & Wales census (2011)[2] $P(\chi^2)<0.0001$
C1	1122 (28%)	10796044 (30%)	
C2	771 (19%)	7865976 (22%)	
D/E	1037 (26%)	8903873 (25%)	

[1]https://www.ons.gov.uk/peoplepopulationandcommunity/populationandmigration/populationestimates/bulletins/annualmidyearpopulationestimates/latest

[2]https://www.ons.gov.uk/peoplepopulationandcommunity/populationandmigration/populationestimates/bulletins/keystatisticsandquickstatisticsforlocalauthoritiesintheunitedkingdom/2013-10-11

[3]A/B High or intermediate managerial, professional or administrative, C1 Supervisory, clerical and junior managerial, professional or administrative, C2 skilled manual workers, D/E semi and unskilled manual workers, casual or lowest grade workers, state pensioners, unemployed with state benefits only

Table E. Categorisation of patient-described scenarios according to clinician ranking as to the likelihood they represent a potentially-harmful preventable-problem

Group	Scores on a 5 point scale of “very likely or certain”, “probably”, “possibly”, “unlikely”, “definitely not” (see table C, online Appendix 2)	Unprompted problems (answered “yes” to Q2, Box1)				All problems within past 12 months (answered “yes” to Q2or Q10, Box1) n=300	
		Within past 12 months n=193		Within past 3 years n=325			
		Clinicians	Members of the Public	Clinicians	Members of the Public	Clinicians	Members of the Public
1. Higher threshold	Median score higher than “probably” or at least one score of “very likely or certain”	16 (8%)	91 (47%)	28 (9%)	165 (51%)	24 (8%)	116 (39%)
2. Lower threshold	Median score higher than “possibly” or at least one score of “probably” or higher	67 (35%)	145 (75%)	124 (38%)	237 (73%)	97 (32%)	198 (66%)
3. Any possibility	At least one score of “unlikely” or higher	141 (73%)	157 (81%)	232 (71%)	254 (78%)	194 (65%)	221 (74%)
4. No problem	All scores “definitely not” (or not-coded)	8 (4%)	0	9 (3%)	0	13 (4%)	0
5. Not-coded	Insufficient information for coding by all raters	44 (23%)	36 (19%)	84 (26%)	71 (22%)	93 (31%)	79 (26%)

Table F. Survey responses and respondent characteristics as predictors of clinician and members of the public estimates of the likelihood that the scenario describes a potentially-harmful preventable problem

Respondent characteristics (total) n=406 (ranked by at least one clinician)	Clinician – lower threshold[1] (n=224, 55%)		Members of the public – higher threshold[2] (n=267, 66%)	
	Frequency (%)	Adjusted odds ratio	Frequency (%)	Adjusted odds ratio
**Source of respondent (0 missing)**				
Ipsos MORI f2f Omnibus (299)	153 (51%)	1 (ref)	182 (61%)	1 (ref)
Pilot survey (107)	71 (66%)	1.5 (0.9 to 2.7)	85 (79%)	5.2 (2.5 to 10.8)
**Gender (3 missing)**				
Male (150)	79 (53%)	1 (ref)	93 (62%)	1 (ref)
Female (253)	142 (56%)	1.2 (0.8 to 1.9)	172 (68%)	1.5 (0.9 to 2.4)
**Age (3 missing)**				
15 to 24 years (46)	21 (46%)	1 (ref)	28 (61%)	1 (ref)
25 to 34 years (60)	34 (57%)	1.5 (0.7 to 3.5)	43 (72%)	1.4 (0.6 to 3.7)
35 to 44 years (38)	24 (63%)	1.8 (0.7 to 4.5)	30 (79%)	1.9 (0.6 to 5.6)
45 to 54 years (74)	44 (59%)	1.5 (0.7 to 3.4)	50 (68%)	1.1 (0.5 to 2.7)
55 to 64 years (82)	45 (55%)	1.4 (0.6 to 3.2)	50 (61%)	1.0 (0.4 to 2.3)
65 to 74 years (75)	39 (52%)	1.2 (0.5 to 2.8)	49 (65%)	1.1 (0.4 to 2.6)
75 years or older (28)	14 (50%)	1.1 (0.4 to 3.2)	15 (54%)	0.6 (0.2 to 1.8)
**Patient estimate of impact of the problem on their health (0 missing)**				
Actually or suspected made health worse (192)	109 (57%)	1 (ref)	139 (73%)	1 (ref)
Noticed before made health worse or had no effect on health (106)	58 (55%)	0.8 (0.5 to 1.4)	69 (65%)	0.6 (0.3 to 1.1)
Prompted by Q10 (108)	57 (53%)	0.7 (0.4 to 1.2)	59 (55%)	0.3 (0.1 to 0.5)
**Patient is qualified as a healthcare professional or volunteers in healthcare research[2] (0 missing)**				
No (339)	177 (52%)	1 (ref)	221 (65%)	1 (ref)
Yes (67)	47 (70%)	2.0 (1.1 to 3.8)	46 (69%)	0.8 (0.4 to 1.7)
**Discussed the problem with somebody working in the primary care service (0 missing)**				
No/don't know/missing (197)	99 (50%)	1 (ref)	119 (60%)	1 (ref)
Yes (209)	125 (60%)	1.3 (0.9 to 2.0)	148 (71%)	1.5 (0.9 to 2.4)
**Service used (1 missing)**				
GP surgery (286)	159 (56%)	1 (ref)	186 (65%)	1 (ref)
Dental surgery (36)	17 (46%)	0.8 (0.3 to 1.7)	12 (33%)	1.1 (0.5 to 2.7)
Walk in clinic (16)	7 (44%)	1.0 (0.4 to 3.0)	10 (63%)	1.7 (0.5 to 5.7)
Ambulance/A&E/ OOH (20)	13 (65%)	2.0 (0.7 to 5.5)	15 (75%)	3.8 (1.0 to 14.1)
Pharmacy (18)	15 (83%)	2.0 (0.5 to 7.8)	3 (17%)	1.0 (0.2 to 4.3)
Other (29)	12 (41%)	0.7 (0.3 to 1.7)	14 (48%)	1.4 (0.6 to 3.4)
**Problem related to (0 missing)**				
A. Healthcare delivery system (65)	25 (38%)	1 (ref)	24 (37%)	1 (ref)
B. Investigation (63)	29 (46%)	1.2 (0.6 to 2.5)	42 (67%)	3.4 (1.5 to 7.6)
C. Treatment process (100)	73 (73%)	3.7 (1.8 to 7.7)	85 (85%)	11.0 (4.6 to 26.5)
D. Communication (66)	36 (55%)	1.8 (0.9 to 3.7)	37 (56%)	2.0 (0.9 to 4.2)
E. Clinical knowledge or skills (43)	23 (53%)	1.8 (0.8 to 4.2)	30 (70%)	3.3 (1.3 to 8.4)
F. Diagnosis (56)	34 (61%)	2.5 (1.1 to 5.4)	79 (21%)	6.2 (2.6 to 15.1)
G. Wrong treatment decision (4)	2 (50%)	1.4 (0.2 to 11.5)	3 (75%)	3.9 (0.4 to 41.7)

H. Other (9)	2 (22%)	0.5 (0.1 to 2.8)	2 (22%)	0.4 (0.1 to 2.2)
**Relevant condition (0 missing)**	**Frequency (%)**	**Unadjusted odds ratio[3]**	**Frequency (%)**	**Unadjusted odds ratio[3]**
All other conditions (47)	24 (51%)	1 (ref)	29 (19%)	1 (ref)
Cardiovascular (8)	7 (88%)	6.7 (0.8 to 58.9)	8 (100%)	-[4]
Diabetes (32)	20 (63%)	1.6 (0.6 to 4.0)	24 (75%)	1.8 (0.7 to 5.0)
Cancer (7)	7 (100%)	-[4]	7 (100%)	-[4]
Mental health (18)	6 (33%)	0.5 (0.2 to 1.5)	15 (83%)	3.1 (0.8 to 12.2)
Dental (33)	16 (48%)	0.9 (0.4 to 2.2)	24 (73%)	1.7 (0.6 to 4.3)
Accidental injury (17)	10 (59%)	1.4 (0.4 to 4.2)	12 (71%)	1.5 (0.4 to 4.9)
Infectious (12)	8 (67%)	1.9 (0.5 to 7.2)	10 (83%)	3.1 (0.6 to 15.8)
Pain/discomfort (15)	8 (53%)	1.1 (0.3 to 3.5)	5 (30%)	0.3 (0.1 to 1.1)
Skin (12)	5 (42%)	0.7 (0.2 to 2.5)	4 (33%)	0.3 (0.1 to 1.2)
Respiratory (13)	9 (69%)	2.2 (0.6 to 8.0)	12 (92%)	7.4 (0.9 to 62.2)
Pregnancy (8)	6 (75%)	2.9 (0.5 to 15.7)	8 (100%)	-[4]
Musculoskeletal (34)	11 (32%)	0.5 (0.2 to 1.1)	16 (47%)	0.6 (0.2 to 1.3)
Ear, nose and throat (9)	6 (67%)	1.9 (0.4 to 8.6)	4 (44%)	0.5 (0.1 to 2.1)
Not relevant/not known (141)	81 (57%)	1.3 (0.7 to 2.5)	89 (63%)	1.1 (0.5 to 2.1)

[1]median score higher than “probably” or at least one score of “very likely or certain”, see Table B

[2]median score higher than “possibly” or at least one score of “probably” or higher, see Table B

[3]unadjusted OR shown due to collinearity between dental problems and dental service

[4]predicts success perfectly (100% of scenarios in this category)

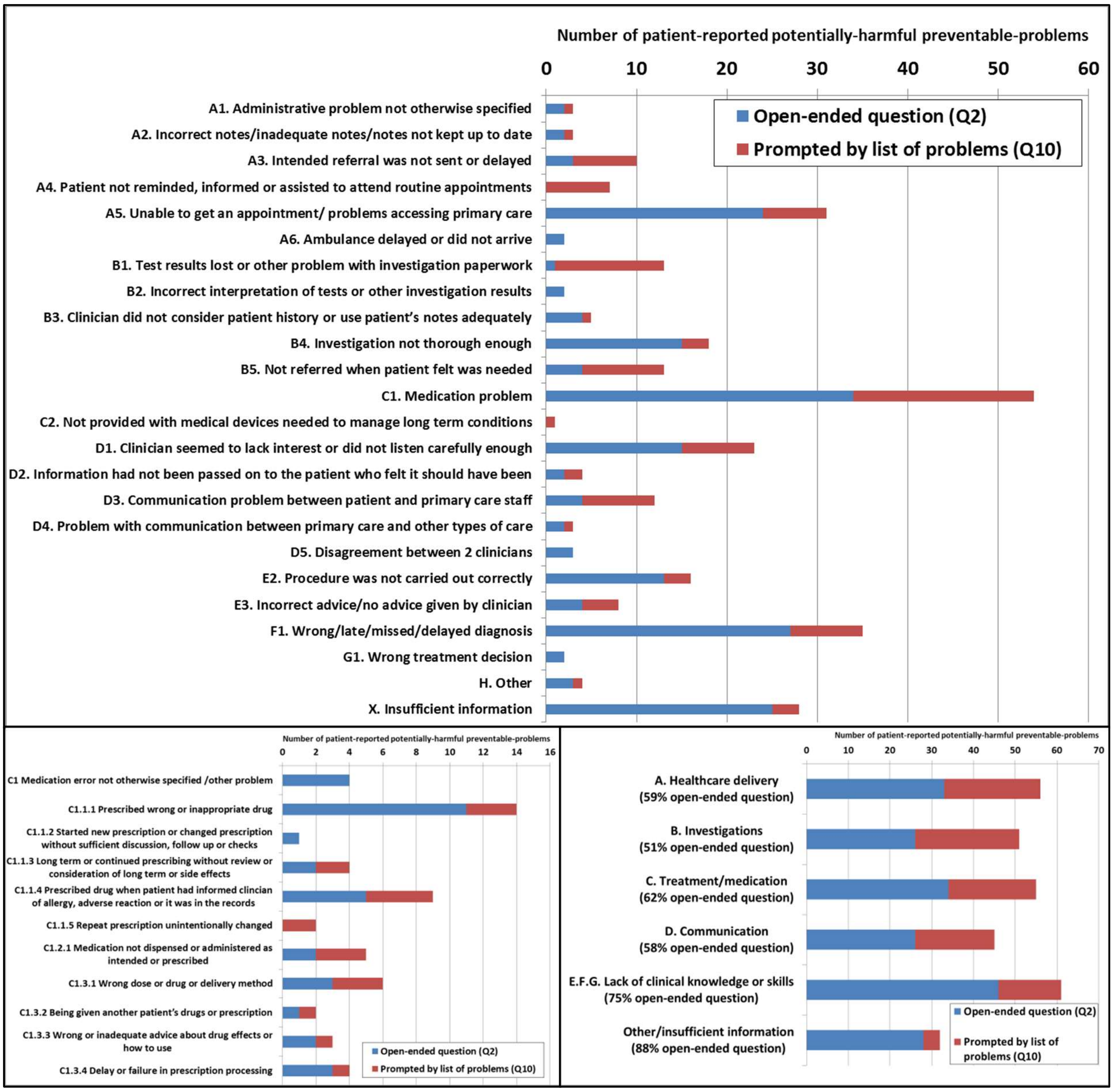

Fig C. Numbers of patient-perceived problems occurring in the last 12 months categorised according to the patient's description (see Table 2) and route through survey *i.e.* originated from open-ended question (Q2) or prompted by list of potential safety problems (Q10). See online Appendix 2 for details of coding; A coded to 2 levels, B medication problems coded to 3 levels, C coded to 1 level
