# Supplementary material for: Frequency and nature of potentially harmful preventable problems in primary care from the patient’s perspective with clinician review: a population-level survey in Great Britain
Source: BMJ Open. 2018 Jun 13;8(6):e020952. doi: 10.1136/bmjopen-2017-020952 (PMC6009615; doi:10.1136/bmjopen-2017-020952)
Supplement: Supplementary file 2 [file bmjopen-2017-020952supp002.pdf]

Appendix 2. SJ Stocks et al. BMJ Open 2018: The frequency and nature of potentially-harmful preventable-problems in primary care from the patient's perspective with clinician review – a population level survey in Great Britain Patient reported scenarios occurring during the past 12 months that clinicians scored as higher likelihood to be a potentially-unsafe preventable-problem in primary care (median score is higher than "possibly" and at least 2 clinicians gave a score or one clinician scored "very likely or certain"). PPI = member of the public, GP = primary care clinician

Scenario1. Ambulance

Briefly describe the mistake or problem and how it happened. *"Heart attack, an ambulance was called and waited an hour and three quarters to arrive"*

Could the mistake or problem have been avoided? If so how? "*The ambulance service needs to be sorted out"*

Were you able to talk about the mistake or problem with anybody working in the primary care service? *"No I was too distressed to discuss the problem or error"*

Patient-reported prospect of harm: health could have been made worse had someone not noticed a problem or error

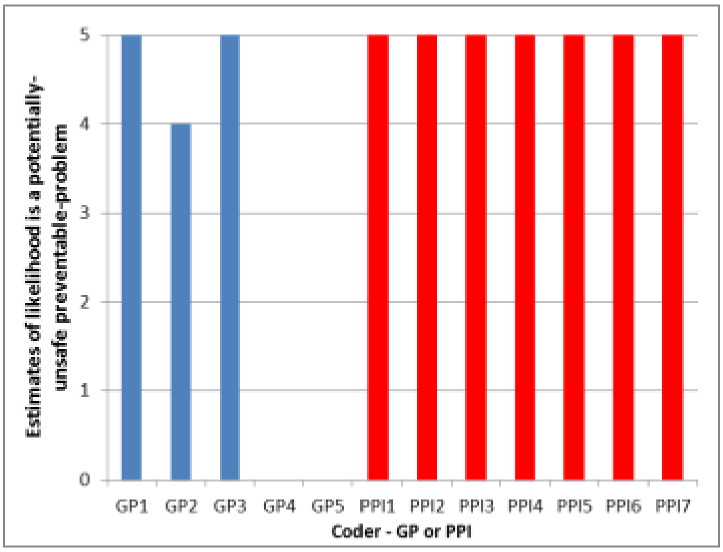

5=very likely or certain, 4=probably, 3=possibly, 2=unlikely, 1=definitely not, 0 = insufficient information or don't know

Patient-perspective problem-type code: A6. Ambulance delayed or did not arrive

Scenario2. GP surgery

Briefly describe the mistake or problem and how it happened. *"I had an ongoing stomach complaint. The GP kept prescribing a steroid treatment but the pharmacist refused to give it to me. He said it was dangerous and I had to get different medication. The GP prescribed an alternative but the pharmacist pointed out that the steroid was supposed to be a short term treatment and that the GP had been prescribing it for over a year."*

Could the mistake or problem have been avoided? If so how? "*The GP obviously didn't read the notes. The GP was probably pushed for time and just wanted to get me out (maybe?)"*

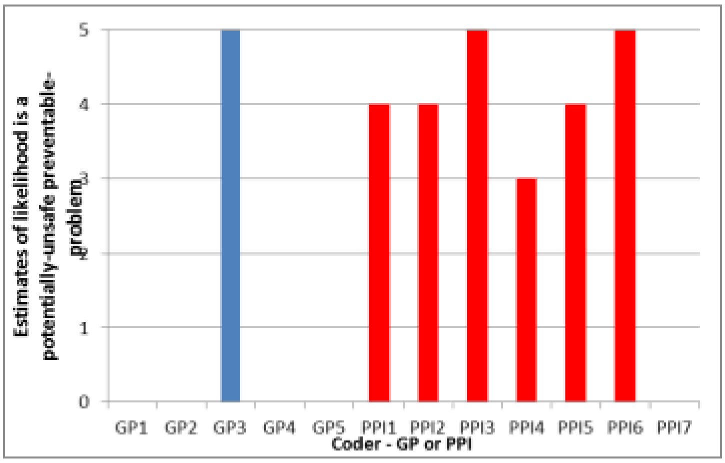

5=very likely or certain, 4=probably, 3=possibly, 2=unlikely, 1=definitely not, 0 = insufficient information or don't know

Were you able to talk about the mistake or problem with anybody working in the primary care service? *"No I was not concerned about the problem or error"*

Patient-reported prospect of harm: prompted via Q10 (Box 1 main paper)

Patient-perspective problem-type code: C1.1.3 Long term or continued prescribing without review or consideration of long term or side effects; B3 Clinician did not consider patient history sufficiently/did not use patient's notes adequately

Scenario3. GP surgery

Briefly describe the mistake or problem and how it happened. *"Participant was prescribed penicillin and it was stated in notes that patient was allergic to penicillin"*

Could the mistake or problem have been avoided? If so how? *"It was avoided as participant didn't take prescription and was prescribed something else"*

Were you able to talk about the mistake or problem with anybody working in the primary care service? *"Yes with GP"*

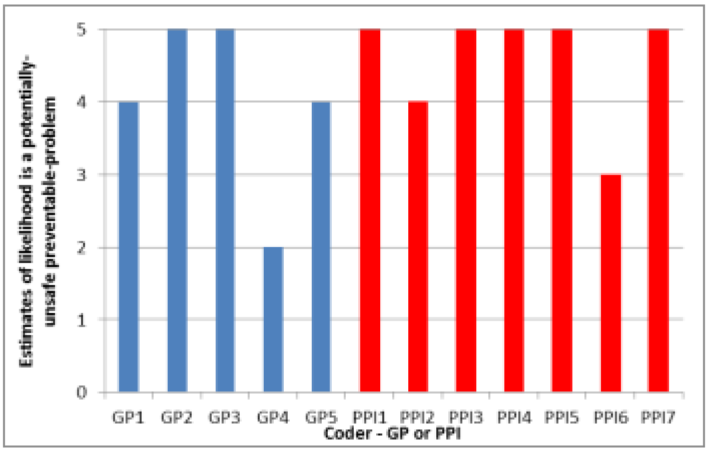

5=very likely or certain, 4=probably, 3=possibly, 2=unlikely, 1=definitely not, 0 = insufficient information or don't know

Patient-reported prospect of harm: health could have been made worse had someone not noticed a problem or error

Patient-perspective problem-type code: C1.1.4 Prescribed drug when should have known contra-indicated *e.g.* patient had informed clinician of allergy, adverse reaction or it was in the records

Scenario4. Optician

Briefly describe the mistake or problem and how it happened. *"Started suffered blurred vision in left eye, eye was bloodshot. Went to get eye check and was sold eye drops to treat infection, told would take five days. After five days of treatment problem was made worse until vision was affected, GP referred to eye clinic diagnosed with iritis. Further treatment at eye clinic cleared up the issue."*

Could the mistake or problem have been avoided? If so how? *"If optometrists had spotted that iris was stuck, had a bit more professional care rather than trying to flog over-the-counter eye drops to clear up infection that wasn't there"*

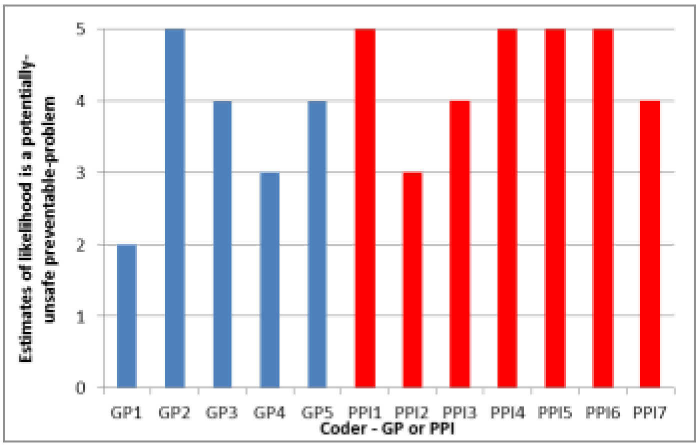

5=very likely or certain, 4=probably, 3=possibly, 2=unlikely, 1=definitely not, 0 = insufficient information or don't know

Were you able to talk about the mistake or problem with anybody working in the primary care service? *"Yes, spoke to GP, immediate referral to eye clinic for treatment"*

Patient-reported prospect of harm: health was actually made worse by a problem or error that could have been prevented

Patient-perspective problem-type code: F1 Wrong/late/missed/delayed diagnosis

Scenario5. GP surgery

Briefly describe the mistake or problem and how it happened. *"Contra-indication with a medicine that was not noticed at time of prescription but was noticed by the participant before they started taking the medicine"*

Could the mistake or problem have been avoided? If so how? "*The contra-indication should have been flagged up on the computer at the time of prescription but it wasn't"*

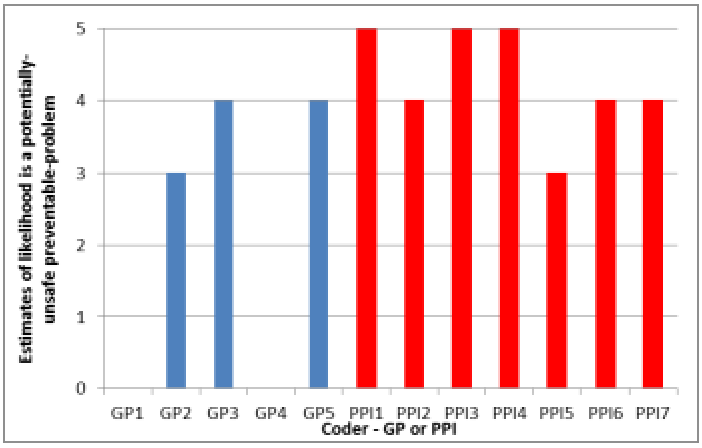

5=very likely or certain, 4=probably, 3=possibly, 2=unlikely, 1=definitely not, 0 = insufficient information or don't know

Were you able to talk about the mistake or problem with anybody working in the primary care service? "Yes, *secretary and a GP"*

Patient-reported prospect of harm: health could have been made worse had someone not noticed a problem or error

Patient-perspective problem-type code: C1.1.4 Prescribed drug when should have known contra-indicated *e.g.* patient had informed clinician of allergy, adverse reaction or it was in the records

Scenario6. GP surgery

Briefly describe the mistake or problem and how it happened. *"Went with a lump to GP. He referred me under the 2 week NICE guidelines. The communication went wrong and I chased it up myself or would have remained sat here. I ended up being diagnosed with cancer but I intervened in time."*

Could the mistake or problem have been avoided? If so how? *"Policies & procedures in place now. If you're sent an appointment that place needs to send a confirmation. That's what happened to stop it happening again."*

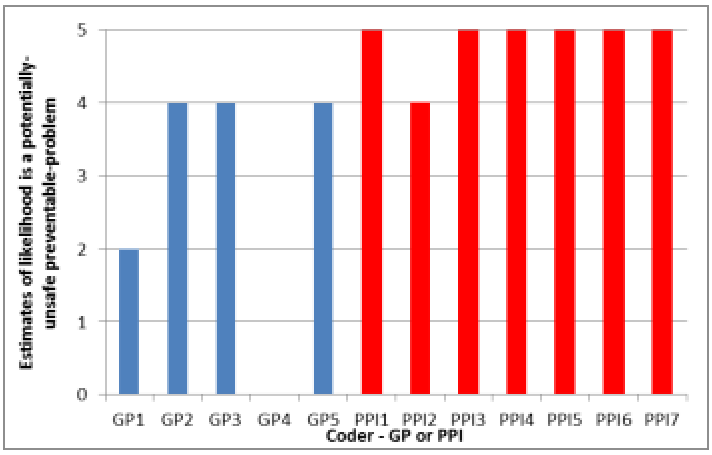

5=very likely or certain, 4=probably, 3=possibly, 2=unlikely, 1=definitely not, 0 = insufficient information or don't know

Were you able to talk about the mistake or problem with anybody working in the primary care service? *"GP investigated it as a significant event. Said if not satisfied come in and chat to us. I had apology from GP."*

Patient-reported prospect of harm: health could have been made worse had someone not noticed a problem or error

Patient-perspective problem-type code: A3. Intended referral was not sent or delayed

Scenario7. Pharmacy

Briefly describe the mistake or problem and how it happened. *"They gave me the wrong tablets and they were heart pills - beta blockers- but I thought they were sleeping pills. I looked at the patient information and thought why am I not sleeping and realised they were for people who had had a heart attack. I was taking them for 6 weeks then I phoned the doctor and he came straight away. The pharmacist no longer works there."*

Could the mistake or problem have been avoided? If so how? *"She just put up the wrong tablets. She should have dispensed the right pills as on my prescription"*

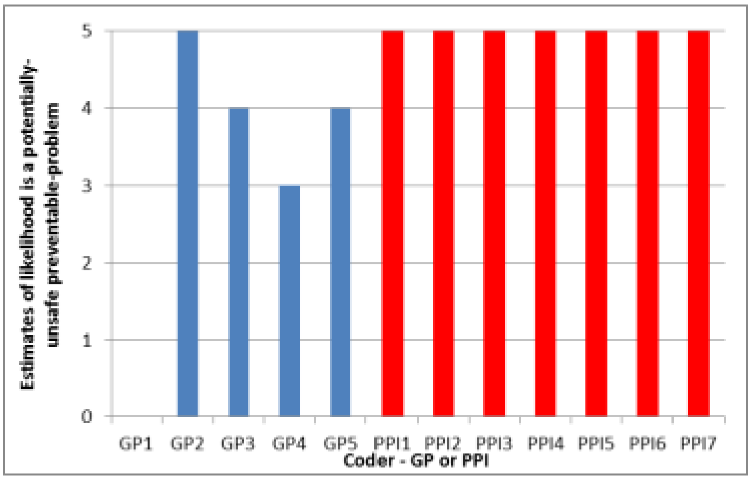

5=very likely or certain, 4=probably, 3=possibly, 2=unlikely, 1=definitely not, 0 = insufficient information or don't know

Were you able to talk about the mistake or problem with anybody working in the primary care service? *"Yes, doctor - he gave me the right ones"*

Patient-reported prospect of harm: health could have been made worse had someone not noticed a problem or error

Patient-perspective problem-type code: C1.2.1 Medication not dispensed or administered as intended or prescribed

Scenario8. Out of hours care

Briefly describe the mistake or problem and how it happened. *"Banged foot at work, hurt a lot, for few days got worse"*

Could the mistake or problem have been avoided? If so how? *"if they had listened to me properly, they didn't therefore toe got amputated for no reason"*

Were you able to talk about the mistake or problem with anybody working in the primary care service? *"Yes, triage nurse"*

Patient-reported prospect of harm: health could have been made worse had someone not noticed a problem or error

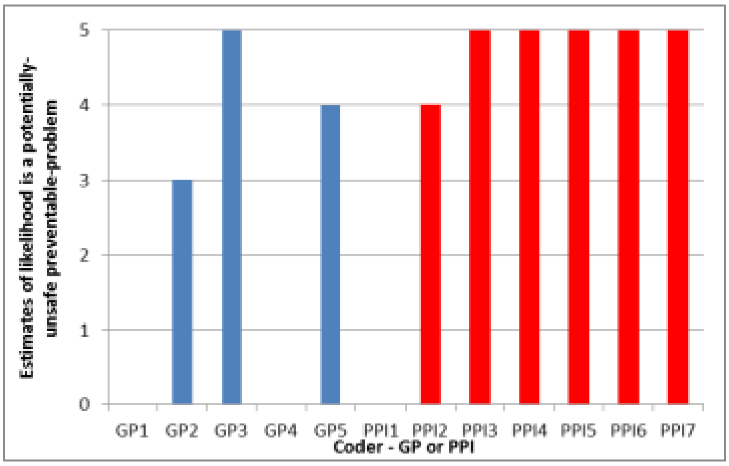

5=very likely or certain, 4=probably, 3=possibly, 2=unlikely, 1=definitely not, 0 = insufficient information or don't know

Patient-perspective problem-type code: B4. Investigation not thorough enough; D1. Clinician seemed to lack interest in the patient's health problem or did not listen carefully enough

Scenario9. GP surgery

Briefly describe the mistake or problem and how it happened. *I was started on warfarin and was fainting and bleeding rectally. I was in town the first time I passed out and did not go to hospital. The second time I went to hospital and the problem was rectified by reducing the dose."*

Could the mistake or problem have been avoided? If so how? *"by giving a smaller dose in the first place. I was told that the amount was too much. Afterwards they put me on something else instead of warfarin."*

Were you able to talk about the mistake or problem with anybody working in the primary care service? *"Yes, doctor in hospital"*

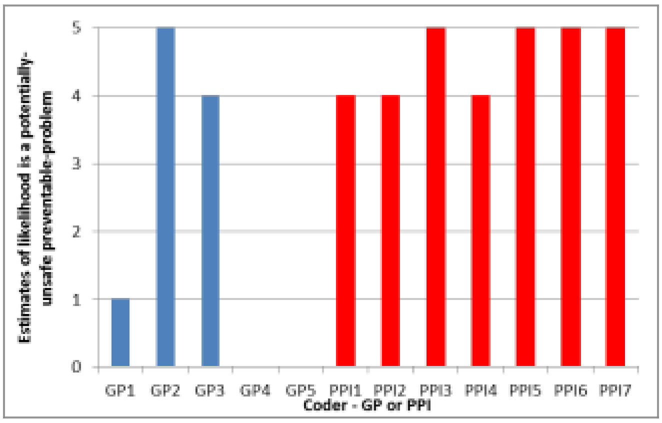

5=very likely or certain, 4=probably, 3=possibly, 2=unlikely, 1=definitely not, 0 = insufficient information or don't know

Patient-reported prospect of harm: health was actually made worse by a problem or error that could have been prevented

Patient-perspective problem-type code: C1.3.1 Wrong dose or drug or delivery method

Scenario10. GP surgery

Briefly describe the mistake or problem and how it happened. *"Couldn't get appointment at GP. Health worsened, ended up in hospital with fluid on lungs and pneumonia. Was rushed in. Heart had to be stopped and restarted."*

Could the mistake or problem have been avoided? If so how? *"Had rung for appointments and asked for doctor to telephone me 3 times. They never rang. They should have signed my prescriptions so I could have medicine and should have seen me in person"*

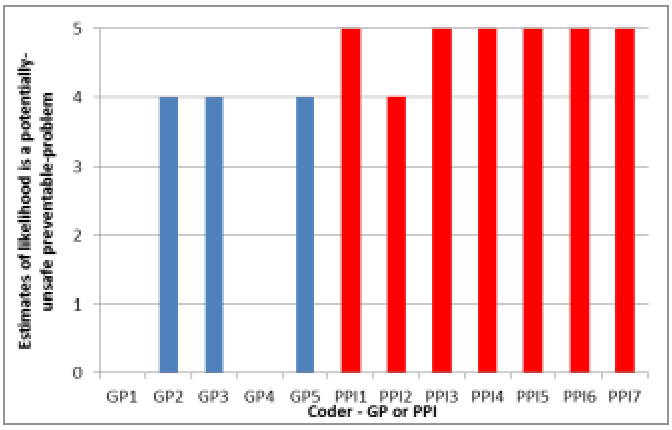

5=very likely or certain, 4=probably, 3=possibly, 2=unlikely, 1=definitely not, 0 = insufficient information or don't know

Were you able to talk about the mistake or problem with anybody working in the primary care service? *"The heart nurse from the community service complained on my behalf to the GP surgery. The chemist shop complained too about prescriptions not being signed and medicine being missed. Appointment was made at surgery to discuss with new doctor, and appointments are guaranteed as now a "supported patient"."*

Patient-reported prospect of harm: health was actually made worse by a problem or error that could have been prevented

Patient-perspective problem-type code: A5. Unable to get an appointment/other problems with making appointment; C1.3.4 Delay or failure in prescription processing

Scenario11. Dental surgery

Briefly describe the mistake or problem and how it happened. *"Dentist numbed me up to pull a wrong tooth"*

Could the mistake or problem have been avoided? If so how? *"By taking care by paying attention to his own notes"*

Were you able to talk about the mistake or problem with anybody working in the primary care service? *"Yes, the dentist himself - he was apologetic."*

Patient-reported prospect of harm: a problem or error that could have been prevented but it did not make your health worse

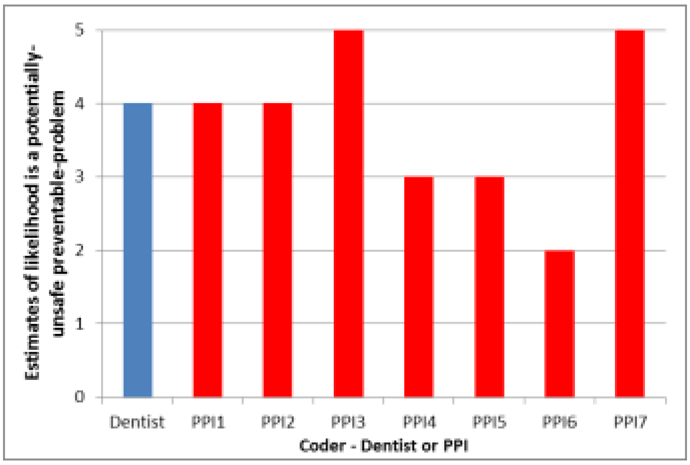

5=very likely or certain, 4=probably, 3=possibly, 2=unlikely, 1=definitely not, 0 = insufficient information or don't know

Patient-perspective problem-type code: E2.Procedure was not carried out correctly

Scenario12. GP surgery

Briefly describe the mistake or problem and how it happened. *"Discharged from hospital following knee replacement surgery, became very ill, lost 1 stone in 7 days, requested home visit from GP as seriously concerned, doctor called by phone and was very brusque, no home visit but medication changed and 6 months later started to feel better"*

Could the mistake or problem have been avoided? If so how? *"if the doctor had come to see me in person who could have made a quicker diagnosis and could have offered some much needed support during a very traumatic time"*

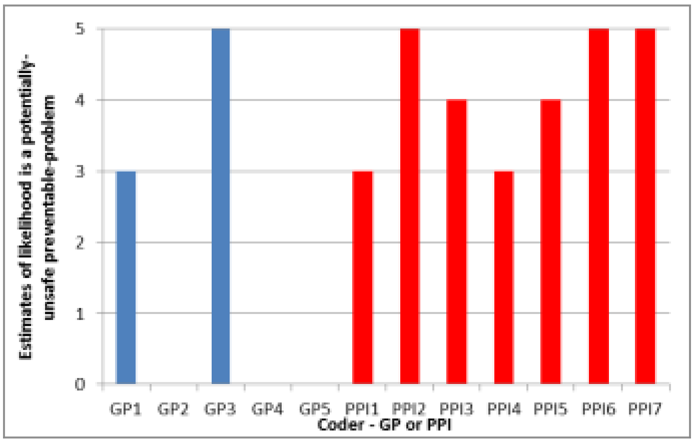

5=very likely or certain, 4=probably, 3=possibly, 2=unlikely, 1=definitely not, 0 = insufficient information or don't know

Were you able to talk about the mistake or problem with anybody working in the primary care service? *"No, I could not find anybody with whom I could discuss the problem or error"*

Patient-reported prospect of harm: suspected your health has been made worse by a problem or error that could have been prevented

Patient-perspective problem-type code: D1. Clinician seemed to lack interest in the patient's health problem or did not listen carefully enough

Scenario13. Pharmacy

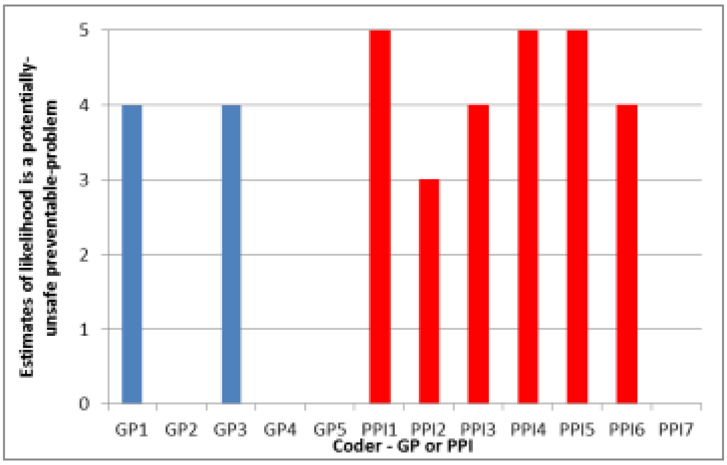

5=very likely or certain, 4=probably, 3=possibly, 2=unlikely, 1=definitely not, 0 = insufficient information or don't know

Briefly describe the mistake or problem and how it happened. *"I use a certain inhaler for COPD. I had run out without realising that I had forgotten to tick it on my repeat prescription. I spoke to the pharmacist and explained to ask him to add it for next time I picked up the repeat prescription. They agreed to do this but when I went to collect it I found that they had ordered a different medicine unrelated to COPD. I was upset because in the meantime my COPD had worsened quite quickly and was causing me distress."*

Could the mistake or problem have been avoided? If so how? *"The chemist should have made a note at the time and written down the medicine that I was asking for. If they had taken the note there and then I don't think this would have happened. I'm assuming he took a note later and failed to remember the name of the medicine correctly. We have a dreadful chemist service here."*

Were you able to talk about the mistake or problem with anybody working in the primary care service? *"No, I was so exasperated I went to my GP to order the medicine directly"*

Patient-reported prospect of harm: prompted via Q10 (Box 1 main paper)

Patient-perspective problem-type code: C1.1.5 Repeat prescription unintentionally changed

Scenario14. GP surgery

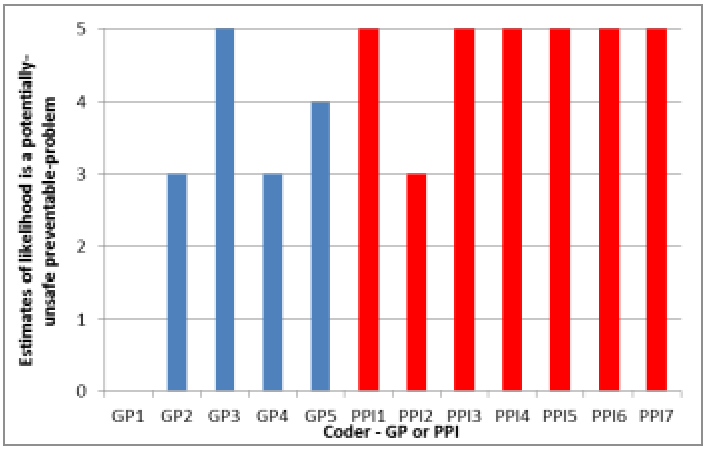

5=very likely or certain, 4=probably, 3=possibly, 2=unlikely, 1=definitely not, 0 = insufficient information or don't know

Briefly describe the mistake or problem and how it happened. *"GP misdiagnosed broken jaw, went to emergency dentist then to A&E where it was operated on and fixed"*

Could the mistake or problem have been avoided? If so how? "*if GP had diagnosed correctly initially"*

Were you able to talk about the mistake or problem with anybody working in the primary care service? *"made complaint to surgery and they wrote back apologising"*

Patient-reported prospect of harm: health was actually made worse by a problem or error that could have been prevented

Patient-perspective problem-type code: F1. Wrong/late/missed/delayed diagnosis

Scenario15. GP surgery

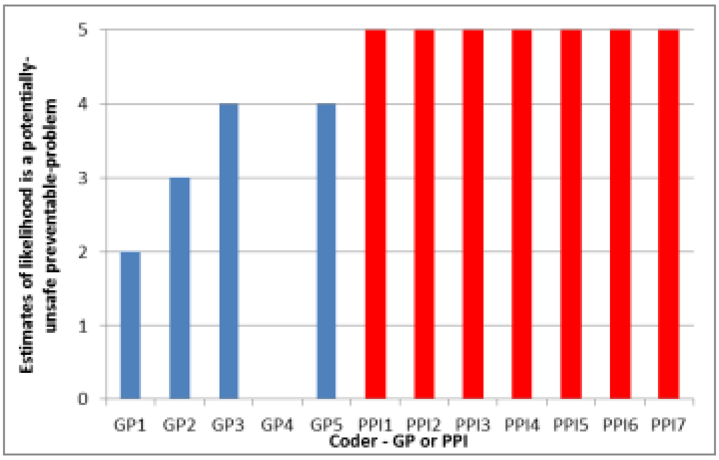

5=very likely or certain, 4=probably, 3=possibly, 2=unlikely, 1=definitely not, 0 = insufficient information or don't know

Briefly describe the mistake or problem and how it happened. *"I was having severe nose bleeds for several months and was told it was hay fever. It was cancer."*

Could the mistake or problem have been avoided? If so how? *"My GP could have sent me for a CT scan as soon as my nose bleeds started."*

Were you able to talk about the mistake or problem with anybody working in the primary care service? *"Yes, I registered with a new GP who sent me for a scan straight away which identified my cancer."*

Patient-reported prospect of harm: health was actually made worse by a problem or error that could have been prevented

Patient-perspective problem-type code: F1. Wrong/late/missed/delayed diagnosis

Scenario16. GP surgery

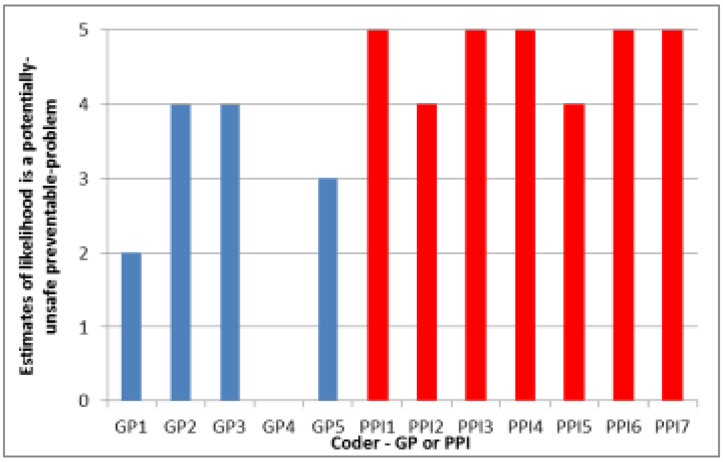

5=very likely or certain, 4=probably, 3=possibly, 2=unlikely, 1=definitely not, 0 = insufficient information or don't know

Briefly describe the mistake or problem and how it happened. *"Doctor prescribed tramadol without checking my notes. I'd already taken four pills and I rang up general enquiries at GP service to say I felt disorientated almost as if it was happening to someone else and not me. Got through to my main doctor and asked whether it was wise to take more, she said don't because you might not be alive if you do. She could see I had the wrong dose, disorientation carried on for a couple of days. It was the wrong medication."*

Could the mistake or problem have been avoided? If so how? *"if he had checked my notes to see what I can and can't take in terms of the actual medication"*

Were you able to talk about the mistake or problem with anybody working in the primary care service? *"discussed it with main doctor who said that she would give me some different pills to take to ease the pain for my trapped nerve in spine and back. She said she would speak to other doctor to see why it happened"*

Patient-reported prospect of harm: health was actually made worse by a problem or error that could have been prevented

Patient-perspective problem-type code: C1.3.1 Wrong dose or drug or delivery method

Scenario17. Out of hours care

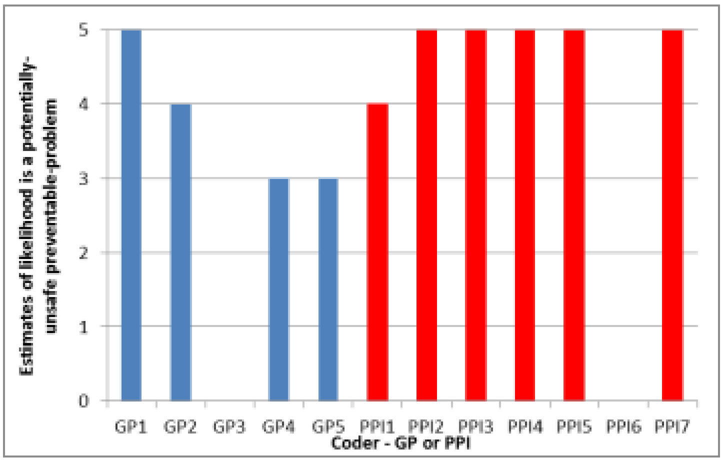

5=very likely or certain, 4=probably, 3=possibly, 2=unlikely, 1=definitely not, 0 = insufficient information or don't know

Briefly describe the mistake or problem and how it happened. *"Threatened miscarriage. Not given anti-D injection and notes were not consulted" (rhesus-negative patient)*

Could the mistake or problem have been avoided? If so how? *"Notes should have been checked"*

Were you able to talk about the mistake or problem with anybody working in the primary care service? *"Yes, hospital consultant who dealt effectively with situation"*

Patient-reported prospect of harm: there was a problem or error that could have been prevented but it did not make your health worse

Patient-perspective problem-type code: B3 Clinician did not consider patient history sufficiently/did not use patient's notes adequately

Scenario18. GP surgery

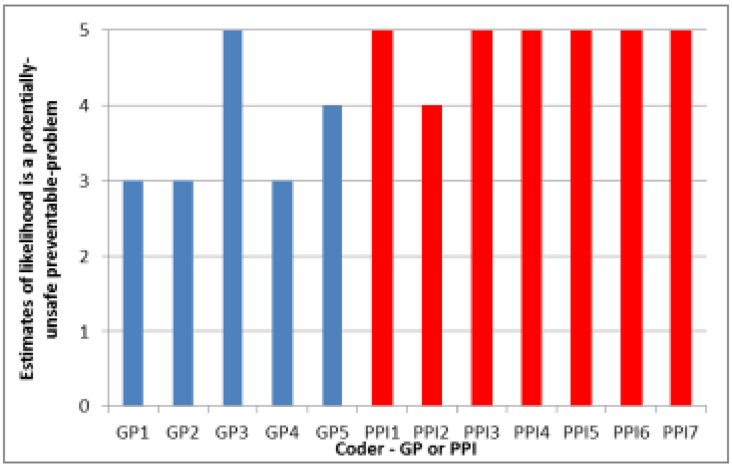

5=very likely or certain, 4=probably, 3=possibly, 2=unlikely, 1=definitely not, 0 = insufficient information or don't know

Briefly describe the mistake or problem and how it happened. *"Had retained placenta 4 weeks after giving birth. GP dismissed it and went to A&E. Had emergency surgery"*

Could the mistake or problem have been avoided? If so how? "Yes, *by improving GP competence levels"*

Were you able to talk about the mistake or problem with anybody working in the primary care service? *"No, I was too distressed to discuss the problem or error"*

Patient-reported prospect of harm: there was a problem or error that could have been prevented but it did not make your health worse

Patient-perspective problem-type code: F1. Wrong/late/missed/delayed diagnosis

Scenario19. GP surgery

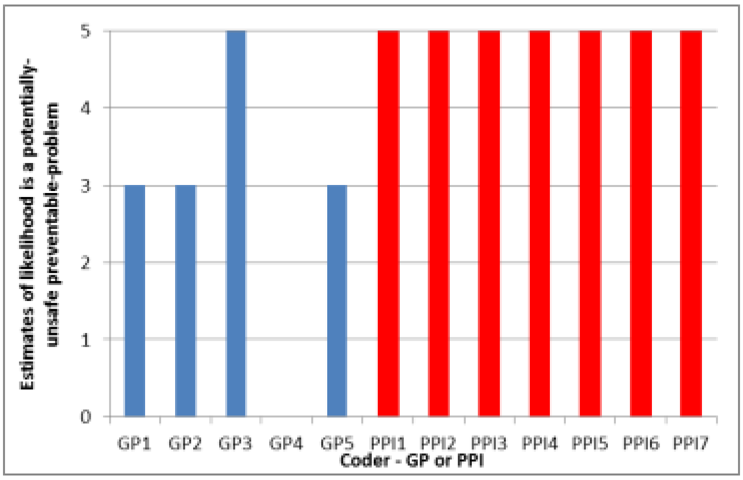

5=very likely or certain, 4=probably, 3=possibly, 2=unlikely, 1=definitely not, 0 = insufficient information or don't know

Briefly describe the mistake or problem and how it happened. *"I had a mole on my arm. It started to itch. I asked the GP if he'd look at it. He said it's fine. Two weeks later I had to see a dermatologist for a different reason. I asked him to look at the mole. He examined it through a magnifying glass. He said he couldn't tell if it was cancerous but recommended me to the local hospital. Two weeks later the hospital informed me the mole was cancerous. They took the mole out immediately. The point is that my GP didn't identify the possible cancer, it was coincidence that I went to the dermatologist who happened to be treating me at the time for a dry skin problem."*

Could the mistake or problem have been avoided? If so how? *"My GP could have examined me properly rather than just looking at the mole or he could have recommended a specialist if he didn't know what it was"*

Were you able to talk about the mistake or problem with anybody working in the primary care service? *"No, I wasn't confident that they would listen/I felt anything I say would fall on deaf ears"*

Patient-reported prospect of harm: health was actually made worse by a problem or error that could have been prevented

Patient-perspective problem-type code: F1. Wrong/late/missed/delayed diagnosis

Scenario20. GP surgery

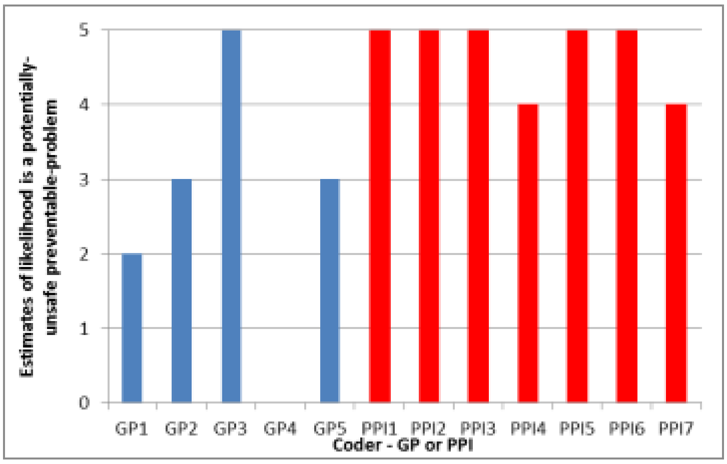

5=very likely or certain, 4=probably, 3=possibly, 2=unlikely, 1=definitely not, 0 = insufficient information or don't know

Briefly describe the mistake or problem and how it happened. *"appendix problem not diagnosed"*

Could the mistake or problem have been avoided? If so how? *"better diagnostic skills"*

Were you able to talk about the mistake or problem with anybody working in the primary care service? *"Yes, another GP who referred me to hospital"*

Patient-reported prospect of harm: health could have been made worse had someone not noticed a problem or error

Patient-perspective problem-type code: F1. Wrong/late/missed/delayed diagnosis

Scenario21. GP surgery

Briefly describe the mistake or problem and how it happened. *"I had something stuck into my ear, a cotton bud. I went to GP and they booked an appointment with a consultant. After 6 months I didn't hear anything from him. Luckily the cotton bud came out by itself, it could have been worse."*

Could the mistake or problem have been avoided? If so how? *"If I could have an appointment with a consultant he could have checked my ear canal"*

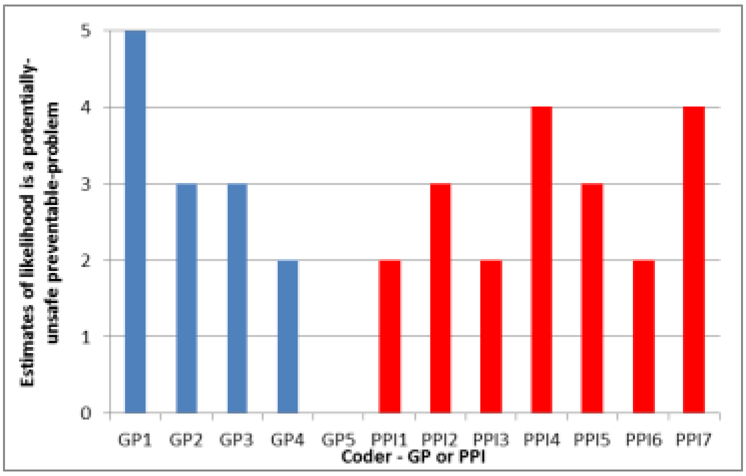

5=very likely or certain, 4=probably, 3=possibly, 2=unlikely, 1=definitely not, 0 = insufficient information or don't know

Were you able to talk about the mistake or problem with anybody working in the primary care service? *"No, I could not find anybody with whom I could discuss the problem or error"*

Patient-reported prospect of harm: health could have been made worse had someone not noticed a problem or error

Patient-perspective problem-type code: A3. Intended referral was not sent or delayed

Scenario22. A&E

Briefly describe the mistake or problem and how it happened. *"Basically told me problem was biliary spasms / colic but it was actually a hole in my stomach"*

Could the mistake or problem have been avoided? If so how? "If the doctor had taken heed of blood results - he ignored blood results - ended in emergency surgery"

Were you able to talk about the mistake or problem with anybody working in the primary care service? *"No, I was too distressed to discuss the problem or error"*

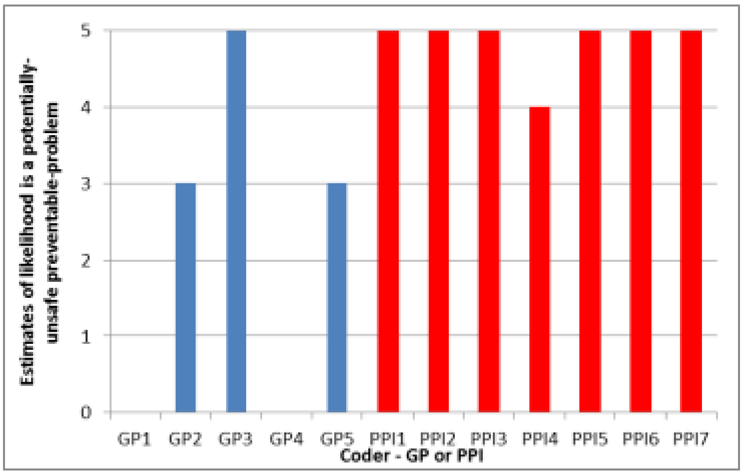

5=very likely or certain, 4=probably, 3=possibly, 2=unlikely, 1=definitely not, 0 = insufficient information or don't know

Patient-reported prospect of harm: health was actually made worse by a problem or error that could have been prevented

Patient-perspective problem-type code: F1. Wrong/late/missed/delayed diagnosis

Scenario23. GP surgery

Briefly describe the mistake or problem and how it happened. *"I have been diagnosed with bowel cancer, I knew something was wrong but over 4 visits to GP surgery over a 2 week period I was fobbed off by the GP who told me it was probably gastritis, it took 2 weeks to get a referral to a specialist"*

Could the mistake or problem have been avoided? If so how? *"I feel it was obvious from my appearance - massively distended stomach that - something serious was wrong with me, by the time I finally was referred I was seriously ill, this could have been avoided by an x-ray or quicker referral"*

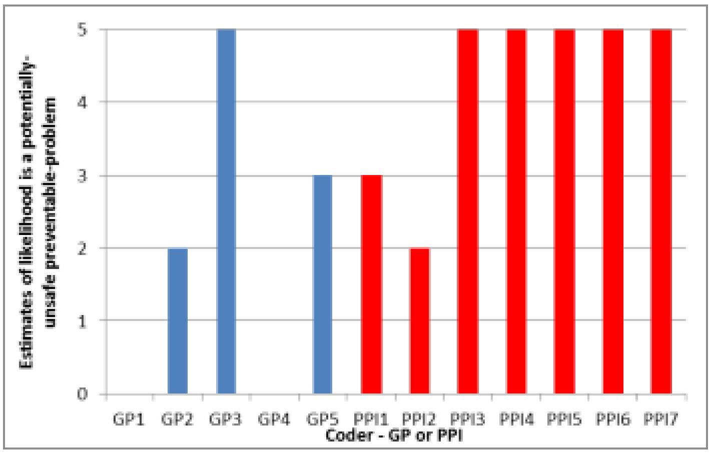

5=very likely or certain, 4=probably, 3=possibly, 2=unlikely, 1=definitely not, 0 = insufficient information or don't know

Were you able to talk about the mistake or problem with anybody working in the primary care service? *"Yes, district nurse, who told me there is a framework in place for GPs that they have to stick to whilst diagnosing issues"*

Patient-reported prospect of harm: health was actually made worse by a problem or error that could have been prevented

Patient-perspective problem-type code: F1. Wrong/late/missed/delayed diagnosis

Scenario24. GP surgery

Briefly describe the mistake or problem and how it happened. *"Low blood count not identified because doctor didn't do blood test. Taken to hospital, died and brought back to life"*

Could the mistake or problem have been avoided? If so how? *"a different drug should have been given"*

Were you able to talk about the mistake or problem with anybody working in the primary care service? *"Yes, the doctor"*

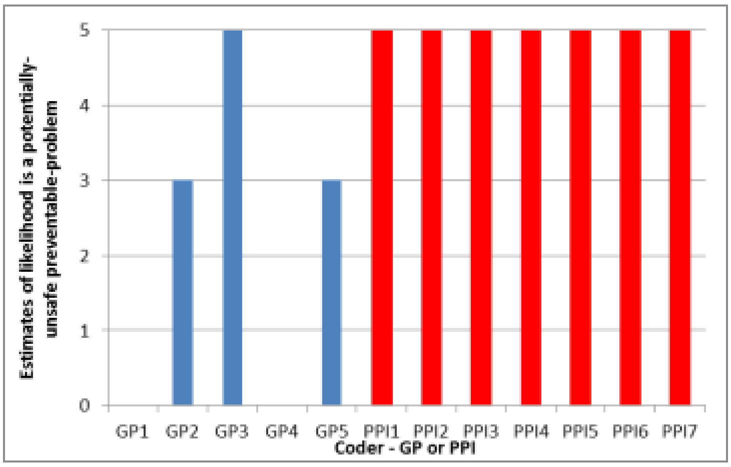

5=very likely or certain, 4=probably, 3=possibly, 2=unlikely, 1=definitely not, 0 = insufficient information or don't know

Patient-reported prospect of harm: health was actually made worse by a problem or error that could have been prevented

Patient-perspective problem-type code: B4. Investigation not thorough enough

Scenario25. GP surgery

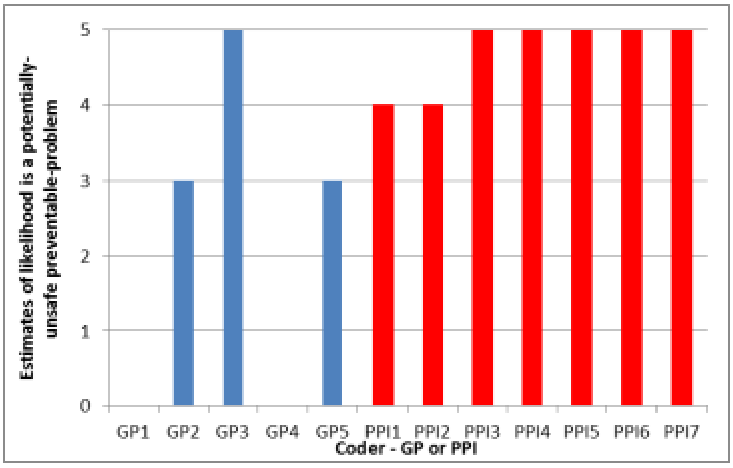

5=very likely or certain, 4=probably, 3=possibly, 2=unlikely, 1=definitely not, 0 = insufficient information or don't know

Briefly describe the mistake or problem and how it happened. *"Had lump on back and thought was an abscess. Went to GP for antibiotics was told "nothing there, it was in my head". Three days later had to have an emergency operation to remove it."*

Could the mistake or problem have been avoided? If so how? *"by correct diagnosis"*

Were you able to talk about the mistake or problem with anybody working in the primary care service? *"No, I had the opportunity but did not feel comfortable discussing the problem or error"*

Patient-reported prospect of harm: health was actually made worse by a problem or error that could have been prevented

Patient-perspective problem-type code: F1. Wrong/late/missed/delayed diagnosis

Scenario26. GP surgery

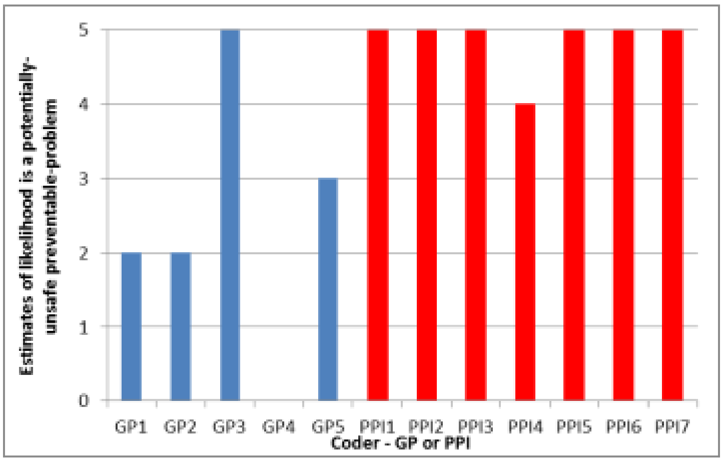

5=very likely or certain, 4=probably, 3=possibly, 2=unlikely, 1=definitely not, 0 = insufficient information or don't know

Briefly describe the mistake or problem and how it happened. "I *had gall stones and they told me it was indigestion. Pain increased over three months. Had to have an emergency operation to have my gall bladder removed. Resulted in me having damage to my liver and pancreatitis"*

Could the mistake or problem have been avoided? If so how? *"listened to me when I told them it wasn't indigestion which would have been nice. The pain felt like I was having a heart attack and not like the pain from eating something dodgy"*

Were you able to talk about the mistake or problem with anybody working in the primary care service? *"No, I could not find anybody with whom I could discuss the problem or error"*

Patient-reported prospect of harm: health was actually made worse by a problem or error that could have been prevented

Patient-perspective problem-type code: F1. Wrong/late/missed/delayed diagnosis; D1. Clinician seemed to lack interest in the patient's health problem or did not listen carefully enough

Scenario27. GP surgery

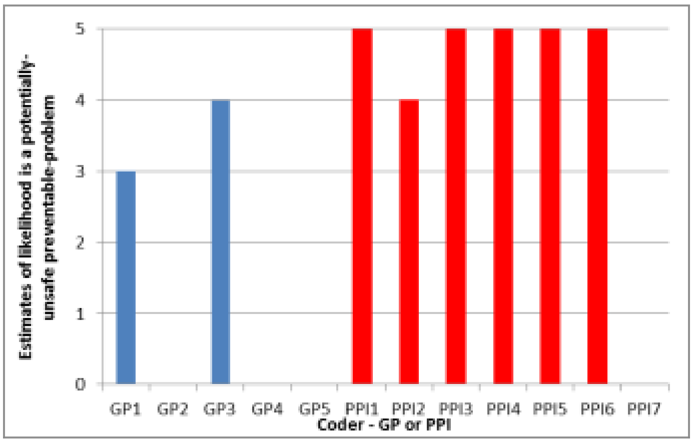

5=very likely or certain, 4=probably, 3=possibly, 2=unlikely, 1=definitely not, 0 = insufficient information or don't know

Briefly describe the mistake or problem and how it happened. *"I have arthritis and I was prescribed a medication, Diclofenac, an anti-inflammatory. After taking this, I had problems and went to the GP and had a blood test. They lost the results and I became even more ill and when I rang them, they told me I was allergic to Diclofenac and I was to stop taking it immediately. It was causing kidney failure, liver failure and high blood pressure."*

Could the mistake or problem have been avoided? If so how? *"They shouldn't have lost the results of the blood test. Later when I was feeling worse and I rang them up, they had found the results but not let me know which was another week later. They should have rung me not the other way round. That was poor communication. There should have been a better way of letting me know the results of the blood test. Luck for me, I was feeling so ill that I stopped taking the Diclofenac which they should have told me I was allergic to"*

Were you able to talk about the mistake or problem with anybody working in the primary care service? *"No, I know they're busy and there are people who need their help more than I do"*

Patient-reported prospect of harm: prompted via Q10 (Box 1 main paper)

Patient-perspective problem-type code: C1.1.3 Long term or continued prescribing without review or consideration of long term or side effects; B1. Test results lost or other problem with investigation paperwork

Scenario28. GP surgery

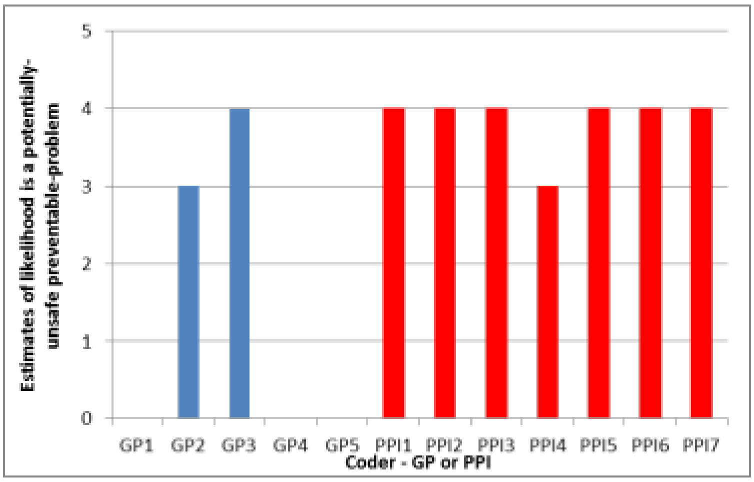

5=very likely or certain, 4=probably, 3=possibly, 2=unlikely, 1=definitely not, 0 = insufficient information or don't know

Briefly describe the mistake or problem and how it happened. *"I had stomach pains and was given the wrong medication which made it worse"*

Could the mistake or problem have been avoided? If so how? *"If I had had more tests the problem could have been avoided."*

Were you able to talk about the mistake or problem with anybody working in the primary care service? *"Yes, another doctor and they advised me to stop taking the medication"*

Patient-reported prospect of harm: health was actually made worse by a problem or error that could have been prevented

Patient-perspective problem-type code: C1.1.1 Prescribed wrong or inappropriate drug; B4. Investigation not thorough enough

Scenario29. GP surgery

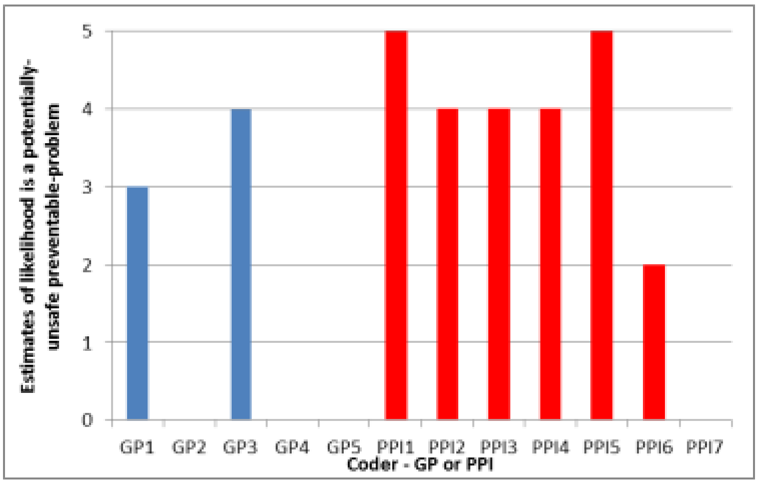

5=very likely or certain, 4=probably, 3=possibly, 2=unlikely, 1=definitely not, 0 = insufficient information or don't know

Briefly describe the mistake or problem and how it happened. *"I went to the GP and had a blood test. A month later they rang me up to tell me they had forgotten to tell me I had streptococcus and should have been on an antibiotic. In the intervening month I was ill without having taken the antibiotic"*

Could the mistake or problem have been avoided? If so how? *"Maybe they should have taken more care of their records and follow up"*

Were you able to talk about the mistake or problem with anybody working in the primary care service? *"No, I did not notice the problem or error at the time"*

Patient-reported prospect of harm: prompted via Q10 (Box 1 main paper)

Patient-perspective problem-type code: F1. Wrong/late/missed/delayed diagnosis; B1. Test results lost or other problem with investigation paperwork

Scenario30. Pharmacy

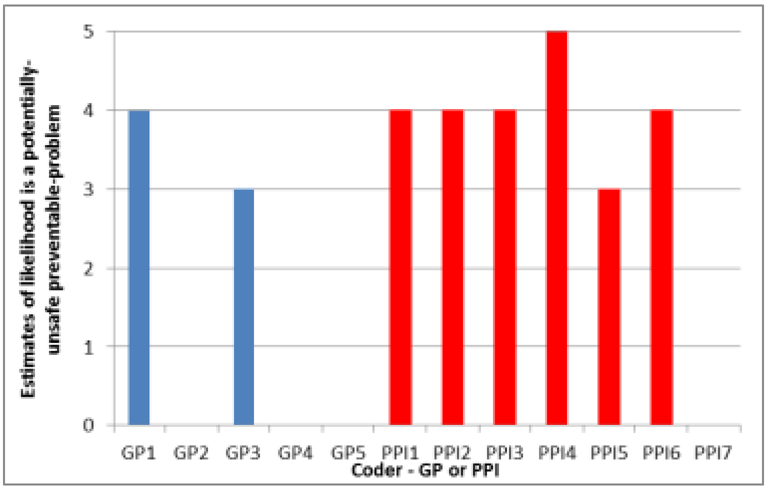

5=very likely or certain, 4=probably, 3=possibly, 2=unlikely, 1=definitely not, 0 = insufficient information or don't know

Briefly describe the mistake or problem and how it happened. *"It was routine prescription for blood pressure pills and they handed them over in a box in a stapled bag and when I got home I saw it was somebody else's medicine with my address label on. My husband took it back and they exchanged it for the correct medicine. About two weeks later we received a letter of apology which said the pharmacy had "put procedures in place so that the mistake wouldn't happen again". We were happy with that."*

Could the mistake or problem have been avoided? If so how? *"I don't know how the problem happened at the pharmacy. Perhaps somebody at the pharmacy could check each prescription before it's issued. Perhaps I could have checked it myself."*

Were you able to talk about the mistake or problem with anybody working in the primary care service? *"Yes, their response was the letter of apology."*

Patient-reported prospect of harm: prompted via Q10 (Box 1 main paper)

Patient-perspective problem-type code: C1.3.2 Being given another patient's drugs or prescription

Scenario31. Pharmacy

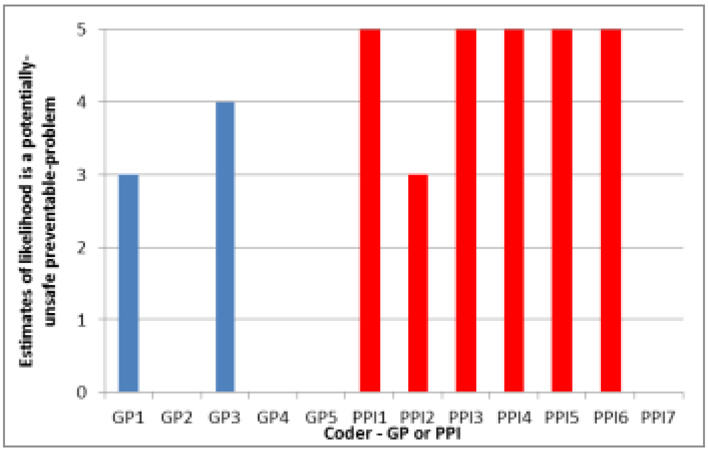

5=very likely or certain, 4=probably, 3=possibly, 2=unlikely, 1=definitely not, 0 = insufficient information or don't know

Briefly describe the mistake or problem and how it happened. *"The GP prescribed particular blood pressure tablets. The pharmacist at Boots changed the GPs prescription for a different tablet which had an adverse effect on me. It made me sick, headaches and dizziness. I went back to the GP who confirmed they were the wrong tablets and that the pharmacist isn't allowed to change a particular make of tablet. I went back to Boots and the pharmacist said they had stopped making the tablets my GP prescribed. I phoned the makers of the tablets and found that the tablets are still made. I remonstrated with the pharmacist who banned me from the shop and threatened to have me physically removed from the shop. I had been using the shop for over 40 years. I came home and phoned Boots head office and told them I would report the incident to my local newspaper and TV. I phoned the newspaper and TV wanted to film me outside the shop but a director from Boots came to my home to apologise personally and the pharmacist was forced to ring me to apologise. The pharmacist agreed that they were in breach of contract by changing the GPs prescription. When they apologised I regarded that as the end of the matter. For the last 3 months they have provided the correct tablets and on time."*

Could the mistake or problem have been avoided? If so how? *"The pharmacy is far too busy and they've exceeded their capability. Their ordering procedure means they too often run out of the correct tablets"*

Were you able to talk about the mistake or problem with anybody working in the primary care service? *"Yes, Chemist / Pharmacist, they admitted that previous medicine was wrong*

Patient-reported prospect of harm: prompted via Q10 (Box 1 main paper)

Patient-perspective problem-type code: C1.2.1 Medication not dispensed or administered as intended or prescribed

Scenario32. Pharmacy

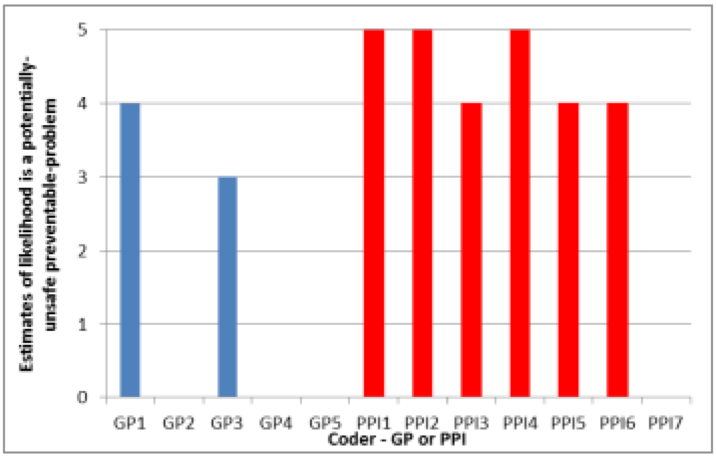

5=very likely or certain, 4=probably, 3=possibly, 2=unlikely, 1=definitely not, 0 = insufficient information or don't know

Briefly describe the mistake or problem and how it happened. *"Wrong prescription tablets issued in error, name of patient was correct but the tablets were totally incorrect."*

Could the mistake or problem have been avoided? If so how? "*Pharmacy should have taken more care"*

Were you able to talk about the mistake or problem with anybody working in the primary care service? "Yes, *spoke to pharmacist and correct prescription was issued"*

Patient-reported prospect of harm: prompted via Q10 (Box 1 main paper)

Patient-perspective problem-type code: C1.2.1 Medication not dispensed or administered as intended or prescribed

Scenario33. GP surgery

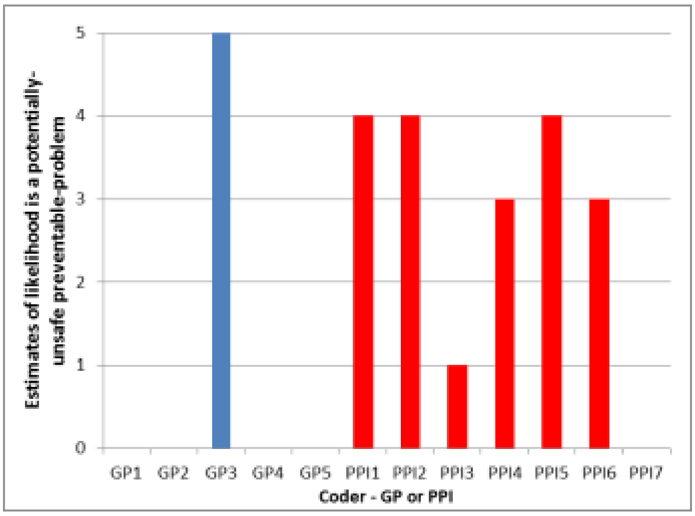

5=very likely or certain, 4=probably, 3=possibly, 2=unlikely, 1=definitely not, 0 = insufficient information or don't know

Briefly describe the mistake or problem and how it happened. *"had ear problem and GP provided treatment for 2 years but no response to medication. Within one month of being referred and treated by specialist the problem cleared up"*

Could the mistake or problem have been avoided? If so how? "*by earlier referral to specialist"*

Were you able to talk about the mistake or problem with anybody working in the primary care service? *"No, I could not find anybody with whom I could discuss the problem or error"*

Patient-reported prospect of harm: prompted via Q10 (Box 1 main paper)

Patient-perspective problem-type code: B5. Not referred when patient felt was needed

Patient reported scenarios occurring during the past 12 months that clinicians scored as higher likelihood to be a potentially-unsafe preventable-problem in primary care (median score is higher than "possibly" and at least 2 clinicians gave a score or one clinician scored "very likely or certain") from the pilot study (reference 24)

Scenario34. GP surgery

Briefly describe the mistake or problem and how it happened. *"Prescription drug, anti-inflammatory for arthritis, caused acute stomach pains & violent vomiting. Repeat prescription for twelve years without any discussion."*

Could the mistake or problem have been avoided? If so how? "*Possible discussion about dangers of continuous taking of prescription drugs, which in the event were stopped after the incident."*

Were you able to talk about the mistake or problem with anybody working in the primary care service? *"No I did not notice the mistake or problem at the time"*

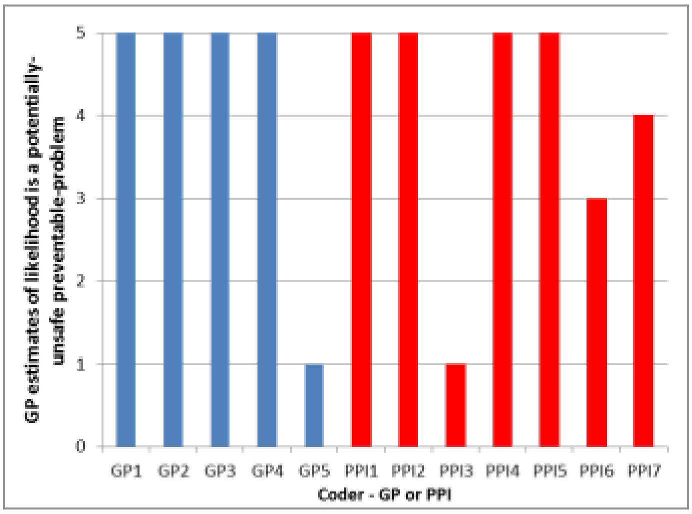

5=very likely or certain, 4=probably, 3=possibly, 2=unlikely, 1=definitely not, 0 = insufficient information or don't know

Patient-reported prospect of harm: suspected your health has been made worse by a problem or error that could have been prevented

Patient-perspective problem-type code: C1.1.3 Long term or continued prescribing without review or consideration of long term or side effects

Scenario35. GP surgery

Briefly describe the mistake or problem and how it happened. *"Insulin type was changed by specialist but previous insulin prescribed by GP as notes had not been updated"*

Could the mistake or problem have been avoided? If so how? "*Yes GP notes should have been updated with new medication"*

Were you able to talk about the mistake or problem with anybody working in the primary care service? *"Practice manager resolved the problem and apologised"*

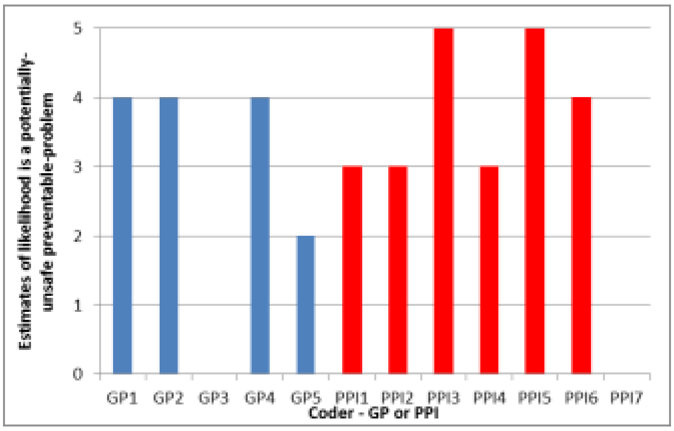

5=very likely or certain, 4=probably, 3=possibly, 2=unlikely, 1=definitely not, 0 = insufficient information or don't know

Patient-reported prospect of harm: prompted via Q10 (Box 1 main paper)

Patient-perspective problem-type code: A2. Incorrect notes/inadequate notes/notes not kept up to date; C1.1.6 Out of date repeat prescription mistakenly re-issued

Scenario36. GP surgery

Briefly describe the mistake or problem and how it happened. *"Two out of three Doctors not listening to what I was asking; April I had two big bleeds from my Penis, Doctor 1 did a test and gave antibiotics. Went to 2nd Doctor for Diabetic check and told him of problem - nothing except another test come back in ten days. Went to the third doctor who said the test didn't show anything but when I mentioned my feelings about a problem, he look and said yes you do have a problem. In 2 weeks I was in having tests and 3 operations for cancer."*

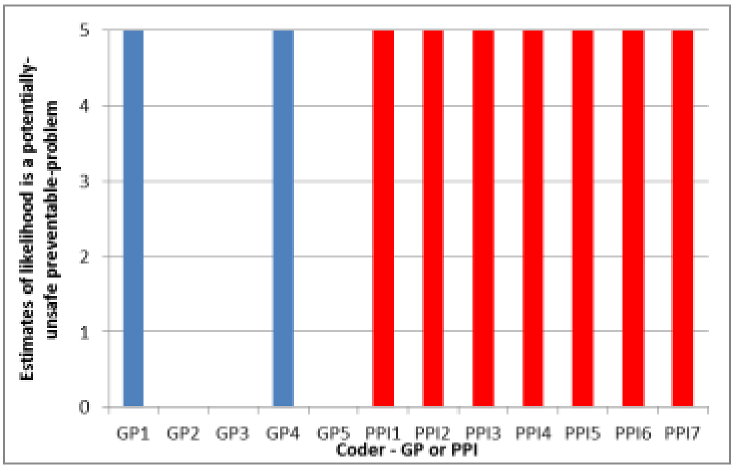

5=very likely or certain, 4=probably, 3=possibly, 2=unlikely, 1=definitely not, 0 = insufficient information or don't know

Could the mistake or problem have been avoided? If so how? "*Listen to me"*

Were you able to talk about the mistake or problem with anybody working in the primary care service? *"No, I could not find anybody with whom I could discuss the mistake or problem (The third doctor was amazing with me. He said to keep in touch and if I had any problems to ring him and he still wants me to ring him after my three operations.)"*

Patient-reported prospect of harm: suspected your health has been made worse by a problem or error that could have been prevented

Patient-perspective problem-type code: D1. Clinician seemed to lack interest in the patient's health problem or did not listen carefully enough; F1. Wrong/late/missed/delayed diagnosis

Scenario37. GP surgery

Briefly describe the mistake or problem and how it happened. *"Changed diabetes medication to an alternative which my notes from 1980's should show I respond badly to"*

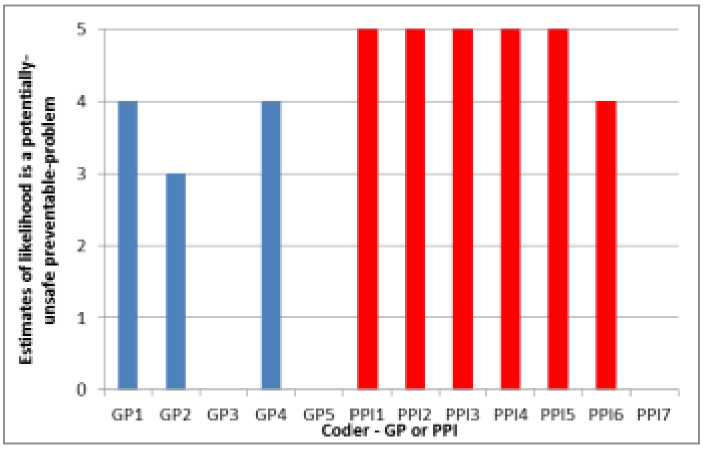

5=very likely or certain, 4=probably, 3=possibly, 2=unlikely, 1=definitely not, 0 = insufficient information or don't know

Could the mistake or problem have been avoided? If so how? "*Read the notes on every medication change but unfortunately that is unrealistic under the time restrictions on GP's. Put early notes on-line and flag medication allergies/problems."*

Were you able to talk about the mistake or problem with anybody working in the primary care service? *"Yes, my own GP who had returned from holiday"*

Patient-reported prospect of harm: suspected your health has been made worse by a problem or error that could have been prevented

Patient-perspective problem-type code: C1.1.4 Prescribed drug when should have known contra-indicated *e.g.* patient had informed clinician of allergy, adverse reaction or it was in the records

Scenario38. GP surgery

Briefly describe the mistake or problem and how it happened. *"Told the GP the medication was making my hair fall out & he kept me on it for another 3 months. I had to see another GP to get him to change my medication. In the meantime I have lost 3/4 of my hair. Not sure if it will ever grow back."*

Could the mistake or problem have been avoided? If so how? "*yes, by the GP listening to what I was saying."*

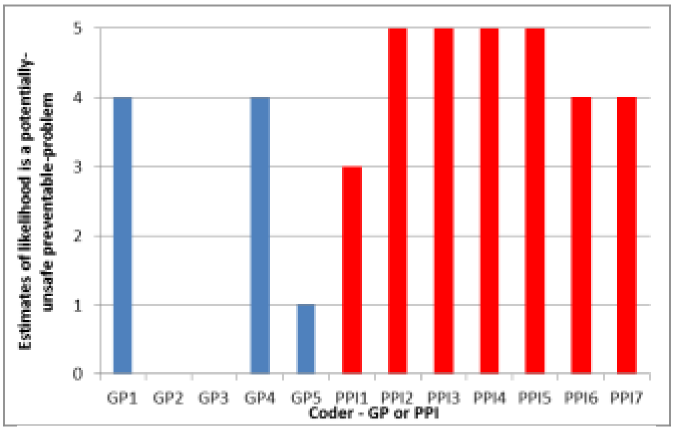

5=very likely or certain, 4=probably, 3=possibly, 2=unlikely, 1=definitely not, 0 = insufficient information or don't know

Were you able to talk about the mistake or problem with anybody working in the primary care service? *"Yes, GP"*

Patient-reported prospect of harm: suspected your health has been made worse by a problem or error that could have been prevented

Patient-perspective problem-type code: D1. Clinician seemed to lack interest in the patient's health problem or did not listen carefully enough; C1.1.3 Long term or continued prescribing without review or consideration of long term or side effects

Scenario39. GP surgery

Briefly describe the mistake or problem and how it happened. *"Successfully treated for prostate cancer 2006 but suffered some loss of sexual performance; Viagra recommended BUT I take isosorbide nitrate for a following heart attack; the two are contradictory and could produce further heart problems. A routine diabetes check-up at which the sexual problem was discussed saw an automatic prescribing of Viagra; obviously without reference to my medical records."*

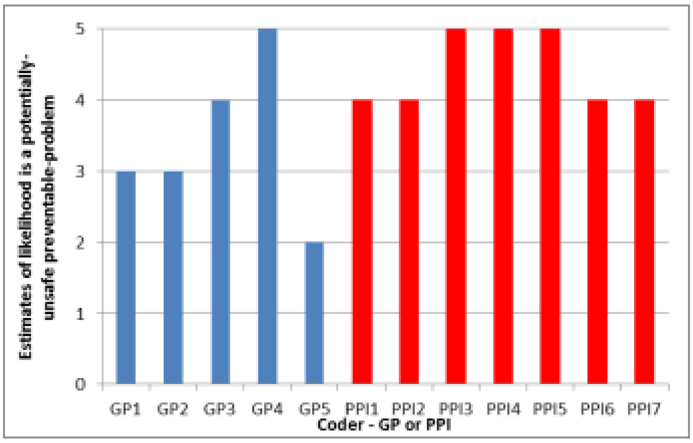

5=very likely or certain, 4=probably, 3=possibly, 2=unlikely, 1=definitely not, 0 = insufficient information or don't know

Could the mistake or problem have been avoided? If so how? "*Read the medical notes."*

Were you able to talk about the mistake or problem with anybody working in the primary care service? *"No; I felt I was going to cause trouble"*

Patient-reported prospect of harm: prompted via Q10 (Box 1 main paper)

Patient-perspective problem-type code: C1.1.1 Prescribed wrong or inappropriate drug

Scenario40. GP surgery

Briefly describe the mistake or problem and how it happened. *"I was given steroids for a chest infection but not alerted to the fact they make your sugars go massively high! Within a few hours I was high and not able to bring them down, fearing a DKA I headed for the hospital to correct a very easily avoidable issue. I also attended my GP 6 years ago to be given strong antacids for pain in my stomach that was actually a DKA I was admitted to hospital a few hours later! The GP never even suggested it could be linked to my diabetes and as it was my first DKA I had no idea that's how they can feel"*

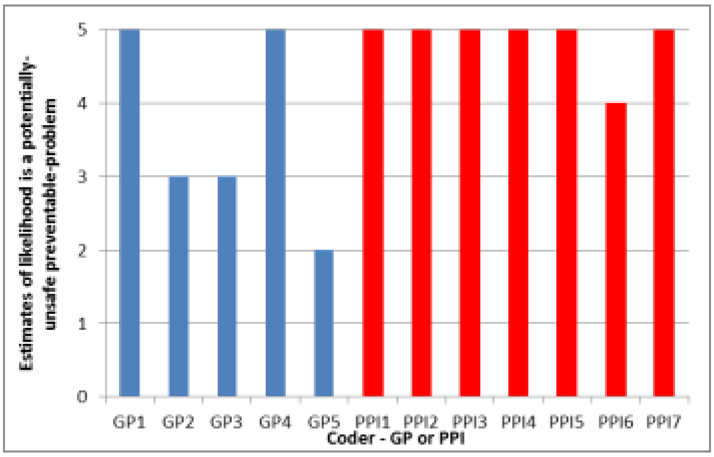

5=very likely or certain, 4=probably, 3=possibly, 2=unlikely, 1=definitely not, 0 = insufficient information or don't know

Could the mistake or problem have been avoided? If so how? "*Both could have been avoided The steroids - if the prescribing nurse had considered my diabetes I'd have been given proper advice as to how to deal with them as a diabetic or given different meds. The DKA simple questions or explanation as to how DKAs can present would have made me family and the doctor realise I was in trouble."*

Were you able to talk about the mistake or problem with anybody working in the primary care service? *"I wrote a letter to the surgery concerning the steroids anonymously to alert them of my concern and the DKA. I was too poorly to even consider seeking correction or explanation"*

Patient-reported prospect of harm: health was actually made worse by a problem or error that could have been prevented

Patient-perspective problem-type code: C1.1.4 Prescribed drug when should have known contra-indicated *e.g.* patient had informed clinician of allergy, adverse reaction or it was in the records; E3. Incorrect advice/no advice given by clinician

Scenario41. GP surgery

Briefly describe the mistake or problem and how it happened. *"reception staff making clinical decisions which were at odds with what had been discussed with my GP"*

Could the mistake or problem have been avoided? If so how? "*Yes, reception staff shouldn't be making clinical decisions"*

Were you able to talk about the mistake or problem with anybody working in the primary care service? *"No, had the opportunity but did not feel comfortable to discuss the mistake or problem"*

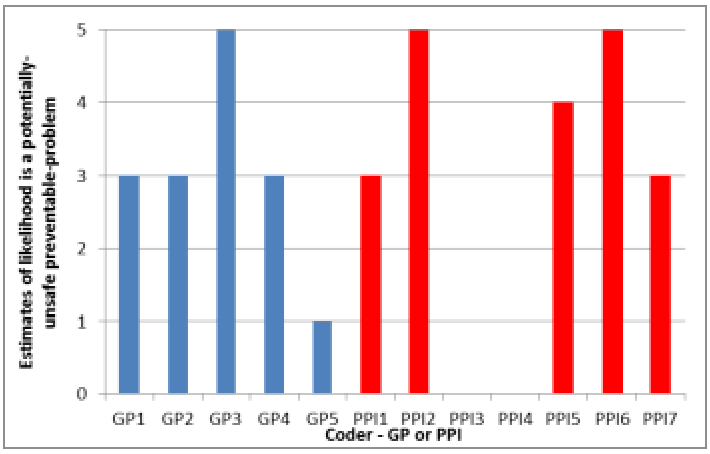

5=very likely or certain, 4=probably, 3=possibly, 2=unlikely, 1=definitely not, 0 = insufficient information or don't know

Patient-reported prospect of harm: suspected your health has been made worse by a problem or error that could have been prevented

Patient-perspective problem-type code: E1. Administrative staff seemed to make clinical decisions

Scenario42. Pharmacist

Briefly describe the mistake or problem and how it happened. *"I was given a medicine belonging to somebody else as part of my monthly repeat prescription"*

Could the mistake or problem have been avoided? If so how? "*More care and attention when checking"*

Were you able to talk about the mistake or problem with anybody working in the primary care service? *"Yes, pharmacist"*

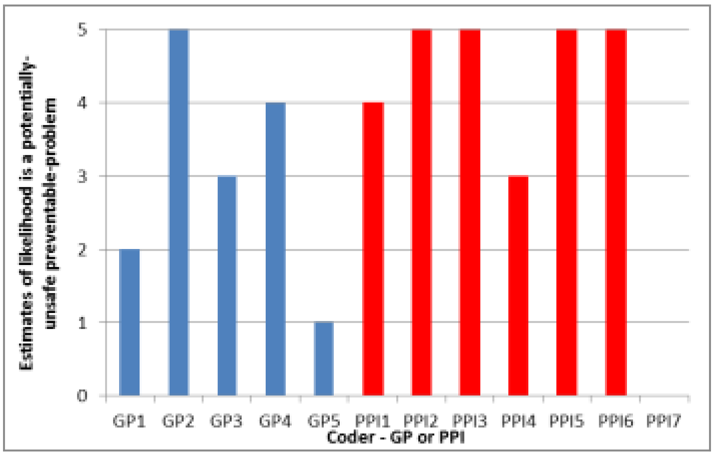

Patient-reported prospect of harm: prompted via Q10 (Box 1 main paper)

5=very likely or certain, 4=probably, 3=possibly, 2=unlikely, 1=definitely not, 0 = insufficient information or don't know

Patient-perspective problem-type code: C1.3.3 Wrong or inadequate advice about drug effects or how to use

Scenario43. GP surgery

Briefly describe the mistake or problem and how it happened. *"Poor diabetic annual review, foot check not correctly done just tested my foot pulses and nothing else"*

Could the mistake or problem have been avoided? If so how? "*Better training of staff"*

Were you able to talk about the mistake or problem with anybody working in the primary care service? *"No, had the opportunity but did not feel comfortable to discuss the mistake or problem"*

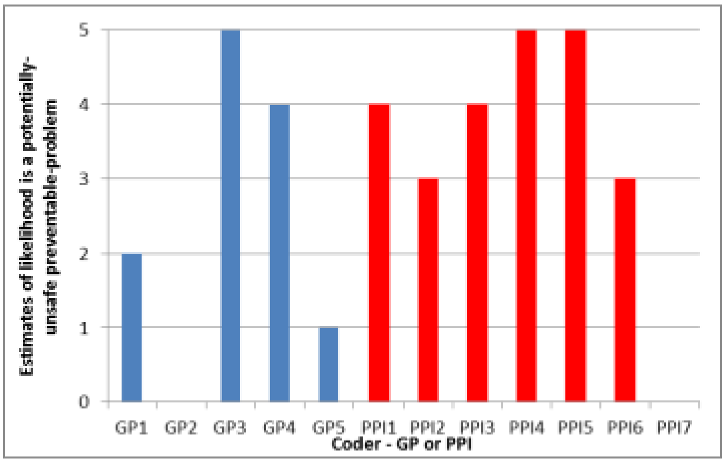

Patient-reported prospect of harm: suspected your health has been made worse by a problem or error that could have been prevented

5=very likely or certain, 4=probably, 3=possibly, 2=unlikely, 1=definitely not, 0 = insufficient information or don't know

Patient-perspective problem-type code: E2. Procedure was not carried out correctly

Scenario44. GP surgery

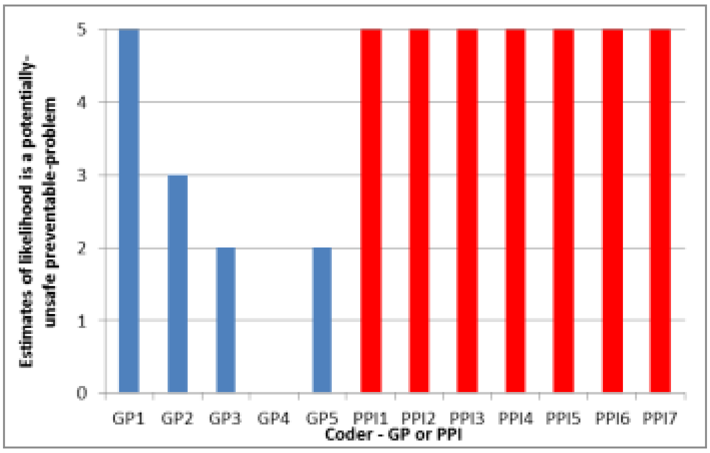

5=very likely or certain, 4=probably, 3=possibly, 2=unlikely, 1=definitely not, 0 = insufficient information or don't know

Briefly describe the mistake or problem and how it happened. *"Prior to a pain killing injection into my knee, I asked the GP who suggested the injection AND the GP who carried out the injection whether, as someone living with Type 1 diabetes, it would have any effect on my blood glucose levels. On both occasions, I was given an unequivocal No . In the event, within a few hours of the injection, my blood glucose rose significantly and remained high for several days. I felt unable to eat anything for 24 hours while I took on more and more insulin in order to bring my glucose levels down - I did not want to go to sleep that night simply because of the massive amount of insulin in my system."*

Could the mistake or problem have been avoided? If so how? "*Yes. I feel that both GPs should have a knowledge about the side effects of drugs they prescribe, administer and recommend."*

Were you able to talk about the mistake or problem with anybody working in the primary care service? *"No I could not find anybody with whom I could discuss the mistake or problem"*

Patient-reported prospect of harm: your health has been made worse by a problem or error that could have been prevented

Patient-perspective problem-type code: E3. Incorrect advice/no advice given by clinician

Scenario45. GP surgery

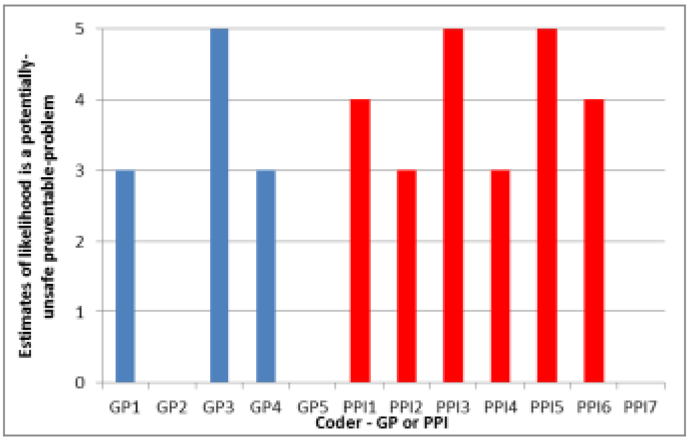

5=very likely or certain, 4=probably, 3=possibly, 2=unlikely, 1=definitely not, 0 = insufficient information or don't know

Briefly describe the mistake or problem and how it happened. *"GP completely overlooked symptoms and prescribed antibiotic after antibiotic without investigation or referral"*

Could the mistake or problem have been avoided? If so how? "*Yes by listening to history of complaints, carrying out appropriate tests instead of just giving antibiotics"*

Were you able to talk about the mistake or problem with anybody working in the primary care service? *"No I did not notice the mistake or problem at the time"*

Patient-reported prospect of harm: prompted via Q10 (Box 1 main paper)

Patient-perspective problem-type code: D1. Clinician seemed to lack interest in the patient's health problem or did not listen carefully enough; F1. Wrong/late/missed/delayed diagnosis

Scenario46. GP surgery

Briefly describe the mistake or problem and how it happened. *"Several times prescriptions have been incorrectly issued due to similar names for drugs or the same name with different strengths"*

Could the mistake or problem have been avoided? If so how? "*Yes, by more accurate or double data entry. Now solved by self-request using web systems."*

Were you able to talk about the mistake or problem with anybody working in the primary care service? "Yes, *they did not want to know or seem to care unless a formal complaint was made"*

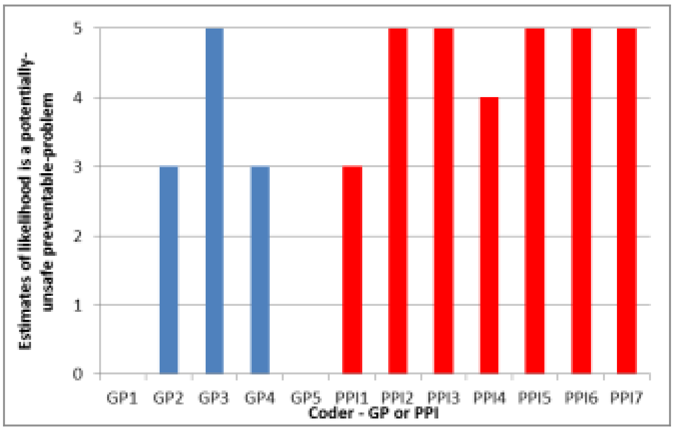

5=very likely or certain, 4=probably, 3=possibly, 2=unlikely, 1=definitely not, 0 = insufficient information or don't know

Patient-reported prospect of harm: your health has been made worse by a problem or error that could have been prevented

Patient-perspective problem-type code: C1.1.5 Repeat prescription unintentionally changed

Scenario47. GP surgery

Briefly describe the mistake or problem and how it happened. *"A simple error occurred with an incorrect prescription. When I tried to bring this to the attention of the receptionist she treated me with disdain and in a challenging manner. She then proceeded to start to read my notes aloud in the public reception area. I felt that this was unacceptable behaviour. When I tried to tackle the receptionist about her behaviour I felt as if I was under threat. It caused me to feel very stressed, frustrated and ill tempered."*

Could the mistake or problem have been avoided? If so how? "*If the receptionist had been willing to listen to what I was saying."*

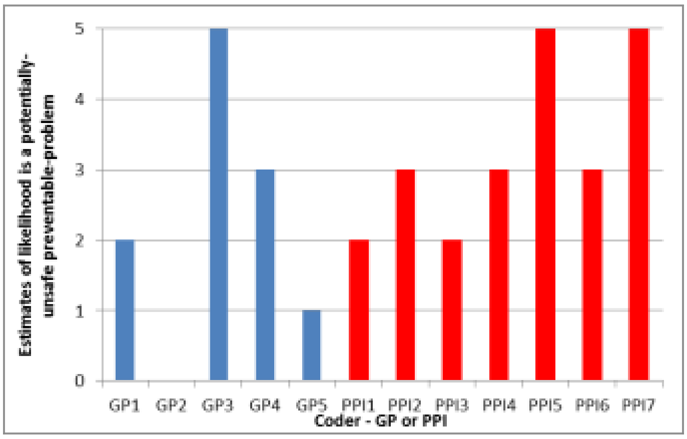

5=very likely or certain, 4=probably, 3=possibly, 2=unlikely, 1=definitely not, 0 = insufficient information or don't know

Were you able to talk about the mistake or problem with anybody working in the primary care service? *"I did speak to a lady who said she was the practice manager but I felt that they were not interested in resolving the problem"*

Patient-reported prospect of harm: suspected your health has been made worse by a problem or error that could have been prevented

Patient-perspective problem-type code: D3. Communication problem between patient and primary care staff; C1 Medication error not otherwise specified /other problem

Scenario48. GP Surgery

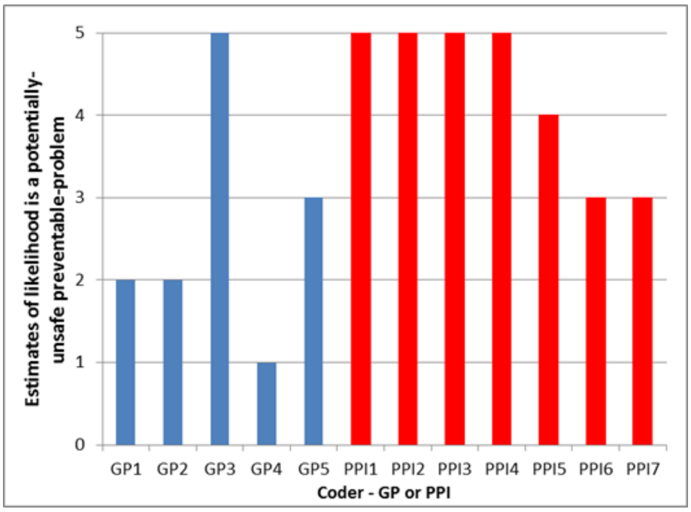

5=very likely or certain, 4=probably, 3=possibly, 2=unlikely, 1=definitely not, 0 = insufficient information or don't know

Briefly describe the mistake or problem and how it happened. "*Went to see GP because I feared the pain in one of my legs may have been Peripheral Artery Disease - hardening of the arteries, having had a (non-blood) relative who suffered from this and subsequently died - of a heart attack. Oh yes, said the GP, well, you will have it won't you? Why? I asked expecting her to say eg because you are a smoker, or maybe my age (65) or something else I wasn't aware of. But what she actually told me was 'Because you are a diabetic!' Whaaat? I exclaimed - you mean ALL diabetics will inevitably get this, and there's no way to prevent it? Yes she said and shrugged. I said 'Thanks for nothing then' and left. Instead I left, came home and went straight on-line to make an appointment with someone more sensible, which I did and after taking my leg/ankle pulses and BPs etc - he chatted to me and said he would refer me for a cardiology consultation at the hospital. This IS what I expected in the first place and now it IS being taken care of***.**"

Could the mistake or problem have been avoided? If so how? "*By training the GP properly in the first place*"

Were you able to talk about the mistake or problem with anybody working in the primary care service? "? *"I explained to GP 2 But I don't know what if anything was done about it, or how I could find that out."*

Patient-reported prospect of harm: your health has been made worse by a problem or error that could have been prevented

Patient-perspective problem-type code: D1. Clinician seemed to lack interest in the patient's health problem or did not listen carefully enough

Patient reported scenarios occurring during the past 12 months that PPIs scored as higher likelihood to be a potentially-unsafe preventable-problem in primary care compared with clinicians – Ipsos MORI survey

Scenario49. GP surgery

Briefly describe the mistake or problem and how it happened. *"I was suicidal, phoned the crisis team and they kept telling me that they couldn't see me because I wasn't under a psychiatrist and that made the situation worse"*

Could the mistake or problem have been avoided? If so how? *"they just simply had to say that they would see me"*

Were you able to talk about the mistake or problem with anybody working in the primary care service?

"No, I did not get to see a psychiatrist until about three months later"

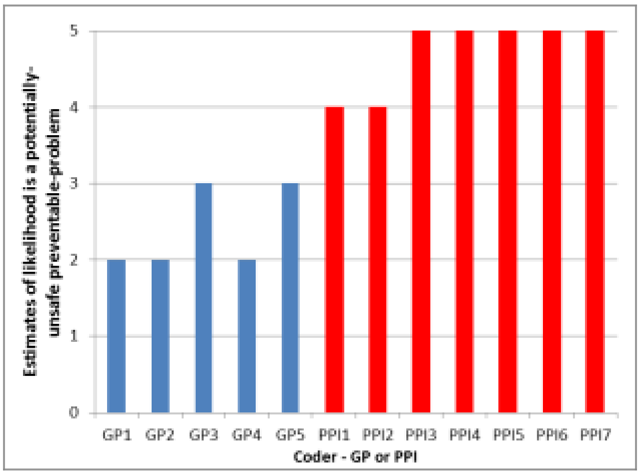

5=very likely or certain, 4=probably, 3=possibly, 2=unlikely, 1=definitely not, 0 = insufficient information or don't know

Patient-reported prospect of harm: your health has been made worse by a problem or error that could have been prevented

Patient-perspective problem-type code: A5. Unable to get an appointment/other problems with making appointment

Scenario50. Physiotherapy at GP surgery

Briefly describe the mistake or problem and how it happened. *"Broken wrist after coming off pushbike"*

Could the mistake or problem have been avoided? If so how? *"Physio caused fracture, after healing, to break again"*

Were you able to talk about the mistake or problem with anybody working in the primary care service? *"Yes, another doctor in practice"*

Patient-reported prospect of harm: your health has been made worse by a problem or error that could have been prevented

Patient-perspective problem-type code: E2.Procedure was not carried out correctly

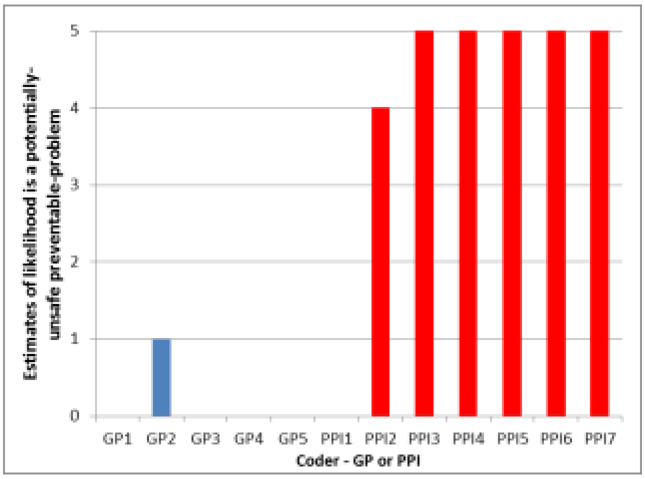

5=very likely or certain, 4=probably, 3=possibly, 2=unlikely, 1=definitely not, 0 = insufficient information or don't know

Scenario51. GP surgery

Briefly describe the mistake or problem and how it happened. *"Given some medication that brought about a nervous breakdown and crisis team attended within 4 hours. Seeing mental health social worker each week now as a result. Hearing voices and seeing things which I didn't before this medication."*

Could the mistake or problem have been avoided? If so how? *"GP could have listened more carefully and not changed my medication"*

Were you able to talk about the mistake or problem with anybody working in the primary care service? *"Yes, the crisis mental health team/the psychologist and social worker"*

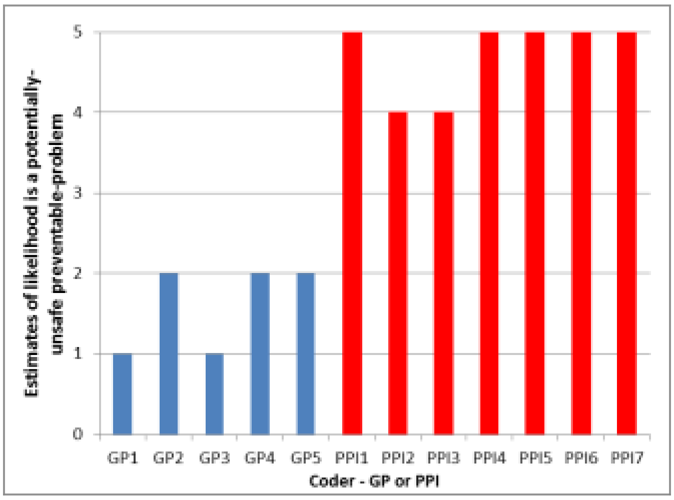

5=very likely or certain, 4=probably, 3=possibly, 2=unlikely, 1=definitely not, 0 = insufficient information or don't know

Patient-reported prospect of harm: suspected your health has been made worse by a problem or error that could have been prevented

Patient-perspective problem-type code: C1.1.2 Started new prescription or changed prescription without sufficient discussion, follow up or checks; D1. Clinician seemed to lack interest in the patient's health problem or did not listen carefully enough

Scenario52. Community mental health

Briefly describe the mistake or problem and how it happened. *"two years delay from GP referral to being able to see psychiatrist at community mental health service. Lack of access meant that he could not be diagnosed with a personality disorder trait in order for medication to be prescribed to treat the problem"*

Could the mistake or problem have been avoided? If so how? *"by referring him back to the previous psychiatrist he was with instead of worrying about boundary changes within the PCTs which are intended to manage caseloads. Basically he was out of catchment, also due to NHS cuts. Also feels these are the result of austerity and people should get social care to help"*

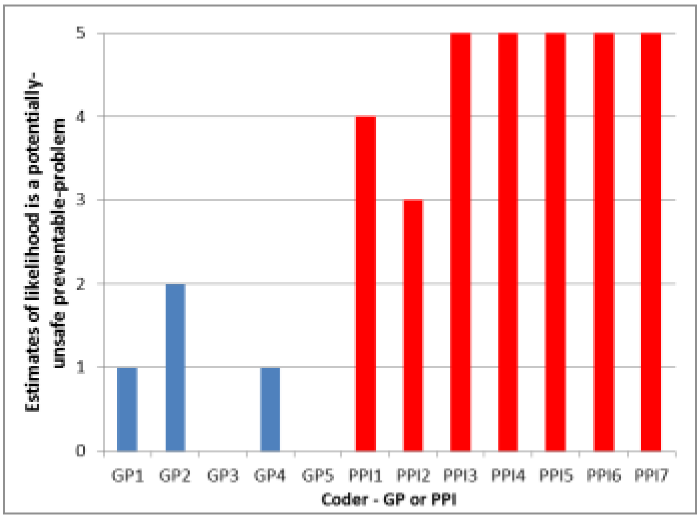

5=very likely or certain, 4=probably, 3=possibly, 2=unlikely, 1=definitely not, 0 = insufficient information or don't know

Were you able to talk about the mistake or problem with anybody working in the primary care service? *"Yes, secretary of mental health psychiatrist he should have seen but waiting for 2 years for*

Patient-reported prospect of harm: your health has been made worse by a problem or error that could have been prevented

Patient-perspective problem-type code: A5. Unable to get an appointment/other problems with making appointment

Scenario53. GP Surgery

Briefly describe the mistake or problem and how it happened. "*I had sore throat and I told the doctor it felt it would go to my chest. He prescribed a throat spray, over 2 days I felt really poorly and ended up in hospital with pneumonia"*

Could the mistake or problem have been avoided? If so how? *"GP should have prescribed antibiotics"*

Were you able to talk about the mistake or problem with anybody working in the primary care service? "*No, I was too distressed to discuss the problem or error"*

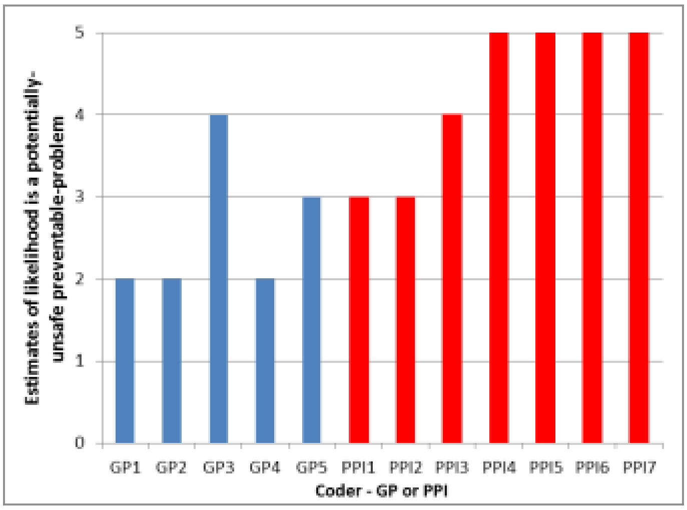

5=very likely or certain, 4=probably, 3=possibly, 2=unlikely, 1=definitely not, 0 = insufficient information or don't know

Patient-reported prospect of harm: your health has been made worse by a problem or error that could have been prevented

Patient-perspective problem-type code: G1.Wrong treatment decision

Scenario54. GP Surgery

Briefly describe the mistake or problem and how it happened. "*Got stomach pain, it was very similar to gall bladder pain but had had that removed before so couldn't be that. At first would have made an appointment with my doctor but none were available for a month. I insisted and found out it was gall bladder stones in bile duct which is serious. Total delay (in pain) 3-4 days"*

Could the mistake or problem have been avoided? If so how? *"Quicker appointment"*

Were you able to talk about the mistake or problem with anybody working in the primary care *"Yes, spoke to doctor about the problem. No apology or changes to the service"*

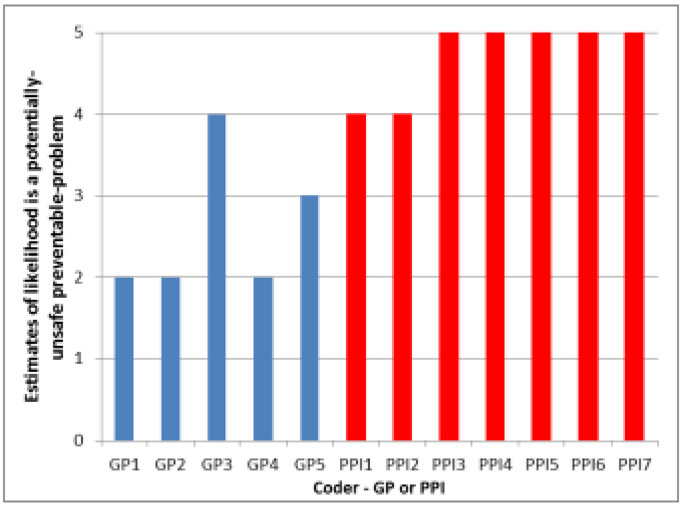

5=very likely or certain, 4=probably, 3=possibly, 2=unlikely, 1=definitely not, 0 = insufficient information or don't know

Patient-reported prospect of harm: health could have been made worse had someone not noticed a problem or error

Patient-perspective problem-type code: A5. Unable to get an appointment/other problems with making appointment

Scenario55. Dental Surgery

Briefly describe the mistake or problem and how it happened. "*Osteonecrosis of the jaw happened due to a tooth being extracted when it should not have been because of medication I was taking"*

Could the mistake or problem have been avoided? If so how? *"More knowledge on the part of the dental profession"*

Were you able to talk about the mistake or problem with anybody working in the primary care service? "*No, there was no point talking about the problem with the primary care service as the situation was beyond that"*

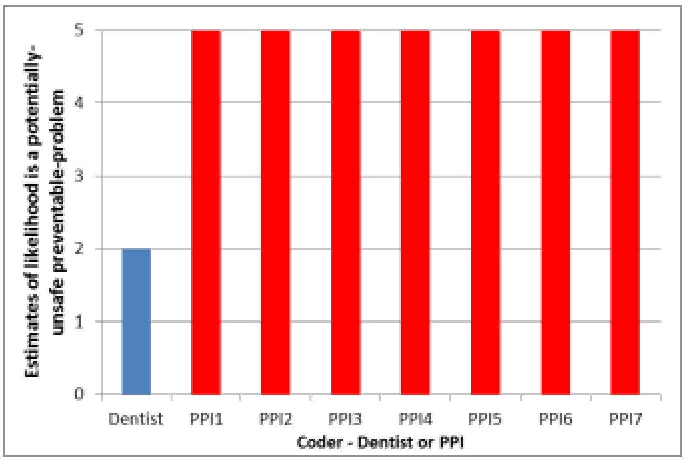

5=very likely or certain, 4=probably, 3=possibly, 2=unlikely, 1=definitely not, 0 = insufficient information or don't know

Patient-reported prospect of harm: your health has been made worse by a problem or error that could have been prevented

Patient-perspective problem-type code: G1. Wrong treatment decision

Scenario56. Physiotherapy

Briefly describe the mistake or problem and how it happened. *"GP referred to physio for shoulder pain, physio made problem worse and operation was required"*

Could the mistake or problem have been avoided? If so how? *"inexperienced physio made wrong diagnosis"*

Were you able to talk about the mistake or problem with anybody working in the primary care service? "*Yes, GP***"**

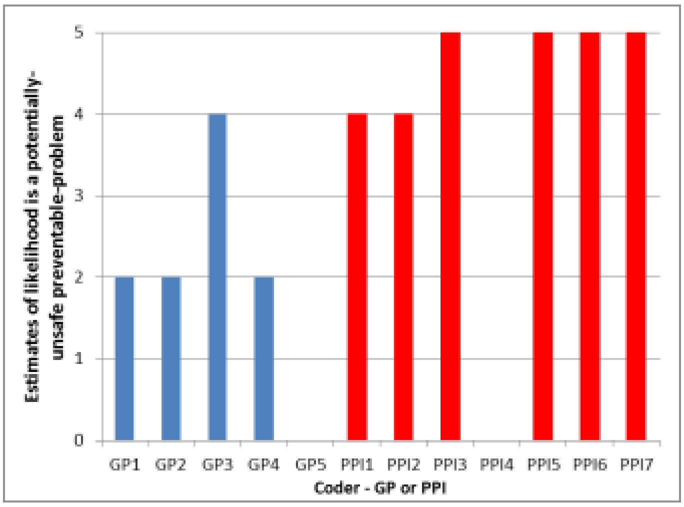

5=very likely or certain, 4=probably, 3=possibly, 2=unlikely, 1=definitely not, 0 = insufficient information or don't know

Patient-reported prospect of harm: your health has been made worse by a problem or error that could have been prevented

Patient-perspective problem-type code:

F1. Wrong/late/missed/delayed diagnosis; G1. Wrong treatment decision

Scenario57. GP Surgery

Briefly describe the mistake or problem and how it happened. *"Have thyroid problem. GP reduced medication dose without a review and caused health to deteriorate"*

Could the mistake or problem have been avoided? If so how? *"by appropriate blood test taken regularly to monitor my thyroid status"*

Were you able to talk about the mistake or problem with anybody working in the primary care service? *"Yes, GP"*

Patient-reported prospect of harm: suspected your health has been made worse by a problem or error that could have been prevented

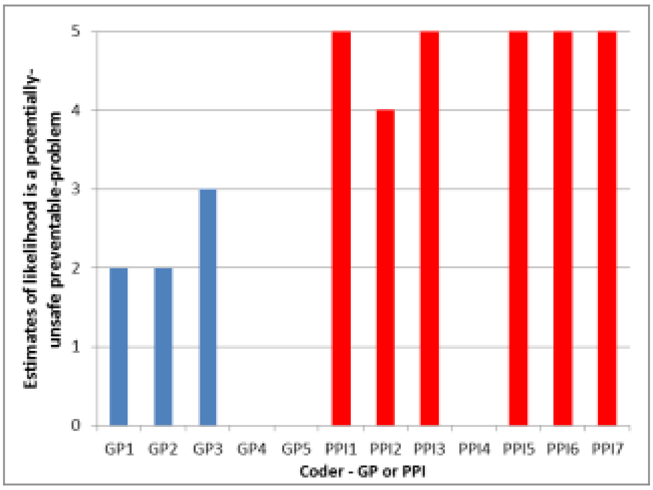

5=very likely or certain, 4=probably, 3=possibly, 2=unlikely, 1=definitely not, 0 = insufficient information or don't know

Patient-perspective problem-type code: B4. Investigation not thorough enough

Scenario58. GP Surgery

Briefly describe the mistake or problem and how it happened. *"review of drugs, GP indicated the high blood pressure, and decided to put me on blood pressure reducing tablets, which resulted in very bad side effects."*

Could the mistake or problem have been avoided? If so how? missing

Were you able to talk about the mistake or problem with anybody working in the primary care service? *"my daughter is GP, she advised me to stop taking the tablets, and monitor my own blood pressure which I did for a week and recorded it."*

Patient-reported prospect of harm: there was a problem or error that could have been prevented but it did not make your health worse

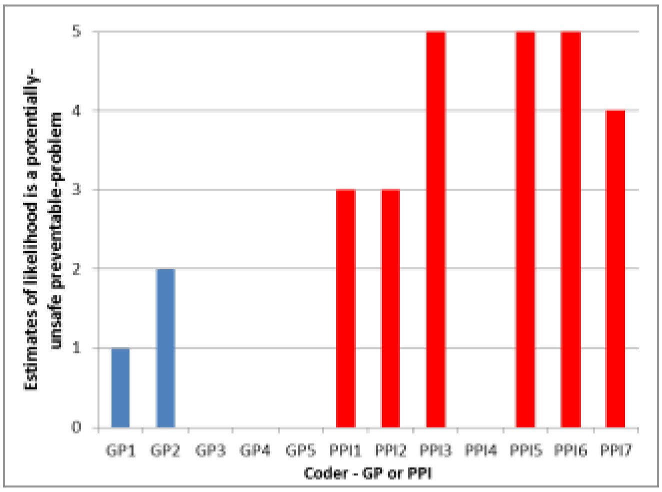

5=very likely or certain, 4=probably, 3=possibly, 2=unlikely, 1=definitely not, 0 = insufficient information or don't know

Patient-perspective problem-type code: C1 Medication error not otherwise specified /other problem

Scenario59. GP Surgery

Briefly describe the mistake or problem and how it happened. *"Complaining about severe pain in right shoulder then left shoulder for 3 years. I demanded to see a specialist. I saw a muscular skeletal specialist who diagnosed me with fibromyalgia, so I am no longer able to go to the gym now."*

Could the mistake or problem have been avoided? If so how? *"If the diagnosis had not have taken as long my overall health and fitness would not have deteriorated. It's affected my mental health and body image and I have paid over 2,000 pounds for private chiropractor"*

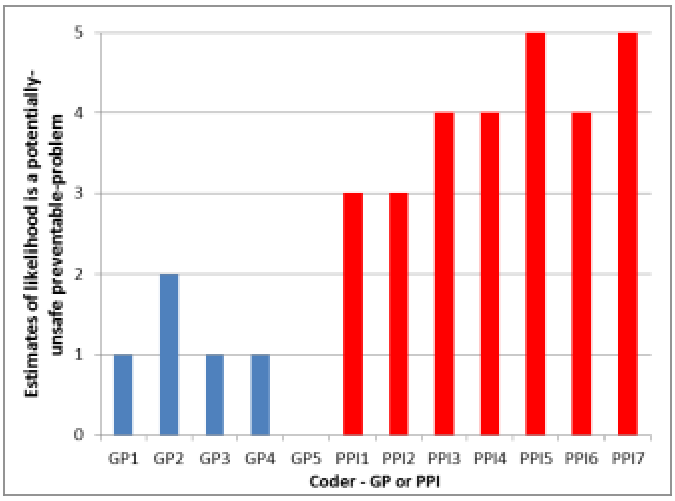

5=very likely or certain, 4=probably, 3=possibly, 2=unlikely, 1=definitely not, 0 = insufficient information or don't know

Were you able to talk about the mistake or problem with anybody working in the primary care service? *"the musculoskeletal specialist when referred listened to me and gave a diagnosis"*

Patient-reported prospect of harm: your health has been made worse by a problem or error that could have been prevented

Patient-perspective problem-type code: B5. Not referred when patient felt was needed

<u>Patient reported scenarios occurring during the past 12 months that PPIs scored as higher likelihood to be a potentially-unsafe preventable-problem in primary care compared with clinicians – pilot survey (reference 24)</u>

Scenario60. GP Surgery

Briefly describe the mistake or problem and how it happened. *"I had a severe reaction to Atorvastatin after a dose increase so much so that I was almost immobile and took 4 months to recover"*

Could the mistake or problem have been avoided? If so how? *"According to guidelines I should have been on the increased dose - it took a long time to convince the GP that I needed blood tests to find out why I couldn't walk. My GP was very hesitant to admit that I did have a reaction to statins."*

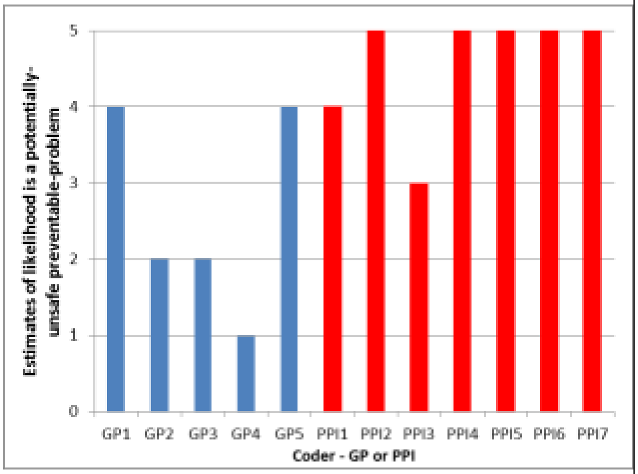

5=very likely or certain, 4=probably, 3=possibly, 2=unlikely, 1=definitely not, 0 = insufficient information or don't know

Were you able to talk about the mistake or problem with anybody working in the primary care service? *"No I could not find anybody with whom I could discuss the mistake or problem. It was not really the GPs fault per se, just took a lot of convincing that there was a problem"*

Patient-reported prospect of harm: health could have been made worse had someone not noticed a problem or error

Patient-perspective problem-type code: C1.1.3 Long term or continued prescribing without review or consideration of long term or side effects

Scenario61. GP Surgery

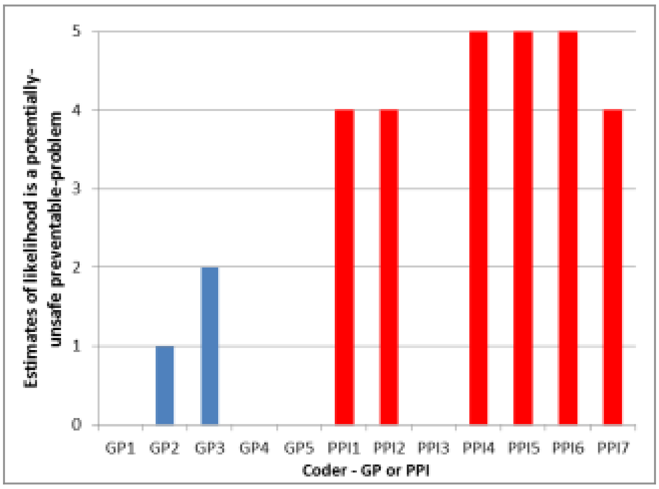

5=very likely or certain, 4=probably, 3=possibly, 2=unlikely, 1=definitely not, 0 = insufficient information or don't know

Briefly describe the mistake or problem and how it happened. *"Doctor kept saying I had vitamin deficiency B1, it turned out I had peripheral neuropathy which is very painful"*

Could the mistake or problem have been avoided? If so how? *"I just needed the proper medication to help"*

Were you able to talk about the mistake or problem with anybody working in the primary care service? *"Just saw another Doctor and she knew straight away what the problem was - she was experienced with Diabetic problems. Yes had the opportunity but did not feel comfortable to discuss the mistake or problem"*

Patient-reported prospect of harm: prompted via Q10 (Box 1 main paper)

Patient-perspective problem-type code: F1. Wrong/late/missed/delayed diagnosis

Scenario62. GP Surgery

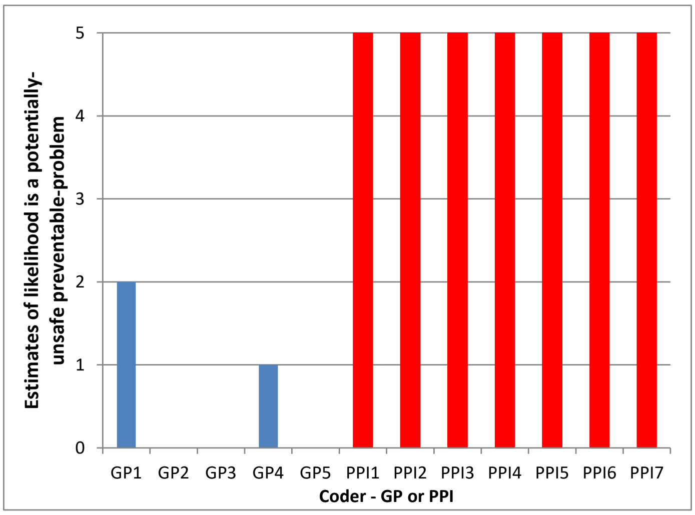

5=very likely or certain, 4=probably, 3=possibly, 2=unlikely, 1=definitely not, 0 = insufficient information or don't know

Briefly describe the mistake or problem and how it happened. *"Incapable diabetic doctor trying to take blood out the back of my hand haphazardly, not listening and resulting in me fitting and the student watching having to get help."*

Could the mistake or problem have been avoided? If so how? *"Yes. By listening to me"*

Were you able to talk about the mistake or problem with anybody working in the primary care service? *"No I could not find anybody with whom I could discuss the mistake or problem"*

Patient-reported prospect of harm: prompted via Q10 (Box 1 main paper)

Patient-perspective problem-type code: E2. Procedure was not carried out correctly; D1. Clinician seemed to lack interest in the patient's health problem or did not listen carefully enough

Scenario63. Dental Surgery

Briefly describe the mistake or problem and how it happened. *"I had an infection under my wisdom tooth. They agreed that the only way to solve the problem was to take the tooth out. They gave me an appointment to do this in 6 weeks. I am a type 1 diabetic and the infection was affecting my blood sugars and I was concerned that I would have to go to A&E if my blood sugars continued to rise due to the infection. It would have affected my health if I had not paid to go to a private dentist."*

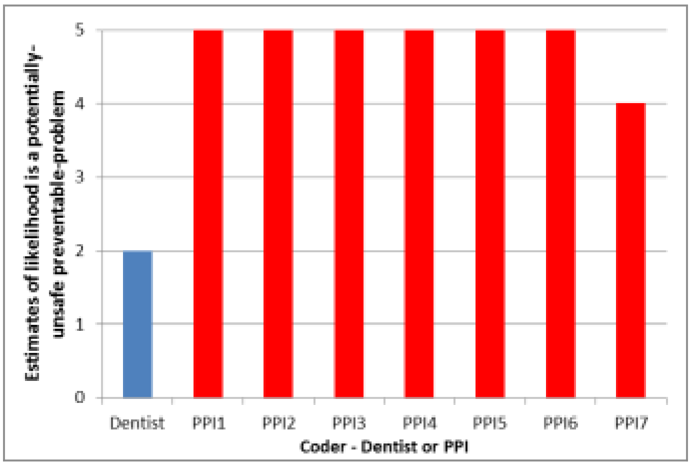

5=very likely or certain, 4=probably, 3=possibly, 2=unlikely, 1=definitely not, 0 = insufficient information or don't know

Could the mistake or problem have been avoided? If so how? *"They could have taken out the tooth straight away. I was happy to wait at the emergency dentist for them to do this."*

Were you able to talk about the mistake or problem with anybody working in the primary care service? *"I explained but they said I would have to wait. They also asked if I needed a sugary drink when I said that my sugars were high so I was too scared to eat and had not eaten in 12hrs. It was clear they didn't understand diabetes."*

Patient-reported prospect of harm: health could have been made worse had someone not noticed a problem or error

Patient-perspective problem-type code: A5. Unable to get an appointment/other problems with making appointment

Scenario64. Dental Surgery

Briefly describe the mistake or problem and how it happened. *"Caries, cavities and problem with crown not diagnosed or treated"*

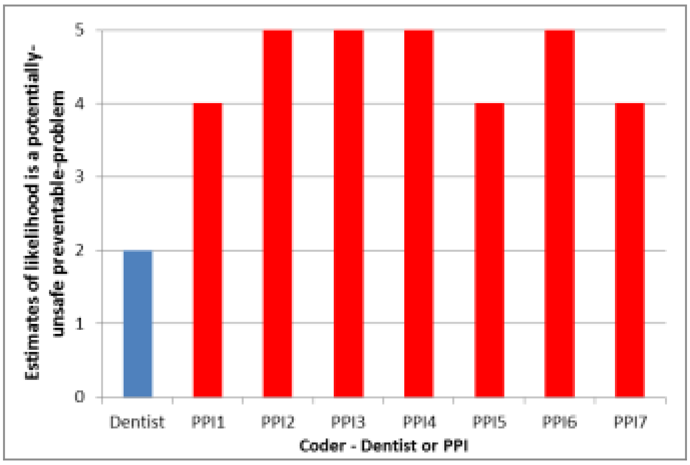

5=very likely or certain, 4=probably, 3=possibly, 2=unlikely, 1=definitely not, 0 = insufficient information or don't know

Could the mistake or problem have been avoided? If so how? *"Better dentist & not working to tight time-scale imposed by company owning dental surgery"*

Were you able to talk about the mistake or problem with anybody working in the primary care service? *"No I could not find anybody with whom I could discuss the mistake or problem"*

Patient-reported prospect of harm: prompted via Q10 (Box 1 main paper)

Patient-perspective problem-type code: C3. Problem with dental treatment or diagnosis

Scenario65. GP Surgery

Briefly describe the mistake or problem and how it happened. *"Using the summary on discharge from hospital, one GP transcribed incorrectly on to my electronic notes ie size of ovarian cyst was 7.5cms and he put 7.5 mms. Another GP requested diagnostic bone density scan but either forgot or did not record it and she ended up questioning why I had it and who requested it. She also referred me for an orthopedic consultation then said I was not funded for the steroid injection put into my swollen elbows."*

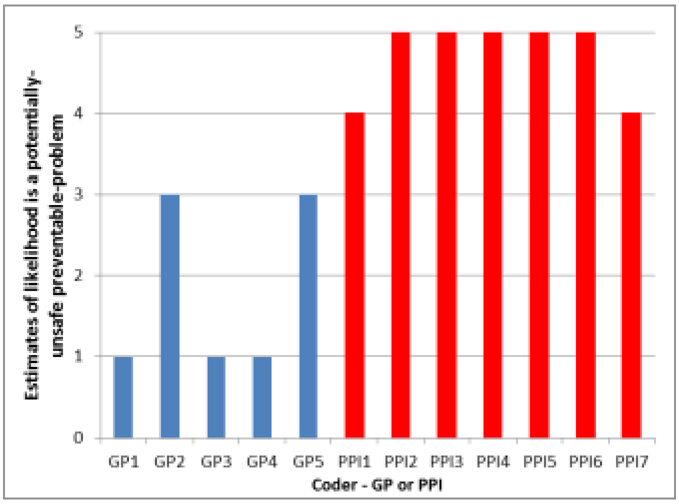

Could the mistake or problem have been avoided? If so how? *"Yes"*

5=very likely or certain, 4=probably, 3=possibly, 2=unlikely, 1=definitely not, 0 = insufficient information or don't know

Were you able to talk about the mistake or problem with anybody working in the primary care service? *"I was too scared to discuss my concerns for fear of being labelled a trouble maker"*

Patient-reported prospect of harm: health could have been made worse had someone not noticed a problem or error

Patient-perspective problem-type code: A2. Incorrect notes/inadequate notes/notes not kept up to date

Scenario66. GP Surgery

Briefly describe the mistake or problem and how it happened. *"GP prescribed pills, but then got phone call saying not to take them"*

Could the mistake or problem have been avoided? If so how? *"Not sure"*

Were you able to talk about the mistake or problem with anybody working in the primary care service? *"No I was not concerned about the problem"*

Patient-reported prospect of harm: prompted via Q10 (Box 1 main paper)

Patient-perspective problem-type code: C1. Medication problem

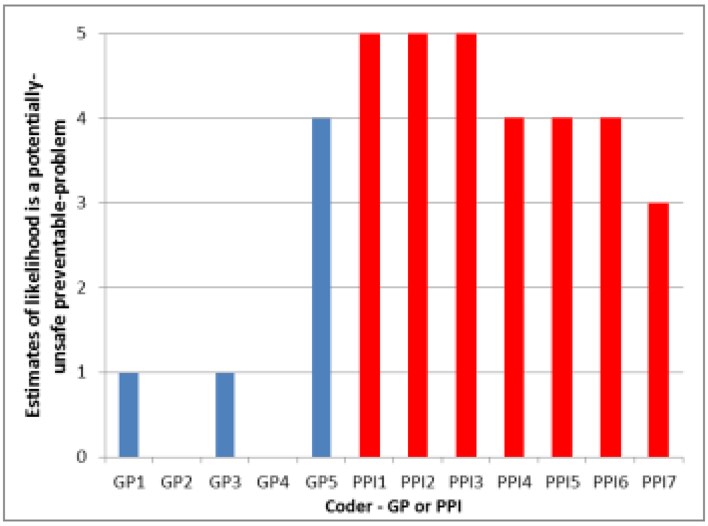

5=very likely or certain, 4=probably, 3=possibly, 2=unlikely, 1=definitely not, 0 = insufficient information or don't know

Scenario67. GP Surgery

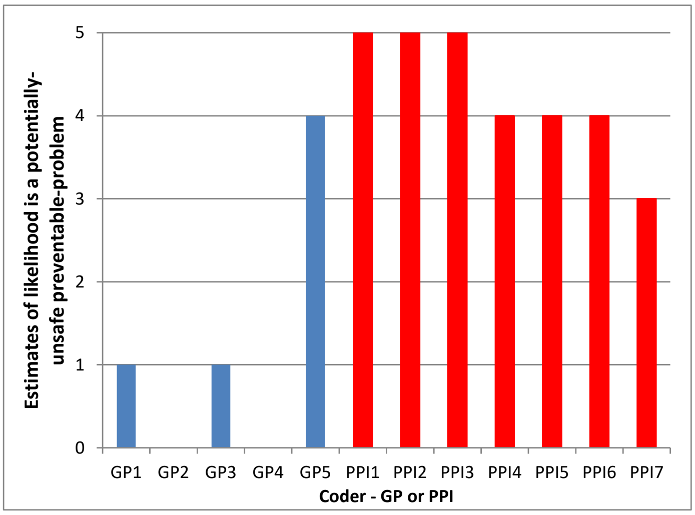

5=very likely or certain, 4=probably, 3=possibly, 2=unlikely, 1=definitely not, 0 = insufficient information or don't know

Briefly describe the mistake or problem and how it happened. *"I had a burst appendix and peritonitis, something that even a scan couldn't detect adequately. My first visit to GP was when I said I think I have appendicitis, no other symptoms only the pain. It was ten days before seeing a consultant, a further 10 days to have a scan, then 2 weeks to be told that I had a lump on my colon which is what my GP had said 5 weeks previously. It was a further 2 weeks before I had surgery."*

Could the mistake or problem have been avoided? If so how? *"If my GP had referred me for a scan immediately it would have saved 3 weeks out of the seven. It was two weeks from scan to results and I hear that is usual, but they're not looking at them for 2 weeks"*

Were you able to talk about the mistake or problem with anybody working in the primary care service? *"Had the outcome been different my widow might have pursued the matter further. The system is at fault rather than any individual."*

Patient-reported prospect of harm: your health has been made worse by a problem or error that could have been prevented

Patient-perspective problem-type code: B5. Not referred when patient felt was needed

<u>Patient reported scenarios occurring during the past 12 months that clinicians scored as definitely not a potentially-unsafe preventable-problem in primary care</u>

Scenario68: GP surgery
Description of event: Surgery arranged visits to cytology department at a local hospital; surgery did not ensure accurate visiting times came to patient
How could it be prevented: better communication between surgery and hospital
Were you able to talk about the problem or error with anybody working in the primary care service? deputy practice manager of GP surgery

Scenario69: GP surgery
Description of event: Given some medication that brought about a nervous breakdown and crisis team attended within 4 hours. Seeing mental health social worker each week now as a result. Hearing voices and seeing things which I didn't before this medication.
How could it be prevented: GP could have listened more carefully and not changed my medication.
Were you able to talk about the problem or error with anybody working in the primary care service? the crisis mental health team/the psychologist and social worker

Scenario70: Out of hours care
Description of event: Needed medication for vertigo but out of hours service sent me to A and E thinking I had had a stroke. Had all investigations for stroke over 4 hours, only for conclusion that it was indeed vertigo.
How could it be prevented: Could have ignored their pathway and had more clinical reasoning at the outset.
Were you able to talk about the problem or error with anybody working in the primary care service? No, once on the pathway you have to continue with it – no point in questioning

Scenario71: GP surgery
Description of event: mental health situation
How could it be prevented: doctor seemed unaware and worsened the condition
Were you able to talk about the problem or error with anybody working in the primary care service? attended A&E which got the doctor re-involved

Scenario72: GP surgery
Description of event: problem with process of obtaining blood test results. Lack of information and no communication
How could it be prevented: better communication
Were you able to talk about the problem or error with anybody working in the primary care service? I could not find anybody with whom I could discuss the problem or error

Scenario73: GP surgery
Description of event: I suspected I was told lies about what was on my record
How could it be prevented: My hunch is in the previous practice I belonged to someone was making up information to hit targets by saying I had test I hadn't had
Were you able to talk about the problem or error with anybody working in the primary care service? GP, it made me doubt my own sanity.

Scenario74: walk in clinic
Description of event: waiting time made the problem worse
How could it be prevented: shorter wait
Were you able to talk about the problem or error with anybody working in the primary care service? I was too distressed to discuss the problem or error

Scenario75: Dental/GP surgery
Description of event: A lump in the mouth resulted in me being referred to as out-patient at hospital. A biopsy was taken and then another was taken from the outside. Nothing has happened since then although I now have an indentation on my face. Referred back to my doctor still awaiting remedial treatment.
How could it be prevented: By my dentist who surely could have treated me properly.
Were you able to talk about the problem or error with anybody working in the primary care service? At the hospital I spoke to a consultant who kept referring to his team. The same thing happened at my doctors. It seems that no one will accept responsibility for the problem caused.

Scenario76: Dental surgery
Description of event: The dentist I was seeing had a plan for my treatment but the dentist who replaced her said the plan was "rubbish" and that I had to have private treatment. I had prepared myself for treatment according to the agreed plan but the new dentist tried to persuade me to spend £5000 on private treatment. As a result the dental treatment I need has not been done on the NHS and I have to find another dentist.
How could it be prevented: The problem was that my original dentist who I was happy with moved to the private sector within the same surgery
Were you able to talk about the problem or error with anybody working in the primary care service? I was too distressed to discuss the problem or error

Scenario77:GP surgery
Description of event: attempting to get routine screening and not being offered a convenient time as there is only a 2 week window
How could it be prevented: longer time scales and more choice over appointments
Were you able to talk about the problem or error with anybody working in the primary care service? it would require enormous effort and it was too time consuming to speak to someone

Scenario78: GP surgery
Description of event: Acne around eyes. Wanted dermatologist appointment which was not granted.
How could it be prevented: GP said only if the condition worsened.
Were you able to talk about the problem or error with anybody working in the primary care service? GP

Scenario79: GP surgery
Description of event: Doctor called me fat.
How could it be prevented: Yes, by better communication.
Were you able to talk about the problem or error with anybody working in the primary care service? I was too distressed to discuss the problem or error

Scenario80: GP surgery
Description of event: Six months ago I was referred by my GP to go for breast cancer screening for all women over 50. Since then I have not received the results of the test. I did not have any further contact so I called to check the result and was told it was with your GP. I called the GP and was told they had sent results to my home but I have not received it and six months on I have not heard.
How could it be prevented: I expected a sooner response or immediate response from the GP whatever the results but have had none I expect to call again tomorrow.
Were you able to talk about the problem or error with anybody working in the primary care service? I could not find anybody with whom I could discuss the problem or error
