# Supplementary material for: Frequency and nature of potentially harmful preventable problems in primary care from the patient’s perspective with clinician review: a population-level survey in Great Britain
Source: BMJ Open. 2018 Jun 13;8(6):e020952. doi: 10.1136/bmjopen-2017-020952 (PMC6009615; doi:10.1136/bmjopen-2017-020952)
Supplement: Author's manuscript [file bmjopen-2017-020952.draft_revisions.pdf]

BMJ Open is committed to open peer review. As part of this commitment we make the peer review history of every article we publish publicly available.

When an article is published we post the peer reviewers' comments and the authors' responses online. We also post the versions of the paper that were used during peer review. These are the versions that the peer review comments apply to.

The versions of the paper that follow are the versions that were submitted during the peer review process. They are not the versions of record or the final published versions. They should not be cited or distributed as the published version of this manuscript.

BMJ Open is an open access journal and the full, final, typeset and author-corrected version of record of the manuscript is available on our site with no access controls, subscription charges or pay-per-view fees (http://bmjopen.bmj.com).

If you have any questions on BMJ Open's open peer review process please email info.bmjopen@bmj.com

BMJ Open

The frequency and nature of potentially-harmful preventable-problems in primary care from the patient's perspective with clinician review – a population level survey in Great Britain

Journal:	*BMJ Open*
Manuscript ID	bmjopen-2017-020952
Article Type:	Research
Date Submitted by the Author:	05-Dec-2017
Complete List of Authors:	Stocks, Susan; University of Manchester, GM Patient Safety Translational Research Centre<br>Donnelly, Ailsa; Research User Group (RUG) of the NIHR Greater Manchester Primary Care Patient Safety Translational Research Centre<br>Esmail, Aneez; University of Manchester, Primary Care Research Group<br>Beresford, Joanne; Research User Group (RUG) of the NIHR Greater Manchester Primary Care Patient Safety Translational Research Centre<br>Luty, Sarah; NHS Greater Glasgow and Clyde<br>Deacon, Richard; St Gabriels Medical Centre<br>Danczak, Avril; University of Manchester, Central and South Manchester Specialty Training Programme for General Practice<br>Mann, Nicola; Research User Group (RUG) of the NIHR Greater Manchester Primary Care Patient Safety Translational Research Centre<br>Townsend, David; Research User Group (RUG) of the NIHR Greater Manchester Primary Care Patient Safety Translational Research Centre<br>Ashley, James; University of Manchester, Division of Dentistry<br>Gamble, Carolyn; Research User Group (RUG) of the NIHR Greater Manchester Primary Care Patient Safety Translational Research Centre<br>Bowie, Paul; GP Section, NHS Education for Scotland<br>Campbell, Stephen; University of Manchester, Centre for Primary Care
Keywords:	PRIMARY CARE, Quality in health care < HEALTH SERVICES ADMINISTRATION & MANAGEMENT, SOCIAL MEDICINE

SCHOLARONE™
Manuscripts

The frequency and nature of potentially-harmful preventable-problems in primary care from the patient's perspective with clinician review – a population level survey in Great Britain

S Jill Stocks *research fellow*[1], Ailsa Donnelly *public and patient involvement partner*[2], Aneez Esmail *professor*[3], Joanne Beresford *public and patient involvement partner*[2], Sarah Luty *general practitioner*[4], Richard Deacon *general practitioner*[5], Avril Danczak *general practitioner*[6], Nicola Mann *general practitioner*[2], David Townsend *general practitioner*[2], James Ashley *general dental practitioner*[7], Carolyn Gamble *public and patient involvement partner*[2], Paul Bowie *programme director*[8,9], Stephen M Campbell *professor*[3]

1. Division of Pharmacy and Optometry, University of Manchester, Manchester M13 9PL, UK
2. Research User Group (RUG) of the NIHR Greater Manchester Primary Care Patient Safety Translational Research Centre, Centre for Primary Care, Division of Population Health, Health Services Research and Primary Care, University of Manchester, Manchester M13 9PL, UK
3. NIHR Greater Manchester Primary Care Patient Safety Translational Research Centre, Centre for Primary Care, Division of Population Health, Health Services Research and Primary Care, University of Manchester, Manchester M13 9PL, UK
4. General Practitioner NHS Greater Glasgow and Clyde, Medical Directorate, NHS Education for Scotland, Glasgow G3 8BW
5. St Gabriels Medical Centre, 4, Bishops Road, Prestwich, Manchester M25 0HT, UK
6. Central and South Manchester Specialty Training Programme for General Practice, Health Education England North West (HEENWE) Education and Research Centre, Wythenshawe Hospital, Manchester M23 9LT, UK
7. Woodlands Dental Practice, Rock Ferry, Wirral, CH42 4NG, UK
8. Medical Directorate, NHS Education for Scotland, 2 Central Quay, Glasgow G3 8BW, UK.
9. Institute of Health and Wellbeing, University of Glasgow, Glasgow G12 0XH, UK

Correspondence to Jill Stocks, Division of Pharmacy and Optometry, University of Manchester, Manchester M13 9PL, UK
Email: sjstocks@btinternet.com
Tel: 01612752375
Fax: 0161 275 7600

Word count: 4789

For peer review only

For peer review only - http://bmjopen.bmj.com/site/about/guidelines.xhtml

Abstract

Objectives: To estimate the frequency of patient-perceived potentially-harmful problems occurring in primary care. To describe the type of problem, patient predictors of perceiving a problem, the primary care service involved, how the problem was discussed and patient suggestions as to how the problem might have been prevented. To compare the opinions of clinicians and members of the public as to the likelihood the patient-described scenario is potentially-harmful.

Design: population level survey

Setting: Great Britain

Participants: 3975 members of the public aged 15 years or older participating in the Ipsos MORI Face to Face Omnibus (f2f Omnibus) during April 2016

Main outcome measures: counts of patient-perceived potentially-harmful problems in the last 12 months, ranking of patient-described scenarios as to their likelihood of being potentially-harmful by primary care clinicians and members of the public

Results:

3975 of 3996 participants in the f2f Omnibus completed the relevant questions (99.5%). 300 (7.6%; 95% confidence intervals 6.7% to 8.4%) of respondents reported experiencing a potentially-harmful preventable-problem in primary care during the past 12 months. 24 (0.6%) patient-described scenarios were ranked as "at least probably" a potentially-harmful preventable-problem and 97 (2.4%) as "at least possibly" by clinicians. A substantial minority (30%) of the patient-perceived problems occurred outside general practice, particularly the dental surgery, walk in clinic, out of hours care and pharmacy. Around half the respondents discussed their concerns within primary care and this did not vary with age, gender, type of service used or clinician ranking of the problem. Those who discussed their perceived-problems appeared to maintain higher trust and confidence in primary care. The strong emphasis on the patient perspective did not identify any new types of potentially-harmful problem.

Conclusions: this study highlights the importance of reconciling clinician and patient views in relation to preventable harm in primary care.

Strengths and limitations of this study

- This is the first quantitative, population level, patient designed study examining patient-perceived potentially harmful problems in primary care purely from the patient perspective.

- The 3975 respondents were demographically similar to the GB population and had a similar level of trust in their GP as measured in the English GP Patient survey.

- Respondents were initially encouraged to express their own views on what constitutes potentially-harmful preventable-problem through the use of a non-leading screening question.

For peer review only

For peer review only - http://bmjopen.bmj.com/site/about/guidelines.xhtml

- Primary care clinicians and members of the public estimated the likelihood that, in their opinion, each patient-described scenario was a potentially-harmful preventable-problem.

- Only 69% of the patient-reported scenarios provided adequate information for clinicians to estimate the likelihood it was a potentially-harmful preventable-problem.

For peer review only

For peer review only - http://bmjopen.bmj.com/site/about/guidelines.xhtml

Background

Patients and clinicians view safety differently; patients tend to consider both serious safety problems as well as lesser causes of distress as safety concerns. (1) Patients judge quality and safety of care in terms of the ongoing care they receive over time whereas healthcare professionals may take the view that they provide high quality healthcare occasionally punctuated by discrete safety incidents and adverse events.(2) Even so patients can report medical errors accurately (3, 4) but they may have different priorities to professionals *e.g.* prioritising psychological and emotional harm over technical errors.(5) Given these differences the patient's approach to preventing safety problems may differ from clinicians, particularly if they believe clinicians to be responsible for the problem rather than the institutional system.(6, 7) Patient safety in primary care is rarely evaluated from the patient's perspective (8) whereas involving patients in identifying errors and reducing harm is common in secondary care.(3,9-11) A more participatory role for patients is advocated as a way to improve safety (12) suggesting a need for patients and professionals to be cognisant of each other's expectations and understanding of safety.

Estimates of the frequency of patient safety problems in primary care are generally from the clinician's perspective and range from less than 1 to 24 per 100 consultations or record review.(13-15) Some studies have quantified patient safety problems in primary care from the patient's perspective (6, 7, 16-18) However, quantitative patient-reported data from the UK is sparse; this may be partly due to the lack of a valid and reliable instrument for measuring safety in primary care from the patient's perspective.(19) Less than 1% of reports to the National Reporting and Learning System (NRLS) in England and Wales originate from primary care (20), probably reflecting under-reporting, but patients cannot make reports directly to the NRLS (21, 22). A European survey in 2013 found that 43% of UK respondents felt that it was "likely" that patients could be harmed by non-hospital healthcare and a recent survey of the UK public found that 21% of respondents reported experiencing a potentially-harmful preventable-problem in primary care within the past 12 months. (23, 24) These surveys suggest large differences between patients and clinicians in their beliefs about potentially-harmful problems in primary care, but this has not been examined at the population level. The PREOS-PC questionnaire has reported qualitatively on patient perceptions of safety in English general practices finding that patient recommendations for safer health care included improvements in patient- centred communication, continuity of care, timely appointments, technical quality of care, active monitoring, teamwork, health records and practice environment.(25, 26)

We aimed to quantify and describe patient-perceived potentially-harmful preventable-problems occurring in UK primary care. We also wanted to explore the differences in opinion between primary care professionals and the public regarding the potential for harm in the patient-described scenarios. Our approach aimed to capture the true patient perspective through extensive public and patient involvement (PPI); the study was conceived, co-designed and implemented by a team of three members of the public and one researcher.(24) The specific aims of the study were to (i) survey a representative sample of the public using a recently developed survey designed from the patient perspective (24) to estimate the annual and three year frequency of patient-reported potentially-harmful preventable-problems occurring in primary care as described by patients (and reviewed by primary care clinicians as part of aim iv); (ii) describe the type of problem and identify

For peer review only - http://bmjopen.bmj.com/site/about/guidelines.xhtml

patient predictors of reporting a problem (age, gender, social class, income, employment status, ethnicity *etc.*), the primary care service involved; (iii) describe how the problem was discussed (if it was) and patient suggestions as to how it might have been prevented; (iv) describe the variation between the reporting patient, other members of the public and clinicians in their opinion as to the likelihood the patient-described scenario is a potentially-harmful preventable-problem.

Methods

The population level survey to address aims (i), (ii) and (iii)

A survey asking about potentially-harmful preventable-problems occurring in primary care has been designed and piloted with extensive PPI as described in detail elsewhere. (24) The questions from this survey (Box 1, online Appendix 1) were embedded in to the Ipsos MORI GB Face to Face Omnibus (f2f Omnibus) and used to survey a nationally and regionally representative sample of 4000 adults aged 15 or over living in private households in Great Britain between 8th and 21st April 2016 using a random sampling design described elsewhere.(27) Briefly 170-180 geographically representative sampling points were randomly selected and interviewers were required to get the interviews from a small group of streets reflecting that sampling point. (Typically an interviewer would get a completed interview from 1 in every 10 to 12 addresses.) The sample size was loosely based on the pilot study (24) which had found that 132/638 (21%) of self-selected respondents had perceived a potentially-harmful preventable-problem (although we anticipated a lower proportion when sampling from the general population). The f2f Omnibus consists of interviews in the participant's home using computer assisted personal interviewing, participation is completely voluntary and there are no incentives to take part. Respondents are free to refuse to answer any questions. The first question (Q1 Box 1) was taken from the English GP patient survey in order to compare the overall level of confidence and trust in their GP among the survey respondents with the larger sample used in the English GP patient survey.(28) The second question (Q2 Box 1) is the main screening question, those responding negatively to Q2 (*i.e.* not experienced a preventable-problem) were directed to a more specific question with a list of commonly understood patient safety events (Q10 Box 1 & online Appendix 1). If this prompted recognition of experiencing a potentially-harmful preventable-problem they were returned to Q4 (Box1). The intention of using a non-leading screening question was to encourage respondents to express their own perspective on what constitutes potentially-harmful preventable-problem rather than being directed towards existing definitions.

Coding of patient-reported scenarios to address aim (ii)

The nature of the problem described by the patient was coded at face value *i.e.* as the patient described without further interpretation, by one author (SJS) and checked by a second author (JA for dental scenarios, PB for all other scenarios) using a taxonomy developed during the pilot study that also mapped on to a previously published taxonomy for errors in general practice (24, 29, 30) (Table A, online Appendix 2). The medication-related scenarios were coded to a finer level (Table B, online Appendix 2).

Likelihood the scenario described a potentially-harmful preventable-problem to address aim (iv)

Five GPs, one general dental practitioner and 7 members of the public estimated the likelihood that, in their opinion, each patient-described scenario was a potentially-harmful preventable-problem.(24) The dental scenarios were only rated by the general dental practitioner and members

For peer review only - http://bmjopen.bmj.com/site/about/guidelines.xhtml

of the public. The raters were given the responses to Q2 and Q4 to Q9 (Box1) without any demographic information and asked to score each scenario on a 5 point scale from “very likely or certain” to “definitely not” a potentially-harmful preventable-problem. The scores were used to categorise the scenarios in to two groups according to the public or clinician-estimated likelihoods that they were a potentially-harmful preventable-problem as below (Table C, online Appendix 2).

- Higher threshold - Median score of “very likely or certain” or “probably” or at least one score of “very likely or certain”
- Lower threshold - Median score of “possibly” or at least one score of “probably” or higher

The median scores excluded responses where the raters scored “don’t know” or “insufficient information”. In order to increase the statistical power in addressing aim iv we combined all the patient-described scenarios occurring in the last 3 years with scenarios from the pilot study (24) occurring in the last 12 months. We judged this acceptable since we were using the scenarios to compare the views of the clinicians and members of the public without making any inference to the wider population.

Statistical analysis to address all aims

The 95% confidence intervals for the population means were calculated assuming a normal distribution for the sample mean. Simple cross tabulations were used to describe the data and a binary logistic regression model was used to explore whether particular types of patient (*e.g.* according to their demographics or surveyed opinions) were more likely to perceive a potentially-harmful preventable-problems and what type of scenario was more likely to be ranked as potentially harmful by clinicians and members of the public. Comparisons between demographics and outcomes for the respondents and the UK population were made using a χ^2 test. Inter-rater agreement for the ranking of the patient-described scenarios by clinicians and members of the public was assessed using a two-way random effects model single-measures intraclass correlation coefficient (ICC).(31). All analyses were done using Stata 14.

Results

The results for aim (i) are shown in Figures A to C and Table A in online appendix 3; for aim (ii) in Tables 1 & 2 and Figure 1; for aim (iii) in Tables 2 & 3 and for aim (iv) in Figure 2, Tables B and C in online appendix 3 and online Appendix 4 shows some examples of patient-reported scenarios.

Of 3996 members of the public participating in the f2f Omnibus, 3984 (99.7%) agreed to complete the questions relevant to this study and 3975 (99.5%) actually completed all the questions. Survey responders were significantly more likely to have confidence and trust in the GP seen at their last appointment than the English population (Table A, online Appendix 3) but there was no significant difference when the graded responses “yes definitely” or “yes to some extent” were combined (91% vs 92%, $P(\chi^2)$=0.2). Survey responders did not differ from the overall UK gender distribution but tended to be slightly older with small but significant differences in ethnicity and social group distributions (Table A, online Appendix 3).

The progress of the respondents through the analysis is summarised in Figures A & B in online Appendix 3. In total 300 (7.6%) of respondents reported experiencing a potentially-harmful preventable-problem during the past 12 months; of these 193 (4.9%) arose directly from the

For peer review only - http://bmjopen.bmj.com/site/about/guidelines.xhtml

screening question (Q2 Box1) and 107 (2.7%) were prompted by a list of potentially-harmful preventable-problems (Q10 Box 1, Appendix 1). Of the 193 unprompted problems (Q2 Box 1), 119 (3.0%) patients suspected, or actually believed, that their health had been made worse as a result of the problem whereas 74 (1.9%) believed that they had either noticed the problem before it had any consequences or it had had no effect on their health. A further 132 potentially-harmful preventable-problems were reported as occurring within the past 1 to 3 years (Fig A, Appendix 3) making a 3 year total of 325 (8.2%) arising only from the screening question (Q2 Box1) as there was no prompt question asking about problems over 12 months ago. The combination of an open-ended question (Q2, Box 1) and prompt question (Q10, Box 1) prioritised sensitivity over specificity (as intended) given that 21% of the perceived problems (79/379) were excluded from the analysis, mainly because the perceived problem was not preventable or did not occur in primary care (Figure A, Appendix 3).

Of the 300 patient-described scenarios occurring within the last 12 months, 207 (69%) provided information of sufficient quality for ranking by at least one clinician. Of these 207, 24 (11.6%, Table B, online Appendix 3) were considered to "at least probably" describe a potentially-harmful preventable-problem by clinicians which corresponds to an annual rate of 0.6% (applying the higher threshold above). Using the lower threshold identified 97 (46.9%) scenarios considered to "at least possibly" describe a potentially-harmful preventable-problem (annual rate of 2.4%). The corresponding frequencies for potentially-harmful preventable-problems occurring in the last 3 years were 28 (9.4%, 3 year rate of 0.7%) for the higher threshold and 124 (41.5%, 3 year rate of 3.1%) when using the lower threshold (Table B, online Appendix 3). The members of the public ranked 116 (39%) scenarios occurring in the last 12 months as "at least probably" a potentially-harmful preventable-problem (higher threshold) which included all 97 scenarios ranked as "at least possibly" by clinicians (lower threshold).

The proportion of respondents reporting a potentially-harmful preventable-problem within the last 12 months by respondent characteristics and unadjusted and adjusted odds ratios estimated by logistic regression are shown in Table 1. Those responding "no, not at all" to the question about trust and confidence in the GP (Q1 Box) were around eight times more likely to report a problem compared to those responding "yes, definitely"(Table 1). Women and rural dwellers were significantly more likely to report experiencing a potentially-harmful preventable-problem even when only including the scenarios judged to be more likely to be potentially-harmful by clinicians (Table 1). People not in employment due to a disability, self-employed or with one or more children were more likely to report a problem but not when only those scenarios judged to be more likely to be potentially-harmful by clinicians were included (Table 1).

Characteristics of the patient-reported scenarios

The types of problem occurring in the last 12 months alongside their clinician rankings are summarised in Figure 1. Generally respondents were equally likely to describe the nature of the problem as related to healthcare delivery, investigation, treatment (mainly medication), communication or lack of clinical knowledge or skills (Panel B Fig 1). Within the medication problems the most common scenarios were being prescribed a wrong, contra-indicated or inappropriate drug or the wrong dose or delivery method (Panel C Fig 1). The respondents did not identify any previously unreported types of problem and the patient-reported scenarios mapped well on to an established taxonomy of errors in primary care (Fig 1). However the prompt question (Q10)

For peer review only - http://bmjopen.bmj.com/site/about/guidelines.xhtml

particularly increased reports of scenarios related to appointments, referrals and reporting of test results suggesting that the respondents did not consider these to be potentially harmful problems in the first instance (Fig C, online Appendix 3). Table 2 provides information about the patient's response to the potentially-harmful preventable-problem and the primary care service involved. A substantial minority (30%) of problems occurred outside general practice, particularly the dental surgery, walk in clinic, out of hours care and pharmacy. Around half of the patients had discussed their problem with a primary care professional and usually this was a person who worked in the same organisation as where their problem had occurred (Table 2). There were no significant differences between patients who discussed the problem, and those who did not, according to gender (males 49% vs females 51%, $P\chi^2$=0.78), age (38% to 62% in 10 year age bands, $P\chi^2$=0.33), type of service being used (general practice 50% vs other services 50%, $P\chi^2$=0.95), working as a healthcare professional (no 56% vs yes 50% $P\chi^2$=0.44) or describing a problem ranked higher by clinicians (below lower threshold 50% vs above lower threshold 50%, $P\chi^2$=0.98). Those reporting a problem in the first instance at Q2 (Box 1) without prompting were somewhat more likely to have discussed the problem (unprompted 53% vs prompted 43%, $P\chi^2$=0.08) whereas ethnic minorities were somewhat less likely to have discussed the problem (white 51% vs other ethnicity 37%, $P\chi^2$=0.09). Patients who discussed their problem were significantly more likely to "definitely" have trust and confidence in their GP (Q1 Box 1; 61% did discuss their problem vs 39% who did not discuss their problem, $P\chi^2$<0.001). The reasons given for not discussing the problem varied but the most common reasons related to feeling uncomfortable about discussing the problem, being too distressed or ill, being unable to find the appropriate person with whom to discuss the problem or the respondent was unconcerned about the problem. The respondent's suggestions as to how the problem might have been prevented are summarised in Table 3. The most frequent suggestions revolved around quicker access to primary care and investigations and a more participatory role. They rarely identified a particular individual as the problem or made specific suggestions for improvement strategies.

Comparison of the opinions of clinicians and members of the public about the patient-reported scenarios

The total number of patient-described scenarios available for analysis was 564 (432 from the main survey last 3 years and 132 from the pilot survey in last 12 months) but only 406 (72%) patients provided sufficient information for at least one clinician to score the scenario on a 5 point scale as to the likelihood that the patient described a potentially-harmful preventable problem (Table C in online Appendix 2). The members of the public scored 426 (76%) of the scenarios. The median scores for each patient-described scenario are shown in Fig 2. Members of the public were significantly more likely to designate the patient-described scenarios as potentially-harmful preventable-problems compared with clinicians (median clinician score of 2.5, "unlikely- possibly" compared with members of the public score of 3.5, "possibly-probably"; Wilcoxon signed-rank test z=16.4, P<0.001). From the clinician perspective just 8% of the problems occurring during the past 12 months were categorised as "probably to almost certainly" potentially harmful whereas for the members of the public the corresponding proportion was 39% (Table B in online Appendix 3 using the higher threshold). The individual patient-described scenarios scored by clinicians as more likely to be a potentially-harmful preventable-problems (median score is higher than "possibly" and scored by at least 2 clinicians, or one clinician scored "very likely or certain") and the scenarios with the greatest disagreement between members of the public and clinicians (median scores differ by 3 points or more on a 5 point scale) are summarised in online Appendix 4. The single measures ICC for

For peer review only - http://bmjopen.bmj.com/site/about/guidelines.xhtml

absolute measures was 0.43 (0.38 to 0.49) for the members of the public and 0.23 (0.09 to 0.40) for clinicians, illustrating that members of the public had somewhat better agreement than clinicians. The associations between the characteristics of the patient or problem, and the clinician rankings of the likelihood it is a potentially-harmful preventable-problem are shown in Table C, online Appendix 3. Clinicians were more likely to rank scenarios as "possibly to almost certainly" potentially-harmful if they related to treatment, diagnosis or the patient was qualified as a healthcare professional (even though they were blind to this information) but for the members of the public scenarios related to treatment, investigation, clinical skills, diagnosis or where the patient had reported a problem in the first instance without prompting. Additionally members of the public were more likely to rank problems reported through the pilot survey as potentially harmful. The diagnoses (as specified by the patient) more likely to be considered a potentially-harmful preventable-problem by both clinicians and members of the public were cancer and cardiovascular problems.

Discussion

Our main finding is that 7.6% of respondents in a GB nationally representative survey of 3975 people reported experiencing a potentially-harmful preventable-problem during the past 12 months, but this proportion fell to 3% when only including those problems ranked by members of the public as "at least probably" a potentially-harmful preventable problem. The proportion fell to just 0.6% when the same exclusion criterion was based on clinician judgements. Members of the public almost always rated a scenario as more likely to be a potentially-harmful preventable-problem than the clinicians (Fig 2). It is important to address this difference in perception between patients and clinicians because respondents perceiving a safety problem were eight times more likely to lose confidence and trust in their GP (Table 1). Those who discussed their problem with a primary care professional, however, were more likely to maintain their confidence and trust, suggesting the importance of encouraging dialogue between patients and clinicians on this topic (alternatively it may be that patients with higher trust and confidence in their GP were more likely to discuss their problem). A large number of patients could potentially benefit by reconciling the patient and clinician perspective; scaling our results up to the GB adult population implies that around 3 million patients (3.8 million; 95% confidence intervals 3.3 million to 4.2 million) believe that they have experienced a potentially-harmful preventable-problem during the past 12 months and 1.5 million (1.2 million to 1.8 million) believe or suspect that their health has been made worse as a result.

Despite this high level of disagreement between clinicians and members of the public in terms of the likelihood that the reported scenarios were a potentially harmful problem, there was agreement on other aspects of the patient-described scenarios. The scenarios fit well in to a taxonomy designed and used by clinicians and researchers (26, 29-30) implying that patients and clinicians agreed on the determinants of a potentially-harmful preventable-problem. Furthermore the clinicians and members of the public were consistent in which scenarios they ranked more highly, it is simply that patients have a lower threshold for concern than clinicians.

Our finding that around 30% of patient-perceived problems occurred outside general practice emphasizes the need for research in other areas of primary care, for example, 9% of the patient-perceived potentially-harmful preventable problems in the last 12 month occurred in dentistry in primary care (corresponding GB estimate 0.34 million; 0.21 million to 0.47 million) yet safety in this area remains largely unexplored.(32, 33)

For peer review only - http://bmjopen.bmj.com/site/about/guidelines.xhtml

Other studies have found differences between patients in perceiving mistakes or evaluating primary care services according to age, ethnicity, physical health and educational level (34) but we did not find this to be the case. We did find, however, that women, respondents with children, rural dwellers, and self-employed people or those not working due to disability were more likely to report a problem; although after ranking by clinicians this only remained significant for women and rural dwellers (Table 1). Some of these groups might be more frequent users of primary care; in the pilot study we observed that more frequent users of primary care were more likely to report experiencing a problem.(24) We also observed that respondents identifying with an ethnic minority group were less likely to discuss their problem with a member of primary care staff. Previous work in secondary care suggested that gender, educational level and employment status were associated with a patient's willingness to question healthcare staff.(35) Generally there were only small differences in demographics between patients in terms of being more or less likely to perceive, or discuss, a problem and it is important not to stereotype patients but to consider each person's problem equally.

Our study goes further than describing and counting the frequency of occurrence of potentially-harmful preventable-problems by providing information about how patients dealt with the problem and how it might have been prevented. Besides quicker access to primary care mentioned above, the second most frequently suggested strategy for prevention was about involving patients more in their care and keeping them informed. Other work suggested that patients are likely to blame individual clinicians for their perceived problem (7) but we did not particularly find this.

Strengths and weaknesses of the study

This large population level survey allowed for generalizable estimates of the frequency of patient-perceived potentially-harmful preventable-problems in GB for the first time and highlights that clinicians tend to judge that the patient-perceived problems are unlikely to be potentially harmful. We have verified that our survey population is similar to the English population in terms of their confidence and trust in their GP as reported in the English GP Patient survey. Previous UK studies (26) have recruited through GP practices whereby patients may be reluctant to disclose problems or answer honestly in case of compromising the patient-clinician relationship; indeed we report here that some patients did not wish to discuss their concern with primary care staff for this, and similar, reasons. Furthermore we believe that we have comprehensively captured the patient perspective through involving members of the public as research partners from study design through data acquisition to analysis and reporting. (24) We collected data related to problems occurring over the last 3 years and our denominator is patients not consultations. Time is an important tool for a primary care clinician but also problems arise over time, and the time of occurrence cannot always be assigned to a single consultation, especially with errors of omission that are associated with greater harm in primary care.(36). Reporting adverse events at a rate per consultation does not reflect the reality of the patient journey in primary care where the concept of patient safety as the management of risk over time fits well with the longer time scales.(2) The use of time in this way needs to be communicated to patients given that the most frequently suggested strategy for preventing the problem was quicker access to primary care including investigations (26%, Table 3).

For peer review only - http://bmjopen.bmj.com/site/about/guidelines.xhtml

The main weakness of the study is the self-reported nature of the problems and consequent relatively high proportion of scenarios that did not provide adequate information for ranking by clinicians. Arguably this would be improved by using a clinically trained interviewer but this could have biased the scenarios towards the clinician perspective and problems occurring outside of general practice might have gone unnoticed. Furthermore the cost of employing clinician interviewers would have been prohibitive for such a large scale survey. Ipsos MORI interviewers are accustomed to asking questions about healthcare; indeed they administer the annual GP patient survey.(28) A further weakness is that the patient suggestions regarding prevention tended to be non-specific. Collecting patients' suggestions about preventing harm was a secondary aim of this survey but patients did engage with the question and further work in partnership with clinicians is needed to develop this aspect of the survey further.

Strengths and weaknesses in relation to other studies

There are few studies undertaken from the patient perspective at the population level but the annual rates are similar to a Spanish study (7.6% vs 7%, 17). A Health Foundation research scan estimated a 1 to 2% adverse event rate per consultation (37) similar to our finding following clinician review (although we do not use consultations as the denominator). A face to face interview in family practice waiting rooms in the USA reported that 16% of respondents believed a physician had made a mistake in their care.(38) The types of problem and patient responses to the problem are similar to those that have been described qualitatively (1, 21, 39-40) but we have taken this further by using a well-defined denominator to quantify the frequency of occurrence and other descriptors of the problem from the patient's perspective.

Meaning of the study: possible explanations and implications for clinicians and policymakers

Patient trust and confidence in primary care could be improved by addressing all patient-perceived potentially harmful problems, not only those that clinicians believe to be potentially-harmful. Greater insight into the patient perspective does not mean that clinicians should intervene at the individual patient level no matter how trivial the problem may appear, with all the resource implications that would entail. Rather, our results suggest that it may be beneficial to educate patients about their responsibilities as a patient and encourage them to have more realistic expectations of primary care. To achieve this requires a step change in culture towards more patient centred care where healthcare is in partnership and patients are included in decisions.(41) Including patients more actively in healthcare may also help diminish the patient's expectations of certainty that seem to be common despite primary care being inherently uncertain.(42) If these differences in opinion between patients and clinicians are to be reconciled further work is needed to better understand why clinicians require a higher burden of proof than the public. Are they considering the problem from a medico-legal perspective or as a matter of allocation of limited resources *e.g.* disagreement about whether emotional discomfort or wasted time constitutes patient harm? (43) Conversely have the members of the public prioritised sensitivity over specificity, taken a more precautionary approach or do they have unrealistic expectations of primary care? Further work is needed to understand more about the factors underlying these different perspectives before we can develop strategies to reconcile these views. This could be done through face to face discussion between members of the public and healthcare professionals based on patient-described scenarios

For peer review only - http://bmjopen.bmj.com/site/about/guidelines.xhtml

of potential harm. In the short term patients need to be encouraged to discuss their concerns with a member of the primary care team; for around two thirds of the scenarios the clinician remained unaware of the patient's concerns given that around two thirds of patients had not discussed their problem. Patients need an accessible, informal route to raise their concerns – the most common reasons for not raising the concerns was being unable to find the appropriate person or feeling uncomfortable about raising their concern, and some were worried about the implications of doing so for their future care. Furthermore given that clinicians were significantly more likely to rank scenarios described by healthcare professionals as potentially harmful, even though they were blind to this information, patients may need support in communicating their concerns to clinicians.

In conclusion we have set out the set out the dilemma we face in reconciling clinician and patient views in relation to preventable harm in primary care. Future work should focus on strategies to encourage patients and clinicians to work together to ensure that primary care not only is safe but is also perceived to be safe by patients.

Acknowledgements: The authors would like to express their thanks and appreciation for the work done by the Mary Aldred, Gitanjali Holt, Manoj Mistry, Carole Bennett and Lindsey Brown in coding the patient-described scenarios.

Contributors: SJS, AD, JB and CG conceived and designed the study. SJS, AD, JB, CG, AE, PB, JA, DT, SL, AD, RD and NM analysed the data. SJS wrote the manuscript, and is guarantor. AD, JB, CG, AE, PB, JA, DT, SL, AD, RD, NM and SC edited the manuscript.

Funding: This study was funded by the National Institute for Health Research (http://www.nihr.ac.uk) through the Greater Manchester Primary Care Patient Safety Translational Research Centre (NIHR GM PSTRC), grant number gmpstrc-2012-1. The views expressed are those of the author(s) and not necessarily those of the NHS, the NIHR or the Department of Health. The funders had no role in study design, data collection and analysis, decision to publish, or preparation of the manuscript.

Competing interests: All authors have completed the ICMJE uniform disclosure form at www.icmje.org/coi_disclosure.pdf (available on request from the corresponding author) and declare no support from any organisation for the submitted work; no financial relationships with any organisations that might have an interest in the submitted work in the previous three years, no other relationships or activities that could appear to have influenced the submitted work.

Ethical approval: University of Manchester Ethics Committee 2 Approval 15372. Respondents to the Ipsos MORI face to face omnibus are not asked to sign a consent document, the invitation into the house after agreement to take part in the survey is considered to be consent. All respondents were provided with the participant information sheet before completing the survey questions specific to this study which explains that participation is entirely voluntary and the participant may choose to stop answering the questions at any time.

Copyright statement: the Corresponding Author has the right to grant on behalf of all authors and does grant on behalf of all authors, an exclusive licence on a worldwide basis to the BMJ Publishing

For peer review only - http://bmjopen.bmj.com/site/about/guidelines.xhtml

Group Ltd to permit this article (if accepted) to be published in BMJ editions and any other BMJPGL products and sublicences such use and exploit all subsidiary rights, as set out in our licence.

Transparency declaration: SJS affirms that this manuscript is an honest, accurate, and transparent account of the study being reported; that no important aspects of the study have been omitted; and that any discrepancies from the study as planned have been explained.

Data sharing: Raw data (coded only) is available from jill.stocks@manchester.ac.uk

Figure legends

Footnote to figure 1: See online Appendix 2 for details of coding; A coded to 2 levels, B coded to 1 level, C medication problems coded to 3 levels

Fig 1. Numbers of patient-perceived problems occurring in the last 12 months categorised according to the patient's description with clinician ranking as to the likelihood it is a potentially-harmful preventable problem (Table B, online Appendix 3).

Figure 2. Median clinician and members of the public estimates of the likelihood that the patient describes a potentially-harmful preventable-problem occurring in the last 12 months

For peer review only - http://bmjopen.bmj.com/site/about/guidelines.xhtml

For peer review only

Box 1. Brief summary of questionnaire – see online Appendix 1 for full version

Q1. Did you have confidence and trust in the GP you saw or spoke to at your last appointment? (benchmarking question)

Q2a. Have you experienced a situation with a primary care service where your health has ACTUALLY been made worse by a problem or error that could have been prevented?

Q2b. And have you experienced a situation with a primary care service where you SUSPECTED your health has been made worse by a problem or error that could have been prevented?

Q2c. And have you experienced a situation with a primary care service where your health could have been made worse had someone not NOTICED a problem or error?

Q2d. And have you experienced a situation with a primary care service where there was a problem or error that could have been prevented but it did not make your health worse?

If "yes" to more than one of Q2a-d ask Q2e to identify which happened most recently

If "no" to Q2a-d go to Q11

Q3. Thinking about the most recent occasion where you experienced a preventable problem or error caused by the primary care service, when did this occur?

Q4. Thinking about the most recent occasion, which primary care service were you using when the problem or error occurred?

Q5. Thinking about the most recent problem or error you experienced, can you briefly describe what it was and how it happened?

Q6. In your opinion, how, if at all, could the problem or error have been avoided?

Q7. Were you able to talk about the problem or error with anybody WORKING IN THE PRIMARY CARE SERVICE?

Q8. You said you were able to discuss the problem or error with somebody working in primary care. Please describe their job or role and their response.

Q9. Which of the following reasons, if any, best describes why you were unable to talk about the problem or error with somebody working in the primary care service?

Q10. In the last 12 months, have any of the following happened to you **while** using primary care, or not? *If yes go to Q4* (See online Appendix 1 for list of preventable problems)

Q11. Do you, personally, work as a Healthcare Professional in any capacity? For example, a doctor/nurse/therapist/pharmacist/other NHS staff, etc.

For peer review only

For peer review only - http://bmjopen.bmj.com/site/about/guidelines.xhtml

Table 1. Prevalence of respondents reporting a potentially-harmful preventable problem within the last 12 months and unadjusted and adjusted odds ratios estimated by logistic regression

Respondent characteristics (total) N=3984	Reported problem in last 12 months (%) n=300	Unadjusted OR–all reports	Adjusted[1] OR-all reports	Adjusted[1] OR after GP review (lower threshold[2]) n=97
**Gender (1 missing)**				
Male (1950)	111 (6%)	1 (ref)	1 (ref)	1 (ref)
Female (2033)	189 (9%)	1.7 (1.3 to 2.2)	1.7 (1.2 to 2.2)	2.3 (1.3 to 3.8)
**Age (years)**				
15 to 24 (533)	38 (7%)	1 (ref)	1 (ref)	1 (ref)
25 to 34 (573)	54 (9%)	1.4 (0.9 to 2.1)	0.7 (0.4 to 1.3)	0.4 (0.2 to 1.2)
35 to 44 (528)	30 (6%)	0.8 (0.5 to 1.3)	0.4 (0.2 to 0.8)	0.1 (0.0 to 0.6)
45 to 54 (629)	54 (9%)	1.2 (0.8 to 1.9)	0.7 (0.4 to 1.4)	0.5 (0.2 to 1.5)
55 to 64 (654)	60 (9%)	1.3 (0.9 to 2.0)	0.8 (0.4 to 1.6)	0.7 (0.2 to 2.0)
65 to 74 (609)	41 (7%)	0.9 (0.6 to 1.5)	0.5 (0.2 to 1.3)	0.7 (0.2 to 3.0)
75 or older (458)	23 (5%)	0.7 (0.4 to 1.2)	0.3 (0.1 to 0.9)	0.3 (0.1 to 1.9)
**Employment status (3 missing)**				
Paid job - full or part time (1719)	119 (7%)	1 (ref)	1 (ref)	1 (ref)
Full time student (283)	14 (5%)	0.7 (0.4 to 1.2)	0.4 (0.1 to 1.1)	0.4 (0.1 to 1.8)
Not working - long term illness/disability (133)	22 (17%)	2.7 (1.6 to 4.4)	2.3 (1.2 to 4.6)	0.9 (0.3 to 3.1)
Not working - other reason (267, includes unemployed)	24 (9%)	1.3 (0.8 to 2.1)	1.3 (0.7 to 2.4)	0.4 (0.1 to 1.4)
Not working - Housewife/husband (201)	19 (9%)	1.4 (0.8 to 2.3)	1.0 (0.5 to 2.0)	0.3 (0.1 to 1.2)
Retired (1198)	80 (7%)	1.0 (0.7 to 1.3)	1.4 (0.8 to 2.6)	0.5 (0.2 to 1.3)
Self-employed (180)	20 (11%)	1.7 (1.0 to 2.8)	2.0 (1.1 to 3.5)	0.5 (0.1 to 2.3)
**Region of domicile (23 missing)**				
Greater London (565)	38 (7%)	1 (ref)	1 (ref)	1 (ref)
East Midlands (262)	9 (3%)	0.5 (0.2 to 1.0)	0.6 (0.2 to 1.4)	0.4 (0.0 to 3.6)
East of England (425)	27 (6%)	0.9 (0.6 to 1.6)	0.6 (0.3 to 1.1)	1.8 (0.5 to 5.8)
North (176)	15 (9%)	1.3 (0.7 to 2.5)	0.8 (0.3 to 1.7)	0.7 (0.1 to 4.3)
North-West (490)	46 (9%)	1.4 (0.9 to 2.2)	1.0 (0.6 to 1.9)	1.4 (0.4 to 4.5)
Scotland (372)	27 (8%)	1.1 (0.7 to 1.8)	0.8 (0.4 to 1.6)	1.8 (0.5 to 6.1)
South East (444)	32 (7%)	1.1 (0.6 to 1.6)	1.1 (0.6 to 2.0)	2.2 (0.7 to 7.0)
South West (281)	33 (12%)	1.8 (1.1 to 3.0)	1.0 (0.5 to 2.0)	1.9 (0.5 to 6.6)
Wales (196)	15 (8%)	1.1 (0.6 to 2.1)	0.6 (0.3 to 1.4)	2.2 (0.5 to 8.5)
West Midlands (377)	19 (5%)	0.7 (0.4 to 1.3)	0.6 (0.3 to 1.3)	1.1 (0.3 to 4.4)
Yorks & Humberside (373)	39 (10%)	1.6 (1.0 to 2.6)	1.2 (0.7 to 2.3)	2.7 (0.8 to 8.4)
**Ethnicity (18 missing)**				
White (3591)	271 (8%)	1 (ref)	1 (ref)	1 (ref)
Other ethnicity (475)	26 (5%)	0.7 (0.5 to 1.0)	1.2 (0.7 to 2.2)	1.1 (0.4 to 3.0)
**Type of community**				
Urban, suburban (3051)	203 (7%)	1 (ref)	1 (ref)	1 (ref)
Rural (933)	97 (10%)	1.6 (1.3 to 2.1)	1.9 (1.3 to 2.7)	2.0 (1.1 to 3.5)
**Parental responsibility**				
Zero children under 19 (2839)	192 (7%)	1 (ref)	1 (ref)	1 (ref)
Child(ren) aged up to 19 (1145)	108 (9%)	1.4 (1.1 to 1.8)	1.2 (0.8 to 1.7)	1.5 (0.8 to 2.8)

For peer review only - http://bmjopen.bmj.com/site/about/guidelines.xhtml

**Tenure (31 missing)**				
Mortgaged (1042)	84 (8%)	1 (ref)	1 (ref)	1 (ref)
Owned outright (1441)	87 (6%)	0.7 (0.5 to 1.0)	0.8 (0.5 to 1.2)	0.9 (0.4 to 1.8)
Rented-housing association (301)	42 (14%)	1.8 (1.2 to 2.7)	1.3 (0.7 to 2.2)	1.1 (0.4 to 2.9)
Rented-private landlord (719)	49 (7%)	0.8 (0.6 to 1.2)	0.9 (0.6 to 1.5)	0.9 (0.4 to 2.1)
Rented-local authority (422)	31 (7%)	0.9 (0.6 to 1.4)	0.6 (0.3 to 1.2)	1.0 (0.4 to 2.8)
Other (28)	4 (14%)	1.9 (0.6 to 5.6)	2.2 (0.6 to 8.2)	-[3]
**Confidence and trust in GP at last appointment?**				
Yes definitely (3031)	144 (5%)	1 (ref)	-	-
Yes, to some extent (611)	68 (11%)	2.5 (1.9 to 3.4)	-	-
No, not at all (311)	88 (28%)	7.9 (5.9 to 10.7)	-	-
Don't know /can't say (31)	0 (0%)	-	-	-

[1]adjusted for gender, age, employment status, ethnicity, tenure, region of domicile, type of community, parental responsibility, highest level of education achieved, marital status, social grade, household income

[2]see Table B online Appendix 3

[3]zero problems in this category

For peer review only

For peer review only - http://bmjopen.bmj.com/site/about/guidelines.xhtml

Table 2. Details of the patient's response to the potentially-harmful preventable-problem and the primary care service involved

**Primary care service involved**	**Problems in last 12 months n=300**	**All problems analysed[1] n=564**
GP surgery	211 (70%)	395 (70%)
Dental surgery	27 (9%)	50 (9%)
Walk in clinic	16 (5%)	22 (4%)
Ambulance/A&E/ Out of hours care	16 (5%)	28 (5%)
Pharmacy	10 (3%)	19 (3%)
Community or district nursing	8 (3%)	21 (4%)
Mental health services	6 (1%)	8 (1%)
Opticians	4 (1%)	5 (1%)
Physiotherapy (in primary care)	2 (1%)	5 (1%)
missing /nk	0 (<1%)	11 (2%)
**Did you discuss the problem with primary care staff?**	**Problems in last 12 months n=300**	**All problems analysed[1] n=564**
Yes	145 (48%)	273 (48%)
No	153 (51%)	273 (48%)
missing /nk	2 (1%)	18 (3%)
**Reasons why patients did not discuss the problem with primary care staff**	**Problems in last 12 months n=153**	**All problems analysed[1] n=273**
Patient had the opportunity but did not feel comfortable discussing the problem or error	16 (10%)	43 (16%)
Patient could not find anybody with whom to discuss the problem or error	37 (24%)	75 (27%)
Patient was not concerned about the problem or error	25 (16%)	37 (14%)
Patient did not notice the problem or error or trusted the clinician's judgement at the time	11 (7%)	25 (9%)
Patient was too distressed or ill to discuss the problem or error	18 (12%)	30 (11%)
Other - problem was resolved in another way by the patient without involving primary care	10 (7%)	13 (5%)
Other - patient believed primary care staff would not be interested in the problem or would not take it seriously or it would not improve primary care	7 (5%)	14 (5%)
Other – patient believed that discussing the problem with a primary care staff might have negative implications for their future care	6 (4%)	6 (2%)
Other - patient did know that they were allowed to express an opinion or how to raise the problem	5 (3%)	5 (2%)
Other - patient accepts that such problems will arise in primary care or didn't want to use primary care resources when primary care staff are very busy	5 (3%)	6 (2%)
Other - patient intends to discuss with primary care	4 (3%)	6 (2%)

For peer review only - http://bmjopen.bmj.com/site/about/guidelines.xhtml

professional at the next opportunity		
Don't Know/missing	9 (6%)	13 (5%)
**Profession of discussant**	**Problems in last 12 months n=145**	**All problems analysed[1] n=273**
GP/practice nurse	66 (46%)	144 (53%)
Practice manager/receptionist/administrator	25 (17%)	39 (14%)
Pharmacist/dispenser	7 (5%)	14 (5%)
General Dental Practitioner	8 (6%)	18 (7%)
Hospital doctor or nurse/A&E or OOH staff/paramedic	15 (10%)	18 (7%)
Other primary care staff	14 (10%)	17 (6%)
PALS or NHS direct staff	1 (1%)	2 (1%)
Unclear, don't know or missing	9 (6%)	21 (8%)
**Role of discussant in patient's care**	**Problems in last 12 months n=145**	**All problems analysed[1] n=273**
Member of staff central to respondent's care	60 (41%)	112 (41%)
Member of staff in the same team or organisation	35 (24%)	84 (31%)
Member of staff in a different team or organisation	31 (21%)	40 (15%)
Role of member of staff is unclear	8 (6%)	20 (7%)
missing	11 (8%)	17 (%)

[1]All problems analysed includes scenarios arising from Ipsos MORI survey in the last 3 years and the pilot survey (24) within the last 12 months

For peer review only

Table 3. Patient suggestions as to how the potentially-harmful preventable problem might have been prevented

How could it be prevented?	Problems in last 12 months n=300	All problems analysed[1] n=564
**1. More resources - total**	**100 (33%)**	**157 (28%)**
1.1 Quicker access to primary care	43 (14%)	62 (11%)
1.2 More thorough and quicker investigations	35 (12%)	59 (10%)
1.3 Fewer demands on primary care – more staff or fewer patients	7 (2%)	12 (2%)
1.4 More time with clinicians for treatment and diagnosis	8 (3%)	12 (2%)
1.5 Improved access to social care	3 (1%)	3 (1%)
1.6 More follow-up by primary care	2 (1%)	3 (1%)
1.7 Improved continuity of care	1 (<1%)	2 (<1%)
1.8 Access to a second opinion	1 (<1%)	2 (<1%)
1.9 Provision of resources to manage long term conditions	0	2 (<1%)
**2. Improved communication and involvement of patients - total**	**53 (18%)**	**92 (16%)**
1.1 Listen to the patient and trust their judgement more	36 (12%)	68 (12%)
1.2 Tell patients about their diagnosis, test results, changes in medication or loss of results	10 (3%)	15 (3%)
1.3 Improve communication between staff (within or outside primary care)	7 (2%)	9 (2%)
**3. Better organisation and administration - total**	**27 (9%)**	**48 (9%)**
3.1 Follow up referrals and appointments to ensure they happen, be consistent in sending routine reminders	12 (4%)	23 (4%)
3.2 Log in or process results as soon as received to avoid loss	5 (2%)	7 (1%)
3.3 Keep the notes up to date, well-organised, safe and ensure information is transcribed accurately	9 (3%)	15 (3%)
3.4 Keep a record of the location of equipment	0	1 (<1%)
3.5 Improve the method of appointment allocation	0	1 (<1%)
3.6 Fine patients for not attending appointments	1 (<1%)	1 (<1%)
**4. Improved prescribing systems - total**	**21 (7%)**	**45 (8%)**
4.1 More when checks on prescribing and dispensing	19 (6%)	32 (6%)
4.2 Check repeat prescriptions carefully, especially for transcribing errors	2 (1%)	10 (2%)
4.3 Use medication reviews and IT clinical decision support systems	0	3 (1%)
**5. Better clinical practice - total**	**17 (6%)**	**47 (8%)**
5.1 Take in to account all the patient's information - their medical history and results and letters	7 (2%)	27 (5%)
5.2 Address the patient's problem in some way – patients can feel their problem is being ignored	9 (3%)	18 (3%)
5.3 Act on advice from other clinicians and test results	1 (<1%)	2 (<1%)
**6. Staff training - total**	**22 (7%)**	**53 (9%)**
6.1 More informed and better trained staff	22 (7%)	53 (9%)

For peer review only - http://bmjopen.bmj.com/site/about/guidelines.xhtml

**Other responses - total**	**60 (20%)**	**122 (22%)**
• Don’t know/missing	28 (9%)	64 (11%)
• Problem was due to an individual member of staff	6 (2%)	11 (2%)
• Do not make wrong, late, delayed diagnosis	7 (2%)	15 (3%)
• Prescribe right, better, different, more, less medicine	8 (3%)	15 (3%)
• Should have been referred	6 (2%)	9 (2%)
• Better organisation	3 (1%)	4 (1%)
• Patient recognised their own responsibility	2 (1%)	2 (<1%)
• Laboratory procedures were the problem	0	2 (<1%)

[1]All problems analysed includes scenarios arising from Ipsos MORI survey in the last 3 years and the pilot survey (24) within the last 12 months

For peer review only - http://bmjopen.bmj.com/site/about/guidelines.xhtml

References

1. Rhodes P, Campbell S, Sanders C. Trust, temporality and systems: how do patients understand patient safety in primary care? A qualitative study. Health Expectations. 2016;19(2):253-63.

2. Vincent C, Amalberti R. Safer Healthcare: Strategies for the Real World. Springer, 2016. http://www.springer.com/gb/book/9783319255576 Accessed 04/04/17

3. The Health Foundation. Evidence scan: Involving patients in improving safety. January 2013. http://www.health.org.uk/sites/health/files/InvolvingPatientsInImprovingSafety.pdf Accessed 04/04/17

4. King A, Daniels J, Lim J, Cochrane DD, Taylor A, Ansermino JM. Time to listen: a review of methods to solicit patient reports of adverse events. Qual Saf Health Care. 2010;19(2):148-57.

5. Kuzel AJ, Woolf SH, Gilchrist VJ, Engel JD, LaVeist TA, Vincent C, et al. Patient reports of preventable problems and harms in primary health care. Ann Fam Med. 2004;2(4):333-40.

6. Blendon RJ, DesRoches CM, Brodie M, Benson JM, Rosen AB, Schneider E, et al. Views of practicing physicians and the public on medical errors. N Engl J Med. 2002;347(24):1933-40.

7. Hotvedt R, Forde OH. Doctors are to blame for perceived medical adverse events. A cross sectional population study. The Tromso Study. BMC health services research. 2013;13:46.

8. The Health Foundation. Evidence Scan: Improving safety in primary care. November 2011. http://www.health.org.uk/sites/health/files/ImprovingSafetyInPrimaryCare.pdf Accessed 04/04/17

9. Weingart SN et al. RS What can hospitalized patients tell us about adverse events? Learning from patient-reported incidents. J Gen Intern Med. 2005 Sep;20(9):830-6.

10. Weissman JS, Schneider EC, Weingart SN, et al. Comparing patient-reported hospital adverse events with medical record review: do patients know something that hospitals do not? Ann Intern Med. 2008 Jul 15;149(2):100-8

11. Southwick FS, Cranley NM, Hallisy JA. A patient-initiated voluntary online survey of adverse medical events: the perspective of 696 injured patients and families. BMJ Qual Saf. 2015;24(10):620-9.

12. Longtin Y, Sax H, Leape LL, Sheridan SE, Donaldson L, Pittet D. Patient participation: current knowledge and applicability to patient safety. Mayo Clinic proceedings. 2010;85(1):53-62.

13. Sandars J, Esmail A. The frequency and nature of medical error in primary care: understanding the diversity across studies. Family practice. 2003;20(3):231-6.

14. Panesar SS, deSilva D, Carson-Stevens A, Cresswell KM, Salvilla SA, Slight SP, et al. How safe is primary care? A systematic review. BMJ Quality & Safety. 2016;25(7):544-53.

15. Michel P, Brami J, Chaneliere M, Kret M, Mosnier A, Dupie I, et al. Patient safety incidents are common in primary care: A national prospective active incident reporting survey. PLoS One. 2017;12(2):e0165455.

For peer review only

For peer review only - http://bmjopen.bmj.com/site/about/guidelines.xhtml

16. Northcott H, Vanderheyden L, Northcott J, Adair C, McBrien-Morrison C, Norton P, et al. Perceptions of preventable medical errors in Alberta, Canada. International journal for quality in health care : journal of the International Society for Quality in Health Care / ISQua. 2008;20(2):115-22.

17. Mira JJ, Nebot C, Lorenzo S, Perez-Jover V. Patient report on information given, consultation time and safety in primary care. Qual Saf Health Care. 2010;19(5):e33.

18. Wasson JH, MacKenzie TA, Hall M. Patients use an internet technology to report when things go wrong. Qual Saf Health Care. 2007;16(3):213-5.

19. Ricci-Cabello I, Goncalves DC, Rojas-Garcia A, Valderas JM. Measuring experiences and outcomes of patient safety in primary care: a systematic review of available instruments. Family practice. 2015;32(1):106-19.

20. Ricci-Cabello I, Avery AJ, Reeves D, Kadam UT, Valderas JM. Measuring Patient Safety in Primary Care: The Development and Validation of the "Patient Reported Experiences and Outcomes of Safety in Primary Care" (PREOS-PC). Ann Fam Med. 2016;14(3):253-61.

21. Ricci-Cabello I, Saletti-Cuesta L, Slight SP, Valderas JM. Identifying patient-centred recommendations for improving patient safety in General Practices in England: a qualitative content analysis of free-text responses using the Patient Reported Experiences and Outcomes of Safety in Primary Care (PREOS-PC) questionnaire. Health Expectations. 2017.

22. NHS England. General practice patient safety reporting form launched. 26th February 2015. https://www.england.nhs.uk/2015/02/gp-patient-safety-reporting/ Accessed 04/04/17

23. European Commission. Special Eurobarometer 411 Patient Safety and Quality of Care 2014. DOI:10.2772/33467 https://ec.europa.eu/health/sites/health/files/patient_safety/docs/ebs_411_sum_en.pdf Accessed 04/04/17

24. Stocks SJ, Donnelly A, Esmail A, Beresford J, Luty S, Deacon R, et al. Development and piloting of a survey to estimate the frequency and nature of potentially-harmful preventable-problems in primary care from a UK patient's perspective. BMJ Open 2017; In press. (bmjopen-2017-017786.R2)

25. NHS Improvement. National quarterly data on patient safety incident reports. September 2016. https://improvement.nhs.uk/resources/national-quarterly-data-patient-safety-incident-reports-september-2016/ Accessed 04/04/17

26. Hutchinson A, Young TA, Cooper KL, McIntosh A, Karnon JD, Scobie S, et al. Trends in healthcare incident reporting and relationship to safety and quality data in acute hospitals: results from the National Reporting and Learning System. Qual Saf Health Care. 2009;18(1):5-10.

27. Ipsos MORI Face-to-Face Omnibus (Capibus) https://www.ipsos-mori.com/ourexpertise/omnibusservices/capibus.aspx Accessed 04/04/17

28. NHS England. GP Patient Survey – National summary report. January 2016. http://gp-survey-production.s3.amazonaws.com/archive/2016/January/January+2016+National+Summary+Report.pdf Accessed 04/04/17

For peer review only - http://bmjopen.bmj.com/site/about/guidelines.xhtml

29. Dovey SM, Meyers DS, Phillips RL, Jr., Green LA, Fryer GE, Galliher JM, et al. A preliminary taxonomy of medical errors in family practice. Qual Saf Health Care. 2002;11(3):233-8.

30. Makeham MA, Dovey SM, County M, Kidd MR. An international taxonomy for errors in general practice: a pilot study. Med J Aust. 2002;177(2):68-72.

31. McGraw K WS. Forming inferences about some intraclass correlation coefficients. Psychological Methods. 1994;1(1):30-46.

32. Bailey E, Tickle M, Campbell S. Patient safety in primary care dentistry: where are we now? Br Dent J. 2014;217(7):339-44.

33. Ensaldo-Carrasco E, Suarez-Ortegon MF, Carson-Stevens A, Cresswell K, Bedi R, Sheikh A. Patient Safety Incidents and Adverse Events in Ambulatory Dental Care: A Systematic Scoping Review. Journal of patient safety. 2016;08:08.

34. Campbell JL, Ramsay J, Green J. Age, gender, socioeconomic, and ethnic differences in patients' assessments of primary health care. Quality in health care : QHC. 2001;10(2):90-5.

35. Davis RE, Koutantji M, Vincent CA. How willing are patients to question healthcare staff on issues related to the quality and safety of their healthcare? An exploratory study. Qual Saf Health Care. 2008;17(2):90-6.

36. The WHO Safer Primary Care Expert Working Group. Safer Primary Care: A Global Challenge. Summary of Inaugral Meeting 2012. http://www.who.int/patientsafety/summary_report_of_primary_care_consultation.pdf Accessed 04/04/17

37. The Health Foundation. Evidence Scan: Levels of Harm in Primary Care. November 2011. http://www.health.org.uk/sites/health/files/LevelsOfHarmInPrimaryCare.pdf Accessed 04/04/17

38. Kistler CE, Walter LC, Mitchell C, Sloane PD. Patient perceptions of mistakes in ambulatory care. Archives of Internal Medicine. 2010;170(16):1480-7.

39. Hernan AL, Giles SJ, Fuller J, Johnson JK, Walker C, Dunbar JA. Patient and carer identified factors which contribute to safety incidents in primary care: a qualitative study. BMJ Qual Saf. 2015; 24(9):583-93.

40. Litchfield I, et al. Routine failures in the process for blood testing and the communication of results to patients in primary care in the UK: a qualitative exploration of patient and provider perspectives. BMJ Qual Saf. 2015 Nov;24(11):681-90

41. The Health Foundation. Person-centred care made simple. 2014. http://www.health.org.uk/sites/health/files/PersonCentredCareMadeSimple.pdf Accessed 04/04/17

42. Heneghan C, Glasziou P, Thompson M, Rose P, Balla J, Lasserson D, et al. Diagnostic strategies used in primary care. BMJ. 2009;338.

43. Elder NC, Meulen MV, Cassedy A. The Identification of Medical Errors by Family Physicians During Outpatient Visits. Annals of Family Medicine. 2004;2(2):125-9.

For peer review only - http://bmjopen.bmj.com/site/about/guidelines.xhtml

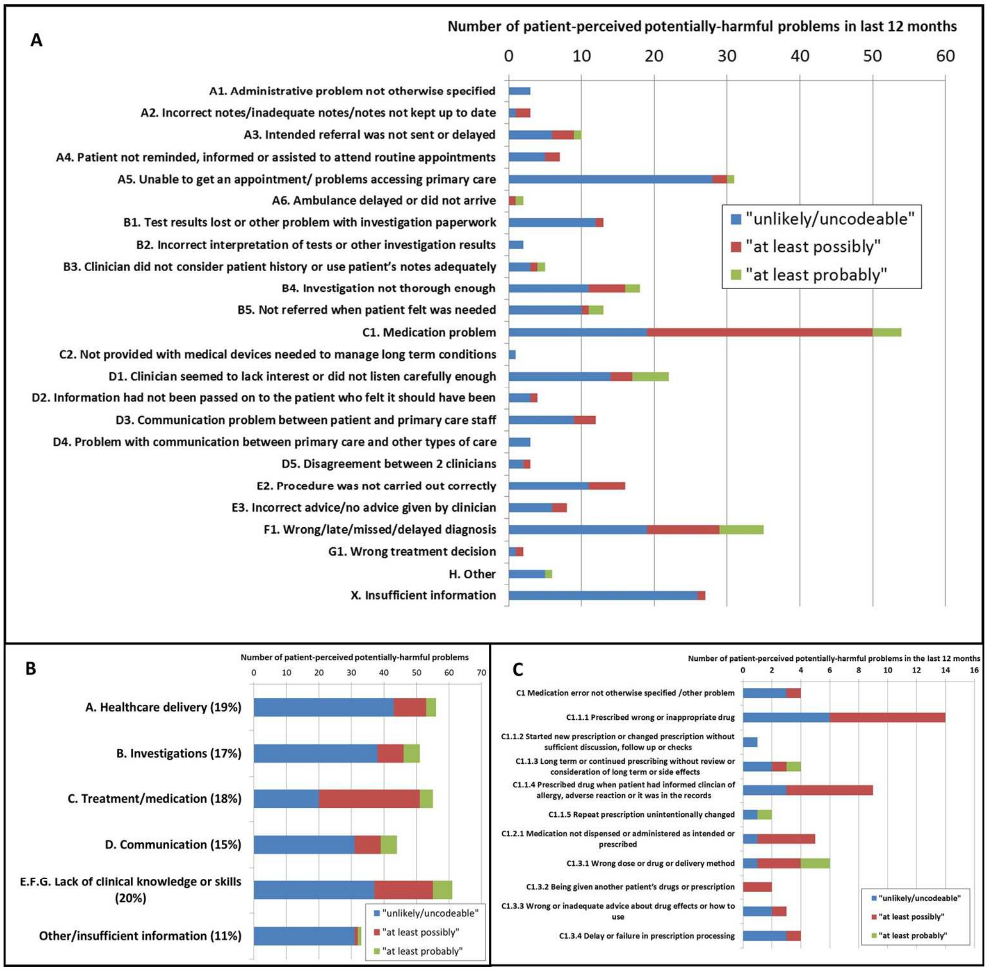

174x170mm (300 x 300 DPI)

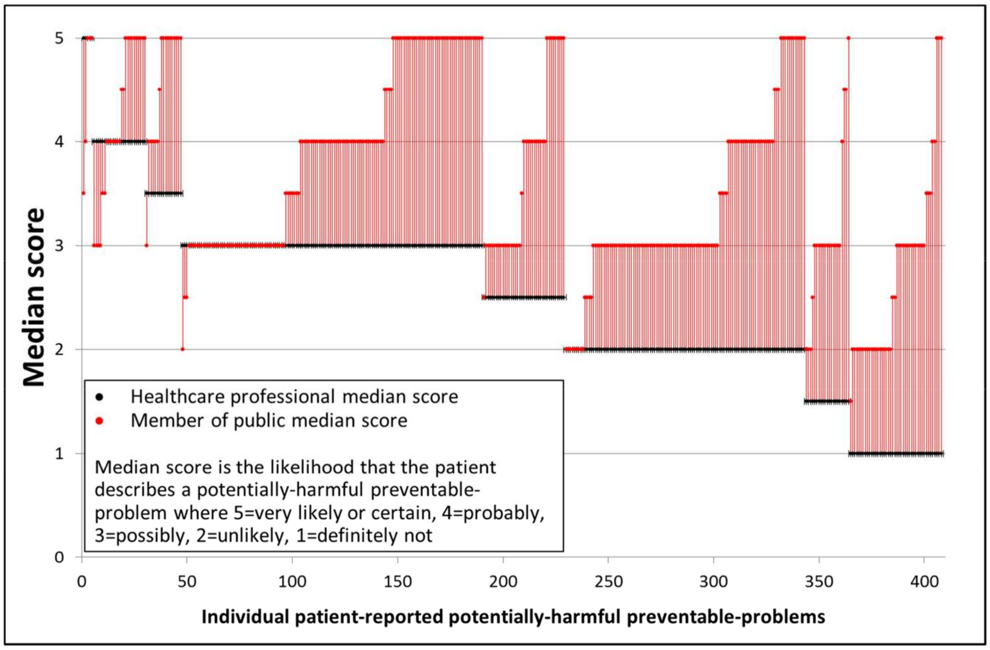

240x158mm (200 x 200 DPI)

view only

Appendix 1. Survey administered as part of the Ipsos MORI GB Face to Face Omnibus between 8th and 21st April 2016

We'd now like you to think about the last time you personally had an appointment for yourself, with a GP.

Q1. Did you have confidence and trust in the GP you saw or spoke to at your last appointment?
1. Yes, definitely 2. Yes, to some extent 3. No, not at all 4. Don't know / can't say

INTERVIEWER INSTRUCTION: READ OUT AND DISPLAY ON SCREEN.

The next few questions are about primary care.

Primary Care is the local healthcare that we receive at our GP or dental surgery, NHS walk-in centres, pharmacists (or high street chemist) and optometrists. This also could include all non-hospital care, for example, healthcare service provided by out of hours care, community (or district) nursing, ambulance, physiotherapy or other types of therapy or tests based at a GP surgery, learning disability services and any other non-hospital medical care.

We understand that this is a highly sensitive topic and would therefore like to remind you that any information you give is strictly confidential and will be used for research purposes only. You will not be identifiable as an individual from the responses you give.

At each question, if you do not wish to answer, you can refuse.

For the next question, we'd like you to think about the occasions when you have personally used primary care for yourself.

Q2a. Have you experienced a situation with a primary care service where your health has ACTUALLY been made worse by a problem or error that could have been prevented?
1. Yes 2. No 3. Don't Know

Q2b. And have you experienced a situation with a primary care service where you SUSPECTED your health has been made worse by a problem or error that could have been prevented?
1. Yes 2. No 3. Don't Know

Q2c. And have you experienced a situation with a primary care service where your health could have been made worse had someone not NOTICED a problem or error?
1. Yes 2. No 3. Don't Know

Q2d. And have you experienced a situation with a primary care service where there was a problem or error that could have been prevented but it did not make your health worse?
1. Yes 2. No 3. Don't Know

IF 2 OR MORE SCENARIOS AT Q2a to Q2e ARE CODED 1 THEN ASK Q2e

Q2e. You mentioned you have experienced the following situation(s) with a primary care service. Which of the following did you experience most recently?

1. 'My health was made worse'
2 'I suspect health was made worse'

For peer review only

3 'My health could have been made worse if the problem or error had not been noticed'
4 'There was no effect on my health'

ASK ALL WHO CODE 1 AT Q2
Q3. Thinking about the most recent occasion where you experienced a preventable problem or error caused by the primary care service, when did this occur?
1. In the last 12 months
2. 1 year up to 2 years ago
3. 2 years up to 3 years ago
4. 3 or more years ago

ASK ALL CODING 1 AT Q2 OR 1 AT Q11
Q4. Thinking about the most recent occasion, which primary care service were you using when the problem or error occurred?
1. GP surgery
2. Out of hours care
3. Walk in clinic
4. Dental surgery
5. Pharmacy
6. Community or district nursing
7. Ambulance
8. Opticians
9. Other (please specify)

INTERVIEWER INSTRUCTION: For the next five questions, please record enough information so that somebody else reading the description can understand what happened.

ASK ALL CODING 1 AT Q2 OR 1 AT Q11
Q5. Thinking about the most recent problem or error you experienced, can you briefly describe what it was and how it happened?

Q6 In your opinion, how, if at all, could the problem or error have been avoided?

Q7. Were you able to talk about the problem or error with anybody WORKING IN THE PRIMARY CARE SERVICE?
1. Yes 2. No

INTERVIEWER INSTRUCTION: if prompted, this can be anyone in the primary care service, including for example, the receptionist at a GP surgery or another nurse/doctor who wasn't working directly in their care.

ASK ALL CODING 1 AT Q7
Q8. You said you were able to discuss the problem or error with somebody working in primary care. Please describe their job or role and their response.

ASK ALL CODING 2 AT Q7
Q9. Which of the following reasons, if any, best describes why you were unable to talk about the problem or error with somebody working in the primary care service?
1. I had the opportunity but did not feel comfortable discussing the problem or error
2. I could not find anybody with whom I could discuss the problem or error

For peer review only

3. I was not concerned about the problem or error
4. I did not notice the problem or error
5. I was too distressed to discuss the problem or error
6. Other (please specify)

ASK IF (Q2 '2 OR DK OR REF')
Q10. In the last 12 months, have any of the following happened to you **while** using primary care, or not? 1. Yes 2. No

IF YES AT Q11, REDIRECT TO Q4

(RANDOMISE 1-16(KEEP 2&3 TOGETHER, KEEP 6&7 TOGETHER, KEEP 9&10 TOGETHER), ALLOW DK AND REF)

1. Received a wrong or late diagnosis
2. Was not referred for further investigation when requested by you as a patient
3. Was not referred for further investigation in error by healthcare practitioner (for example, they forgot to refer you onwards)
4. Test results lost or mixed up
5. Received the wrong medicine or wrong dose
6. Should not have been prescribed medicine because of another health problem
7. Should not have been prescribed medicine because of another medication already being taken
8. Poor communication leading to misunderstanding of diagnosis or treatment
9. Not referred to a specialist when needed when requested by you as a patient
10. Not referred to a specialist when needed in error by healthcare practitioner (for example, they forgot to refer you onwards)
11. Received unclear instructions about treatment
12. Not offered access to prevention or screening programmes e.g. CVD/stroke prevention clinics
13. A medical professional failed to recognise or act on vulnerable people's needs e.g. child abuse, suicide risk or mental health problems
14. Mistake with a procedure e.g. dental treatment, injection, ear syringing, physiotherapy
15. Not notified about recommended vaccinations e.g. flu, HPV
16. A medical professional practicing poor hygiene

ASK ALL CODING 1 AT Q2 OR 1 AT Q11
Q11. Do you, personally, work as a Healthcare Professional in any capacity? For example, a doctor/nurse/therapist/pharmacist/other NHS staff, etc.
1. Yes 2. No

For peer review only

Appendix 2.

Table A. Coding of patient-reported potentially-unsafe scenarios in primary care

**1. Errors in the process of the healthcare delivery system**	
Makeham 2002, Dovey 2002	Common threads reported in this study
1.1. Errors in the process of conducting an administrative task	A1. Administrative problem not otherwise specified
1.1.1. Information filed in wrong place or wrong time	
1.1.2. Unavailability of information that should have been in patients charts<br>1.1.2.1. Entire chart or part of chart could not be accessed when needed<br>1.1.2.2. Care provided was not documented<br>1.1.2.3. Item(s) of information missing from chart	A2. Incorrect notes/inadequate notes/notes not kept up to date
1.1.3. Errors in patient's movement through the healthcare delivery system	A3. Intended referral was not sent or delayed<br>A4. Patient not reminded, informed or assisted to attend regular check-ups or other necessary routine treatments
1.1.4. Errors in the taking and distributing of messages	
1.1.5. Errors in managing appointments for healthcare	A5. Unable to get an appointment/other problems with making appointment<br>A6. Ambulance delayed or did not arrive
**1.2. Errors in the process of investigating a patient's condition**	
1.2.1. Laboratory errors<br>1.2.1.1. Wrong test ordered or test not ordered when appropriate<br>1.2.1.2. Errors in the process of obtaining or processing a laboratory specimen<br>1.2.1.3. Error in the process of physician receiving accurate laboratory results in a timely fashion<br>1.2.1.4. Inappropriate response to an abnormal laboratory result<br>1.2.3. Errors in the processes of other investigations<br>1.2.3.1. Wrong test ordered or test not ordered when appropriate<br>1.2.3.2. Errors in the process of obtaining or processing of other diagnostic investigation<br>1.2.3.3. Error in the process of physician receiving accurate test results of other investigation in a timely fashion<br>1.2.3.4. Inappropriate response to an abnormal result of other investigation	B1. Test results lost or other problem with investigation or paperwork<br>B2. Incorrect interpretation of tests or other investigation results<br>B3. Clinician did not consider patient history sufficiently/did not use patient's notes adequately<br>B4. Investigation not thorough enough<br>B5. Not referred when patient felt was needed
**1.3. Errors in the process of treating a patient's condition**	
1.3.1. Errors in the process of treating with medications<br>1.3.1.1. Wrong medication or wrong dose of medication ordered or medication not ordered by physician when appropriate<br>1.3.1.2. Error in the process of delivering a medication order or inappropriate medication order by a provider working under physician supervision<br>1.3.1.3. Error in the process of dispensing medication as ordered	C1. Medication problem<br><br>C2. Not provided with medical devices needed to manage long term conditions
1.3.2. Errors in other treatments	C3. Problem with dental treatment or

	diagnosis
**1.4. Errors in the process of communication**	
1.4.1. Errors in communication between primary healthcare provider and patients	D1. Clinician seemed to lack interest in the patient's health problem or did not listen carefully enough<br>D2. Information about the patient's health had not been passed on to the patient who felt it should have been<br>D3. Communication problem between patient and primary care staff
1.4.2. Errors in communication between healthcare providers	D4. Problem with communication between primary care and other types of care including secondary care<br>D5. Disagreement between 2 clinicians
**2. Errors arising from lack of clinical knowledge or skills**	
2.1. Errors in the execution of a clinical task<br>2.1.1. Non-clinical staff made the wrong clinical decision<br>2.1.2. Failed to follow standard practice<br>2.1.3. Lacked needed experience or expertise in a clinical task	E1. Administrative staff seemed to make clinical decisions<br>E2. Procedure was not carried out correctly<br>E3. Incorrect advice/no advice given by clinician
**2.2. Errors in diagnosis**<br>2.2.1. Wrong or delayed diagnosis	F1. Wrong/late/missed/delayed diagnosis
**2.3. Wrong treatment decision**	G1. Wrong treatment decision
	H. Other
	X. Not a problem/ insufficient information/refused/don't know

Table B. Level 4 coding of patient-reported potentially-unsafe medication scenarios

Common threads reported in this study grouped as described by Makeham 2002, Dovey 2002
C1 Medication error not otherwise specified /other problem
• **1.3.1.1. Ordering medications (prescribing)**
C1.1.1 Prescribed wrong or inappropriate drug
C1.1.2 Started new prescription or changed prescription without sufficient discussion, follow up or checks
C1.1.3 Long term or continued prescribing without review or consideration of long term or side effects
C1.1.4 Prescribed drug when should have known contra-indicated *e.g.* patient had informed clinician of allergy, adverse reaction or it was in the records
C1.1.5 Repeat prescription unintentionally changed
C1.1.6 Out of date repeat prescription mistakenly re-issued
• **1.3.1.2./1.3.1.3. Implementing or receiving medications (dispensing or issuing)**
C1.2.1 Medication not dispensed or administered as intended or prescribed
• **1.3.1.1/1.3.1.2./1.3.1.3. Ordering, implementing or receiving medications**
C1.3.1 Wrong dose or drug or delivery method
C1.3.2 Being given another patient's drugs or prescription
C1.3.3 Wrong or inadequate advice about drug effects or how to use
C1.3.4 Delay or failure in prescription processing

For peer review only

Table C. Scoring for likelihood that the patient-reported scenario is potentially-unsafe

Score	How likely do you think it is the patient was correct in thinking that their health might be worsened, or actually was made worse, because of a mistake or a problem in primary care that could have been prevented? Choose from the options below.
5	Very likely or certain (75-100% confident is a potentially unsafe scenario)
4	Probably (50-74% confident is a potentially unsafe scenario)
3	Possibly (25-49% confident is a potentially unsafe scenario)
2	Unlikely (bottom 25% confident is a potentially unsafe scenario)
1	Definitely not a potentially unsafe event (0% chance is a potentially unsafe scenario)
-	Insufficient information
-	Don't know
-	Other - add text at end of row

For peer review only

Appendix 3. Additional results

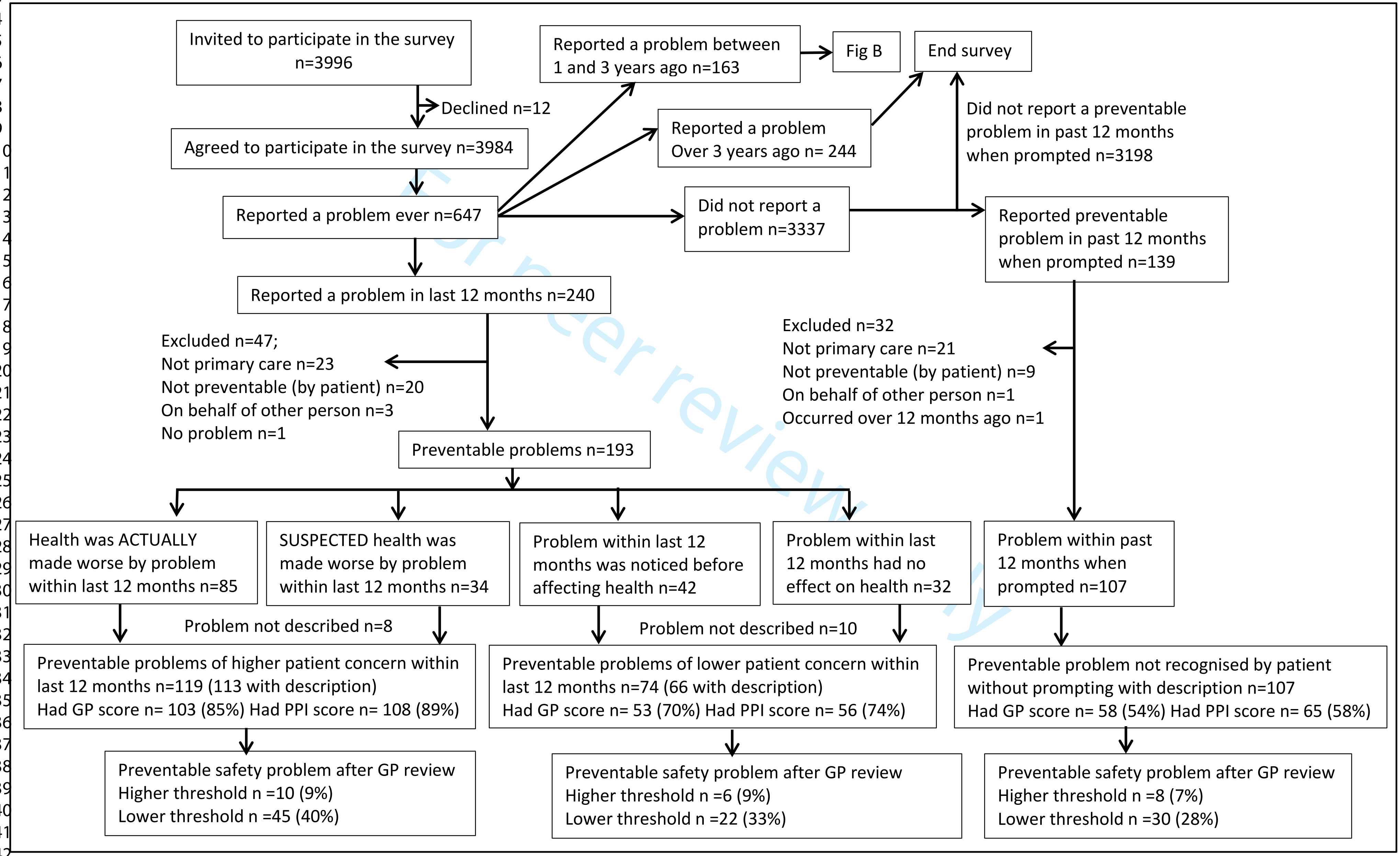

Fig A. Flow chart of participants reporting a potential-harmful preventable-problem within the last 12 months

For peer review only - http://bmjopen.bmj.com/site/about/guidelines.xhtml

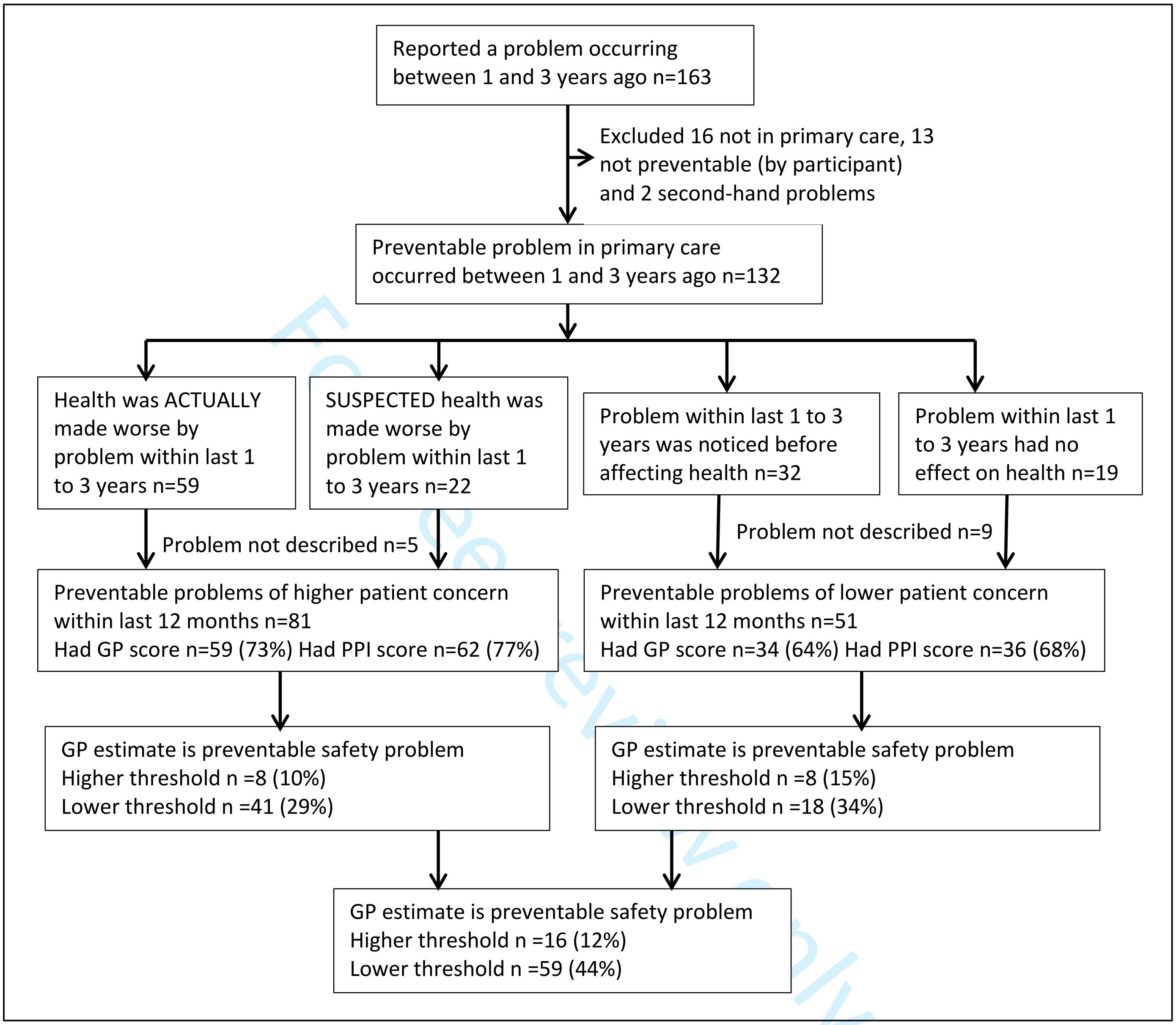

Fig B. Flow chart of participants reporting a potential-harmful preventable-problem within the last 1 to 3 years

For peer review only - http://bmjopen.bmj.com/site/about/guidelines.xhtml

Table A. Demographics of responders to Ipsos MORI GB Face to Face Omnibus April 2016

	Number of participants (%) n=3984	Population level estimates for comparison	Population comparator source; $P(\chi^2)$= probability survey population differs from population comparator
**Confidence and trust in GP at last appointment?**			
Yes definitely	3031 (76%)	523498 (63%)	GP patient survey in England mid-2015(25) $P(\chi^2)<0.0001$
Yes, to some extent	611 (15%)	235760 (29%)	
No, not at all	311 (8%)	37743 (5%)	
Don't know /can't say	31 (1%)	28866 (3%)	
**Gender (1 missing)**			
Male	1950 (49%)	32074400 (49%)	ONS mid-2015 estimates[1] $P(\chi^2)=0.7$
Female	2033 (51%)	33035600 (51%)	
**Age**			
15 to 24	533 (13%)	8118600 (15%)	ONS mid-2015 estimates[1] $P(\chi^2)<0.0001$
25 to 34	573 (14%)	8822700 (16%)	
35 to 44	528 (13%)	8378300 (16%)	
45 to 54	629 (16%)	9196000 (17%)	
55 to 64	654 (16%)	7452400 (13%)	
65 to 74	609 (15%)	6339800 (11%)	
75 or older	458 (12%)	5271400 (10%)	
**Ethnicity (18 missing)**			
White	3491 (88%)	48209395 (86%)	England & Wales census (2011)[2] $P(\chi^2)<0.0001$
Other ethnicity	475 (12%)	7866517 (14%)	
**Social Grade[3]**			
A/B	1054 (26%)	8081619 (23%)	England & Wales census (2011)[2] $P(\chi^2)<0.0001$
C1	1122 (28%)	10796044 (30%)	
C2	771 (19%)	7865976 (22%)	
D/E	1037 (26%)	8903873 (25%)	

[1]https://www.ons.gov.uk/peoplepopulationandcommunity/populationandmigration/populationestimates/bulletins/annualmidyearpopulationestimates/latest

[2]https://www.ons.gov.uk/peoplepopulationandcommunity/populationandmigration/populationestimates/bulletins/keystatisticsandquickstatisticsforlocalauthoritiesintheunitedkingdom/2013-10-11

[3]A/B High or intermediate managerial, professional or administrative, C1 Supervisory, clerical and junior managerial, professional or administrative, C2 skilled manual workers, D/E semi and unskilled manual workers, casual or lowest grade workers, state pensioners, unemployed with state benefits only

For peer review only - http://bmjopen.bmj.com/site/about/guidelines.xhtml

Table B. Categorisation of patient-described scenarios according to clinician ranking as to the likelihood they represent a potentially-harmful preventable-problem

Group	Scores on a 5 point scale of "very likely or certain", "probably", "possibly", "unlikely", "definitely not" (see table C, online Appendix 2)	Unprompted problems (answered "yes" to Q2, Box1)				All problems within past 12 months (answered "yes" to Q2or Q10, Box1) n=300	
		Within past 12 months n=193		Within past 3 years n=325			
		Clinicians	Members of the Public	Clinicians	Members of the Public	Clinicians	Members of the Public
1. Higher threshold	Median score higher than "probably" or at least one score of "very likely or certain"	16 (8%)	91 (47%)	28 (9%)	165 (51%)	24 (8%)	116 (39%)
2. Lower threshold	Median score higher than "possibly" or at least one score of "probably" or higher	67 (35%)	145 (75%)	124 (38%)	237 (73%)	97 (32%)	198 (66%)
3. Any possibility	At least one score of "unlikely" or higher	141 (73%)	157 (81%)	232 (71%)	254 (78%)	194 (65%)	221 (74%)
4. No problem	All scores "definitely not" (or not-coded)	8 (4%)	0	9 (3%)	0	13 (4%)	0
5. Not-coded	Insufficient information for coding by all raters	44 (23%)	36 (19%)	84 (26%)	71 (22%)	93 (31%)	79 (26%)

For peer review only - http://bmjopen.bmj.com/site/about/guidelines.xhtml

Table C. Survey responses and respondent characteristics as predictors of clinician and members of the public estimates of the likelihood that the scenario describes a potentially-harmful preventable problem

Respondent characteristics (total) n=406 (ranked by at least one clinician)	Clinician – lower threshold[1] (n=224, 55%)		Members of the public – higher threshold[2] (n=267, 66%)	
	Frequency (%)	Adjusted odds ratio	Frequency (%)	Adjusted odds ratio
**Source of respondent (0 missing)**				
Ipsos MORI f2f Omnibus (299)	153 (51%)	1 (ref)	182 (61%)	1 (ref)
Pilot survey (107)	71 (66%)	1.5 (0.9 to 2.7)	85 (79%)	5.2 (2.5 to 10.8)
**Gender (3 missing)**				
Male (150)	79 (53%)	1 (ref)	93 (62%)	1 (ref)
Female (253)	142 (56%)	1.2 (0.8 to 1.9)	172 (68%)	1.5 (0.9 to 2.4)
**Age (3 missing)**				
15 to 24 years (46)	21 (46%)	1 (ref)	28 (61%)	1 (ref)
25 to 34 years (60)	34 (57%)	1.5 (0.7 to 3.5)	43 (72%)	1.4 (0.6 to 3.7)
35 to 44 years (38)	24 (63%)	1.8 (0.7 to 4.5)	30 (79%)	1.9 (0.6 to 5.6)
45 to 54 years (74)	44 (59%)	1.5 (0.7 to 3.4)	50 (68%)	1.1 (0.5 to 2.7)
55 to 64 years (82)	45 (55%)	1.4 (0.6 to 3.2)	50 (61%)	1.0 (0.4 to 2.3)
65 to 74 years (75)	39 (52%)	1.2 (0.5 to 2.8)	49 (65%)	1.1 (0.4 to 2.6)
75 years or older (28)	14 (50%)	1.1 (0.4 to 3.2)	15 (54%)	0.6 (0.2 to 1.8)
**Patient estimate of impact of the problem on their health (0 missing)**				
Actually or suspected made health worse (192)	109 (57%)	1 (ref)	139 (73%)	1 (ref)
Noticed before made health worse or had no effect on health (106)	58 (55%)	0.8 (0.5 to 1.4)	69 (65%)	0.6 (0.3 to 1.1)
Prompted by Q10 (108)	57 (53%)	0.7 (0.4 to 1.2)	59 (55%)	0.3 (0.1 to 0.5)
**Patient is qualified as a healthcare professional or volunteers in healthcare research[2] (0 missing)**				
No (339)	177 (52%)	1 (ref)	221 (65%)	1 (ref)
Yes (67)	47 (70%)	2.0 (1.1 to 3.8)	46 (69%)	0.8 (0.4 to 1.7)
**Discussed the problem with somebody working in the primary care service (0 missing)**				
No/don't know/missing (197)	99 (50%)	1 (ref)	119 (60%)	1 (ref)
Yes (209)	125 (60%)	1.3 (0.9 to 2.0)	148 (71%)	1.5 (0.9 to 2.4)
**Service used (1 missing)**				
GP surgery (286)	159 (56%)	1 (ref)	186 (65%)	1 (ref)
Dental surgery (36)	17 (46%)	0.8 (0.3 to 1.7)	12 (33%)	1.1 (0.5 to 2.7)
Walk in clinic (16)	7 (44%)	1.0 (0.4 to 3.0)	10 (63%)	1.7 (0.5 to 5.7)
Ambulance/A&E/ OOH (20)	13 (65%)	2.0 (0.7 to 5.5)	15 (75%)	3.8 (1.0 to 14.1)
Pharmacy (18)	15 (83%)	2.0 (0.5 to 7.8)	3 (17%)	1.0 (0.2 to 4.3)
Other (29)	12 (41%)	0.7 (0.3 to 1.7)	14 (48%)	1.4 (0.6 to 3.4)
**Problem related to (0 missing)**				
A. Healthcare delivery system (65)	25 (38%)	1 (ref)	24 (37%)	1 (ref)
B. Investigation (63)	29 (46%)	1.2 (0.6 to 2.5)	42 (67%)	3.4 (1.5 to 7.6)
C. Treatment process (100)	73 (73%)	3.7 (1.8 to 7.7)	85 (85%)	11.0 (4.6 to 26.5)
D. Communication (66)	36 (55%)	1.8 (0.9 to 3.7)	37 (56%)	2.0 (0.9 to 4.2)
E. Clinical knowledge or skills (43)	23 (53%)	1.8 (0.8 to 4.2)	30 (70%)	3.3 (1.3 to 8.4)
F. Diagnosis (56)	34 (61%)	2.5 (1.1 to 5.4)	79 (21%)	6.2 (2.6 to 15.1)
G. Wrong treatment decision (4)	2 (50%)	1.4 (0.2 to 11.5)	3 (75%)	3.9 (0.4 to 41.7)

For peer review only - http://bmjopen.bmj.com/site/about/guidelines.xhtml

H. Other (9)	2 (22%)	0.5 (0.1 to 2.8)	2 (22%)	0.4 (0.1 to 2.2)
**Relevant condition (0 missing)**	**Frequency (%)**	**Unadjusted odds ratio[3]**	**Frequency (%)**	**Unadjusted odds ratio[3]**
All other conditions (47)	24 (51%)	1 (ref)	29 (19%)	1 (ref)
Cardiovascular (8)	7 (88%)	6.7 (0.8 to 58.9)	8 (100%)	-[4]
Diabetes (32)	20 (63%)	1.6 (0.6 to 4.0)	24 (75%)	1.8 (0.7 to 5.0)
Cancer (7)	7 (100%)	-[4]	7 (100%)	-[4]
Mental health (18)	6 (33%)	0.5 (0.2 to 1.5)	15 (83%)	3.1 (0.8 to 12.2)
Dental (33)	16 (48%)	0.9 (0.4 to 2.2)	24 (73%)	1.7 (0.6 to 4.3)
Accidental injury (17)	10 (59%)	1.4 (0.4 to 4.2)	12 (71%)	1.5 (0.4 to 4.9)
Infectious (12)	8 (67%)	1.9 (0.5 to 7.2)	10 (83%)	3.1 (0.6 to 15.8)
Pain/discomfort (15)	8 (53%)	1.1 (0.3 to 3.5)	5 (30%)	0.3 (0.1 to 1.1)
Skin (12)	5 (42%)	0.7 (0.2 to 2.5)	4 (33%)	0.3 (0.1 to 1.2)
Respiratory (13)	9 (69%)	2.2 (0.6 to 8.0)	12 (92%)	7.4 (0.9 to 62.2)
Pregnancy (8)	6 (75%)	2.9 (0.5 to 15.7)	8 (100%)	-[4]
Musculoskeletal (34)	11 (32%)	0.5 (0.2 to 1.1)	16 (47%)	0.6 (0.2 to 1.3)
Ear, nose and throat (9)	6 (67%)	1.9 (0.4 to 8.6)	4 (44%)	0.5 (0.1 to 2.1)
Not relevant/not known (141)	81 (57%)	1.3 (0.7 to 2.5)	89 (63%)	1.1 (0.5 to 2.1)

[1]median score higher than “probably” or at least one score of “very likely or certain”, see Table B

[2]median score higher than “possibly” or at least one score of “probably” or higher, see Table B

[3]unadjusted OR shown due to collinearity between dental problems and dental service

[4]predicts success perfectly (100% of scenarios in this category)

For peer review only - http://bmjopen.bmj.com/site/about/guidelines.xhtml

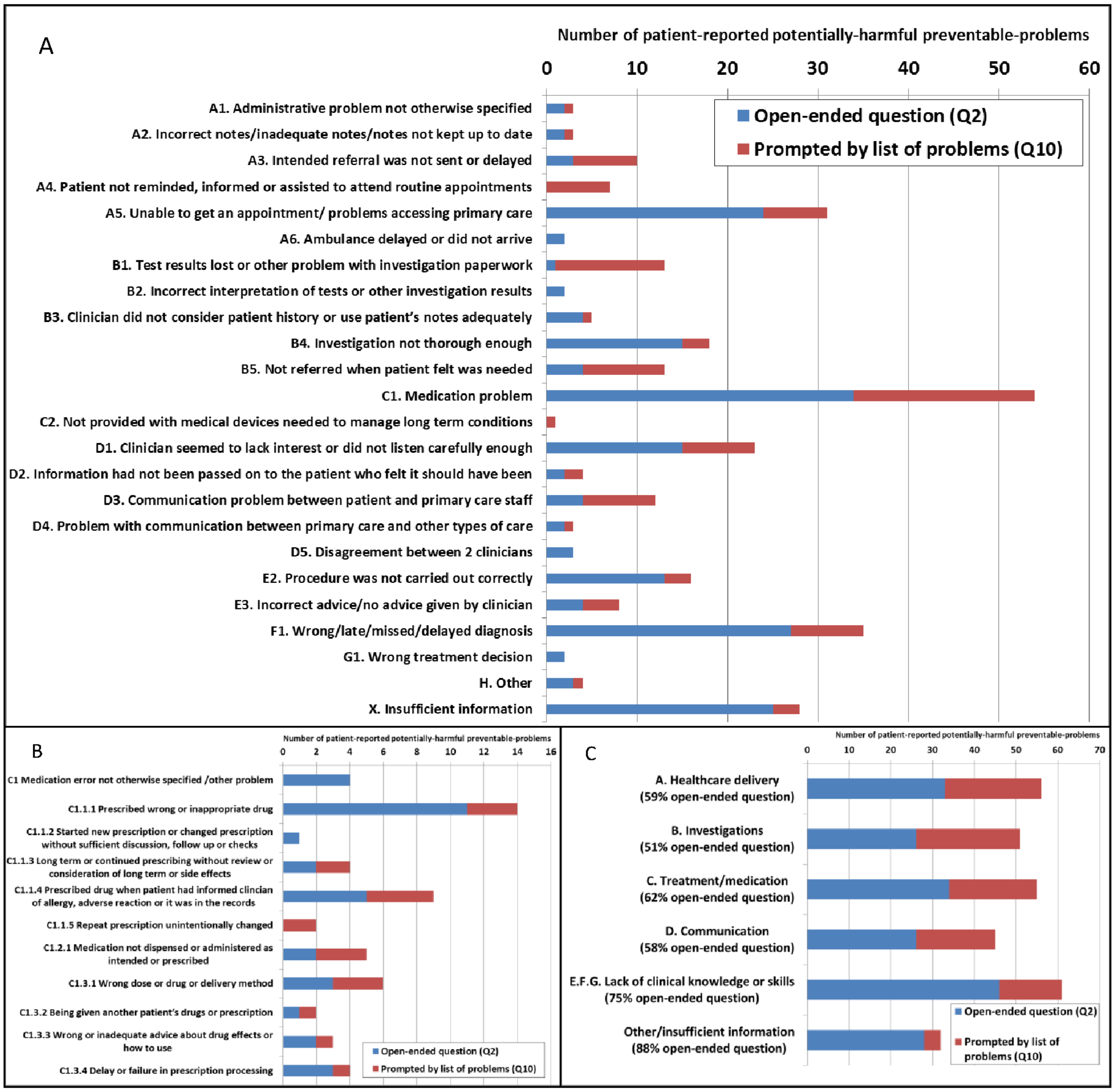

Fig C. Numbers of patient-perceived problems occurring in the last 12 months categorised according to the patient's description (see Table 2) and route through survey *i.e.* originated from open-ended question (Q2) or prompted by list of potential safety problems (Q10). See online Appendix 2 for details of coding; A coded to 2 levels, B medication problems coded to 3 levels, C coded to 1 level

For peer review only - http://bmjopen.bmj.com/site/about/guidelines.xhtml

Appendix 4.

Patient reported scenarios occurring during the past 12 months that GPs scored as higher likelihood to be a potentially-unsafe preventable-problem in primary care (median score is higher than "possibly" and at least 2 GPs gave a score or one GP scored "very likely or certain") from the Ipsos MORI survey

Abbreviations: PPI member of the public, GP general practitioner

Scenario1/2567**. Ambulance**

Briefly describe the mistake or problem and how it happened. *"Heart attack, an ambulance was called and waited an hour and three quarters to arrive"*

Could the mistake or problem have been avoided? If so how? "*The ambulance service needs to be sorted out"*

Were you able to talk about the mistake or problem with anybody working in the primary care service? *"No I was too distressed to discuss the problem or error"*

Patient-reported prospect of harm: health could have been made worse had someone not noticed a problem or error

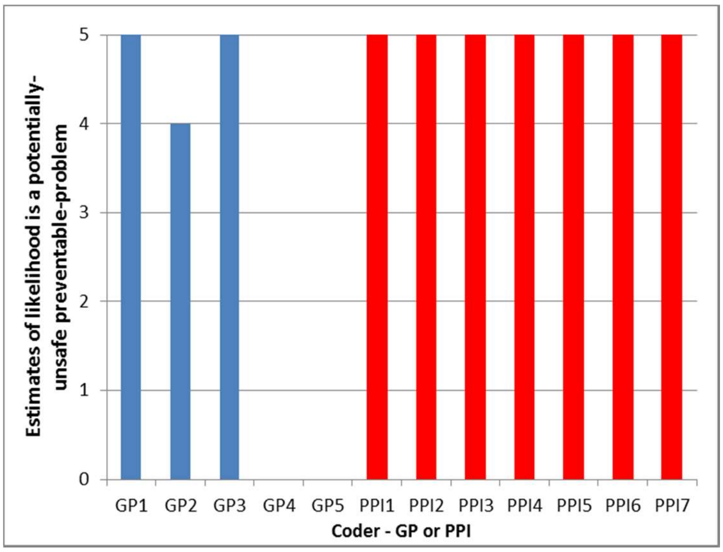

5=very likely or certain, 4=probably, 3=possibly, 2=unlikely, 1=definitely not, 0 = insufficient information or don't know

Patient-perspective problem-type code: A6. Ambulance delayed or did not arrive

Scenario1/3292**. GP surgery**

Briefly describe the mistake or problem and how it happened. *"I had an ongoing stomach complaint. The GP kept prescribing a steroid treatment but the pharmacist refused to give it to me. He said it was dangerous and I had to get different medication. The GP prescribed an alternative but the pharmacist pointed out that the steroid was supposed to be a short term treatment and that the GP had been prescribing it for over a year."*

Could the mistake or problem have been avoided? If so how? *"The GP obviously didn't read the notes. The GP was probably pushed for time and just wanted to get me out (maybe?)"*

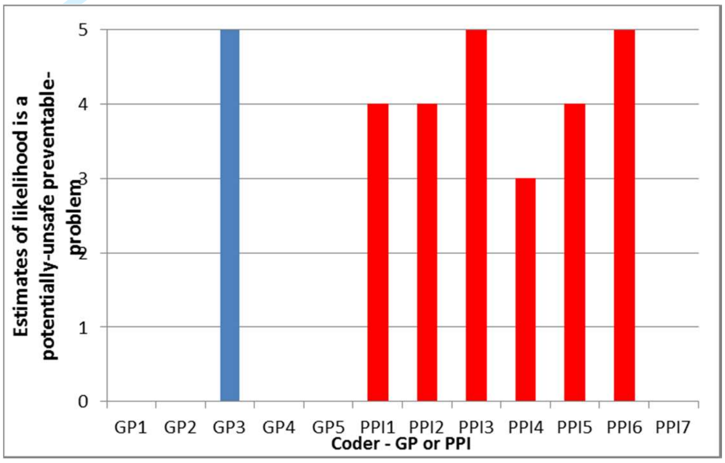

5=very likely or certain, 4=probably, 3=possibly, 2=unlikely, 1=definitely not, 0 = insufficient information or don't know

Were you able to talk about the mistake or problem with anybody working in the primary care service? *"No I was not concerned about the problem or error"*

Patient-reported prospect of harm: prompted via Q10 (Box 1 main paper)

Patient-perspective problem-type code: C1.1.3 Long term or continued prescribing without review or consideration of long term or side effects; B3 Clinician did not consider patient history sufficiently/did not use patient's notes adequately

Scenario2/2836. **GP surgery**

Briefly describe the mistake or problem and how it happened. *"Participant was prescribed penicillin and it was stated in notes that patient was allergic to penicillin"*

Could the mistake or problem have been avoided? If so how? *"It was avoided as participant didn't take prescription and was prescribed something else"*

Were you able to talk about the mistake or problem with anybody working in the primary care service? *"Yes with GP"*

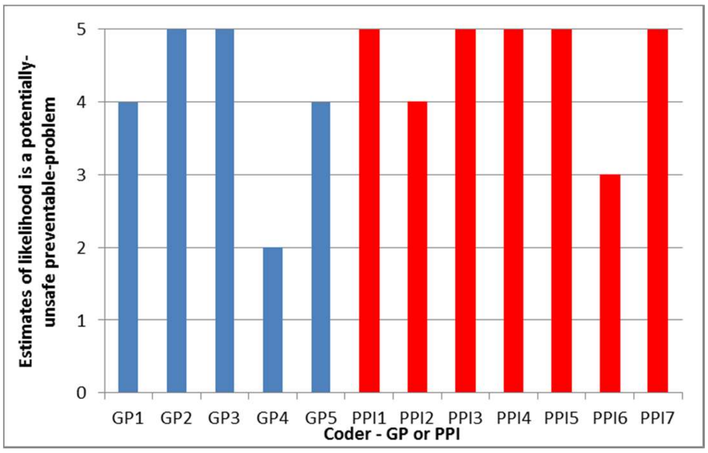

5=very likely or certain, 4=probably, 3=possibly, 2=unlikely, 1=definitely not, 0 = insufficient information or don't know

Patient-reported prospect of harm: health could have been made worse had someone not noticed a problem or error

Patient-perspective problem-type code: C1.1.4 Prescribed drug when should have known contra-indicated *e.g.* patient had informed clinician of allergy, adverse reaction or it was in the records

Scenario2/1875. **Optician**

Briefly describe the mistake or problem and how it happened. *"Started suffered blurred vision in left eye, eye was bloodshot. Went to get eye check and was sold eye drops to treat infection, told would take five days. After five days of treatment problem was made worse until vision was affected, GP referred to eye clinic diagnosed with iritis. Further treatment at eye clinic cleared up the issue."*

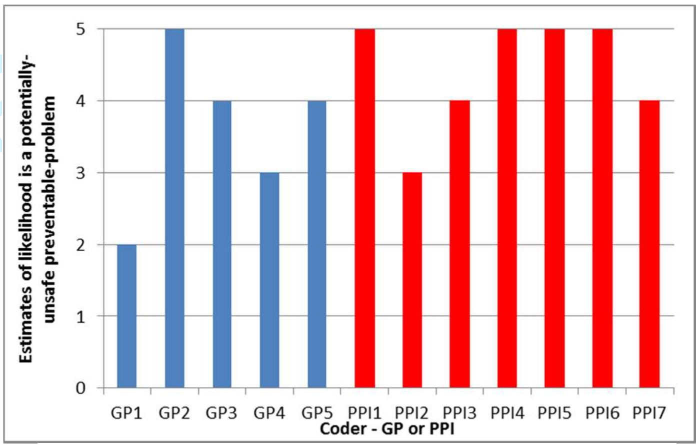

5=very likely or certain, 4=probably, 3=possibly, 2=unlikely, 1=definitely not, 0 = insufficient information or don't know

Could the mistake or problem have been avoided? If so how? *"If optometrists had spotted that iris was stuck, had a bit more professional care rather than trying to flog over-the-counter eye drops to clear up infection that wasn't there"*

Were you able to talk about the mistake or problem with anybody working in the primary care service? *"Yes, spoke to GP, immediate referral to eye clinic for treatment"*

Patient-reported prospect of harm: health was actually made worse by a problem or error that could have been prevented

Patient-perspective problem-type code: F1 Wrong/late/missed/delayed diagnosis

For peer review only

Scenario1/1527. **GP surgery**

Briefly describe the mistake or problem and how it happened. *"Contra-indication with a medicine that was not noticed at time of prescription but was noticed by the participant before they started taking the medicine"*

Could the mistake or problem have been avoided? If so how? "*The contra-indication should have been flagged up on the computer at the time of prescription but it wasn't"*

Were you able to talk about the mistake or problem with anybody working in the primary care service? "Yes, *secretary and a GP"*

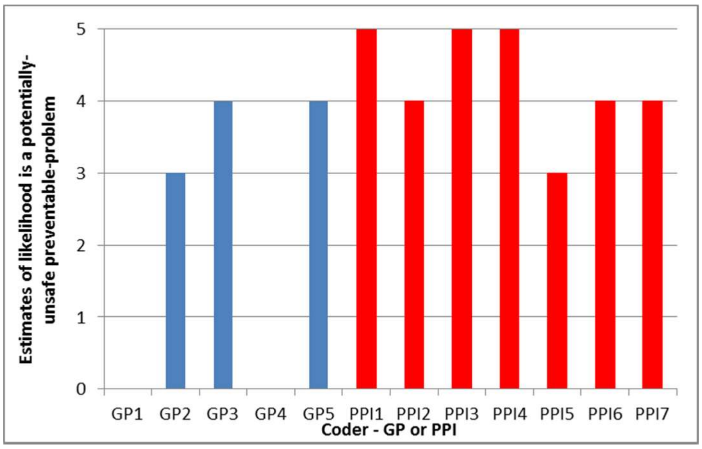

5=very likely or certain, 4=probably, 3=possibly, 2=unlikely, 1=definitely not, 0 = insufficient information or don't know

Patient-reported prospect of harm: health could have been made worse had someone not noticed a problem or error

Patient-perspective problem-type code: C1.1.4 Prescribed drug when should have known contra-indicated *e.g.* patient had informed clinician of allergy, adverse reaction or it was in the records

Scenario1/2412. **GP surgery**

Briefly describe the mistake or problem and how it happened. *"Went with a lump to GP. He referred me under the 2 week NICE guidelines. The communication went wrong and I chased it up myself or would have remained sat here. I ended up being diagnosed with cancer but I intervened in time."*

Could the mistake or problem have been avoided? If so how? *"Policies & procedures in place now. If you're sent an appointment that place needs to send a confirmation. That's what happened to stop it happening again."*

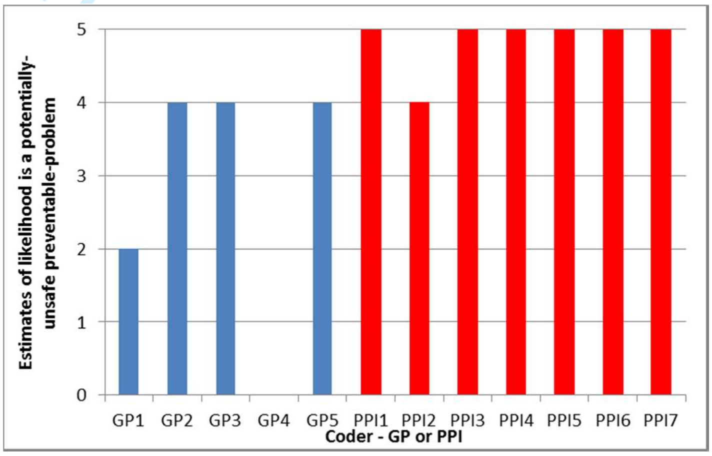

5=very likely or certain, 4=probably, 3=possibly, 2=unlikely, 1=definitely not, 0 = insufficient information or don't know

Were you able to talk about the mistake or problem with anybody working in the primary care service? *"GP investigated it as a significant event. Said if not satisfied come in and chat to us. I had apology from GP."*

Patient-reported prospect of harm: health could have been made worse had someone not noticed a problem or error

Patient-perspective problem-type code: A3. Intended referral was not sent or delayed

Scenario2/2999. Pharmacy

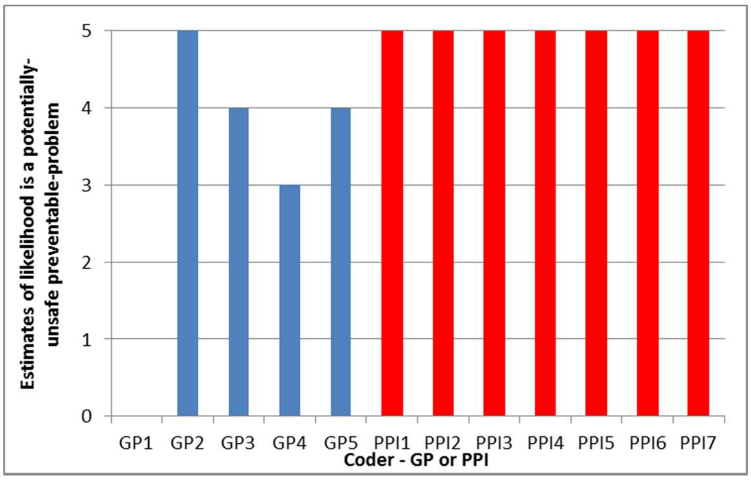

5=very likely or certain, 4=probably, 3=possibly, 2=unlikely, 1=definitely not, 0 = insufficient information or don't know

Briefly describe the mistake or problem and how it happened. *"They gave me the wrong tablets and they were heart pills - beta blockers- but I thought they were sleeping pills. I looked at the patient information and thought why am I not sleeping and realised they were for people who had had a heart attack. I was taking them for 6 weeks then I phoned the doctor and he came straight away. The pharmacist no longer works there."*

Could the mistake or problem have been avoided? If so how? *"She just put up the wrong tablets. She should have dispensed the right pills as on my prescription"*

Were you able to talk about the mistake or problem with anybody working in the primary care service? *"Yes, doctor - he gave me the right ones"*

Patient-reported prospect of harm: health could have been made worse had someone not noticed a problem or error

Patient-perspective problem-type code: C1.2.1 Medication not dispensed or administered as intended or prescribed

Scenario1/2410. Out of hours care

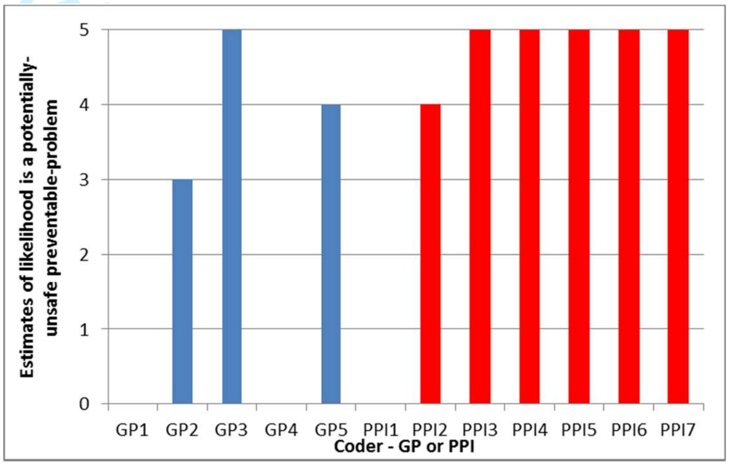

5=very likely or certain, 4=probably, 3=possibly, 2=unlikely, 1=definitely not, 0 = insufficient information or don't know

Briefly describe the mistake or problem and how it happened. *"Banged foot at work, hurt a lot, for few days got worse"*

Could the mistake or problem have been avoided? If so how? *"if they had listened to me properly, they didn't therefore toe got amputated for no reason"*

Were you able to talk about the mistake or problem with anybody working in the primary care service? *"Yes, triage nurse"*

Patient-reported prospect of harm: health could have been made worse had someone not noticed a problem or error

Patient-perspective problem-type code: B4. Investigation not thorough enough; D1. Clinician seemed to lack interest in the patient's health problem or did not listen carefully enough

Scenario2/1432. GP surgery

Briefly describe the mistake or problem and how it happened. *I was started on warfarin and was fainting and bleeding rectally. I was in town the first time I passed out and did not go to hospital. The second time I went to hospital and the problem was rectified by reducing the dose."*

Could the mistake or problem have been avoided? If so how? *"by giving a smaller dose in the first place. I was told that the amount was too much. Afterwards they put me on something else instead of warfarin."*

Were you able to talk about the mistake or problem with anybody working in the primary care service? *"Yes, doctor in hospital"*

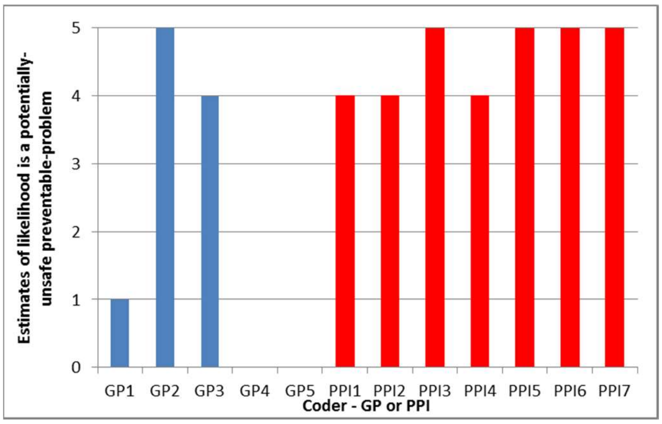

5=very likely or certain, 4=probably, 3=possibly, 2=unlikely, 1=definitely not, 0 = insufficient information or don't know

Patient-reported prospect of harm: health was actually made worse by a problem or error that could have been prevented

Patient-perspective problem-type code: C1.3.1 Wrong dose or drug or delivery method

Scenario1/1586. GP surgery

Briefly describe the mistake or problem and how it happened. *"Couldn't get appointment at GP. Health worsened, ended up in hospital with fluid on lungs and pneumonia. Was rushed in. Heart had to be stopped and restarted."*

Could the mistake or problem have been avoided? If so how? *"Had rung for appointments and asked for doctor to telephone me 3 times. They never rang. They should have signed my prescriptions so I could have medicine and should have seen me in person"*

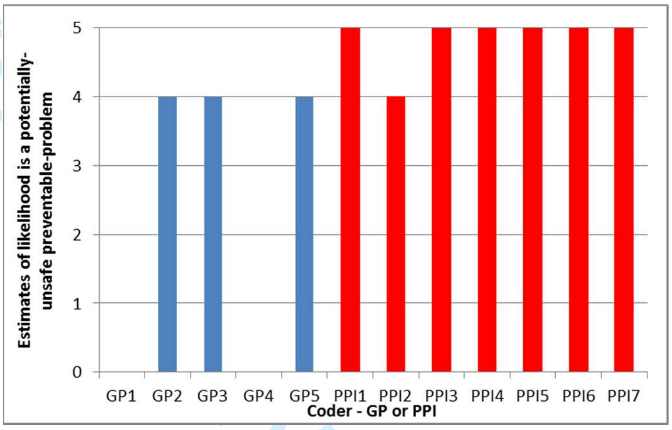

5=very likely or certain, 4=probably, 3=possibly, 2=unlikely, 1=definitely not, 0 = insufficient information or don't know

Were you able to talk about the mistake or problem with anybody working in the primary care service? *"The heart nurse from the community service complained on my behalf to the GP surgery. The chemist shop complained too about prescriptions not being signed and medicine being missed. Appointment was made at surgery to discuss with new doctor, and appointments are guaranteed as now a "supported patient"."*

Patient-reported prospect of harm: health was actually made worse by a problem or error that could have been prevented

Patient-perspective problem-type code: A5. Unable to get an appointment/other problems with making appointment; C1.3.4 Delay or failure in prescription processing

For peer review only - http://bmjopen.bmj.com/site/about/guidelines.xhtml

Scenario1/2797. **Dental surgery**

Briefly describe the mistake or problem and how it happened. *"Dentist numbed me up to pull a wrong tooth"*

Could the mistake or problem have been avoided? If so how? *"By taking care by paying attention to his own notes"*

Were you able to talk about the mistake or problem with anybody working in the primary care service? *"Yes, the dentist himself - he was apologetic."*

Patient-reported prospect of harm: a problem or error that could have been prevented but it did not make your health worse

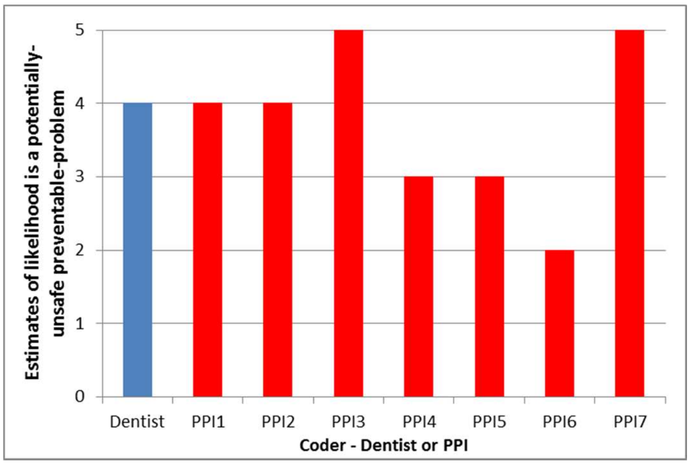

5=very likely or certain, 4=probably, 3=possibly, 2=unlikely, 1=definitely not, 0 = insufficient information or don't know

Patient-perspective problem-type code: E2.Procedure was not carried out correctly

Scenario2/1773. **GP surgery**

Briefly describe the mistake or problem and how it happened. *"Discharged from hospital following knee replacement surgery, became very ill, lost 1 stone in 7 days, requested home visit from GP as seriously concerned, doctor called by phone and was very brusque, no home visit but medication changed and 6 months later started to feel better"*

Could the mistake or problem have been avoided? If so how? *"if the doctor had come to see me in person who could have made a quicker diagnosis and could have offered some much needed support during a very traumatic time"*

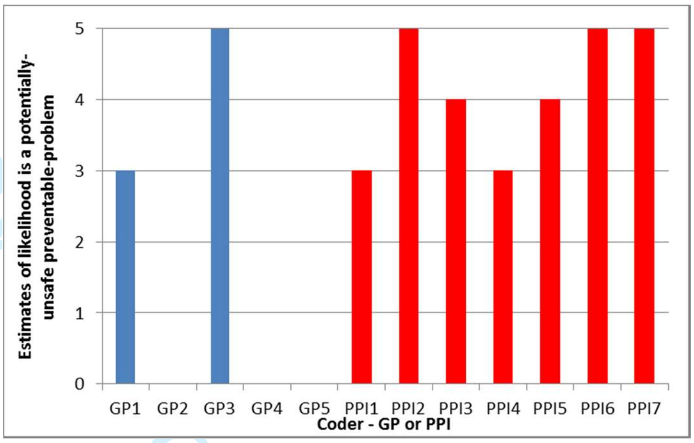

5=very likely or certain, 4=probably, 3=possibly, 2=unlikely, 1=definitely not, 0 = insufficient information or don't know

Were you able to talk about the mistake or problem with anybody working in the primary care service? *"No, I could not find anybody with whom I could discuss the problem or error"*

Patient-reported prospect of harm: suspected your health has been made worse by a problem or error that could have been prevented

Patient-perspective problem-type code: D1. Clinician seemed to lack interest in the patient's health problem or did not listen carefully enough

For peer review only

Scenario2/3423. Pharmacy

Briefly describe the mistake or problem and how it happened. *"I use a certain inhaler for COPD. I had run out without realising that I had forgotten to tick it on my repeat prescription. I spoke to the pharmacist and explained to ask him to add it for next time I picked up the repeat prescription. They agreed to do this but when I went to collect it I found that they had ordered a different medicine unrelated to COPD. I was upset because in the meantime my COPD had worsened quite quickly and was causing me distress."*

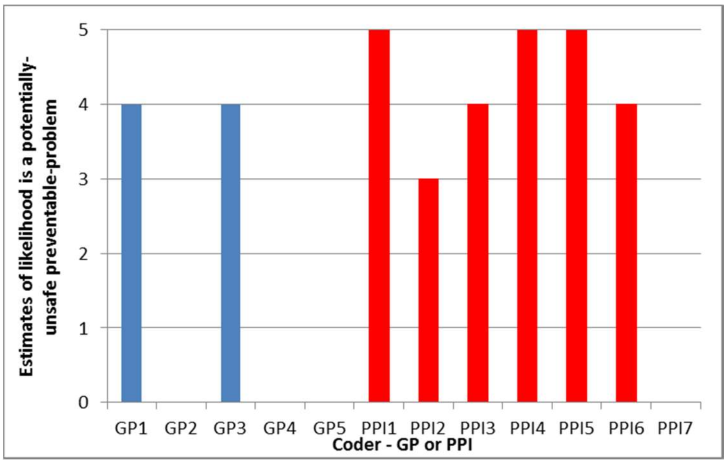

5=very likely or certain, 4=probably, 3=possibly, 2=unlikely, 1=definitely not, 0 = insufficient information or don't know

Could the mistake or problem have been avoided? If so how? *"The chemist should have made a note at the time and written down the medicine that I was asking for. If they had taken the note there and then I don't think this would have happened. I'm assuming he took a note later and failed to remember the name of the medicine correctly. We have a dreadful chemist service here."*

Were you able to talk about the mistake or problem with anybody working in the primary care service? *"No, I was so exasperated I went to my GP to order the medicine directly"*

Patient-reported prospect of harm: prompted via Q10 (Box 1 main paper)

Patient-perspective problem-type code: C1.1.5 Repeat prescription unintentionally changed

Scenario2/3011. GP surgery

Briefly describe the mistake or problem and how it happened. *"GP misdiagnosed broken jaw, went to emergency dentist then to A&E where it was operated on and fixed"*

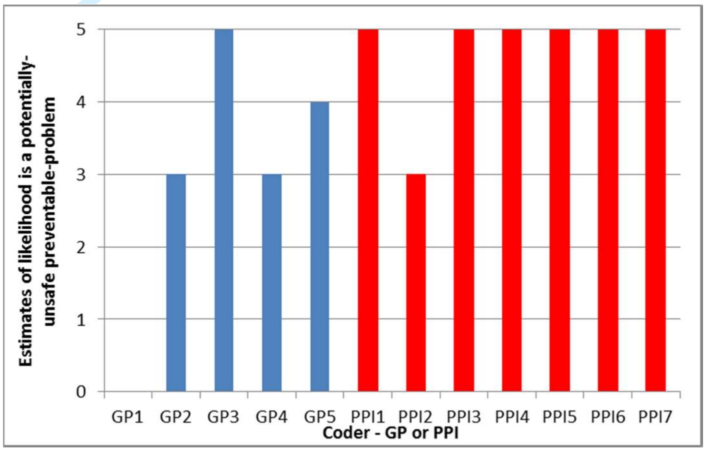

5=very likely or certain, 4=probably, 3=possibly, 2=unlikely, 1=definitely not, 0 = insufficient information or don't know

Could the mistake or problem have been avoided? If so how? "*if GP had diagnosed correctly initially"*

Were you able to talk about the mistake or problem with anybody working in the primary care service? *"made complaint to surgery and they wrote back apologising"*

Patient-reported prospect of harm: health was actually made worse by a problem or error that could have been prevented

Patient-perspective problem-type code: F1. Wrong/late/missed/delayed diagnosis

For peer review only

Scenario2/1159. **GP surgery**

Briefly describe the mistake or problem and how it happened. *"I was having severe nose bleeds for several months and was told it was hay fever. It was cancer."*

Could the mistake or problem have been avoided? If so how? *"My GP could have sent me for a CT scan as soon as my nose bleeds started."*

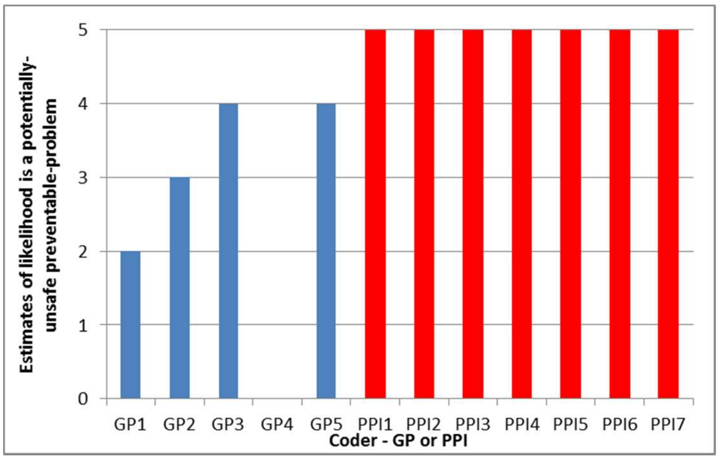

5=very likely or certain, 4=probably, 3=possibly, 2=unlikely, 1=definitely not, 0 = insufficient information or don't know

Were you able to talk about the mistake or problem with anybody working in the primary care service? *"Yes, I registered with a new GP who sent me for a scan straight away which identified my cancer."*

Patient-reported prospect of harm: health was actually made worse by a problem or error that could have been prevented

Patient-perspective problem-type code: F1. Wrong/late/missed/delayed diagnosis

Scenario2/2518. **GP surgery**

Briefly describe the mistake or problem and how it happened. *"Doctor prescribed tramadol without checking my notes. I'd already taken four pills and I rang up general enquiries at GP service to say I felt disorientated almost as if it was happening to someone else and not me. Got through to my main doctor and asked whether it was wise to take more, she said don't because you might not be alive if you do. She could see I had the wrong dose, disorientation carried on for a couple of days. It was the wrong medication."*

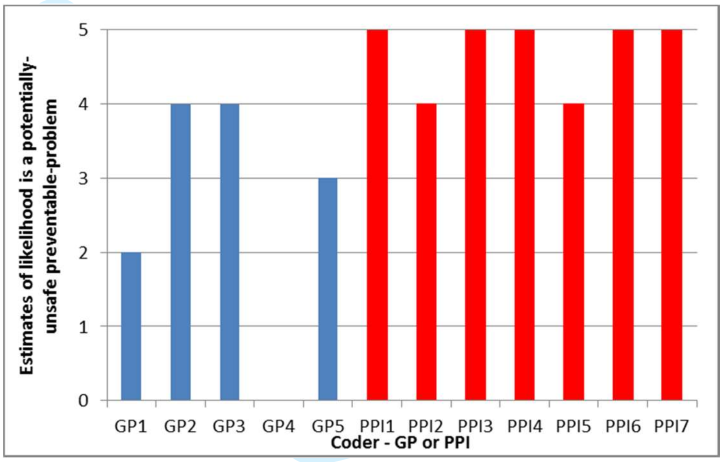

5=very likely or certain, 4=probably, 3=possibly, 2=unlikely, 1=definitely not, 0 = insufficient information or don't know

Could the mistake or problem have been avoided? If so how? *"if he had checked my notes to see what I can and can't take in terms of the actual medication"*

Were you able to talk about the mistake or problem with anybody working in the primary care service? *"discussed it with main doctor who said that she would give me some different pills to take to ease the pain for my trapped nerve in spine and back. She said she would speak to other doctor to see why it happened"*

Patient-reported prospect of harm: health was actually made worse by a problem or error that could have been prevented

Patient-perspective problem-type code: C1.3.1 Wrong dose or drug or delivery method

For peer review only - http://bmjopen.bmj.com/site/about/guidelines.xhtml

Scenario1/1947**. Out of hours care**

Briefly describe the mistake or problem and how it happened. *“Threatened miscarriage. Not given anti-D injection and notes were not consulted” (rhesus-negative patient)*

Could the mistake or problem have been avoided? If so how? *“Notes should have been checked”*

Were you able to talk about the mistake or problem with anybody working in the primary care service? *“Yes, hospital consultant who dealt effectively with situation”*

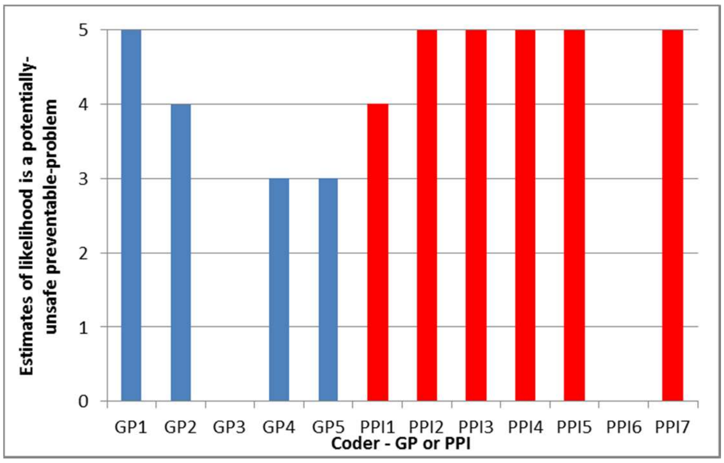

5=very likely or certain, 4=probably, 3=possibly, 2=unlikely, 1=definitely not, 0 = insufficient information or don’t know

Patient-reported prospect of harm: there was a problem or error that could have been prevented but it did not make your health worse

Patient-perspective problem-type code: B3 Clinician did not consider patient history sufficiently/did not use patient’s notes adequately

Scenario2/3009**. GP surgery**

Briefly describe the mistake or problem and how it happened. *“Had retained placenta 4 weeks after giving birth. GP dismissed it and went to A&E. Had emergency surgery”*

Could the mistake or problem have been avoided? If so how? “Yes, *by improving GP competence levels”*

Were you able to talk about the mistake or problem with anybody working in the primary care service? *“No, I was too distressed to discuss the problem or error”*

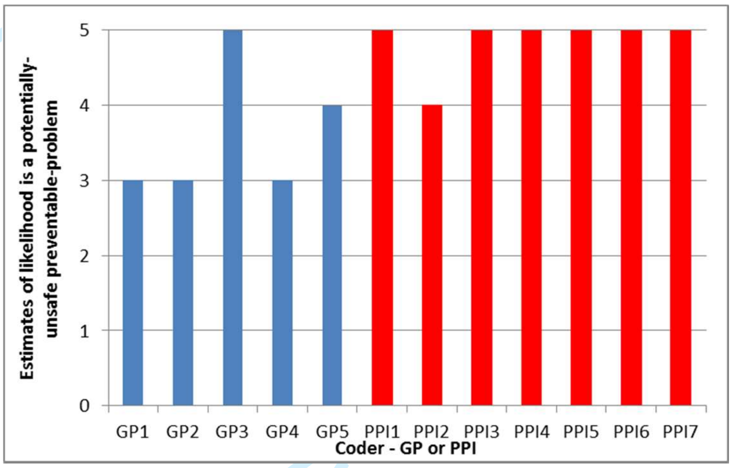

5=very likely or certain, 4=probably, 3=possibly, 2=unlikely, 1=definitely not, 0 = insufficient information or don’t know

Patient-reported prospect of harm: there was a problem or error that could have been prevented but it did not make your health worse

Patient-perspective problem-type code: F1. Wrong/late/missed/delayed diagnosis

Scenario1/2753. **GP surgery**

Briefly describe the mistake or problem and how it happened. *"I had a mole on my arm. It started to itch. I asked the GP if he'd look at it. He said it's fine. Two weeks later I had to see a dermatologist for a different reason. I asked him to look at the mole. He examined it through a magnifying glass. He said he couldn't tell if it was cancerous but recommended me to the local hospital. Two weeks later the hospital informed me the mole was cancerous. They took the mole out immediately. The point is that my GP didn't identify the possible cancer, it was coincidence that I went to the dermatologist who happened to be treating me at the time for a dry skin problem."*

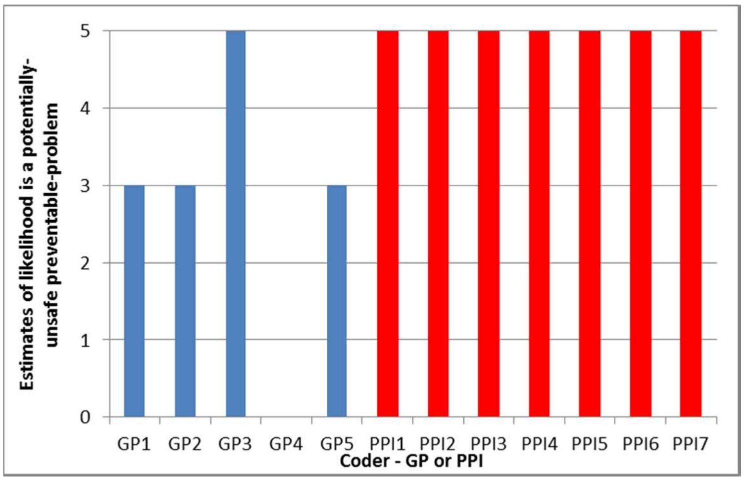

5=very likely or certain, 4=probably, 3=possibly, 2=unlikely, 1=definitely not, 0 = insufficient information or don't know

Could the mistake or problem have been avoided? If so how? *"My GP could have examined me properly rather than just looking at the mole or he could have recommended a specialist if he didn't know what it was"*

Were you able to talk about the mistake or problem with anybody working in the primary care service? *"No, I wasn't confident that they would listen/I felt anything I say would fall on deaf ears"*

Patient-reported prospect of harm: health was actually made worse by a problem or error that could have been prevented

Patient-perspective problem-type code: F1. Wrong/late/missed/delayed diagnosis

For peer review

Scenario2/1556. **GP surgery**

Briefly describe the mistake or problem and how it happened. *"appendix problem not diagnosed"*

Could the mistake or problem have been avoided? If so how? *"better diagnostic skills"*

Were you able to talk about the mistake or problem with anybody working in the primary care service? *"Yes, another GP who referred me to hospital"*

Patient-reported prospect of harm: health could have been made worse had someone not noticed a problem or error

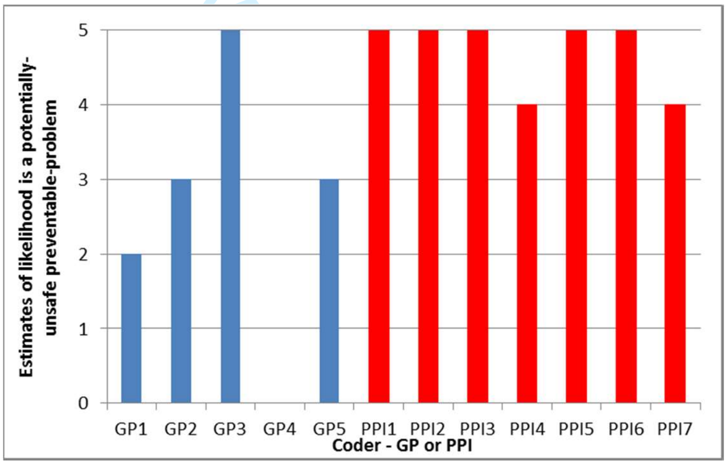

5=very likely or certain, 4=probably, 3=possibly, 2=unlikely, 1=definitely not, 0 = insufficient information or don't know

Patient-perspective problem-type code: F1. Wrong/late/missed/delayed diagnosis

Scenario2/1957**. GP surgery**

Briefly describe the mistake or problem and how it happened. *"I had something stuck into my ear, a cotton bud. I went to GP and they booked an appointment with a consultant. After 6 months I didn't hear anything from him. Luckily the cotton bud came out by itself, it could have been worse."*

Could the mistake or problem have been avoided? If so how? *"If I could have an appointment with a consultant he could have checked my ear canal"*

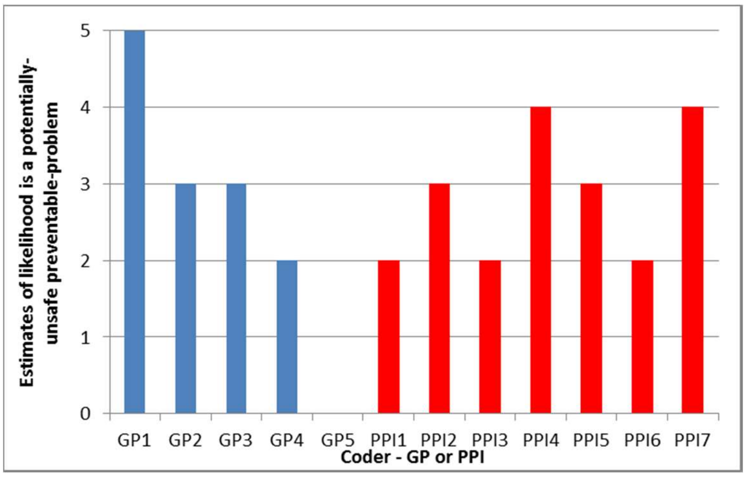

5=very likely or certain, 4=probably, 3=possibly, 2=unlikely, 1=definitely not, 0 = insufficient information or don't know

Were you able to talk about the mistake or problem with anybody working in the primary care service? *"No, I could not find anybody with whom I could discuss the problem or error"*

Patient-reported prospect of harm: health could have been made worse had someone not noticed a problem or error

Patient-perspective problem-type code: A3. Intended referral was not sent or delayed

For peer re

Scenario1/1374**. A&E**

Briefly describe the mistake or problem and how it happened. *"Basically told me problem was biliary spasms / colic but it was actually a hole in my stomach"*

Could the mistake or problem have been avoided? If so how? "If the doctor had taken heed of blood results - he ignored blood results - ended in emergency surgery"

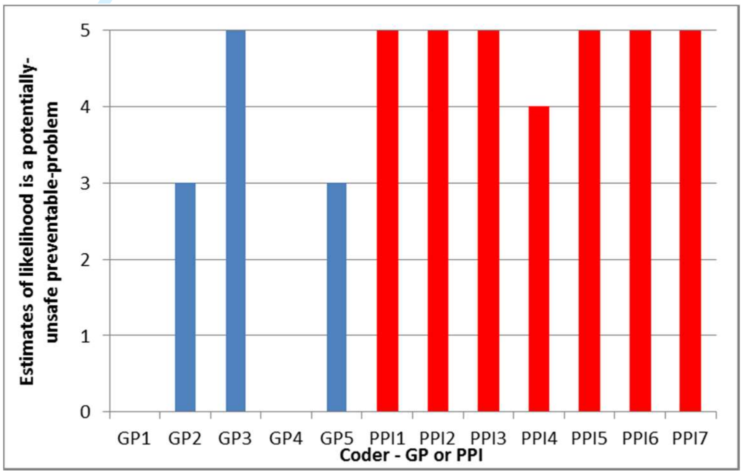

5=very likely or certain, 4=probably, 3=possibly, 2=unlikely, 1=definitely not, 0 = insufficient information or don't know

Were you able to talk about the mistake or problem with anybody working in the primary care service? *"No, I was too distressed to discuss the problem or error"*

Patient-reported prospect of harm: health was actually made worse by a problem or error that could have been prevented

Patient-perspective problem-type code: F1. Wrong/late/missed/delayed diagnosis

For peer review only - http://bmjopen.bmj.com/site/about/guidelines.xhtml

Scenario1/2268**. GP surgery**

Briefly describe the mistake or problem and how it happened. *"I have been diagnosed with bowel cancer, I knew something was wrong but over 4 visits to GP surgery over a 2 week period I was fobbed off by the GP who told me it was probably gastritis, it took 2 weeks to get a referral to a specialist"*

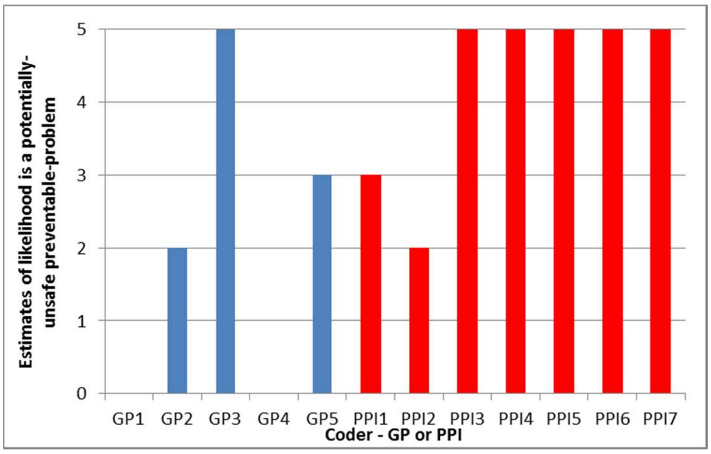

5=very likely or certain, 4=probably, 3=possibly, 2=unlikely, 1=definitely not, 0 = insufficient information or don't know

Could the mistake or problem have been avoided? If so how? *"I feel it was obvious from my appearance - massively distended stomach that - something serious was wrong with me, by the time I finally was referred I was seriously ill, this could have been avoided by an x-ray or quicker referral"*

Were you able to talk about the mistake or problem with anybody working in the primary care service? *"Yes, district nurse, who told me there is a framework in place for GPs that they have to stick to whilst diagnosing issues"*

Patient-reported prospect of harm: health was actually made worse by a problem or error that could have been prevented

Patient-perspective problem-type code: F1. Wrong/late/missed/delayed diagnosis

Scenario2/1305**. GP surgery**

Briefly describe the mistake or problem and how it happened. *"Low blood count not identified because doctor didn't do blood test. Taken to hospital, died and brought back to life"*

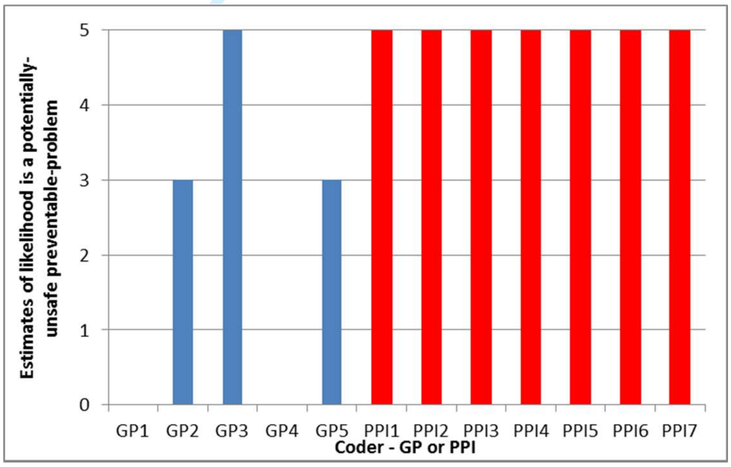

5=very likely or certain, 4=probably, 3=possibly, 2=unlikely, 1=definitely not, 0 = insufficient information or don't know

Could the mistake or problem have been avoided? If so how? *"a different drug should have been given"*

Were you able to talk about the mistake or problem with anybody working in the primary care service? *"Yes, the doctor"*

Patient-reported prospect of harm: health was actually made worse by a problem or error that could have been prevented

Patient-perspective problem-type code: B4. Investigation not thorough enough

For peer review

Scenario2/1725. GP surgery

Briefly describe the mistake or problem and how it happened. *"Had lump on back and thought was an abscess. Went to GP for antibiotics was told "nothing there, it was in my head". Three days later had to have an emergency operation to remove it."*

Could the mistake or problem have been avoided? If so how? *"by correct diagnosis"*

Were you able to talk about the mistake or problem with anybody working in the primary care service? *"No, I had the opportunity but did not feel comfortable discussing the problem or error"*

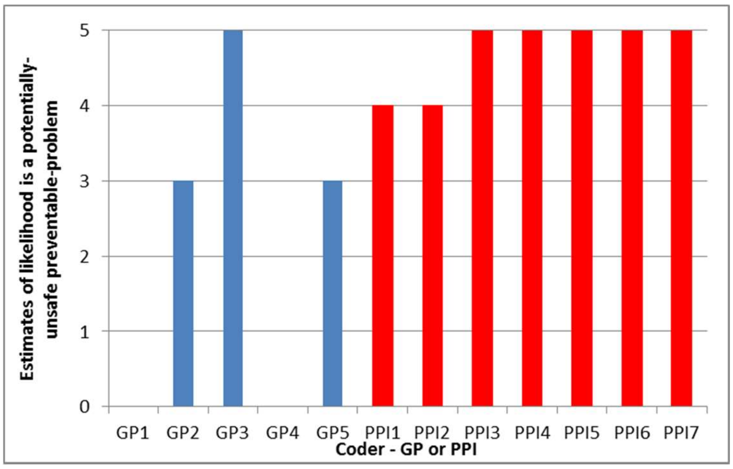

5=very likely or certain, 4=probably, 3=possibly, 2=unlikely, 1=definitely not, 0 = insufficient information or don't know

Patient-reported prospect of harm: health was actually made worse by a problem or error that could have been prevented

Patient-perspective problem-type code: F1. Wrong/late/missed/delayed diagnosis

Scenario2/1327. GP surgery

Briefly describe the mistake or problem and how it happened. "*I had gall stones and they told me it was indigestion. Pain increased over three months. Had to have an emergency operation to have my gall bladder removed. Resulted in me having damage to my liver and pancreatitis"*

Could the mistake or problem have been avoided? If so how? *"listened to me when I told them it wasn't indigestion which would have been nice. The pain felt like I was having a heart attack and not like the pain from eating something dodgy"*

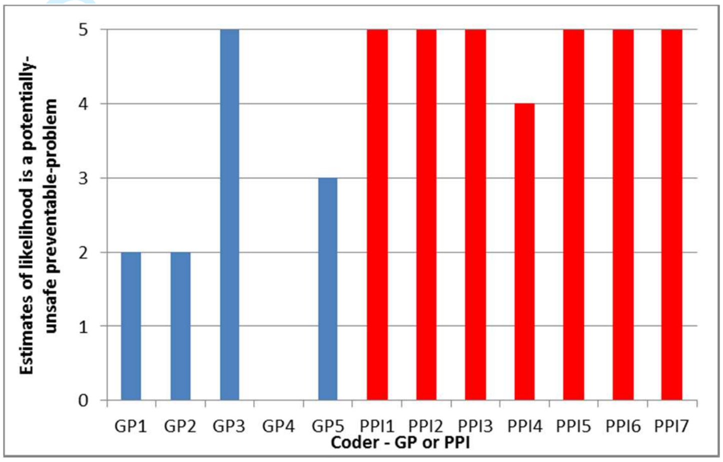

5=very likely or certain, 4=probably, 3=possibly, 2=unlikely, 1=definitely not, 0 = insufficient information or don't know

Were you able to talk about the mistake or problem with anybody working in the primary care service? *"No, I could not find anybody with whom I could discuss the problem or error"*

Patient-reported prospect of harm: health was actually made worse by a problem or error that could have been prevented

Patient-perspective problem-type code: F1. Wrong/late/missed/delayed diagnosis; D1. Clinician seemed to lack interest in the patient's health problem or did not listen carefully enough

Scenario1/1610. **GP surgery**

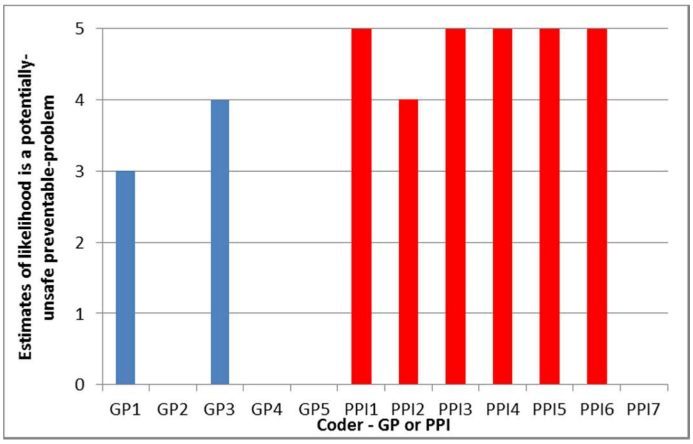

5=very likely or certain, 4=probably, 3=possibly, 2=unlikely, 1=definitely not, 0 = insufficient information or don't know

Briefly describe the mistake or problem and how it happened. *"I have arthritis and I was prescribed a medication, Diclofenac, an anti-inflammatory. After taking this, I had problems and went to the GP and had a blood test. They lost the results and I became even more ill and when I rang them, they told me I was allergic to Diclofenac and I was to stop taking it immediately. It was causing kidney failure, liver failure and high blood pressure."*

Could the mistake or problem have been avoided? If so how? *"They shouldn't have lost the results of the blood test. Later when I was feeling worse and I rang them up, they had found the results but not let me know which was another week later. They should have rung me not the other way round. That was poor communication. There should have been a better way of letting me know the results of the blood test. Luck for me, I was feeling so ill that I stopped taking the Diclofenac which they should have told me I was allergic to"*

Were you able to talk about the mistake or problem with anybody working in the primary care service? *"No, I know they're busy and there are people who need their help more than I do"*

Patient-reported prospect of harm: prompted via Q10 (Box 1 main paper)

Patient-perspective problem-type code: C1.1.3 Long term or continued prescribing without review or consideration of long term or side effects; B1. Test results lost or other problem with investigation paperwork

Scenario1/1046. **GP surgery**

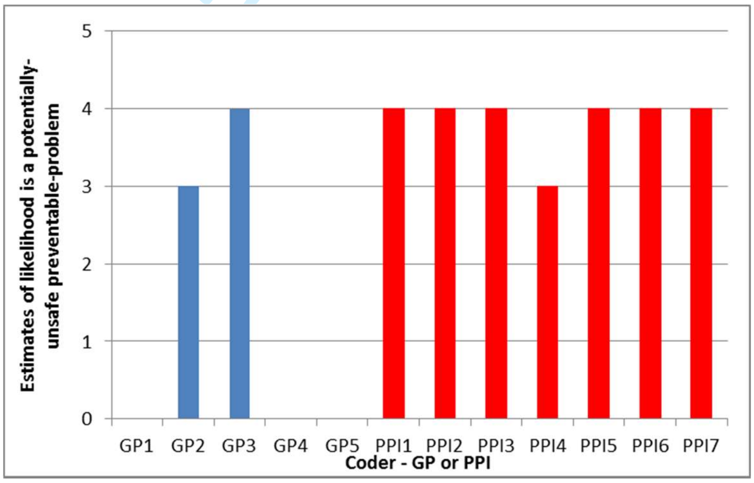

5=very likely or certain, 4=probably, 3=possibly, 2=unlikely, 1=definitely not, 0 = insufficient information or don't know

Briefly describe the mistake or problem and how it happened. *"I had stomach pains and was given the wrong medication which made it worse"*

Could the mistake or problem have been avoided? If so how? *"If I had had more tests the problem could have been avoided."*

Were you able to talk about the mistake or problem with anybody working in the primary care service? *"Yes, another doctor and they advised me to stop taking the medication"*

Patient-reported prospect of harm: health was actually made worse by a problem or error that could have been prevented

Patient-perspective problem-type code: C1.1.1 Prescribed wrong or inappropriate drug; B4. Investigation not thorough enough

For peer review only

Scenario1/3378. GP surgery

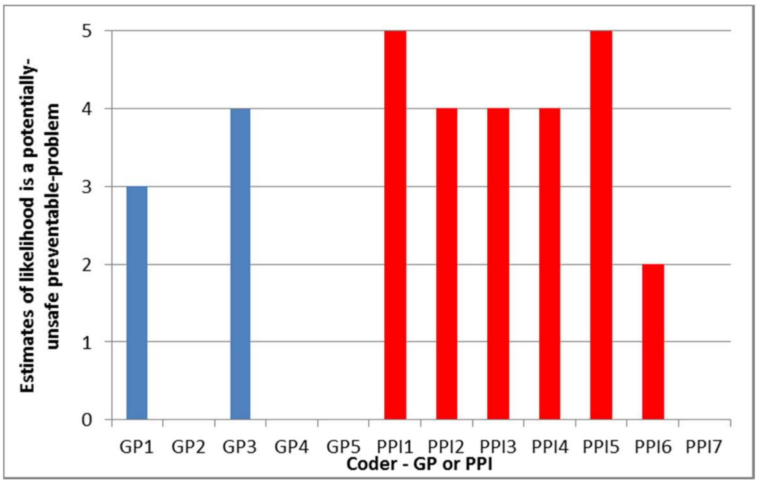

5=very likely or certain, 4=probably, 3=possibly, 2=unlikely, 1=definitely not, 0 = insufficient information or don't know

Briefly describe the mistake or problem and how it happened. *"I went to the GP and had a blood test. A month later they rang me up to tell me they had forgotten to tell me I had streptococcus and should have been on an antibiotic. In the intervening month I was ill without having taken the antibiotic"*

Could the mistake or problem have been avoided? If so how? *"Maybe they should have taken more care of their records and follow up"*

Were you able to talk about the mistake or problem with anybody working in the primary care service? *"No, I did not notice the problem or error at the time"*

Patient-reported prospect of harm: prompted via Q10 (Box 1 main paper)

Patient-perspective problem-type code: F1. Wrong/late/missed/delayed diagnosis; B1. Test results lost or other problem with investigation paperwork

Scenario1/3296. Pharmacy

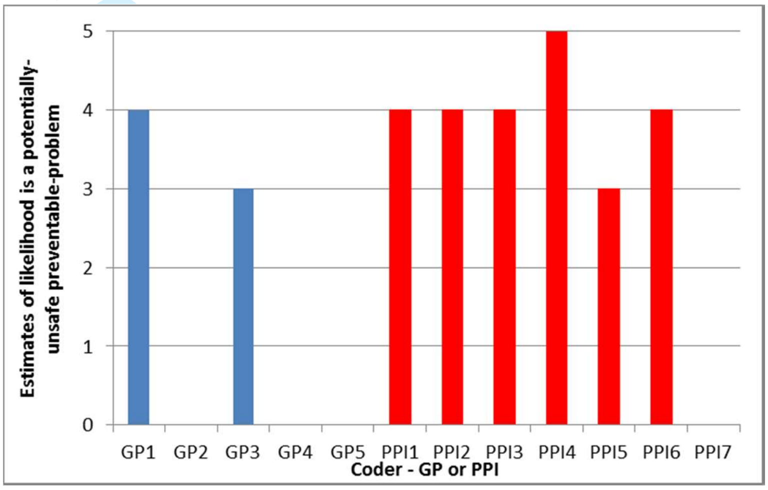

5=very likely or certain, 4=probably, 3=possibly, 2=unlikely, 1=definitely not, 0 = insufficient information or don't know

Briefly describe the mistake or problem and how it happened. *"It was routine prescription for blood pressure pills and they handed them over in a box in a stapled bag and when I got home I saw it was somebody else's medicine with my address label on. My husband took it back and they exchanged it for the correct medicine. About two weeks later we received a letter of apology which said the pharmacy had "put procedures in place so that the mistake wouldn't happen again". We were happy with that."*

Could the mistake or problem have been avoided? If so how? *"I don't know how the problem happened at the pharmacy. Perhaps somebody at the pharmacy could check each prescription before it's issued. Perhaps I could have checked it myself."*

Were you able to talk about the mistake or problem with anybody working in the primary care service? *"Yes, their response was the letter of apology."*

Patient-reported prospect of harm: prompted via Q10 (Box 1 main paper)

Patient-perspective problem-type code: C1.3.2 Being given another patient's drugs or prescription

For peer review

Scenario2/3425**. Pharmacy**

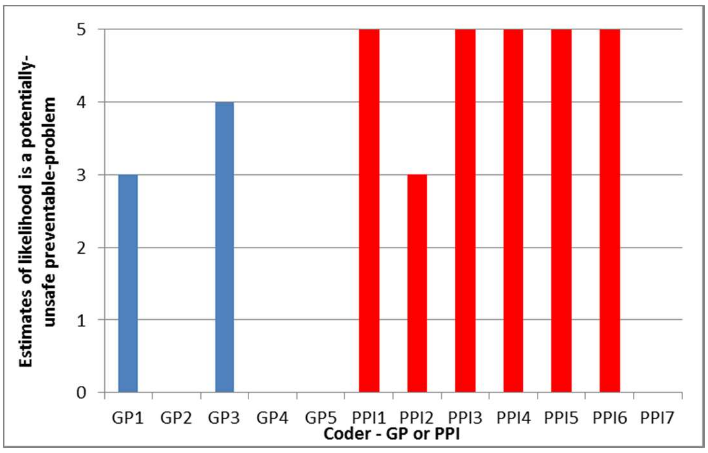

5=very likely or certain, 4=probably, 3=possibly, 2=unlikely, 1=definitely not, 0 = insufficient information or don't know

Briefly describe the mistake or problem and how it happened. *"The GP prescribed particular blood pressure tablets. The pharmacist at Boots changed the GPs prescription for a different tablet which had an adverse effect on me. It made me sick, headaches and dizziness. I went back to the GP who confirmed they were the wrong tablets and that the pharmacist isn't allowed to change a particular make of tablet. I went back to Boots and the pharmacist said they had stopped making the tablets my GP prescribed. I phoned the makers of the tablets and found that the tablets are still made. I remonstrated with the pharmacist who banned me from the shop and threatened to have me physically removed from the shop. I had been using the shop for over 40 years. I came home and phoned Boots head office and told them I would report the incident to my local newspaper and TV. I phoned the newspaper and TV wanted to film me outside the shop but a director from Boots came to my home to apologise personally and the pharmacist was forced to ring me to apologise. The pharmacist agreed that they were in breach of contract by changing the GPs prescription. When they apologised I regarded that as the end of the matter. For the last 3 months they have provided the correct tablets and on time."*

Could the mistake or problem have been avoided? If so how? *"The pharmacy is far too busy and they've exceeded their capability. Their ordering procedure means they too often run out of the correct tablets"*

Were you able to talk about the mistake or problem with anybody working in the primary care service? *"Yes, Chemist / Pharmacist, they admitted that previous medicine was wrong*

Patient-reported prospect of harm: prompted via Q10 (Box 1 main paper)

Patient-perspective problem-type code: C1.2.1 Medication not dispensed or administered as intended or prescribed

For peer review only

Scenario2/2122**. Pharmacy**

Briefly describe the mistake or problem and how it happened. *"Wrong prescription tablets issued in error, name of patient was correct but the tablets were totally incorrect."*

Could the mistake or problem have been avoided? If so how? "*Pharmacy should have taken more care"*

Were you able to talk about the mistake or problem with anybody working in the primary care service? "Yes, *spoke to pharmacist and correct prescription was issued"*

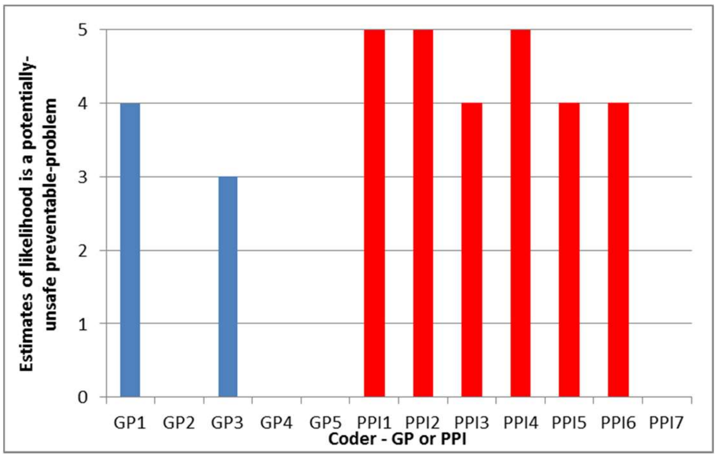

5=very likely or certain, 4=probably, 3=possibly, 2=unlikely, 1=definitely not, 0 = insufficient information or don't know

Patient-reported prospect of harm: prompted via Q10 (Box 1 main paper)

Patient-perspective problem-type code: C1.2.1 Medication not dispensed or administered as intended or prescribed

Scenario1/2503**. GP surgery**

Briefly describe the mistake or problem and how it happened. *"had ear problem and GP provided treatment for 2 years but no response to medication. Within one month of being referred and treated by specialist the problem cleared up"*

Could the mistake or problem have been avoided? If so how? "*by earlier referral to specialist"*

Were you able to talk about the mistake or problem with anybody working in the primary care service? *"No, I could not find anybody with whom I could discuss the problem or error"*

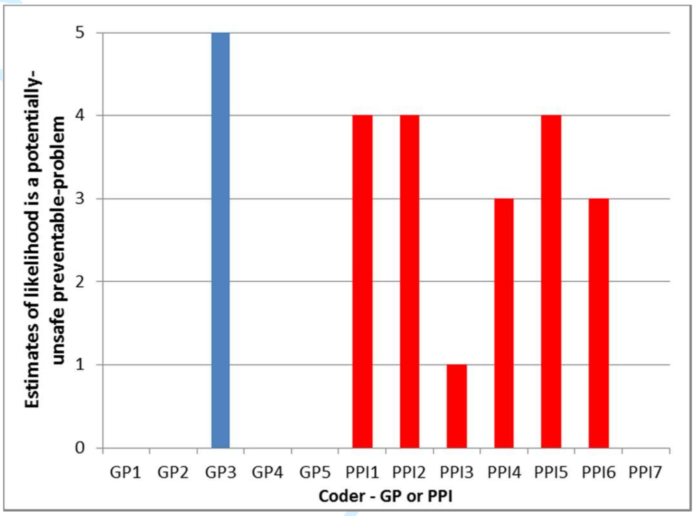

5=very likely or certain, 4=probably, 3=possibly, 2=unlikely, 1=definitely not, 0 = insufficient information or don't know

Patient-reported prospect of harm: prompted via Q10 (Box 1 main paper)

Patient-perspective problem-type code: B5. Not referred when patient felt was needed

Patient reported scenarios occurring during the past 12 months that GPs scored as higher likelihood to be a potentially-unsafe preventable-problem in primary care (median score is higher than “possibly” and at least 2 GPs gave a score or one GP scored “very likely or certain”) from the pilot study (23)

Scenario4. GP surgery

Briefly describe the mistake or problem and how it happened. *“Prescription drug, anti-inflammatory for arthritis, caused acute stomach pains & violent vomiting. Repeat prescription for twelve years without any discussion.”*

Could the mistake or problem have been avoided? If so how? “*Possible discussion about dangers of continuous taking of prescription drugs, which in the event were stopped after the incident.”*

Were you able to talk about the mistake or problem with anybody working in the primary care service? *“No I did not notice the mistake or problem at the time”*

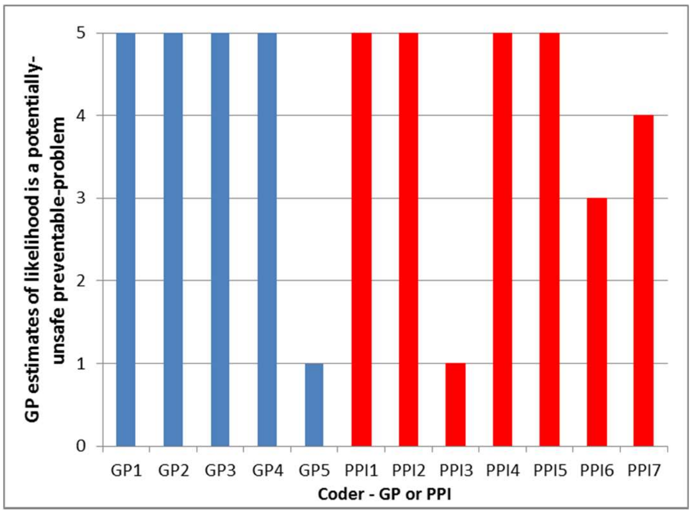

5=very likely or certain, 4=probably, 3=possibly, 2=unlikely, 1=definitely not, 0 = insufficient information or don’t know

Patient-reported prospect of harm: suspected your health has been made worse by a problem or error that could have been prevented

Patient-perspective problem-type code: C1.1.3 Long term or continued prescribing without review or consideration of long term or side effects

Scenario236. GP surgery

Briefly describe the mistake or problem and how it happened. *“Insulin type was changed by specialist but previous insulin prescribed by GP as notes had not been updated”*

Could the mistake or problem have been avoided? If so how? “*Yes GP notes should have been updated with new medication”*

Were you able to talk about the mistake or problem with anybody working in the primary care service? *“Practice manager resolved the problem and apologised”*

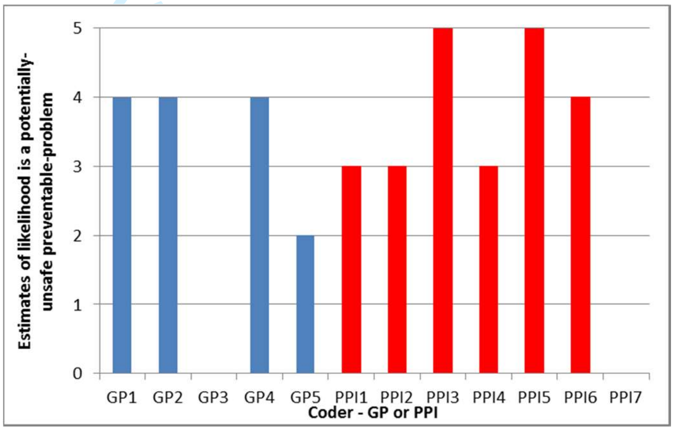

5=very likely or certain, 4=probably, 3=possibly, 2=unlikely, 1=definitely not, 0 = insufficient information or don’t know

Patient-reported prospect of harm: prompted via Q10 (Box 1 main paper)

Patient-perspective problem-type code: A2. Incorrect notes/inadequate notes/notes not kept up to date; C1.1.6 Out of date repeat prescription mistakenly re-issued

For peer review

Scenario229. GP surgery

Briefly describe the mistake or problem and how it happened. *"Two out of three Doctors not listening to what I was asking; April I had two big bleeds from my Penis, Doctor 1 did a test and gave antibiotics. Went to 2nd Doctor for Diabetic check and told him of problem - nothing except another test come back in ten days. Went to the third doctor who said the test didn't show anything but when I mentioned my feelings about a problem, he look and said yes you do have a problem. In 2 weeks I was in having tests and 3 operations for cancer."*

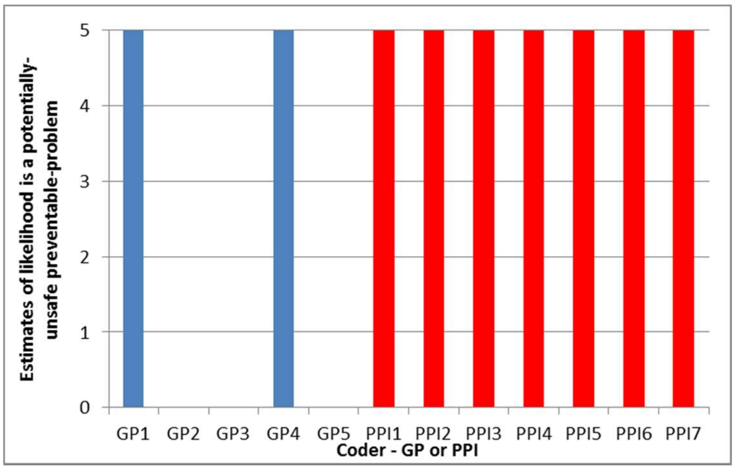

5=very likely or certain, 4=probably, 3=possibly, 2=unlikely, 1=definitely not, 0 = insufficient information or don't know

Could the mistake or problem have been avoided? If so how? "*Listen to me"*

Were you able to talk about the mistake or problem with anybody working in the primary care service? *"No, I could not find anybody with whom I could discuss the mistake or problem (The third doctor was amazing with me. He said to keep in touch and if I had any problems to ring him and he still wants me to ring him after my three operations.)"*

Patient-reported prospect of harm: suspected your health has been made worse by a problem or error that could have been prevented

Patient-perspective problem-type code: D1. Clinician seemed to lack interest in the patient's health problem or did not listen carefully enough; F1. Wrong/late/missed/delayed diagnosis

Scenario113. GP surgery

Briefly describe the mistake or problem and how it happened. *"Changed diabetes medication to an alternative which my notes from 1980's should show I respond badly to"*

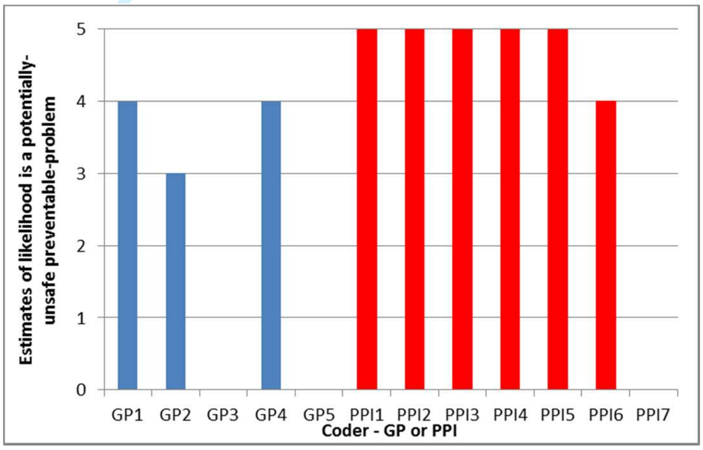

5=very likely or certain, 4=probably, 3=possibly, 2=unlikely, 1=definitely not, 0 = insufficient information or don't know

Could the mistake or problem have been avoided? If so how? "*Read the notes on every medication change but unfortunately that is unrealistic under the time restrictions on GP's. Put early notes on-line and flag medication allergies/problems."*

Were you able to talk about the mistake or problem with anybody working in the primary care service? *"Yes, my own GP who had returned from holiday"*

Patient-reported prospect of harm: suspected your health has been made worse by a problem or error that could have been prevented

Patient-perspective problem-type code: C1.1.4 Prescribed drug when should have known contra-indicated *e.g.* patient had informed clinician of allergy, adverse reaction or it was in the records

For peer review

Scenario297. GP surgery

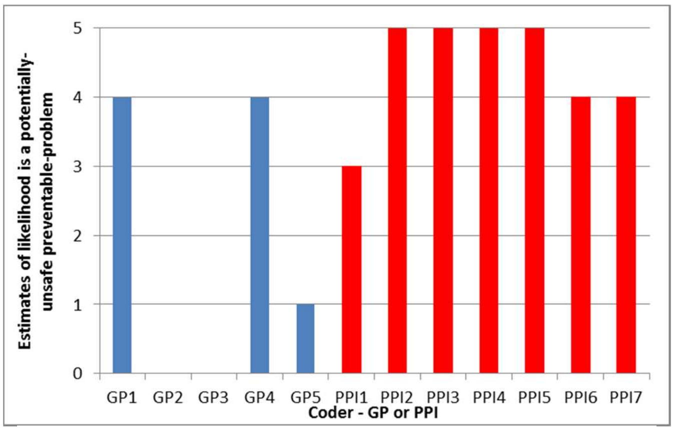

5=very likely or certain, 4=probably, 3=possibly, 2=unlikely, 1=definitely not, 0 = insufficient information or don't know

Briefly describe the mistake or problem and how it happened. *"Told the GP the medication was making my hair fall out & he kept me on it for another 3 months. I had to see another GP to get him to change my medication. In the meantime I have lost 3/4 of my hair. Not sure if it will ever grow back."*

Could the mistake or problem have been avoided? If so how? "*yes, by the GP listening to what I was saying."*

Were you able to talk about the mistake or problem with anybody working in the primary care service? *"Yes, GP"*

Patient-reported prospect of harm: suspected your health has been made worse by a problem or error that could have been prevented

Patient-perspective problem-type code: D1. Clinician seemed to lack interest in the patient's health problem or did not listen carefully enough; C1.1.3 Long term or continued prescribing without review or consideration of long term or side effects

Scenario177. GP surgery

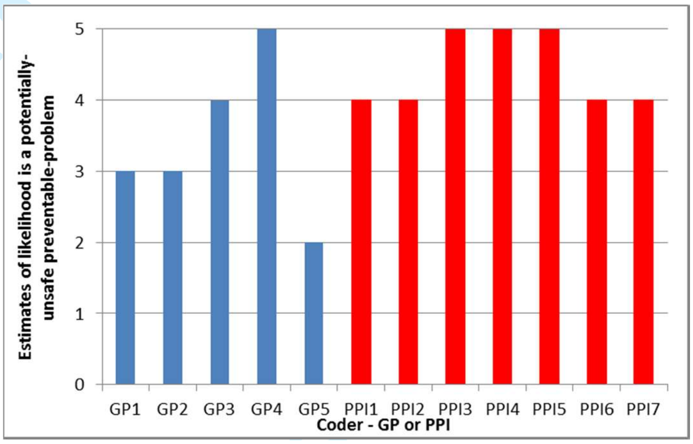

5=very likely or certain, 4=probably, 3=possibly, 2=unlikely, 1=definitely not, 0 = insufficient information or don't know

Briefly describe the mistake or problem and how it happened. *"Successfully treated for prostate cancer 2006 but suffered some loss of sexual performance; Viagra recommended BUT I take isosorbide nitrate for a following heart attack; the two are contradictory and could produce further heart problems. A routine diabetes check-up at which the sexual problem was discussed saw an automatic prescribing of Viagra; obviously without reference to my medical records."*

Could the mistake or problem have been avoided? If so how? "*Read the medical notes."*

Were you able to talk about the mistake or problem with anybody working in the primary care service? *"No; I felt I was going to cause trouble"*

Patient-reported prospect of harm: prompted via Q10 (Box 1 main paper)

Patient-perspective problem-type code: C1.1.1 Prescribed wrong or inappropriate drug

For peer review

Scenario404. GP surgery

Briefly describe the mistake or problem and how it happened. *"I was given steroids for a chest infection but not alerted to the fact they make your sugars go massively high! Within a few hours I was high and not able to bring them down, fearing a DKA I headed for the hospital to correct a very easily avoidable issue. I also attended my GP 6 years ago to be given strong antacids for pain in my stomach that was actually a DKA I was admitted to hospital a few hours later! The GP never even suggested it could be linked to my diabetes and as it was my first DKA I had no idea that's how they can feel"*

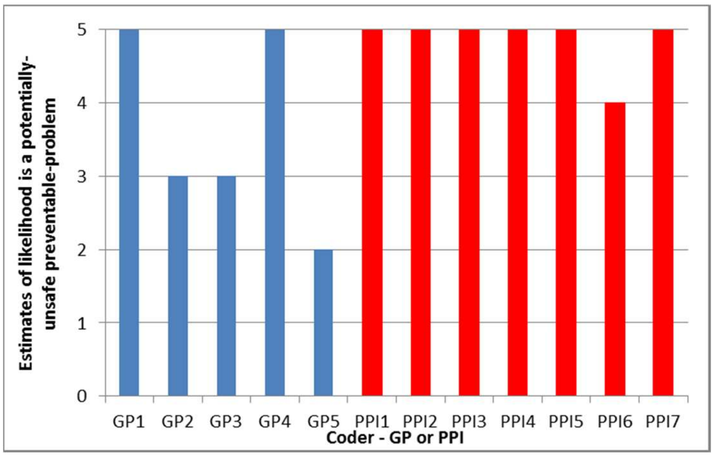

5=very likely or certain, 4=probably, 3=possibly, 2=unlikely, 1=definitely not, 0 = insufficient information or don't know

Could the mistake or problem have been avoided? If so how? "*Both could have been avoided The steroids - if the prescribing nurse had considered my diabetes I'd have been given proper advice as to how to deal with them as a diabetic or given different meds. The DKA simple questions or explanation as to how DKAs can present would have made me family and the doctor realise I was in trouble."*

Were you able to talk about the mistake or problem with anybody working in the primary care service? *"I wrote a letter to the surgery concerning the steroids anonymously to alert them of my concern and the DKA. I was too poorly to even consider seeking correction or explanation"*

Patient-reported prospect of harm: health was actually made worse by a problem or error that could have been prevented

Patient-perspective problem-type code: C1.1.4 Prescribed drug when should have known contra-indicated *e.g.* patient had informed clinician of allergy, adverse reaction or it was in the records; E3. Incorrect advice/no advice given by clinician

Scenario29. GP surgery

Briefly describe the mistake or problem and how it happened. *"reception staff making clinical decisions which were at odds with what had been discussed with my GP"*

Could the mistake or problem have been avoided? If so how? "*Yes, reception staff shouldn't be making clinical decisions"*

Were you able to talk about the mistake or problem with anybody working in the primary care service? *"No, had the opportunity but did not feel comfortable to discuss the mistake or problem"*

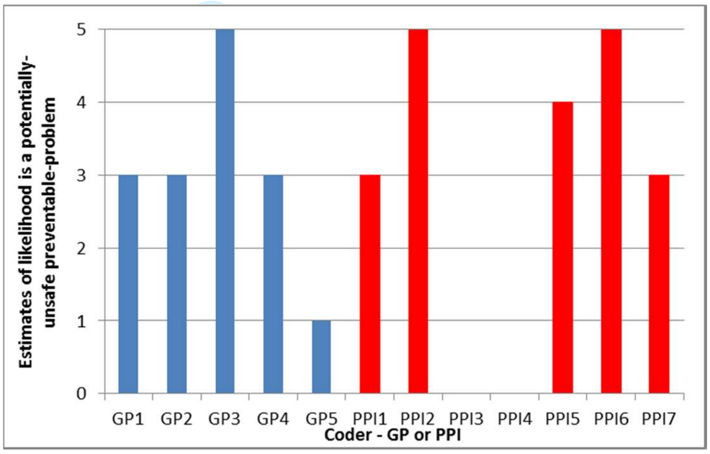

5=very likely or certain, 4=probably, 3=possibly, 2=unlikely, 1=definitely not, 0 = insufficient information or don't know

Patient-reported prospect of harm: suspected your health has been made worse by a problem or error that could have been prevented

Patient-perspective problem-type code: E1. Administrative staff seemed to make clinical decisions

Scenario621. Pharmacist

Briefly describe the mistake or problem and how it happened. *"I was given a medicine belonging to somebody else as part of my monthly repeat prescription"*

Could the mistake or problem have been avoided? If so how? "*More care and attention when checking"*

Were you able to talk about the mistake or problem with anybody working in the primary care service? *"Yes, pharmacist"*

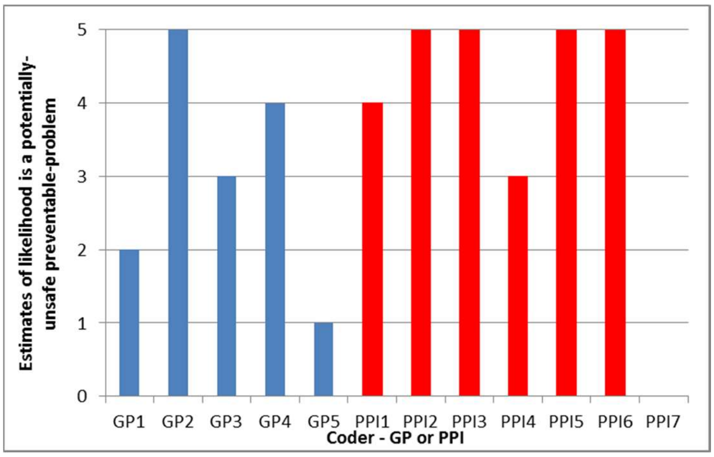

5=very likely or certain, 4=probably, 3=possibly, 2=unlikely, 1=definitely not, 0 = insufficient information or don't know

Patient-reported prospect of harm: prompted via Q10 (Box 1 main paper)

Patient-perspective problem-type code: C1.3.3 Wrong or inadequate advice about drug effects or how to use

Scenario296. GP surgery

Briefly describe the mistake or problem and how it happened. *"Poor diabetic annual review, foot check not correctly done just tested my foot pulses and nothing else"*

Could the mistake or problem have been avoided? If so how? "*Better training of staff"*

Were you able to talk about the mistake or problem with anybody working in the primary care service? *"No, had the opportunity but did not feel comfortable to discuss the mistake or problem"*

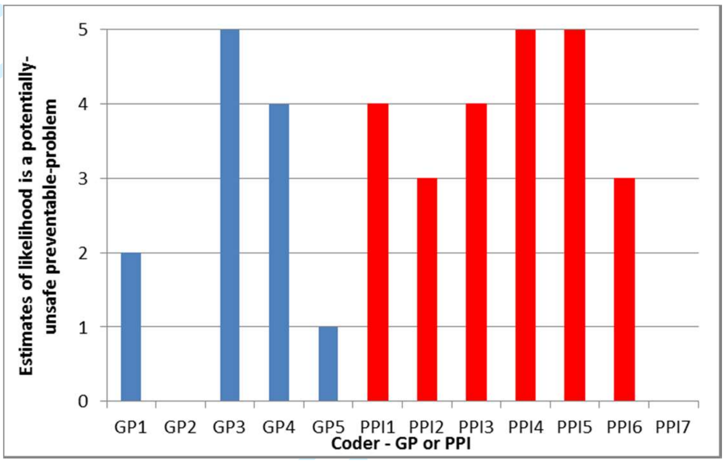

5=very likely or certain, 4=probably, 3=possibly, 2=unlikely, 1=definitely not, 0 = insufficient information or don't know

Patient-reported prospect of harm: suspected your health has been made worse by a problem or error that could have been prevented

Patient-perspective problem-type code: E2. Procedure was not carried out correctly

Scenario239. GP surgery

Briefly describe the mistake or problem and how it happened. *"Prior to a pain killing injection into my knee, I asked the GP who suggested the injection AND the GP who carried out the injection whether, as someone living with Type 1 diabetes, it would have any effect on my blood glucose levels. On both occasions, I was given an unequivocal No . In the event, within a few hours of the injection, my blood glucose rose significantly and remained high for several days. I felt unable to eat anything for 24 hours while I took on more and more insulin in order to bring my glucose levels down - I did not want to go to sleep that night simply because of the massive amount of insulin in my system."*

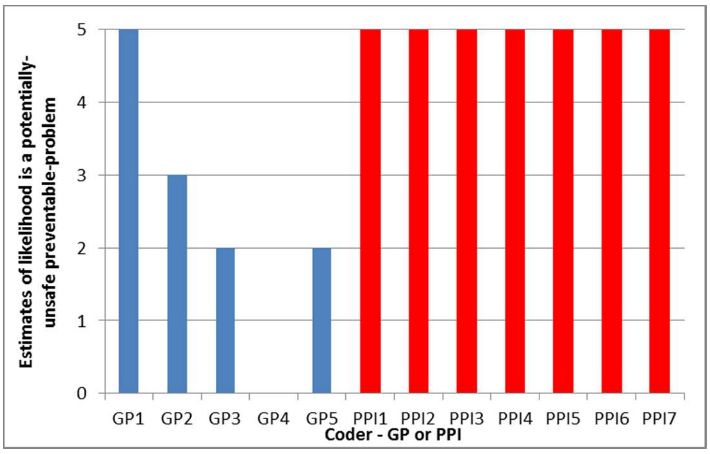

5=very likely or certain, 4=probably, 3=possibly, 2=unlikely, 1=definitely not, 0 = insufficient information or don't know

Could the mistake or problem have been avoided? If so how? "*Yes. I feel that both GPs should have a knowledge about the side effects of drugs they prescribe, administer and recommend."*

Were you able to talk about the mistake or problem with anybody working in the primary care service? *"No I could not find anybody with whom I could discuss the mistake or problem"*

Patient-reported prospect of harm: your health has been made worse by a problem or error that could have been prevented

Patient-perspective problem-type code: E3. Incorrect advice/no advice given by clinician

Scenario87. GP surgery

Briefly describe the mistake or problem and how it happened. *"GP completely overlooked symptoms and prescribed antibiotic after antibiotic without investigation or referral"*

Could the mistake or problem have been avoided? If so how? "*Yes by listening to history of complaints, carrying out appropriate tests instead of just giving antibiotics"*

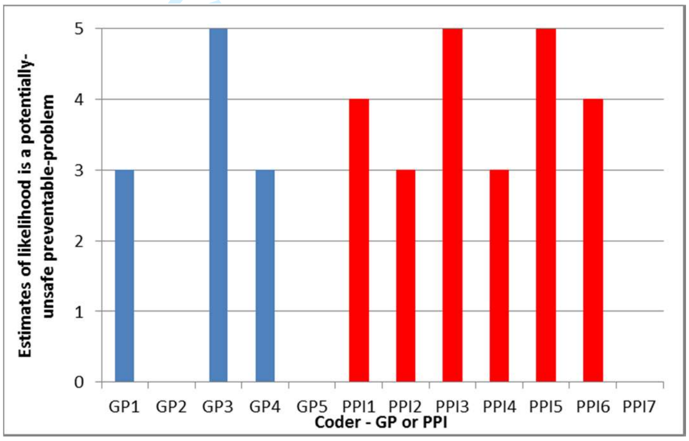

5=very likely or certain, 4=probably, 3=possibly, 2=unlikely, 1=definitely not, 0 = insufficient information or don't know

Were you able to talk about the mistake or problem with anybody working in the primary care service? *"No I did not notice the mistake or problem at the time"*

Patient-reported prospect of harm: prompted via Q10 (Box 1 main paper)

Patient-perspective problem-type code: D1. Clinician seemed to lack interest in the patient's health problem or did not listen carefully enough; F1. Wrong/late/missed/delayed diagnosis

For peer review

For peer review only - http://bmjopen.bmj.com/site/about/guidelines.xhtml

Scenario294. GP surgery

Briefly describe the mistake or problem and how it happened. *"Several times prescriptions have been incorrectly issued due to similar names for drugs or the same name with different strengths"*

Could the mistake or problem have been avoided? If so how? "*Yes, by more accurate or double data entry. Now solved by self-request using web systems."*

Were you able to talk about the mistake or problem with anybody working in the primary care service? "Yes, *they did not want to know or seem to care unless a formal complaint was made"*

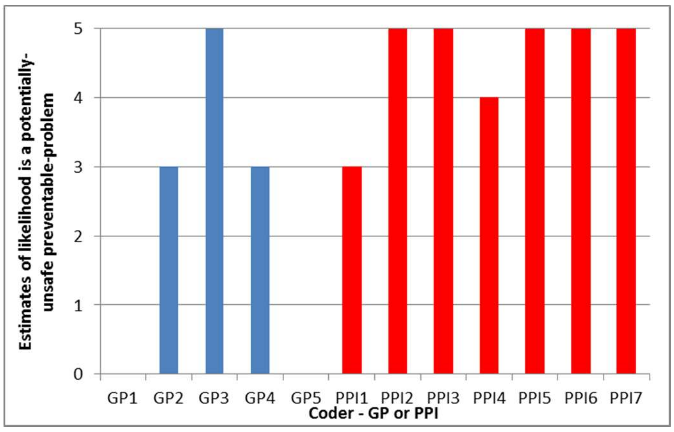

5=very likely or certain, 4=probably, 3=possibly, 2=unlikely, 1=definitely not, 0 = insufficient information or don't know

Patient-reported prospect of harm: your health has been made worse by a problem or error that could have been prevented

Patient-perspective problem-type code: C1.1.5 Repeat prescription unintentionally changed

Scenario327. GP surgery

Briefly describe the mistake or problem and how it happened. *"A simple error occurred with an incorrect prescription. When I tried to bring this to the attention of the receptionist she treated me with disdain and in a challenging manner. She then proceeded to start to read my notes aloud in the public reception area. I felt that this was unacceptable behaviour. When I tried to tackle the receptionist about her behaviour I felt as if I was under threat. It caused me to feel very stressed, frustrated and ill tempered."*

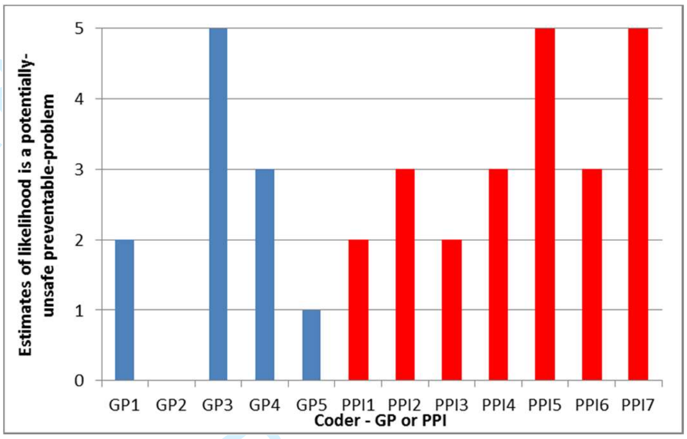

5=very likely or certain, 4=probably, 3=possibly, 2=unlikely, 1=definitely not, 0 = insufficient information or don't know

Could the mistake or problem have been avoided? If so how? "*If the receptionist had been willing to listen to what I was saying."*

Were you able to talk about the mistake or problem with anybody working in the primary care service? *"I did speak to a lady who said she was the practice manager but I felt that they were not interested in resolving the problem"*

Patient-reported prospect of harm: suspected your health has been made worse by a problem or error that could have been prevented

Patient-perspective problem-type code: D3. Communication problem between patient and primary care staff; C1 Medication error not otherwise specified /other problem

Scenario2/330. GP Surgery

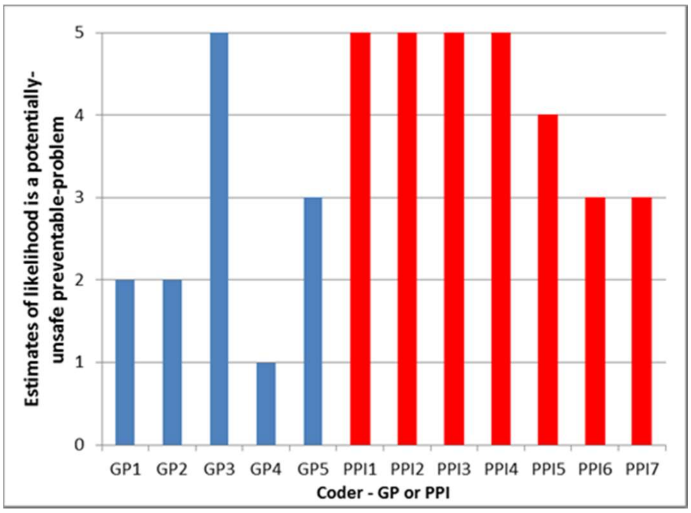

5=very likely or certain, 4=probably, 3=possibly, 2=unlikely, 1=definitely not, 0 = insufficient information or don't know

Briefly describe the mistake or problem and how it happened. *"Went to see GP because I feared the pain in one of my legs may have been Peripheral Artery Disease - hardening of the arteries, having had a (non-blood) relative who suffered from this and subsequently died - of a heart attack. Oh yes, said the GP, well, you will have it won't you? Why? I asked expecting her to say eg because you are a smoker, or maybe my age (65) or something else I wasn't aware of. But what she actually told me was 'Because you are a diabetic!' Whaaat? I exclaimed - you mean ALL diabetics will inevitably get this, and there's no way to prevent it? Yes she said and shrugged. I said 'Thanks for nothing then' and left. Instead I left, came home and went straight on-line to make an appointment with someone more sensible, which I did and after taking my leg/ankle pulses and BPs etc - he chatted to me and said he would refer me for a cardiology consultation at the hospital. This IS what I expected in the first place and now it IS being taken care of*."

Could the mistake or problem have been avoided? If so how? *"By training the GP properly in the first place"*

Were you able to talk about the mistake or problem with anybody working in the primary care service? "? *"I explained to GP 2 But I don't know what if anything was done about it, or how I could find that out."*

Patient-reported prospect of harm: your health has been made worse by a problem or error that could have been prevented

Patient-perspective problem-type code: D1. Clinician seemed to lack interest in the patient's health problem or did not listen carefully enough

For peer review only

Patient reported scenarios occurring during the past 12 months that PPIs scored as higher likelihood to be a potentially-unsafe preventable-problem in primary care compared with GPs – Ipsos MORI survey

Scenario1/1040. GP surgery

Briefly describe the mistake or problem and how it happened. *"I was suicidal, phoned the crisis team and they kept telling me that they couldn't see me because I wasn't under a psychiatrist and that made the situation worse"*

Could the mistake or problem have been avoided? If so how? *"they just simply had to say that they would see me"*

Were you able to talk about the mistake or problem with anybody working in the primary care service?

"No, I did not get to see a psychiatrist until about three months later"

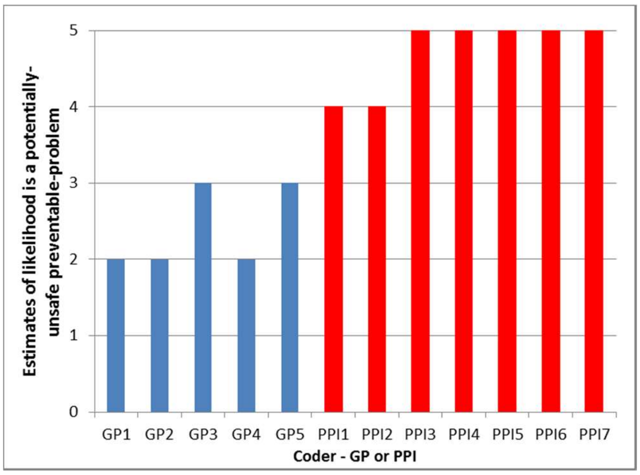

5=very likely or certain, 4=probably, 3=possibly, 2=unlikely, 1=definitely not, 0 = insufficient information or don't know

Patient-reported prospect of harm: your health has been made worse by a problem or error that could have been prevented

Patient-perspective problem-type code: A5. Unable to get an appointment/other problems with making appointment

Scenario1/1561. Physiotherapy at GP surgery

Briefly describe the mistake or problem and how it happened. *"Broken wrist after coming off pushbike"*

Could the mistake or problem have been avoided? If so how? *"Physio caused fracture, after healing, to break again"*

Were you able to talk about the mistake or problem with anybody working in the primary care service? *"Yes, another doctor in practice"*

Patient-reported prospect of harm: your health has been made worse by a problem or error that could have been prevented

Patient-perspective problem-type code: E2.Procedure was not carried out correctly

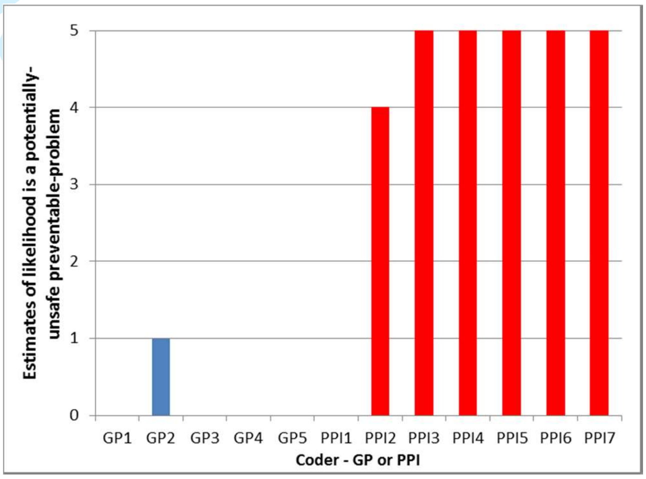

5=very likely or certain, 4=probably, 3=possibly, 2=unlikely, 1=definitely not, 0 = insufficient information or don't know

Scenario1/1578. GP surgery

Briefly describe the mistake or problem and how it happened. *"Given some medication that brought about a nervous breakdown and crisis team attended within 4 hours. Seeing mental health social worker each week now as a result. Hearing voices and seeing things which I didn't before this medication."*

Could the mistake or problem have been avoided? If so how? *"GP could have listened more carefully and not changed my medication"*

Were you able to talk about the mistake or problem with anybody working in the primary care service? "*Yes, the crisis mental health team/the psychologist and social worker"*

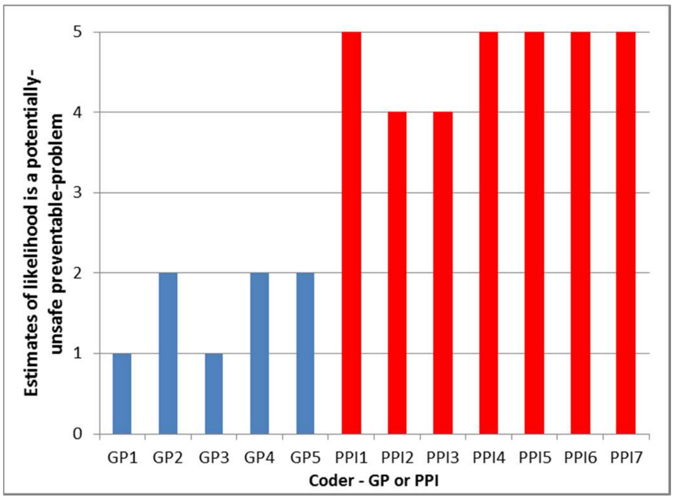

5=very likely or certain, 4=probably, 3=possibly, 2=unlikely, 1=definitely not, 0 = insufficient information or don't know

Patient-reported prospect of harm: suspected your health has been made worse by a problem or error that could have been prevented

Patient-perspective problem-type code: C1.1.2 Started new prescription or changed prescription without sufficient discussion, follow up or checks; D1. Clinician seemed to lack interest in the patient's health problem or did not listen carefully enough

Scenario1/2521. Community mental health

Briefly describe the mistake or problem and how it happened. *"two years delay from GP referral to being able to see psychiatrist at community mental health service. Lack of access meant that he could not be diagnosed with a personality disorder trait in order for medication to be prescribed to treat the problem"*

Could the mistake or problem have been avoided? If so how? "*by referring him back to the previous psychiatrist he was with instead of worrying about boundary changes within the PCTs which are intended to manage caseloads. Basically he was out of catchment, also due to NHS cuts. Also feels these are the result of austerity and people should get social care to help"*

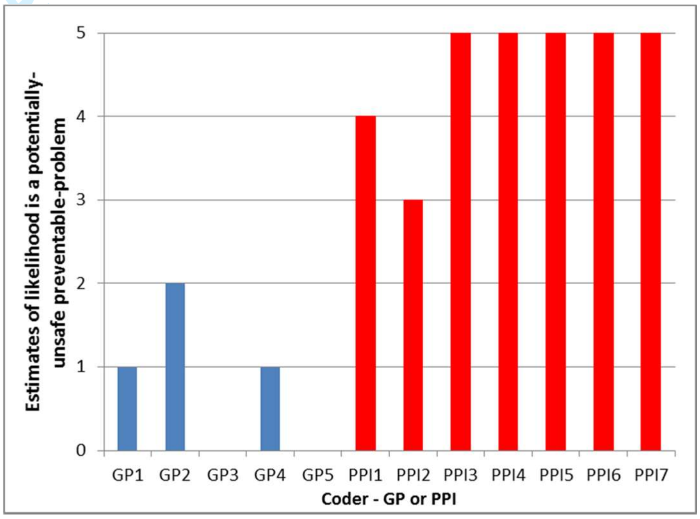

5=very likely or certain, 4=probably, 3=possibly, 2=unlikely, 1=definitely not, 0 = insufficient information or don't know

Were you able to talk about the mistake or problem with anybody working in the primary care service? "*Yes, secretary of mental health psychiatrist he should have seen but waiting for 2 years for*

Patient-reported prospect of harm: your health has been made worse by a problem or error that could have been prevented

Patient-perspective problem-type code: A5. Unable to get an appointment/other problems with making appointment

Scenario2/1148. GP Surgery

Briefly describe the mistake or problem and how it happened. *"I had sore throat and I told the doctor it felt it would go to my chest. He prescribed a throat spray, over 2 days I felt really poorly and ended up in hospital with pneumonia"*

Could the mistake or problem have been avoided? If so how? *"GP should have prescribed antibiotics"*

Were you able to talk about the mistake or problem with anybody working in the primary care service? "No, I was too distressed to discuss the problem or error*"*

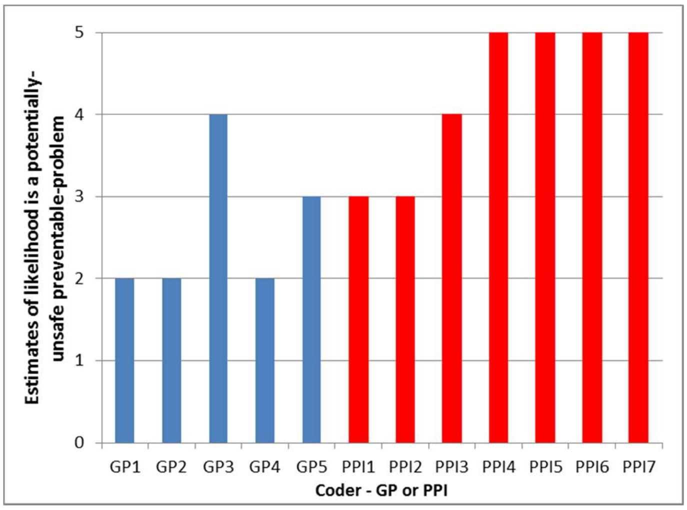

5=very likely or certain, 4=probably, 3=possibly, 2=unlikely, 1=definitely not, 0 = insufficient information or don't know

Patient-reported prospect of harm: your health has been made worse by a problem or error that could have been prevented

Patient-perspective problem-type code: G1.Wrong treatment decision

Scenario2/1188. GP Surgery

Briefly describe the mistake or problem and how it happened. "*Got stomach pain, it was very similar to gall bladder pain but had had that removed before so couldn't be that. At first would have made an appointment with my doctor but none were available for a month. I insisted and found out it was gall bladder stones in bile duct which is serious. Total delay (in pain) 3-4 days"*

Could the mistake or problem have been avoided? If so how? *"Quicker appointment"*

Were you able to talk about the mistake or problem with anybody working in the primary care *"Yes, spoke to doctor about the problem. No apology or changes to the service"*

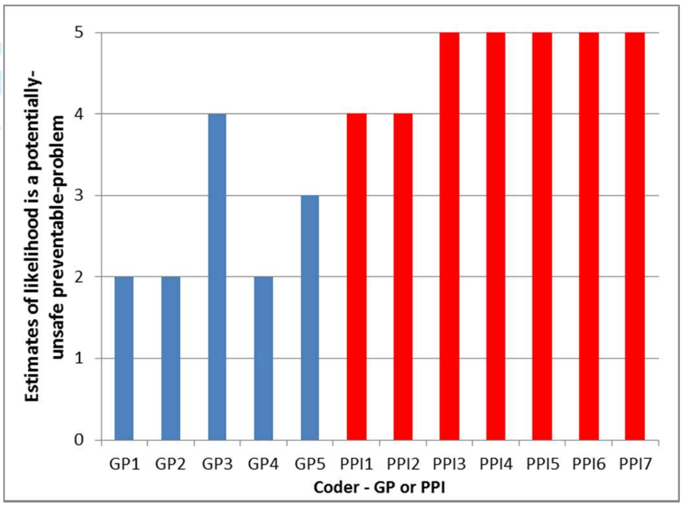

5=very likely or certain, 4=probably, 3=possibly, 2=unlikely, 1=definitely not, 0 = insufficient information or don't know

Patient-reported prospect of harm: health could have been made worse had someone not noticed a problem or error

Patient-perspective problem-type code: A5. Unable to get an appointment/other problems with making appointment

Scenario2/1866. Dental Surgery

Briefly describe the mistake or problem and how it happened. “*Osteonecrosis of the jaw happened due to a tooth being extracted when it should not have been because of medication I was taking”*

Could the mistake or problem have been avoided? If so how? *“More knowledge on the part of the dental profession”*

Were you able to talk about the mistake or problem with anybody working in the primary care service? “*No, there was no point talking about the problem with the primary care service as the situation was beyond that”*

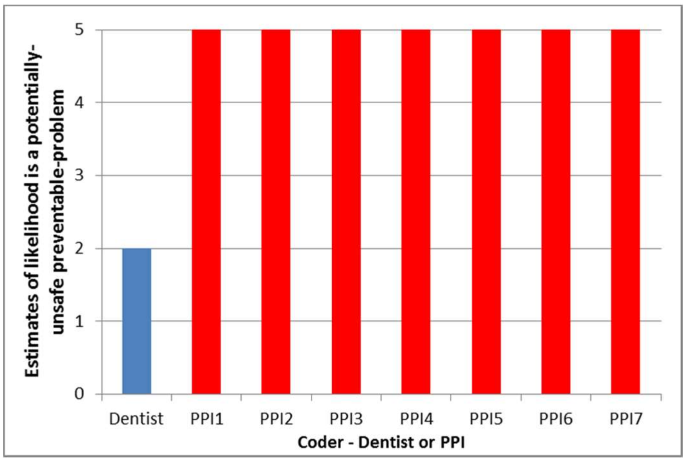

5=very likely or certain, 4=probably, 3=possibly, 2=unlikely, 1=definitely not, 0 = insufficient information or don’t know

Patient-reported prospect of harm: your health has been made worse by a problem or error that could have been prevented

Patient-perspective problem-type code: G1. Wrong treatment decision

Scenario2/3357. Physiotherapy

Briefly describe the mistake or problem and how it happened. *“GP referred to physio for shoulder pain, physio made problem worse and operation was required”*

Could the mistake or problem have been avoided? If so how? *“inexperienced physio made wrong diagnosis”*

Were you able to talk about the mistake or problem with anybody working in the primary care service? “*Yes, GP”*

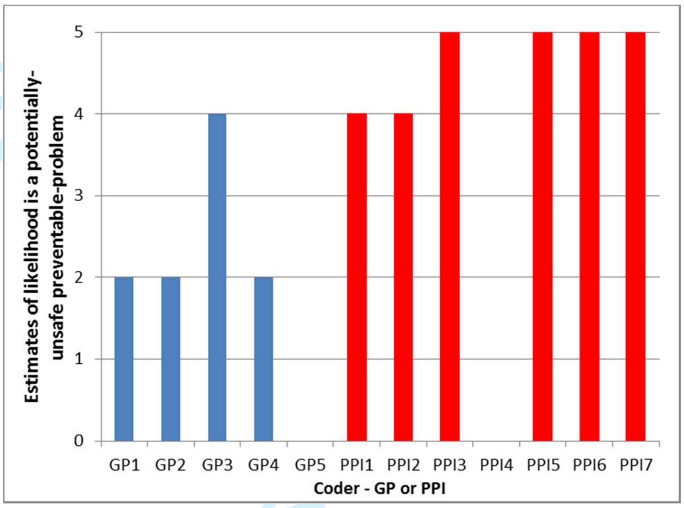

5=very likely or certain, 4=probably, 3=possibly, 2=unlikely, 1=definitely not, 0 = insufficient information or don’t know

Patient-reported prospect of harm: your health has been made worse by a problem or error that could have been prevented

Patient-perspective problem-type code:

F1. Wrong/late/missed/delayed diagnosis; G1. Wrong treatment decision

For peer review only

Scenario2/3359. GP Surgery

Briefly describe the mistake or problem and how it happened. *"Have thyroid problem. GP reduced medication dose without a review and caused health to deteriorate"*

Could the mistake or problem have been avoided? If so how? *"by appropriate blood test taken regularly to monitor my thyroid status"*

Were you able to talk about the mistake or problem with anybody working in the primary care service? *"Yes, GP"*

Patient-reported prospect of harm: suspected your health has been made worse by a problem or error that could have been prevented

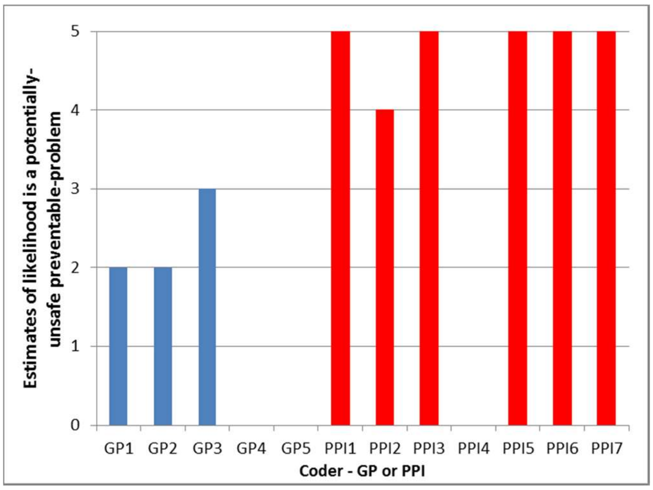

5=very likely or certain, 4=probably, 3=possibly, 2=unlikely, 1=definitely not, 0 = insufficient information or don't know

Patient-perspective problem-type code: B4. Investigation not thorough enough

Scenario1/2451. GP Surgery

Briefly describe the mistake or problem and how it happened. *"review of drugs, GP indicated the high blood pressure, and decided to put me on blood pressure reducing tablets, which resulted in very bad side effects."*

Could the mistake or problem have been avoided? If so how? missing

Were you able to talk about the mistake or problem with anybody working in the primary care service? *"my daughter is GP, she advised me to stop taking the tablets, and monitor my own blood pressure which I did for a week and recorded it."*

Patient-reported prospect of harm: there was a problem or error that could have been prevented but it did not make your health worse

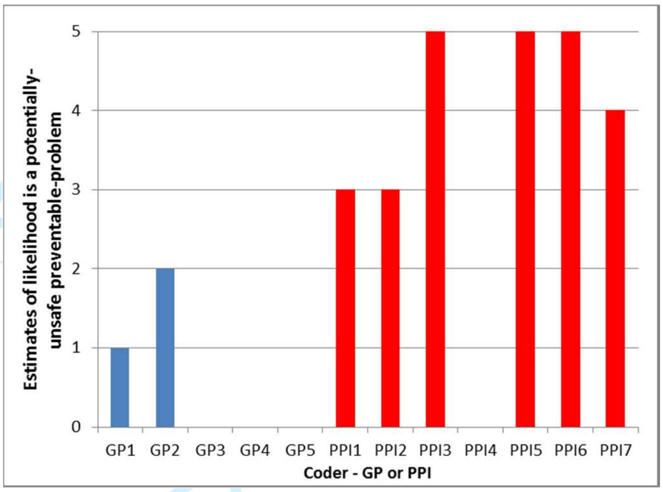

5=very likely or certain, 4=probably, 3=possibly, 2=unlikely, 1=definitely not, 0 = insufficient information or don't know

Patient-perspective problem-type code: C1 Medication error not otherwise specified /other problem

Scenario2/1525. GP Surgery

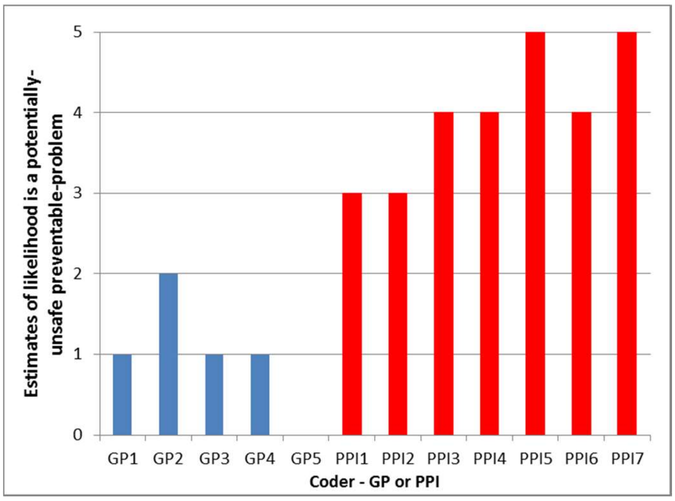

5=very likely or certain, 4=probably, 3=possibly, 2=unlikely, 1=definitely not, 0 = insufficient information or don't know

Briefly describe the mistake or problem and how it happened. *"Complaining about severe pain in right shoulder then left shoulder for 3 years. I demanded to see a specialist. I saw a muscular skeletal specialist who diagnosed me with fibromyalgia, so I am no longer able to go to the gym now."*

Could the mistake or problem have been avoided? If so how? *"If the diagnosis had not have taken as long my overall health and fitness would not have deteriorated. It's affected my mental health and body image and I have paid over 2,000 pounds for private chiropractor"*

Were you able to talk about the mistake or problem with anybody working in the primary care service? *"the musculoskeletal specialist when referred listened to me and gave a diagnosis"*

Patient-reported prospect of harm: your health has been made worse by a problem or error that could have been prevented

Patient-perspective problem-type code: B5. Not referred when patient felt was needed

Patient reported scenarios occurring during the past 12 months that PPIs scored as higher likelihood to be a potentially-unsafe preventable-problem in primary care compared with GPs – pilot survey

Scenario3/179. GP Surgery

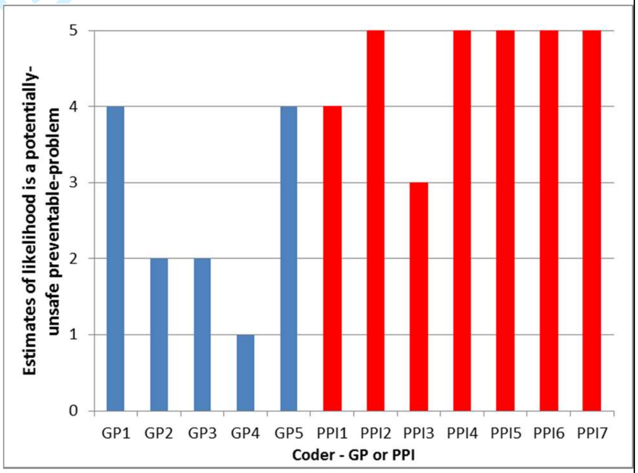

5=very likely or certain, 4=probably, 3=possibly, 2=unlikely, 1=definitely not, 0 = insufficient information or don't know

Briefly describe the mistake or problem and how it happened. *"I had a severe reaction to Atorvastatin after a dose increase so much so that I was almost immobile and took 4 months to recover"*

Could the mistake or problem have been avoided? If so how? *"According to guidelines I should have been on the increased dose - it took a long time to convince the GP that I needed blood tests to find out why I couldn't walk. My GP was very hesitant to admit that I did have a reaction to statins."*

Were you able to talk about the mistake or problem with anybody working in the primary care service? *"No I could not find anybody with whom I could discuss the mistake or problem. It was not really the GPs fault per se, just took a lot of convincing that there was a problem"*

Patient-reported prospect of harm: health could have been made worse had someone not noticed a problem or error

Patient-perspective problem-type code: C1.1.3 Long term or continued prescribing without review or consideration of long term or side effects

Scenario3/285. GP Surgery

Briefly describe the mistake or problem and how it happened. *"Doctor kept saying I had vitamin deficiency B1, it turned out I had peripheral neuropathy which is very painful"*

Could the mistake or problem have been avoided? If so how? *"I just needed the proper medication to help"*

Were you able to talk about the mistake or problem with anybody working in the primary care service? *"Just saw another Doctor and she knew straight away what the problem was - she was experienced with Diabetic problems. Yes had the opportunity but did not feel comfortable to discuss the mistake or problem"*

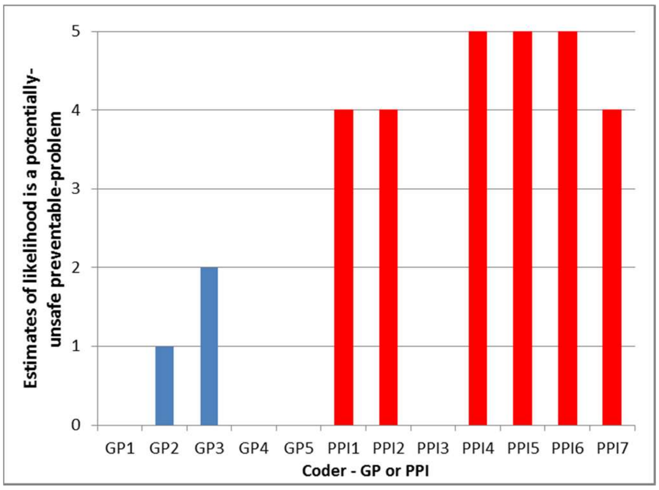

5=very likely or certain, 4=probably, 3=possibly, 2=unlikely, 1=definitely not, 0 = insufficient information or don't know

Patient-reported prospect of harm: prompted via Q10 (Box 1 main paper)

Patient-perspective problem-type code: F1. Wrong/late/missed/delayed diagnosis

Scenario3/347. GP Surgery

Briefly describe the mistake or problem and how it happened. *"Incapable diabetic doctor trying to take blood out the back of my hand haphazardly, not listening and resulting in me fitting and the student watching having to get help."*

Could the mistake or problem have been avoided? If so how? *"Yes. By listening to me"*

Were you able to talk about the mistake or problem with anybody working in the primary care service? *"No I could not find anybody with whom I could discuss the mistake or problem"*

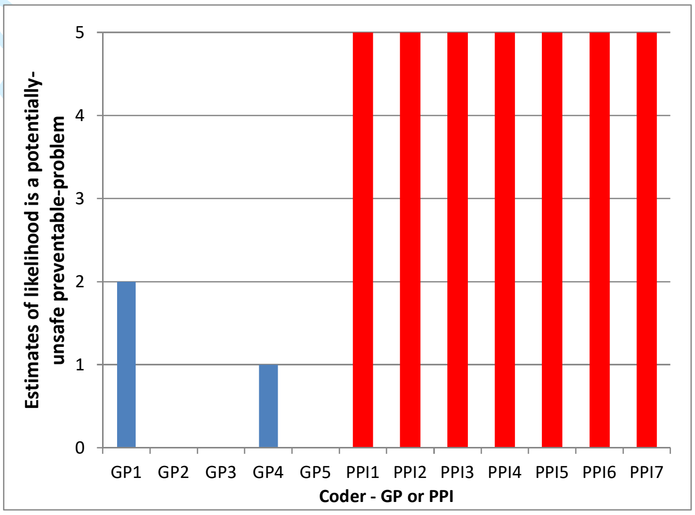

5=very likely or certain, 4=probably, 3=possibly, 2=unlikely, 1=definitely not, 0 = insufficient information or don't know

Patient-reported prospect of harm: prompted via Q10 (Box 1 main paper)

Patient-perspective problem-type code: E2. Procedure was not carried out correctly; D1. Clinician seemed to lack interest in the patient's health problem or did not listen carefully enough

Scenario3/384. Dental Surgery

Briefly describe the mistake or problem and how it happened. *"I had an infection under my wisdom tooth. They agreed that the only way to solve the problem was to take the tooth out. They gave me an appointment to do this in 6 weeks. I am a type 1 diabetic and the infection was affecting my blood sugars and I was concerned that I would have to go to A&E if my blood sugars continued to rise due to the infection. It would have affected my health if I had not paid to go to a private dentist."*

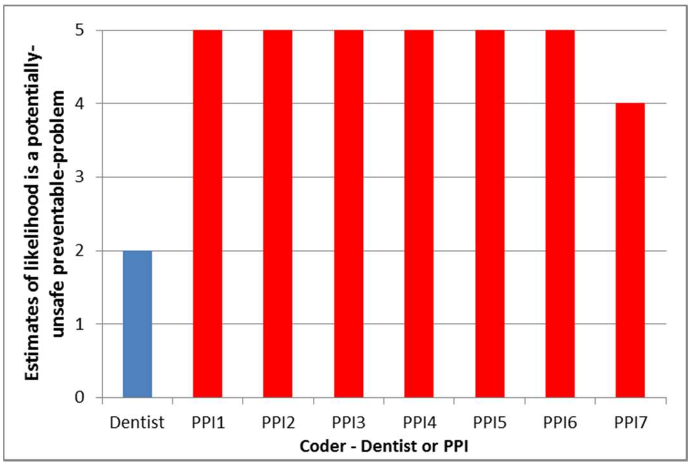

5=very likely or certain, 4=probably, 3=possibly, 2=unlikely, 1=definitely not, 0 = insufficient information or don't know

Could the mistake or problem have been avoided? If so how? *"They could have taken out the tooth straight away. I was happy to wait at the emergency dentist for them to do this."*

Were you able to talk about the mistake or problem with anybody working in the primary care service? *"I explained but they said I would have to wait. They also asked if I needed a sugary drink when I said that my sugars were high so I was too scared to eat and had not eaten in 12hrs. It was clear they didn't understand diabetes."*

Patient-reported prospect of harm: health could have been made worse had someone not noticed a problem or error

Patient-perspective problem-type code: A5. Unable to get an appointment/other problems with making appointment

Scenario3/366. Dental Surgery

Briefly describe the mistake or problem and how it happened. *"Caries, cavities and problem with crown not diagnosed or treated"*

Could the mistake or problem have been avoided? If so how? *"Better dentist & not working to tight time-scale imposed by company owning dental surgery"*

Were you able to talk about the mistake or problem with anybody working in the primary care service? *"No I could not find anybody with whom I could discuss the mistake or problem"*

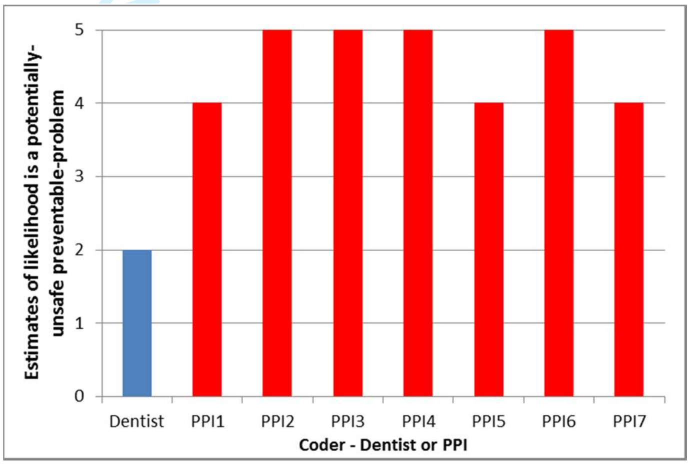

5=very likely or certain, 4=probably, 3=possibly, 2=unlikely, 1=definitely not, 0 = insufficient information or don't know

Patient-reported prospect of harm: prompted via Q10 (Box 1 main paper)

Patient-perspective problem-type code: C3. Problem with dental treatment or diagnosis

For peer review only - http://bmjopen.bmj.com/site/about/guidelines.xhtml

Scenario3/458. GP Surgery

Briefly describe the mistake or problem and how it happened. *"Using the summary on discharge from hospital, one GP transcribed incorrectly on to my electronic notes ie size of ovarian cyst was 7.5cms and he put 7.5 mms. Another GP requested diagnostic bone density scan but either forgot or did not record it and she ended up questioning why I had it and who requested it. She also referred me for an orthopedic consultation then said I was not funded for the steroid injection put into my swollen elbows."*

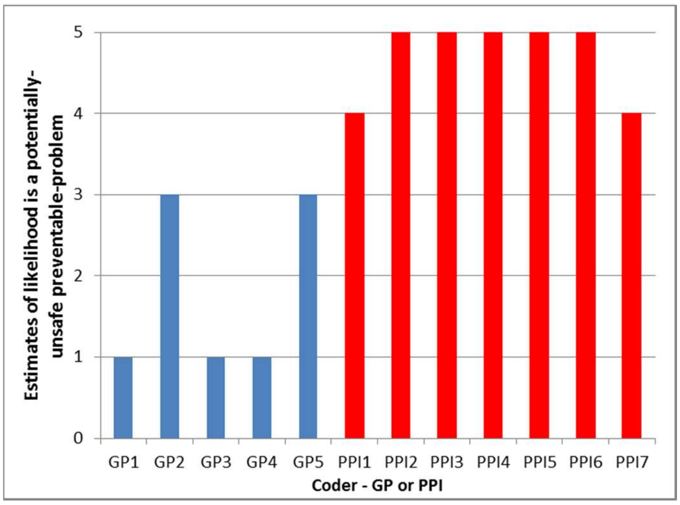

5=very likely or certain, 4=probably, 3=possibly, 2=unlikely, 1=definitely not, 0 = insufficient information or don't know

Could the mistake or problem have been avoided? If so how? *"Yes"*

Were you able to talk about the mistake or problem with anybody working in the primary care service? *"I was too scared to discuss my concerns for fear of being labelled a trouble maker"*

Patient-reported prospect of harm: health could have been made worse had someone not noticed a problem or error

Patient-perspective problem-type code: A2. Incorrect notes/inadequate notes/notes not kept up to date

Scenario3/484. GP Surgery

Briefly describe the mistake or problem and how it happened. *"GP prescribed pills, but then got phone call saying not to take them"*

Could the mistake or problem have been avoided? If so how? *"Not sure"*

Were you able to talk about the mistake or problem with anybody working in the primary care service? *"No I was not concerned about the problem"*

Patient-reported prospect of harm: prompted via Q10 (Box 1 main paper)

Patient-perspective problem-type code: C1. Medication problem

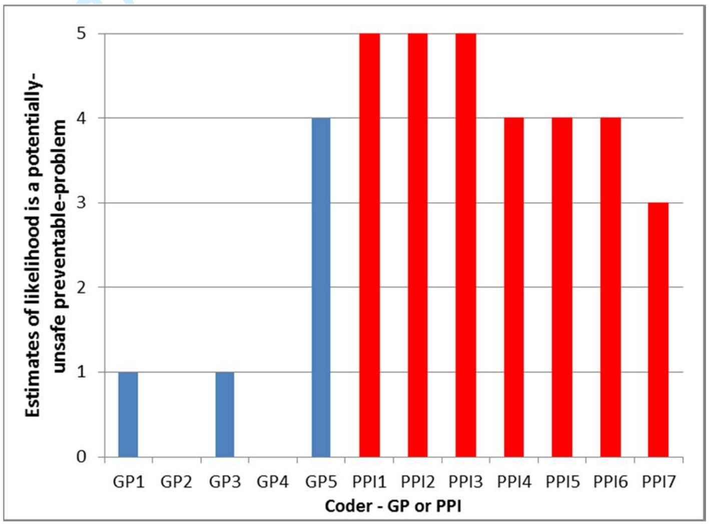

5=very likely or certain, 4=probably, 3=possibly, 2=unlikely, 1=definitely not, 0 = insufficient information or don't know

Scenario3/555. GP Surgery

Briefly describe the mistake or problem and how it happened. *"I had a burst appendix and peritonitis, something that even a scan couldn't detect adequately. My first visit to GP was when I said I think I have appendicitis, no other symptoms only the pain. It was ten days before seeing a consultant, a further 10 days to have a scan, then 2 weeks to be told that I had a lump on my colon which is what my GP had said 5 weeks previously. It was a further 2 weeks before I had surgery."*

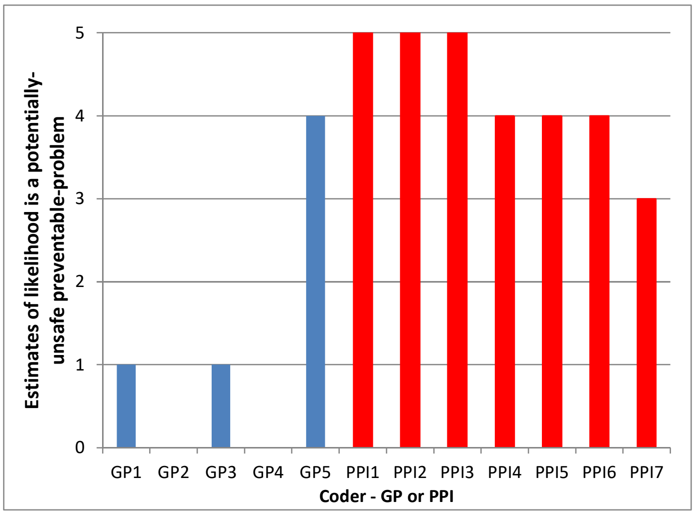

5=very likely or certain, 4=probably, 3=possibly, 2=unlikely, 1=definitely not, 0 = insufficient information or don't know

Could the mistake or problem have been avoided? If so how? *"If my GP had referred me for a scan immediately it would have saved 3 weeks out of the seven. It was two weeks from scan to results and I hear that is usual, but they're not looking at them for 2 weeks"*

Were you able to talk about the mistake or problem with anybody working in the primary care service? *"Had the outcome been different my widow might have pursued the matter further. The system is at fault rather than any individual."*

Patient-reported prospect of harm: your health has been made worse by a problem or error that could have been prevented

Patient-perspective problem-type code: B5. Not referred when patient felt was needed

For peer review only

STROBE Statement—Checklist of items that should be included in reports of ***cross-sectional studies***

	Item No	Recommendation
**Title and abstract**	1	(*a*) Indicate the study's design with a commonly used term in the title or the abstract Yes p1
		(*b*) Provide in the abstract an informative and balanced summary of what was done and what was found yes p3
**Introduction**		
Background/rationale	2	Explain the scientific background and rationale for the investigation being reported Yes p4
Objectives	3	State specific objectives, including any prespecified hypotheses yes p4-5
**Methods**		
Study design	4	Present key elements of study design early in the paper yes p5
Setting	5	Describe the setting, locations, and relevant dates, including periods of recruitment, exposure, follow-up, and data collection yes p5
Participants	6	(*a*) Give the eligibility criteria, and the sources and methods of selection of participants yes p5
Variables	7	Clearly define all outcomes, exposures, predictors, potential confounders, and effect modifiers. Give diagnostic criteria, if applicable yes box1, online appendix 1
Data sources/ measurement	8*	For each variable of interest, give sources of data and details of methods of assessment (measurement). Describe comparability of assessment methods if there is more than one group yes p5, online appendix 1
Bias	9	Describe any efforts to address potential sources of bias yes p5 and reference 23
Study size	10	Explain how the study size was arrived at n/a power calculation described in protocol in terms of confidence intervals for generalisability to UK population but sample size was determined for practical reasons as is a descriptive analysis.
Quantitative variables	11	Explain how quantitative variables were handled in the analyses. If applicable, describe which groupings were chosen and why yes p6-7
Statistical methods	12	(*a*) Describe all statistical methods, including those used to control for confounding yes p5
		(*b*) Describe any methods used to examine subgroups and interactions, yes just chi2 tests p5
		(*c*) Explain how missing data were addressed all missing data is listed in the tables so it is completely transparent how this was dealt with, there were few missing data
		(*d*) If applicable, describe analytical methods taking account of sampling strategy the unweighted sample was used. This is not discussed as the difference was very small and adds much complexity without adding important information.
		(*e*) Describe any sensitivity analyses none done
**Results**		
Participants	13*	(a) Report numbers of individuals at each stage of study—eg numbers potentially eligible, examined for eligibility, confirmed eligible, included in the study, completing follow-up, and analysed yes online appendix 3
		(b) Give reasons for non-participation at each stage yes online appendix 3
		(c) Consider use of a flow diagram yes online appendix 3
Descriptive data	14*	(a) Give characteristics of study participants (eg demographic, clinical, social) and information on exposures and potential confounders yes table 1
		(b) Indicate number of participants with missing data for each variable of interest yes

For peer review only

		all tables
Outcome data	15*	Report numbers of outcome events or summary measures yes all tables
Main results	16	(*a*) Give unadjusted estimates and, if applicable, confounder-adjusted estimates and their precision (eg, 95% confidence interval). Make clear which confounders were adjusted for and why they were included yes table 3
		(*b*) Report category boundaries when continuous variables were categorized yes all tables
		(*c*) If relevant, consider translating estimates of relative risk into absolute risk for a meaningful time period yes p9
Other analyses	17	Report other analyses done—eg analyses of subgroups and interactions, and sensitivity analyses table 6 considers demographics for problems more likely to be a potentially harmful.
**Discussion**		
Key results	18	Summarise key results with reference to study objectives yes p9
Limitations	19	Discuss limitations of the study, taking into account sources of potential bias or imprecision. Discuss both direction and magnitude of any potential bias yes p11
Interpretation	20	Give a cautious overall interpretation of results considering objectives, limitations, multiplicity of analyses, results from similar studies, and other relevant evidence yes p11-12
Generalisability	21	Discuss the generalisability (external validity) of the study results yes p10
**Other information**		
Funding	22	Give the source of funding and the role of the funders for the present study and, if applicable, for the original study on which the present article is based yes p13

*Give information separately for exposed and unexposed groups.

Note: An Explanation and Elaboration article discusses each checklist item and gives methodological background and published examples of transparent reporting. The STROBE checklist is best used in conjunction with this article (freely available on the Web sites of PLoS Medicine at http://www.plosmedicine.org/, Annals of Internal Medicine at http://www.annals.org/, and Epidemiology at http://www.epidem.com/). Information on the STROBE Initiative is available at www.strobe-statement.org.

For peer review only

For peer review only - http://bmjopen.bmj.com/site/about/guidelines.xhtml

BMJ Open

The frequency and nature of potentially-harmful preventable-problems in primary care from the patient's perspective with clinician review – a population level survey in Great Britain

Journal:	*BMJ Open*
Manuscript ID	bmjopen-2017-020952.R1
Article Type:	Research
Date Submitted by the Author:	26-Mar-2018
Complete List of Authors:	Stocks, Susan; University of Manchester, GM Patient Safety Translational Research Centre<br>Donnelly, Ailsa; Research User Group (RUG) of the NIHR Greater Manchester Primary Care Patient Safety Translational Research Centre<br>Esmail, Aneez; University of Manchester, Primary Care Research Group<br>Beresford, Joanne; Research User Group (RUG) of the NIHR Greater Manchester Primary Care Patient Safety Translational Research Centre<br>Luty, Sarah; NHS Greater Glasgow and Clyde<br>Deacon, Richard; St Gabriels Medical Centre<br>Danczak, Avril; University of Manchester, Central and South Manchester Specialty Training Programme for General Practice<br>Mann, Nicola; Research User Group (RUG) of the NIHR Greater Manchester Primary Care Patient Safety Translational Research Centre<br>Townsend, David; Research User Group (RUG) of the NIHR Greater Manchester Primary Care Patient Safety Translational Research Centre<br>Ashley, James; University of Manchester, Division of Dentistry<br>Gamble, Carolyn; Research User Group (RUG) of the NIHR Greater Manchester Primary Care Patient Safety Translational Research Centre<br>Bowie, Paul; GP Section, NHS Education for Scotland<br>Campbell, Stephen; University of Manchester, Centre for Primary Care
**Primary Subject Heading**:	General practice / Family practice
Secondary Subject Heading:	Health services research
Keywords:	PRIMARY CARE, Quality in health care < HEALTH SERVICES ADMINISTRATION & MANAGEMENT, SOCIAL MEDICINE

SCHOLARONE™
Manuscripts

The frequency and nature of potentially-harmful preventable-problems in primary care from the patient's perspective with clinician review – a population level survey in Great Britain

S Jill Stocks *research fellow*[1], Ailsa Donnelly *public and patient involvement partner*[2], Aneez Esmail *professor*[3], Joanne Beresford *public and patient involvement partner*[2], Sarah Luty *general practitioner*[4], Richard Deacon *general practitioner*[5], Avril Danczak *general practitioner*[6], Nicola Mann *general practitioner*[2], David Townsend *general practitioner*[2], James Ashley *general dental practitioner*[7], Carolyn Gamble *public and patient involvement partner*[2], Paul Bowie *programme director*[8,9], Stephen M Campbell *professor*[3]

1.Division of Population Health, Health Services Research and Primary Care, University of Manchester, Manchester M13 9PL, UK

2. Research User Group (RUG) of the NIHR Greater Manchester Primary Care Patient Safety Translational Research Centre, Centre for Primary Care, Division of Population Health, Health Services Research and Primary Care, University of Manchester, Manchester M13 9PL, UK

3. NIHR Greater Manchester Primary Care Patient Safety Translational Research Centre, Centre for Primary Care, Division of Population Health, Health Services Research and Primary Care, University of Manchester, Manchester M13 9PL, UK

4. General Practitioner NHS Greater Glasgow and Clyde, Medical Directorate, NHS Education for Scotland, Glasgow G3 8BW

5. St Gabriels Medical Centre, 4, Bishops Road, Prestwich, Manchester M25 0HT, UK

6. Central and South Manchester Specialty Training Programme for General Practice, Health Education England North West (HEENWE) Education and Research Centre, Wythenshawe Hospital, Manchester M23 9LT, UK

7. Woodlands Dental Practice, Rock Ferry, Wirral, CH42 4NG, UK

8. Medical Directorate, NHS Education for Scotland, 2 Central Quay, Glasgow G3 8BW, UK.

9. Institute of Health and Wellbeing, University of Glasgow, Glasgow G12 0XH, UK

Correspondence to Jill Stocks, Division of Population Health, Health Services Research and Primary Care, University of Manchester, Manchester M13 9PL, UK
Email: sjstocks@btinternet.com
Tel: 01612752375
Fax: 0161 275 7600

Word count: 4701

For peer review only

For peer review only - http://bmjopen.bmj.com/site/about/guidelines.xhtml

Abstract

Objectives: To estimate the frequency of patient-perceived potentially-harmful problems occurring in primary care. To describe the type of problem, patient predictors of perceiving a problem, the primary care service involved, how the problem was discussed and patient suggestions as to how the problem might have been prevented. To describe clinician/public opinions regarding the likelihood that the patient-described scenario is potentially-harmful.

Design: population level survey

Setting: Great Britain

Participants: A nationally representative sample of 3975 members of the public aged 15 years or older interviewed during April 2016

Main outcome measures: counts of patient-perceived potentially-harmful problems in the last 12 months, descriptions of patient-described scenarios and review by clinicians/members of the public

Results:

3975 of 3996 participants in a nationally-representative survey completed the relevant questions (99.5%). 300 (7.6%; 95% confidence intervals 6.7% to 8.4%) of respondents reported experiencing a potentially-harmful preventable-problem in primary care during the past 12 months and 145 (48%) discussed their concerns within primary care. This did not vary with age, gender or type of service used. A substantial minority (30%) of the patient-perceived problems occurred outside general practice, particularly the dental surgery, walk in clinic, out of hours care and pharmacy. Patients perceiving a potentially-harmful preventable-problem were 8 times more likely to have "no confidence and trust in primary care" compared with "yes, definitely" (odds ratio 7.9; 5.9 to 10.7) but those who discussed their perceived-problem appeared to maintain higher trust and confidence. Generally clinicians ranked the patient-described scenarios as unlikely to be potentially harmful.

Conclusions: this study highlights the importance of actively soliciting patient's views about preventable harm in primary care as patients frequently perceive potentially-harmful preventable-problems and make useful suggestions for their prevention. Such engagement may also help to improve confidence and trust in primary care.

Strengths and limitations of this study

- This is the first quantitative, population level, patient designed study examining patient-perceived potentially harmful problems in primary care purely from the patient perspective.
- The 3975 respondents were demographically similar to the British population and had a similar level of trust in their GP as measured in the English GP Patient survey.

For peer review only - http://bmjopen.bmj.com/site/about/guidelines.xhtml

- Respondents were initially encouraged to express their own views on what constitutes potentially-harmful preventable-problem through the use of a non-leading screening question.

- Primary care clinicians and members of the public estimated the likelihood that, in their opinion, each patient-described scenario was a potentially-harmful preventable-problem.

For peer review only - http://bmjopen.bmj.com/site/about/guidelines.xhtml

Background

Patients and clinicians view safety differently; patients tend to consider both serious safety problems as well as lesser causes of distress as safety concerns.(1) Patients judge quality and safety of care in terms of the ongoing care they receive over time whereas healthcare professionals may take the view that they provide high quality healthcare occasionally punctuated by discrete safety incidents and adverse events.(2) Even so patients can report medical errors accurately (3, 4) but they may have different priorities to professionals *e.g.* prioritising psychological and emotional harm over technical errors.(5) Given these differences the patient's approach to preventing safety problems may differ from clinicians, particularly if they believe clinicians to be responsible for the problem rather than the institutional system.(6, 7) Patient safety in primary care is rarely evaluated from the patient's perspective (8) whereas involving patients in identifying errors and reducing harm is common in secondary care.(3,9-11) A more participatory role for patients is advocated as a way to improve safety (12) suggesting a need for patients and professionals to be cognisant of each other's expectations and understanding of safety.

Estimates of the frequency of patient safety problems in primary care are generally from the clinician's perspective and range from less than 1 to 24 per 100 consultations or record review.(13-15) Some studies have quantified patient safety problems in primary care from the patient's perspective (6, 7, 16-18) However, quantitative patient-reported data from the UK is sparse; this may be partly due to the lack of a valid and reliable instrument for measuring safety in primary care from the patient's perspective.(19) The National Reporting and Learning System (NRLS) in England and Wales is a voluntary reporting scheme for NHS staff to report patient safety incidents. Less than 1% of reports originate from primary care (20), probably reflecting under-reporting. Until recently patients could not make reports directly to the NRLS. (21, 22) A European survey in 2013 found that 43% of UK respondents felt that it was "likely" that patients could be harmed by non-hospital healthcare and a recent survey of the UK public found that 21% of respondents reported experiencing a potentially-harmful preventable-problem in primary care within the past 12 months. (23, 24) These surveys suggest large differences between patients and clinicians in their beliefs about potentially-harmful problems in primary care, but this has not been examined at the population level. The PREOS-PC questionnaire has reported qualitatively on patient perceptions of safety in English general practices finding that patient recommendations for safer health care included improvements in patient- centred communication, continuity of care, timely appointments, technical quality of care, active monitoring, teamwork, health records and practice environment.(25, 26)

We aimed to quantify and describe patient-perceived potentially-harmful preventable-problems occurring in UK primary care. We also wanted to explore the differences in opinion between primary care professionals and the public regarding the potential for harm in the patient-described scenarios. Our approach aimed to capture the true patient perspective through extensive public and patient involvement (PPI); the study was conceived, co-designed and implemented by a team of three members of the public and one researcher.(24) The primary aims of the study were to estimate the annual and three year frequency of patient-reported potentially-harmful preventable-problems occurring in primary care as described by patients and describe the type of problem. The secondary aims were to identify patient predictors of reporting a problem (*e.g.* age, gender, social

For peer review only - http://bmjopen.bmj.com/site/about/guidelines.xhtml

class, income, employment status, ethnicity, to describe the primary care service involved), how the problem was discussed (if it was), patient suggestions as to how it might have been prevented and the variation in opinion between the reporting patient, other members of the public and clinicians in their opinion as to the likelihood the patient-described scenario is a potentially-harmful preventable-problem.

Methods

The population level survey

A survey asking about potentially-harmful preventable-problems occurring in primary care has been designed and piloted with extensive PPI as described in detail elsewhere. (24) The questions from this survey (Box 1, online Appendix 1) were embedded in to the Ipsos MORI GB Face to Face Omnibus (f2f Omnibus, a weekly survey that is used to track British attitudes to issues facing the country). It was used to survey a nationally and regionally representative sample of 4000 adults aged 15 or over living in private households in Great Britain between 8th and 21st April 2016 using a random sampling design described elsewhere.(27) Briefly 170-180 geographically representative sampling points were randomly selected and interviewers were required to get the interviews from a small group of streets reflecting that sampling point. (Typically an interviewer would get a completed interview from 1 in every 10 to 12 addresses.) The sample size was loosely based on the pilot study (24) which had found that 132/638 (21%) of self-selected respondents had perceived a potentially-harmful preventable-problem (although we anticipated a lower proportion when sampling from the general population). The f2f Omnibus consists of interviews in the participant's home using computer assisted personal interviewing, participation is completely voluntary and there are no incentives to take part. Respondents are free to refuse to answer any questions. The first question (Q1 Box 1) was taken from the English GP patient survey in order to compare the overall level of confidence and trust in their GP among the survey respondents with the larger sample used in the English GP patient survey.(28) The second question (Q2 Box 1) is the main screening question, those responding negatively to Q2 (*i.e.* not experienced a preventable-problem) were directed to a more specific question with a list of commonly understood patient safety events (Q10 Box 1 & online Appendix 1). If this prompted recognition of experiencing a potentially-harmful preventable-problem they were returned to Q4 (Box1). The intention of using a non-leading screening question was to encourage respondents to express their own perspective on what constitutes potentially-harmful preventable-problem rather than being directed towards existing definitions.

Coding of patient-reported scenarios

The nature of the problem described by the patient was coded at face value *i.e.* as the patient described without further interpretation, by one author (SJS) and checked by a second author (JA for dental scenarios, PB for all other scenarios) using a taxonomy developed during the pilot study that also mapped on to a previously published taxonomy for errors in general practice (24, 29, 30) (Table A, online Appendix 1). The medication-related scenarios were coded to a finer level (Table B, online Appendix 1).

Likelihood the scenario described a potentially-harmful preventable-problem

Five GPs, one general dental practitioner and 7 members of the public estimated the likelihood that, in their opinion, each patient-described scenario was a potentially-harmful preventable-problem.(24) The dental scenarios were only rated by the general dental practitioner and members

For peer review only - http://bmjopen.bmj.com/site/about/guidelines.xhtml

of the public. The raters were given the responses to Q2 and Q4 to Q9 (Box1) without any demographic information and asked to score each scenario on a 5 point scale from “very likely or certain” to “definitely not” a potentially-harmful preventable-problem. The scores were used to categorise the scenarios in to two groups according to the public or clinician-estimated likelihoods that they were a potentially-harmful preventable-problem as below. This is described in detail in Table C in online Appendix 1 and individual coding is shown in online Appendix 2.

- Group 1: patient-described scenarios with higher threshold as to likelihood of potential harm; Median score of “very likely or certain” or “probably” or at least one person gave a score of “very likely or certain”
- Group 2: patient-described scenarios with lower threshold as to likelihood of potential harm; Median score of “possibly” or at least one person gave a score of “probably” or higher
- All other scenarios – Median score below 3 (“possibly”) and zero scores above 3 (“possibly”)

The median scores excluded responses where the raters scored “don’t know” or “insufficient information”. We combined all the patient-described scenarios occurring in the last 3 years with scenarios from the pilot study (24) occurring in the last 12 months. We judged this acceptable since we were using the scenarios to compare the views of the clinicians and members of the public without making any inference to the wider population.

Statistical analysis

The 95% confidence intervals for the population means were calculated assuming a normal distribution for the sample mean. Simple cross tabulations were used to describe the data and a binary logistic regression model was used to explore whether particular types of patient (*e.g.* according to their demographics or surveyed opinions) were more likely to perceive a potentially-harmful preventable-problems and what type of scenario was more likely to be ranked as potentially harmful by clinicians and members of the public. Comparisons between demographics and outcomes for the respondents and the UK population were made using a χ^2 test. Inter-rater agreement for the ranking of the patient-described scenarios by clinicians and members of the public was assessed using a two-way random effects model single-measures intraclass correlation coefficient (ICC).(31). All analyses were done using Stata 14.

Public and Patient Involvement

PPI was central to this co-designed survey and was provided through the GMPSTRC RUG and other PPI networks (24). The study was conceived, designed, implemented and analysed by a team of three members of the public (AD, CG, JB) and one researcher (SJS). The piloting of the survey was through existing PPI networks (24). The scoring of the questions as to the likelihood they described a potentially-harmful preventable-problem was undertaken by 7 members of the public, 2 of whom had no previous experience in PPI. These findings will be disseminated to all the PPI groups that contributed to the pilot study and the authors will forward these results to their personal contacts who contributed to the questionnaire design.

Results

For peer review only - http://bmjopen.bmj.com/site/about/guidelines.xhtml

Of 3996 members of the public participating in the f2f Omnibus, 3984 (99.7%) agreed to complete the questions relevant to this study and 3975 (99.5%) actually completed all the questions. Survey responders were broadly representative of the GB population but were significantly more likely to have confidence and trust in the GP seen at their last appointment than the English population (Table D, online Appendix 1) although there was no significant difference when the graded responses "yes definitely" or "yes to some extent" were combined (91% vs 92%, P(χ^2)=0.2).

The progress of the respondents through the analysis is summarised in Figures A & B in online Appendix 1. In total 300 (7.6%) of respondents reported experiencing a potentially-harmful preventable-problem during the past 12 months; of these 193 (4.9%) arose directly from the screening question (Q2 Box1) and 107 (2.7%) were prompted by a list of potentially-harmful preventable-problems (Q10 Box 1, Appendix 1). Of the 193 unprompted problems (Q2 Box 1), 119 (3.0%) patients suspected, or actually believed, that their health had been made worse as a result of the problem whereas 74 (1.9%) believed that they had either noticed the problem before it had any consequences or it had had no effect on their health. A further 132 potentially-harmful preventable-problems were reported as occurring within the past 1 to 3 years (Fig A, Appendix 1) making a 3 year total of 325 (8.2%) arising only from the screening question (Q2 Box1) as there was no prompt question asking about problems over 12 months ago. The combination of an open-ended question (Q2, Box 1) and prompt question (Q10, Box 1) prioritised sensitivity over specificity (as intended) given that 21% of the reported problems (79/379) were excluded from being a potentially-harmful preventable-problem in primary care by the respondent themselves by their response to questions 4 and 6 (*i.e.* not preventable or not in primary care, Box1).

Of the 300 patient-described scenarios occurring within the last 12 months, 93 (31%) were not ranked by any of the 6 clinicians mostly due to insufficient information (in the clinician's opinion). Of the 207 that were ranked by at least one clinician, 24 (11.6%, Table E, online Appendix 1) were considered to "at least probably" describe a potentially-harmful preventable-problem by clinicians (group 1 above). Group 2 (defined above) included 97 (46.9%) scenarios considered to "at least possibly" describe a potentially-harmful preventable-problem by clinicians. The members of the public ranked 116 (39%) scenarios occurring in the last 12 months as "at least probably" a potentially-harmful preventable-problem (group 1) and this included all 97 scenarios ranked as "at least possibly" by clinicians (group 2).

The proportion of respondents reporting a potentially-harmful preventable-problem within the last 12 months by respondent characteristics and unadjusted and adjusted odds ratios estimated by logistic regression are shown in Table 1. Those responding "no, not at all" to the question about trust and confidence in the GP (Q1 Box) were around eight times more likely to report a problem compared to those responding "yes, definitely"(Table 1). Women and rural dwellers were significantly more likely to report experiencing a potentially-harmful preventable-problem even when only including the scenarios judged to be more likely to be potentially-harmful by clinicians (Table 1). People not in employment due to a disability, self-employed or with one or more children were more likely to report a problem but not when only those scenarios judged to be more likely to be potentially-harmful by clinicians were included (Table 1).

Characteristics of the patient-reported scenarios

For peer review only - http://bmjopen.bmj.com/site/about/guidelines.xhtml

The types of problem occurring in the last 12 months alongside their clinician rankings are summarised in Figure 1. Generally respondents were equally likely to describe the nature of the problem as related to healthcare delivery, investigation, treatment (mainly medication), communication or lack of clinical knowledge or skills (Panel B Fig 1). Within the medication problems the most common scenarios were being prescribed a wrong, contra-indicated or inappropriate drug or the wrong dose or delivery method (Panel C Fig 1). The respondents did not identify any previously unreported types of problem and the patient-reported scenarios mapped well on to an established taxonomy of errors in primary care (Fig 1). However the prompt question (Q10) particularly increased reports of scenarios related to appointments, referrals and reporting of test results suggesting that the respondents did not consider these to be potentially harmful problems in the first instance (Fig F, online Appendix 1). Table 2 provides information about the patient's response to the potentially-harmful preventable-problem and the primary care service involved. A substantial minority (30%) of problems occurred outside general practice, particularly the dental surgery, walk in clinic, out of hours care and pharmacy. Around half of the patients had discussed their problem with a primary care professional and usually this was a person who worked in the same organisation as where their problem had occurred (Table 2). There were no significant differences between patients who discussed the problem, and those who did not, according to gender (males 49% vs females 51%, $P\chi^2$=0.78), age (38% to 62% in 10 year age bands, $P\chi^2$=0.33), type of service being used (general practice 50% vs other services 50%, $P\chi^2$=0.95), working as a healthcare professional (no 56% vs yes 50% $P\chi^2$=0.44) or describing a problem ranked higher by clinicians (below lower threshold 50% vs above lower threshold 50%, $P\chi^2$=0.98). Those reporting a problem in the first instance at Q2 (Box 1) without prompting were somewhat more likely to have discussed the problem (unprompted 53% vs prompted 43%, $P\chi^2$=0.08) whereas ethnic minorities were somewhat less likely to have discussed the problem (white 51% vs other ethnicity 37%, $P\chi^2$=0.09). Patients who discussed their problem were significantly more likely to "definitely" have trust and confidence in their GP (Q1 Box 1; 61% did discuss their problem vs 39% who did not discuss their problem, $P\chi^2$<0.001). The reasons given for not discussing the problem varied but the most common reasons related to feeling uncomfortable about discussing the problem, being too distressed or ill, being unable to find the appropriate person with whom to discuss the problem or the respondent was unconcerned about the problem. The respondent's suggestions as to how the problem might have been prevented are summarised in Table 3. The most frequent suggestions revolved around quicker access to primary care and investigations and a more participatory role. They rarely identified a particular individual as the problem or made specific suggestions for improvement strategies.

Comparison of the opinions of clinicians and members of the public about the patient-reported scenarios

The total number of patient-described scenarios available for analysis was 564 (432 from the main survey last 3 years and 132 from the pilot survey in last 12 months) but only 406 (72%) patients provided sufficient information for at least one clinician to score the scenario on a 5 point scale as to the likelihood that the patient described a potentially-harmful preventable problem (Table C in online Appendix 1). The members of the public scored 426 (76%) of the scenarios. The median scores for each patient-described scenario are shown in Fig 2. Members of the public were significantly more likely to designate the patient-described scenarios as potentially-harmful preventable-problems compared with clinicians (median clinician score of 2.5, "unlikely- possibly" compared with members of the public score of 3.5, "possibly-probably"; Wilcoxon signed-rank test

For peer review only - http://bmjopen.bmj.com/site/about/guidelines.xhtml

z=16.4, P<0.001). From the clinician perspective just 8% of the problems occurring during the past 12 months were categorised as “probably to almost certainly” potentially harmful whereas for the members of the public the corresponding proportion was 39% (Table E in online Appendix 1 using the higher threshold). The individual patient-described scenarios scored by clinicians as more likely to be a potentially-harmful preventable-problems (median score is higher than “possibly” and scored by at least 2 clinicians, or one clinician scored “very likely or certain”) and the scenarios with the greatest disagreement between members of the public and clinicians (median scores differ by 3 points or more on a 5 point scale) are summarised in online Appendix 2. The single measures ICC for absolute measures was 0.43 (0.38 to 0.49) for the members of the public and 0.23 (0.09 to 0.40) for clinicians, illustrating that members of the public had somewhat better agreement than clinicians. The associations between the characteristics of the patient or problem, and the clinician rankings of the likelihood it is a potentially-harmful preventable-problem are shown in Table F, online Appendix 1. Clinicians were more likely to rank scenarios as “possibly to almost certainly” potentially-harmful if they related to treatment, diagnosis or the patient was qualified as a healthcare professional (even though they were blind to this information) but for the members of the public scenarios related to treatment, investigation, clinical skills, diagnosis or where the patient had reported a problem in the first instance without prompting. Additionally members of the public were more likely to rank problems reported through the pilot survey as potentially harmful. The diagnoses (as specified by the patient) more likely to be considered a potentially-harmful preventable-problem by both clinicians and members of the public were cancer and cardiovascular problems.

Discussion

Our main finding is that 7.6% of respondents in a GB nationally representative survey of 3975 people reported experiencing a potentially-harmful preventable-problem in primary care during the past 12 months. This is important, not only because patients may be experiencing genuine safety problems, but also because respondents perceiving a potentially-harmful preventable-problem were found to be eight times less likely to have confidence and trust in their GP (Table 1). Furthermore only around half of these patients perceiving a problem discussed their concern with a primary care professional. The implication is that many patient-perceived problems remain unknown to clinicians - scaling our results up to the GB adult population implies that around 3 million patients (3.8 million; 95% confidence intervals 3.3 million to 4.2 million) believe that they have experienced a potentially-harmful preventable-problem during the past 12 months and 1.5 million (1.2 million to 1.8 million) believe or suspect that their health has been made worse as a result. Clearly clinicians need to be aware of these patient-perceived preventable-problems where there is the potential for harm, but our findings also suggest that discussing such problems with the patient may also help to maintain confidence and trust in primary care among those who perceived a problem. (As this is a cross sectional study we cannot know whether the patients who discussed their problem did so because they already had a higher level of confidence and trust in their GP or discussing the problem contributed to the higher level of confidence and trust.) An accessible, informal route to actively engage and solicit patient’s concerns about primary care may be helpful particularly given that the most common reasons patients gave for not discussing their problems are modifiable *e.g.* being unable to find the appropriate person or feeling uncomfortable about raising their concern and some were worried about the implications of doing so for their future care. Furthermore improving communication and patient involvement was one of the most frequently suggested strategies for preventing the potentially-harmful preventable-problem (alongside quicker access to primary care

For peer review only - http://bmjopen.bmj.com/site/about/guidelines.xhtml

and investigations). Other work suggested that patients are likely to blame individual clinicians for their perceived problem (7) but we did not particularly find this.

Our finding that around 30% of patient-perceived problems in primary care occurred outside general practice emphasizes the need for research in other areas of primary care, for example, 9% of the patient-perceived potentially-harmful preventable problems in the last 12 month occurred in dentistry in primary care (corresponding GB estimate 0.34 million; 0.21 million to 0.47 million) yet safety in this area remains largely unexplored.(32, 33)

Other studies have found differences between patients in perceiving mistakes or evaluating primary care services according to age, ethnicity, physical health and educational level (34) but we did not find this to be the case. We did find, however, that women, respondents with children, rural dwellers, and self-employed people or those not working due to disability were more likely to report a problem (Table 1). Some of these groups might be more frequent users of primary care; in the pilot study we observed that more frequent users of primary care were more likely to report experiencing a problem.(24) We also observed that respondents identifying with an ethnic minority group were less likely to discuss their problem with a member of primary care staff. Previous work in secondary care suggested that gender, educational level and employment status were associated with a patient's willingness to question healthcare staff.(35) Generally there were only small differences in demographics between patients in terms of being more or less likely to perceive, or discuss, a problem and it is important to consider each person's problem equally and encourage all groups, including minorities, to share their concerns.

We found that the survey respondents had similar views to clinicians and researchers in what constituted a potentially-harmful preventable problem given that the patient-described scenarios fit well in to a taxonomy designed and used by clinicians and researchers.(26, 29-30) We did not identify any new types of potentially-harmful preventable-problems unique to the patient perspective in primary care. Furthermore the clinicians and members of the public were consistent in which scenarios they ranked as more likely to be potentially harmful but patients have a much lower threshold for concern than clinicians e.g. just 8% of the 300 patient-reported scenarios were ranked by clinicians as "at least probably" a potentially-harmful preventable problem whereas for the members of the public it was 39%. While this may not be surprising it is important in the context of the discussion above. Clinicians may need to address patient-perceived problems that they do not believe to be harmful if they seek to improve public confidence and trust in primary care.

Strengths and weaknesses of the study

This large population level survey allowed for generalizable estimates of the frequency of patient-perceived potentially-harmful preventable-problems in primary care in GB for the first time and highlights that primary care clinicians tend to judge that the patient-perceived problems are unlikely to be potentially harmful. We have verified that our survey population is similar to the English population in terms of their confidence and trust in their GP as reported in the English GP Patient survey. Previous UK studies (26) have recruited through GP practices whereby patients may be reluctant to disclose problems or answer honestly in case of compromising the patient-clinician relationship; indeed we report here that some patients did not wish to discuss their concern with

For peer review only - http://bmjopen.bmj.com/site/about/guidelines.xhtml

primary care staff for this, and similar, reasons. Furthermore we believe that we have comprehensively captured the patient perspective through involving members of the public as research partners from study design through data acquisition to analysis and reporting. (24) We collected data related to problems occurring over the last 3 years and our denominator is patients not consultations. Time is an important tool for a primary care clinician but also problems arise over time, and the time of occurrence cannot always be assigned to a single consultation, especially with errors of omission that are associated with greater harm in primary care.(36). Reporting adverse events at a rate per consultation does not reflect the reality of the patient journey in primary care where the concept of patient safety as the management of risk over time fits well with the longer time scales.(2) The use of time in this way needs to be communicated to patients given that the most frequently suggested strategy for preventing the problem was quicker access to primary care including investigations (26%, Table 3).

The main weakness of the study is the self-reported nature of the problems and consequent relatively high proportion of scenarios that did not provide adequate information for ranking by clinicians (in their opinion). Arguably this would be improved by using a clinically trained interviewer but this could have biased the scenarios towards the clinician perspective and problems occurring outside of general practice might have gone unnoticed. Furthermore the cost of employing clinician interviewers would have been prohibitive for such a large scale survey. Ipsos MORI interviewers are accustomed to asking questions about healthcare; indeed they administer the annual GP patient survey.(28) A further weakness is that the patient suggestions regarding prevention tended to be non-specific. Collecting patients' suggestions about preventing harm was a secondary aim of this survey but patients did engage with the question and further work in partnership with clinicians is needed to develop this aspect of the survey further.

Strengths and weaknesses in relation to other studies

There are few studies undertaken from the patient perspective at the population level but the annual rates are similar to a Spanish study (7.6% vs 7%, 17). A Health Foundation research scan estimated a 1 to 2% adverse event rate per consultation (37) similar to our finding following clinician review (although we do not use consultations as the denominator). A face to face interview in family practice waiting rooms in the USA reported that 16% of respondents believed a physician had made a mistake in their care.(38) The types of problem and patient responses to the problem are similar to those that have been described qualitatively (1, 21, 39-40) but we have taken this further by using a well-defined denominator to quantify the frequency of occurrence and other descriptors of the problem from the patient's perspective.

Meaning of the study: possible explanations and implications for clinicians and policymakers

There are potentially a large number of patients in GB who believe they have experienced a potentially-harmful preventable problem in primary care but, based on the problems described by patients in this study, primary care clinicians rarely agree that these problems are likely to be potentially harmful. There are already many initiatives in UK primary care aiming to address patient safety but how do we address the patient-perceived problems that clinicians do not recognise as potentially harmful? Similar differences have been observed in UK secondary care where staff

For peer review only - http://bmjopen.bmj.com/site/about/guidelines.xhtml

measures of patient safety culture were not correlated with patient measures.(41) These differing views are likely to be multi-factorial in nature, for example perhaps clinicians are considering the problem from a medico-legal perspective or as a matter of allocation of limited resources *e.g.* disagreement about whether emotional discomfort or wasted time constitutes patient harm? (42) Conversely have the members of the public prioritised sensitivity over specificity or taken a more precautionary approach. Previous qualitative work has observed that, for patients, safety in primary care safety is contingent on the clinician patient relationship where among professionals the systems approach to patient safety is prevalent.(1) While reconciling the differing perspectives of patient and clinician may not be realisable, our study suggests that providing opportunities for, and encouraging, patients to discuss their concerns informally with a member of the primary care team may help with building trust, clarifying expectations and ensuring understanding. The patient suggestions for preventing their perceived problem seem to be asking for more patient centred care where healthcare is in partnership and patients are included in decisions.(43) Including patients more actively in healthcare may also help diminish the patient's expectations of certainty that seem to be common despite primary care being inherently uncertain.(44) Future work should focus on strategies to encourage patients and clinicians to work together to ensure that primary care not only is safe but is also perceived to be safe by patients.

Acknowledgements: The authors would like to express their thanks and appreciation for the work done by the Mary Aldred, Gitanjali Holt, Manoj Mistry, Carole Bennett and Lindsey Brown in coding the patient-described scenarios. Also thank you to the PPI groups who were involved in the piloting of the survey; HelpBeatDiabetes, The Primary Care Research in Manchester Engagement Resource, Associate Research User Group of the Greater Manchester Primary Care Patient Safety Translational Research Centre, The Nowgen Centre, The Citizen Scientist project and North West People in Research Forum. For more information see http://bmjopen.bmj.com/content/bmjopen/8/2/e017786.full.pdf

Contributors: SJS, AD, JB and CG conceived and designed the study. SJS, AD, JB, CG, AE, PB, JA, DT, SL, AD, RD and NM analysed the data. SJS wrote the manuscript, and is guarantor. AD, JB, CG, AE, PB, JA, DT, SL, AD, RD, NM and SC edited the manuscript.

Funding: This study was funded by the National Institute for Health Research (http://www.nihr.ac.uk) through the Greater Manchester Primary Care Patient Safety Translational Research Centre (NIHR GM PSTRC), grant number gmpstrc-2012-1. The views expressed are those of the author(s) and not necessarily those of the NHS, the NIHR or the Department of Health. The funders had no role in study design, data collection and analysis, decision to publish, or preparation of the manuscript.

Competing interests: All authors have completed the ICMJE uniform disclosure form at www.icmje.org/coi_disclosure.pdf (available on request from the corresponding author) and declare no support from any organisation for the submitted work; no financial relationships with any organisations that might have an interest in the submitted work in the previous three years, no other relationships or activities that could appear to have influenced the submitted work.

Ethical approval: University of Manchester Ethics Committee 2 Approval 15372. Respondents to the Ipsos MORI face to face omnibus are not asked to sign a consent document, the invitation into the

For peer review only - http://bmjopen.bmj.com/site/about/guidelines.xhtml

house after agreement to take part in the survey is considered to be consent. All respondents were provided with the participant information sheet before completing the survey questions specific to this study which explains that participation is entirely voluntary and the participant may choose to stop answering the questions at any time.

Copyright statement: the Corresponding Author has the right to grant on behalf of all authors and does grant on behalf of all authors, an exclusive licence on a worldwide basis to the BMJ Publishing Group Ltd to permit this article (if accepted) to be published in BMJ editions and any other BMJPGL products and sublicences such use and exploit all subsidiary rights, as set out in our licence.

Transparency declaration: SJS affirms that this manuscript is an honest, accurate, and transparent account of the study being reported; that no important aspects of the study have been omitted; and that any discrepancies from the study as planned have been explained.

Data sharing: Raw data (coded only) is available from jill.stocks@manchester.ac.uk

Figure legends

Footnote to figure 1: See Tables A&B, online Appendix 1 for details of coding; A coded to 2 levels, B coded to 1 level, C medication problems coded to 3 levels

Fig 1. Numbers of patient-perceived problems occurring in the last 12 months categorised according to the patient's description with clinician ranking as to the likelihood it is a potentially-harmful preventable problem (Table E, online Appendix 1).

Figure 2. Median clinician and members of the public estimates of the likelihood that the patient describes a potentially-harmful preventable-problem occurring in the last 12 months

For peer review only - http://bmjopen.bmj.com/site/about/guidelines.xhtml

Box 1. Brief summary of questionnaire – see online Appendix 1 for full version

Q1. Did you have confidence and trust in the GP you saw or spoke to at your last appointment? (benchmarking question)

Q2a. Have you experienced a situation with a primary care service where your health has ACTUALLY been made worse by a problem or error that could have been prevented?

Q2b. And have you experienced a situation with a primary care service where you SUSPECTED your health has been made worse by a problem or error that could have been prevented?

Q2c. And have you experienced a situation with a primary care service where your health could have been made worse had someone not NOTICED a problem or error?

Q2d. And have you experienced a situation with a primary care service where there was a problem or error that could have been prevented but it did not make your health worse?

If "yes" to more than one of Q2a-d ask Q2e to identify which happened most recently

If "no" to Q2a-d go to Q11

Q3. Thinking about the most recent occasion where you experienced a preventable problem or error caused by the primary care service, when did this occur?

Q4. Thinking about the most recent occasion, which primary care service were you using when the problem or error occurred?

Q5. Thinking about the most recent problem or error you experienced, can you briefly describe what it was and how it happened?

Q6. In your opinion, how, if at all, could the problem or error have been avoided?

Q7. Were you able to talk about the problem or error with anybody WORKING IN THE PRIMARY CARE SERVICE?

Q8. You said you were able to discuss the problem or error with somebody working in primary care. Please describe their job or role and their response.

Q9. Which of the following reasons, if any, best describes why you were unable to talk about the problem or error with somebody working in the primary care service?

Q10. In the last 12 months, have any of the following happened to you **while** using primary care, or not? *If yes go to Q4* (See online Appendix 1 for list of preventable problems)

Q11. Do you, personally, work as a Healthcare Professional in any capacity? For example, a doctor/nurse/therapist/pharmacist/other NHS staff, etc.

For peer review only - http://bmjopen.bmj.com/site/about/guidelines.xhtml

1 Table 1. Prevalence of respondents reporting a potentially-harmful preventable problem within the
2 last 12 months and unadjusted and adjusted odds ratios estimated by logistic regression

Respondent characteristics (total) N=3984	Reported problem in last 12 months (%) n=300	Unadjusted OR–all reports	Adjusted[1] OR-all reports	Adjusted[1] OR after GP review (lower threshold[2]) n=97
**Gender (1 missing)**				
Male (1950)	111 (6%)	1 (ref)	1 (ref)	1 (ref)
Female (2033)	189 (9%)	1.7 (1.3 to 2.2)	1.7 (1.2 to 2.2)	2.3 (1.3 to 3.8)
**Age (years)**				
15 to 24 (533)	38 (7%)	1 (ref)	1 (ref)	1 (ref)
25 to 34 (573)	54 (9%)	1.4 (0.9 to 2.1)	0.7 (0.4 to 1.3)	0.4 (0.2 to 1.2)
35 to 44 (528)	30 (6%)	0.8 (0.5 to 1.3)	0.4 (0.2 to 0.8)	0.1 (0.0 to 0.6)
45 to 54 (629)	54 (9%)	1.2 (0.8 to 1.9)	0.7 (0.4 to 1.4)	0.5 (0.2 to 1.5)
55 to 64 (654)	60 (9%)	1.3 (0.9 to 2.0)	0.8 (0.4 to 1.6)	0.7 (0.2 to 2.0)
65 to 74 (609)	41 (7%)	0.9 (0.6 to 1.5)	0.5 (0.2 to 1.3)	0.7 (0.2 to 3.0)
75 or older (458)	23 (5%)	0.7 (0.4 to 1.2)	0.3 (0.1 to 0.9)	0.3 (0.1 to 1.9)
**Employment status (3 missing)**				
Paid job - full or part time (1719)	119 (7%)	1 (ref)	1 (ref)	1 (ref)
Full time student (283)	14 (5%)	0.7 (0.4 to 1.2)	0.4 (0.1 to 1.1)	0.4 (0.1 to 1.8)
Not working - long term illness/disability (133)	22 (17%)	2.7 (1.6 to 4.4)	2.3 (1.2 to 4.6)	0.9 (0.3 to 3.1)
Not working - other reason (267, includes unemployed)	24 (9%)	1.3 (0.8 to 2.1)	1.3 (0.7 to 2.4)	0.4 (0.1 to 1.4)
Not working - Housewife/husband (201)	19 (9%)	1.4 (0.8 to 2.3)	1.0 (0.5 to 2.0)	0.3 (0.1 to 1.2)
Retired (1198)	80 (7%)	1.0 (0.7 to 1.3)	1.4 (0.8 to 2.6)	0.5 (0.2 to 1.3)
Self-employed (180)	20 (11%)	1.7 (1.0 to 2.8)	2.0 (1.1 to 3.5)	0.5 (0.1 to 2.3)
**Region of domicile (23 missing)**				
Greater London (565)	38 (7%)	1 (ref)	1 (ref)	1 (ref)
East Midlands (262)	9 (3%)	0.5 (0.2 to 1.0)	0.6 (0.2 to 1.4)	0.4 (0.0 to 3.6)
East of England (425)	27 (6%)	0.9 (0.6 to 1.6)	0.6 (0.3 to 1.1)	1.8 (0.5 to 5.8)
North (176)	15 (9%)	1.3 (0.7 to 2.5)	0.8 (0.3 to 1.7)	0.7 (0.1 to 4.3)
North-West (490)	46 (9%)	1.4 (0.9 to 2.2)	1.0 (0.6 to 1.9)	1.4 (0.4 to 4.5)
Scotland (372)	27 (8%)	1.1 (0.7 to 1.8)	0.8 (0.4 to 1.6)	1.8 (0.5 to 6.1)
South East (444)	32 (7%)	1.1 (0.6 to 1.6)	1.1 (0.6 to 2.0)	2.2 (0.7 to 7.0)
South West (281)	33 (12%)	1.8 (1.1 to 3.0)	1.0 (0.5 to 2.0)	1.9 (0.5 to 6.6)
Wales (196)	15 (8%)	1.1 (0.6 to 2.1)	0.6 (0.3 to 1.4)	2.2 (0.5 to 8.5)
West Midlands (377)	19 (5%)	0.7 (0.4 to 1.3)	0.6 (0.3 to 1.3)	1.1 (0.3 to 4.4)
Yorks & Humberside (373)	39 (10%)	1.6 (1.0 to 2.6)	1.2 (0.7 to 2.3)	2.7 (0.8 to 8.4)
**Ethnicity (18 missing)**				
White (3591)	271 (8%)	1 (ref)	1 (ref)	1 (ref)
Other ethnicity (475)	26 (5%)	0.7 (0.5 to 1.0)	1.2 (0.7 to 2.2)	1.1 (0.4 to 3.0)
**Type of community**				
Urban, suburban (3051)	203 (7%)	1 (ref)	1 (ref)	1 (ref)
Rural (933)	97 (10%)	1.6 (1.3 to 2.1)	1.9 (1.3 to 2.7)	2.0 (1.1 to 3.5)
**Parental responsibility**				
Zero children under 19 (2839)	192 (7%)	1 (ref)	1 (ref)	1 (ref)
Child(ren) aged up to 19 (1145)	108 (9%)	1.4 (1.1 to 1.8)	1.2 (0.8 to 1.7)	1.5 (0.8 to 2.8)

For peer review only - http://bmjopen.bmj.com/site/about/guidelines.xhtml

**Tenure (31 missing)**					
Mortgaged (1042)	84 (8%)	1 (ref)	1 (ref)	1 (ref)
Owned outright (1441)	87 (6%)	0.7 (0.5 to 1.0)	0.8 (0.5 to 1.2)	0.9 (0.4 to 1.8)
Rented-housing association (301)	42 (14%)	1.8 (1.2 to 2.7)	1.3 (0.7 to 2.2)	1.1 (0.4 to 2.9)
Rented-private landlord (719)	49 (7%)	0.8 (0.6 to 1.2)	0.9 (0.6 to 1.5)	0.9 (0.4 to 2.1)
Rented-local authority (422)	31 (7%)	0.9 (0.6 to 1.4)	0.6 (0.3 to 1.2)	1.0 (0.4 to 2.8)
Other (28)	4 (14%)	1.9 (0.6 to 5.6)	2.2 (0.6 to 8.2)	-[3]
**Confidence and trust in GP at last appointment?**				
Yes definitely (3031)	144 (5%)	1 (ref)	-	-
Yes, to some extent (611)	68 (11%)	2.5 (1.9 to 3.4)	-	-
No, not at all (311)	88 (28%)	7.9 (5.9 to 10.7)	-	-
Don't know /can't say (31)	0 (0%)	-	-	-

[1]adjusted for gender, age, employment status, ethnicity, tenure, region of domicile, type of community, parental responsibility, highest level of education achieved, marital status, social grade, household income

[2]see Table E online Appendix 1

[3]zero problems in this category

For peer review only

Table 2. Details of the patient's response to the potentially-harmful preventable-problem and the primary care service involved

**Primary care service involved**	**Problems in last 12 months n=300**	**All problems analysed[1] n=564**
GP surgery	211 (70%)	395 (70%)
Dental surgery	27 (9%)	50 (9%)
Walk in clinic	16 (5%)	22 (4%)
Ambulance/A&E/ Out of hours care	16 (5%)	28 (5%)
Pharmacy	10 (3%)	19 (3%)
Community or district nursing	8 (3%)	21 (4%)
Mental health services	6 (1%)	8 (1%)
Opticians	4 (1%)	5 (1%)
Physiotherapy (in primary care)	2 (1%)	5 (1%)
missing /nk	0 (<1%)	11 (2%)
**Did you discuss the problem with primary care staff?**	**Problems in last 12 months n=300**	**All problems analysed[1] n=564**
Yes	145 (48%)	273 (48%)
No	153 (51%)	273 (48%)
missing /nk	2 (1%)	18 (3%)
**Reasons why patients did not discuss the problem with primary care staff**	**Problems in last 12 months n=153**	**All problems analysed[1] n=273**
Patient had the opportunity but did not feel comfortable discussing the problem or error	16 (10%)	43 (16%)
Patient could not find anybody with whom to discuss the problem or error	37 (24%)	75 (27%)
Patient was not concerned about the problem or error	25 (16%)	37 (14%)
Patient did not notice the problem or error or trusted the clinician's judgement at the time	11 (7%)	25 (9%)
Patient was too distressed or ill to discuss the problem or error	18 (12%)	30 (11%)
Other - problem was resolved in another way by the patient without involving primary care	10 (7%)	13 (5%)
Other - patient believed primary care staff would not be interested in the problem or would not take it seriously or it would not improve primary care	7 (5%)	14 (5%)
Other – patient believed that discussing the problem with a primary care staff might have negative implications for their future care	6 (4%)	6 (2%)
Other - patient did know that they were allowed to express an opinion or how to raise the problem	5 (3%)	5 (2%)
Other - patient accepts that such problems will arise in primary care or didn't want to use primary care resources when primary care staff are very busy	5 (3%)	6 (2%)
Other - patient intends to discuss with primary care	4 (3%)	6 (2%)

For peer review only

For peer review only - http://bmjopen.bmj.com/site/about/guidelines.xhtml

professional at the next opportunity		
Don't Know/missing	9 (6%)	13 (5%)
**Profession of discussant**	**Problems in last 12 months n=145**	**All problems analysed[1] n=273**
GP/practice nurse	66 (46%)	144 (53%)
Practice manager/receptionist/administrator	25 (17%)	39 (14%)
Pharmacist/dispenser	7 (5%)	14 (5%)
General Dental Practitioner	8 (6%)	18 (7%)
Hospital doctor or nurse/A&E or OOH staff/paramedic	15 (10%)	18 (7%)
Other primary care staff	14 (10%)	17 (6%)
PALS or NHS direct staff	1 (1%)	2 (1%)
Unclear, don't know or missing	9 (6%)	21 (8%)
**Role of discussant in patient's care**	**Problems in last 12 months n=145**	**All problems analysed[1] n=273**
Member of staff central to respondent's care	60 (41%)	112 (41%)
Member of staff in the same team or organisation	35 (24%)	84 (31%)
Member of staff in a different team or organisation	31 (21%)	40 (15%)
Role of member of staff is unclear	8 (6%)	20 (7%)
missing	11 (8%)	17 (%)

1

2 [1]All problems analysed includes scenarios arising from Ipsos MORI survey in the last 3 years and the pilot survey (24) within the last 12 months

For peer review only - http://bmjopen.bmj.com/site/about/guidelines.xhtml

Table 3. Patient suggestions as to how the potentially-harmful preventable problem might have been prevented

How could it be prevented?	Problems in last 12 months n=300	All problems analysed[1] n=564
**1. More resources - total**	**100 (33%)**	**157 (28%)**
1.1 Quicker access to primary care	43 (14%)	62 (11%)
1.2 More thorough and quicker investigations	35 (12%)	59 (10%)
1.3 Fewer demands on primary care – more staff or fewer patients	7 (2%)	12 (2%)
1.4 More time with clinicians for treatment and diagnosis	8 (3%)	12 (2%)
1.5 Improved access to social care	3 (1%)	3 (1%)
1.6 More follow-up by primary care	2 (1%)	3 (1%)
1.7 Improved continuity of care	1 (<1%)	2 (<1%)
1.8 Access to a second opinion	1 (<1%)	2 (<1%)
1.9 Provision of resources to manage long term conditions	0	2 (<1%)
**2. Improved communication and involvement of patients - total**	**53 (18%)**	**92 (16%)**
1.1 Listen to the patient and trust their judgement more	36 (12%)	68 (12%)
1.2 Tell patients about their diagnosis, test results, changes in medication or loss of results	10 (3%)	15 (3%)
1.3 Improve communication between staff (within or outside primary care)	7 (2%)	9 (2%)
**3. Better organisation and administration - total**	**27 (9%)**	**48 (9%)**
3.1 Follow up referrals and appointments to ensure they happen, be consistent in sending routine reminders	12 (4%)	23 (4%)
3.2 Log in or process results as soon as received to avoid loss	5 (2%)	7 (1%)
3.3 Keep the notes up to date, well-organised, safe and ensure information is transcribed accurately	9 (3%)	15 (3%)
3.4 Keep a record of the location of equipment	0	1 (<1%)
3.5 Improve the method of appointment allocation	0	1 (<1%)
3.6 Fine patients for not attending appointments	1 (<1%)	1 (<1%)
**4. Improved prescribing systems - total**	**21 (7%)**	**45 (8%)**
4.1 More when checks on prescribing and dispensing	19 (6%)	32 (6%)
4.2 Check repeat prescriptions carefully, especially for transcribing errors	2 (1%)	10 (2%)
4.3 Use medication reviews and IT clinical decision support systems	0	3 (1%)
**5. Better clinical practice - total**	**17 (6%)**	**47 (8%)**
5.1 Take in to account all the patient's information - their medical history and results and letters	7 (2%)	27 (5%)
5.2 Address the patient's problem in some way – patients can feel their problem is being ignored	9 (3%)	18 (3%)
5.3 Act on advice from other clinicians and test results	1 (<1%)	2 (<1%)
**6. Staff training - total**	**22 (7%)**	**53 (9%)**
6.1 More informed and better trained staff	22 (7%)	53 (9%)

For peer review only - http://bmjopen.bmj.com/site/about/guidelines.xhtml

**Other responses - total**	**60 (20%)**	**122 (22%)**
•Don't know/missing	28 (9%)	64 (11%)
•Problem was due to an individual member of staff	6 (2%)	11 (2%)
•Do not make wrong, late, delayed diagnosis	7 (2%)	15 (3%)
•Prescribe right, better, different, more, less medicine	8 (3%)	15 (3%)
•Should have been referred	6 (2%)	9 (2%)
•Better organisation	3 (1%)	4 (1%)
•Patient recognised their own responsibility	2 (1%)	2 (<1%)
•Laboratory procedures were the problem	0	2 (<1%)

[1]All problems analysed includes scenarios arising from Ipsos MORI survey in the last 3 years and the pilot survey (24) within the last 12 months

For peer review only

For peer review only - http://bmjopen.bmj.com/site/about/guidelines.xhtml

References

1. Rhodes P, Campbell S, Sanders C. Trust, temporality and systems: how do patients understand patient safety in primary care? A qualitative study. Health Expectations. 2016;19(2):253-63.

2. Vincent C, Amalberti R. Safer Healthcare: Strategies for the Real World. Springer, 2016. http://www.springer.com/gb/book/9783319255576 Accessed 04/04/17

3. The Health Foundation. Evidence scan: Involving patients in improving safety. January 2013. http://www.health.org.uk/sites/health/files/InvolvingPatientsInImprovingSafety.pdf Accessed 04/04/17

4. King A, Daniels J, Lim J, Cochrane DD, Taylor A, Ansermino JM. Time to listen: a review of methods to solicit patient reports of adverse events. Qual Saf Health Care. 2010;19(2):148-57.

5. Kuzel AJ, Woolf SH, Gilchrist VJ, Engel JD, LaVeist TA, Vincent C, et al. Patient reports of preventable problems and harms in primary health care. Ann Fam Med. 2004;2(4):333-40.

6. Blendon RJ, DesRoches CM, Brodie M, Benson JM, Rosen AB, Schneider E, et al. Views of practicing physicians and the public on medical errors. N Engl J Med. 2002;347(24):1933-40.

7. Hotvedt R, Forde OH. Doctors are to blame for perceived medical adverse events. A cross sectional population study. The Tromso Study. BMC health services research. 2013;13:46.

8. The Health Foundation. Evidence Scan: Improving safety in primary care. November 2011. http://www.health.org.uk/sites/health/files/ImprovingSafetyInPrimaryCare.pdf Accessed 04/04/17

9. Weingart SN et al. RS What can hospitalized patients tell us about adverse events? Learning from patient-reported incidents. J Gen Intern Med. 2005 Sep;20(9):830-6.

10. Weissman JS, Schneider EC, Weingart SN, et al. Comparing patient-reported hospital adverse events with medical record review: do patients know something that hospitals do not? Ann Intern Med. 2008 Jul 15;149(2):100-8

11. Southwick FS, Cranley NM, Hallisy JA. A patient-initiated voluntary online survey of adverse medical events: the perspective of 696 injured patients and families. BMJ Qual Saf. 2015;24(10):620-9.

12. Longtin Y, Sax H, Leape LL, Sheridan SE, Donaldson L, Pittet D. Patient participation: current knowledge and applicability to patient safety. Mayo Clinic proceedings. 2010;85(1):53-62.

13. Sandars J, Esmail A. The frequency and nature of medical error in primary care: understanding the diversity across studies. Family practice. 2003;20(3):231-6.

14. Panesar SS, deSilva D, Carson-Stevens A, Cresswell KM, Salvilla SA, Slight SP, et al. How safe is primary care? A systematic review. BMJ Quality & Safety. 2016;25(7):544-53.

15. Michel P, Brami J, Chaneliere M, Kret M, Mosnier A, Dupie I, et al. Patient safety incidents are common in primary care: A national prospective active incident reporting survey. PLoS One. 2017;12(2):e0165455.

For peer review only - http://bmjopen.bmj.com/site/about/guidelines.xhtml

16. Northcott H, Vanderheyden L, Northcott J, Adair C, McBrien-Morrison C, Norton P, et al. Perceptions of preventable medical errors in Alberta, Canada. International journal for quality in health care : journal of the International Society for Quality in Health Care / ISQua. 2008;20(2):115-22.

17. Mira JJ, Nebot C, Lorenzo S, Perez-Jover V. Patient report on information given, consultation time and safety in primary care. Qual Saf Health Care. 2010;19(5):e33.

18. Wasson JH, MacKenzie TA, Hall M. Patients use an internet technology to report when things go wrong. Qual Saf Health Care. 2007;16(3):213-5.

19. Ricci-Cabello I, Goncalves DC, Rojas-Garcia A, Valderas JM. Measuring experiences and outcomes of patient safety in primary care: a systematic review of available instruments. Family practice. 2015;32(1):106-19.

20. Ricci-Cabello I, Avery AJ, Reeves D, Kadam UT, Valderas JM. Measuring Patient Safety in Primary Care: The Development and Validation of the "Patient Reported Experiences and Outcomes of Safety in Primary Care" (PREOS-PC). Ann Fam Med. 2016;14(3):253-61.

21. Ricci-Cabello I, Saletti-Cuesta L, Slight SP, Valderas JM. Identifying patient-centred recommendations for improving patient safety in General Practices in England: a qualitative content analysis of free-text responses using the Patient Reported Experiences and Outcomes of Safety in Primary Care (PREOS-PC) questionnaire. Health Expectations. 2017.

22. NHS England. General practice patient safety reporting form launched. 26th February 2015. https://www.england.nhs.uk/2015/02/gp-patient-safety-reporting/ Accessed 04/04/17

23. European Commission. Special Eurobarometer 411 Patient Safety and Quality of Care 2014. DOI:10.2772/33467 https://ec.europa.eu/health/sites/health/files/patient_safety/docs/ebs_411_sum_en.pdf Accessed 04/04/17

24. Stocks SJ, Donnelly A, Esmail A, Beresford J, Luty S, Deacon R, et al. Development and piloting of a survey to estimate the frequency and nature of potentially-harmful preventable-problems in primary care from a UK patient's perspective. BMJ Open. 2017;8:e017786. doi:10.1136/ bmjopen-2017-01778

25. NHS Improvement. National quarterly data on patient safety incident reports. September 2016. https://improvement.nhs.uk/resources/national-quarterly-data-patient-safety-incident-reports-september-2016/ Accessed 04/04/17

26. Hutchinson A, Young TA, Cooper KL, McIntosh A, Karnon JD, Scobie S, et al. Trends in healthcare incident reporting and relationship to safety and quality data in acute hospitals: results from the National Reporting and Learning System. Qual Saf Health Care. 2009;18(1):5-10.

27. Ipsos MORI Face-to-Face Omnibus (Capibus) https://www.ipsos-mori.com/ourexpertise/omnibusservices/capibus.aspx Accessed 04/04/17

28. NHS England. GP Patient Survey – National summary report. January 2016. http://gp-survey-production.s3.amazonaws.com/archive/2016/January/January+2016+National+Summary+Report.pdf Accessed 04/04/17

For peer review only - http://bmjopen.bmj.com/site/about/guidelines.xhtml

29. Dovey SM, Meyers DS, Phillips RL, Jr., Green LA, Fryer GE, Galliher JM, et al. A preliminary taxonomy of medical errors in family practice. Qual Saf Health Care. 2002;11(3):233-8.

30. Makeham MA, Dovey SM, County M, Kidd MR. An international taxonomy for errors in general practice: a pilot study. Med J Aust. 2002;177(2):68-72.

31. McGraw K WS. Forming inferences about some intraclass correlation coefficients. Psychological Methods. 1994;1(1):30-46.

32. Bailey E, Tickle M, Campbell S. Patient safety in primary care dentistry: where are we now? Br Dent J. 2014;217(7):339-44.

33. Ensaldo-Carrasco E, Suarez-Ortegon MF, Carson-Stevens A, Cresswell K, Bedi R, Sheikh A. Patient Safety Incidents and Adverse Events in Ambulatory Dental Care: A Systematic Scoping Review. Journal of patient safety. 2016;08:08.

34. Campbell JL, Ramsay J, Green J. Age, gender, socioeconomic, and ethnic differences in patients' assessments of primary health care. Quality in health care : QHC. 2001;10(2):90-5.

35. Davis RE, Koutantji M, Vincent CA. How willing are patients to question healthcare staff on issues related to the quality and safety of their healthcare? An exploratory study. Qual Saf Health Care. 2008;17(2):90-6.

36. The WHO Safer Primary Care Expert Working Group. Safer Primary Care: A Global Challenge. Summary of Inaugral Meeting 2012. http://www.who.int/patientsafety/summary_report_of_primary_care_consultation.pdf Accessed 04/04/17

37. The Health Foundation. Evidence Scan: Levels of Harm in Primary Care. November 2011. http://www.health.org.uk/sites/health/files/LevelsOfHarmInPrimaryCare.pdf Accessed 04/04/17

38. Kistler CE, Walter LC, Mitchell C, Sloane PD. Patient perceptions of mistakes in ambulatory care. Archives of Internal Medicine. 2010;170(16):1480-7.

39. Hernan AL, Giles SJ, Fuller J, Johnson JK, Walker C, Dunbar JA. Patient and carer identified factors which contribute to safety incidents in primary care: a qualitative study. BMJ Qual Saf. 2015; 24(9):583-93.

40. Litchfield I, et al. Routine failures in the process for blood testing and the communication of results to patients in primary care in the UK: a qualitative exploration of patient and provider perspectives. BMJ Qual Saf. 2015 Nov;24(11):681-90

41. Lawton R, O'Hara JK, Sheard L, Reynolds C, Cocks K, Armitage G, et al. Can staff and patient perspectives on hospital safety predict harm-free care? An analysis of staff and patient survey data and routinely collected outcomes. BMJ Qual Saf. 2015;24(6):369-376.

42. Elder NC, Meulen MV, Cassedy A. The Identification of Medical Errors by Family Physicians During Outpatient Visits. Annals of Family Medicine. 2004;2(2):125-9.

For peer review only - http://bmjopen.bmj.com/site/about/guidelines.xhtml

43. Heneghan C, Glasziou P, Thompson M, Rose P, Balla J, Lasserson D, et al. Diagnostic strategies used in primary care. BMJ. 2009;338.

44. The Health Foundation. Person-centred care made simple. 2014. http://www.health.org.uk/sites/health/files/PersonCentredCareMadeSimple.pdf Accessed 04/04/17

For peer review only

For peer review only - http://bmjopen.bmj.com/site/about/guidelines.xhtml

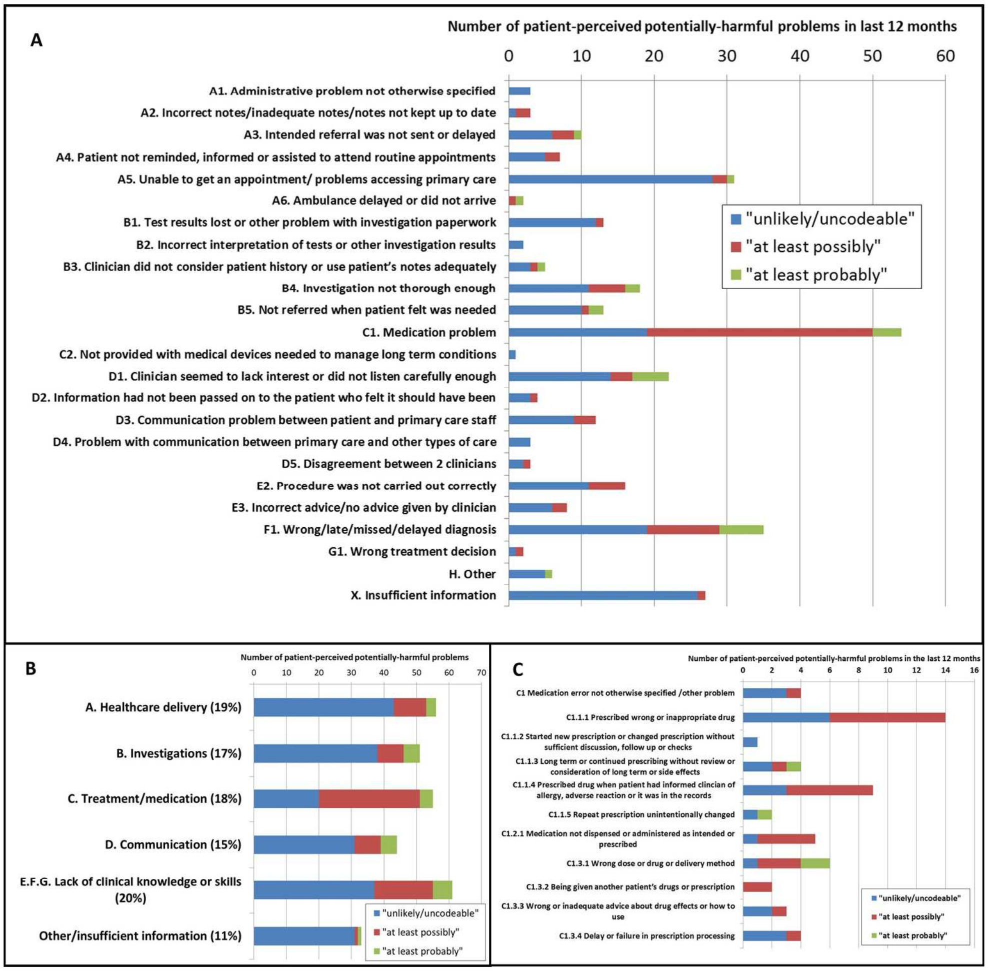

Footnote to figure 1: See Tables A&B, online Appendix 1 for details of coding; A coded to 2 levels, B coded to 1 level, C medication problems coded to 3 levels

Fig 1. Numbers of patient-perceived problems occurring in the last 12 months categorised according to the patient's description with clinician ranking as to the likelihood it is a potentially-harmful preventable problem (Table E, online Appendix 1).

174x170mm (300 x 300 DPI)

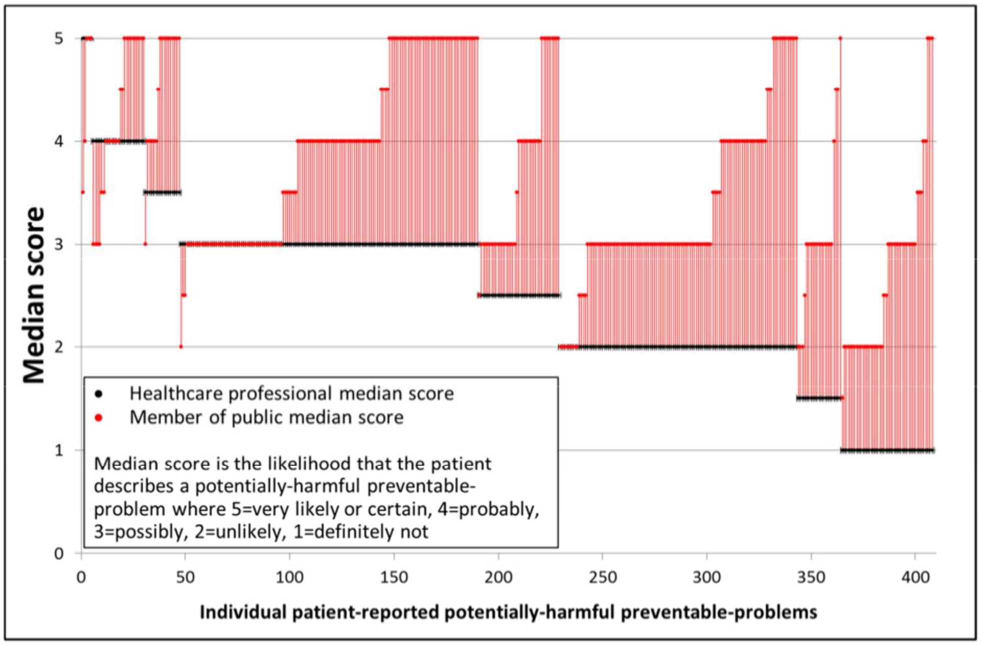

Figure 2. Median clinician and members of the public estimates of the likelihood that the patient describes a potentially-harmful preventable-problem occurring in the last 12 months

81x53mm (300 x 300 DPI)

Appendix 1. Supplementary methods and results

SJ Stocks et al. BMJ Open 2018: The frequency and nature of potentially-harmful preventable-problems in primary care from the patient's perspective with clinician review – a population level survey in Great Britain

Survey administered as part of the Ipsos MORI GB Face to Face Omnibus between 8th and 21st April 2016

We'd now like you to think about the last time you personally had an appointment for yourself, with a GP.

Q1. Did you have confidence and trust in the GP you saw or spoke to at your last appointment?
1. Yes, definitely 2. Yes, to some extent 3. No, not at all 4. Don't know / can't say

INTERVIEWER INSTRUCTION: READ OUT AND DISPLAY ON SCREEN.

The next few questions are about primary care.

Primary Care is the local healthcare that we receive at our GP or dental surgery, NHS walk-in centres, pharmacists (or high street chemist) and optometrists. This also could include all non-hospital care, for example, healthcare service provided by out of hours care, community (or district) nursing, ambulance, physiotherapy or other types of therapy or tests based at a GP surgery, learning disability services and any other non-hospital medical care.

We understand that this is a highly sensitive topic and would therefore like to remind you that any information you give is strictly confidential and will be used for research purposes only. You will not be identifiable as an individual from the responses you give.

At each question, if you do not wish to answer, you can refuse.

For the next question, we'd like you to think about the occasions when you have personally used primary care for yourself.

Q2a. Have you experienced a situation with a primary care service where your health has ACTUALLY been made worse by a problem or error that could have been prevented?
1. Yes 2. No 3. Don't Know

Q2b. And have you experienced a situation with a primary care service where you SUSPECTED your health has been made worse by a problem or error that could have been prevented?
1. Yes 2. No 3. Don't Know

Q2c. And have you experienced a situation with a primary care service where your health could have been made worse had someone not NOTICED a problem or error?
1. Yes 2. No 3. Don't Know

Q2d. And have you experienced a situation with a primary care service where there was a problem or error that could have been prevented but it did not make your health worse?
1. Yes 2. No 3. Don't Know

IF 2 OR MORE SCENARIOS AT Q2a to Q2e ARE CODED 1 THEN ASK Q2e

For peer review only

Q2e. You mentioned you have experienced the following situation(s) with a primary care service. Which of the following did you experience most recently?

1. 'My health was made worse'
2 'I suspect health was made worse'
3 'My health could have been made worse if the problem or error had not been noticed'
4 'There was no effect on my health'

ASK ALL WHO CODE 1 AT Q2
Q3. Thinking about the most recent occasion where you experienced a preventable problem or error caused by the primary care service, when did this occur?

1. In the last 12 months
2. 1 year up to 2 years ago
3. 2 years up to 3 years ago
4. 3 or more years ago

ASK ALL CODING 1 AT Q2 OR 1 AT Q11
Q4. Thinking about the most recent occasion, which primary care service were you using when the problem or error occurred?

1. GP surgery
2. Out of hours care
3. Walk in clinic
4. Dental surgery
5. Pharmacy
6. Community or district nursing
7. Ambulance
8. Opticians
9. Other (please specify)

INTERVIEWER INSTRUCTION: For the next five questions, please record enough information so that somebody else reading the description can understand what happened.

ASK ALL CODING 1 AT Q2 OR 1 AT Q11
Q5. Thinking about the most recent problem or error you experienced, can you briefly describe what it was and how it happened?

Q6 In your opinion, how, if at all, could the problem or error have been avoided?

Q7. Were you able to talk about the problem or error with anybody WORKING IN THE PRIMARY CARE SERVICE?
1. Yes 2. No

INTERVIEWER INSTRUCTION: if prompted, this can be anyone in the primary care service, including for example, the receptionist at a GP surgery or another nurse/doctor who wasn't working directly in their care.

ASK ALL CODING 1 AT Q7
Q8. You said you were able to discuss the problem or error with somebody working in primary care. Please describe their job or role and their response.

For peer review only

ASK ALL CODING 2 AT Q7

Q9. Which of the following reasons, if any, best describes why you were unable to talk about the problem or error with somebody working in the primary care service?

1. I had the opportunity but did not feel comfortable discussing the problem or error
2. I could not find anybody with whom I could discuss the problem or error
3. I was not concerned about the problem or error
4. I did not notice the problem or error
5. I was too distressed to discuss the problem or error
6. Other (please specify)

ASK IF (Q2 '2 OR DK OR REF')

Q10. In the last 12 months, have any of the following happened to you **while** using primary care, or not? 1. Yes 2. No

IF YES AT Q11, REDIRECT TO Q4

(RANDOMISE 1-16(KEEP 2&3 TOGETHER, KEEP 6&7 TOGETHER, KEEP 9&10 TOGETHER), ALLOW DK AND REF)

1. Received a wrong or late diagnosis
2. Was not referred for further investigation when requested by you as a patient
3. Was not referred for further investigation in error by healthcare practitioner (for example, they forgot to refer you onwards)
4. Test results lost or mixed up
5. Received the wrong medicine or wrong dose
6. Should not have been prescribed medicine because of another health problem
7. Should not have been prescribed medicine because of another medication already being taken
8. Poor communication leading to misunderstanding of diagnosis or treatment
9. Not referred to a specialist when needed when requested by you as a patient
10. Not referred to a specialist when needed in error by healthcare practitioner (for example, they forgot to refer you onwards)
11. Received unclear instructions about treatment
12. Not offered access to prevention or screening programmes e.g. CVD/stroke prevention clinics
13. A medical professional failed to recognise or act on vulnerable people's needs e.g. child abuse, suicide risk or mental health problems
14. Mistake with a procedure e.g. dental treatment, injection, ear syringing, physiotherapy
15. Not notified about recommended vaccinations e.g. flu, HPV
16. A medical professional practicing poor hygiene

ASK ALL CODING 1 AT Q2 OR 1 AT Q11

Q11. Do you, personally, work as a Healthcare Professional in any capacity? For example, a doctor/nurse/therapist/pharmacist/other NHS staff, etc.

1. Yes 2. No

For peer review only

Table A. Coding of patient-reported potentially-unsafe scenarios in primary care

**1. Errors in the process of the healthcare delivery system**	
Makeham 2002, Dovey 2002	Common threads reported in this study
1.1. Errors in the process of conducting an administrative task	A1. Administrative problem not otherwise specified
1.1.1. Information filed in wrong place or wrong time	
1.1.2. Unavailability of information that should have been in patients charts<br>1.1.2.1. Entire chart or part of chart could not be accessed when needed<br>1.1.2.2. Care provided was not documented<br>1.1.2.3. Item(s) of information missing from chart	A2. Incorrect notes/inadequate notes/notes not kept up to date
1.1.3. Errors in patient's movement through the healthcare delivery system	A3. Intended referral was not sent or delayed<br>A4. Patient not reminded, informed or assisted to attend regular check-ups or other necessary routine treatments
1.1.4. Errors in the taking and distributing of messages	
1.1.5. Errors in managing appointments for healthcare	A5. Unable to get an appointment/other problems with making appointment<br>A6. Ambulance delayed or did not arrive
**1.2. Errors in the process of investigating a patient's condition**	
1.2.1. Laboratory errors<br>1.2.1.1. Wrong test ordered or test not ordered when appropriate<br>1.2.1.2. Errors in the process of obtaining or processing a laboratory specimen<br>1.2.1.3. Error in the process of physician receiving accurate laboratory results in a timely fashion<br>1.2.1.4. Inappropriate response to an abnormal laboratory result	B1. Test results lost or other problem with investigation or paperwork<br>B2. Incorrect interpretation of tests or other investigation results<br>B3. Clinician did not consider patient history sufficiently/did not use patient's notes adequately<br>B4. Investigation not thorough enough<br>B5. Not referred when patient felt was needed
1.2.3. Errors in the processes of other investigations<br>1.2.3.1. Wrong test ordered or test not ordered when appropriate<br>1.2.3.2. Errors in the process of obtaining or processing of other diagnostic investigation<br>1.2.3.3. Error in the process of physician receiving accurate test results of other investigation in a timely fashion<br>1.2.3.4. Inappropriate response to an abnormal result of other investigation	
**1.3. Errors in the process of treating a patient's condition**	
1.3.1. Errors in the process of treating with medications<br>1.3.1.1. Wrong medication or wrong dose of medication ordered or medication not ordered by physician when appropriate<br>1.3.1.2. Error in the process of delivering a medication order or inappropriate medication order by a provider working under physician supervision<br>1.3.1.3. Error in the process of dispensing medication as ordered	C1. Medication problem<br>C2. Not provided with medical devices needed to manage long term conditions

For peer review only

1.3.2. Errors in other treatments	C3. Problem with dental treatment or diagnosis
**1.4. Errors in the process of communication**	
1.4.1. Errors in communication between primary healthcare provider and patients	D1. Clinician seemed to lack interest in the patient's health problem or did not listen carefully enough<br>D2. Information about the patient's health had not been passed on to the patient who felt it should have been<br>D3. Communication problem between patient and primary care staff
1.4.2. Errors in communication between healthcare providers	D4. Problem with communication between primary care and other types of care including secondary care<br>D5. Disagreement between 2 clinicians
**2. Errors arising from lack of clinical knowledge or skills**	
2.1. Errors in the execution of a clinical task<br>2.1.1. Non-clinical staff made the wrong clinical decision<br>2.1.2. Failed to follow standard practice<br>2.1.3. Lacked needed experience or expertise in a clinical task	E1. Administrative staff seemed to make clinical decisions<br>E2. Procedure was not carried out correctly<br>E3. Incorrect advice/no advice given by clinician
**2.2. Errors in diagnosis**<br>2.2.1. Wrong or delayed diagnosis	F1. Wrong/late/missed/delayed diagnosis
**2.3. Wrong treatment decision**	G1. Wrong treatment decision
	H. Other
	X. Not a problem/ insufficient information/refused/don't know

Table B. Level 4 coding of patient-reported potentially-unsafe medication scenarios

Common threads reported in this study grouped as described by Makeham 2002, Dovey 2002
C1 Medication error not otherwise specified /other problem
• **1.3.1.1. Ordering medications (prescribing)**
C1.1.1 Prescribed wrong or inappropriate drug
C1.1.2 Started new prescription or changed prescription without sufficient discussion, follow up or checks
C1.1.3 Long term or continued prescribing without review or consideration of long term or side effects
C1.1.4 Prescribed drug when should have known contra-indicated *e.g.* patient had informed clinician of allergy, adverse reaction or it was in the records
C1.1.5 Repeat prescription unintentionally changed
C1.1.6 Out of date repeat prescription mistakenly re-issued
• **1.3.1.2./1.3.1.3. Implementing or receiving medications (dispensing or issuing)**
C1.2.1 Medication not dispensed or administered as intended or prescribed
• **1.3.1.1/1.3.1.2./1.3.1.3. Ordering, implementing or receiving medications**
C1.3.1 Wrong dose or drug or delivery method
C1.3.2 Being given another patient's drugs or prescription
C1.3.3 Wrong or inadequate advice about drug effects or how to use
C1.3.4 Delay or failure in prescription processing

For peer review only

Table C. Scoring for likelihood that the patient-reported scenario is potentially-unsafe

Score	How likely do you think it is the patient was correct in thinking that their health might be worsened, or actually was made worse, because of a mistake or a problem in primary care that could have been prevented? Choose from the options below.
5	Very likely or certain (75-100% confident is a potentially unsafe scenario)
4	Probably (50-74% confident is a potentially unsafe scenario)
3	Possibly (25-49% confident is a potentially unsafe scenario)
2	Unlikely (bottom 25% confident is a potentially unsafe scenario)
1	Definitely not a potentially unsafe event (0% chance is a potentially unsafe scenario)
-	Insufficient information
-	Don't know
-	Other - add text at end of row

For peer review only

Supplementary results

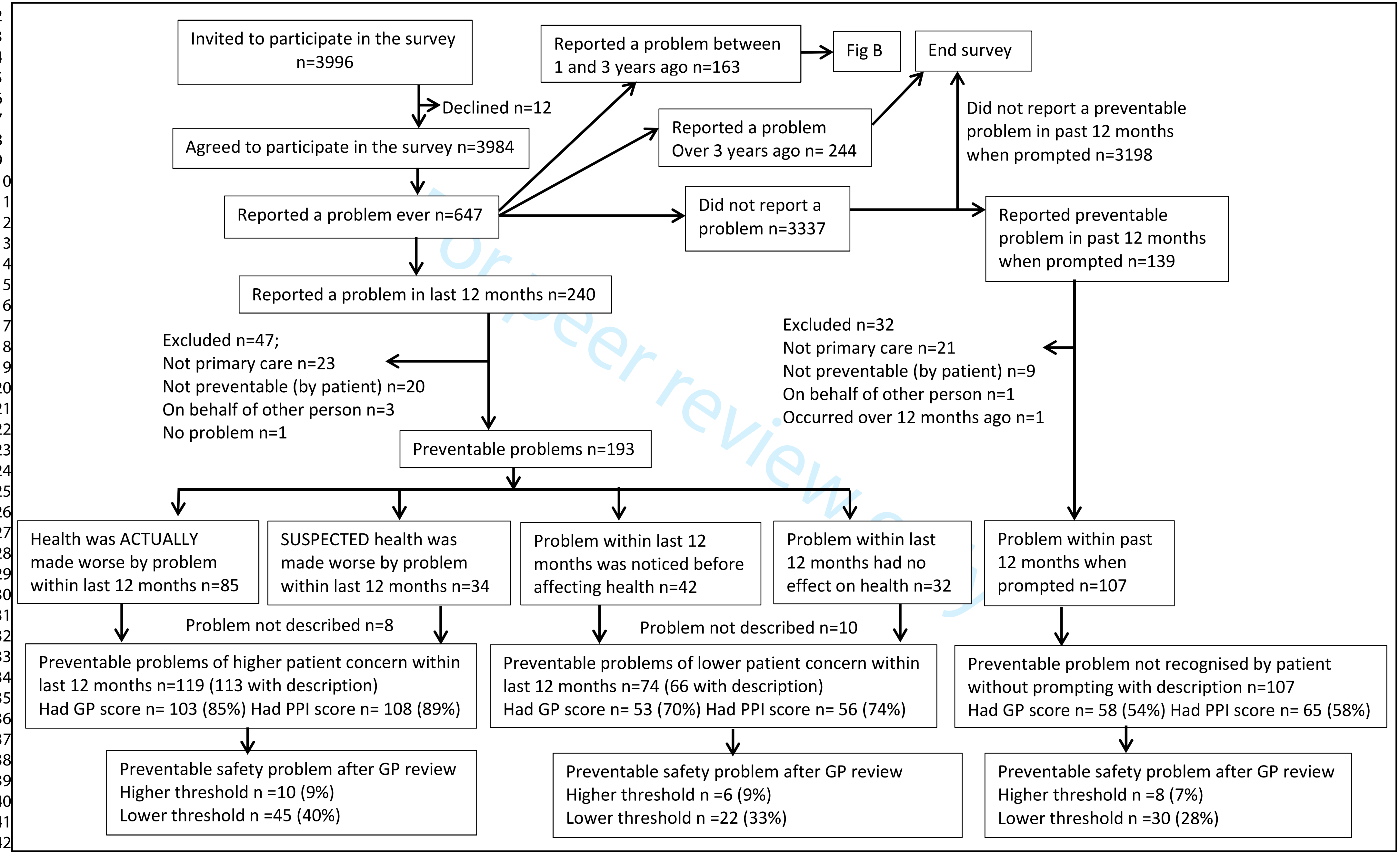

Fig A. Flow chart of participants reporting a potential-harmful preventable-problem within the last 12 months

For peer review only - http://bmjopen.bmj.com/site/about/guidelines.xhtml

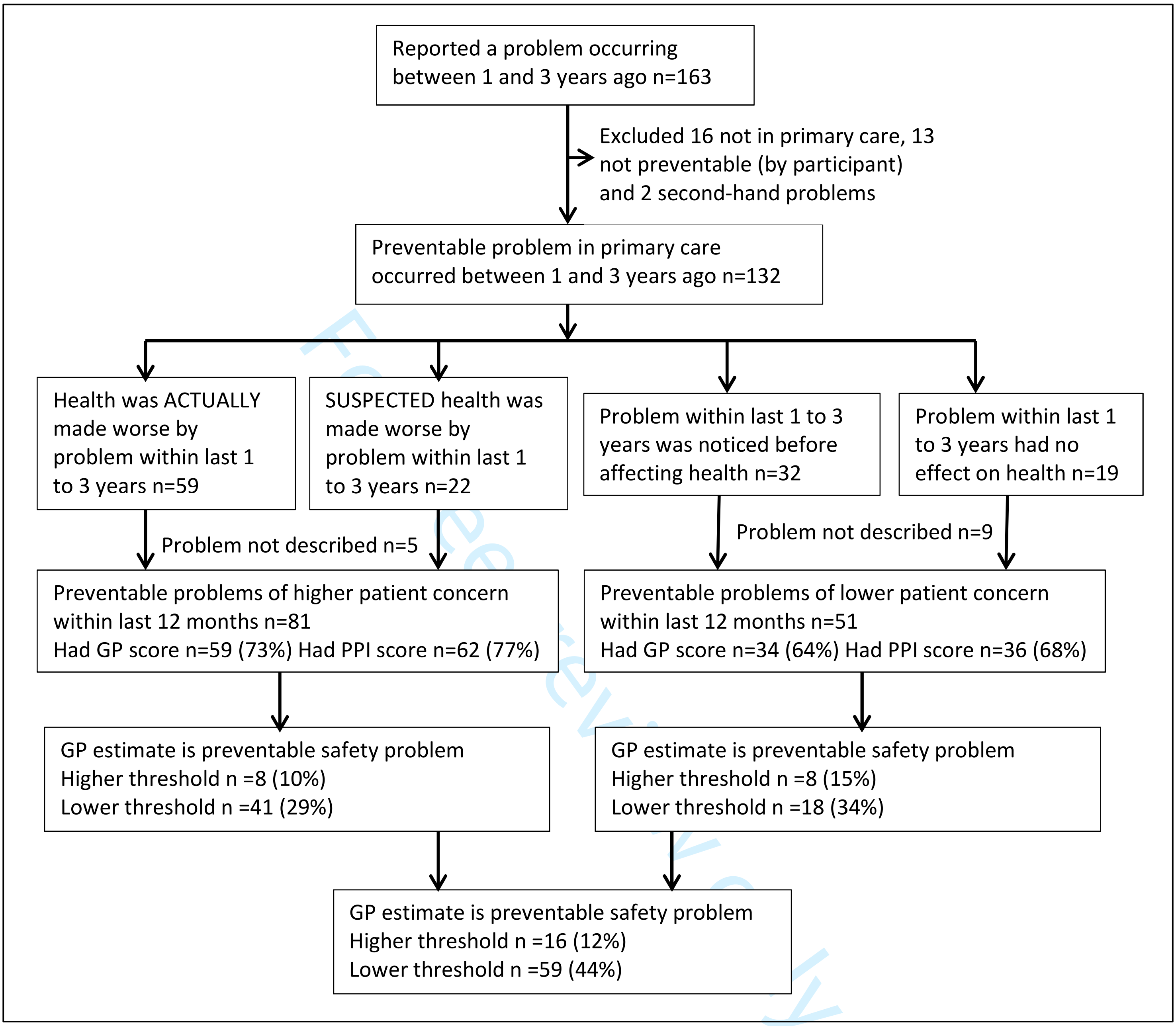

Fig B. Flow chart of participants reporting a potential-harmful preventable-problem within the last 1 to 3 years

For peer review only - http://bmjopen.bmj.com/site/about/guidelines.xhtml

Table D. Demographics of responders to Ipsos MORI GB Face to Face Omnibus April 2016

	Number of participants (%) n=3984	Population level estimates for comparison	Population comparator source; P(χ^2)= probability survey population differs from population comparator
**Confidence and trust in GP at last appointment?**			
Yes definitely	3031 (76%)	523498 (63%)	GP patient survey in England mid-2015(25) P(χ^2)<0.0001
Yes, to some extent	611 (15%)	235760 (29%)	
No, not at all	311 (8%)	37743 (5%)	
Don't know /can't say	31 (1%)	28866 (3%)	
**Gender (1 missing)**			
Male	1950 (49%)	32074400 (49%)	ONS mid-2015 estimates[1] P(χ^2)=0.7
Female	2033 (51%)	33035600 (51%)	
**Age**			
15 to 24	533 (13%)	8118600 (15%)	ONS mid-2015 estimates[1] P(χ^2)<0.0001
25 to 34	573 (14%)	8822700 (16%)	
35 to 44	528 (13%)	8378300 (16%)	
45 to 54	629 (16%)	9196000 (17%)	
55 to 64	654 (16%)	7452400 (13%)	
65 to 74	609 (15%)	6339800 (11%)	
75 or older	458 (12%)	5271400 (10%)	
**Ethnicity (18 missing)**			
White	3491 (88%)	48209395 (86%)	England & Wales census (2011)[2] P(χ^2)<0.0001
Other ethnicity	475 (12%)	7866517 (14%)	
**Social Grade[3]**			
A/B	1054 (26%)	8081619 (23%)	England & Wales census (2011)[2] P(χ^2)<0.0001
C1	1122 (28%)	10796044 (30%)	
C2	771 (19%)	7865976 (22%)	
D/E	1037 (26%)	8903873 (25%)	

[1]https://www.ons.gov.uk/peoplepopulationandcommunity/populationandmigration/populationestimates/bulletins/annualmidyearpopulationestimates/latest

[2]https://www.ons.gov.uk/peoplepopulationandcommunity/populationandmigration/populationestimates/bulletins/keystatisticsandquickstatisticsforlocalauthoritiesintheunitedkingdom/2013-10-11

[3]A/B High or intermediate managerial, professional or administrative, C1 Supervisory, clerical and junior managerial, professional or administrative, C2 skilled manual workers, D/E semi and unskilled manual workers, casual or lowest grade workers, state pensioners, unemployed with state benefits only

For peer review only - http://bmjopen.bmj.com/site/about/guidelines.xhtml

For peer review only

Table E. Categorisation of patient-described scenarios according to clinician ranking as to the likelihood they represent a potentially-harmful preventable-problem

Group	Scores on a 5 point scale of "very likely or certain", "probably", "possibly", "unlikely", "definitely not" (see table C, online Appendix 2)	Unprompted problems (answered "yes" to Q2, Box1)				All problems within past 12 months (answered "yes" to Q2or Q10, Box1) n=300	
		Within past 12 months n=193		Within past 3 years n=325			
		Clinicians	Members of the Public	Clinicians	Members of the Public	Clinicians	Members of the Public
1. Higher threshold	Median score higher than "probably" or at least one score of "very likely or certain"	16 (8%)	91 (47%)	28 (9%)	165 (51%)	24 (8%)	116 (39%)
2. Lower threshold	Median score higher than "possibly" or at least one score of "probably" or higher	67 (35%)	145 (75%)	124 (38%)	237 (73%)	97 (32%)	198 (66%)
3. Any possibility	At least one score of "unlikely" or higher	141 (73%)	157 (81%)	232 (71%)	254 (78%)	194 (65%)	221 (74%)
4. No problem	All scores "definitely not" (or not-coded)	8 (4%)	0	9 (3%)	0	13 (4%)	0
5. Not-coded	Insufficient information for coding by all raters	44 (23%)	36 (19%)	84 (26%)	71 (22%)	93 (31%)	79 (26%)

For peer review only - http://bmjopen.bmj.com/site/about/guidelines.xhtml

Table F. Survey responses and respondent characteristics as predictors of clinician and members of the public estimates of the likelihood that the scenario describes a potentially-harmful preventable problem

Respondent characteristics (total) n=406 (ranked by at least one clinician)	Clinician – lower threshold[1] (n=224, 55%)		Members of the public – higher threshold[2] (n=267, 66%)	
	Frequency (%)	Adjusted odds ratio	Frequency (%)	Adjusted odds ratio
**Source of respondent (0 missing)**				
Ipsos MORI f2f Omnibus (299)	153 (51%)	1 (ref)	182 (61%)	1 (ref)
Pilot survey (107)	71 (66%)	1.5 (0.9 to 2.7)	85 (79%)	5.2 (2.5 to 10.8)
**Gender (3 missing)**				
Male (150)	79 (53%)	1 (ref)	93 (62%)	1 (ref)
Female (253)	142 (56%)	1.2 (0.8 to 1.9)	172 (68%)	1.5 (0.9 to 2.4)
**Age (3 missing)**				
15 to 24 years (46)	21 (46%)	1 (ref)	28 (61%)	1 (ref)
25 to 34 years (60)	34 (57%)	1.5 (0.7 to 3.5)	43 (72%)	1.4 (0.6 to 3.7)
35 to 44 years (38)	24 (63%)	1.8 (0.7 to 4.5)	30 (79%)	1.9 (0.6 to 5.6)
45 to 54 years (74)	44 (59%)	1.5 (0.7 to 3.4)	50 (68%)	1.1 (0.5 to 2.7)
55 to 64 years (82)	45 (55%)	1.4 (0.6 to 3.2)	50 (61%)	1.0 (0.4 to 2.3)
65 to 74 years (75)	39 (52%)	1.2 (0.5 to 2.8)	49 (65%)	1.1 (0.4 to 2.6)
75 years or older (28)	14 (50%)	1.1 (0.4 to 3.2)	15 (54%)	0.6 (0.2 to 1.8)
**Patient estimate of impact of the problem on their health (0 missing)**				
Actually or suspected made health worse (192)	109 (57%)	1 (ref)	139 (73%)	1 (ref)
Noticed before made health worse or had no effect on health (106)	58 (55%)	0.8 (0.5 to 1.4)	69 (65%)	0.6 (0.3 to 1.1)
Prompted by Q10 (108)	57 (53%)	0.7 (0.4 to 1.2)	59 (55%)	0.3 (0.1 to 0.5)
**Patient is qualified as a healthcare professional or volunteers in healthcare research[2] (0 missing)**				
No (339)	177 (52%)	1 (ref)	221 (65%)	1 (ref)
Yes (67)	47 (70%)	2.0 (1.1 to 3.8)	46 (69%)	0.8 (0.4 to 1.7)
**Discussed the problem with somebody working in the primary care service (0 missing)**				
No/don't know/missing (197)	99 (50%)	1 (ref)	119 (60%)	1 (ref)
Yes (209)	125 (60%)	1.3 (0.9 to 2.0)	148 (71%)	1.5 (0.9 to 2.4)
**Service used (1 missing)**				
GP surgery (286)	159 (56%)	1 (ref)	186 (65%)	1 (ref)
Dental surgery (36)	17 (46%)	0.8 (0.3 to 1.7)	12 (33%)	1.1 (0.5 to 2.7)
Walk in clinic (16)	7 (44%)	1.0 (0.4 to 3.0)	10 (63%)	1.7 (0.5 to 5.7)
Ambulance/A&E/ OOH (20)	13 (65%)	2.0 (0.7 to 5.5)	15 (75%)	3.8 (1.0 to 14.1)
Pharmacy (18)	15 (83%)	2.0 (0.5 to 7.8)	3 (17%)	1.0 (0.2 to 4.3)
Other (29)	12 (41%)	0.7 (0.3 to 1.7)	14 (48%)	1.4 (0.6 to 3.4)
**Problem related to (0 missing)**				
A. Healthcare delivery system (65)	25 (38%)	1 (ref)	24 (37%)	1 (ref)
B. Investigation (63)	29 (46%)	1.2 (0.6 to 2.5)	42 (67%)	3.4 (1.5 to 7.6)
C. Treatment process (100)	73 (73%)	3.7 (1.8 to 7.7)	85 (85%)	11.0 (4.6 to 26.5)
D. Communication (66)	36 (55%)	1.8 (0.9 to 3.7)	37 (56%)	2.0 (0.9 to 4.2)
E. Clinical knowledge or skills (43)	23 (53%)	1.8 (0.8 to 4.2)	30 (70%)	3.3 (1.3 to 8.4)
F. Diagnosis (56)	34 (61%)	2.5 (1.1 to 5.4)	79 (21%)	6.2 (2.6 to 15.1)
G. Wrong treatment decision (4)	2 (50%)	1.4 (0.2 to 11.5)	3 (75%)	3.9 (0.4 to 41.7)

For peer review only - http://bmjopen.bmj.com/site/about/guidelines.xhtml

H. Other (9)	2 (22%)	0.5 (0.1 to 2.8)	2 (22%)	0.4 (0.1 to 2.2)
**Relevant condition (0 missing)**	**Frequency (%)**	**Unadjusted odds ratio[3]**	**Frequency (%)**	**Unadjusted odds ratio[3]**
All other conditions (47)	24 (51%)	1 (ref)	29 (19%)	1 (ref)
Cardiovascular (8)	7 (88%)	6.7 (0.8 to 58.9)	8 (100%)	-[4]
Diabetes (32)	20 (63%)	1.6 (0.6 to 4.0)	24 (75%)	1.8 (0.7 to 5.0)
Cancer (7)	7 (100%)	-[4]	7 (100%)	-[4]
Mental health (18)	6 (33%)	0.5 (0.2 to 1.5)	15 (83%)	3.1 (0.8 to 12.2)
Dental (33)	16 (48%)	0.9 (0.4 to 2.2)	24 (73%)	1.7 (0.6 to 4.3)
Accidental injury (17)	10 (59%)	1.4 (0.4 to 4.2)	12 (71%)	1.5 (0.4 to 4.9)
Infectious (12)	8 (67%)	1.9 (0.5 to 7.2)	10 (83%)	3.1 (0.6 to 15.8)
Pain/discomfort (15)	8 (53%)	1.1 (0.3 to 3.5)	5 (30%)	0.3 (0.1 to 1.1)
Skin (12)	5 (42%)	0.7 (0.2 to 2.5)	4 (33%)	0.3 (0.1 to 1.2)
Respiratory (13)	9 (69%)	2.2 (0.6 to 8.0)	12 (92%)	7.4 (0.9 to 62.2)
Pregnancy (8)	6 (75%)	2.9 (0.5 to 15.7)	8 (100%)	-[4]
Musculoskeletal (34)	11 (32%)	0.5 (0.2 to 1.1)	16 (47%)	0.6 (0.2 to 1.3)
Ear, nose and throat (9)	6 (67%)	1.9 (0.4 to 8.6)	4 (44%)	0.5 (0.1 to 2.1)
Not relevant/not known (141)	81 (57%)	1.3 (0.7 to 2.5)	89 (63%)	1.1 (0.5 to 2.1)

[1]median score higher than “probably” or at least one score of “very likely or certain”, see Table B

[2]median score higher than “possibly” or at least one score of “probably” or higher, see Table B

[3]unadjusted OR shown due to collinearity between dental problems and dental service

[4]predicts success perfectly (100% of scenarios in this category)

For peer review only

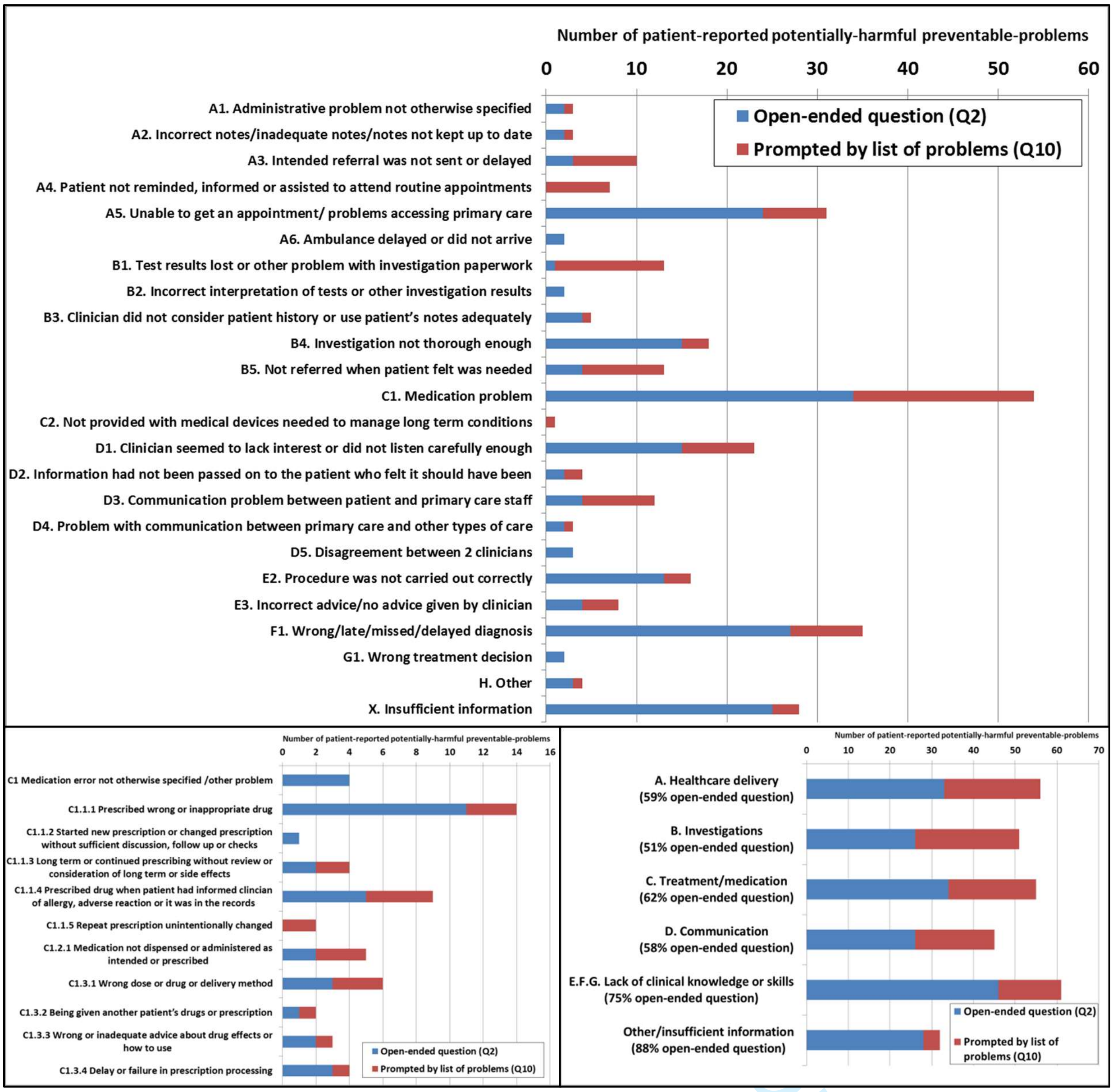

Fig C. Numbers of patient-perceived problems occurring in the last 12 months categorised according to the patient's description (see Table 2) and route through survey *i.e.* originated from open-ended question (Q2) or prompted by list of potential safety problems (Q10). See online Appendix 2 for details of coding; A coded to 2 levels, B medication problems coded to 3 levels, C coded to 1 level

For peer review only - http://bmjopen.bmj.com/site/about/guidelines.xhtml

Appendix 2. SJ Stocks et al. BMJ Open 2018: The frequency and nature of potentially-harmful preventable-problems in primary care from the patient's perspective with clinician review – a population level survey in Great Britain Patient reported scenarios occurring during the past 12 months that clinicians scored as higher likelihood to be a potentially-unsafe preventable-problem in primary care (median score is higher than "possibly" and at least 2 clinicians gave a score or one clinician scored "very likely or certain"). PPI = member of the public, GP = primary care clinician

Scenario1. Ambulance

Briefly describe the mistake or problem and how it happened. *"Heart attack, an ambulance was called and waited an hour and three quarters to arrive"*

Could the mistake or problem have been avoided? If so how? "*The ambulance service needs to be sorted out"*

Were you able to talk about the mistake or problem with anybody working in the primary care service? *"No I was too distressed to discuss the problem or error"*

Patient-reported prospect of harm: health could have been made worse had someone not noticed a problem or error

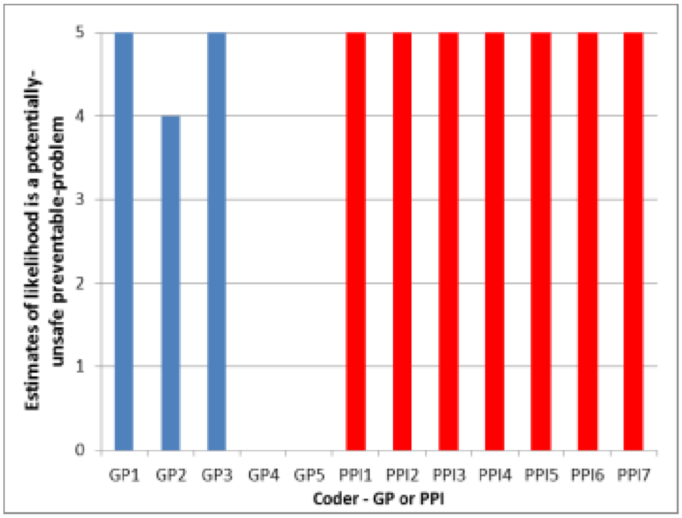

5=very likely or certain, 4=probably, 3=possibly, 2=unlikely, 1=definitely not, 0 = insufficient information or don't know

Patient-perspective problem-type code: A6. Ambulance delayed or did not arrive

Scenario2. GP surgery

Briefly describe the mistake or problem and how it happened. *"I had an ongoing stomach complaint. The GP kept prescribing a steroid treatment but the pharmacist refused to give it to me. He said it was dangerous and I had to get different medication. The GP prescribed an alternative but the pharmacist pointed out that the steroid was supposed to be a short term treatment and that the GP had been prescribing it for over a year."*

Could the mistake or problem have been avoided? If so how? "*The GP obviously didn't read the notes. The GP was probably pushed for time and just wanted to get me out (maybe?)"*

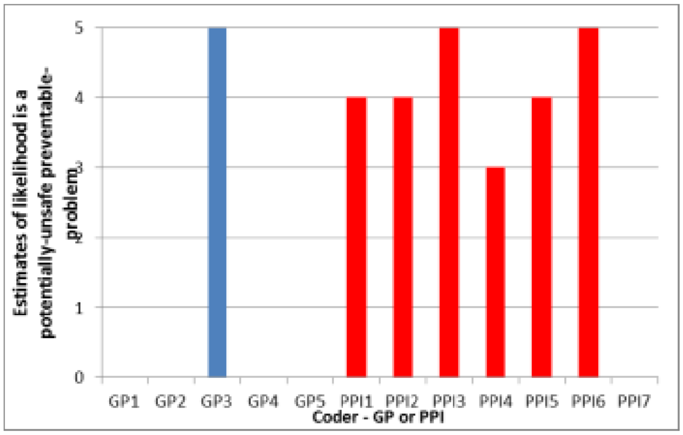

5=very likely or certain, 4=probably, 3=possibly, 2=unlikely, 1=definitely not, 0 = insufficient information or don't know

Were you able to talk about the mistake or problem with anybody working in the primary care service? *"No I was not concerned about the problem or error"*

Patient-reported prospect of harm: prompted via Q10 (Box 1 main paper)

Patient-perspective problem-type code: C1.1.3 Long term or continued prescribing without review or consideration of long term or side effects; B3 Clinician did not consider patient history sufficiently/did not use patient's notes adequately

Scenario3. GP surgery

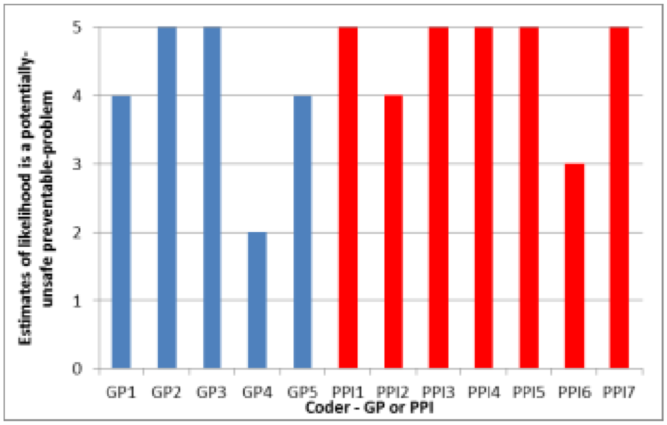

5=very likely or certain, 4=probably, 3=possibly, 2=unlikely, 1=definitely not, 0 = insufficient information or don't know

Briefly describe the mistake or problem and how it happened. *"Participant was prescribed penicillin and it was stated in notes that patient was allergic to penicillin"*

Could the mistake or problem have been avoided? If so how? *"It was avoided as participant didn't take prescription and was prescribed something else"*

Were you able to talk about the mistake or problem with anybody working in the primary care service? *"Yes with GP"*

Patient-reported prospect of harm: health could have been made worse had someone not noticed a problem or error

Patient-perspective problem-type code: C1.1.4 Prescribed drug when should have known contra-indicated *e.g.* patient had informed clinician of allergy, adverse reaction or it was in the records

Scenario4. Optician

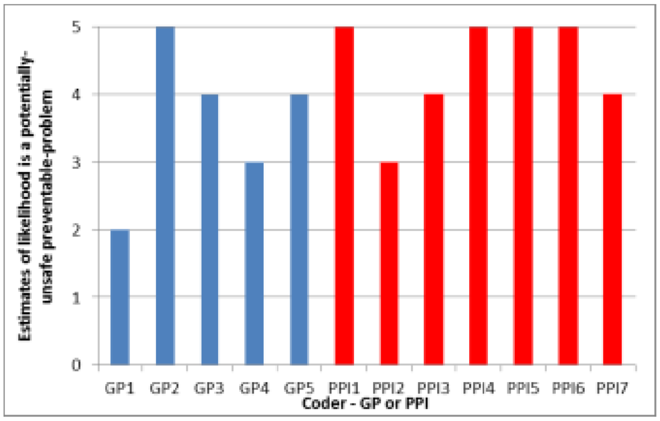

5=very likely or certain, 4=probably, 3=possibly, 2=unlikely, 1=definitely not, 0 = insufficient information or don't know

Briefly describe the mistake or problem and how it happened. *"Started suffered blurred vision in left eye, eye was bloodshot. Went to get eye check and was sold eye drops to treat infection, told would take five days. After five days of treatment problem was made worse until vision was affected, GP referred to eye clinic diagnosed with iritis. Further treatment at eye clinic cleared up the issue."*

Could the mistake or problem have been avoided? If so how? *"If optometrists had spotted that iris was stuck, had a bit more professional care rather than trying to flog over-the-counter eye drops to clear up infection that wasn't there"*

Were you able to talk about the mistake or problem with anybody working in the primary care service? *"Yes, spoke to GP, immediate referral to eye clinic for treatment"*

Patient-reported prospect of harm: health was actually made worse by a problem or error that could have been prevented

Patient-perspective problem-type code: F1 Wrong/late/missed/delayed diagnosis

Scenario5. GP surgery

Briefly describe the mistake or problem and how it happened. *"Contra-indication with a medicine that was not noticed at time of prescription but was noticed by the participant before they started taking the medicine"*

Could the mistake or problem have been avoided? If so how? "*The contra-indication should have been flagged up on the computer at the time of prescription but it wasn't"*

Were you able to talk about the mistake or problem with anybody working in the primary care service? "Yes, *secretary and a GP"*

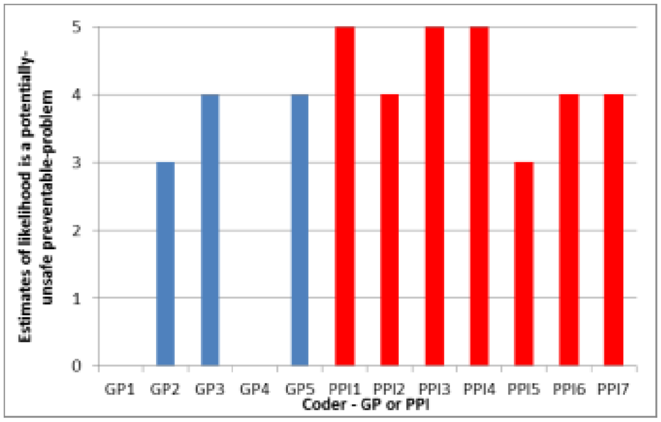

5=very likely or certain, 4=probably, 3=possibly, 2=unlikely, 1=definitely not, 0 = insufficient information or don't know

Patient-reported prospect of harm: health could have been made worse had someone not noticed a problem or error

Patient-perspective problem-type code: C1.1.4 Prescribed drug when should have known contra-indicated *e.g.* patient had informed clinician of allergy, adverse reaction or it was in the records

Scenario6. GP surgery

Briefly describe the mistake or problem and how it happened. *"Went with a lump to GP. He referred me under the 2 week NICE guidelines. The communication went wrong and I chased it up myself or would have remained sat here. I ended up being diagnosed with cancer but I intervened in time."*

Could the mistake or problem have been avoided? If so how? *"Policies & procedures in place now. If you're sent an appointment that place needs to send a confirmation. That's what happened to stop it happening again."*

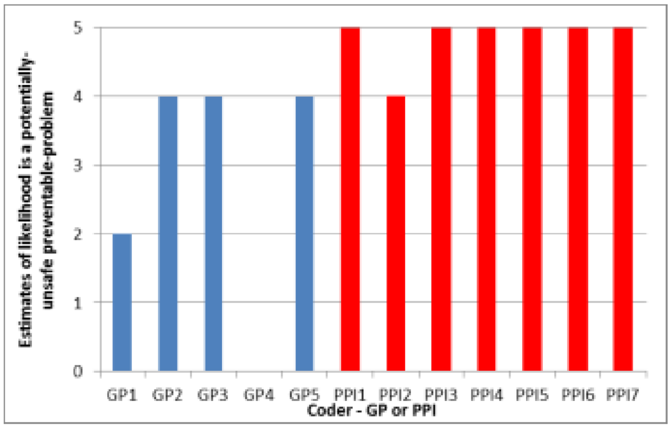

5=very likely or certain, 4=probably, 3=possibly, 2=unlikely, 1=definitely not, 0 = insufficient information or don't know

Were you able to talk about the mistake or problem with anybody working in the primary care service? *"GP investigated it as a significant event. Said if not satisfied come in and chat to us. I had apology from GP."*

Patient-reported prospect of harm: health could have been made worse had someone not noticed a problem or error

Patient-perspective problem-type code: A3. Intended referral was not sent or delayed

Scenario7. Pharmacy

Briefly describe the mistake or problem and how it happened. *"They gave me the wrong tablets and they were heart pills - beta blockers- but I thought they were sleeping pills. I looked at the patient information and thought why am I not sleeping and realised they were for people who had had a heart attack. I was taking them for 6 weeks then I phoned the doctor and he came straight away. The pharmacist no longer works there."*

Could the mistake or problem have been avoided? If so how? *"She just put up the wrong tablets. She should have dispensed the right pills as on my prescription"*

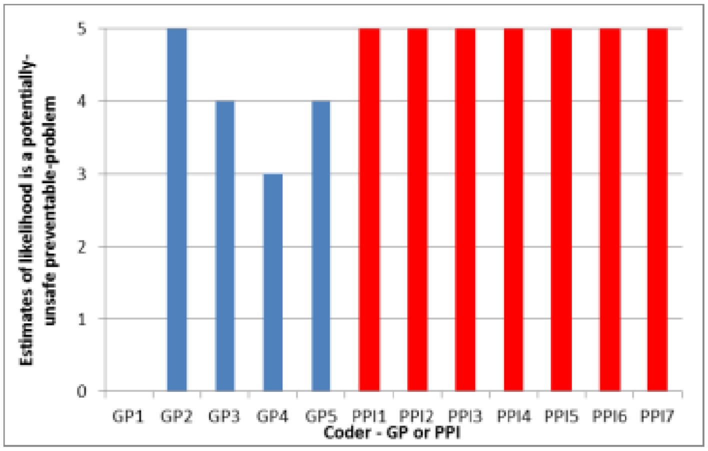

5=very likely or certain, 4=probably, 3=possibly, 2=unlikely, 1=definitely not, 0 = insufficient information or don't know

Were you able to talk about the mistake or problem with anybody working in the primary care service? *"Yes, doctor - he gave me the right ones"*

Patient-reported prospect of harm: health could have been made worse had someone not noticed a problem or error

Patient-perspective problem-type code: C1.2.1 Medication not dispensed or administered as intended or prescribed

Scenario8. Out of hours care

Briefly describe the mistake or problem and how it happened. *"Banged foot at work, hurt a lot, for few days got worse"*

Could the mistake or problem have been avoided? If so how? *"if they had listened to me properly, they didn't therefore toe got amputated for no reason"*

Were you able to talk about the mistake or problem with anybody working in the primary care service? *"Yes, triage nurse"*

Patient-reported prospect of harm: health could have been made worse had someone not noticed a problem or error

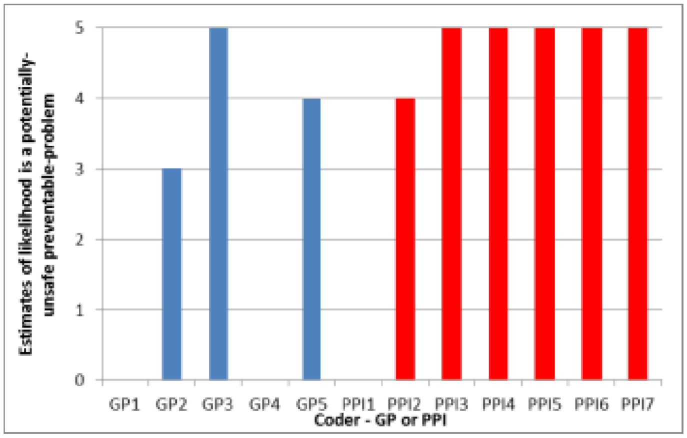

5=very likely or certain, 4=probably, 3=possibly, 2=unlikely, 1=definitely not, 0 = insufficient information or don't know

Patient-perspective problem-type code: B4. Investigation not thorough enough; D1. Clinician seemed to lack interest in the patient's health problem or did not listen carefully enough

Scenario9. GP surgery

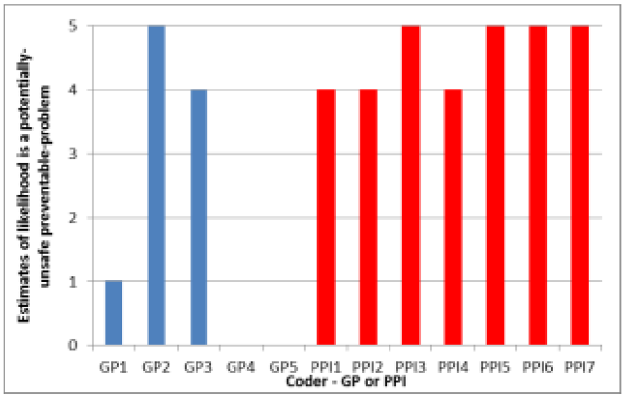

5=very likely or certain, 4=probably, 3=possibly, 2=unlikely, 1=definitely not, 0 = insufficient information or don't know

Briefly describe the mistake or problem and how it happened. *I was started on warfarin and was fainting and bleeding rectally. I was in town the first time I passed out and did not go to hospital. The second time I went to hospital and the problem was rectified by reducing the dose."*

Could the mistake or problem have been avoided? If so how? *"by giving a smaller dose in the first place. I was told that the amount was too much. Afterwards they put me on something else instead of warfarin."*

Were you able to talk about the mistake or problem with anybody working in the primary care service? *"Yes, doctor in hospital"*

Patient-reported prospect of harm: health was actually made worse by a problem or error that could have been prevented

Patient-perspective problem-type code: C1.3.1 Wrong dose or drug or delivery method

Scenario10. GP surgery

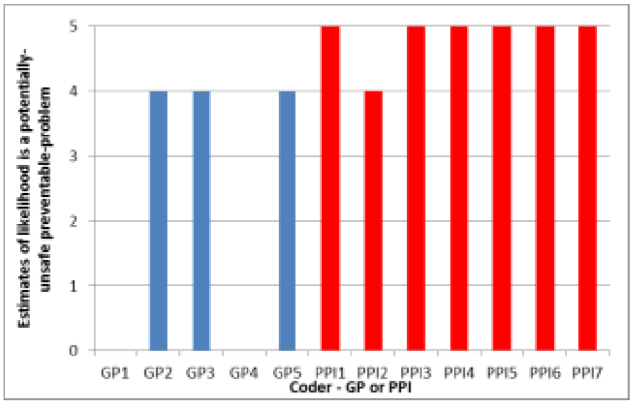

5=very likely or certain, 4=probably, 3=possibly, 2=unlikely, 1=definitely not, 0 = insufficient information or don't know

Briefly describe the mistake or problem and how it happened. *"Couldn't get appointment at GP. Health worsened, ended up in hospital with fluid on lungs and pneumonia. Was rushed in. Heart had to be stopped and restarted."*

Could the mistake or problem have been avoided? If so how? *"Had rung for appointments and asked for doctor to telephone me 3 times. They never rang. They should have signed my prescriptions so I could have medicine and should have seen me in person"*

Were you able to talk about the mistake or problem with anybody working in the primary care service? *"The heart nurse from the community service complained on my behalf to the GP surgery. The chemist shop complained too about prescriptions not being signed and medicine being missed. Appointment was made at surgery to discuss with new doctor, and appointments are guaranteed as now a "supported patient"."*

Patient-reported prospect of harm: health was actually made worse by a problem or error that could have been prevented

Patient-perspective problem-type code: A5. Unable to get an appointment/other problems with making appointment; C1.3.4 Delay or failure in prescription processing

Scenario11. Dental surgery

Briefly describe the mistake or problem and how it happened. *"Dentist numbed me up to pull a wrong tooth"*

Could the mistake or problem have been avoided? If so how? *"By taking care by paying attention to his own notes"*

Were you able to talk about the mistake or problem with anybody working in the primary care service? *"Yes, the dentist himself - he was apologetic."*

Patient-reported prospect of harm: a problem or error that could have been prevented but it did not make your health worse

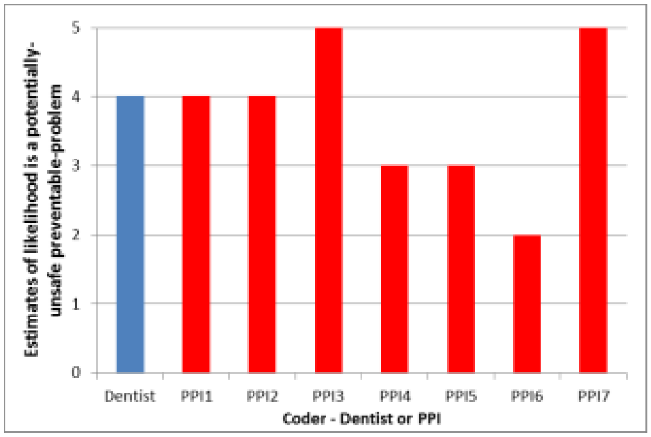

5=very likely or certain, 4=probably, 3=possibly, 2=unlikely, 1=definitely not, 0 = insufficient information or don't know

Patient-perspective problem-type code: E2.Procedure was not carried out correctly

Scenario12. GP surgery

Briefly describe the mistake or problem and how it happened. *"Discharged from hospital following knee replacement surgery, became very ill, lost 1 stone in 7 days, requested home visit from GP as seriously concerned, doctor called by phone and was very brusque, no home visit but medication changed and 6 months later started to feel better"*

Could the mistake or problem have been avoided? If so how? *"if the doctor had come to see me in person who could have made a quicker diagnosis and could have offered some much needed support during a very traumatic time"*

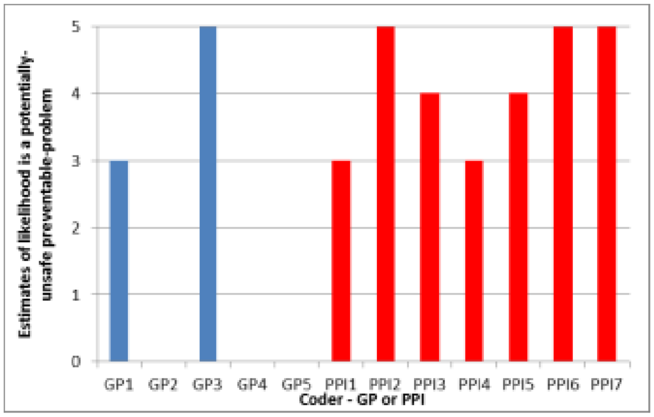

5=very likely or certain, 4=probably, 3=possibly, 2=unlikely, 1=definitely not, 0 = insufficient information or don't know

Were you able to talk about the mistake or problem with anybody working in the primary care service? *"No, I could not find anybody with whom I could discuss the problem or error"*

Patient-reported prospect of harm: suspected your health has been made worse by a problem or error that could have been prevented

Patient-perspective problem-type code: D1. Clinician seemed to lack interest in the patient's health problem or did not listen carefully enough

Scenario13. Pharmacy

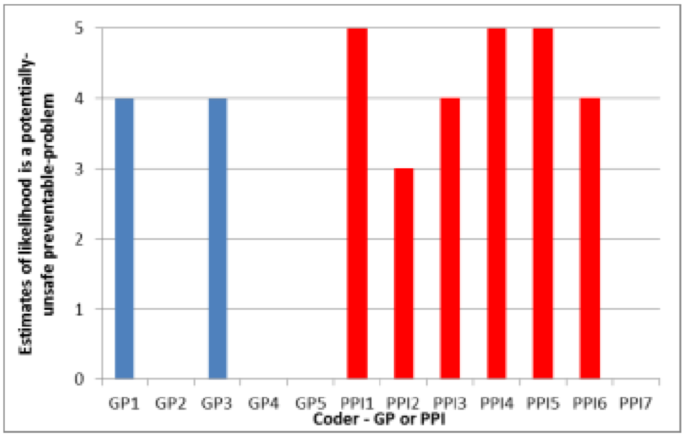

5=very likely or certain, 4=probably, 3=possibly, 2=unlikely, 1=definitely not, 0 = insufficient information or don't know

Briefly describe the mistake or problem and how it happened. *"I use a certain inhaler for COPD. I had run out without realising that I had forgotten to tick it on my repeat prescription. I spoke to the pharmacist and explained to ask him to add it for next time I picked up the repeat prescription. They agreed to do this but when I went to collect it I found that they had ordered a different medicine unrelated to COPD. I was upset because in the meantime my COPD had worsened quite quickly and was causing me distress."*

Could the mistake or problem have been avoided? If so how? *"The chemist should have made a note at the time and written down the medicine that I was asking for. If they had taken the note there and then I don't think this would have happened. I'm assuming he took a note later and failed to remember the name of the medicine correctly. We have a dreadful chemist service here."*

Were you able to talk about the mistake or problem with anybody working in the primary care service? *"No, I was so exasperated I went to my GP to order the medicine directly"*

Patient-reported prospect of harm: prompted via Q10 (Box 1 main paper)

Patient-perspective problem-type code: C1.1.5 Repeat prescription unintentionally changed

Scenario14. GP surgery

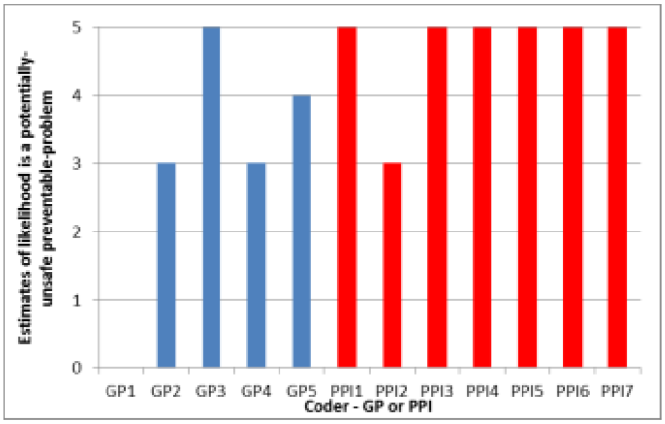

5=very likely or certain, 4=probably, 3=possibly, 2=unlikely, 1=definitely not, 0 = insufficient information or don't know

Briefly describe the mistake or problem and how it happened. *"GP misdiagnosed broken jaw, went to emergency dentist then to A&E where it was operated on and fixed"*

Could the mistake or problem have been avoided? If so how? "*if GP had diagnosed correctly initially"*

Were you able to talk about the mistake or problem with anybody working in the primary care service? *"made complaint to surgery and they wrote back apologising"*

Patient-reported prospect of harm: health was actually made worse by a problem or error that could have been prevented

Patient-perspective problem-type code: F1. Wrong/late/missed/delayed diagnosis

Scenario15. GP surgery

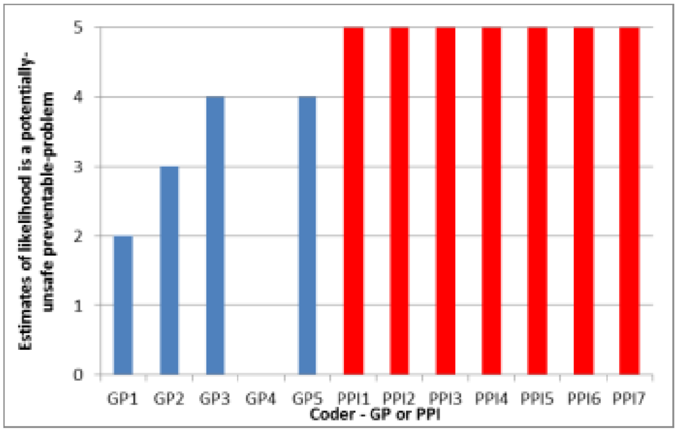

5=very likely or certain, 4=probably, 3=possibly, 2=unlikely, 1=definitely not, 0 = insufficient information or don't know

Briefly describe the mistake or problem and how it happened. *"I was having severe nose bleeds for several months and was told it was hay fever. It was cancer."*

Could the mistake or problem have been avoided? If so how? *"My GP could have sent me for a CT scan as soon as my nose bleeds started."*

Were you able to talk about the mistake or problem with anybody working in the primary care service? *"Yes, I registered with a new GP who sent me for a scan straight away which identified my cancer."*

Patient-reported prospect of harm: health was actually made worse by a problem or error that could have been prevented

Patient-perspective problem-type code: F1. Wrong/late/missed/delayed diagnosis

Scenario16. GP surgery

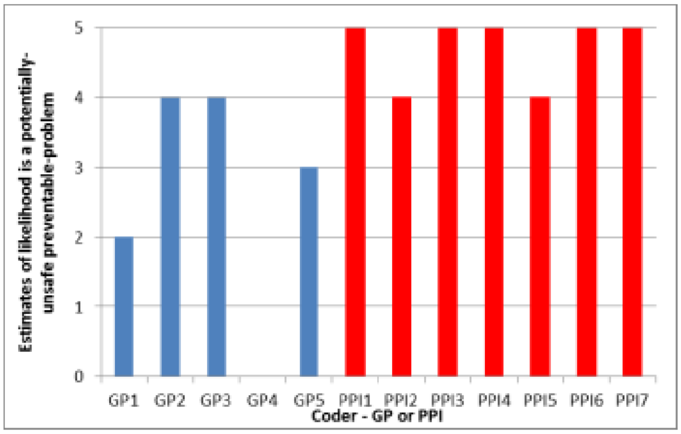

5=very likely or certain, 4=probably, 3=possibly, 2=unlikely, 1=definitely not, 0 = insufficient information or don't know

Briefly describe the mistake or problem and how it happened. *"Doctor prescribed tramadol without checking my notes. I'd already taken four pills and I rang up general enquiries at GP service to say I felt disorientated almost as if it was happening to someone else and not me. Got through to my main doctor and asked whether it was wise to take more, she said don't because you might not be alive if you do. She could see I had the wrong dose, disorientation carried on for a couple of days. It was the wrong medication."*

Could the mistake or problem have been avoided? If so how? *"if he had checked my notes to see what I can and can't take in terms of the actual medication"*

Were you able to talk about the mistake or problem with anybody working in the primary care service? *"discussed it with main doctor who said that she would give me some different pills to take to ease the pain for my trapped nerve in spine and back. She said she would speak to other doctor to see why it happened"*

Patient-reported prospect of harm: health was actually made worse by a problem or error that could have been prevented

Patient-perspective problem-type code: C1.3.1 Wrong dose or drug or delivery method

Scenario17. Out of hours care

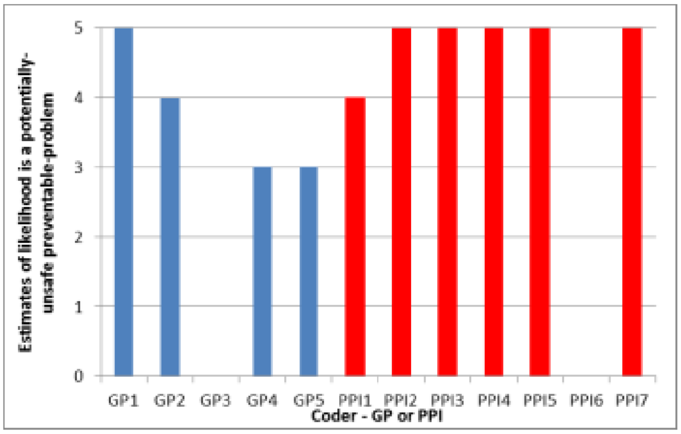

5=very likely or certain, 4=probably, 3=possibly, 2=unlikely, 1=definitely not, 0 = insufficient information or don't know

Briefly describe the mistake or problem and how it happened. *"Threatened miscarriage. Not given anti-D injection and notes were not consulted" (rhesus-negative patient)*

Could the mistake or problem have been avoided? If so how? *"Notes should have been checked"*

Were you able to talk about the mistake or problem with anybody working in the primary care service? *"Yes, hospital consultant who dealt effectively with situation"*

Patient-reported prospect of harm: there was a problem or error that could have been prevented but it did not make your health worse

Patient-perspective problem-type code: B3 Clinician did not consider patient history sufficiently/did not use patient's notes adequately

Scenario18. GP surgery

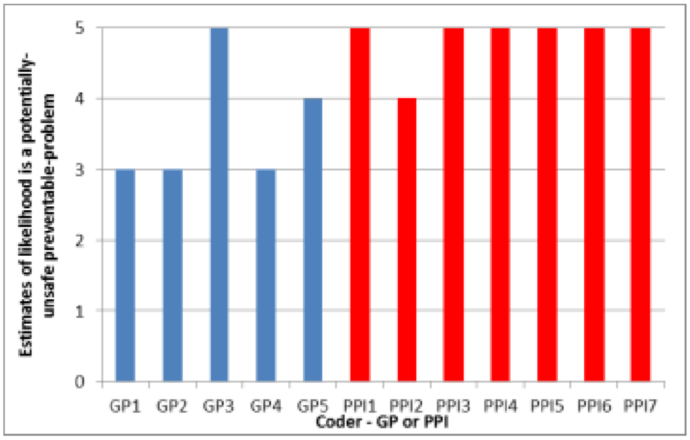

5=very likely or certain, 4=probably, 3=possibly, 2=unlikely, 1=definitely not, 0 = insufficient information or don't know

Briefly describe the mistake or problem and how it happened. *"Had retained placenta 4 weeks after giving birth. GP dismissed it and went to A&E. Had emergency surgery"*

Could the mistake or problem have been avoided? If so how? "Yes, *by improving GP competence levels"*

Were you able to talk about the mistake or problem with anybody working in the primary care service? *"No, I was too distressed to discuss the problem or error"*

Patient-reported prospect of harm: there was a problem or error that could have been prevented but it did not make your health worse

Patient-perspective problem-type code: F1. Wrong/late/missed/delayed diagnosis

Scenario19. GP surgery

Briefly describe the mistake or problem and how it happened. *"I had a mole on my arm. It started to itch. I asked the GP if he'd look at it. He said it's fine. Two weeks later I had to see a dermatologist for a different reason. I asked him to look at the mole. He examined it through a magnifying glass. He said he couldn't tell if it was cancerous but recommended me to the local hospital. Two weeks later the hospital informed me the mole was cancerous. They took the mole out immediately. The point is that my GP didn't identify the possible cancer, it was coincidence that I went to the dermatologist who happened to be treating me at the time for a dry skin problem."*

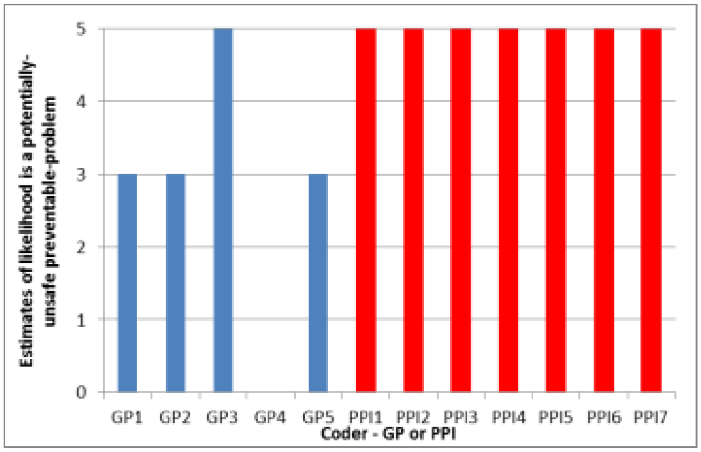

5=very likely or certain, 4=probably, 3=possibly, 2=unlikely, 1=definitely not, 0 = insufficient information or don't know

Could the mistake or problem have been avoided? If so how? *"My GP could have examined me properly rather than just looking at the mole or he could have recommended a specialist if he didn't know what it was"*

Were you able to talk about the mistake or problem with anybody working in the primary care service? *"No, I wasn't confident that they would listen/I felt anything I say would fall on deaf ears"*

Patient-reported prospect of harm: health was actually made worse by a problem or error that could have been prevented

Patient-perspective problem-type code: F1. Wrong/late/missed/delayed diagnosis

Scenario20. GP surgery

Briefly describe the mistake or problem and how it happened. *"appendix problem not diagnosed"*

Could the mistake or problem have been avoided? If so how? *"better diagnostic skills"*

Were you able to talk about the mistake or problem with anybody working in the primary care service? *"Yes, another GP who referred me to hospital"*

Patient-reported prospect of harm: health could have been made worse had someone not noticed a problem or error

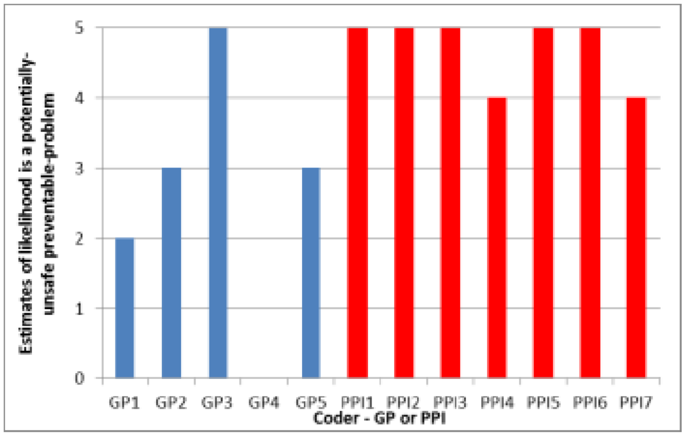

5=very likely or certain, 4=probably, 3=possibly, 2=unlikely, 1=definitely not, 0 = insufficient information or don't know

Patient-perspective problem-type code: F1. Wrong/late/missed/delayed diagnosis

Scenario21. GP surgery

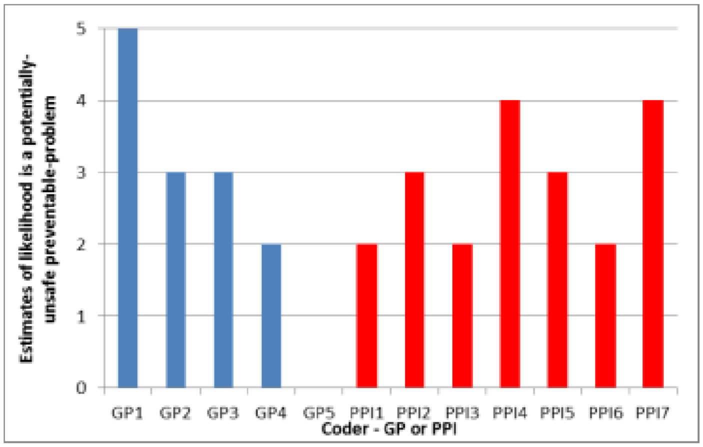

5=very likely or certain, 4=probably, 3=possibly, 2=unlikely, 1=definitely not, 0 = insufficient information or don't know

Briefly describe the mistake or problem and how it happened. *"I had something stuck into my ear, a cotton bud. I went to GP and they booked an appointment with a consultant. After 6 months I didn't hear anything from him. Luckily the cotton bud came out by itself, it could have been worse."*

Could the mistake or problem have been avoided? If so how? *"If I could have an appointment with a consultant he could have checked my ear canal"*

Were you able to talk about the mistake or problem with anybody working in the primary care service? *"No, I could not find anybody with whom I could discuss the problem or error"*

Patient-reported prospect of harm: health could have been made worse had someone not noticed a problem or error

Patient-perspective problem-type code: A3. Intended referral was not sent or delayed

Scenario22. A&E

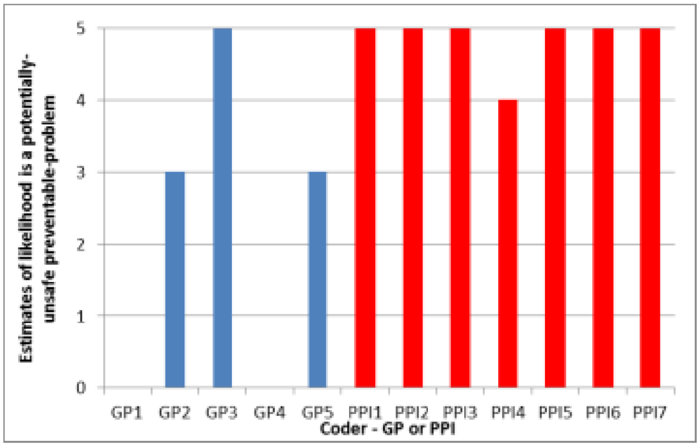

5=very likely or certain, 4=probably, 3=possibly, 2=unlikely, 1=definitely not, 0 = insufficient information or don't know

Briefly describe the mistake or problem and how it happened. *"Basically told me problem was biliary spasms / colic but it was actually a hole in my stomach"*

Could the mistake or problem have been avoided? If so how? "If the doctor had taken heed of blood results - he ignored blood results - ended in emergency surgery"

Were you able to talk about the mistake or problem with anybody working in the primary care service? *"No, I was too distressed to discuss the problem or error"*

Patient-reported prospect of harm: health was actually made worse by a problem or error that could have been prevented

Patient-perspective problem-type code: F1. Wrong/late/missed/delayed diagnosis

Scenario23. GP surgery

Briefly describe the mistake or problem and how it happened. *"I have been diagnosed with bowel cancer, I knew something was wrong but over 4 visits to GP surgery over a 2 week period I was fobbed off by the GP who told me it was probably gastritis, it took 2 weeks to get a referral to a specialist"*

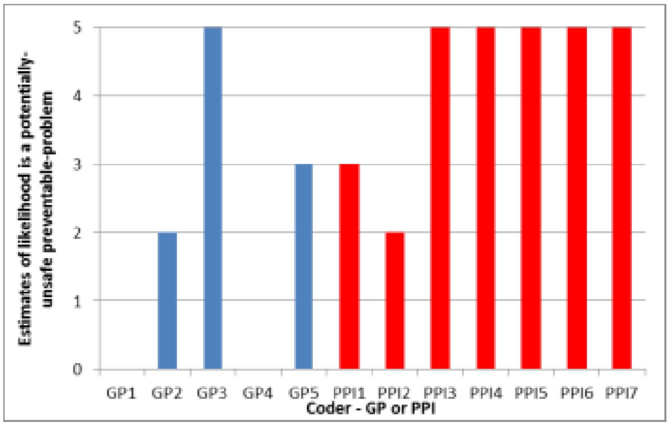

5=very likely or certain, 4=probably, 3=possibly, 2=unlikely, 1=definitely not, 0 = insufficient information or don't know

Could the mistake or problem have been avoided? If so how? *"I feel it was obvious from my appearance - massively distended stomach that - something serious was wrong with me, by the time I finally was referred I was seriously ill, this could have been avoided by an x-ray or quicker referral"*

Were you able to talk about the mistake or problem with anybody working in the primary care service? *"Yes, district nurse, who told me there is a framework in place for GPs that they have to stick to whilst diagnosing issues"*

Patient-reported prospect of harm: health was actually made worse by a problem or error that could have been prevented

Patient-perspective problem-type code: F1. Wrong/late/missed/delayed diagnosis

Scenario24. GP surgery

Briefly describe the mistake or problem and how it happened. *"Low blood count not identified because doctor didn't do blood test. Taken to hospital, died and brought back to life"*

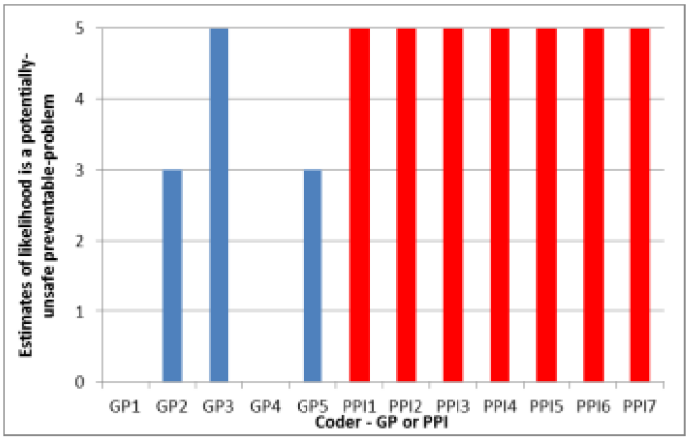

5=very likely or certain, 4=probably, 3=possibly, 2=unlikely, 1=definitely not, 0 = insufficient information or don't know

Could the mistake or problem have been avoided? If so how? *"a different drug should have been given"*

Were you able to talk about the mistake or problem with anybody working in the primary care service? *"Yes, the doctor"*

Patient-reported prospect of harm: health was actually made worse by a problem or error that could have been prevented

Patient-perspective problem-type code: B4. Investigation not thorough enough

Scenario25. GP surgery

Briefly describe the mistake or problem and how it happened. *"Had lump on back and thought was an abscess. Went to GP for antibiotics was told "nothing there, it was in my head". Three days later had to have an emergency operation to remove it."*

Could the mistake or problem have been avoided? If so how? *"by correct diagnosis"*

Were you able to talk about the mistake or problem with anybody working in the primary care service? *"No, I had the opportunity but did not feel comfortable discussing the problem or error"*

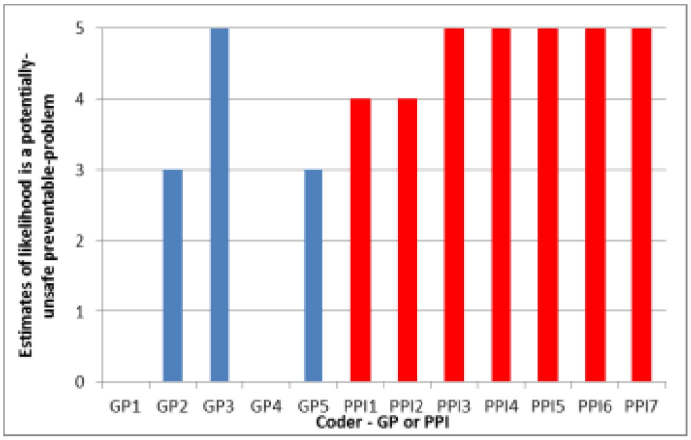

5=very likely or certain, 4=probably, 3=possibly, 2=unlikely, 1=definitely not, 0 = insufficient information or don't know

Patient-reported prospect of harm: health was actually made worse by a problem or error that could have been prevented

Patient-perspective problem-type code: F1. Wrong/late/missed/delayed diagnosis

Scenario26. GP surgery

Briefly describe the mistake or problem and how it happened. "*I had gall stones and they told me it was indigestion. Pain increased over three months. Had to have an emergency operation to have my gall bladder removed. Resulted in me having damage to my liver and pancreatitis"*

Could the mistake or problem have been avoided? If so how? *"listened to me when I told them it wasn't indigestion which would have been nice. The pain felt like I was having a heart attack and not like the pain from eating something dodgy"*

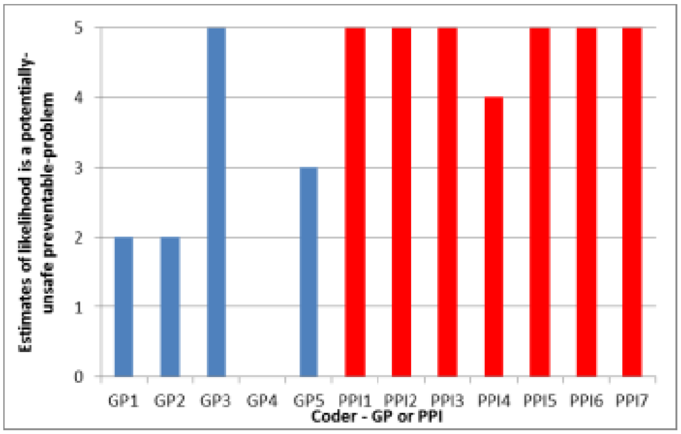

5=very likely or certain, 4=probably, 3=possibly, 2=unlikely, 1=definitely not, 0 = insufficient information or don't know

Were you able to talk about the mistake or problem with anybody working in the primary care service? *"No, I could not find anybody with whom I could discuss the problem or error"*

Patient-reported prospect of harm: health was actually made worse by a problem or error that could have been prevented

Patient-perspective problem-type code: F1. Wrong/late/missed/delayed diagnosis; D1. Clinician seemed to lack interest in the patient's health problem or did not listen carefully enough

Scenario27. GP surgery

Briefly describe the mistake or problem and how it happened. *"I have arthritis and I was prescribed a medication, Diclofenac, an anti-inflammatory. After taking this, I had problems and went to the GP and had a blood test. They lost the results and I became even more ill and when I rang them, they told me I was allergic to Diclofenac and I was to stop taking it immediately. It was causing kidney failure, liver failure and high blood pressure."*

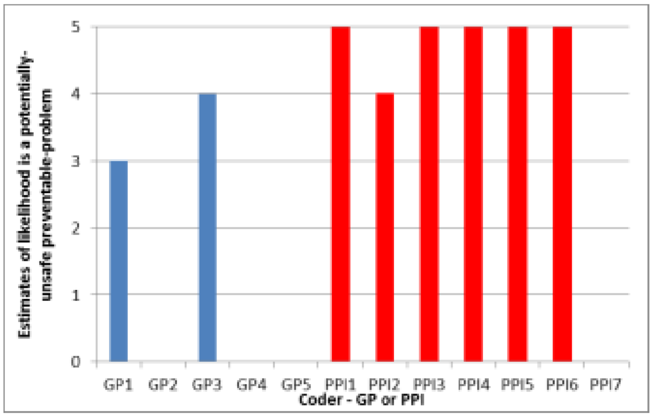

5=very likely or certain, 4=probably, 3=possibly, 2=unlikely, 1=definitely not, 0 = insufficient information or don't know

Could the mistake or problem have been avoided? If so how? *"They shouldn't have lost the results of the blood test. Later when I was feeling worse and I rang them up, they had found the results but not let me know which was another week later. They should have rung me not the other way round. That was poor communication. There should have been a better way of letting me know the results of the blood test. Luck for me, I was feeling so ill that I stopped taking the Diclofenac which they should have told me I was allergic to"*

Were you able to talk about the mistake or problem with anybody working in the primary care service? *"No, I know they're busy and there are people who need their help more than I do"*

Patient-reported prospect of harm: prompted via Q10 (Box 1 main paper)

Patient-perspective problem-type code: C1.1.3 Long term or continued prescribing without review or consideration of long term or side effects; B1. Test results lost or other problem with investigation paperwork

Scenario28. GP surgery

Briefly describe the mistake or problem and how it happened. *"I had stomach pains and was given the wrong medication which made it worse"*

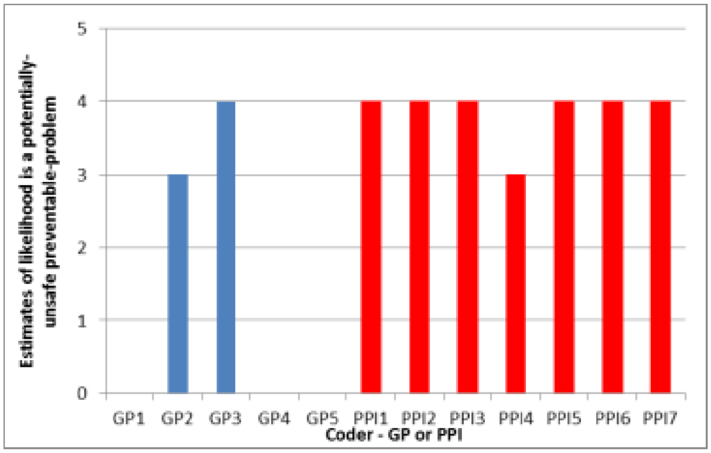

5=very likely or certain, 4=probably, 3=possibly, 2=unlikely, 1=definitely not, 0 = insufficient information or don't know

Could the mistake or problem have been avoided? If so how? *"If I had had more tests the problem could have been avoided."*

Were you able to talk about the mistake or problem with anybody working in the primary care service? *"Yes, another doctor and they advised me to stop taking the medication"*

Patient-reported prospect of harm: health was actually made worse by a problem or error that could have been prevented

Patient-perspective problem-type code: C1.1.1 Prescribed wrong or inappropriate drug; B4. Investigation not thorough enough

Scenario29. GP surgery

Briefly describe the mistake or problem and how it happened. *"I went to the GP and had a blood test. A month later they rang me up to tell me they had forgotten to tell me I had streptococcus and should have been on an antibiotic. In the intervening month I was ill without having taken the antibiotic"*

Could the mistake or problem have been avoided? If so how? *"Maybe they should have taken more care of their records and follow up"*

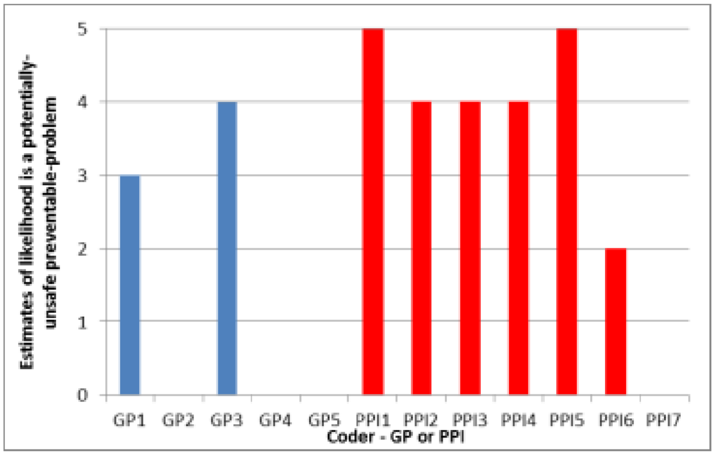

5=very likely or certain, 4=probably, 3=possibly, 2=unlikely, 1=definitely not, 0 = insufficient information or don't know

Were you able to talk about the mistake or problem with anybody working in the primary care service? *"No, I did not notice the problem or error at the time"*

Patient-reported prospect of harm: prompted via Q10 (Box 1 main paper)

Patient-perspective problem-type code: F1. Wrong/late/missed/delayed diagnosis; B1. Test results lost or other problem with investigation paperwork

Scenario30. Pharmacy

Briefly describe the mistake or problem and how it happened. *"It was routine prescription for blood pressure pills and they handed them over in a box in a stapled bag and when I got home I saw it was somebody else's medicine with my address label on. My husband took it back and they exchanged it for the correct medicine. About two weeks later we received a letter of apology which said the pharmacy had "put procedures in place so that the mistake wouldn't happen again". We were happy with that."*

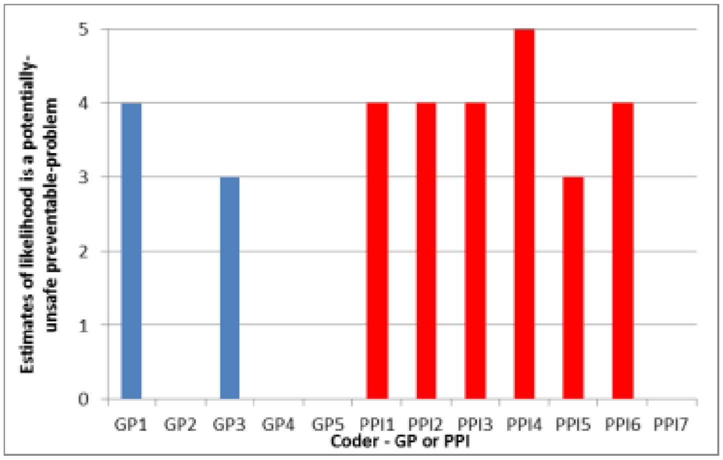

5=very likely or certain, 4=probably, 3=possibly, 2=unlikely, 1=definitely not, 0 = insufficient information or don't know

Could the mistake or problem have been avoided? If so how? *"I don't know how the problem happened at the pharmacy. Perhaps somebody at the pharmacy could check each prescription before it's issued. Perhaps I could have checked it myself."*

Were you able to talk about the mistake or problem with anybody working in the primary care service? *"Yes, their response was the letter of apology."*

Patient-reported prospect of harm: prompted via Q10 (Box 1 main paper)

Patient-perspective problem-type code: C1.3.2 Being given another patient's drugs or prescription

Scenario31. Pharmacy

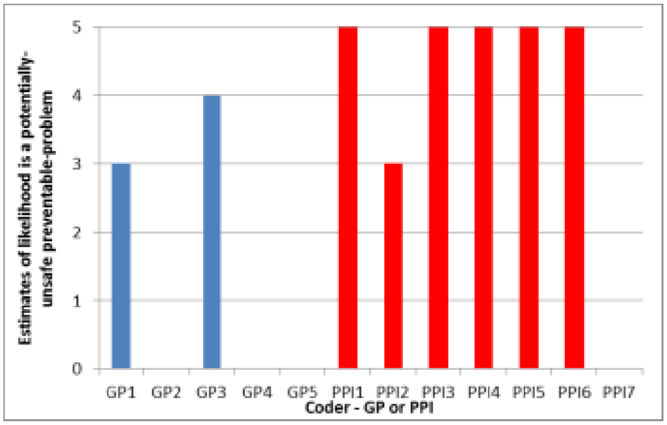

5=very likely or certain, 4=probably, 3=possibly, 2=unlikely, 1=definitely not, 0 = insufficient information or don't know

Briefly describe the mistake or problem and how it happened. *"The GP prescribed particular blood pressure tablets. The pharmacist at Boots changed the GPs prescription for a different tablet which had an adverse effect on me. It made me sick, headaches and dizziness. I went back to the GP who confirmed they were the wrong tablets and that the pharmacist isn't allowed to change a particular make of tablet. I went back to Boots and the pharmacist said they had stopped making the tablets my GP prescribed. I phoned the makers of the tablets and found that the tablets are still made. I remonstrated with the pharmacist who banned me from the shop and threatened to have me physically removed from the shop. I had been using the shop for over 40 years. I came home and phoned Boots head office and told them I would report the incident to my local newspaper and TV. I phoned the newspaper and TV wanted to film me outside the shop but a director from Boots came to my home to apologise personally and the pharmacist was forced to ring me to apologise. The pharmacist agreed that they were in breach of contract by changing the GPs prescription. When they apologised I regarded that as the end of the matter. For the last 3 months they have provided the correct tablets and on time."*

Could the mistake or problem have been avoided? If so how? *"The pharmacy is far too busy and they've exceeded their capability. Their ordering procedure means they too often run out of the correct tablets"*

Were you able to talk about the mistake or problem with anybody working in the primary care service? *"Yes, Chemist / Pharmacist, they admitted that previous medicine was wrong*

Patient-reported prospect of harm: prompted via Q10 (Box 1 main paper)

Patient-perspective problem-type code: C1.2.1 Medication not dispensed or administered as intended or prescribed

Scenario32. Pharmacy

Briefly describe the mistake or problem and how it happened. *"Wrong prescription tablets issued in error, name of patient was correct but the tablets were totally incorrect."*

Could the mistake or problem have been avoided? If so how? "*Pharmacy should have taken more care"*

Were you able to talk about the mistake or problem with anybody working in the primary care service? "Yes, *spoke to pharmacist and correct prescription was issued"*

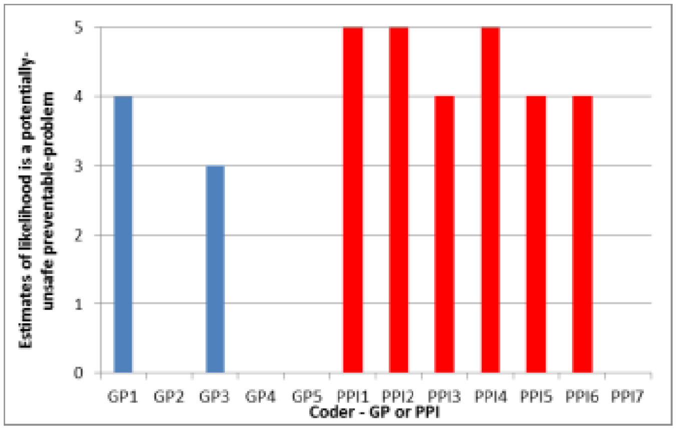

5=very likely or certain, 4=probably, 3=possibly, 2=unlikely, 1=definitely not, 0 = insufficient information or don't know

Patient-reported prospect of harm: prompted via Q10 (Box 1 main paper)

Patient-perspective problem-type code: C1.2.1 Medication not dispensed or administered as intended or prescribed

Scenario33. GP surgery

Briefly describe the mistake or problem and how it happened. *"had ear problem and GP provided treatment for 2 years but no response to medication. Within one month of being referred and treated by specialist the problem cleared up"*

Could the mistake or problem have been avoided? If so how? "*by earlier referral to specialist"*

Were you able to talk about the mistake or problem with anybody working in the primary care service? *"No, I could not find anybody with whom I could discuss the problem or error"*

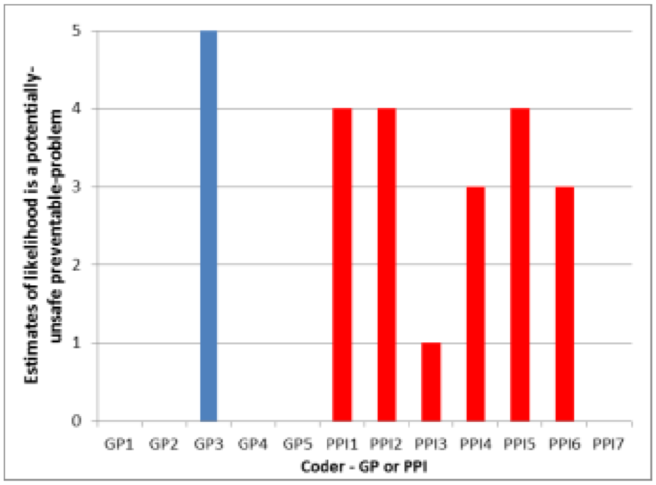

5=very likely or certain, 4=probably, 3=possibly, 2=unlikely, 1=definitely not, 0 = insufficient information or don't know

Patient-reported prospect of harm: prompted via Q10 (Box 1 main paper)

Patient-perspective problem-type code: B5. Not referred when patient felt was needed

<u>Patient reported scenarios occurring during the past 12 months that clinicians scored as higher likelihood to be a potentially-unsafe preventable-problem in primary care (median score is higher than "possibly" and at least 2 clinicians gave a score or one clinician scored "very likely or certain") from the pilot study (reference 24)</u>

Scenario34. GP surgery

Briefly describe the mistake or problem and how it happened. *"Prescription drug, anti-inflammatory for arthritis, caused acute stomach pains & violent vomiting. Repeat prescription for twelve years without any discussion."*

Could the mistake or problem have been avoided? If so how? "*Possible discussion about dangers of continuous taking of prescription drugs, which in the event were stopped after the incident."*

Were you able to talk about the mistake or problem with anybody working in the primary care service? *"No I did not notice the mistake or problem at the time"*

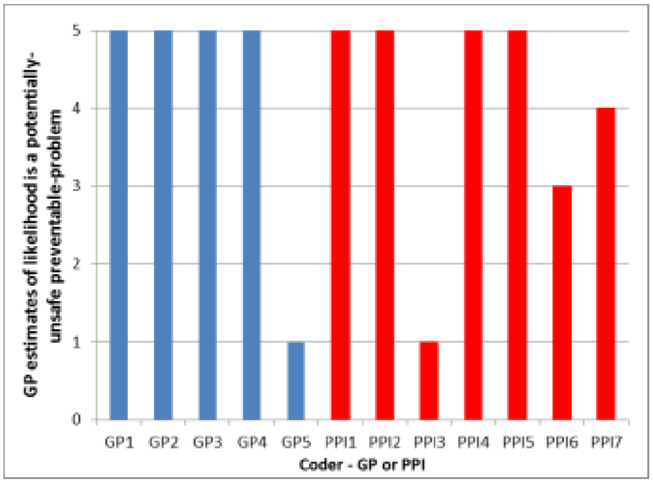

5=very likely or certain, 4=probably, 3=possibly, 2=unlikely, 1=definitely not, 0 = insufficient information or don't know

Patient-reported prospect of harm: suspected your health has been made worse by a problem or error that could have been prevented

Patient-perspective problem-type code: C1.1.3 Long term or continued prescribing without review or consideration of long term or side effects

Scenario35. GP surgery

Briefly describe the mistake or problem and how it happened. *"Insulin type was changed by specialist but previous insulin prescribed by GP as notes had not been updated"*

Could the mistake or problem have been avoided? If so how? "*Yes GP notes should have been updated with new medication"*

Were you able to talk about the mistake or problem with anybody working in the primary care service? *"Practice manager resolved the problem and apologised"*

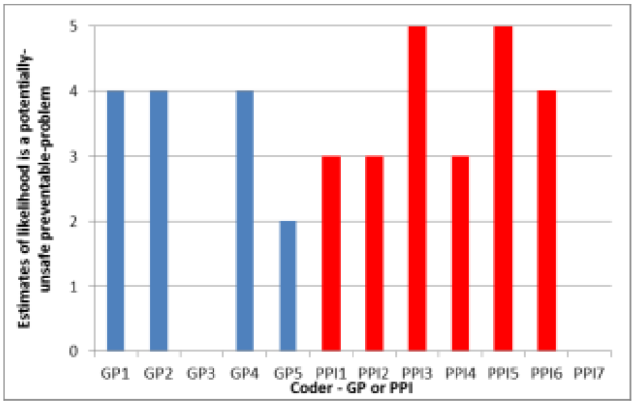

5=very likely or certain, 4=probably, 3=possibly, 2=unlikely, 1=definitely not, 0 = insufficient information or don't know

Patient-reported prospect of harm: prompted via Q10 (Box 1 main paper)

Patient-perspective problem-type code: A2. Incorrect notes/inadequate notes/notes not kept up to date; C1.1.6 Out of date repeat prescription mistakenly re-issued

Scenario36. GP surgery

Briefly describe the mistake or problem and how it happened. *"Two out of three Doctors not listening to what I was asking; April I had two big bleeds from my Penis, Doctor 1 did a test and gave antibiotics. Went to 2nd Doctor for Diabetic check and told him of problem - nothing except another test come back in ten days. Went to the third doctor who said the test didn't show anything but when I mentioned my feelings about a problem, he look and said yes you do have a problem. In 2 weeks I was in having tests and 3 operations for cancer."*

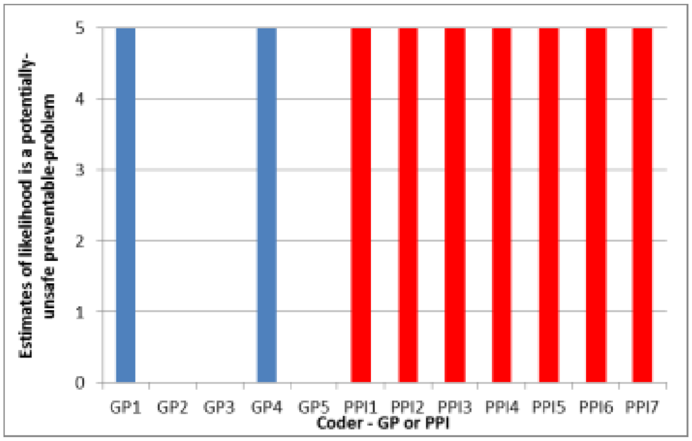

5=very likely or certain, 4=probably, 3=possibly, 2=unlikely, 1=definitely not, 0 = insufficient information or don't know

Could the mistake or problem have been avoided? If so how? "*Listen to me"*

Were you able to talk about the mistake or problem with anybody working in the primary care service? *"No, I could not find anybody with whom I could discuss the mistake or problem (The third doctor was amazing with me. He said to keep in touch and if I had any problems to ring him and he still wants me to ring him after my three operations.)"*

Patient-reported prospect of harm: suspected your health has been made worse by a problem or error that could have been prevented

Patient-perspective problem-type code: D1. Clinician seemed to lack interest in the patient's health problem or did not listen carefully enough; F1. Wrong/late/missed/delayed diagnosis

Scenario37. GP surgery

Briefly describe the mistake or problem and how it happened. *"Changed diabetes medication to an alternative which my notes from 1980's should show I respond badly to"*

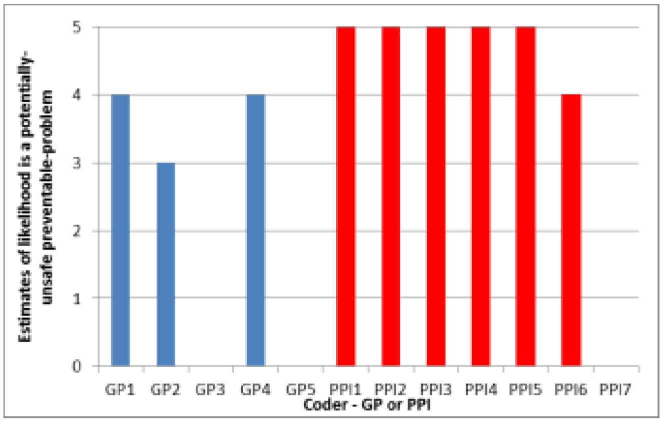

5=very likely or certain, 4=probably, 3=possibly, 2=unlikely, 1=definitely not, 0 = insufficient information or don't know

Could the mistake or problem have been avoided? If so how? "*Read the notes on every medication change but unfortunately that is unrealistic under the time restrictions on GP's. Put early notes on-line and flag medication allergies/problems."*

Were you able to talk about the mistake or problem with anybody working in the primary care service? *"Yes, my own GP who had returned from holiday"*

Patient-reported prospect of harm: suspected your health has been made worse by a problem or error that could have been prevented

Patient-perspective problem-type code: C1.1.4 Prescribed drug when should have known contra-indicated *e.g.* patient had informed clinician of allergy, adverse reaction or it was in the records

Scenario38. GP surgery

Briefly describe the mistake or problem and how it happened. *"Told the GP the medication was making my hair fall out & he kept me on it for another 3 months. I had to see another GP to get him to change my medication. In the meantime I have lost 3/4 of my hair. Not sure if it will ever grow back."*

Could the mistake or problem have been avoided? If so how? "*yes, by the GP listening to what I was saying."*

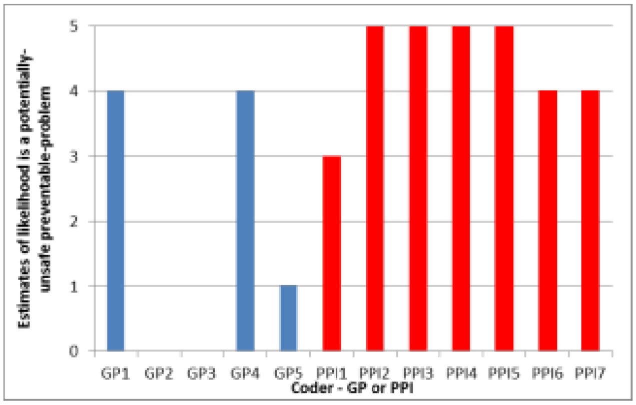

5=very likely or certain, 4=probably, 3=possibly, 2=unlikely, 1=definitely not, 0 = insufficient information or don't know

Were you able to talk about the mistake or problem with anybody working in the primary care service? *"Yes, GP"*

Patient-reported prospect of harm: suspected your health has been made worse by a problem or error that could have been prevented

Patient-perspective problem-type code: D1. Clinician seemed to lack interest in the patient's health problem or did not listen carefully enough; C1.1.3 Long term or continued prescribing without review or consideration of long term or side effects

Scenario39. GP surgery

Briefly describe the mistake or problem and how it happened. *"Successfully treated for prostate cancer 2006 but suffered some loss of sexual performance; Viagra recommended BUT I take isosorbide nitrate for a following heart attack; the two are contradictory and could produce further heart problems. A routine diabetes check-up at which the sexual problem was discussed saw an automatic prescribing of Viagra; obviously without reference to my medical records."*

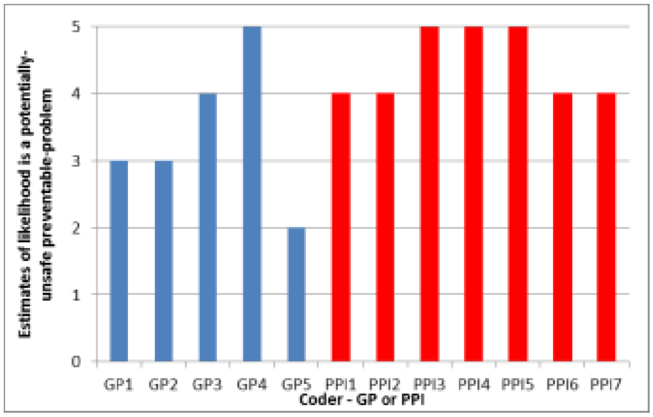

5=very likely or certain, 4=probably, 3=possibly, 2=unlikely, 1=definitely not, 0 = insufficient information or don't know

Could the mistake or problem have been avoided? If so how? "*Read the medical notes."*

Were you able to talk about the mistake or problem with anybody working in the primary care service? *"No; I felt I was going to cause trouble"*

Patient-reported prospect of harm: prompted via Q10 (Box 1 main paper)

Patient-perspective problem-type code: C1.1.1 Prescribed wrong or inappropriate drug

Scenario40. GP surgery

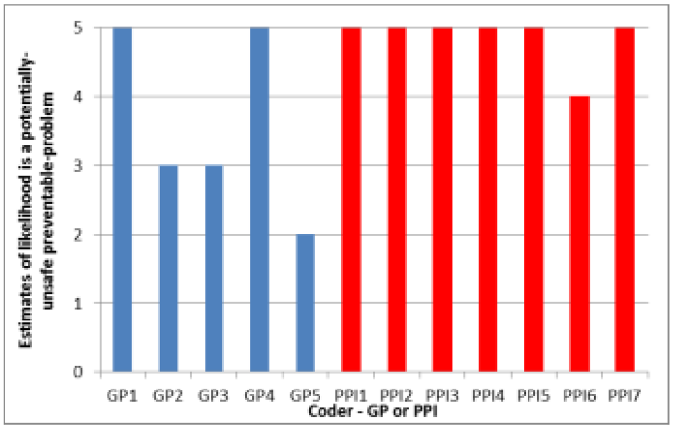

5=very likely or certain, 4=probably, 3=possibly, 2=unlikely, 1=definitely not, 0 = insufficient information or don't know

Briefly describe the mistake or problem and how it happened. *"I was given steroids for a chest infection but not alerted to the fact they make your sugars go massively high! Within a few hours I was high and not able to bring them down, fearing a DKA I headed for the hospital to correct a very easily avoidable issue. I also attended my GP 6 years ago to be given strong antacids for pain in my stomach that was actually a DKA I was admitted to hospital a few hours later! The GP never even suggested it could be linked to my diabetes and as it was my first DKA I had no idea that's how they can feel"*

Could the mistake or problem have been avoided? If so how? "*Both could have been avoided The steroids - if the prescribing nurse had considered my diabetes I'd have been given proper advice as to how to deal with them as a diabetic or given different meds. The DKA simple questions or explanation as to how DKAs can present would have made me family and the doctor realise I was in trouble."*

Were you able to talk about the mistake or problem with anybody working in the primary care service? *"I wrote a letter to the surgery concerning the steroids anonymously to alert them of my concern and the DKA. I was too poorly to even consider seeking correction or explanation"*

Patient-reported prospect of harm: health was actually made worse by a problem or error that could have been prevented

Patient-perspective problem-type code: C1.1.4 Prescribed drug when should have known contra-indicated *e.g.* patient had informed clinician of allergy, adverse reaction or it was in the records; E3. Incorrect advice/no advice given by clinician

Scenario41. GP surgery

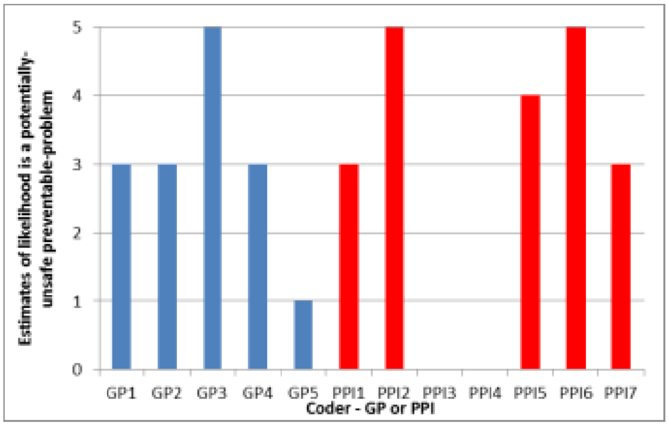

5=very likely or certain, 4=probably, 3=possibly, 2=unlikely, 1=definitely not, 0 = insufficient information or don't know

Briefly describe the mistake or problem and how it happened. *"reception staff making clinical decisions which were at odds with what had been discussed with my GP"*

Could the mistake or problem have been avoided? If so how? "*Yes, reception staff shouldn't be making clinical decisions"*

Were you able to talk about the mistake or problem with anybody working in the primary care service? *"No, had the opportunity but did not feel comfortable to discuss the mistake or problem"*

Patient-reported prospect of harm: suspected your health has been made worse by a problem or error that could have been prevented

Patient-perspective problem-type code: E1. Administrative staff seemed to make clinical decisions

Scenario42. Pharmacist

Briefly describe the mistake or problem and how it happened. *"I was given a medicine belonging to somebody else as part of my monthly repeat prescription"*

Could the mistake or problem have been avoided? If so how? "*More care and attention when checking"*

Were you able to talk about the mistake or problem with anybody working in the primary care service? *"Yes, pharmacist"*

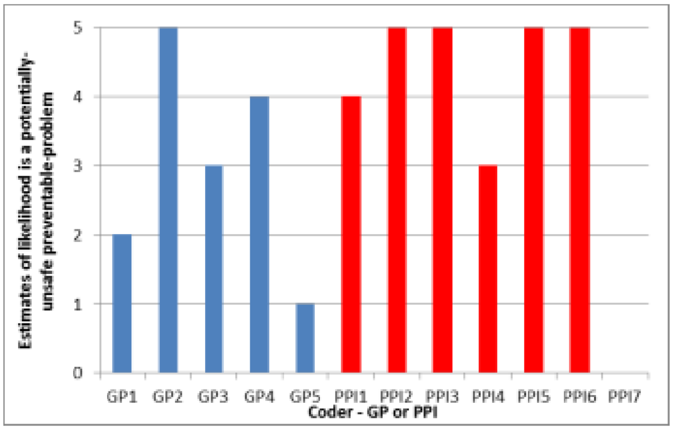

5=very likely or certain, 4=probably, 3=possibly, 2=unlikely, 1=definitely not, 0 = insufficient information or don't know

Patient-reported prospect of harm: prompted via Q10 (Box 1 main paper)

Patient-perspective problem-type code: C1.3.3 Wrong or inadequate advice about drug effects or how to use

Scenario43. GP surgery

Briefly describe the mistake or problem and how it happened. *"Poor diabetic annual review, foot check not correctly done just tested my foot pulses and nothing else"*

Could the mistake or problem have been avoided? If so how? "*Better training of staff"*

Were you able to talk about the mistake or problem with anybody working in the primary care service? *"No, had the opportunity but did not feel comfortable to discuss the mistake or problem"*

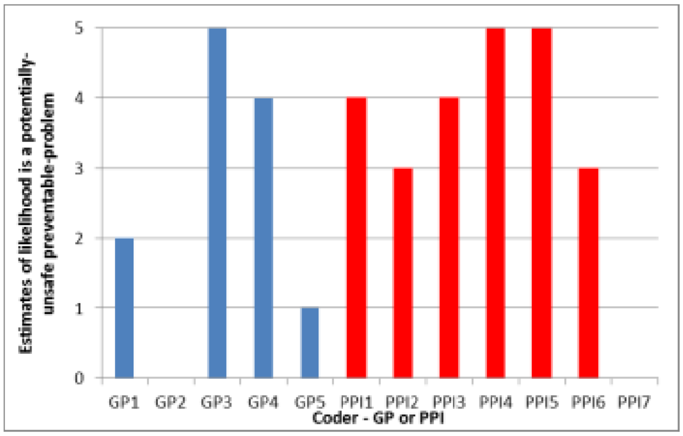

5=very likely or certain, 4=probably, 3=possibly, 2=unlikely, 1=definitely not, 0 = insufficient information or don't know

Patient-reported prospect of harm: suspected your health has been made worse by a problem or error that could have been prevented

Patient-perspective problem-type code: E2. Procedure was not carried out correctly

Scenario44. GP surgery

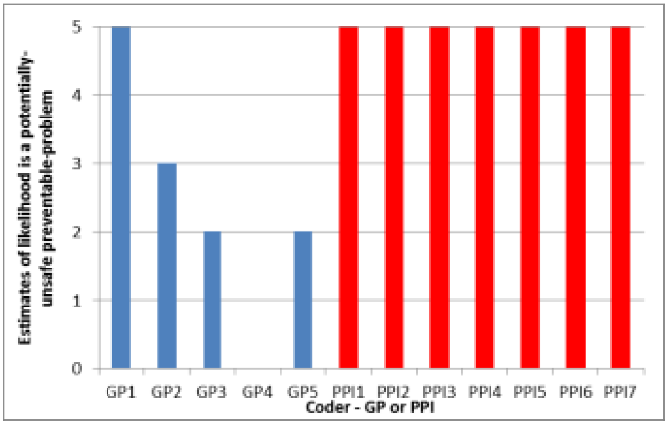

5=very likely or certain, 4=probably, 3=possibly, 2=unlikely, 1=definitely not, 0 = insufficient information or don't know

Briefly describe the mistake or problem and how it happened. *"Prior to a pain killing injection into my knee, I asked the GP who suggested the injection AND the GP who carried out the injection whether, as someone living with Type 1 diabetes, it would have any effect on my blood glucose levels. On both occasions, I was given an unequivocal No . In the event, within a few hours of the injection, my blood glucose rose significantly and remained high for several days. I felt unable to eat anything for 24 hours while I took on more and more insulin in order to bring my glucose levels down - I did not want to go to sleep that night simply because of the massive amount of insulin in my system."*

Could the mistake or problem have been avoided? If so how? "*Yes. I feel that both GPs should have a knowledge about the side effects of drugs they prescribe, administer and recommend."*

Were you able to talk about the mistake or problem with anybody working in the primary care service? *"No I could not find anybody with whom I could discuss the mistake or problem"*

Patient-reported prospect of harm: your health has been made worse by a problem or error that could have been prevented

Patient-perspective problem-type code: E3. Incorrect advice/no advice given by clinician

Scenario45. GP surgery

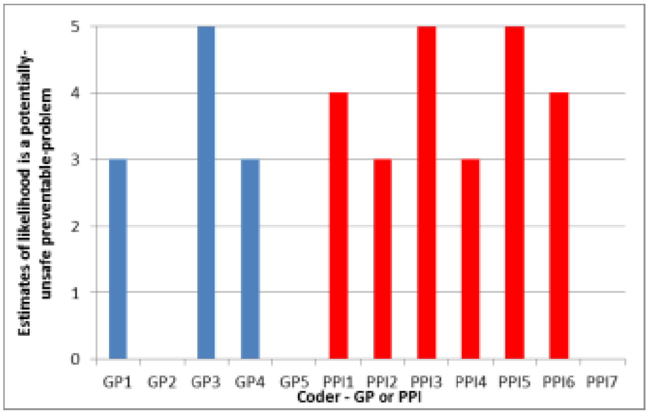

5=very likely or certain, 4=probably, 3=possibly, 2=unlikely, 1=definitely not, 0 = insufficient information or don't know

Briefly describe the mistake or problem and how it happened. *"GP completely overlooked symptoms and prescribed antibiotic after antibiotic without investigation or referral"*

Could the mistake or problem have been avoided? If so how? "*Yes by listening to history of complaints, carrying out appropriate tests instead of just giving antibiotics"*

Were you able to talk about the mistake or problem with anybody working in the primary care service? *"No I did not notice the mistake or problem at the time"*

Patient-reported prospect of harm: prompted via Q10 (Box 1 main paper)

Patient-perspective problem-type code: D1. Clinician seemed to lack interest in the patient's health problem or did not listen carefully enough; F1. Wrong/late/missed/delayed diagnosis

Scenario46. GP surgery

Briefly describe the mistake or problem and how it happened. *"Several times prescriptions have been incorrectly issued due to similar names for drugs or the same name with different strengths"*

Could the mistake or problem have been avoided? If so how? "*Yes, by more accurate or double data entry. Now solved by self-request using web systems."*

Were you able to talk about the mistake or problem with anybody working in the primary care service? "Yes, *they did not want to know or seem to care unless a formal complaint was made"*

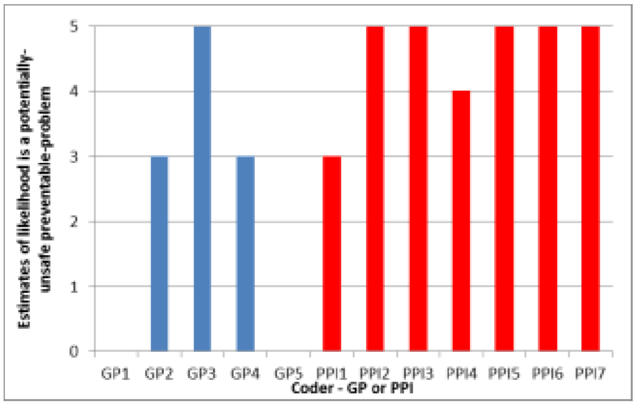

5=very likely or certain, 4=probably, 3=possibly, 2=unlikely, 1=definitely not, 0 = insufficient information or don't know

Patient-reported prospect of harm: your health has been made worse by a problem or error that could have been prevented

Patient-perspective problem-type code: C1.1.5 Repeat prescription unintentionally changed

Scenario47. GP surgery

Briefly describe the mistake or problem and how it happened. *"A simple error occurred with an incorrect prescription. When I tried to bring this to the attention of the receptionist she treated me with disdain and in a challenging manner. She then proceeded to start to read my notes aloud in the public reception area. I felt that this was unacceptable behaviour. When I tried to tackle the receptionist about her behaviour I felt as if I was under threat. It caused me to feel very stressed, frustrated and ill tempered."*

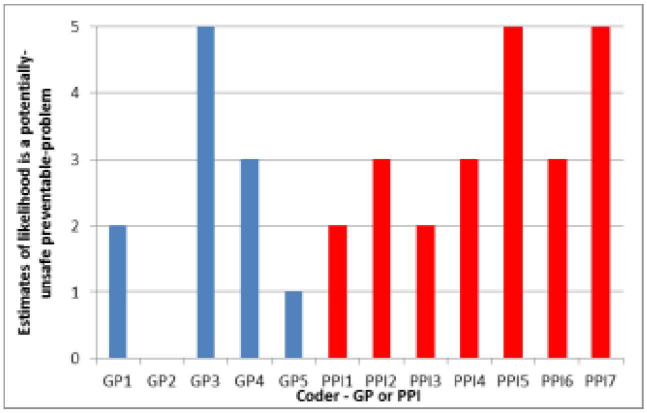

5=very likely or certain, 4=probably, 3=possibly, 2=unlikely, 1=definitely not, 0 = insufficient information or don't know

Could the mistake or problem have been avoided? If so how? "*If the receptionist had been willing to listen to what I was saying."*

Were you able to talk about the mistake or problem with anybody working in the primary care service? "*I did speak to a lady who said she was the practice manager but I felt that they were not interested in resolving the problem"*

Patient-reported prospect of harm: suspected your health has been made worse by a problem or error that could have been prevented

Patient-perspective problem-type code: D3. Communication problem between patient and primary care staff; C1 Medication error not otherwise specified /other problem

Scenario48. GP Surgery

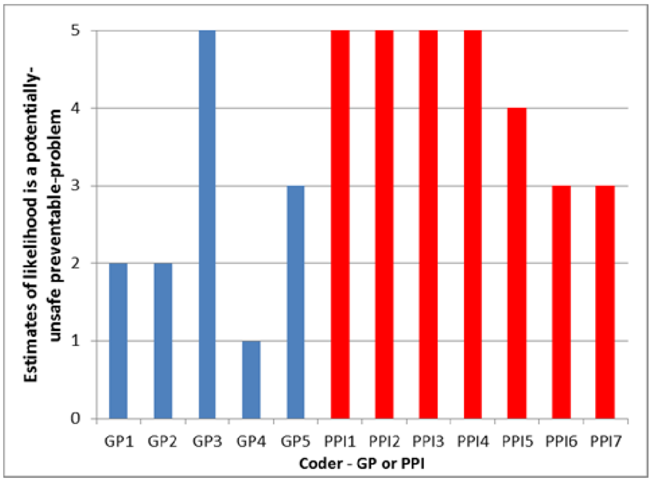

5=very likely or certain, 4=probably, 3=possibly, 2=unlikely, 1=definitely not, 0 = insufficient information or don't know

Briefly describe the mistake or problem and how it happened. *"Went to see GP because I feared the pain in one of my legs may have been Peripheral Artery Disease - hardening of the arteries, having had a (non-blood) relative who suffered from this and subsequently died - of a heart attack. Oh yes, said the GP, well, you will have it won't you? Why? I asked expecting her to say eg because you are a smoker, or maybe my age (65) or something else I wasn't aware of. But what she actually told me was 'Because you are a diabetic!' Whaaat? I exclaimed - you mean ALL diabetics will inevitably get this, and there's no way to prevent it? Yes she said and shrugged. I said 'Thanks for nothing then' and left. Instead I left, came home and went straight on-line to make an appointment with someone more sensible, which I did and after taking my leg/ankle pulses and BPs etc - he chatted to me and said he would refer me for a cardiology consultation at the hospital. This IS what I expected in the first place and now it IS being taken care of."*

Could the mistake or problem have been avoided? If so how? *"By training the GP properly in the first place"*

Were you able to talk about the mistake or problem with anybody working in the primary care service? "? *"I explained to GP 2 But I don't know what if anything was done about it, or how I could find that out."*

Patient-reported prospect of harm: your health has been made worse by a problem or error that could have been prevented

Patient-perspective problem-type code: D1. Clinician seemed to lack interest in the patient's health problem or did not listen carefully enough

Patient reported scenarios occurring during the past 12 months that PPIs scored as higher likelihood to be a potentially-unsafe preventable-problem in primary care compared with clinicians – Ipsos MORI survey

Scenario49. GP surgery

Briefly describe the mistake or problem and how it happened. *"I was suicidal, phoned the crisis team and they kept telling me that they couldn't see me because I wasn't under a psychiatrist and that made the situation worse"*

Could the mistake or problem have been avoided? If so how? *"they just simply had to say that they would see me"*

Were you able to talk about the mistake or problem with anybody working in the primary care service?

"No, I did not get to see a psychiatrist until about three months later"

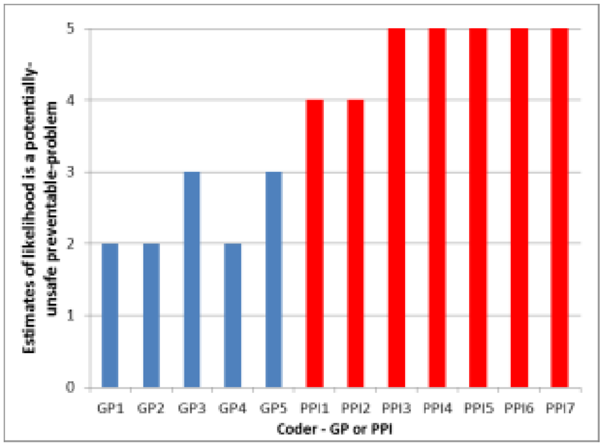

5=very likely or certain, 4=probably, 3=possibly, 2=unlikely, 1=definitely not, 0 = insufficient information or don't know

Patient-reported prospect of harm: your health has been made worse by a problem or error that could have been prevented

Patient-perspective problem-type code: A5. Unable to get an appointment/other problems with making appointment

Scenario50. Physiotherapy at GP surgery

Briefly describe the mistake or problem and how it happened. *"Broken wrist after coming off pushbike"*

Could the mistake or problem have been avoided? If so how? *"Physio caused fracture, after healing, to break again"*

Were you able to talk about the mistake or problem with anybody working in the primary care service? *"Yes, another doctor in practice"*

Patient-reported prospect of harm: your health has been made worse by a problem or error that could have been prevented

Patient-perspective problem-type code: E2.Procedure was not carried out correctly

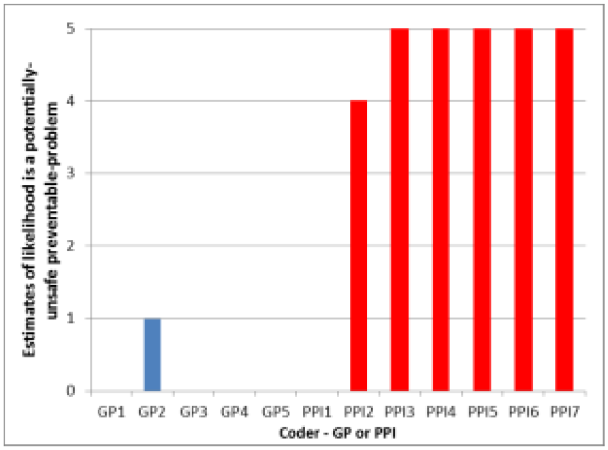

5=very likely or certain, 4=probably, 3=possibly, 2=unlikely, 1=definitely not, 0 = insufficient information or don't know

Scenario51. GP surgery

Briefly describe the mistake or problem and how it happened. *"Given some medication that brought about a nervous breakdown and crisis team attended within 4 hours. Seeing mental health social worker each week now as a result. Hearing voices and seeing things which I didn't before this medication."*

Could the mistake or problem have been avoided? If so how? *"GP could have listened more carefully and not changed my medication"*

Were you able to talk about the mistake or problem with anybody working in the primary care service? *"Yes, the crisis mental health team/the psychologist and social worker"*

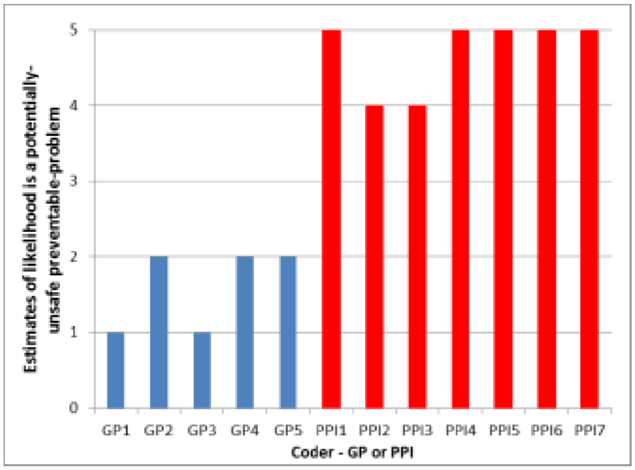

5=very likely or certain, 4=probably, 3=possibly, 2=unlikely, 1=definitely not, 0 = insufficient information or don't know

Patient-reported prospect of harm: suspected your health has been made worse by a problem or error that could have been prevented

Patient-perspective problem-type code: C1.1.2 Started new prescription or changed prescription without sufficient discussion, follow up or checks; D1. Clinician seemed to lack interest in the patient's health problem or did not listen carefully enough

Scenario52. Community mental health

Briefly describe the mistake or problem and how it happened. *"two years delay from GP referral to being able to see psychiatrist at community mental health service. Lack of access meant that he could not be diagnosed with a personality disorder trait in order for medication to be prescribed to treat the problem"*

Could the mistake or problem have been avoided? If so how? *"by referring him back to the previous psychiatrist he was with instead of worrying about boundary changes within the PCTs which are intended to manage caseloads. Basically he was out of catchment, also due to NHS cuts. Also feels these are the result of austerity and people should get social care to help"*

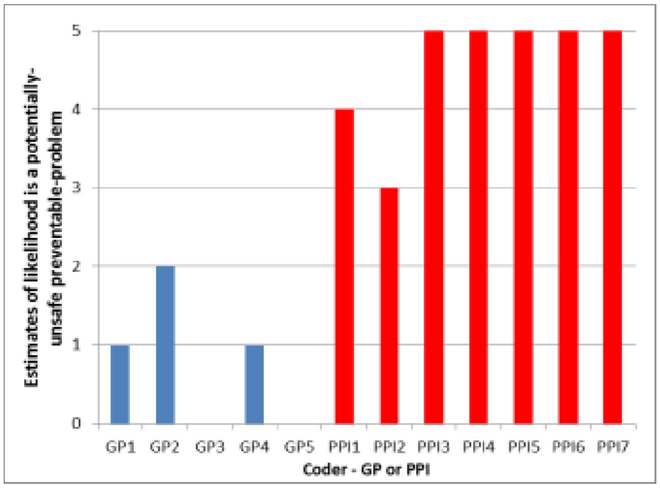

5=very likely or certain, 4=probably, 3=possibly, 2=unlikely, 1=definitely not, 0 = insufficient information or don't know

Were you able to talk about the mistake or problem with anybody working in the primary care service? *"Yes, secretary of mental health psychiatrist he should have seen but waiting for 2 years for*

Patient-reported prospect of harm: your health has been made worse by a problem or error that could have been prevented

Patient-perspective problem-type code: A5. Unable to get an appointment/other problems with making appointment

Scenario53. GP Surgery

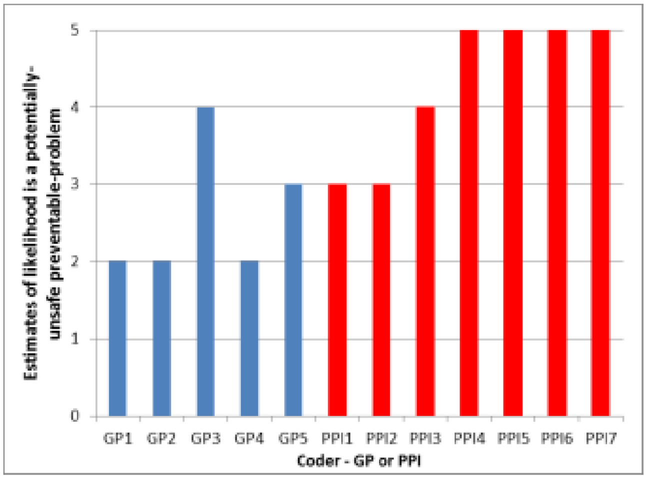

5=very likely or certain, 4=probably, 3=possibly, 2=unlikely, 1=definitely not, 0 = insufficient information or don't know

Briefly describe the mistake or problem and how it happened. "*I had sore throat and I told the doctor it felt it would go to my chest. He prescribed a throat spray, over 2 days I felt really poorly and ended up in hospital with pneumonia*"

Could the mistake or problem have been avoided? If so how? "*GP should have prescribed antibiotics*"

Were you able to talk about the mistake or problem with anybody working in the primary care service? "*No, I was too distressed to discuss the problem or error*"

Patient-reported prospect of harm: your health has been made worse by a problem or error that could have been prevented

Patient-perspective problem-type code: G1.Wrong treatment decision

Scenario54. GP Surgery

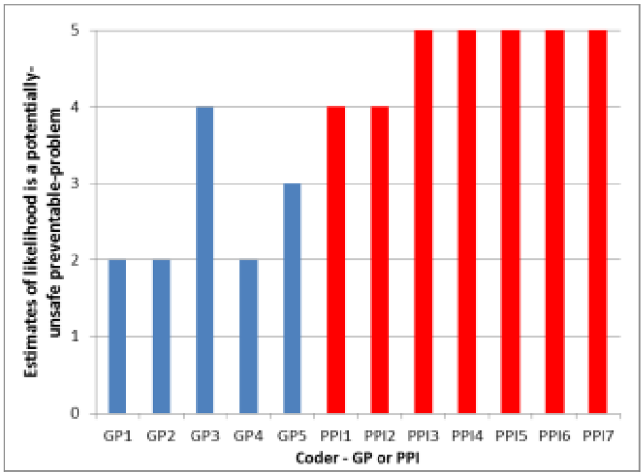

5=very likely or certain, 4=probably, 3=possibly, 2=unlikely, 1=definitely not, 0 = insufficient information or don't know

Briefly describe the mistake or problem and how it happened. "*Got stomach pain, it was very similar to gall bladder pain but had had that removed before so couldn't be that. At first would have made an appointment with my doctor but none were available for a month. I insisted and found out it was gall bladder stones in bile duct which is serious. Total delay (in pain) 3-4 days*"

Could the mistake or problem have been avoided? If so how? "*Quicker appointment*"

Were you able to talk about the mistake or problem with anybody working in the primary care "*Yes, spoke to doctor about the problem. No apology or changes to the service*"

Patient-reported prospect of harm: health could have been made worse had someone not noticed a problem or error

Patient-perspective problem-type code: A5. Unable to get an appointment/other problems with making appointment

Scenario55. Dental Surgery

Briefly describe the mistake or problem and how it happened. **“***Osteonecrosis of the jaw happened due to a tooth being extracted when it should not have been because of medication I was taking”*

Could the mistake or problem have been avoided? If so how? *“More knowledge on the part of the dental profession”*

Were you able to talk about the mistake or problem with anybody working in the primary care service? “*No, there was no point talking about the problem with the primary care service as the situation was beyond that”*

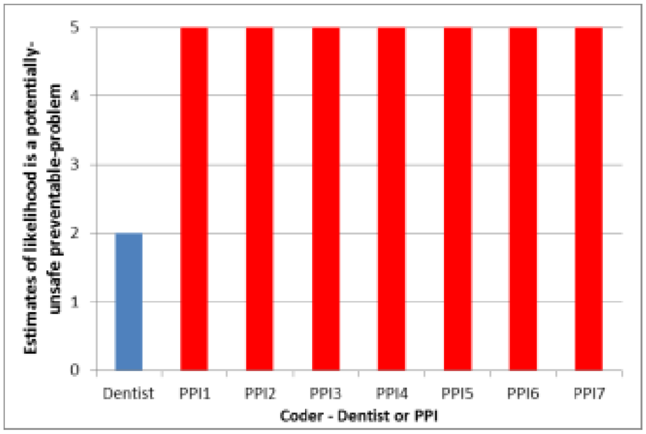

5=very likely or certain, 4=probably, 3=possibly, 2=unlikely, 1=definitely not, 0 = insufficient information or don’t know

Patient-reported prospect of harm: your health has been made worse by a problem or error that could have been prevented

Patient-perspective problem-type code: G1. Wrong treatment decision

Scenario56. Physiotherapy

Briefly describe the mistake or problem and how it happened. *“GP referred to physio for shoulder pain, physio made problem worse and operation was required”*

Could the mistake or problem have been avoided? If so how? *“inexperienced physio made wrong diagnosis”*

Were you able to talk about the mistake or problem with anybody working in the primary care service? “*Yes, GP***”**

Patient-reported prospect of harm: your health has been made worse by a problem or error that could have been prevented

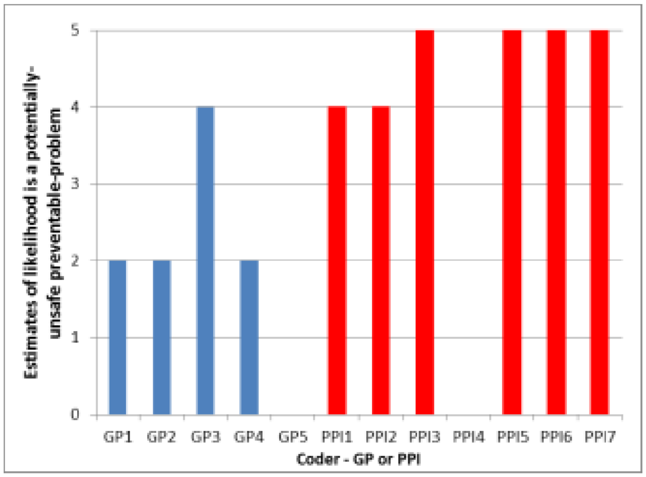

5=very likely or certain, 4=probably, 3=possibly, 2=unlikely, 1=definitely not, 0 = insufficient information or don’t know

Patient-perspective problem-type code:

F1. Wrong/late/missed/delayed diagnosis; G1. Wrong treatment decision

Scenario57. GP Surgery

Briefly describe the mistake or problem and how it happened. *"Have thyroid problem. GP reduced medication dose without a review and caused health to deteriorate"*

Could the mistake or problem have been avoided? If so how? *"by appropriate blood test taken regularly to monitor my thyroid status"*

Were you able to talk about the mistake or problem with anybody working in the primary care service? *"Yes, GP"*

Patient-reported prospect of harm: suspected your health has been made worse by a problem or error that could have been prevented

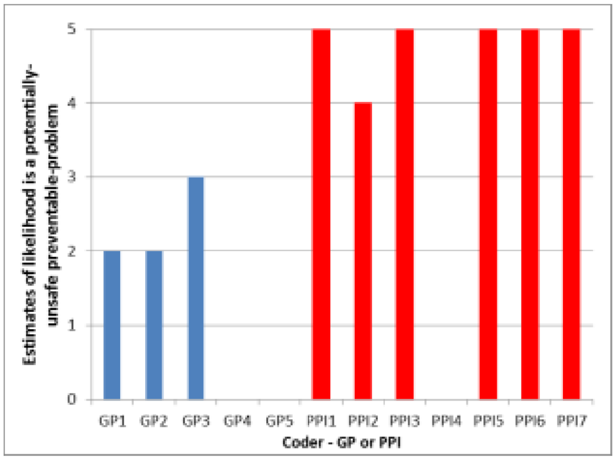

5=very likely or certain, 4=probably, 3=possibly, 2=unlikely, 1=definitely not, 0 = insufficient information or don't know

Patient-perspective problem-type code: B4. Investigation not thorough enough

Scenario58. GP Surgery

Briefly describe the mistake or problem and how it happened. *"review of drugs, GP indicated the high blood pressure, and decided to put me on blood pressure reducing tablets, which resulted in very bad side effects."*

Could the mistake or problem have been avoided? If so how? missing

Were you able to talk about the mistake or problem with anybody working in the primary care service? *"my daughter is GP, she advised me to stop taking the tablets, and monitor my own blood pressure which I did for a week and recorded it."*

Patient-reported prospect of harm: there was a problem or error that could have been prevented but it did not make your health worse

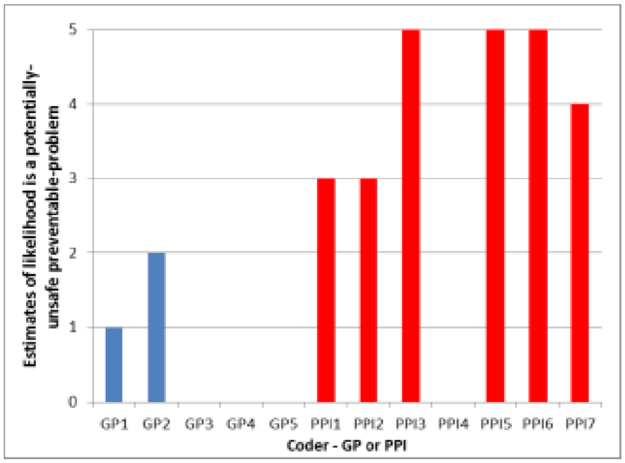

5=very likely or certain, 4=probably, 3=possibly, 2=unlikely, 1=definitely not, 0 = insufficient information or don't know

Patient-perspective problem-type code: C1 Medication error not otherwise specified /other problem

Scenario59. GP Surgery

Briefly describe the mistake or problem and how it happened. *"Complaining about severe pain in right shoulder then left shoulder for 3 years. I demanded to see a specialist. I saw a muscular skeletal specialist who diagnosed me with fibromyalgia, so I am no longer able to go to the gym now."*

Could the mistake or problem have been avoided? If so how? *"If the diagnosis had not have taken as long my overall health and fitness would not have deteriorated. It's affected my mental health and body image and I have paid over 2,000 pounds for private chiropractor"*

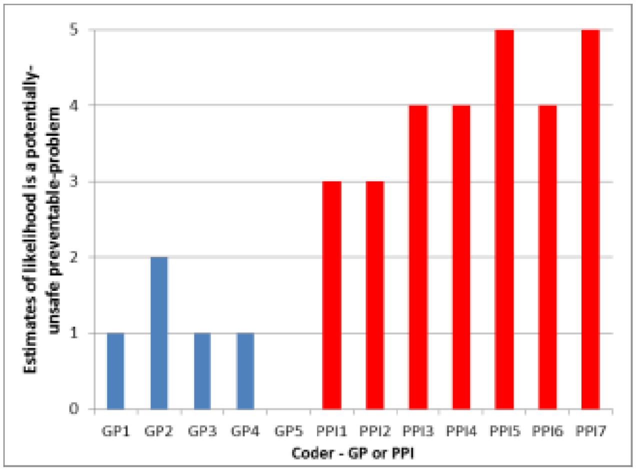

5=very likely or certain, 4=probably, 3=possibly, 2=unlikely, 1=definitely not, 0 = insufficient information or don't know

Were you able to talk about the mistake or problem with anybody working in the primary care service? *"the musculoskeletal specialist when referred listened to me and gave a diagnosis"*

Patient-reported prospect of harm: your health has been made worse by a problem or error that could have been prevented

Patient-perspective problem-type code: B5. Not referred when patient felt was needed

<u>Patient reported scenarios occurring during the past 12 months that PPIs scored as higher likelihood to be a potentially-unsafe preventable-problem in primary care compared with clinicians – pilot survey (reference 24)</u>

Scenario60. GP Surgery

Briefly describe the mistake or problem and how it happened. "*I had a severe reaction to Atorvastatin after a dose increase so much so that I was almost immobile and took 4 months to recover*"

Could the mistake or problem have been avoided? If so how? "*According to guidelines I should have been on the increased dose - it took a long time to convince the GP that I needed blood tests to find out why I couldn't walk. My GP was very hesitant to admit that I did have a reaction to statins.*"

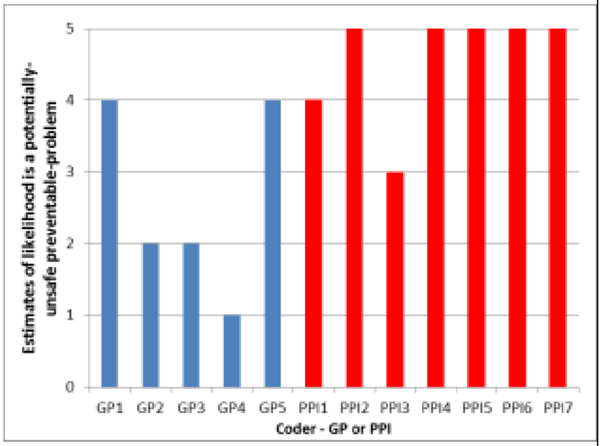

5=very likely or certain, 4=probably, 3=possibly, 2=unlikely, 1=definitely not, 0 = insufficient information or don't know

Were you able to talk about the mistake or problem with anybody working in the primary care service? **"***No I could not find anybody with whom I could discuss the mistake or problem. It was not really the GPs fault per se, just took a lot of convincing that there was a problem"*

Patient-reported prospect of harm: health could have been made worse had someone not noticed a problem or error

Patient-perspective problem-type code: C1.1.3 Long term or continued prescribing without review or consideration of long term or side effects

Scenario61. GP Surgery

Briefly describe the mistake or problem and how it happened. *"Doctor kept saying I had vitamin deficiency B1, it turned out I had peripheral neuropathy which is very painful"*

Could the mistake or problem have been avoided? If so how? *"I just needed the proper medication to help"*

Were you able to talk about the mistake or problem with anybody working in the primary care service? *"Just saw another Doctor and she knew straight away what the problem was - she was experienced with Diabetic problems. Yes had the opportunity but did not feel comfortable to discuss the mistake or problem"*

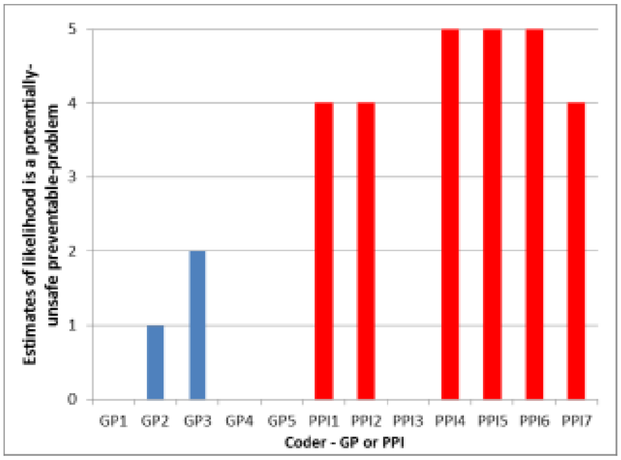

5=very likely or certain, 4=probably, 3=possibly, 2=unlikely, 1=definitely not, 0 = insufficient information or don't know

Patient-reported prospect of harm: prompted via Q10 (Box 1 main paper)

Patient-perspective problem-type code: F1. Wrong/late/missed/delayed diagnosis

Scenario62. GP Surgery

Briefly describe the mistake or problem and how it happened. *"Incapable diabetic doctor trying to take blood out the back of my hand haphazardly, not listening and resulting in me fitting and the student watching having to get help."*

Could the mistake or problem have been avoided? If so how? *"Yes. By listening to me"*

Were you able to talk about the mistake or problem with anybody working in the primary care service? *"No I could not find anybody with whom I could discuss the mistake or problem"*

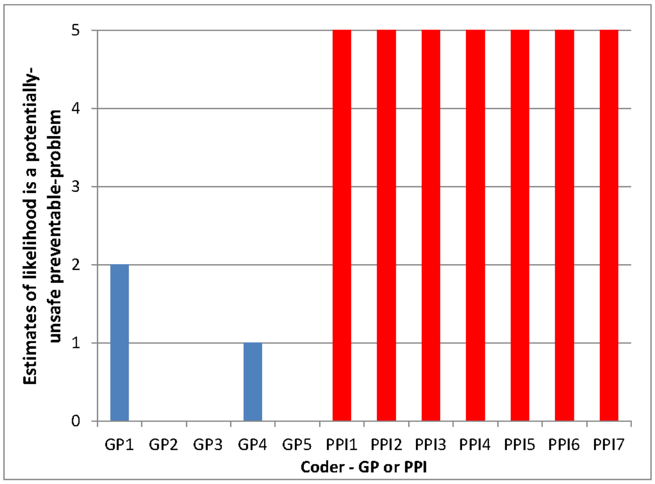

5=very likely or certain, 4=probably, 3=possibly, 2=unlikely, 1=definitely not, 0 = insufficient information or don't know

Patient-reported prospect of harm: prompted via Q10 (Box 1 main paper)

Patient-perspective problem-type code: E2. Procedure was not carried out correctly; D1. Clinician seemed to lack interest in the patient's health problem or did not listen carefully enough

Scenario63. Dental Surgery

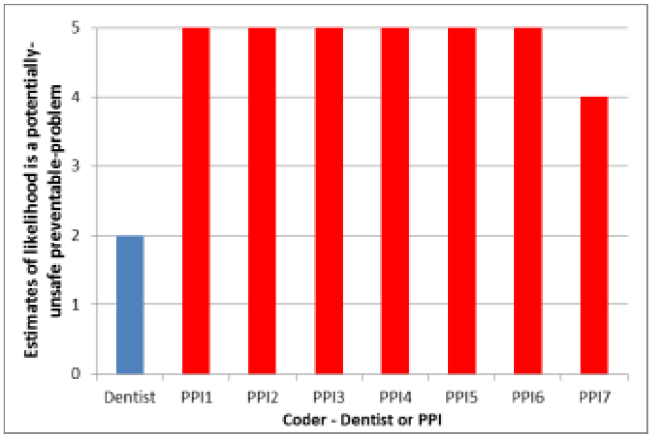

5=very likely or certain, 4=probably, 3=possibly, 2=unlikely, 1=definitely not, 0 = insufficient information or don't know

Briefly describe the mistake or problem and how it happened. *"I had an infection under my wisdom tooth. They agreed that the only way to solve the problem was to take the tooth out. They gave me an appointment to do this in 6 weeks. I am a type 1 diabetic and the infection was affecting my blood sugars and I was concerned that I would have to go to A&E if my blood sugars continued to rise due to the infection. It would have affected my health if I had not paid to go to a private dentist."*

Could the mistake or problem have been avoided? If so how? *"They could have taken out the tooth straight away. I was happy to wait at the emergency dentist for them to do this."*

Were you able to talk about the mistake or problem with anybody working in the primary care service? *"I explained but they said I would have to wait. They also asked if I needed a sugary drink when I said that my sugars were high so I was too scared to eat and had not eaten in 12hrs. It was clear they didn't understand diabetes."*

Patient-reported prospect of harm: health could have been made worse had someone not noticed a problem or error

Patient-perspective problem-type code: A5. Unable to get an appointment/other problems with making appointment

Scenario64. Dental Surgery

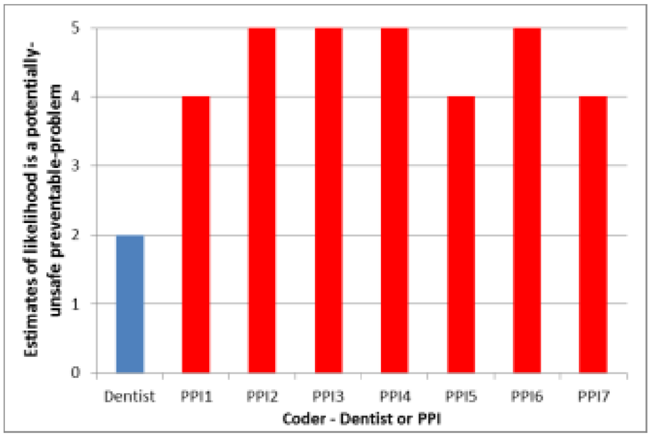

5=very likely or certain, 4=probably, 3=possibly, 2=unlikely, 1=definitely not, 0 = insufficient information or don't know

Briefly describe the mistake or problem and how it happened. *"Caries, cavities and problem with crown not diagnosed or treated"*

Could the mistake or problem have been avoided? If so how? *"Better dentist & not working to tight time-scale imposed by company owning dental surgery"*

Were you able to talk about the mistake or problem with anybody working in the primary care service? *"No I could not find anybody with whom I could discuss the mistake or problem"*

Patient-reported prospect of harm: prompted via Q10 (Box 1 main paper)

Patient-perspective problem-type code: C3. Problem with dental treatment or diagnosis

Scenario65. GP Surgery

Briefly describe the mistake or problem and how it happened. *"Using the summary on discharge from hospital, one GP transcribed incorrectly on to my electronic notes ie size of ovarian cyst was 7.5cms and he put 7.5 mms. Another GP requested diagnostic bone density scan but either forgot or did not record it and she ended up questioning why I had it and who requested it. She also referred me for an orthopedic consultation then said I was not funded for the steroid injection put into my swollen elbows."*

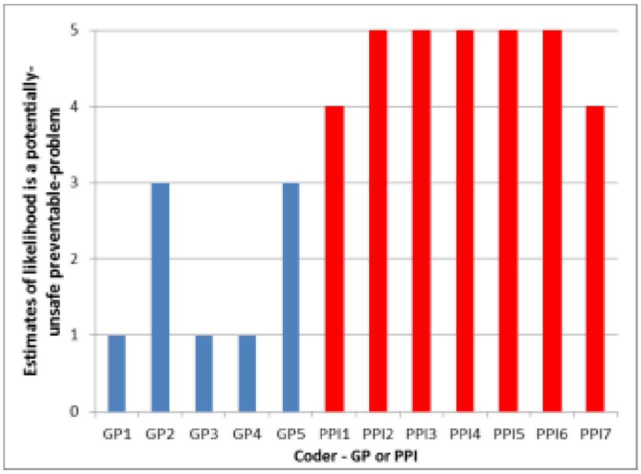

5=very likely or certain, 4=probably, 3=possibly, 2=unlikely, 1=definitely not, 0 = insufficient information or don't know

Could the mistake or problem have been avoided? If so how? *"Yes"*

Were you able to talk about the mistake or problem with anybody working in the primary care service? *"I was too scared to discuss my concerns for fear of being labelled a trouble maker"*

Patient-reported prospect of harm: health could have been made worse had someone not noticed a problem or error

Patient-perspective problem-type code: A2. Incorrect notes/inadequate notes/notes not kept up to date

Scenario66. GP Surgery

Briefly describe the mistake or problem and how it happened. *"GP prescribed pills, but then got phone call saying not to take them"*

Could the mistake or problem have been avoided? If so how? *"Not sure"*

Were you able to talk about the mistake or problem with anybody working in the primary care service? *"No I was not concerned about the problem"*

Patient-reported prospect of harm: prompted via Q10 (Box 1 main paper)

Patient-perspective problem-type code: C1. Medication problem

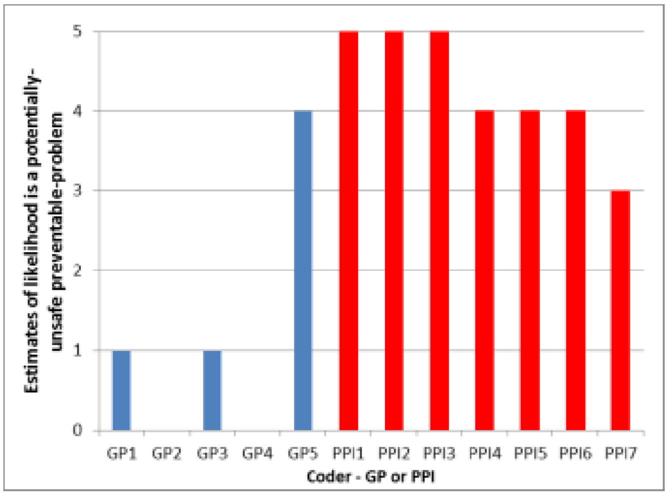

5=very likely or certain, 4=probably, 3=possibly, 2=unlikely, 1=definitely not, 0 = insufficient information or don't know

Scenario67. GP Surgery

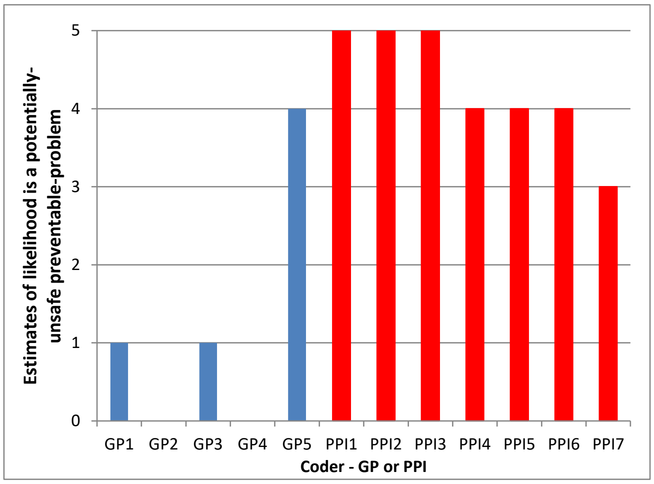

5=very likely or certain, 4=probably, 3=possibly, 2=unlikely, 1=definitely not, 0 = insufficient information or don't know

Briefly describe the mistake or problem and how it happened. *"I had a burst appendix and peritonitis, something that even a scan couldn't detect adequately. My first visit to GP was when I said I think I have appendicitis, no other symptoms only the pain. It was ten days before seeing a consultant, a further 10 days to have a scan, then 2 weeks to be told that I had a lump on my colon which is what my GP had said 5 weeks previously. It was a further 2 weeks before I had surgery."*

Could the mistake or problem have been avoided? If so how? *"If my GP had referred me for a scan immediately it would have saved 3 weeks out of the seven. It was two weeks from scan to results and I hear that is usual, but they're not looking at them for 2 weeks"*

Were you able to talk about the mistake or problem with anybody working in the primary care service? *"Had the outcome been different my widow might have pursued the matter further. The system is at fault rather than any individual."*

Patient-reported prospect of harm: your health has been made worse by a problem or error that could have been prevented

Patient-perspective problem-type code: B5. Not referred when patient felt was needed

<u>Patient reported scenarios occurring during the past 12 months that clinicians scored as definitely not a potentially-unsafe preventable-problem in primary care</u>

Scenario68: GP surgery
Description of event: Surgery arranged visits to cytology department at a local hospital; surgery did not ensure accurate visiting times came to patient
How could it be prevented: better communication between surgery and hospital
Were you able to talk about the problem or error with anybody working in the primary care service? deputy practice manager of GP surgery

Scenario69: GP surgery
Description of event: Given some medication that brought about a nervous breakdown and crisis team attended within 4 hours. Seeing mental health social worker each week now as a result. Hearing voices and seeing things which I didn't before this medication.
How could it be prevented: GP could have listened more carefully and not changed my medication.
Were you able to talk about the problem or error with anybody working in the primary care service? the crisis mental health team/the psychologist and social worker

For peer review only

Scenario70: Out of hours care

Description of event: Needed medication for vertigo but out of hours service sent me to A and E thinking I had had a stroke. Had all investigations for stroke over 4 hours, only for conclusion that it was indeed vertigo.

How could it be prevented: Could have ignored their pathway and had more clinical reasoning at the outset.

Were you able to talk about the problem or error with anybody working in the primary care service? No, once on the pathway you have to continue with it – no point in questioning

Scenario71: GP surgery

Description of event: mental health situation

How could it be prevented: doctor seemed unaware and worsened the condition

Were you able to talk about the problem or error with anybody working in the primary care service? attended A&E which got the doctor re-involved

Scenario72: GP surgery

Description of event: problem with process of obtaining blood test results. Lack of information and no communication

How could it be prevented: better communication

Were you able to talk about the problem or error with anybody working in the primary care service? I could not find anybody with whom I could discuss the problem or error

Scenario73: GP surgery

Description of event: I suspected I was told lies about what was on my record

How could it be prevented: My hunch is in the previous practice I belonged to someone was making up information to hit targets by saying I had test I hadn't had

Were you able to talk about the problem or error with anybody working in the primary care service? GP, it made me doubt my own sanity.

Scenario74: walk in clinic

Description of event: waiting time made the problem worse

How could it be prevented: shorter wait

Were you able to talk about the problem or error with anybody working in the primary care service? I was too distressed to discuss the problem or error

Scenario75: Dental/GP surgery

Description of event: A lump in the mouth resulted in me being referred to as out-patient at hospital. A biopsy was taken and then another was taken from the outside. Nothing has happened since then although I now have an indentation on my face. Referred back to my doctor still awaiting remedial treatment.

How could it be prevented: By my dentist who surely could have treated me properly.

Were you able to talk about the problem or error with anybody working in the primary care service? At the hospital I spoke to a consultant who kept referring to his team. The same thing happened at my doctors. It seems that no one will accept responsibility for the problem caused.

For peer review only

Scenario76: Dental surgery

Description of event: The dentist I was seeing had a plan for my treatment but the dentist who replaced her said the plan was "rubbish" and that I had to have private treatment. I had prepared myself for treatment according to the agreed plan but the new dentist tried to persuade me to spend £5000 on private treatment. As a result the dental treatment I need has not been done on the NHS and I have to find another dentist.

How could it be prevented: The problem was that my original dentist who I was happy with moved to the private sector within the same surgery

Were you able to talk about the problem or error with anybody working in the primary care service? I was too distressed to discuss the problem or error

Scenario77:GP surgery

Description of event: attempting to get routine screening and not being offered a convenient time as there is only a 2 week window

How could it be prevented: longer time scales and more choice over appointments

Were you able to talk about the problem or error with anybody working in the primary care service? it would require enormous effort and it was too time consuming to speak to someone

Scenario78: GP surgery

Description of event: Acne around eyes. Wanted dermatologist appointment which was not granted.

How could it be prevented: GP said only if the condition worsened.

Were you able to talk about the problem or error with anybody working in the primary care service? GP

Scenario79: GP surgery

Description of event: Doctor called me fat.

How could it be prevented: Yes, by better communication.

Were you able to talk about the problem or error with anybody working in the primary care service? I was too distressed to discuss the problem or error

Scenario80: GP surgery

Description of event: Six months ago I was referred by my GP to go for breast cancer screening for all women over 50. Since then I have not received the results of the test. I did not have any further contact so I called to check the result and was told it was with your GP. I called the GP and was told they had sent results to my home but I have not received it and six months on I have not heard.

How could it be prevented: I expected a sooner response or immediate response from the GP whatever the results but have had none I expect to call again tomorrow.

Were you able to talk about the problem or error with anybody working in the primary care service? I could not find anybody with whom I could discuss the problem or error

For peer review only

STROBE Statement—Checklist of items that should be included in reports of ***cross-sectional studies***

	Item No	Recommendation
**Title and abstract**	1	(*a*) Indicate the study's design with a commonly used term in the title or the abstract Yes p1
		(*b*) Provide in the abstract an informative and balanced summary of what was done and what was found yes p3
**Introduction**		
Background/rationale	2	Explain the scientific background and rationale for the investigation being reported Yes p4
Objectives	3	State specific objectives, including any prespecified hypotheses yes p4-5
**Methods**		
Study design	4	Present key elements of study design early in the paper yes p5
Setting	5	Describe the setting, locations, and relevant dates, including periods of recruitment, exposure, follow-up, and data collection yes p5
Participants	6	(*a*) Give the eligibility criteria, and the sources and methods of selection of participants yes p5
Variables	7	Clearly define all outcomes, exposures, predictors, potential confounders, and effect modifiers. Give diagnostic criteria, if applicable yes box1, online appendix 1
Data sources/ measurement	8*	For each variable of interest, give sources of data and details of methods of assessment (measurement). Describe comparability of assessment methods if there is more than one group yes p5, online appendix 1
Bias	9	Describe any efforts to address potential sources of bias yes p5 and reference 23
Study size	10	Explain how the study size was arrived at n/a power calculation described in protocol in terms of confidence intervals for generalisability to UK population but sample size was determined for practical reasons as is a descriptive analysis.
Quantitative variables	11	Explain how quantitative variables were handled in the analyses. If applicable, describe which groupings were chosen and why yes p6-7
Statistical methods	12	(*a*) Describe all statistical methods, including those used to control for confounding yes p5
		(*b*) Describe any methods used to examine subgroups and interactions, yes just chi2 tests p5
		(*c*) Explain how missing data were addressed all missing data is listed in the tables so it is completely transparent how this was dealt with, there were few missing data
		(*d*) If applicable, describe analytical methods taking account of sampling strategy the unweighted sample was used. This is not discussed as the difference was very small and adds much complexity without adding important information.
		(*e*) Describe any sensitivity analyses none done
**Results**		
Participants	13*	(a) Report numbers of individuals at each stage of study—eg numbers potentially eligible, examined for eligibility, confirmed eligible, included in the study, completing follow-up, and analysed yes online appendix 3
		(b) Give reasons for non-participation at each stage yes online appendix 3
		(c) Consider use of a flow diagram yes online appendix 3
Descriptive data	14*	(a) Give characteristics of study participants (eg demographic, clinical, social) and information on exposures and potential confounders yes table 1
		(b) Indicate number of participants with missing data for each variable of interest yes

For peer review only

For peer review only - http://bmjopen.bmj.com/site/about/guidelines.xhtml

		all tables
Outcome data	15*	Report numbers of outcome events or summary measures yes all tables
Main results	16	(*a*) Give unadjusted estimates and, if applicable, confounder-adjusted estimates and their precision (eg, 95% confidence interval). Make clear which confounders were adjusted for and why they were included yes table 3
		(*b*) Report category boundaries when continuous variables were categorized yes all tables
		(*c*) If relevant, consider translating estimates of relative risk into absolute risk for a meaningful time period yes p9
Other analyses	17	Report other analyses done—eg analyses of subgroups and interactions, and sensitivity analyses table 6 considers demographics for problems more likely to be a potentially harmful.
**Discussion**		
Key results	18	Summarise key results with reference to study objectives yes p9
Limitations	19	Discuss limitations of the study, taking into account sources of potential bias or imprecision. Discuss both direction and magnitude of any potential bias yes p11
Interpretation	20	Give a cautious overall interpretation of results considering objectives, limitations, multiplicity of analyses, results from similar studies, and other relevant evidence yes p11-12
Generalisability	21	Discuss the generalisability (external validity) of the study results yes p10
**Other information**		
Funding	22	Give the source of funding and the role of the funders for the present study and, if applicable, for the original study on which the present article is based yes p13

*Give information separately for exposed and unexposed groups.

Note: An Explanation and Elaboration article discusses each checklist item and gives methodological background and published examples of transparent reporting. The STROBE checklist is best used in conjunction with this article (freely available on the Web sites of PLoS Medicine at http://www.plosmedicine.org/, Annals of Internal Medicine at http://www.annals.org/, and Epidemiology at http://www.epidem.com/). Information on the STROBE Initiative is available at www.strobe-statement.org.

For peer review only

For peer review only - http://bmjopen.bmj.com/site/about/guidelines.xhtml

BMJ Open

The frequency and nature of potentially-harmful preventable-problems in primary care from the patient's perspective with clinician review – a population level survey in Great Britain

Journal:	*BMJ Open*
Manuscript ID	bmjopen-2017-020952.R2
Article Type:	Research
Date Submitted by the Author:	27-Apr-2018
Complete List of Authors:	Stocks, Susan; University of Manchester, GM Patient Safety Translational Research Centre<br>Donnelly, Ailsa; Research User Group (RUG) of the NIHR Greater Manchester Primary Care Patient Safety Translational Research Centre<br>Esmail, Aneez; University of Manchester, Primary Care Research Group<br>Beresford, Joanne; Research User Group (RUG) of the NIHR Greater Manchester Primary Care Patient Safety Translational Research Centre<br>Luty, Sarah; NHS Greater Glasgow and Clyde<br>Deacon, Richard; St Gabriels Medical Centre<br>Danczak, Avril; University of Manchester, Central and South Manchester Specialty Training Programme for General Practice<br>Mann, Nicola; Research User Group (RUG) of the NIHR Greater Manchester Primary Care Patient Safety Translational Research Centre<br>Townsend, David; Research User Group (RUG) of the NIHR Greater Manchester Primary Care Patient Safety Translational Research Centre<br>Ashley, James; University of Manchester, Division of Dentistry<br>Gamble, Carolyn; Research User Group (RUG) of the NIHR Greater Manchester Primary Care Patient Safety Translational Research Centre<br>Bowie, Paul; GP Section, NHS Education for Scotland<br>Campbell, Stephen; University of Manchester, Centre for Primary Care
**Primary Subject Heading**:	General practice / Family practice
Secondary Subject Heading:	Health services research
Keywords:	PRIMARY CARE, Quality in health care < HEALTH SERVICES ADMINISTRATION & MANAGEMENT, SOCIAL MEDICINE

SCHOLARONE™
Manuscripts

The frequency and nature of potentially-harmful preventable-problems in primary care from the patient's perspective with clinician review – a population level survey in Great Britain

S Jill Stocks *research fellow*[1], Ailsa Donnelly *public and patient involvement partner*[2], Aneez Esmail *professor*[3], Joanne Beresford *public and patient involvement partner*[2], Sarah Luty *general practitioner*[4], Richard Deacon *general practitioner*[5], Avril Danczak *general practitioner*[6], Nicola Mann *general practitioner*[2], David Townsend *general practitioner*[2], James Ashley *general dental practitioner*[7], Carolyn Gamble *public and patient involvement partner*[2], Paul Bowie *programme director*[8,9], Stephen M Campbell *professor*[3]

1. Centre for Epidemiology, Division of Population Health, Health Services Research and Primary Care, University of Manchester, Manchester M13 9PL, UK
2. Research User Group (RUG) of the NIHR Greater Manchester Primary Care Patient Safety Translational Research Centre, Centre for Primary Care, Division of Population Health, Health Services Research and Primary Care, University of Manchester, Manchester M13 9PL, UK
3. NIHR Greater Manchester Primary Care Patient Safety Translational Research Centre, Centre for Primary Care, Division of Population Health, Health Services Research and Primary Care, University of Manchester, Manchester M13 9PL, UK
4. General Practitioner NHS Greater Glasgow and Clyde, Medical Directorate, NHS Education for Scotland, Glasgow G3 8BW
5. St Gabriels Medical Centre, 4, Bishops Road, Prestwich, Manchester M25 0HT, UK
6. Central and South Manchester Specialty Training Programme for General Practice, Health Education England North West (HEENWE) Education and Research Centre, Wythenshawe Hospital, Manchester M23 9LT, UK
7. Woodlands Dental Practice, Rock Ferry, Wirral, CH42 4NG, UK
8. Medical Directorate, NHS Education for Scotland, 2 Central Quay, Glasgow G3 8BW, UK.
9. Institute of Health and Wellbeing, University of Glasgow, Glasgow G12 0XH, UK

Correspondence to Jill Stocks, Centre for Epidemiology, Division of Population Health, Health Services Research and Primary Care, University of Manchester, Manchester M13 9PL, UK
Email: sjstocks@btinternet.com
Tel: 01612752375
Fax: 0161 275 7600

Word count: 4865

For peer review only

For peer review only - http://bmjopen.bmj.com/site/about/guidelines.xhtml

Abstract

Objectives: To estimate the frequency of patient-perceived potentially-harmful problems occurring in primary care. To describe the type of problem, patient predictors of perceiving a problem, the primary care service involved, how the problem was discussed and patient suggestions as to how the problem might have been prevented. To describe clinician/public opinions regarding the likelihood that the patient-described scenario is potentially-harmful.

Design: population level survey

Setting: Great Britain

Participants: A nationally representative sample of 3975 members of the public aged 15 years or older interviewed during April 2016

Main outcome measures: counts of patient-perceived potentially-harmful problems in the last 12 months, descriptions of patient-described scenarios and review by clinicians/members of the public

Results:

3975 of 3996 participants in a nationally-representative survey completed the relevant questions (99.5%). 300 (7.6%; 95% confidence intervals 6.7% to 8.4%) of respondents reported experiencing a potentially-harmful preventable-problem in primary care during the past 12 months and 145 (48%) discussed their concerns within primary care. This did not vary with age, gender or type of service used. A substantial minority (30%) of the patient-perceived problems occurred outside general practice, particularly the dental surgery, walk in clinic, out of hours care and pharmacy. Patients perceiving a potentially-harmful preventable-problem were 8 times more likely to have "no confidence and trust in primary care" compared with "yes, definitely" (odds ratio 7.9; 5.9 to 10.7) but those who discussed their perceived-problem appeared to maintain higher trust and confidence. Generally clinicians ranked the patient-described scenarios as unlikely to be potentially harmful.

Conclusions: this study highlights the importance of actively soliciting patient's views about preventable harm in primary care as patients frequently perceive potentially-harmful preventable-problems and make useful suggestions for their prevention. Such engagement may also help to improve confidence and trust in primary care.

Strengths and limitations of this study

- We used a questionnaire co-designed with members of the public to quantify and describe patient-perceived potentially-harmful preventable-problems in primary care.

- The survey population was drawn from randomly-selected group of addresses to give a representative sample of the GB population.

- The potentially-harmful preventable-problems were self-reported by the survey respondents but primary care clinicians and members of the public estimated the likelihood that, in their opinion, each patient-described scenario was a potentially-harmful preventable-problem.

For peer review only

For peer review only - http://bmjopen.bmj.com/site/about/guidelines.xhtml

Background

Patients and clinicians view safety differently; patients tend to consider both serious safety problems as well as lesser causes of distress as safety concerns.(1) Patients judge quality and safety of care in terms of the ongoing care they receive over time whereas healthcare professionals may take the view that they provide high quality healthcare occasionally punctuated by discrete safety incidents and adverse events.(2) Even so patients can report medical errors accurately (3, 4) but they may have different priorities to professionals *e.g.* prioritising psychological and emotional harm over technical errors.(5) Given these differences the patient's approach to preventing safety problems may differ from clinicians, particularly if they believe clinicians to be responsible for the problem rather than the institutional system.(6, 7) Patient safety in primary care is rarely evaluated from the patient's perspective (8) whereas involving patients in identifying errors and reducing harm is common in secondary care.(3,9-11) A more participatory role for patients is advocated as a way to improve safety (12) suggesting a need for patients and professionals to be cognisant of each other's expectations and understanding of safety.

Estimates of the frequency of patient safety problems in primary care are generally from the clinician's perspective and range from less than 1 to 24 per 100 consultations or record review.(13-15) Some studies have quantified patient safety problems in primary care from the patient's perspective (6, 7, 16-18) However, quantitative patient-reported data from the UK is sparse; this may be partly due to the lack of a valid and reliable instrument for measuring safety in primary care from the patient's perspective.(19) The National Reporting and Learning System (NRLS) in England and Wales is a voluntary reporting scheme for NHS staff to report patient safety incidents. Less than 1% of reports originate from primary care (20), probably reflecting under-reporting. Until recently patients could not make reports directly to the NRLS. (21, 22) A European survey in 2013 found that 43% of UK respondents felt that it was "likely" that patients could be harmed by non-hospital healthcare and a recent survey of the UK public found that 21% of respondents reported experiencing a potentially-harmful preventable-problem in primary care within the past 12 months. (23, 24) These surveys suggest large differences between patients and clinicians in their beliefs about potentially-harmful problems in primary care, but this has not been examined at the population level. The PREOS-PC questionnaire has reported qualitatively on patient perceptions of safety in English general practices finding that patient recommendations for safer health care included improvements in patient- centred communication, continuity of care, timely appointments, technical quality of care, active monitoring, teamwork, health records and practice environment.(25, 26)

We aimed to quantify and describe patient-perceived potentially-harmful preventable-problems occurring in UK primary care. We also wanted to explore the differences in opinion between primary care professionals and the public regarding the potential for harm in the patient-described scenarios. Our approach aimed to capture the true patient perspective through extensive public and patient involvement (PPI); the study was conceived, co-designed and implemented by a team of three members of the public and one researcher.(24) The primary aims of the study were to estimate the annual and three year frequency of patient-reported potentially-harmful preventable-problems occurring in primary care as described by patients and describe the type of problem. The secondary aims were to identify patient predictors of reporting a problem (*e.g.* age, gender, social

For peer review only - http://bmjopen.bmj.com/site/about/guidelines.xhtml

class, income, employment status, ethnicity, to describe the primary care service involved), how the problem was discussed (if it was), patient suggestions as to how it might have been prevented and the variation in opinion between the reporting patient, other members of the public and clinicians in their opinion as to the likelihood the patient-described scenario is a potentially-harmful preventable-problem.

Methods

The population level survey

A survey asking about potentially-harmful preventable-problems occurring in primary care has been designed and piloted with extensive PPI as described in detail elsewhere. (24) The questions from this survey (Box 1, online Appendix 1) were embedded in to the Ipsos MORI GB Face to Face Omnibus (f2f Omnibus, a weekly survey that is used to track British attitudes to issues facing the country). It was used to survey a nationally and regionally representative sample of 4000 adults aged 15 or over living in private households in Great Britain between 8th and 21st April 2016 using a random sampling design described elsewhere.(27) Briefly 170-180 geographically representative sampling points were randomly selected and interviewers were required to get the interviews from a small group of streets reflecting that sampling point. (Typically an interviewer would get a completed interview from 1 in every 10 to 12 addresses.) The sample size was loosely based on the pilot study (24) which had found that 132/638 (21%) of self-selected respondents had perceived a potentially-harmful preventable-problem (although we anticipated a lower proportion when sampling from the general population). The f2f Omnibus consists of interviews in the participant's home using computer assisted personal interviewing, participation is completely voluntary and there are no incentives to take part. Respondents are free to refuse to answer any questions. The first question (Q1 Box 1) was taken from the English GP patient survey in order to compare the overall level of confidence and trust in their GP among the survey respondents with the larger sample used in the English GP patient survey.(28) The second question (Q2 Box 1) is the main screening question, those responding negatively to Q2 (*i.e.* not experienced a preventable-problem) were directed to a more specific question with a list of commonly understood patient safety events (Q10 Box 1 & online Appendix 1). If this prompted recognition of experiencing a potentially-harmful preventable-problem they were returned to Q4 (Box1). The intention of using a non-leading screening question was to encourage respondents to express their own perspective on what constitutes potentially-harmful preventable-problem rather than being directed towards existing definitions.

Coding of patient-reported scenarios

The nature of the problem described by the patient was coded at face value *i.e.* as the patient described without further interpretation, by one author (SJS) and checked by a second author (JA for dental scenarios, PB for all other scenarios) using a taxonomy developed during the pilot study that also mapped on to a previously published taxonomy for errors in general practice (24, 29, 30) (Table A, online Appendix 1). The medication-related scenarios were coded to a finer level (Table B, online Appendix 1).

Likelihood the scenario described a potentially-harmful preventable-problem

Five GPs, one general dental practitioner and 7 members of the public estimated the likelihood that, in their opinion, each patient-described scenario was a potentially-harmful preventable-problem.(24) The dental scenarios were only rated by the general dental practitioner and members

For peer review only - http://bmjopen.bmj.com/site/about/guidelines.xhtml

of the public. The raters were given the responses to Q2 and Q4 to Q9 (Box1) without any demographic information and asked to score each scenario on a 5 point scale from "very likely or certain" to "definitely not" a potentially-harmful preventable-problem. The scores were used to categorise the scenarios in to two groups according to the public or clinician-estimated likelihoods that they were a potentially-harmful preventable-problem as below. This is described in detail in Table C in online Appendix 1 and individual coding is shown in online Appendix 2.

- Group 1: patient-described scenarios with higher threshold as to likelihood of potential harm; Median score of "very likely or certain" or "probably" or at least one person gave a score of "very likely or certain"
- Group 2: patient-described scenarios with lower threshold as to likelihood of potential harm; Median score of "possibly" or at least one person gave a score of "probably" or higher
- All other scenarios – Median score below 3 ("possibly") and zero scores above 3 ("possibly")

The median scores excluded responses where the raters scored "don't know" or "insufficient information". We combined all the patient-described scenarios occurring in the last 3 years with scenarios from the pilot study (24) occurring in the last 12 months. We judged this acceptable since we were using the scenarios to compare the views of the clinicians and members of the public without making any inference to the wider population.

<u>Statistical analysis</u>

The 95% confidence intervals for the population means were calculated assuming a normal distribution for the sample mean. Simple cross tabulations were used to describe the data and a binary logistic regression model was used to explore whether particular types of patient (*e.g.* according to their demographics or surveyed opinions) were more likely to perceive a potentially-harmful preventable-problems and what type of scenario was more likely to be ranked as potentially harmful by clinicians and members of the public. Comparisons between demographics and outcomes for the respondents and the UK population were made using a χ^2 test. Inter-rater agreement for the ranking of the patient-described scenarios by clinicians and members of the public was assessed using a two-way random effects model single-measures intraclass correlation coefficient (ICC).(31). All analyses were done using Stata 14.

<u>Public and Patient Involvement (PPI)</u>

PPI was central to this co-designed survey and was provided through the Greater Manchester Primary Care Patient Safety Translational Research Centre Research User Group and other PPI networks (24). The study was conceived, designed, implemented and analysed by a team of three members of the public (AD, CG, JB) and one researcher (SJS). The piloting of the survey was through existing PPI networks (24). The scoring of the questions as to the likelihood they described a potentially-harmful preventable-problem was undertaken by 7 members of the public, 2 of whom had no previous experience in PPI. These findings will be disseminated to all the PPI groups that contributed to the pilot study and the authors will forward these results to their personal contacts who contributed to the questionnaire design.

<u>Results</u>

For peer review only - http://bmjopen.bmj.com/site/about/guidelines.xhtml

Of 3996 members of the public participating in the f2f Omnibus, 3984 (99.7%) agreed to complete the questions relevant to this study and 3975 (99.5%) actually completed all the questions. Survey responders were broadly representative of the GB population but were significantly more likely to have confidence and trust in the GP seen at their last appointment than the English population (Table D, online Appendix 1) although there was no significant difference when the graded responses "yes definitely" or "yes to some extent" were combined (91% vs 92%, $P(\chi^2)=0.2$).

The progress of the respondents through the analysis is summarised in Figures A & B in online Appendix 1. In total 300 (7.6%) of respondents reported experiencing a potentially-harmful preventable-problem during the past 12 months; of these 193 (4.9%) arose directly from the screening question (Q2 Box1) and 107 (2.7%) were prompted by a list of potentially-harmful preventable-problems (Q10 Box 1, Appendix 1). Of the 193 unprompted problems (Q2 Box 1), 119 (3.0%) patients suspected, or actually believed, that their health had been made worse as a result of the problem whereas 74 (1.9%) believed that they had either noticed the problem before it had any consequences or it had had no effect on their health. A further 132 potentially-harmful preventable-problems were reported as occurring within the past 1 to 3 years (Fig A, Appendix 1) making a 3 year total of 325 (8.2%) arising only from the screening question (Q2 Box1) as there was no prompt question (Q10, Box 1) asking about problems over 12 months ago. The combination of an open-ended question (Q2, Box 1) and prompt question (Q10, Box 1) prioritised sensitivity over specificity (as intended) given that 21% of the reported problems (79/379) were excluded from being a potentially-harmful preventable-problem in primary care by the respondent themselves by their response to questions 4 and 6 (*i.e.* not preventable or not in primary care, Box1).

Of the 300 patient-described scenarios occurring within the last 12 months, 93 (31%) were not ranked by any of the 6 clinicians mostly due to insufficient information (in the clinician's opinion). Of the 207 that were ranked by at least one clinician, 24 (11.6%, Table E, online Appendix 1) were considered to "at least probably" describe a potentially-harmful preventable-problem by clinicians (group 1 above). Group 2 (defined above) included 97 (46.9%) scenarios considered to "at least possibly" describe a potentially-harmful preventable-problem by clinicians. The members of the public ranked 116 (39%) scenarios occurring in the last 12 months as "at least probably" a potentially-harmful preventable-problem (group 1) and this included all 97 scenarios ranked as "at least possibly" by clinicians (group 2).

The proportion of respondents reporting a potentially-harmful preventable-problem within the last 12 months by respondent characteristics and unadjusted and adjusted odds ratios estimated by logistic regression are shown in Table 1. Those responding "no, not at all" to the question about trust and confidence in the GP (Q1 Box) were around eight times more likely to report a problem compared to those responding "yes, definitely"(Table 1). Women and rural dwellers were significantly more likely to report experiencing a potentially-harmful preventable-problem even when only including the scenarios judged to be more likely to be potentially-harmful by clinicians (Table 1). People not in employment due to a disability, self-employed or with one or more children were more likely to report a problem but not when only those scenarios judged to be more likely to be potentially-harmful by clinicians were included (Table 1).

Characteristics of the patient-reported scenarios

For peer review only - http://bmjopen.bmj.com/site/about/guidelines.xhtml

The types of problem occurring in the last 12 months alongside their clinician rankings are summarised in panel A, Figure 1. Generally respondents were equally likely to describe the nature of the problem as related to healthcare delivery, investigation, treatment (mainly medication), communication or lack of clinical knowledge or skills (panel B Fig 1). Within the medication problems the most common scenarios were being prescribed a wrong, contra-indicated or inappropriate drug or the wrong dose or delivery method (panel C Fig 1). The respondents did not identify any previously unreported types of problem and the patient-reported scenarios mapped well on to an established taxonomy of errors in primary care (Fig 1). However the prompt question (Q10) particularly increased reports of scenarios related to appointments, referrals and reporting of test results suggesting that the respondents did not consider these to be potentially harmful problems in the first instance (Fig C, online Appendix 1). Table 2 provides information about the patient's response to the potentially-harmful preventable-problem and the primary care service involved. A substantial minority (30%) of problems occurred outside general practice, particularly the dental surgery, walk in clinic, out of hours care and pharmacy. Around half of the patients had discussed their problem with a primary care professional and usually this was a person who worked in the same organisation as where their problem had occurred (Table 2). There were no significant differences between patients who discussed the problem, and those who did not, according to gender (males 49% vs females 51%, $P\chi^2$=0.78), age (38% to 62% in 10 year age bands, $P\chi^2$=0.33), type of service being used (general practice 50% vs other services 50%, $P\chi^2$=0.95), working as a healthcare professional (no 56% vs yes 50% $P\chi^2$=0.44) or describing a problem ranked higher by clinicians (below lower threshold 50% vs above lower threshold 50%, $P\chi^2$=0.98). Those reporting a problem in the first instance at Q2 (Box 1) without prompting were somewhat more likely to have discussed the problem (unprompted 53% vs prompted 43%, $P\chi^2$=0.08) whereas ethnic minorities were somewhat less likely to have discussed the problem (white 51% vs other ethnicity 37%, $P\chi^2$=0.09). Patients who discussed their problem were significantly more likely to "definitely" have trust and confidence in their GP (Q1 Box 1; 61% did discuss their problem vs 39% who did not discuss their problem, $P\chi^2$<0.001). The reasons given for not discussing the problem varied but the most common reasons related to feeling uncomfortable about discussing the problem, being too distressed or ill, being unable to find the appropriate person with whom to discuss the problem or the respondent was unconcerned about the problem. The respondent's suggestions as to how the problem might have been prevented are summarised in Table 3. The most frequent suggestions revolved around quicker access to primary care and investigations and a more participatory role. They rarely identified a particular individual as the problem or made specific suggestions for improvement strategies.

<u>Comparison of the opinions of clinicians and members of the public about the patient-reported scenarios</u>

The total number of patient-described scenarios available for analysis was 564 (432 from the main survey last 3 years and 132 from the pilot survey in last 12 months) but only 406 (72%) patients provided adequate information for at least one clinician to score the scenario on a 5 point scale as to the likelihood that the patient described a potentially-harmful preventable problem (Table C in online Appendix 1). The members of the public scored 426 (76%) of the scenarios. The median scores for each patient-described scenario are shown in Fig 2. Members of the public were significantly more likely to designate the patient-described scenarios as potentially-harmful preventable-problems compared with clinicians (median clinician score of 2.5, "unlikely- possibly" compared with members of the public score of 3.5, "possibly-probably"; Wilcoxon signed-rank test

For peer review only

z=16.4, P<0.001). From the clinician perspective just 8% of the problems occurring during the past 12 months were categorised as "probably to almost certainly" potentially harmful whereas for the members of the public the corresponding proportion was 39% (Table E in online Appendix 1 using the higher threshold). The individual patient-described scenarios scored by clinicians as more likely to be a potentially-harmful preventable-problems (median score is higher than "possibly" and scored by at least 2 clinicians, or one clinician scored "very likely or certain") and the scenarios with the greatest disagreement between members of the public and clinicians (median scores differ by 3 points or more on a 5 point scale) are summarised in online Appendix 2. The single measures ICC for absolute measures was 0.43 (0.38 to 0.49) for the members of the public and 0.23 (0.09 to 0.40) for clinicians, illustrating that members of the public had somewhat better agreement than clinicians. The associations between the characteristics of the patient or problem, and the clinician rankings of the likelihood it is a potentially-harmful preventable-problem are shown in Table F, online Appendix 1. Clinicians were more likely to rank scenarios as "possibly to almost certainly" potentially-harmful if they related to treatment, diagnosis or the patient was qualified as a healthcare professional (even though they were blind to this information) but for the members of the public scenarios related to treatment, investigation, clinical skills, diagnosis or where the patient had reported a problem in the first instance without prompting. Additionally members of the public were more likely to rank problems reported through the pilot survey as potentially harmful. Potentially-harmful preventable-problems involving cancer diagnoses or cardiovascular problems were more likely to be considered a potentially-harmful preventable-problem by both clinicians and members of the public compared with other diagnoses (as specified by the patient).

Discussion

Our main finding is that 7.6% of respondents in a GB nationally representative survey of 3975 people reported experiencing a potentially-harmful preventable-problem in primary care during the past 12 months. This is important, not only because patients may be experiencing genuine safety problems, but also because respondents perceiving a potentially-harmful preventable-problem were found to be eight times less likely to have confidence and trust in their GP (Table 1). Furthermore only around half of these patients perceiving a problem discussed their concern with a primary care professional. The implication is that many patient-perceived problems remain unknown to clinicians - scaling our results up to the GB adult population implies that around 3 million patients (3.8 million; 95% confidence intervals 3.3 million to 4.2 million) believe that they have experienced a potentially-harmful preventable-problem during the past 12 months and 1.5 million (1.2 million to 1.8 million) believe or suspect that their health has been made worse as a result. Clearly clinicians need to be aware of these patient-perceived preventable-problems where there is the potential for harm, but our findings also suggest that discussing such problems with the patient may also help to maintain confidence and trust in primary care among those who perceived a problem. (As this is a cross sectional study we cannot know whether the patients who discussed their problem did so because they already had a higher level of confidence and trust in their GP or discussing the problem contributed to the higher level of confidence and trust.) An accessible, informal route to actively engage and solicit patient's concerns about primary care may be helpful particularly given that the most common reasons patients gave for not discussing their problems are modifiable *e.g.* being unable to find the appropriate person or feeling uncomfortable about raising their concern and some were worried about the implications of doing so for their future care. Furthermore improving communication and patient involvement was one of the most frequently suggested strategies for

For peer review only - http://bmjopen.bmj.com/site/about/guidelines.xhtml

preventing the potentially-harmful preventable-problem (alongside quicker access to primary care and investigations). Other work suggested that patients are likely to blame individual clinicians for their perceived problem (7) but we did not particularly find this.

Our finding that around 30% of patient-perceived problems in primary care occurred outside general practice emphasizes the need for research in other areas of primary care, for example, 9% of the patient-perceived potentially-harmful preventable problems in the last 12 month occurred in dentistry in primary care (corresponding GB estimate 0.34 million; 0.21 million to 0.47 million) yet safety in this area remains largely unexplored.(32, 33)

Other studies have found differences between patients in perceiving mistakes or evaluating primary care services according to age, ethnicity, physical health and educational level (34) but we did not find this to be the case. We did find, however, that women, respondents with children, rural dwellers, and self-employed people or those not working due to disability were more likely to report a problem (Table 1). Some of these groups might be more frequent users of primary care; in the pilot study we observed that more frequent users of primary care were more likely to report experiencing a problem.(24) We also observed that respondents identifying with an ethnic minority group were less likely to discuss their problem with a member of primary care staff. Previous work in secondary care suggested that gender, educational level and employment status were associated with a patient's willingness to question healthcare staff.(35) Generally there were only small differences in demographics between patients in terms of being more or less likely to perceive, or discuss, a problem and it is important to consider each person's problem equally and encourage all groups, including minorities, to share their concerns.

We found that the survey respondents had similar views to clinicians and researchers in what constituted a potentially-harmful preventable problem given that the patient-described scenarios fit well in to a taxonomy designed and used by clinicians and researchers.(26, 29-30) We did not identify any new types of potentially-harmful preventable-problems unique to the patient perspective in primary care. Furthermore the clinicians and members of the public were consistent in which scenarios they ranked as more likely to be potentially harmful but patients have a much lower threshold for concern than clinicians e.g. just 8% of the 300 patient-reported scenarios were ranked by clinicians as "at least probably" a potentially-harmful preventable problem whereas for the members of the public it was 39%. While this may not be surprising it is important in the context of the discussion above. Clinicians may need to address patient-perceived problems that they do not believe to be harmful if they seek to improve public confidence and trust in primary care.

Strengths and weaknesses of the study

This large population level survey allowed for generalizable estimates of the frequency of patient-perceived potentially-harmful preventable-problems in primary care in GB for the first time and highlights that primary care clinicians tend to judge that the patient-perceived problems are unlikely to be potentially harmful. We have verified that our survey population is similar to the English population in terms of their confidence and trust in their GP as reported in the English GP Patient survey. Previous UK studies (26) have recruited through GP practices whereby patients may be reluctant to disclose problems or answer honestly in case of compromising the patient-clinician

For peer review only - http://bmjopen.bmj.com/site/about/guidelines.xhtml

relationship; indeed we report here that some patients did not wish to discuss their concern with primary care staff for this, and similar, reasons. Furthermore we believe that we have comprehensively captured the patient perspective through involving members of the public as research partners from study design through data acquisition to analysis and reporting. (24) We collected data related to problems occurring over the last 3 years and our denominator is patients not consultations. Time is an important tool for a primary care clinician but also problems arise over time, and the time of occurrence cannot always be assigned to a single consultation, especially with errors of omission that are associated with greater harm in primary care.(36). Reporting adverse events at a rate per consultation does not reflect the reality of the patient journey in primary care where the concept of patient safety as the management of risk over time fits well with the longer time scales.(2) The use of time in this way needs to be communicated to patients given that the most frequently suggested strategy for preventing the problem was quicker access to primary care including investigations (26%, Table 3).

The main weakness of the study is the relatively high proportion of scenarios that did not provide adequate information for ranking by clinicians (in their opinion). Arguably this would be improved by using a clinically trained interviewer but this could have biased the scenarios towards the clinician perspective and problems occurring outside of general practice might have gone unnoticed. Furthermore the cost of employing clinician interviewers would have been prohibitive for such a large scale survey. Ipsos MORI interviewers are accustomed to asking questions about healthcare; indeed they administer the annual GP patient survey.(28) Perhaps this could have been mitigated by using a more detailed questionnaire but the resources were not available and a longer questionnaire might have reduced the completion rate. A further weakness is that the patient suggestions regarding prevention tended to be non-specific. Collecting patients' suggestions about preventing harm was a secondary aim of this survey but patients did engage with the question and further work in partnership with clinicians is needed to develop this aspect of the survey further.

Strengths and weaknesses in relation to other studies

There are few studies undertaken from the patient perspective at the population level but the annual rates are similar to a Spanish study (7.6% vs 7%, 17). A Health Foundation research scan estimated a 1 to 2% adverse event rate per consultation (37) similar to our finding following clinician review (although we do not use consultations as the denominator). A face to face interview in family practice waiting rooms in the USA reported that 16% of respondents believed a physician had made a mistake in their care.(38) The types of problem and patient responses to the problem are similar to those that have been described qualitatively (1, 21, 39-40) but we have taken this further by using a well-defined denominator to quantify the frequency of occurrence and other descriptors of the problem from the patient's perspective.

Meaning of the study: possible explanations and implications for clinicians and policymakers

There are potentially a large number of patients in GB who believe they have experienced a potentially-harmful preventable problem in primary care but, based on the problems described by patients in this study, primary care clinicians rarely agree that these problems are likely to be potentially harmful. There are already many initiatives in UK primary care aiming to address patient

For peer review only - http://bmjopen.bmj.com/site/about/guidelines.xhtml

For peer review only

safety but how do we address the patient-perceived problems that clinicians do not recognise as potentially harmful? Similar differences have been observed in UK secondary care where staff measures of patient safety culture were not correlated with patient measures.(41) These differing views are likely to be multi-factorial in nature, for example perhaps clinicians are considering the problem from a medico-legal perspective or as a matter of allocation of limited resources *e.g.* disagreement about whether emotional discomfort or wasted time constitutes patient harm? (42) Conversely have the members of the public prioritised sensitivity over specificity or taken a more precautionary approach. Previous qualitative work has observed that, for patients, safety in primary care safety is contingent on the clinician patient relationship where among professionals the systems approach to patient safety is prevalent.(1) While reconciling the differing perspectives of patient and clinician may not be realisable, our study suggests that providing opportunities for, and encouraging, patients to discuss their concerns informally with a member of the primary care team may help with building trust, clarifying expectations and ensuring understanding. The patient suggestions for preventing their perceived problem seem to be asking for more patient centred care where healthcare is in partnership and patients are included in decisions.(43) Including patients more actively in healthcare may also help diminish the patient's expectations of certainty that seem to be common despite primary care being inherently uncertain.(44) Future work should focus on strategies to encourage patients and clinicians to work together to ensure that primary care not only is safe but is also perceived to be safe by patients.

Acknowledgements: The authors would like to express their thanks and appreciation for the work done by the Mary Aldred, Gitanjali Holt, Manoj Mistry, Carole Bennett and Lindsey Brown in coding the patient-described scenarios. Also thank you to the PPI groups who were involved in the piloting of the survey; HelpBeatDiabetes, The Primary Care Research in Manchester Engagement Resource, Associate Research User Group of the Greater Manchester Primary Care Patient Safety Translational Research Centre, The Nowgen Centre, The Citizen Scientist project and North West People in Research Forum. For more information see http://bmjopen.bmj.com/content/bmjopen/8/2/e017786.full.pdf

Contributors: SJS, AD, JB and CG conceived and designed the study. SJS, AD, JB, CG, AE, PB, JA, DT, SL, AD, RD and NM analysed the data. SJS wrote the manuscript, and is guarantor. AD, JB, CG, AE, PB, JA, DT, SL, AD, RD, NM and SC edited the manuscript.

Funding: This study was funded by the National Institute for Health Research (http://www.nihr.ac.uk) through the Greater Manchester Primary Care Patient Safety Translational Research Centre (NIHR GM PSTRC), grant number gmpstrc-2012-1. The views expressed are those of the author(s) and not necessarily those of the NHS, the NIHR or the Department of Health. The funders had no role in study design, data collection and analysis, decision to publish, or preparation of the manuscript.

Competing interests: All authors have completed the ICMJE uniform disclosure form at www.icmje.org/coi_disclosure.pdf (available on request from the corresponding author) and declare no support from any organisation for the submitted work; no financial relationships with any organisations that might have an interest in the submitted work in the previous three years, no other relationships or activities that could appear to have influenced the submitted work.

For peer review only - http://bmjopen.bmj.com/site/about/guidelines.xhtml

Ethical approval: University of Manchester Ethics Committee 2 Approval 15372. Respondents to the Ipsos MORI face to face omnibus are not asked to sign a consent document, the invitation into the house after agreement to take part in the survey is considered to be consent. All respondents were provided with the participant information sheet before completing the survey questions specific to this study which explains that participation is entirely voluntary and the participant may choose to stop answering the questions at any time.

Copyright statement: the Corresponding Author has the right to grant on behalf of all authors and does grant on behalf of all authors, an exclusive licence on a worldwide basis to the BMJ Publishing Group Ltd to permit this article (if accepted) to be published in BMJ editions and any other BMJPGL products and sublicences such use and exploit all subsidiary rights, as set out in our licence.

Transparency declaration: SJS affirms that this manuscript is an honest, accurate, and transparent account of the study being reported; that no important aspects of the study have been omitted; and that any discrepancies from the study as planned have been explained.

Data sharing: Raw data (coded only) is available from jill.stocks@manchester.ac.uk

Figure legends

Footnote to figure 1: See Tables A&B, online Appendix 1 for details of coding; A coded to 2 levels, B coded to 1 level, C medication problems coded to 3 levels

Fig 1. Numbers of patient-perceived problems occurring in the last 12 months categorised according to the patient’s description with clinician ranking as to the likelihood it is a potentially-harmful preventable problem (Table E, online Appendix 1).

Figure 2. Median clinician and members of the public estimates of the likelihood that the patient describes a potentially-harmful preventable-problem occurring in the last 12 months

For peer review only - http://bmjopen.bmj.com/site/about/guidelines.xhtml

Box 1. Brief summary of questionnaire – see online Appendix 1 for full version
Q1. Did you have confidence and trust in the GP you saw or spoke to at your last appointment? (benchmarking question)
Q2a. Have you experienced a situation with a primary care service where your health has ACTUALLY been made worse by a problem or error that could have been prevented?<br>Q2b. And have you experienced a situation with a primary care service where you SUSPECTED your health has been made worse by a problem or error that could have been prevented?<br>Q2c. And have you experienced a situation with a primary care service where your health could have been made worse had someone not NOTICED a problem or error?<br>Q2d. And have you experienced a situation with a primary care service where there was a problem or error that could have been prevented but it did not make your health worse?
If "yes" to more than one of Q2a-d ask Q2e to identify which happened most recently<br>*If "no" to Q2a-d go to Q11*
Q3. Thinking about the most recent occasion where you experienced a preventable problem or error caused by the primary care service, when did this occur?
Q4. Thinking about the most recent occasion, which primary care service were you using when the problem or error occurred?
Q5. Thinking about the most recent problem or error you experienced, can you briefly describe what it was and how it happened?
Q6. In your opinion, how, if at all, could the problem or error have been avoided?
Q7. Were you able to talk about the problem or error with anybody WORKING IN THE PRIMARY CARE SERVICE?
Q8. You said you were able to discuss the problem or error with somebody working in primary care. Please describe their job or role and their response.
Q9. Which of the following reasons, if any, best describes why you were unable to talk about the problem or error with somebody working in the primary care service?
Q10. In the last 12 months, have any of the following happened to you **while** using primary care, or not? *If yes go to Q4* (See online Appendix 1 for list of preventable problems)
Q11. Do you, personally, work as a Healthcare Professional in any capacity? For example, a doctor/nurse/therapist/pharmacist/other NHS staff, etc.

For peer review only - http://bmjopen.bmj.com/site/about/guidelines.xhtml

Table 1. Prevalence of respondents reporting a potentially-harmful preventable problem within the last 12 months and unadjusted and adjusted odds ratios estimated by logistic regression

Respondent characteristics (total) N=3984	Reported problem in last 12 months (%) n=300	Unadjusted OR–all reports	Adjusted[1] OR-all reports	Adjusted[1] OR after GP review (lower threshold[2]) n=97
**Gender (1 missing)**				
Male (1950)	111 (6%)	1 (ref)	1 (ref)	1 (ref)
Female (2033)	189 (9%)	1.7 (1.3 to 2.2)	1.7 (1.2 to 2.2)	2.3 (1.3 to 3.8)
**Age (years)**				
15 to 24 (533)	38 (7%)	1 (ref)	1 (ref)	1 (ref)
25 to 34 (573)	54 (9%)	1.4 (0.9 to 2.1)	0.7 (0.4 to 1.3)	0.4 (0.2 to 1.2)
35 to 44 (528)	30 (6%)	0.8 (0.5 to 1.3)	0.4 (0.2 to 0.8)	0.1 (0.0 to 0.6)
45 to 54 (629)	54 (9%)	1.2 (0.8 to 1.9)	0.7 (0.4 to 1.4)	0.5 (0.2 to 1.5)
55 to 64 (654)	60 (9%)	1.3 (0.9 to 2.0)	0.8 (0.4 to 1.6)	0.7 (0.2 to 2.0)
65 to 74 (609)	41 (7%)	0.9 (0.6 to 1.5)	0.5 (0.2 to 1.3)	0.7 (0.2 to 3.0)
75 or older (458)	23 (5%)	0.7 (0.4 to 1.2)	0.3 (0.1 to 0.9)	0.3 (0.1 to 1.9)
**Employment status (3 missing)**				
Paid job - full or part time (1719)	119 (7%)	1 (ref)	1 (ref)	1 (ref)
Full time student (283)	14 (5%)	0.7 (0.4 to 1.2)	0.4 (0.1 to 1.1)	0.4 (0.1 to 1.8)
Not working - long term illness/disability (133)	22 (17%)	2.7 (1.6 to 4.4)	2.3 (1.2 to 4.6)	0.9 (0.3 to 3.1)
Not working - other reason (267, includes unemployed)	24 (9%)	1.3 (0.8 to 2.1)	1.3 (0.7 to 2.4)	0.4 (0.1 to 1.4)
Not working - Housewife/husband (201)	19 (9%)	1.4 (0.8 to 2.3)	1.0 (0.5 to 2.0)	0.3 (0.1 to 1.2)
Retired (1198)	80 (7%)	1.0 (0.7 to 1.3)	1.4 (0.8 to 2.6)	0.5 (0.2 to 1.3)
Self-employed (180)	20 (11%)	1.7 (1.0 to 2.8)	2.0 (1.1 to 3.5)	0.5 (0.1 to 2.3)
**Region of domicile (23 missing)**				
Greater London (565)	38 (7%)	1 (ref)	1 (ref)	1 (ref)
East Midlands (262)	9 (3%)	0.5 (0.2 to 1.0)	0.6 (0.2 to 1.4)	0.4 (0.0 to 3.6)
East of England (425)	27 (6%)	0.9 (0.6 to 1.6)	0.6 (0.3 to 1.1)	1.8 (0.5 to 5.8)
North (176)	15 (9%)	1.3 (0.7 to 2.5)	0.8 (0.3 to 1.7)	0.7 (0.1 to 4.3)
North-West (490)	46 (9%)	1.4 (0.9 to 2.2)	1.0 (0.6 to 1.9)	1.4 (0.4 to 4.5)
Scotland (372)	27 (8%)	1.1 (0.7 to 1.8)	0.8 (0.4 to 1.6)	1.8 (0.5 to 6.1)
South East (444)	32 (7%)	1.1 (0.6 to 1.6)	1.1 (0.6 to 2.0)	2.2 (0.7 to 7.0)
South West (281)	33 (12%)	1.8 (1.1 to 3.0)	1.0 (0.5 to 2.0)	1.9 (0.5 to 6.6)
Wales (196)	15 (8%)	1.1 (0.6 to 2.1)	0.6 (0.3 to 1.4)	2.2 (0.5 to 8.5)
West Midlands (377)	19 (5%)	0.7 (0.4 to 1.3)	0.6 (0.3 to 1.3)	1.1 (0.3 to 4.4)
Yorks & Humberside (373)	39 (10%)	1.6 (1.0 to 2.6)	1.2 (0.7 to 2.3)	2.7 (0.8 to 8.4)
**Ethnicity (18 missing)**				
White (3591)	271 (8%)	1 (ref)	1 (ref)	1 (ref)
Other ethnicity (475)	26 (5%)	0.7 (0.5 to 1.0)	1.2 (0.7 to 2.2)	1.1 (0.4 to 3.0)
**Type of community**				
Urban, suburban (3051)	203 (7%)	1 (ref)	1 (ref)	1 (ref)
Rural (933)	97 (10%)	1.6 (1.3 to 2.1)	1.9 (1.3 to 2.7)	2.0 (1.1 to 3.5)
**Parental responsibility**				
Zero children under 19 (2839)	192 (7%)	1 (ref)	1 (ref)	1 (ref)
Child(ren) aged up to 19 (1145)	108 (9%)	1.4 (1.1 to 1.8)	1.2 (0.8 to 1.7)	1.5 (0.8 to 2.8)

For peer review only - http://bmjopen.bmj.com/site/about/guidelines.xhtml

**Tenure (31 missing)**				
Mortgaged (1042)	84 (8%)	1 (ref)	1 (ref)	1 (ref)
Owned outright (1441)	87 (6%)	0.7 (0.5 to 1.0)	0.8 (0.5 to 1.2)	0.9 (0.4 to 1.8)
Rented-housing association (301)	42 (14%)	1.8 (1.2 to 2.7)	1.3 (0.7 to 2.2)	1.1 (0.4 to 2.9)
Rented-private landlord (719)	49 (7%)	0.8 (0.6 to 1.2)	0.9 (0.6 to 1.5)	0.9 (0.4 to 2.1)
Rented-local authority (422)	31 (7%)	0.9 (0.6 to 1.4)	0.6 (0.3 to 1.2)	1.0 (0.4 to 2.8)
Other (28)	4 (14%)	1.9 (0.6 to 5.6)	2.2 (0.6 to 8.2)	-[3]
**Confidence and trust in GP at last appointment?**				
Yes definitely (3031)	144 (5%)	1 (ref)	-	-
Yes, to some extent (611)	68 (11%)	2.5 (1.9 to 3.4)	-	-
No, not at all (311)	88 (28%)	7.9 (5.9 to 10.7)	-	-
Don't know /can't say (31)	0 (0%)	-	-	-

[1]adjusted for gender, age, employment status, ethnicity, tenure, region of domicile, type of community, parental responsibility, highest level of education achieved, marital status, social grade, household income

[2]see Table E online Appendix 1

[3]zero problems in this category

For peer review only

For peer review only - http://bmjopen.bmj.com/site/about/guidelines.xhtml

Table 2. Details of the patient's response to the potentially-harmful preventable-problem and the primary care service involved

**Primary care service involved**	**Problems in last 12 months n=300**	**All problems analysed[1] n=564**
GP surgery	211 (70%)	395 (70%)
Dental surgery	27 (9%)	50 (9%)
Walk in clinic	16 (5%)	22 (4%)
Ambulance/A&E/ Out of hours care	16 (5%)	28 (5%)
Pharmacy	10 (3%)	19 (3%)
Community or district nursing	8 (3%)	21 (4%)
Mental health services	6 (1%)	8 (1%)
Opticians	4 (1%)	5 (1%)
Physiotherapy (in primary care)	2 (1%)	5 (1%)
missing /nk	0 (<1%)	11 (2%)
**Did you discuss the problem with primary care staff?**	**Problems in last 12 months n=300**	**All problems analysed[1] n=564**
Yes	145 (48%)	273 (48%)
No	153 (51%)	273 (48%)
missing /nk	2 (1%)	18 (3%)
**Reasons why patients did not discuss the problem with primary care staff**	**Problems in last 12 months n=153**	**All problems analysed[1] n=273**
Patient had the opportunity but did not feel comfortable discussing the problem or error	16 (10%)	43 (16%)
Patient could not find anybody with whom to discuss the problem or error	37 (24%)	75 (27%)
Patient was not concerned about the problem or error	25 (16%)	37 (14%)
Patient did not notice the problem or error or trusted the clinician's judgement at the time	11 (7%)	25 (9%)
Patient was too distressed or ill to discuss the problem or error	18 (12%)	30 (11%)
Other - problem was resolved in another way by the patient without involving primary care	10 (7%)	13 (5%)
Other - patient believed primary care staff would not be interested in the problem or would not take it seriously or it would not improve primary care	7 (5%)	14 (5%)
Other – patient believed that discussing the problem with a primary care staff might have negative implications for their future care	6 (4%)	6 (2%)
Other - patient did know that they were allowed to express an opinion or how to raise the problem	5 (3%)	5 (2%)
Other - patient accepts that such problems will arise in primary care or didn't want to use primary care resources when primary care staff are very busy	5 (3%)	6 (2%)
Other - patient intends to discuss with primary care	4 (3%)	6 (2%)

For peer review only

For peer review only - http://bmjopen.bmj.com/site/about/guidelines.xhtml

professional at the next opportunity		
Don't Know/missing	9 (6%)	13 (5%)
**Profession of discussant**	**Problems in last 12 months n=145**	**All problems analysed[1] n=273**
GP/practice nurse	66 (46%)	144 (53%)
Practice manager/receptionist/administrator	25 (17%)	39 (14%)
Pharmacist/dispenser	7 (5%)	14 (5%)
General Dental Practitioner	8 (6%)	18 (7%)
Hospital doctor or nurse/A&E or OOH staff/paramedic	15 (10%)	18 (7%)
Other primary care staff	14 (10%)	17 (6%)
PALS or NHS direct staff	1 (1%)	2 (1%)
Unclear, don't know or missing	9 (6%)	21 (8%)
**Role of discussant in patient's care**	**Problems in last 12 months n=145**	**All problems analysed[1] n=273**
Member of staff central to respondent's care	60 (41%)	112 (41%)
Member of staff in the same team or organisation	35 (24%)	84 (31%)
Member of staff in a different team or organisation	31 (21%)	40 (15%)
Role of member of staff is unclear	8 (6%)	20 (7%)
missing	11 (8%)	17 (%)

1

2 [1]All problems analysed includes scenarios arising from Ipsos MORI survey in the last 3 years and the pilot survey (24) within the last 12 months

For peer review only

1 Table 3. Patient suggestions as to how the potentially-harmful preventable problem might have
2 been prevented

How could it be prevented?	Problems in last 12 months n=300	All problems analysed[1] n=564
**1. More resources - total**	**100 (33%)**	**157 (28%)**
1.1 Quicker access to primary care	43 (14%)	62 (11%)
1.2 More thorough and quicker investigations	35 (12%)	59 (10%)
1.3 Fewer demands on primary care – more staff or fewer patients	7 (2%)	12 (2%)
1.4 More time with clinicians for treatment and diagnosis	8 (3%)	12 (2%)
1.5 Improved access to social care	3 (1%)	3 (1%)
1.6 More follow-up by primary care	2 (1%)	3 (1%)
1.7 Improved continuity of care	1 (<1%)	2 (<1%)
1.8 Access to a second opinion	1 (<1%)	2 (<1%)
1.9 Provision of resources to manage long term conditions	0	2 (<1%)
**2. Improved communication and involvement of patients - total**	**53 (18%)**	**92 (16%)**
1.1 Listen to the patient and trust their judgement more	36 (12%)	68 (12%)
1.2 Tell patients about their diagnosis, test results, changes in medication or loss of results	10 (3%)	15 (3%)
1.3 Improve communication between staff (within or outside primary care)	7 (2%)	9 (2%)
**3. Better organisation and administration - total**	**27 (9%)**	**48 (9%)**
3.1 Follow up referrals and appointments to ensure they happen, be consistent in sending routine reminders	12 (4%)	23 (4%)
3.2 Log in or process results as soon as received to avoid loss	5 (2%)	7 (1%)
3.3 Keep the notes up to date, well-organised, safe and ensure information is transcribed accurately	9 (3%)	15 (3%)
3.4 Keep a record of the location of equipment	0	1 (<1%)
3.5 Improve the method of appointment allocation	0	1 (<1%)
3.6 Fine patients for not attending appointments	1 (<1%)	1 (<1%)
**4. Improved prescribing systems - total**	**21 (7%)**	**45 (8%)**
4.1 More when checks on prescribing and dispensing	19 (6%)	32 (6%)
4.2 Check repeat prescriptions carefully, especially for transcribing errors	2 (1%)	10 (2%)
4.3 Use medication reviews and IT clinical decision support systems	0	3 (1%)
**5. Better clinical practice - total**	**17 (6%)**	**47 (8%)**
5.1 Take in to account all the patient's information - their medical history and results and letters	7 (2%)	27 (5%)
5.2 Address the patient's problem in some way – patients can feel their problem is being ignored	9 (3%)	18 (3%)
5.3 Act on advice from other clinicians and test results	1 (<1%)	2 (<1%)
**6. Staff training - total**	**22 (7%)**	**53 (9%)**
6.1 More informed and better trained staff	22 (7%)	53 (9%)

For peer review only - http://bmjopen.bmj.com/site/about/guidelines.xhtml

**Other responses - total**	**60 (20%)**	**122 (22%)**
•Don't know/missing	28 (9%)	64 (11%)
•Problem was due to an individual member of staff	6 (2%)	11 (2%)
•Do not make wrong, late, delayed diagnosis	7 (2%)	15 (3%)
•Prescribe right, better, different, more, less medicine	8 (3%)	15 (3%)
•Should have been referred	6 (2%)	9 (2%)
•Better organisation	3 (1%)	4 (1%)
•Patient recognised their own responsibility	2 (1%)	2 (<1%)
•Laboratory procedures were the problem	0	2 (<1%)

[1]All problems analysed includes scenarios arising from Ipsos MORI survey in the last 3 years and the pilot survey (24) within the last 12 months

For peer review only

For peer review only - http://bmjopen.bmj.com/site/about/guidelines.xhtml

References

1. Rhodes P, Campbell S, Sanders C. Trust, temporality and systems: how do patients understand patient safety in primary care? A qualitative study. Health Expectations. 2016;19(2):253-63.

2. Vincent C, Amalberti R. Safer Healthcare: Strategies for the Real World. Springer, 2016. http://www.springer.com/gb/book/9783319255576 Accessed 04/04/17

3. The Health Foundation. Evidence scan: Involving patients in improving safety. January 2013. http://www.health.org.uk/sites/health/files/InvolvingPatientsInImprovingSafety.pdf Accessed 04/04/17

4. King A, Daniels J, Lim J, Cochrane DD, Taylor A, Ansermino JM. Time to listen: a review of methods to solicit patient reports of adverse events. Qual Saf Health Care. 2010;19(2):148-57.

5. Kuzel AJ, Woolf SH, Gilchrist VJ, Engel JD, LaVeist TA, Vincent C, et al. Patient reports of preventable problems and harms in primary health care. Ann Fam Med. 2004;2(4):333-40.

6. Blendon RJ, DesRoches CM, Brodie M, Benson JM, Rosen AB, Schneider E, et al. Views of practicing physicians and the public on medical errors. N Engl J Med. 2002;347(24):1933-40.

7. Hotvedt R, Forde OH. Doctors are to blame for perceived medical adverse events. A cross sectional population study. The Tromso Study. BMC health services research. 2013;13:46.

8. The Health Foundation. Evidence Scan: Improving safety in primary care. November 2011. http://www.health.org.uk/sites/health/files/ImprovingSafetyInPrimaryCare.pdf Accessed 04/04/17

9. Weingart SN et al. RS What can hospitalized patients tell us about adverse events? Learning from patient-reported incidents. J Gen Intern Med. 2005 Sep;20(9):830-6.

10. Weissman JS, Schneider EC, Weingart SN, et al. Comparing patient-reported hospital adverse events with medical record review: do patients know something that hospitals do not? Ann Intern Med. 2008 Jul 15;149(2):100-8

11. Southwick FS, Cranley NM, Hallisy JA. A patient-initiated voluntary online survey of adverse medical events: the perspective of 696 injured patients and families. BMJ Qual Saf. 2015;24(10):620-9.

12. Longtin Y, Sax H, Leape LL, Sheridan SE, Donaldson L, Pittet D. Patient participation: current knowledge and applicability to patient safety. Mayo Clinic proceedings. 2010;85(1):53-62.

13. Sandars J, Esmail A. The frequency and nature of medical error in primary care: understanding the diversity across studies. Family practice. 2003;20(3):231-6.

14. Panesar SS, deSilva D, Carson-Stevens A, Cresswell KM, Salvilla SA, Slight SP, et al. How safe is primary care? A systematic review. BMJ Quality & Safety. 2016;25(7):544-53.

15. Michel P, Brami J, Chaneliere M, Kret M, Mosnier A, Dupie I, et al. Patient safety incidents are common in primary care: A national prospective active incident reporting survey. PLoS One. 2017;12(2):e0165455.

For peer review only - http://bmjopen.bmj.com/site/about/guidelines.xhtml

16. Northcott H, Vanderheyden L, Northcott J, Adair C, McBrien-Morrison C, Norton P, et al. Perceptions of preventable medical errors in Alberta, Canada. International journal for quality in health care : journal of the International Society for Quality in Health Care / ISQua. 2008;20(2):115-22.

17. Mira JJ, Nebot C, Lorenzo S, Perez-Jover V. Patient report on information given, consultation time and safety in primary care. Qual Saf Health Care. 2010;19(5):e33.

18. Wasson JH, MacKenzie TA, Hall M. Patients use an internet technology to report when things go wrong. Qual Saf Health Care. 2007;16(3):213-5.

19. Ricci-Cabello I, Goncalves DC, Rojas-Garcia A, Valderas JM. Measuring experiences and outcomes of patient safety in primary care: a systematic review of available instruments. Family practice. 2015;32(1):106-19.

20. Ricci-Cabello I, Avery AJ, Reeves D, Kadam UT, Valderas JM. Measuring Patient Safety in Primary Care: The Development and Validation of the "Patient Reported Experiences and Outcomes of Safety in Primary Care" (PREOS-PC). Ann Fam Med. 2016;14(3):253-61.

21. Ricci-Cabello I, Saletti-Cuesta L, Slight SP, Valderas JM. Identifying patient-centred recommendations for improving patient safety in General Practices in England: a qualitative content analysis of free-text responses using the Patient Reported Experiences and Outcomes of Safety in Primary Care (PREOS-PC) questionnaire. Health Expectations. 2017.

22. NHS England. General practice patient safety reporting form launched. 26th February 2015. https://www.england.nhs.uk/2015/02/gp-patient-safety-reporting/ Accessed 04/04/17

23. European Commission. Special Eurobarometer 411 Patient Safety and Quality of Care 2014. DOI:10.2772/33467 https://ec.europa.eu/health/sites/health/files/patient_safety/docs/ebs_411_sum_en.pdf Accessed 04/04/17

24. Stocks SJ, Donnelly A, Esmail A, Beresford J, Luty S, Deacon R, et al. Development and piloting of a survey to estimate the frequency and nature of potentially-harmful preventable-problems in primary care from a UK patient's perspective. BMJ Open. 2017;8:e017786. doi:10.1136/ bmjopen-2017-01778

25. NHS Improvement. National quarterly data on patient safety incident reports. September 2016. https://improvement.nhs.uk/resources/national-quarterly-data-patient-safety-incident-reports-september-2016/ Accessed 04/04/17

26. Hutchinson A, Young TA, Cooper KL, McIntosh A, Karnon JD, Scobie S, et al. Trends in healthcare incident reporting and relationship to safety and quality data in acute hospitals: results from the National Reporting and Learning System. Qual Saf Health Care. 2009;18(1):5-10.

27. Ipsos MORI Face-to-Face Omnibus (Capibus) https://www.ipsos-mori.com/ourexpertise/omnibusservices/capibus.aspx Accessed 04/04/17

28. NHS England. GP Patient Survey – National summary report. January 2016. http://gp-survey-production.s3.amazonaws.com/archive/2016/January/January+2016+National+Summary+Report.pdf Accessed 04/04/17

For peer review only - http://bmjopen.bmj.com/site/about/guidelines.xhtml

29. Dovey SM, Meyers DS, Phillips RL, Jr., Green LA, Fryer GE, Galliher JM, et al. A preliminary taxonomy of medical errors in family practice. Qual Saf Health Care. 2002;11(3):233-8.

30. Makeham MA, Dovey SM, County M, Kidd MR. An international taxonomy for errors in general practice: a pilot study. Med J Aust. 2002;177(2):68-72.

31. McGraw K WS. Forming inferences about some intraclass correlation coefficients. Psychological Methods. 1994;1(1):30-46.

32. Bailey E, Tickle M, Campbell S. Patient safety in primary care dentistry: where are we now? Br Dent J. 2014;217(7):339-44.

33. Ensaldo-Carrasco E, Suarez-Ortegon MF, Carson-Stevens A, Cresswell K, Bedi R, Sheikh A. Patient Safety Incidents and Adverse Events in Ambulatory Dental Care: A Systematic Scoping Review. Journal of patient safety. 2016;08:08.

34. Campbell JL, Ramsay J, Green J. Age, gender, socioeconomic, and ethnic differences in patients' assessments of primary health care. Quality in health care : QHC. 2001;10(2):90-5.

35. Davis RE, Koutantji M, Vincent CA. How willing are patients to question healthcare staff on issues related to the quality and safety of their healthcare? An exploratory study. Qual Saf Health Care. 2008;17(2):90-6.

36. The WHO Safer Primary Care Expert Working Group. Safer Primary Care: A Global Challenge. Summary of Inaugral Meeting 2012. http://www.who.int/patientsafety/summary_report_of_primary_care_consultation.pdf Accessed 04/04/17

37. The Health Foundation. Evidence Scan: Levels of Harm in Primary Care. November 2011. http://www.health.org.uk/sites/health/files/LevelsOfHarmInPrimaryCare.pdf Accessed 04/04/17

38. Kistler CE, Walter LC, Mitchell C, Sloane PD. Patient perceptions of mistakes in ambulatory care. Archives of Internal Medicine. 2010;170(16):1480-7.

39. Hernan AL, Giles SJ, Fuller J, Johnson JK, Walker C, Dunbar JA. Patient and carer identified factors which contribute to safety incidents in primary care: a qualitative study. BMJ Qual Saf. 2015; 24(9):583-93.

40. Litchfield I, et al. Routine failures in the process for blood testing and the communication of results to patients in primary care in the UK: a qualitative exploration of patient and provider perspectives. BMJ Qual Saf. 2015 Nov;24(11):681-90

41. Lawton R, O'Hara JK, Sheard L, Reynolds C, Cocks K, Armitage G, et al. Can staff and patient perspectives on hospital safety predict harm-free care? An analysis of staff and patient survey data and routinely collected outcomes. BMJ Qual Saf. 2015;24(6):369-376.

42. Elder NC, Meulen MV, Cassedy A. The Identification of Medical Errors by Family Physicians During Outpatient Visits. Annals of Family Medicine. 2004;2(2):125-9.

For peer review only - http://bmjopen.bmj.com/site/about/guidelines.xhtml

43. Heneghan C, Glasziou P, Thompson M, Rose P, Balla J, Lasserson D, et al. Diagnostic strategies used in primary care. BMJ. 2009;338.

44. The Health Foundation. Person-centred care made simple. 2014. http://www.health.org.uk/sites/health/files/PersonCentredCareMadeSimple.pdf Accessed 04/04/17

For peer review only

For peer review only - http://bmjopen.bmj.com/site/about/guidelines.xhtml

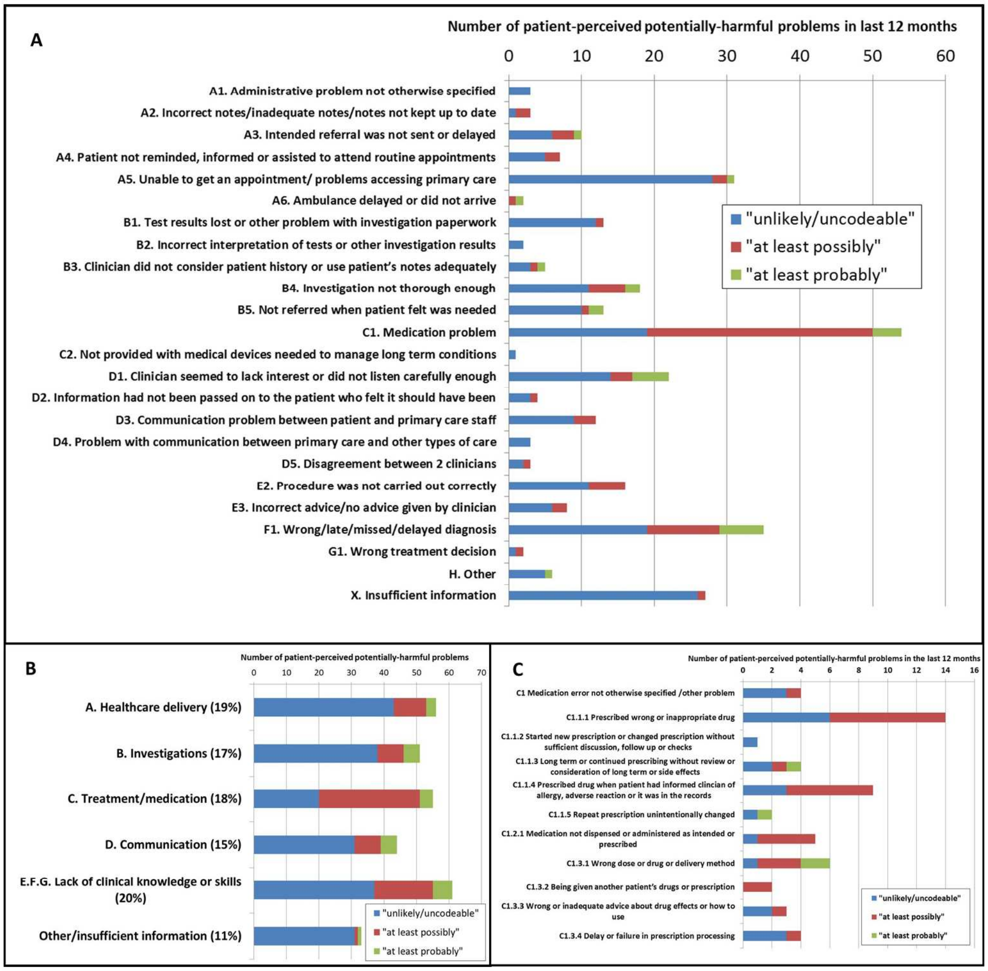

Footnote to figure 1: See Tables A&B, online Appendix 1 for details of coding; A coded to 2 levels, B coded to 1 level, C medication problems coded to 3 levels

Fig 1. Numbers of patient-perceived problems occurring in the last 12 months categorised according to the patient's description with clinician ranking as to the likelihood it is a potentially-harmful preventable problem (Table E, online Appendix 1).

174x170mm (300 x 300 DPI)

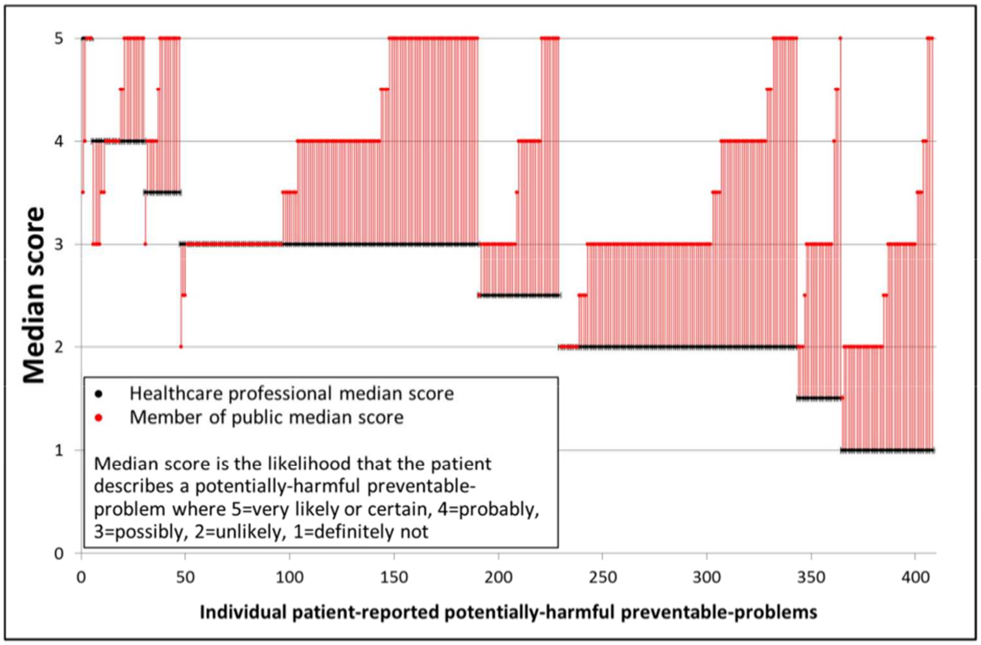

Figure 2. Median clinician and members of the public estimates of the likelihood that the patient describes a potentially-harmful preventable-problem occurring in the last 12 months

81x53mm (300 x 300 DPI)

Appendix 1. Supplementary methods and results

SJ Stocks et al. BMJ Open 2018: The frequency and nature of potentially-harmful preventable-problems in primary care from the patient's perspective with clinician review – a population level survey in Great Britain

Survey administered as part of the Ipsos MORI GB Face to Face Omnibus between 8th and 21st April 2016

We'd now like you to think about the last time you personally had an appointment for yourself, with a GP.

Q1. Did you have confidence and trust in the GP you saw or spoke to at your last appointment?
1. Yes, definitely 2. Yes, to some extent 3. No, not at all 4. Don't know / can't say

INTERVIEWER INSTRUCTION: READ OUT AND DISPLAY ON SCREEN.

The next few questions are about primary care.

Primary Care is the local healthcare that we receive at our GP or dental surgery, NHS walk-in centres, pharmacists (or high street chemist) and optometrists. This also could include all non-hospital care, for example, healthcare service provided by out of hours care, community (or district) nursing, ambulance, physiotherapy or other types of therapy or tests based at a GP surgery, learning disability services and any other non-hospital medical care.

We understand that this is a highly sensitive topic and would therefore like to remind you that any information you give is strictly confidential and will be used for research purposes only. You will not be identifiable as an individual from the responses you give.

At each question, if you do not wish to answer, you can refuse.

For the next question, we'd like you to think about the occasions when you have personally used primary care for yourself.

Q2a. Have you experienced a situation with a primary care service where your health has ACTUALLY been made worse by a problem or error that could have been prevented?
1. Yes 2. No 3. Don't Know

Q2b. And have you experienced a situation with a primary care service where you SUSPECTED your health has been made worse by a problem or error that could have been prevented?
1. Yes 2. No 3. Don't Know

Q2c. And have you experienced a situation with a primary care service where your health could have been made worse had someone not NOTICED a problem or error?
1. Yes 2. No 3. Don't Know

Q2d. And have you experienced a situation with a primary care service where there was a problem or error that could have been prevented but it did not make your health worse?
1. Yes 2. No 3. Don't Know

IF 2 OR MORE SCENARIOS AT Q2a to Q2e ARE CODED 1 THEN ASK Q2e

For peer review only

Q2e. You mentioned you have experienced the following situation(s) with a primary care service. Which of the following did you experience most recently?

1. 'My health was made worse'
2 'I suspect health was made worse'
3 'My health could have been made worse if the problem or error had not been noticed'
4 'There was no effect on my health'

ASK ALL WHO CODE 1 AT Q2
Q3. Thinking about the most recent occasion where you experienced a preventable problem or error caused by the primary care service, when did this occur?
1. In the last 12 months
2. 1 year up to 2 years ago
3. 2 years up to 3 years ago
4. 3 or more years ago

ASK ALL CODING 1 AT Q2 OR 1 AT Q11
Q4. Thinking about the most recent occasion, which primary care service were you using when the problem or error occurred?
1. GP surgery
2. Out of hours care
3. Walk in clinic
4. Dental surgery
5. Pharmacy
6. Community or district nursing
7. Ambulance
8. Opticians
9. Other (please specify)

INTERVIEWER INSTRUCTION: For the next five questions, please record enough information so that somebody else reading the description can understand what happened.

ASK ALL CODING 1 AT Q2 OR 1 AT Q11
Q5. Thinking about the most recent problem or error you experienced, can you briefly describe what it was and how it happened?

Q6 In your opinion, how, if at all, could the problem or error have been avoided?

Q7. Were you able to talk about the problem or error with anybody WORKING IN THE PRIMARY CARE SERVICE?
1. Yes 2. No

INTERVIEWER INSTRUCTION: if prompted, this can be anyone in the primary care service, including for example, the receptionist at a GP surgery or another nurse/doctor who wasn't working directly in their care.

ASK ALL CODING 1 AT Q7
Q8. You said you were able to discuss the problem or error with somebody working in primary care. Please describe their job or role and their response.

For peer review only

ASK ALL CODING 2 AT Q7

Q9. Which of the following reasons, if any, best describes why you were unable to talk about the problem or error with somebody working in the primary care service?

1. I had the opportunity but did not feel comfortable discussing the problem or error
2. I could not find anybody with whom I could discuss the problem or error
3. I was not concerned about the problem or error
4. I did not notice the problem or error
5. I was too distressed to discuss the problem or error
6. Other (please specify)

ASK IF (Q2 '2 OR DK OR REF')

Q10. In the last 12 months, have any of the following happened to you **while** using primary care, or not? 1. Yes 2. No

IF YES AT Q11, REDIRECT TO Q4

(RANDOMISE 1-16(KEEP 2&3 TOGETHER, KEEP 6&7 TOGETHER, KEEP 9&10 TOGETHER), ALLOW DK AND REF)

1. Received a wrong or late diagnosis
2. Was not referred for further investigation when requested by you as a patient
3. Was not referred for further investigation in error by healthcare practitioner (for example, they forgot to refer you onwards)
4. Test results lost or mixed up
5. Received the wrong medicine or wrong dose
6. Should not have been prescribed medicine because of another health problem
7. Should not have been prescribed medicine because of another medication already being taken
8. Poor communication leading to misunderstanding of diagnosis or treatment
9. Not referred to a specialist when needed when requested by you as a patient
10. Not referred to a specialist when needed in error by healthcare practitioner (for example, they forgot to refer you onwards)
11. Received unclear instructions about treatment
12. Not offered access to prevention or screening programmes e.g. CVD/stroke prevention clinics
13. A medical professional failed to recognise or act on vulnerable people's needs e.g. child abuse, suicide risk or mental health problems
14. Mistake with a procedure e.g. dental treatment, injection, ear syringing, physiotherapy
15. Not notified about recommended vaccinations e.g. flu, HPV
16. A medical professional practicing poor hygiene

ASK ALL CODING 1 AT Q2 OR 1 AT Q11

Q11. Do you, personally, work as a Healthcare Professional in any capacity? For example, a doctor/nurse/therapist/pharmacist/other NHS staff, etc.

1. Yes 2. No

For peer review only

Table A. Coding of patient-reported potentially-unsafe scenarios in primary care

**1. Errors in the process of the healthcare delivery system**	
Makeham 2002, Dovey 2002	Common threads reported in this study
1.1. Errors in the process of conducting an administrative task	A1. Administrative problem not otherwise specified
1.1.1. Information filed in wrong place or wrong time	
1.1.2. Unavailability of information that should have been in patients charts<br>1.1.2.1. Entire chart or part of chart could not be accessed when needed<br>1.1.2.2. Care provided was not documented<br>1.1.2.3. Item(s) of information missing from chart	A2. Incorrect notes/inadequate notes/notes not kept up to date
1.1.3. Errors in patient's movement through the healthcare delivery system	A3. Intended referral was not sent or delayed<br>A4. Patient not reminded, informed or assisted to attend regular check-ups or other necessary routine treatments
1.1.4. Errors in the taking and distributing of messages	
1.1.5. Errors in managing appointments for healthcare	A5. Unable to get an appointment/other problems with making appointment<br>A6. Ambulance delayed or did not arrive
**1.2. Errors in the process of investigating a patient's condition**	
1.2.1. Laboratory errors<br>1.2.1.1. Wrong test ordered or test not ordered when appropriate<br>1.2.1.2. Errors in the process of obtaining or processing a laboratory specimen<br>1.2.1.3. Error in the process of physician receiving accurate laboratory results in a timely fashion<br>1.2.1.4. Inappropriate response to an abnormal laboratory result	B1. Test results lost or other problem with investigation or paperwork<br>B2. Incorrect interpretation of tests or other investigation results<br>B3. Clinician did not consider patient history sufficiently/did not use patient's notes adequately<br>B4. Investigation not thorough enough<br>B5. Not referred when patient felt was needed
1.2.3. Errors in the processes of other investigations<br>1.2.3.1. Wrong test ordered or test not ordered when appropriate<br>1.2.3.2. Errors in the process of obtaining or processing of other diagnostic investigation<br>1.2.3.3. Error in the process of physician receiving accurate test results of other investigation in a timely fashion<br>1.2.3.4. Inappropriate response to an abnormal result of other investigation	
**1.3. Errors in the process of treating a patient's condition**	
1.3.1. Errors in the process of treating with medications<br>1.3.1.1. Wrong medication or wrong dose of medication ordered or medication not ordered by physician when appropriate<br>1.3.1.2. Error in the process of delivering a medication order or inappropriate medication order by a provider working under physician supervision<br>1.3.1.3. Error in the process of dispensing medication as ordered	C1. Medication problem<br><br>C2. Not provided with medical devices needed to manage long term conditions

For peer review only

1.3.2. Errors in other treatments	C3. Problem with dental treatment or diagnosis
**1.4. Errors in the process of communication**	
1.4.1. Errors in communication between primary healthcare provider and patients	D1. Clinician seemed to lack interest in the patient's health problem or did not listen carefully enough<br>D2. Information about the patient's health had not been passed on to the patient who felt it should have been<br>D3. Communication problem between patient and primary care staff
1.4.2. Errors in communication between healthcare providers	D4. Problem with communication between primary care and other types of care including secondary care<br>D5. Disagreement between 2 clinicians
**2. Errors arising from lack of clinical knowledge or skills**	
2.1. Errors in the execution of a clinical task<br>2.1.1. Non-clinical staff made the wrong clinical decision<br>2.1.2. Failed to follow standard practice<br>2.1.3. Lacked needed experience or expertise in a clinical task	E1. Administrative staff seemed to make clinical decisions<br>E2. Procedure was not carried out correctly<br>E3. Incorrect advice/no advice given by clinician
**2.2. Errors in diagnosis**<br>2.2.1. Wrong or delayed diagnosis	F1. Wrong/late/missed/delayed diagnosis
**2.3. Wrong treatment decision**	G1. Wrong treatment decision
	H. Other
	X. Not a problem/ insufficient information/refused/don't know

Table B. Level 4 coding of patient-reported potentially-unsafe medication scenarios

Common threads reported in this study grouped as described by Makeham 2002, Dovey 2002
C1 Medication error not otherwise specified /other problem
• **1.3.1.1. Ordering medications (prescribing)**
C1.1.1 Prescribed wrong or inappropriate drug
C1.1.2 Started new prescription or changed prescription without sufficient discussion, follow up or checks
C1.1.3 Long term or continued prescribing without review or consideration of long term or side effects
C1.1.4 Prescribed drug when should have known contra-indicated *e.g.* patient had informed clinician of allergy, adverse reaction or it was in the records
C1.1.5 Repeat prescription unintentionally changed
C1.1.6 Out of date repeat prescription mistakenly re-issued
• **1.3.1.2./1.3.1.3. Implementing or receiving medications (dispensing or issuing)**
C1.2.1 Medication not dispensed or administered as intended or prescribed
• **1.3.1.1/1.3.1.2./1.3.1.3. Ordering, implementing or receiving medications**
C1.3.1 Wrong dose or drug or delivery method
C1.3.2 Being given another patient's drugs or prescription
C1.3.3 Wrong or inadequate advice about drug effects or how to use
C1.3.4 Delay or failure in prescription processing

For peer review only

Table C. Scoring for likelihood that the patient-reported scenario is potentially-unsafe

Score	How likely do you think it is the patient was correct in thinking that their health might be worsened, or actually was made worse, because of a mistake or a problem in primary care that could have been prevented? Choose from the options below.
5	Very likely or certain (75-100% confident is a potentially unsafe scenario)
4	Probably (50-74% confident is a potentially unsafe scenario)
3	Possibly (25-49% confident is a potentially unsafe scenario)
2	Unlikely (bottom 25% confident is a potentially unsafe scenario)
1	Definitely not a potentially unsafe event (0% chance is a potentially unsafe scenario)
-	Insufficient information
-	Don't know
-	Other - add text at end of row

For peer review only

Supplementary results

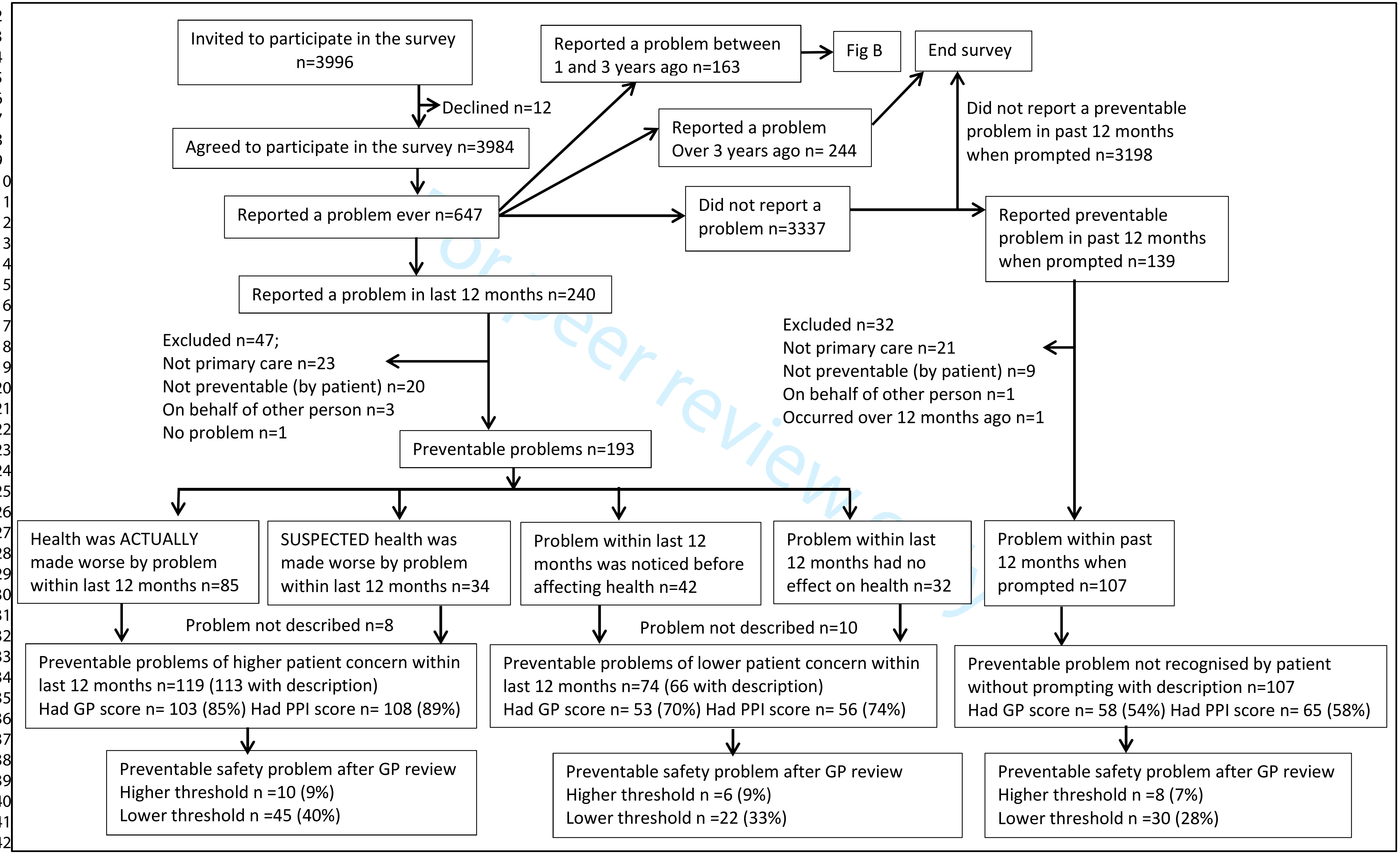

Fig A. Flow chart of participants reporting a potential-harmful preventable-problem within the last 12 months

For peer review only - http://bmjopen.bmj.com/site/about/guidelines.xhtml

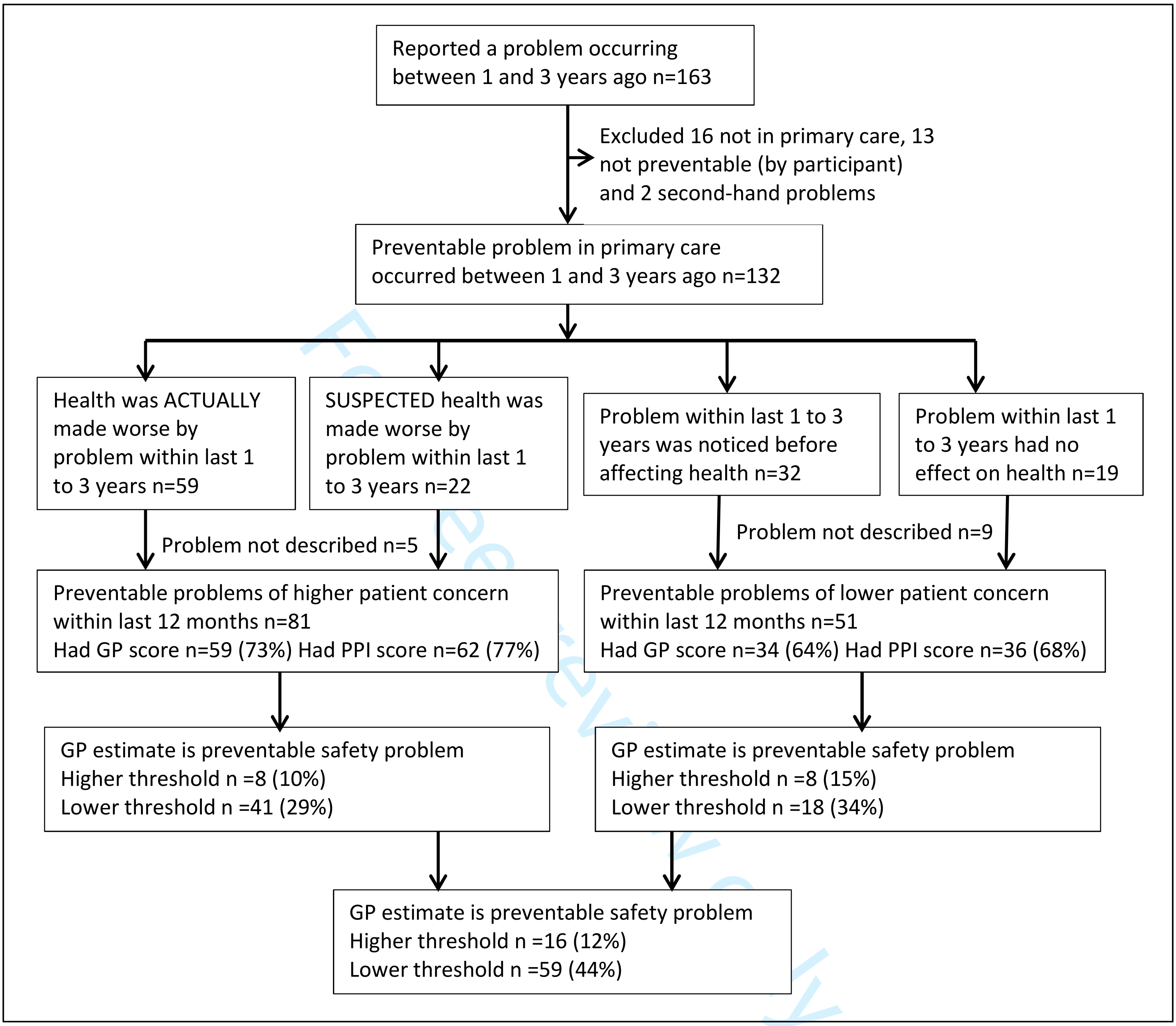

Fig B. Flow chart of participants reporting a potential-harmful preventable-problem within the last 1 to 3 years

For peer review only - http://bmjopen.bmj.com/site/about/guidelines.xhtml

Table D. Demographics of responders to Ipsos MORI GB Face to Face Omnibus April 2016

	Number of participants (%) n=3984	Population level estimates for comparison	Population comparator source; $P(\chi^2)$= probability survey population differs from population comparator
**Confidence and trust in GP at last appointment?**			
Yes definitely	3031 (76%)	523498 (63%)	GP patient survey in England mid-2015(25) $P(\chi^2)<0.0001$
Yes, to some extent	611 (15%)	235760 (29%)	
No, not at all	311 (8%)	37743 (5%)	
Don't know /can't say	31 (1%)	28866 (3%)	
**Gender (1 missing)**			
Male	1950 (49%)	32074400 (49%)	ONS mid-2015 estimates[1] $P(\chi^2)=0.7$
Female	2033 (51%)	33035600 (51%)	
**Age**			
15 to 24	533 (13%)	8118600 (15%)	ONS mid-2015 estimates[1] $P(\chi^2)<0.0001$
25 to 34	573 (14%)	8822700 (16%)	
35 to 44	528 (13%)	8378300 (16%)	
45 to 54	629 (16%)	9196000 (17%)	
55 to 64	654 (16%)	7452400 (13%)	
65 to 74	609 (15%)	6339800 (11%)	
75 or older	458 (12%)	5271400 (10%)	
**Ethnicity (18 missing)**			
White	3491 (88%)	48209395 (86%)	England & Wales census (2011)[2] $P(\chi^2)<0.0001$
Other ethnicity	475 (12%)	7866517 (14%)	
**Social Grade[3]**			
A/B	1054 (26%)	8081619 (23%)	England & Wales census (2011)[2] $P(\chi^2)<0.0001$
C1	1122 (28%)	10796044 (30%)	
C2	771 (19%)	7865976 (22%)	
D/E	1037 (26%)	8903873 (25%)	

[1]https://www.ons.gov.uk/peoplepopulationandcommunity/populationandmigration/populationestimates/bulletins/annualmidyearpopulationestimates/latest
[2]https://www.ons.gov.uk/peoplepopulationandcommunity/populationandmigration/populationestimates/bulletins/keystatisticsandquickstatisticsforlocalauthoritiesintheunitedkingdom/2013-10-11
[3]A/B High or intermediate managerial, professional or administrative, C1 Supervisory, clerical and junior managerial, professional or administrative, C2 skilled manual workers, D/E semi and unskilled manual workers, casual or lowest grade workers, state pensioners, unemployed with state benefits only

For peer review only - http://bmjopen.bmj.com/site/about/guidelines.xhtml

Table E. Categorisation of patient-described scenarios according to clinician ranking as to the likelihood they represent a potentially-harmful preventable-problem

Group	Scores on a 5 point scale of "very likely or certain", "probably", "possibly", "unlikely", "definitely not" (see table C, online Appendix 2)	Unprompted problems (answered "yes" to Q2, Box1)				All problems within past 12 months (answered "yes" to Q2or Q10, Box1) n=300	
		Within past 12 months n=193		Within past 3 years n=325			
		Clinicians	Members of the Public	Clinicians	Members of the Public	Clinicians	Members of the Public
1. Higher threshold	Median score higher than "probably" or at least one score of "very likely or certain"	16 (8%)	91 (47%)	28 (9%)	165 (51%)	24 (8%)	116 (39%)
2. Lower threshold	Median score higher than "possibly" or at least one score of "probably" or higher	67 (35%)	145 (75%)	124 (38%)	237 (73%)	97 (32%)	198 (66%)
3. Any possibility	At least one score of "unlikely" or higher	141 (73%)	157 (81%)	232 (71%)	254 (78%)	194 (65%)	221 (74%)
4. No problem	All scores "definitely not" (or not-coded)	8 (4%)	0	9 (3%)	0	13 (4%)	0
5. Not-coded	Insufficient information for coding by all raters	44 (23%)	36 (19%)	84 (26%)	71 (22%)	93 (31%)	79 (26%)

For peer review only

Table F. Survey responses and respondent characteristics as predictors of clinician and members of the public estimates of the likelihood that the scenario describes a potentially-harmful preventable problem

Respondent characteristics (total) n=406 (ranked by at least one clinician)	Clinician – lower threshold[1] (n=224, 55%)		Members of the public – higher threshold[2] (n=267, 66%)	
	Frequency (%)	Adjusted odds ratio	Frequency (%)	Adjusted odds ratio
**Source of respondent (0 missing)**				
Ipsos MORI f2f Omnibus (299)	153 (51%)	1 (ref)	182 (61%)	1 (ref)
Pilot survey (107)	71 (66%)	1.5 (0.9 to 2.7)	85 (79%)	5.2 (2.5 to 10.8)
**Gender (3 missing)**				
Male (150)	79 (53%)	1 (ref)	93 (62%)	1 (ref)
Female (253)	142 (56%)	1.2 (0.8 to 1.9)	172 (68%)	1.5 (0.9 to 2.4)
**Age (3 missing)**				
15 to 24 years (46)	21 (46%)	1 (ref)	28 (61%)	1 (ref)
25 to 34 years (60)	34 (57%)	1.5 (0.7 to 3.5)	43 (72%)	1.4 (0.6 to 3.7)
35 to 44 years (38)	24 (63%)	1.8 (0.7 to 4.5)	30 (79%)	1.9 (0.6 to 5.6)
45 to 54 years (74)	44 (59%)	1.5 (0.7 to 3.4)	50 (68%)	1.1 (0.5 to 2.7)
55 to 64 years (82)	45 (55%)	1.4 (0.6 to 3.2)	50 (61%)	1.0 (0.4 to 2.3)
65 to 74 years (75)	39 (52%)	1.2 (0.5 to 2.8)	49 (65%)	1.1 (0.4 to 2.6)
75 years or older (28)	14 (50%)	1.1 (0.4 to 3.2)	15 (54%)	0.6 (0.2 to 1.8)
**Patient estimate of impact of the problem on their health (0 missing)**				
Actually or suspected made health worse (192)	109 (57%)	1 (ref)	139 (73%)	1 (ref)
Noticed before made health worse or had no effect on health (106)	58 (55%)	0.8 (0.5 to 1.4)	69 (65%)	0.6 (0.3 to 1.1)
Prompted by Q10 (108)	57 (53%)	0.7 (0.4 to 1.2)	59 (55%)	0.3 (0.1 to 0.5)
**Patient is qualified as a healthcare professional or volunteers in healthcare research[2] (0 missing)**				
No (339)	177 (52%)	1 (ref)	221 (65%)	1 (ref)
Yes (67)	47 (70%)	2.0 (1.1 to 3.8)	46 (69%)	0.8 (0.4 to 1.7)
**Discussed the problem with somebody working in the primary care service (0 missing)**				
No/don't know/missing (197)	99 (50%)	1 (ref)	119 (60%)	1 (ref)
Yes (209)	125 (60%)	1.3 (0.9 to 2.0)	148 (71%)	1.5 (0.9 to 2.4)
**Service used (1 missing)**				
GP surgery (286)	159 (56%)	1 (ref)	186 (65%)	1 (ref)
Dental surgery (36)	17 (46%)	0.8 (0.3 to 1.7)	12 (33%)	1.1 (0.5 to 2.7)
Walk in clinic (16)	7 (44%)	1.0 (0.4 to 3.0)	10 (63%)	1.7 (0.5 to 5.7)
Ambulance/A&E/ OOH (20)	13 (65%)	2.0 (0.7 to 5.5)	15 (75%)	3.8 (1.0 to 14.1)
Pharmacy (18)	15 (83%)	2.0 (0.5 to 7.8)	3 (17%)	1.0 (0.2 to 4.3)
Other (29)	12 (41%)	0.7 (0.3 to 1.7)	14 (48%)	1.4 (0.6 to 3.4)
**Problem related to (0 missing)**				
A. Healthcare delivery system (65)	25 (38%)	1 (ref)	24 (37%)	1 (ref)
B. Investigation (63)	29 (46%)	1.2 (0.6 to 2.5)	42 (67%)	3.4 (1.5 to 7.6)
C. Treatment process (100)	73 (73%)	3.7 (1.8 to 7.7)	85 (85%)	11.0 (4.6 to 26.5)
D. Communication (66)	36 (55%)	1.8 (0.9 to 3.7)	37 (56%)	2.0 (0.9 to 4.2)
E. Clinical knowledge or skills (43)	23 (53%)	1.8 (0.8 to 4.2)	30 (70%)	3.3 (1.3 to 8.4)
F. Diagnosis (56)	34 (61%)	2.5 (1.1 to 5.4)	79 (21%)	6.2 (2.6 to 15.1)
G. Wrong treatment decision (4)	2 (50%)	1.4 (0.2 to 11.5)	3 (75%)	3.9 (0.4 to 41.7)

For peer review only - http://bmjopen.bmj.com/site/about/guidelines.xhtml

H. Other (9)	2 (22%)	0.5 (0.1 to 2.8)	2 (22%)	0.4 (0.1 to 2.2)
**Relevant condition (0 missing)**	**Frequency (%)**	**Unadjusted odds ratio[3]**	**Frequency (%)**	**Unadjusted odds ratio[3]**
All other conditions (47)	24 (51%)	1 (ref)	29 (19%)	1 (ref)
Cardiovascular (8)	7 (88%)	6.7 (0.8 to 58.9)	8 (100%)	-[4]
Diabetes (32)	20 (63%)	1.6 (0.6 to 4.0)	24 (75%)	1.8 (0.7 to 5.0)
Cancer (7)	7 (100%)	-[4]	7 (100%)	-[4]
Mental health (18)	6 (33%)	0.5 (0.2 to 1.5)	15 (83%)	3.1 (0.8 to 12.2)
Dental (33)	16 (48%)	0.9 (0.4 to 2.2)	24 (73%)	1.7 (0.6 to 4.3)
Accidental injury (17)	10 (59%)	1.4 (0.4 to 4.2)	12 (71%)	1.5 (0.4 to 4.9)
Infectious (12)	8 (67%)	1.9 (0.5 to 7.2)	10 (83%)	3.1 (0.6 to 15.8)
Pain/discomfort (15)	8 (53%)	1.1 (0.3 to 3.5)	5 (30%)	0.3 (0.1 to 1.1)
Skin (12)	5 (42%)	0.7 (0.2 to 2.5)	4 (33%)	0.3 (0.1 to 1.2)
Respiratory (13)	9 (69%)	2.2 (0.6 to 8.0)	12 (92%)	7.4 (0.9 to 62.2)
Pregnancy (8)	6 (75%)	2.9 (0.5 to 15.7)	8 (100%)	-[4]
Musculoskeletal (34)	11 (32%)	0.5 (0.2 to 1.1)	16 (47%)	0.6 (0.2 to 1.3)
Ear, nose and throat (9)	6 (67%)	1.9 (0.4 to 8.6)	4 (44%)	0.5 (0.1 to 2.1)
Not relevant/not known (141)	81 (57%)	1.3 (0.7 to 2.5)	89 (63%)	1.1 (0.5 to 2.1)

[1]median score higher than “probably” or at least one score of “very likely or certain”, see Table B
[2]median score higher than “possibly” or at least one score of “probably” or higher, see Table B
[3]unadjusted OR shown due to collinearity between dental problems and dental service
[4]predicts success perfectly (100% of scenarios in this category)

For peer review only

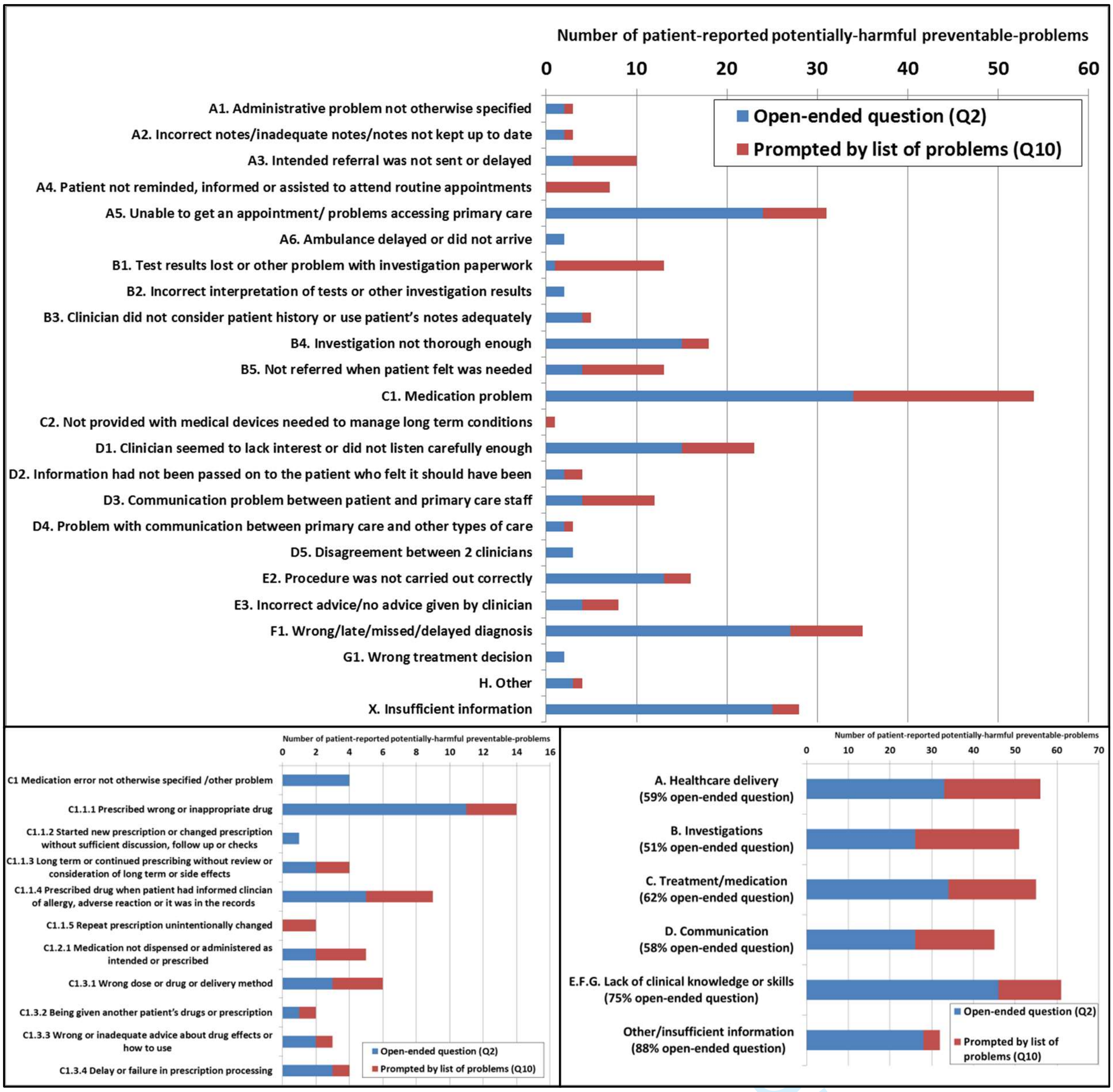

Fig C. Numbers of patient-perceived problems occurring in the last 12 months categorised according to the patient's description (see Table 2) and route through survey *i.e.* originated from open-ended question (Q2) or prompted by list of potential safety problems (Q10). See online Appendix 2 for details of coding; A coded to 2 levels, B medication problems coded to 3 levels, C coded to 1 level

For peer review only - http://bmjopen.bmj.com/site/about/guidelines.xhtml

Appendix 2. SJ Stocks et al. BMJ Open 2018: The frequency and nature of potentially-harmful preventable-problems in primary care from the patient's perspective with clinician review – a population level survey in Great Britain Patient reported scenarios occurring during the past 12 months that clinicians scored as higher likelihood to be a potentially-unsafe preventable-problem in primary care (median score is higher than "possibly" and at least 2 clinicians gave a score or one clinician scored "very likely or certain"). PPI = member of the public, GP = primary care clinician

Scenario1. Ambulance

Briefly describe the mistake or problem and how it happened. *"Heart attack, an ambulance was called and waited an hour and three quarters to arrive"*

Could the mistake or problem have been avoided? If so how? "*The ambulance service needs to be sorted out"*

Were you able to talk about the mistake or problem with anybody working in the primary care service? *"No I was too distressed to discuss the problem or error"*

Patient-reported prospect of harm: health could have been made worse had someone not noticed a problem or error

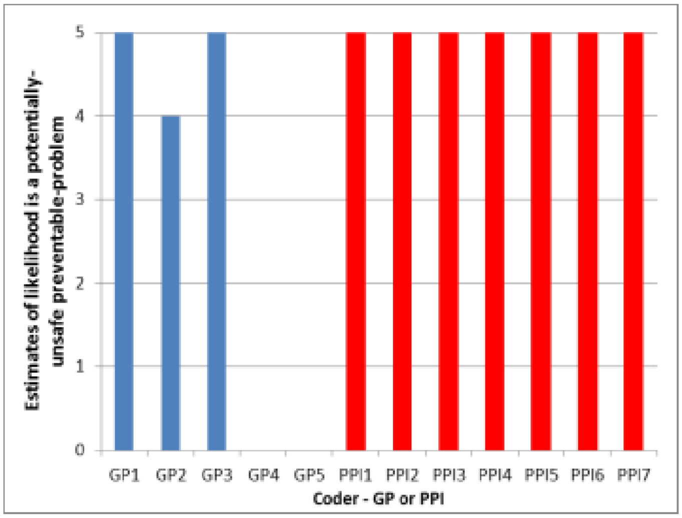

5=very likely or certain, 4=probably, 3=possibly, 2=unlikely, 1=definitely not, 0 = insufficient information or don't know

Patient-perspective problem-type code: A6. Ambulance delayed or did not arrive

Scenario2. GP surgery

Briefly describe the mistake or problem and how it happened. *"I had an ongoing stomach complaint. The GP kept prescribing a steroid treatment but the pharmacist refused to give it to me. He said it was dangerous and I had to get different medication. The GP prescribed an alternative but the pharmacist pointed out that the steroid was supposed to be a short term treatment and that the GP had been prescribing it for over a year."*

Could the mistake or problem have been avoided? If so how? "*The GP obviously didn't read the notes. The GP was probably pushed for time and just wanted to get me out (maybe?)"*

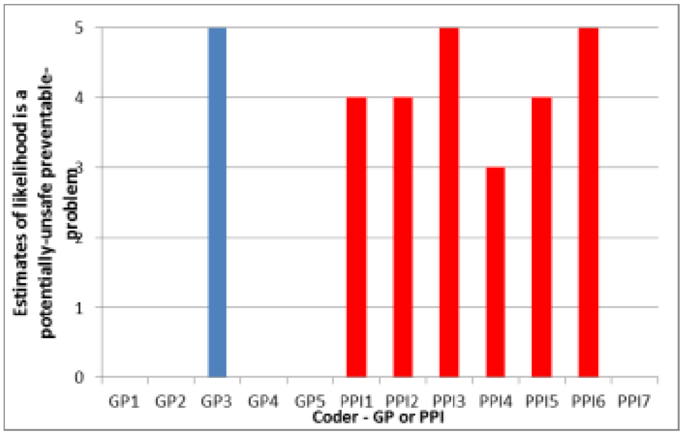

5=very likely or certain, 4=probably, 3=possibly, 2=unlikely, 1=definitely not, 0 = insufficient information or don't know

Were you able to talk about the mistake or problem with anybody working in the primary care service? *"No I was not concerned about the problem or error"*

Patient-reported prospect of harm: prompted via Q10 (Box 1 main paper)

Patient-perspective problem-type code: C1.1.3 Long term or continued prescribing without review or consideration of long term or side effects; B3 Clinician did not consider patient history sufficiently/did not use patient's notes adequately

Scenario3. GP surgery

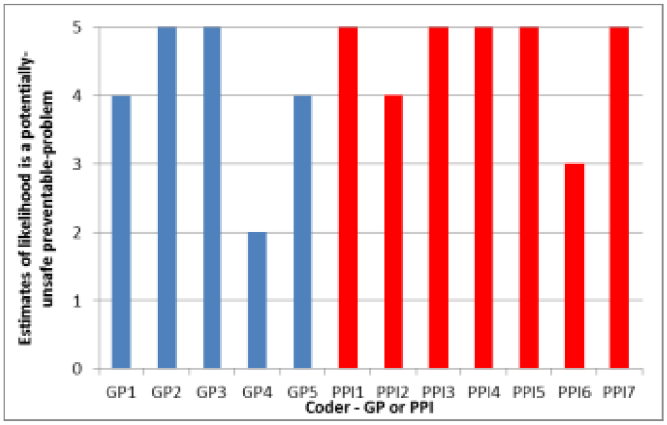

5=very likely or certain, 4=probably, 3=possibly, 2=unlikely, 1=definitely not, 0 = insufficient information or don't know

Briefly describe the mistake or problem and how it happened. *"Participant was prescribed penicillin and it was stated in notes that patient was allergic to penicillin"*

Could the mistake or problem have been avoided? If so how? *"It was avoided as participant didn't take prescription and was prescribed something else"*

Were you able to talk about the mistake or problem with anybody working in the primary care service? *"Yes with GP"*

Patient-reported prospect of harm: health could have been made worse had someone not noticed a problem or error

Patient-perspective problem-type code: C1.1.4 Prescribed drug when should have known contra-indicated *e.g.* patient had informed clinician of allergy, adverse reaction or it was in the records

Scenario4. Optician

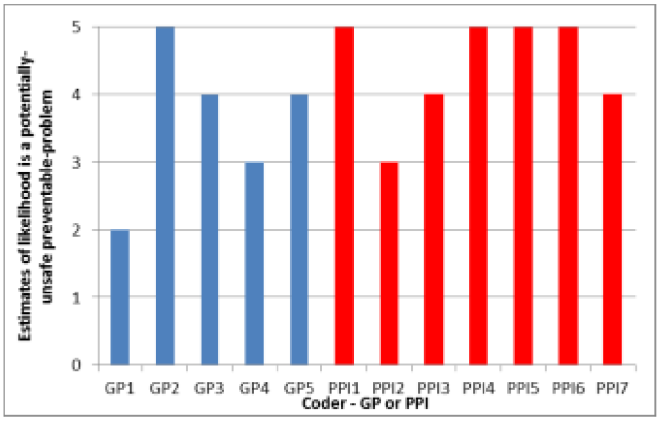

5=very likely or certain, 4=probably, 3=possibly, 2=unlikely, 1=definitely not, 0 = insufficient information or don't know

Briefly describe the mistake or problem and how it happened. *"Started suffered blurred vision in left eye, eye was bloodshot. Went to get eye check and was sold eye drops to treat infection, told would take five days. After five days of treatment problem was made worse until vision was affected, GP referred to eye clinic diagnosed with iritis. Further treatment at eye clinic cleared up the issue."*

Could the mistake or problem have been avoided? If so how? *"If optometrists had spotted that iris was stuck, had a bit more professional care rather than trying to flog over-the-counter eye drops to clear up infection that wasn't there"*

Were you able to talk about the mistake or problem with anybody working in the primary care service? *"Yes, spoke to GP, immediate referral to eye clinic for treatment"*

Patient-reported prospect of harm: health was actually made worse by a problem or error that could have been prevented

Patient-perspective problem-type code: F1 Wrong/late/missed/delayed diagnosis

Scenario5. GP surgery

Briefly describe the mistake or problem and how it happened. *"Contra-indication with a medicine that was not noticed at time of prescription but was noticed by the participant before they started taking the medicine"*

Could the mistake or problem have been avoided? If so how? "*The contra-indication should have been flagged up on the computer at the time of prescription but it wasn't"*

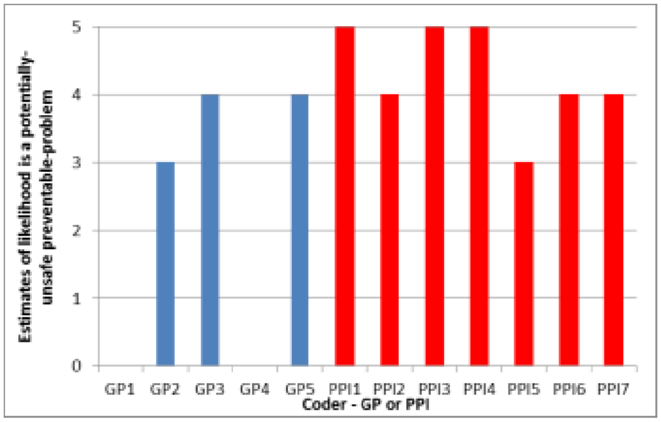

5=very likely or certain, 4=probably, 3=possibly, 2=unlikely, 1=definitely not, 0 = insufficient information or don't know

Were you able to talk about the mistake or problem with anybody working in the primary care service? "Yes, *secretary and a GP"*

Patient-reported prospect of harm: health could have been made worse had someone not noticed a problem or error

Patient-perspective problem-type code: C1.1.4 Prescribed drug when should have known contra-indicated *e.g.* patient had informed clinician of allergy, adverse reaction or it was in the records

Scenario6. GP surgery

Briefly describe the mistake or problem and how it happened. *"Went with a lump to GP. He referred me under the 2 week NICE guidelines. The communication went wrong and I chased it up myself or would have remained sat here. I ended up being diagnosed with cancer but I intervened in time."*

Could the mistake or problem have been avoided? If so how? *"Policies & procedures in place now. If you're sent an appointment that place needs to send a confirmation. That's what happened to stop it happening again."*

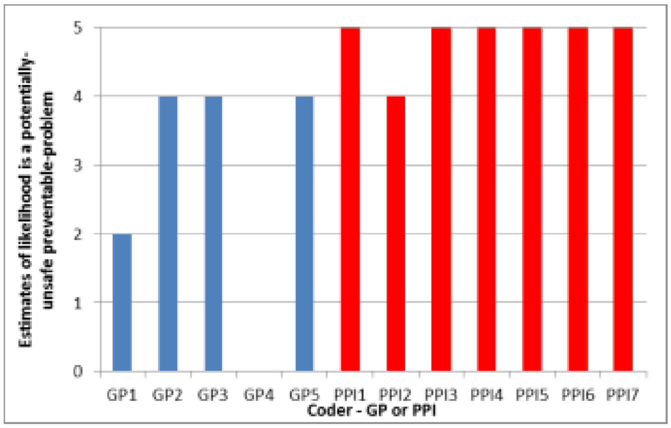

5=very likely or certain, 4=probably, 3=possibly, 2=unlikely, 1=definitely not, 0 = insufficient information or don't know

Were you able to talk about the mistake or problem with anybody working in the primary care service? *"GP investigated it as a significant event. Said if not satisfied come in and chat to us. I had apology from GP."*

Patient-reported prospect of harm: health could have been made worse had someone not noticed a problem or error

Patient-perspective problem-type code: A3. Intended referral was not sent or delayed

Scenario7. Pharmacy

Briefly describe the mistake or problem and how it happened. *"They gave me the wrong tablets and they were heart pills - beta blockers- but I thought they were sleeping pills. I looked at the patient information and thought why am I not sleeping and realised they were for people who had had a heart attack. I was taking them for 6 weeks then I phoned the doctor and he came straight away. The pharmacist no longer works there."*

Could the mistake or problem have been avoided? If so how? *"She just put up the wrong tablets. She should have dispensed the right pills as on my prescription"*

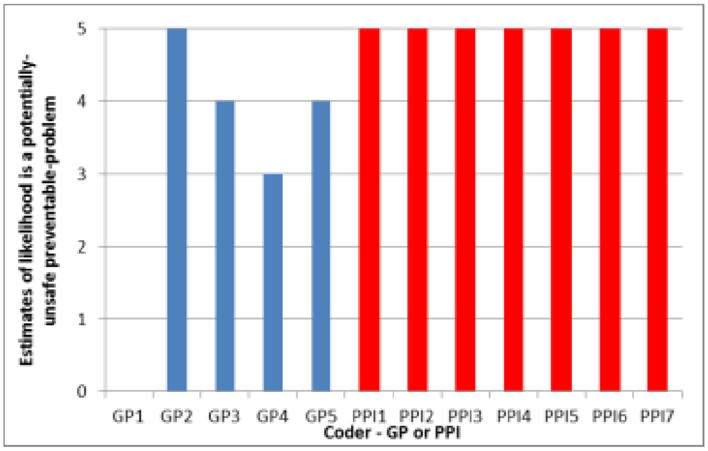

5=very likely or certain, 4=probably, 3=possibly, 2=unlikely, 1=definitely not, 0 = insufficient information or don't know

Were you able to talk about the mistake or problem with anybody working in the primary care service? *"Yes, doctor - he gave me the right ones"*

Patient-reported prospect of harm: health could have been made worse had someone not noticed a problem or error

Patient-perspective problem-type code: C1.2.1 Medication not dispensed or administered as intended or prescribed

Scenario8. Out of hours care

Briefly describe the mistake or problem and how it happened. *"Banged foot at work, hurt a lot, for few days got worse"*

Could the mistake or problem have been avoided? If so how? *"if they had listened to me properly, they didn't therefore toe got amputated for no reason"*

Were you able to talk about the mistake or problem with anybody working in the primary care service? *"Yes, triage nurse"*

Patient-reported prospect of harm: health could have been made worse had someone not noticed a problem or error

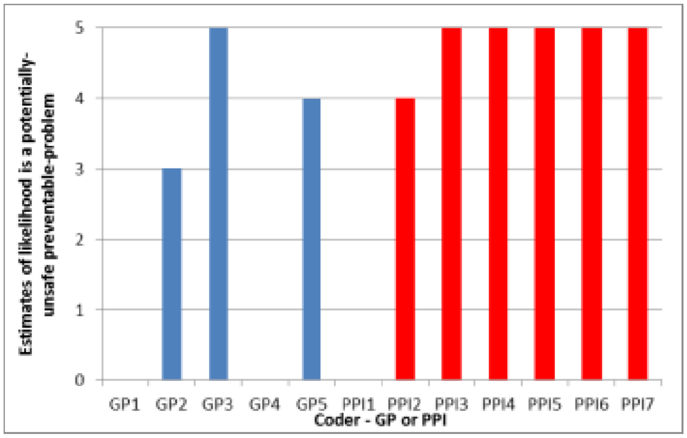

5=very likely or certain, 4=probably, 3=possibly, 2=unlikely, 1=definitely not, 0 = insufficient information or don't know

Patient-perspective problem-type code: B4. Investigation not thorough enough; D1. Clinician seemed to lack interest in the patient's health problem or did not listen carefully enough

Scenario9. GP surgery

Briefly describe the mistake or problem and how it happened. *I was started on warfarin and was fainting and bleeding rectally. I was in town the first time I passed out and did not go to hospital. The second time I went to hospital and the problem was rectified by reducing the dose."*

Could the mistake or problem have been avoided? If so how? *"by giving a smaller dose in the first place. I was told that the amount was too much. Afterwards they put me on something else instead of warfarin."*

Were you able to talk about the mistake or problem with anybody working in the primary care service? *"Yes, doctor in hospital"*

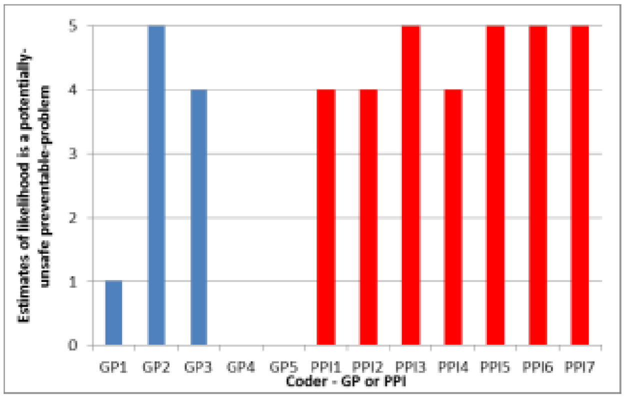

5=very likely or certain, 4=probably, 3=possibly, 2=unlikely, 1=definitely not, 0 = insufficient information or don't know

Patient-reported prospect of harm: health was actually made worse by a problem or error that could have been prevented

Patient-perspective problem-type code: C1.3.1 Wrong dose or drug or delivery method

Scenario10. GP surgery

Briefly describe the mistake or problem and how it happened. *"Couldn't get appointment at GP. Health worsened, ended up in hospital with fluid on lungs and pneumonia. Was rushed in. Heart had to be stopped and restarted."*

Could the mistake or problem have been avoided? If so how? *"Had rung for appointments and asked for doctor to telephone me 3 times. They never rang. They should have signed my prescriptions so I could have medicine and should have seen me in person"*

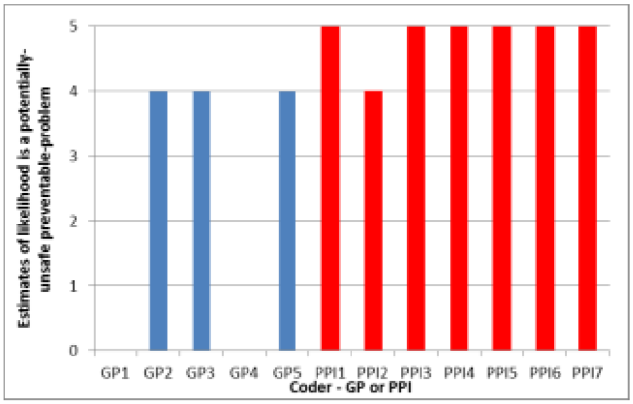

5=very likely or certain, 4=probably, 3=possibly, 2=unlikely, 1=definitely not, 0 = insufficient information or don't know

Were you able to talk about the mistake or problem with anybody working in the primary care service? *"The heart nurse from the community service complained on my behalf to the GP surgery. The chemist shop complained too about prescriptions not being signed and medicine being missed. Appointment was made at surgery to discuss with new doctor, and appointments are guaranteed as now a "supported patient"."*

Patient-reported prospect of harm: health was actually made worse by a problem or error that could have been prevented

Patient-perspective problem-type code: A5. Unable to get an appointment/other problems with making appointment; C1.3.4 Delay or failure in prescription processing

Scenario11. Dental surgery

Briefly describe the mistake or problem and how it happened. *“Dentist numbed me up to pull a wrong tooth”*

Could the mistake or problem have been avoided? If so how? *“By taking care by paying attention to his own notes”*

Were you able to talk about the mistake or problem with anybody working in the primary care service? *“Yes, the dentist himself - he was apologetic.”*

Patient-reported prospect of harm: a problem or error that could have been prevented but it did not make your health worse

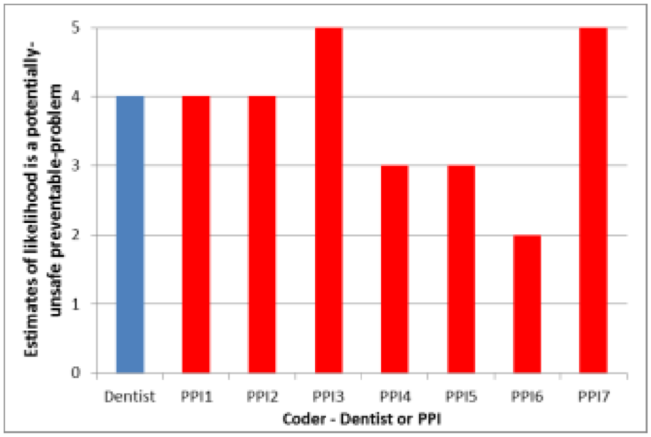

5=very likely or certain, 4=probably, 3=possibly, 2=unlikely, 1=definitely not, 0 = insufficient information or don’t know

Patient-perspective problem-type code: E2.Procedure was not carried out correctly

Scenario12. GP surgery

Briefly describe the mistake or problem and how it happened. *“Discharged from hospital following knee replacement surgery, became very ill, lost 1 stone in 7 days, requested home visit from GP as seriously concerned, doctor called by phone and was very brusque, no home visit but medication changed and 6 months later started to feel better”*

Could the mistake or problem have been avoided? If so how? *“if the doctor had come to see me in person who could have made a quicker diagnosis and could have offered some much needed support during a very traumatic time”*

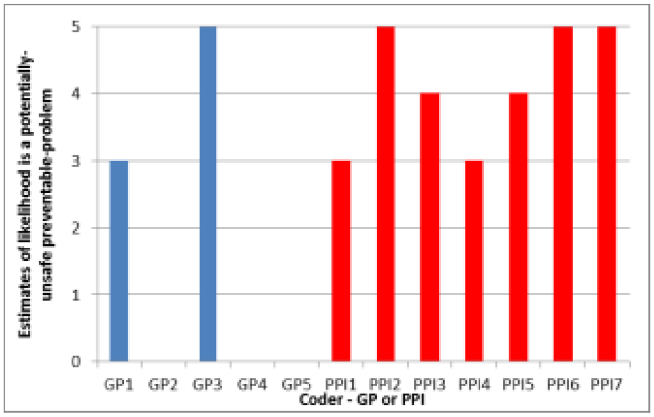

5=very likely or certain, 4=probably, 3=possibly, 2=unlikely, 1=definitely not, 0 = insufficient information or don’t know

Were you able to talk about the mistake or problem with anybody working in the primary care service? *“No, I could not find anybody with whom I could discuss the problem or error”*

Patient-reported prospect of harm: suspected your health has been made worse by a problem or error that could have been prevented

Patient-perspective problem-type code: D1. Clinician seemed to lack interest in the patient’s health problem or did not listen carefully enough

Scenario13. Pharmacy

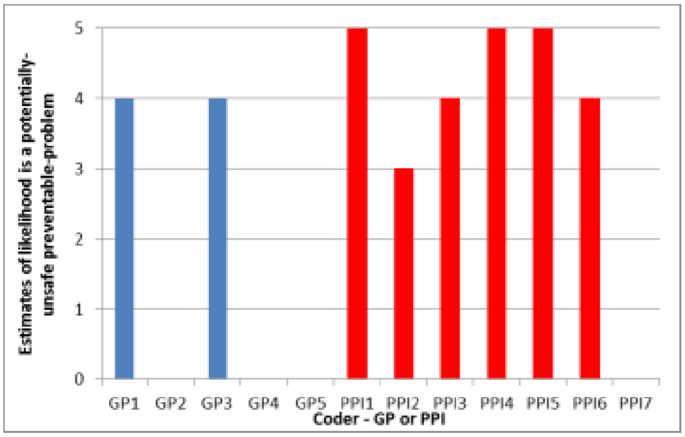

5=very likely or certain, 4=probably, 3=possibly, 2=unlikely, 1=definitely not, 0 = insufficient information or don't know

Briefly describe the mistake or problem and how it happened. *"I use a certain inhaler for COPD. I had run out without realising that I had forgotten to tick it on my repeat prescription. I spoke to the pharmacist and explained to ask him to add it for next time I picked up the repeat prescription. They agreed to do this but when I went to collect it I found that they had ordered a different medicine unrelated to COPD. I was upset because in the meantime my COPD had worsened quite quickly and was causing me distress."*

Could the mistake or problem have been avoided? If so how? *"The chemist should have made a note at the time and written down the medicine that I was asking for. If they had taken the note there and then I don't think this would have happened. I'm assuming he took a note later and failed to remember the name of the medicine correctly. We have a dreadful chemist service here."*

Were you able to talk about the mistake or problem with anybody working in the primary care service? *"No, I was so exasperated I went to my GP to order the medicine directly"*

Patient-reported prospect of harm: prompted via Q10 (Box 1 main paper)

Patient-perspective problem-type code: C1.1.5 Repeat prescription unintentionally changed

Scenario14. GP surgery

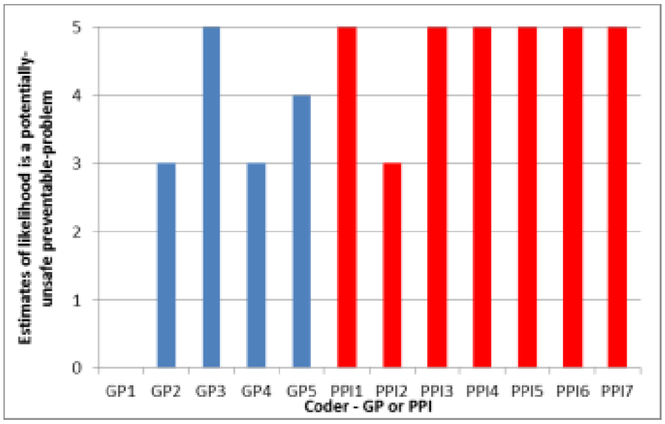

5=very likely or certain, 4=probably, 3=possibly, 2=unlikely, 1=definitely not, 0 = insufficient information or don't know

Briefly describe the mistake or problem and how it happened. *"GP misdiagnosed broken jaw, went to emergency dentist then to A&E where it was operated on and fixed"*

Could the mistake or problem have been avoided? If so how? "*if GP had diagnosed correctly initially"*

Were you able to talk about the mistake or problem with anybody working in the primary care service? *"made complaint to surgery and they wrote back apologising"*

Patient-reported prospect of harm: health was actually made worse by a problem or error that could have been prevented

Patient-perspective problem-type code: F1. Wrong/late/missed/delayed diagnosis

Scenario15. GP surgery

Briefly describe the mistake or problem and how it happened. *"I was having severe nose bleeds for several months and was told it was hay fever. It was cancer."*

Could the mistake or problem have been avoided? If so how? *"My GP could have sent me for a CT scan as soon as my nose bleeds started."*

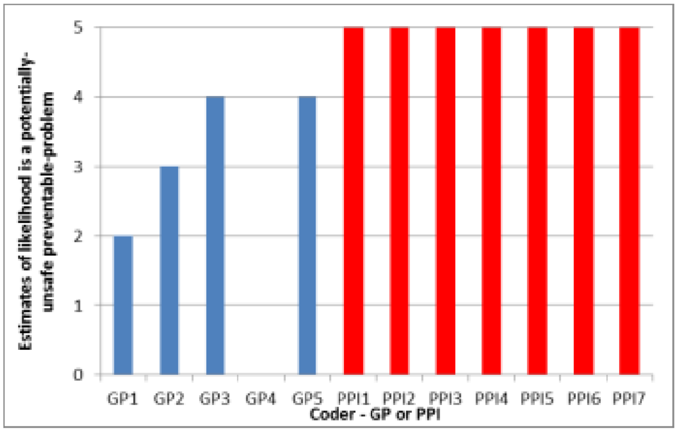

5=very likely or certain, 4=probably, 3=possibly, 2=unlikely, 1=definitely not, 0 = insufficient information or don't know

Were you able to talk about the mistake or problem with anybody working in the primary care service? *"Yes, I registered with a new GP who sent me for a scan straight away which identified my cancer."*

Patient-reported prospect of harm: health was actually made worse by a problem or error that could have been prevented

Patient-perspective problem-type code: F1. Wrong/late/missed/delayed diagnosis

Scenario16. GP surgery

Briefly describe the mistake or problem and how it happened. *"Doctor prescribed tramadol without checking my notes. I'd already taken four pills and I rang up general enquiries at GP service to say I felt disorientated almost as if it was happening to someone else and not me. Got through to my main doctor and asked whether it was wise to take more, she said don't because you might not be alive if you do. She could see I had the wrong dose, disorientation carried on for a couple of days. It was the wrong medication."*

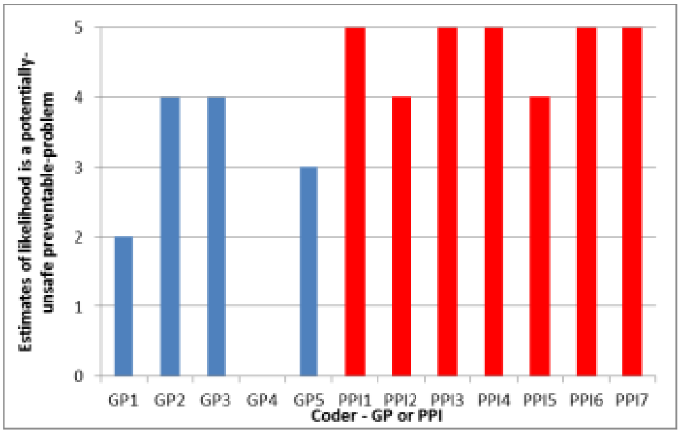

5=very likely or certain, 4=probably, 3=possibly, 2=unlikely, 1=definitely not, 0 = insufficient information or don't know

Could the mistake or problem have been avoided? If so how? *"if he had checked my notes to see what I can and can't take in terms of the actual medication"*

Were you able to talk about the mistake or problem with anybody working in the primary care service? *"discussed it with main doctor who said that she would give me some different pills to take to ease the pain for my trapped nerve in spine and back. She said she would speak to other doctor to see why it happened"*

Patient-reported prospect of harm: health was actually made worse by a problem or error that could have been prevented

Patient-perspective problem-type code: C1.3.1 Wrong dose or drug or delivery method

Scenario17. Out of hours care

Briefly describe the mistake or problem and how it happened. *"Threatened miscarriage. Not given anti-D injection and notes were not consulted" (rhesus-negative patient)*

Could the mistake or problem have been avoided? If so how? *"Notes should have been checked"*

Were you able to talk about the mistake or problem with anybody working in the primary care service? *"Yes, hospital consultant who dealt effectively with situation"*

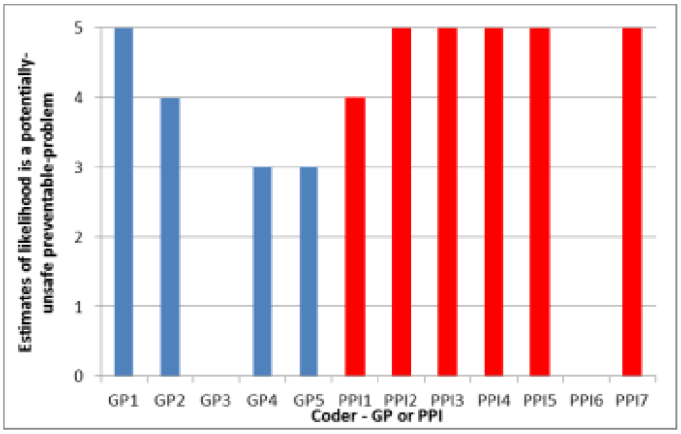

5=very likely or certain, 4=probably, 3=possibly, 2=unlikely, 1=definitely not, 0 = insufficient information or don't know

Patient-reported prospect of harm: there was a problem or error that could have been prevented but it did not make your health worse

Patient-perspective problem-type code: B3 Clinician did not consider patient history sufficiently/did not use patient's notes adequately

Scenario18. GP surgery

Briefly describe the mistake or problem and how it happened. *"Had retained placenta 4 weeks after giving birth. GP dismissed it and went to A&E. Had emergency surgery"*

Could the mistake or problem have been avoided? If so how? "Yes, *by improving GP competence levels"*

Were you able to talk about the mistake or problem with anybody working in the primary care service? *"No, I was too distressed to discuss the problem or error"*

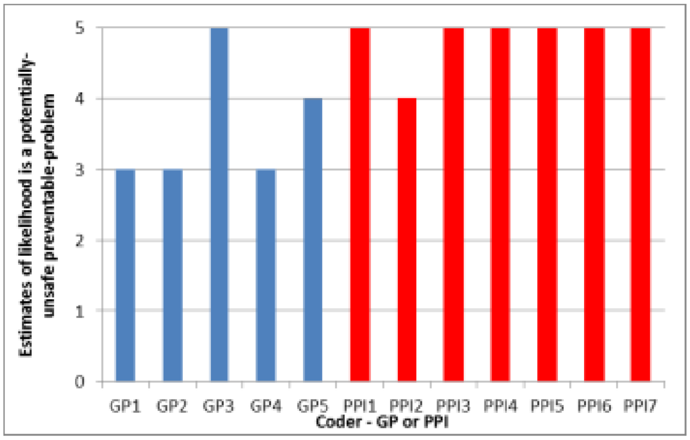

5=very likely or certain, 4=probably, 3=possibly, 2=unlikely, 1=definitely not, 0 = insufficient information or don't know

Patient-reported prospect of harm: there was a problem or error that could have been prevented but it did not make your health worse

Patient-perspective problem-type code: F1. Wrong/late/missed/delayed diagnosis

Scenario19. GP surgery

Briefly describe the mistake or problem and how it happened. *"I had a mole on my arm. It started to itch. I asked the GP if he'd look at it. He said it's fine. Two weeks later I had to see a dermatologist for a different reason. I asked him to look at the mole. He examined it through a magnifying glass. He said he couldn't tell if it was cancerous but recommended me to the local hospital. Two weeks later the hospital informed me the mole was cancerous. They took the mole out immediately. The point is that my GP didn't identify the possible cancer, it was coincidence that I went to the dermatologist who happened to be treating me at the time for a dry skin problem."*

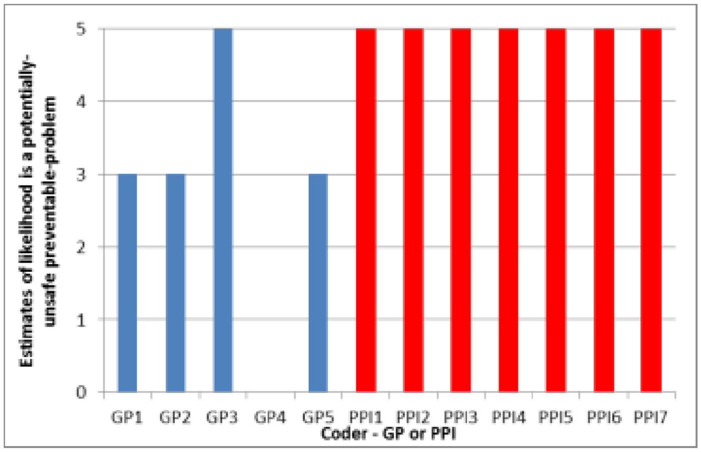

5=very likely or certain, 4=probably, 3=possibly, 2=unlikely, 1=definitely not, 0 = insufficient information or don't know

Could the mistake or problem have been avoided? If so how? *"My GP could have examined me properly rather than just looking at the mole or he could have recommended a specialist if he didn't know what it was"*

Were you able to talk about the mistake or problem with anybody working in the primary care service? *"No, I wasn't confident that they would listen/I felt anything I say would fall on deaf ears"*

Patient-reported prospect of harm: health was actually made worse by a problem or error that could have been prevented

Patient-perspective problem-type code: F1. Wrong/late/missed/delayed diagnosis

Scenario20. GP surgery

Briefly describe the mistake or problem and how it happened. *"appendix problem not diagnosed"*

Could the mistake or problem have been avoided? If so how? *"better diagnostic skills"*

Were you able to talk about the mistake or problem with anybody working in the primary care service? *"Yes, another GP who referred me to hospital"*

Patient-reported prospect of harm: health could have been made worse had someone not noticed a problem or error

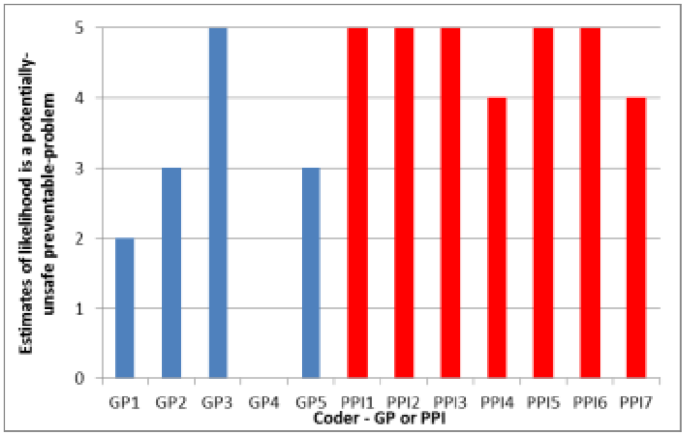

5=very likely or certain, 4=probably, 3=possibly, 2=unlikely, 1=definitely not, 0 = insufficient information or don't know

Patient-perspective problem-type code: F1. Wrong/late/missed/delayed diagnosis

Scenario21. GP surgery

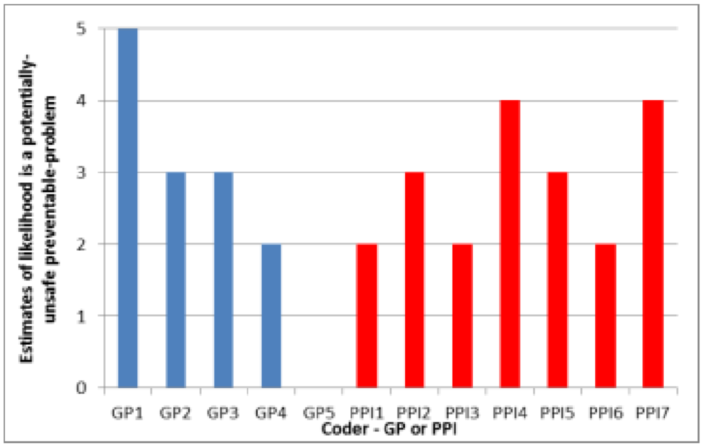

5=very likely or certain, 4=probably, 3=possibly, 2=unlikely, 1=definitely not, 0 = insufficient information or don't know

Briefly describe the mistake or problem and how it happened. *"I had something stuck into my ear, a cotton bud. I went to GP and they booked an appointment with a consultant. After 6 months I didn't hear anything from him. Luckily the cotton bud came out by itself, it could have been worse."*

Could the mistake or problem have been avoided? If so how? *"If I could have an appointment with a consultant he could have checked my ear canal"*

Were you able to talk about the mistake or problem with anybody working in the primary care service? *"No, I could not find anybody with whom I could discuss the problem or error"*

Patient-reported prospect of harm: health could have been made worse had someone not noticed a problem or error

Patient-perspective problem-type code: A3. Intended referral was not sent or delayed

Scenario22. A&E

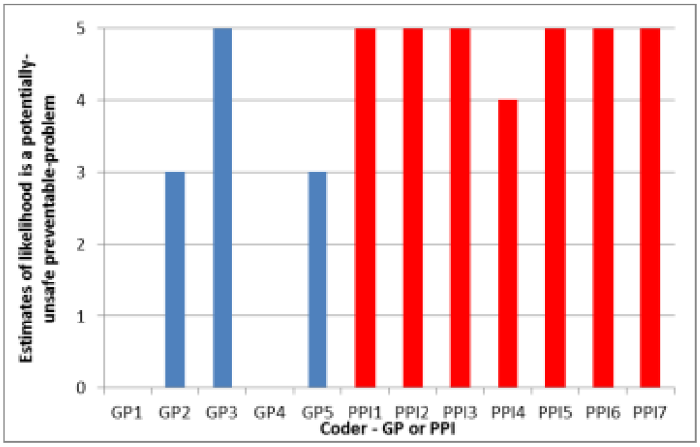

5=very likely or certain, 4=probably, 3=possibly, 2=unlikely, 1=definitely not, 0 = insufficient information or don't know

Briefly describe the mistake or problem and how it happened. "*Basically told me problem was biliary spasms / colic but it was actually a hole in my stomach"*

Could the mistake or problem have been avoided? If so how? "If the doctor had taken heed of blood results - he ignored blood results - ended in emergency surgery"

Were you able to talk about the mistake or problem with anybody working in the primary care service? *"No, I was too distressed to discuss the problem or error"*

Patient-reported prospect of harm: health was actually made worse by a problem or error that could have been prevented

Patient-perspective problem-type code: F1. Wrong/late/missed/delayed diagnosis

Scenario23. GP surgery

Briefly describe the mistake or problem and how it happened. *"I have been diagnosed with bowel cancer, I knew something was wrong but over 4 visits to GP surgery over a 2 week period I was fobbed off by the GP who told me it was probably gastritis, it took 2 weeks to get a referral to a specialist"*

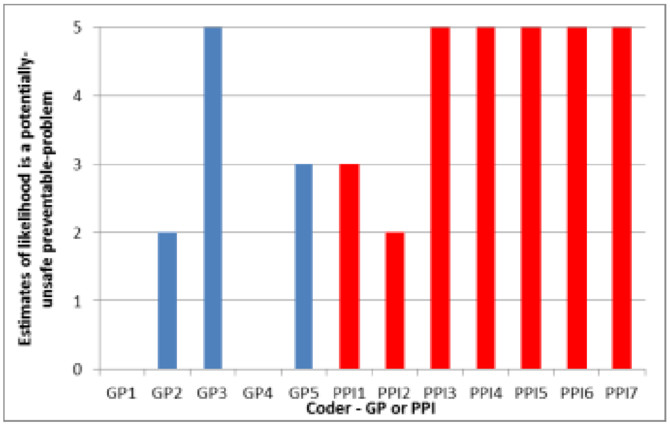

5=very likely or certain, 4=probably, 3=possibly, 2=unlikely, 1=definitely not, 0 = insufficient information or don't know

Could the mistake or problem have been avoided? If so how? *"I feel it was obvious from my appearance - massively distended stomach that - something serious was wrong with me, by the time I finally was referred I was seriously ill, this could have been avoided by an x-ray or quicker referral"*

Were you able to talk about the mistake or problem with anybody working in the primary care service? *"Yes, district nurse, who told me there is a framework in place for GPs that they have to stick to whilst diagnosing issues"*

Patient-reported prospect of harm: health was actually made worse by a problem or error that could have been prevented

Patient-perspective problem-type code: F1. Wrong/late/missed/delayed diagnosis

Scenario24. GP surgery

Briefly describe the mistake or problem and how it happened. *"Low blood count not identified because doctor didn't do blood test. Taken to hospital, died and brought back to life"*

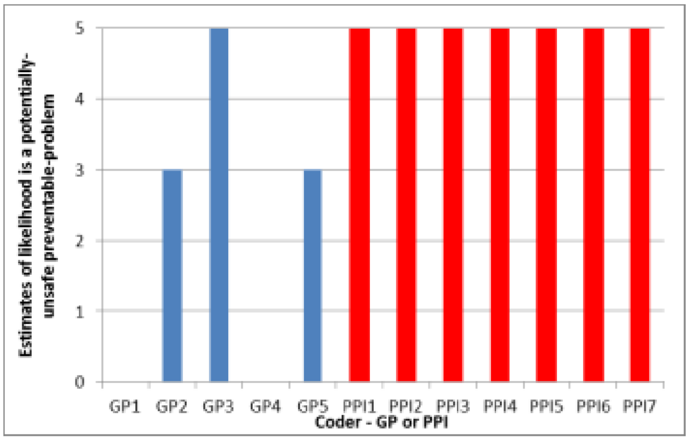

5=very likely or certain, 4=probably, 3=possibly, 2=unlikely, 1=definitely not, 0 = insufficient information or don't know

Could the mistake or problem have been avoided? If so how? *"a different drug should have been given"*

Were you able to talk about the mistake or problem with anybody working in the primary care service? *"Yes, the doctor"*

Patient-reported prospect of harm: health was actually made worse by a problem or error that could have been prevented

Patient-perspective problem-type code: B4. Investigation not thorough enough

Scenario25. GP surgery

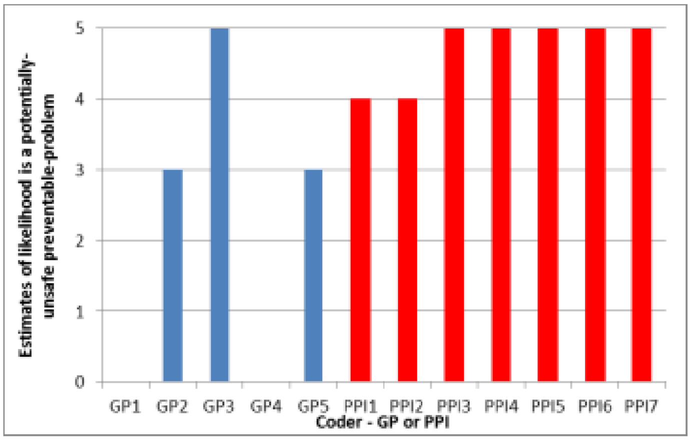

5=very likely or certain, 4=probably, 3=possibly, 2=unlikely, 1=definitely not, 0 = insufficient information or don't know

Briefly describe the mistake or problem and how it happened. *"Had lump on back and thought was an abscess. Went to GP for antibiotics was told "nothing there, it was in my head". Three days later had to have an emergency operation to remove it."*

Could the mistake or problem have been avoided? If so how? *"by correct diagnosis"*

Were you able to talk about the mistake or problem with anybody working in the primary care service? *"No, I had the opportunity but did not feel comfortable discussing the problem or error"*

Patient-reported prospect of harm: health was actually made worse by a problem or error that could have been prevented

Patient-perspective problem-type code: F1. Wrong/late/missed/delayed diagnosis

Scenario26. GP surgery

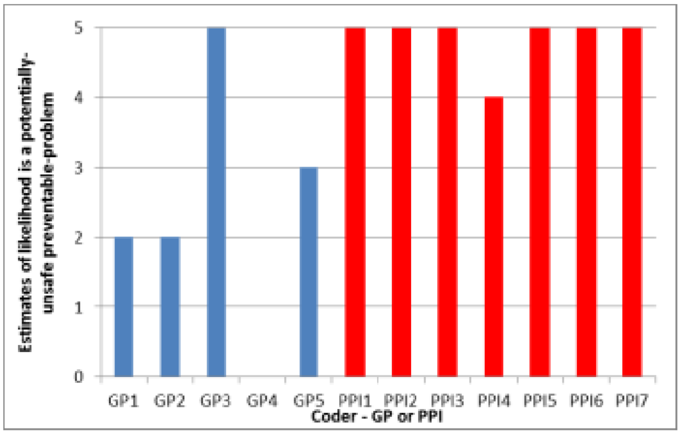

5=very likely or certain, 4=probably, 3=possibly, 2=unlikely, 1=definitely not, 0 = insufficient information or don't know

Briefly describe the mistake or problem and how it happened. "I *had gall stones and they told me it was indigestion. Pain increased over three months. Had to have an emergency operation to have my gall bladder removed. Resulted in me having damage to my liver and pancreatitis"*

Could the mistake or problem have been avoided? If so how? *"listened to me when I told them it wasn't indigestion which would have been nice. The pain felt like I was having a heart attack and not like the pain from eating something dodgy"*

Were you able to talk about the mistake or problem with anybody working in the primary care service? *"No, I could not find anybody with whom I could discuss the problem or error"*

Patient-reported prospect of harm: health was actually made worse by a problem or error that could have been prevented

Patient-perspective problem-type code: F1. Wrong/late/missed/delayed diagnosis; D1. Clinician seemed to lack interest in the patient's health problem or did not listen carefully enough

Scenario27. GP surgery

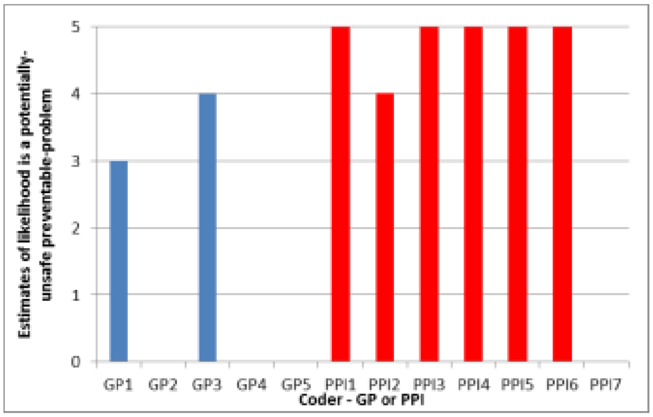

5=very likely or certain, 4=probably, 3=possibly, 2=unlikely, 1=definitely not, 0 = insufficient information or don't know

Briefly describe the mistake or problem and how it happened. *"I have arthritis and I was prescribed a medication, Diclofenac, an anti-inflammatory. After taking this, I had problems and went to the GP and had a blood test. They lost the results and I became even more ill and when I rang them, they told me I was allergic to Diclofenac and I was to stop taking it immediately. It was causing kidney failure, liver failure and high blood pressure."*

Could the mistake or problem have been avoided? If so how? *"They shouldn't have lost the results of the blood test. Later when I was feeling worse and I rang them up, they had found the results but not let me know which was another week later. They should have rung me not the other way round. That was poor communication. There should have been a better way of letting me know the results of the blood test. Luck for me, I was feeling so ill that I stopped taking the Diclofenac which they should have told me I was allergic to"*

Were you able to talk about the mistake or problem with anybody working in the primary care service? *"No, I know they're busy and there are people who need their help more than I do"*

Patient-reported prospect of harm: prompted via Q10 (Box 1 main paper)

Patient-perspective problem-type code: C1.1.3 Long term or continued prescribing without review or consideration of long term or side effects; B1. Test results lost or other problem with investigation paperwork

Scenario28. GP surgery

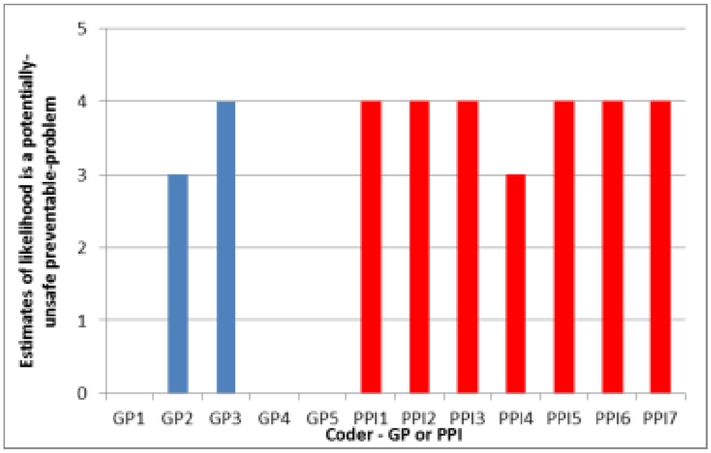

5=very likely or certain, 4=probably, 3=possibly, 2=unlikely, 1=definitely not, 0 = insufficient information or don't know

Briefly describe the mistake or problem and how it happened. *"I had stomach pains and was given the wrong medication which made it worse"*

Could the mistake or problem have been avoided? If so how? *"If I had had more tests the problem could have been avoided."*

Were you able to talk about the mistake or problem with anybody working in the primary care service? *"Yes, another doctor and they advised me to stop taking the medication"*

Patient-reported prospect of harm: health was actually made worse by a problem or error that could have been prevented

Patient-perspective problem-type code: C1.1.1 Prescribed wrong or inappropriate drug; B4. Investigation not thorough enough

Scenario29. GP surgery

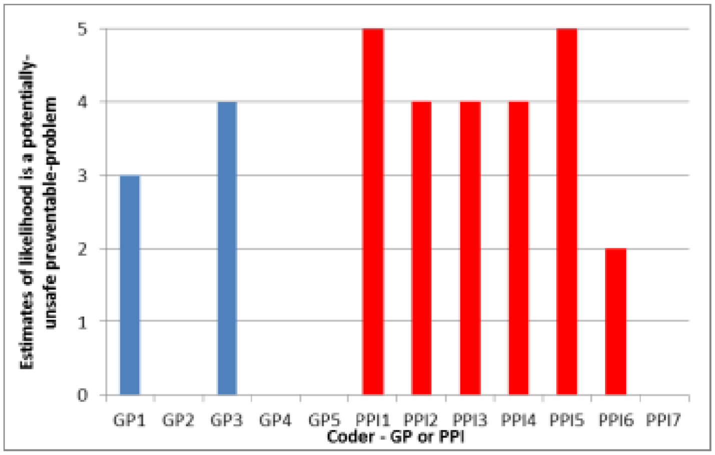

5=very likely or certain, 4=probably, 3=possibly, 2=unlikely, 1=definitely not, 0 = insufficient information or don't know

Briefly describe the mistake or problem and how it happened. *"I went to the GP and had a blood test. A month later they rang me up to tell me they had forgotten to tell me I had streptococcus and should have been on an antibiotic. In the intervening month I was ill without having taken the antibiotic"*

Could the mistake or problem have been avoided? If so how? *"Maybe they should have taken more care of their records and follow up"*

Were you able to talk about the mistake or problem with anybody working in the primary care service? *"No, I did not notice the problem or error at the time"*

Patient-reported prospect of harm: prompted via Q10 (Box 1 main paper)

Patient-perspective problem-type code: F1. Wrong/late/missed/delayed diagnosis; B1. Test results lost or other problem with investigation paperwork

Scenario30. Pharmacy

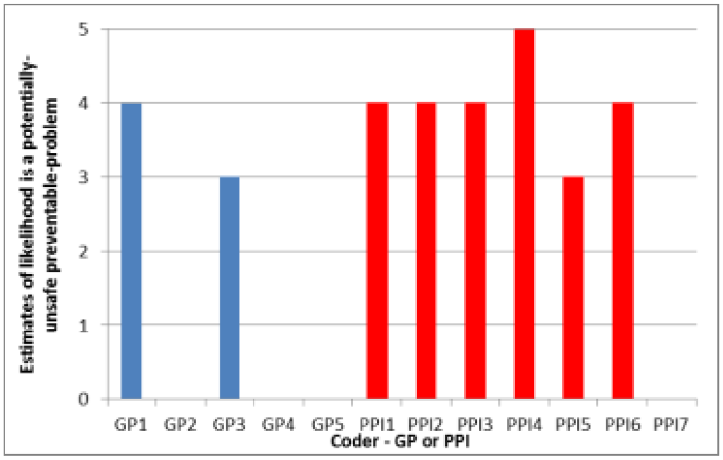

5=very likely or certain, 4=probably, 3=possibly, 2=unlikely, 1=definitely not, 0 = insufficient information or don't know

Briefly describe the mistake or problem and how it happened. *"It was routine prescription for blood pressure pills and they handed them over in a box in a stapled bag and when I got home I saw it was somebody else's medicine with my address label on. My husband took it back and they exchanged it for the correct medicine. About two weeks later we received a letter of apology which said the pharmacy had "put procedures in place so that the mistake wouldn't happen again". We were happy with that."*

Could the mistake or problem have been avoided? If so how? *"I don't know how the problem happened at the pharmacy. Perhaps somebody at the pharmacy could check each prescription before it's issued. Perhaps I could have checked it myself."*

Were you able to talk about the mistake or problem with anybody working in the primary care service? *"Yes, their response was the letter of apology."*

Patient-reported prospect of harm: prompted via Q10 (Box 1 main paper)

Patient-perspective problem-type code: C1.3.2 Being given another patient's drugs or prescription

Scenario31. Pharmacy

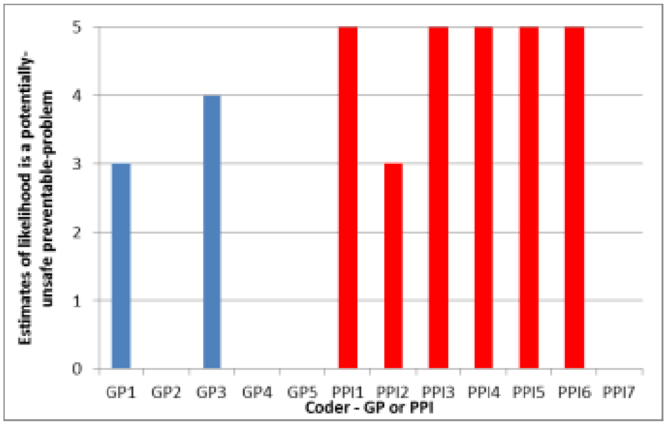

5=very likely or certain, 4=probably, 3=possibly, 2=unlikely, 1=definitely not, 0 = insufficient information or don't know

Briefly describe the mistake or problem and how it happened. *"The GP prescribed particular blood pressure tablets. The pharmacist at Boots changed the GPs prescription for a different tablet which had an adverse effect on me. It made me sick, headaches and dizziness. I went back to the GP who confirmed they were the wrong tablets and that the pharmacist isn't allowed to change a particular make of tablet. I went back to Boots and the pharmacist said they had stopped making the tablets my GP prescribed. I phoned the makers of the tablets and found that the tablets are still made. I remonstrated with the pharmacist who banned me from the shop and threatened to have me physically removed from the shop. I had been using the shop for over 40 years. I came home and phoned Boots head office and told them I would report the incident to my local newspaper and TV. I phoned the newspaper and TV wanted to film me outside the shop but a director from Boots came to my home to apologise personally and the pharmacist was forced to ring me to apologise. The pharmacist agreed that they were in breach of contract by changing the GPs prescription. When they apologised I regarded that as the end of the matter. For the last 3 months they have provided the correct tablets and on time."*

Could the mistake or problem have been avoided? If so how? *"The pharmacy is far too busy and they've exceeded their capability. Their ordering procedure means they too often run out of the correct tablets"*

Were you able to talk about the mistake or problem with anybody working in the primary care service? *"Yes, Chemist / Pharmacist, they admitted that previous medicine was wrong*

Patient-reported prospect of harm: prompted via Q10 (Box 1 main paper)

Patient-perspective problem-type code: C1.2.1 Medication not dispensed or administered as intended or prescribed

Scenario32. Pharmacy

Briefly describe the mistake or problem and how it happened. *"Wrong prescription tablets issued in error, name of patient was correct but the tablets were totally incorrect."*

Could the mistake or problem have been avoided? If so how? "*Pharmacy should have taken more care"*

Were you able to talk about the mistake or problem with anybody working in the primary care service? "Yes, *spoke to pharmacist and correct prescription was issued"*

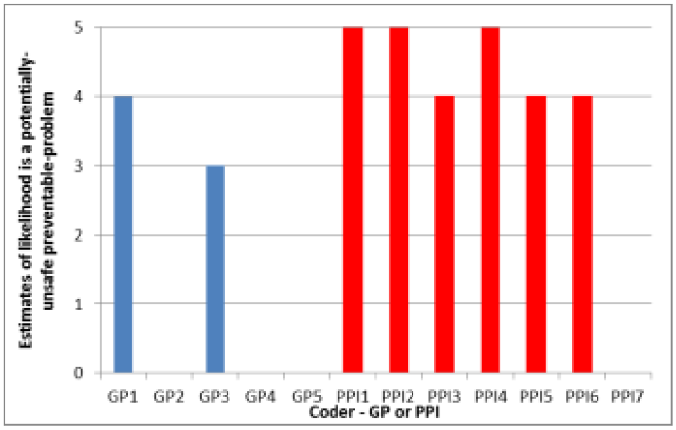

5=very likely or certain, 4=probably, 3=possibly, 2=unlikely, 1=definitely not, 0 = insufficient information or don't know

Patient-reported prospect of harm: prompted via Q10 (Box 1 main paper)

Patient-perspective problem-type code: C1.2.1 Medication not dispensed or administered as intended or prescribed

Scenario33. GP surgery

Briefly describe the mistake or problem and how it happened. *"had ear problem and GP provided treatment for 2 years but no response to medication. Within one month of being referred and treated by specialist the problem cleared up"*

Could the mistake or problem have been avoided? If so how? "*by earlier referral to specialist"*

Were you able to talk about the mistake or problem with anybody working in the primary care service? *"No, I could not find anybody with whom I could discuss the problem or error"*

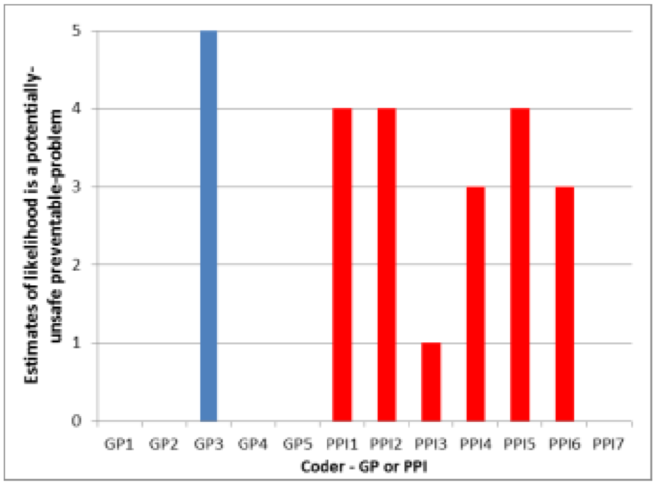

5=very likely or certain, 4=probably, 3=possibly, 2=unlikely, 1=definitely not, 0 = insufficient information or don't know

Patient-reported prospect of harm: prompted via Q10 (Box 1 main paper)

Patient-perspective problem-type code: B5. Not referred when patient felt was needed

<u>Patient reported scenarios occurring during the past 12 months that clinicians scored as higher likelihood to be a potentially-unsafe preventable-problem in primary care (median score is higher than "possibly" and at least 2 clinicians gave a score or one clinician scored "very likely or certain") from the pilot study (reference 24)</u>

Scenario34. GP surgery

Briefly describe the mistake or problem and how it happened. *"Prescription drug, anti-inflammatory for arthritis, caused acute stomach pains & violent vomiting. Repeat prescription for twelve years without any discussion."*

Could the mistake or problem have been avoided? If so how? "*Possible discussion about dangers of continuous taking of prescription drugs, which in the event were stopped after the incident."*

Were you able to talk about the mistake or problem with anybody working in the primary care service? *"No I did not notice the mistake or problem at the time"*

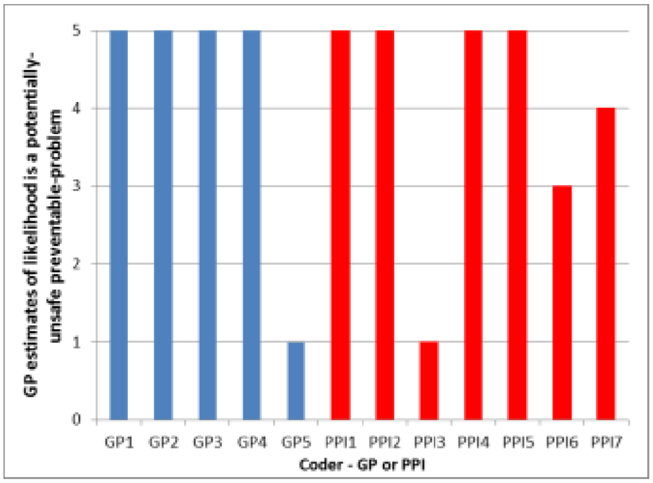

5=very likely or certain, 4=probably, 3=possibly, 2=unlikely, 1=definitely not, 0 = insufficient information or don't know

Patient-reported prospect of harm: suspected your health has been made worse by a problem or error that could have been prevented

Patient-perspective problem-type code: C1.1.3 Long term or continued prescribing without review or consideration of long term or side effects

Scenario35. GP surgery

Briefly describe the mistake or problem and how it happened. *"Insulin type was changed by specialist but previous insulin prescribed by GP as notes had not been updated"*

Could the mistake or problem have been avoided? If so how? "*Yes GP notes should have been updated with new medication"*

Were you able to talk about the mistake or problem with anybody working in the primary care service? *"Practice manager resolved the problem and apologised"*

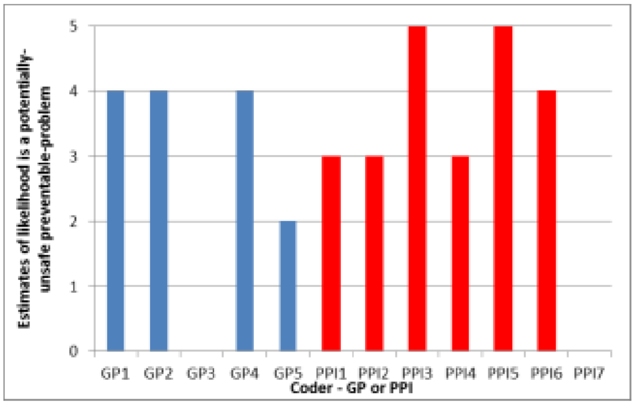

5=very likely or certain, 4=probably, 3=possibly, 2=unlikely, 1=definitely not, 0 = insufficient information or don't know

Patient-reported prospect of harm: prompted via Q10 (Box 1 main paper)

Patient-perspective problem-type code: A2. Incorrect notes/inadequate notes/notes not kept up to date; C1.1.6 Out of date repeat prescription mistakenly re-issued

Scenario36. GP surgery

Briefly describe the mistake or problem and how it happened. *"Two out of three Doctors not listening to what I was asking; April I had two big bleeds from my Penis, Doctor 1 did a test and gave antibiotics. Went to 2nd Doctor for Diabetic check and told him of problem - nothing except another test come back in ten days. Went to the third doctor who said the test didn't show anything but when I mentioned my feelings about a problem, he look and said yes you do have a problem. In 2 weeks I was in having tests and 3 operations for cancer."*

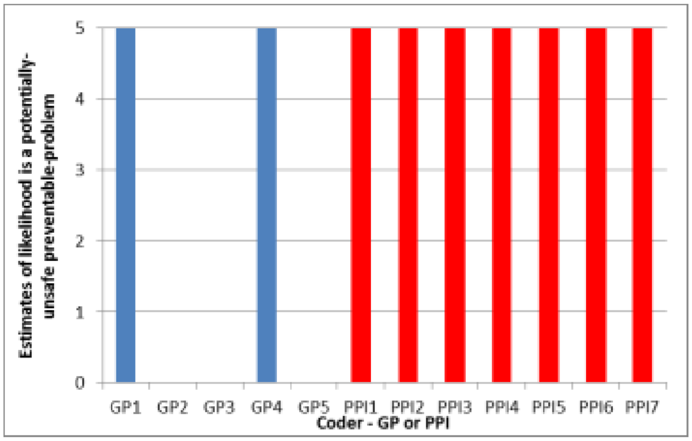

5=very likely or certain, 4=probably, 3=possibly, 2=unlikely, 1=definitely not, 0 = insufficient information or don't know

Could the mistake or problem have been avoided? If so how? "*Listen to me"*

Were you able to talk about the mistake or problem with anybody working in the primary care service? *"No, I could not find anybody with whom I could discuss the mistake or problem (The third doctor was amazing with me. He said to keep in touch and if I had any problems to ring him and he still wants me to ring him after my three operations.)"*

Patient-reported prospect of harm: suspected your health has been made worse by a problem or error that could have been prevented

Patient-perspective problem-type code: D1. Clinician seemed to lack interest in the patient's health problem or did not listen carefully enough; F1. Wrong/late/missed/delayed diagnosis

Scenario37. GP surgery

Briefly describe the mistake or problem and how it happened. *"Changed diabetes medication to an alternative which my notes from 1980's should show I respond badly to"*

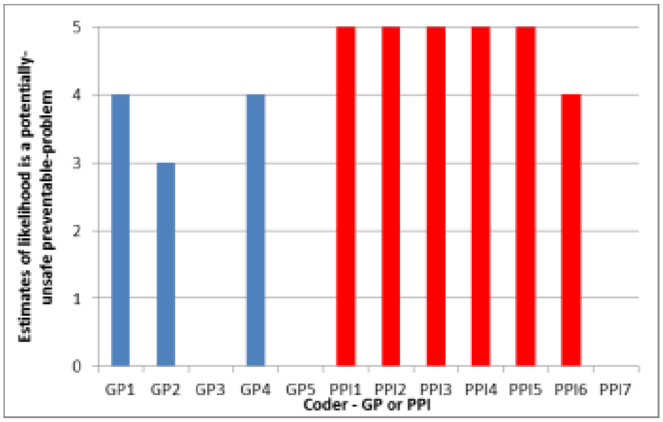

5=very likely or certain, 4=probably, 3=possibly, 2=unlikely, 1=definitely not, 0 = insufficient information or don't know

Could the mistake or problem have been avoided? If so how? "*Read the notes on every medication change but unfortunately that is unrealistic under the time restrictions on GP's. Put early notes on-line and flag medication allergies/problems."*

Were you able to talk about the mistake or problem with anybody working in the primary care service? *"Yes, my own GP who had returned from holiday"*

Patient-reported prospect of harm: suspected your health has been made worse by a problem or error that could have been prevented

Patient-perspective problem-type code: C1.1.4 Prescribed drug when should have known contra-indicated *e.g.* patient had informed clinician of allergy, adverse reaction or it was in the records

Scenario38. GP surgery

Briefly describe the mistake or problem and how it happened. *"Told the GP the medication was making my hair fall out & he kept me on it for another 3 months. I had to see another GP to get him to change my medication. In the meantime I have lost 3/4 of my hair. Not sure if it will ever grow back."*

Could the mistake or problem have been avoided? If so how? "*yes, by the GP listening to what I was saying."*

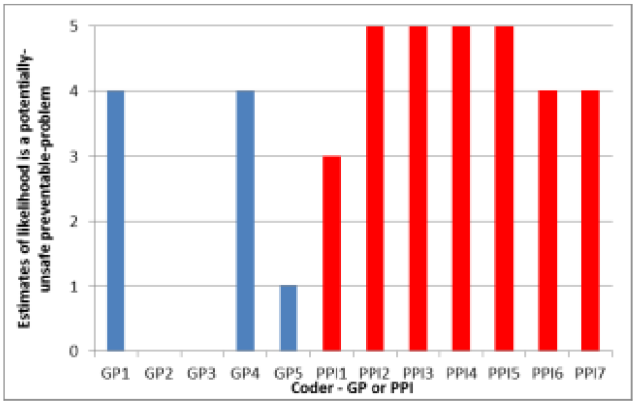

5=very likely or certain, 4=probably, 3=possibly, 2=unlikely, 1=definitely not, 0 = insufficient information or don't know

Were you able to talk about the mistake or problem with anybody working in the primary care service? *"Yes, GP"*

Patient-reported prospect of harm: suspected your health has been made worse by a problem or error that could have been prevented

Patient-perspective problem-type code: D1. Clinician seemed to lack interest in the patient's health problem or did not listen carefully enough; C1.1.3 Long term or continued prescribing without review or consideration of long term or side effects

Scenario39. GP surgery

Briefly describe the mistake or problem and how it happened. *"Successfully treated for prostate cancer 2006 but suffered some loss of sexual performance; Viagra recommended BUT I take isosorbide nitrate for a following heart attack; the two are contradictory and could produce further heart problems. A routine diabetes check-up at which the sexual problem was discussed saw an automatic prescribing of Viagra; obviously without reference to my medical records."*

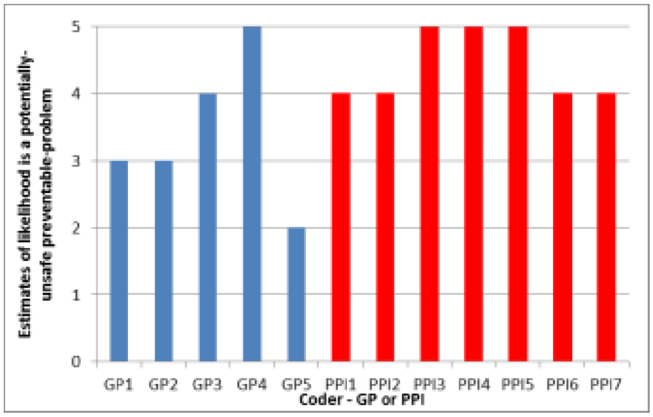

Could the mistake or problem have been avoided? If so how? "*Read the medical notes."*

5=very likely or certain, 4=probably, 3=possibly, 2=unlikely, 1=definitely not, 0 = insufficient information or don't know

Were you able to talk about the mistake or problem with anybody working in the primary care service? *"No; I felt I was going to cause trouble"*

Patient-reported prospect of harm: prompted via Q10 (Box 1 main paper)

Patient-perspective problem-type code: C1.1.1 Prescribed wrong or inappropriate drug

Scenario40. GP surgery

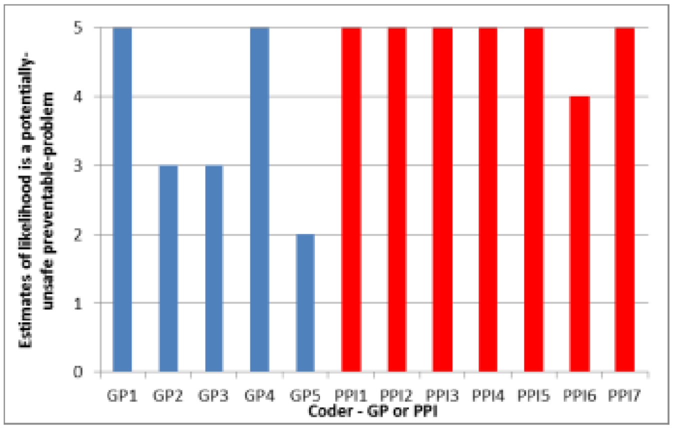

5=very likely or certain, 4=probably, 3=possibly, 2=unlikely, 1=definitely not, 0 = insufficient information or don't know

Briefly describe the mistake or problem and how it happened. *"I was given steroids for a chest infection but not alerted to the fact they make your sugars go massively high! Within a few hours I was high and not able to bring them down, fearing a DKA I headed for the hospital to correct a very easily avoidable issue. I also attended my GP 6 years ago to be given strong antacids for pain in my stomach that was actually a DKA I was admitted to hospital a few hours later! The GP never even suggested it could be linked to my diabetes and as it was my first DKA I had no idea that's how they can feel"*

Could the mistake or problem have been avoided? If so how? "*Both could have been avoided The steroids - if the prescribing nurse had considered my diabetes I'd have been given proper advice as to how to deal with them as a diabetic or given different meds. The DKA simple questions or explanation as to how DKAs can present would have made me family and the doctor realise I was in trouble."*

Were you able to talk about the mistake or problem with anybody working in the primary care service? *"I wrote a letter to the surgery concerning the steroids anonymously to alert them of my concern and the DKA. I was too poorly to even consider seeking correction or explanation"*

Patient-reported prospect of harm: health was actually made worse by a problem or error that could have been prevented

Patient-perspective problem-type code: C1.1.4 Prescribed drug when should have known contra-indicated *e.g.* patient had informed clinician of allergy, adverse reaction or it was in the records; E3. Incorrect advice/no advice given by clinician

Scenario41. GP surgery

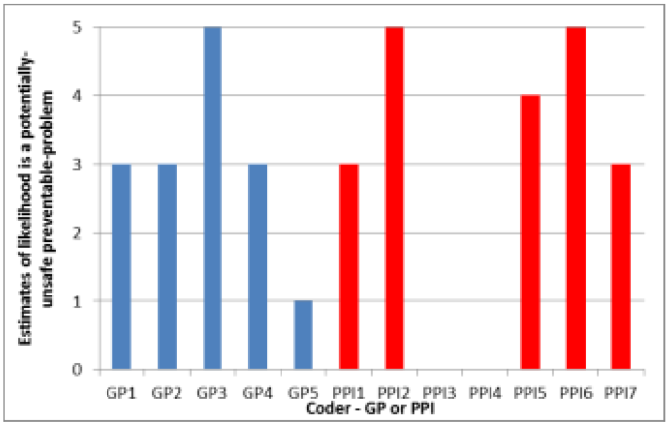

5=very likely or certain, 4=probably, 3=possibly, 2=unlikely, 1=definitely not, 0 = insufficient information or don't know

Briefly describe the mistake or problem and how it happened. *"reception staff making clinical decisions which were at odds with what had been discussed with my GP"*

Could the mistake or problem have been avoided? If so how? "*Yes, reception staff shouldn't be making clinical decisions"*

Were you able to talk about the mistake or problem with anybody working in the primary care service? *"No, had the opportunity but did not feel comfortable to discuss the mistake or problem"*

Patient-reported prospect of harm: suspected your health has been made worse by a problem or error that could have been prevented

Patient-perspective problem-type code: E1. Administrative staff seemed to make clinical decisions

Scenario42. Pharmacist

Briefly describe the mistake or problem and how it happened. *"I was given a medicine belonging to somebody else as part of my monthly repeat prescription"*

Could the mistake or problem have been avoided? If so how? "*More care and attention when checking"*

Were you able to talk about the mistake or problem with anybody working in the primary care service? *"Yes, pharmacist"*

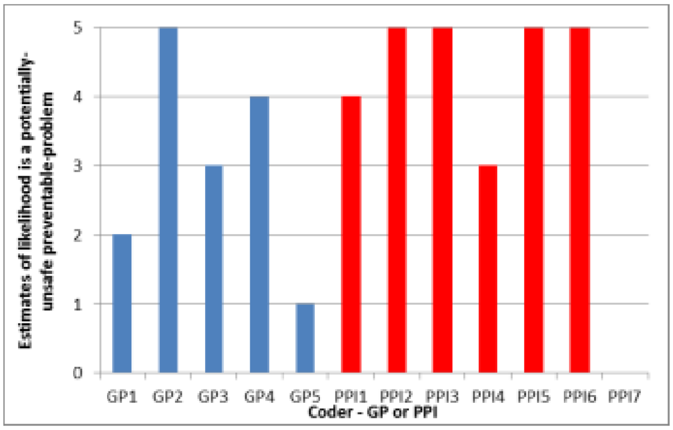

5=very likely or certain, 4=probably, 3=possibly, 2=unlikely, 1=definitely not, 0 = insufficient information or don't know

Patient-reported prospect of harm: prompted via Q10 (Box 1 main paper)

Patient-perspective problem-type code: C1.3.3 Wrong or inadequate advice about drug effects or how to use

Scenario43. GP surgery

Briefly describe the mistake or problem and how it happened. *"Poor diabetic annual review, foot check not correctly done just tested my foot pulses and nothing else"*

Could the mistake or problem have been avoided? If so how? "*Better training of staff"*

Were you able to talk about the mistake or problem with anybody working in the primary care service? *"No, had the opportunity but did not feel comfortable to discuss the mistake or problem"*

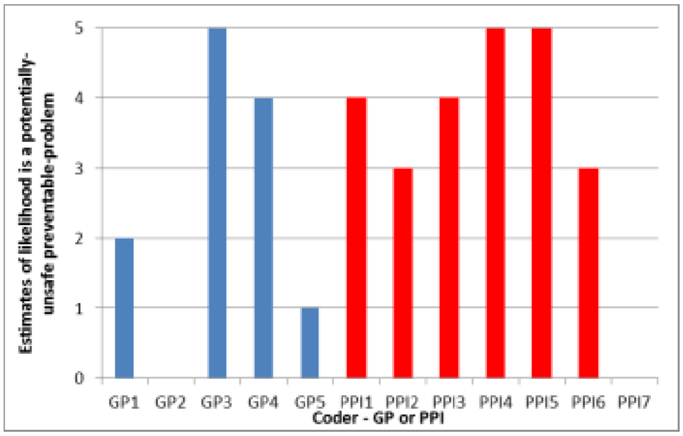

5=very likely or certain, 4=probably, 3=possibly, 2=unlikely, 1=definitely not, 0 = insufficient information or don't know

Patient-reported prospect of harm: suspected your health has been made worse by a problem or error that could have been prevented

Patient-perspective problem-type code: E2. Procedure was not carried out correctly

Scenario44. GP surgery

Briefly describe the mistake or problem and how it happened. *"Prior to a pain killing injection into my knee, I asked the GP who suggested the injection AND the GP who carried out the injection whether, as someone living with Type 1 diabetes, it would have any effect on my blood glucose levels. On both occasions, I was given an unequivocal No . In the event, within a few hours of the injection, my blood glucose rose significantly and remained high for several days. I felt unable to eat anything for 24 hours while I took on more and more insulin in order to bring my glucose levels down - I did not want to go to sleep that night simply because of the massive amount of insulin in my system."*

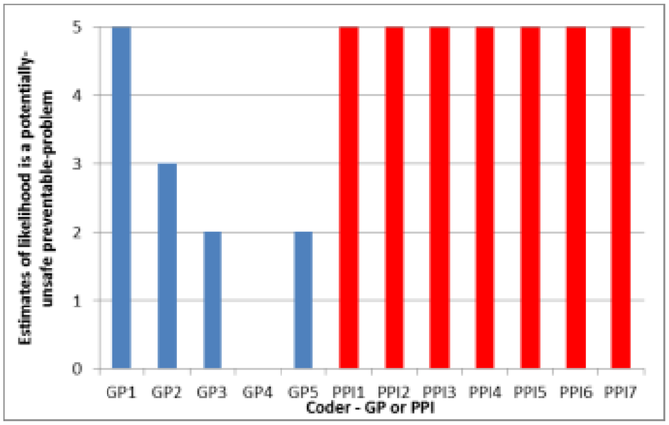

5=very likely or certain, 4=probably, 3=possibly, 2=unlikely, 1=definitely not, 0 = insufficient information or don't know

Could the mistake or problem have been avoided? If so how? "*Yes. I feel that both GPs should have a knowledge about the side effects of drugs they prescribe, administer and recommend."*

Were you able to talk about the mistake or problem with anybody working in the primary care service? *"No I could not find anybody with whom I could discuss the mistake or problem"*

Patient-reported prospect of harm: your health has been made worse by a problem or error that could have been prevented

Patient-perspective problem-type code: E3. Incorrect advice/no advice given by clinician

Scenario45. GP surgery

Briefly describe the mistake or problem and how it happened. *"GP completely overlooked symptoms and prescribed antibiotic after antibiotic without investigation or referral"*

Could the mistake or problem have been avoided? If so how? "*Yes by listening to history of complaints, carrying out appropriate tests instead of just giving antibiotics"*

Were you able to talk about the mistake or problem with anybody working in the primary care service? *"No I did not notice the mistake or problem at the time"*

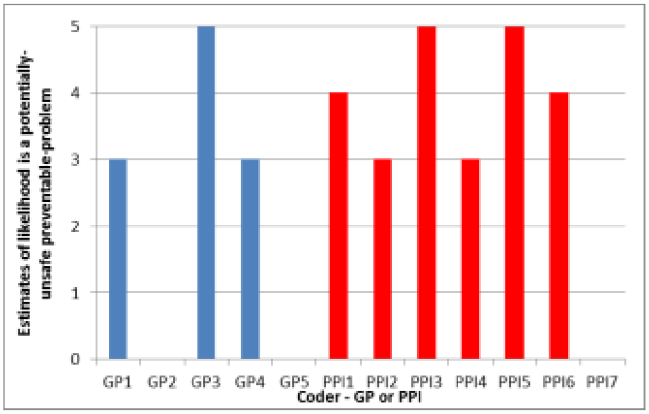

5=very likely or certain, 4=probably, 3=possibly, 2=unlikely, 1=definitely not, 0 = insufficient information or don't know

Patient-reported prospect of harm: prompted via Q10 (Box 1 main paper)

Patient-perspective problem-type code: D1. Clinician seemed to lack interest in the patient's health problem or did not listen carefully enough; F1. Wrong/late/missed/delayed diagnosis

Scenario46. GP surgery

Briefly describe the mistake or problem and how it happened. *"Several times prescriptions have been incorrectly issued due to similar names for drugs or the same name with different strengths"*

Could the mistake or problem have been avoided? If so how? "*Yes, by more accurate or double data entry. Now solved by self-request using web systems."*

Were you able to talk about the mistake or problem with anybody working in the primary care service? "Yes, *they did not want to know or seem to care unless a formal complaint was made"*

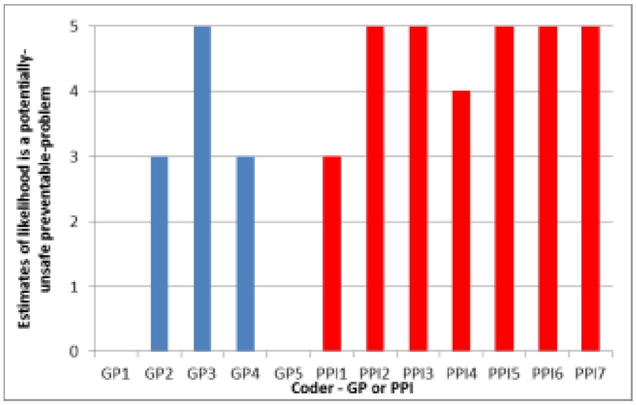

5=very likely or certain, 4=probably, 3=possibly, 2=unlikely, 1=definitely not, 0 = insufficient information or don't know

Patient-reported prospect of harm: your health has been made worse by a problem or error that could have been prevented

Patient-perspective problem-type code: C1.1.5 Repeat prescription unintentionally changed

Scenario47. GP surgery

Briefly describe the mistake or problem and how it happened. *"A simple error occurred with an incorrect prescription. When I tried to bring this to the attention of the receptionist she treated me with disdain and in a challenging manner. She then proceeded to start to read my notes aloud in the public reception area. I felt that this was unacceptable behaviour. When I tried to tackle the receptionist about her behaviour I felt as if I was under threat. It caused me to feel very stressed, frustrated and ill tempered."*

Could the mistake or problem have been avoided? If so how? "*If the receptionist had been willing to listen to what I was saying."*

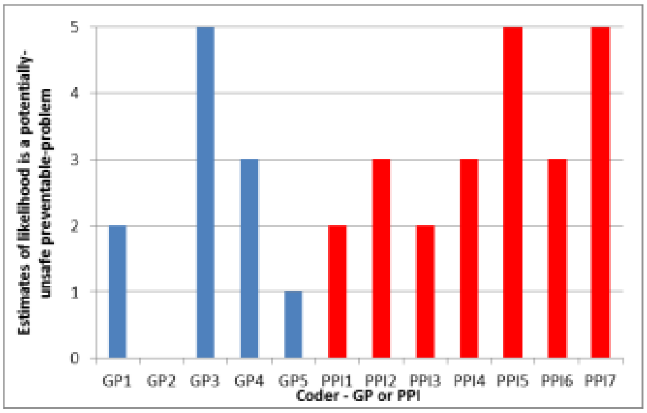

5=very likely or certain, 4=probably, 3=possibly, 2=unlikely, 1=definitely not, 0 = insufficient information or don't know

Were you able to talk about the mistake or problem with anybody working in the primary care service? "*I did speak to a lady who said she was the practice manager but I felt that they were not interested in resolving the problem"*

Patient-reported prospect of harm: suspected your health has been made worse by a problem or error that could have been prevented

Patient-perspective problem-type code: D3. Communication problem between patient and primary care staff; C1 Medication error not otherwise specified /other problem

Scenario48. GP Surgery

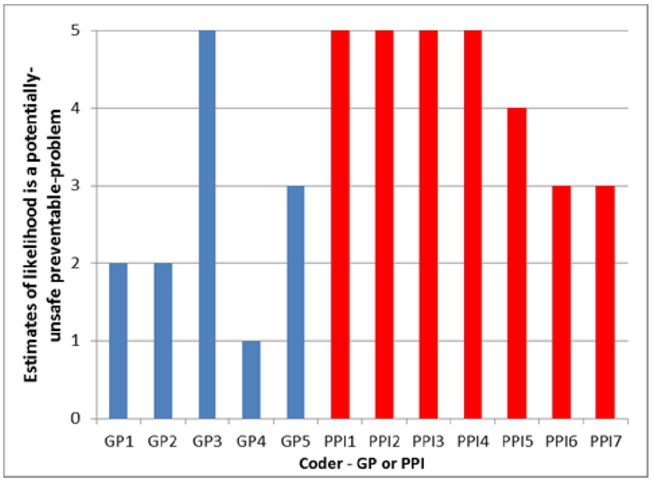

5=very likely or certain, 4=probably, 3=possibly, 2=unlikely, 1=definitely not, 0 = insufficient information or don't know

Briefly describe the mistake or problem and how it happened. *"Went to see GP because I feared the pain in one of my legs may have been Peripheral Artery Disease - hardening of the arteries, having had a (non-blood) relative who suffered from this and subsequently died - of a heart attack. Oh yes, said the GP, well, you will have it won't you? Why? I asked expecting her to say eg because you are a smoker, or maybe my age (65) or something else I wasn't aware of. But what she actually told me was 'Because you are a diabetic!' Whaaat? I exclaimed - you mean ALL diabetics will inevitably get this, and there's no way to prevent it? Yes she said and shrugged. I said 'Thanks for nothing then' and left. Instead I left, came home and went straight on-line to make an appointment with someone more sensible, which I did and after taking my leg/ankle pulses and BPs etc - he chatted to me and said he would refer me for a cardiology consultation at the hospital. This IS what I expected in the first place and now it IS being taken care of."*

Could the mistake or problem have been avoided? If so how? *"By training the GP properly in the first place"*

Were you able to talk about the mistake or problem with anybody working in the primary care service? "? *"I explained to GP 2 But I don't know what if anything was done about it, or how I could find that out."*

Patient-reported prospect of harm: your health has been made worse by a problem or error that could have been prevented

Patient-perspective problem-type code: D1. Clinician seemed to lack interest in the patient's health problem or did not listen carefully enough

Patient reported scenarios occurring during the past 12 months that PPIs scored as higher likelihood to be a potentially-unsafe preventable-problem in primary care compared with clinicians – Ipsos MORI survey

Scenario49. GP surgery

Briefly describe the mistake or problem and how it happened. *"I was suicidal, phoned the crisis team and they kept telling me that they couldn't see me because I wasn't under a psychiatrist and that made the situation worse"*

Could the mistake or problem have been avoided? If so how? *"they just simply had to say that they would see me"*

Were you able to talk about the mistake or problem with anybody working in the primary care service?

"No, I did not get to see a psychiatrist until about three months later"

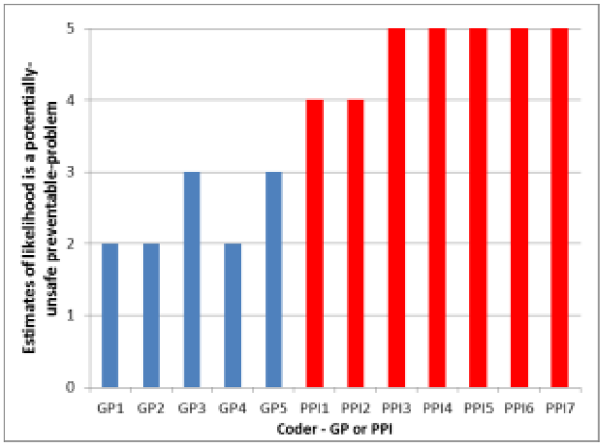

5=very likely or certain, 4=probably, 3=possibly, 2=unlikely, 1=definitely not, 0 = insufficient information or don't know

Patient-reported prospect of harm: your health has been made worse by a problem or error that could have been prevented

Patient-perspective problem-type code: A5. Unable to get an appointment/other problems with making appointment

Scenario50. Physiotherapy at GP surgery

Briefly describe the mistake or problem and how it happened. *"Broken wrist after coming off pushbike"*

Could the mistake or problem have been avoided? If so how? *"Physio caused fracture, after healing, to break again"*

Were you able to talk about the mistake or problem with anybody working in the primary care service? *"Yes, another doctor in practice"*

Patient-reported prospect of harm: your health has been made worse by a problem or error that could have been prevented

Patient-perspective problem-type code: E2.Procedure was not carried out correctly

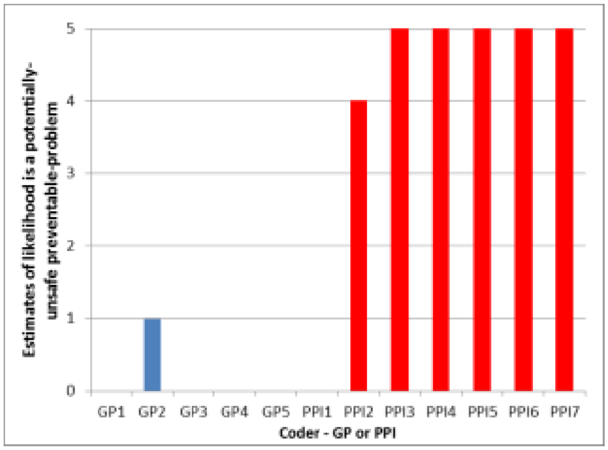

5=very likely or certain, 4=probably, 3=possibly, 2=unlikely, 1=definitely not, 0 = insufficient information or don't know

Scenario51. GP surgery

Briefly describe the mistake or problem and how it happened. *"Given some medication that brought about a nervous breakdown and crisis team attended within 4 hours. Seeing mental health social worker each week now as a result. Hearing voices and seeing things which I didn't before this medication."*

Could the mistake or problem have been avoided? If so how? *"GP could have listened more carefully and not changed my medication"*

Were you able to talk about the mistake or problem with anybody working in the primary care service? *"Yes, the crisis mental health team/the psychologist and social worker"*

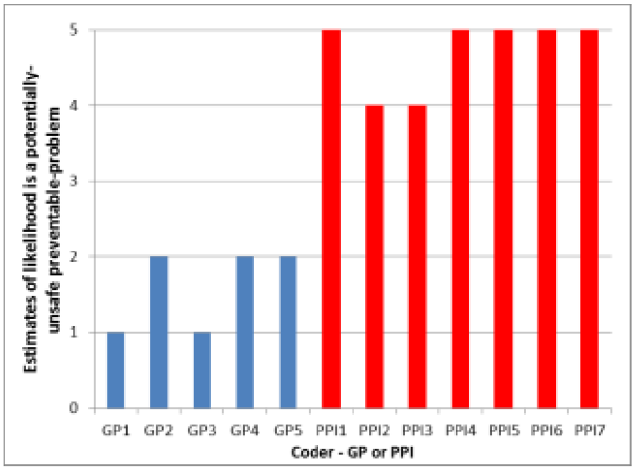

5=very likely or certain, 4=probably, 3=possibly, 2=unlikely, 1=definitely not, 0 = insufficient information or don't know

Patient-reported prospect of harm: suspected your health has been made worse by a problem or error that could have been prevented

Patient-perspective problem-type code: C1.1.2 Started new prescription or changed prescription without sufficient discussion, follow up or checks; D1. Clinician seemed to lack interest in the patient's health problem or did not listen carefully enough

Scenario52. Community mental health

Briefly describe the mistake or problem and how it happened. *"two years delay from GP referral to being able to see psychiatrist at community mental health service. Lack of access meant that he could not be diagnosed with a personality disorder trait in order for medication to be prescribed to treat the problem"*

Could the mistake or problem have been avoided? If so how? *"by referring him back to the previous psychiatrist he was with instead of worrying about boundary changes within the PCTs which are intended to manage caseloads. Basically he was out of catchment, also due to NHS cuts. Also feels these are the result of austerity and people should get social care to help"*

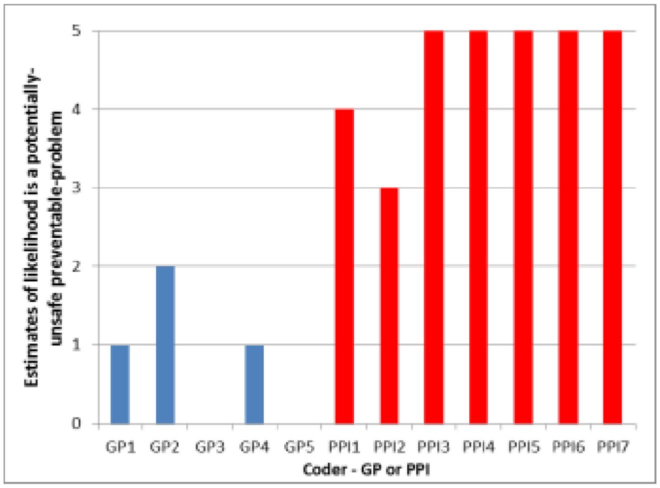

5=very likely or certain, 4=probably, 3=possibly, 2=unlikely, 1=definitely not, 0 = insufficient information or don't know

Were you able to talk about the mistake or problem with anybody working in the primary care service? *"Yes, secretary of mental health psychiatrist he should have seen but waiting for 2 years for*

Patient-reported prospect of harm: your health has been made worse by a problem or error that could have been prevented

Patient-perspective problem-type code: A5. Unable to get an appointment/other problems with making appointment

Scenario53. GP Surgery

Briefly describe the mistake or problem and how it happened. "*I had sore throat and I told the doctor it felt it would go to my chest. He prescribed a throat spray, over 2 days I felt really poorly and ended up in hospital with pneumonia"*

Could the mistake or problem have been avoided? If so how? *"GP should have prescribed antibiotics"*

Were you able to talk about the mistake or problem with anybody working in the primary care service? "*No, I was too distressed to discuss the problem or error"*

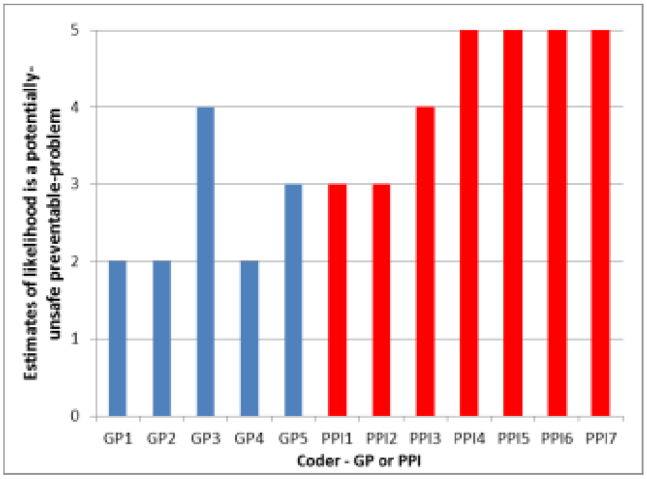

5=very likely or certain, 4=probably, 3=possibly, 2=unlikely, 1=definitely not, 0 = insufficient information or don't know

Patient-reported prospect of harm: your health has been made worse by a problem or error that could have been prevented

Patient-perspective problem-type code: G1.Wrong treatment decision

Scenario54. GP Surgery

Briefly describe the mistake or problem and how it happened. "*Got stomach pain, it was very similar to gall bladder pain but had had that removed before so couldn't be that. At first would have made an appointment with my doctor but none were available for a month. I insisted and found out it was gall bladder stones in bile duct which is serious. Total delay (in pain) 3-4 days"*

Could the mistake or problem have been avoided? If so how? *"Quicker appointment"*

Were you able to talk about the mistake or problem with anybody working in the primary care *"Yes, spoke to doctor about the problem. No apology or changes to the service"*

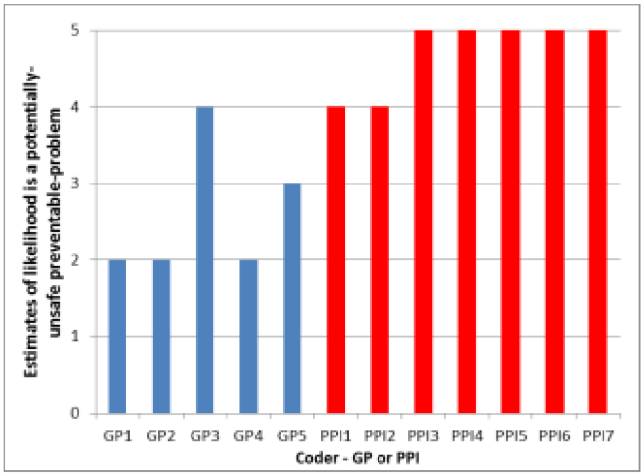

5=very likely or certain, 4=probably, 3=possibly, 2=unlikely, 1=definitely not, 0 = insufficient information or don't know

Patient-reported prospect of harm: health could have been made worse had someone not noticed a problem or error

Patient-perspective problem-type code: A5. Unable to get an appointment/other problems with making appointment

Scenario55. Dental Surgery

Briefly describe the mistake or problem and how it happened. *"Osteonecrosis of the jaw happened due to a tooth being extracted when it should not have been because of medication I was taking"*

Could the mistake or problem have been avoided? If so how? *"More knowledge on the part of the dental profession"*

Were you able to talk about the mistake or problem with anybody working in the primary care service? *"No, there was no point talking about the problem with the primary care service as the situation was beyond that"*

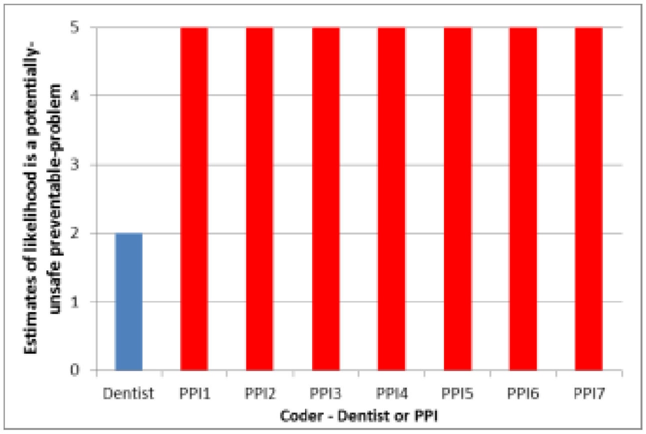

5=very likely or certain, 4=probably, 3=possibly, 2=unlikely, 1=definitely not, 0 = insufficient information or don't know

Patient-reported prospect of harm: your health has been made worse by a problem or error that could have been prevented

Patient-perspective problem-type code: G1. Wrong treatment decision

Scenario56. Physiotherapy

Briefly describe the mistake or problem and how it happened. *"GP referred to physio for shoulder pain, physio made problem worse and operation was required"*

Could the mistake or problem have been avoided? If so how? *"inexperienced physio made wrong diagnosis"*

Were you able to talk about the mistake or problem with anybody working in the primary care service? "*Yes, GP***"**

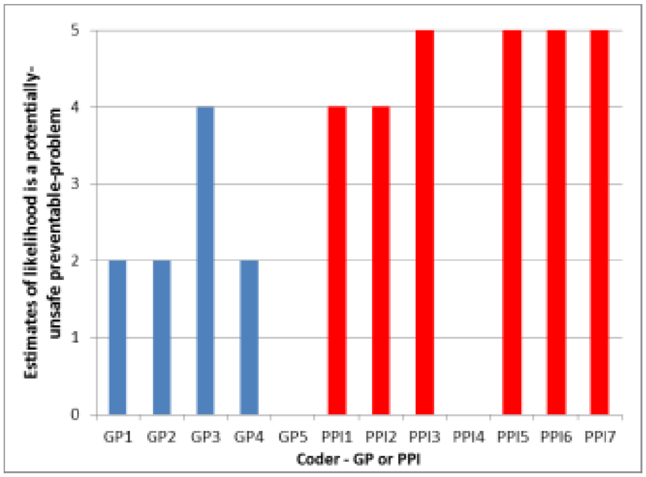

5=very likely or certain, 4=probably, 3=possibly, 2=unlikely, 1=definitely not, 0 = insufficient information or don't know

Patient-reported prospect of harm: your health has been made worse by a problem or error that could have been prevented

Patient-perspective problem-type code:

F1. Wrong/late/missed/delayed diagnosis; G1. Wrong treatment decision

Scenario57. GP Surgery

Briefly describe the mistake or problem and how it happened. *"Have thyroid problem. GP reduced medication dose without a review and caused health to deteriorate"*

Could the mistake or problem have been avoided? If so how? *"by appropriate blood test taken regularly to monitor my thyroid status"*

Were you able to talk about the mistake or problem with anybody working in the primary care service? *"Yes, GP"*

Patient-reported prospect of harm: suspected your health has been made worse by a problem or error that could have been prevented

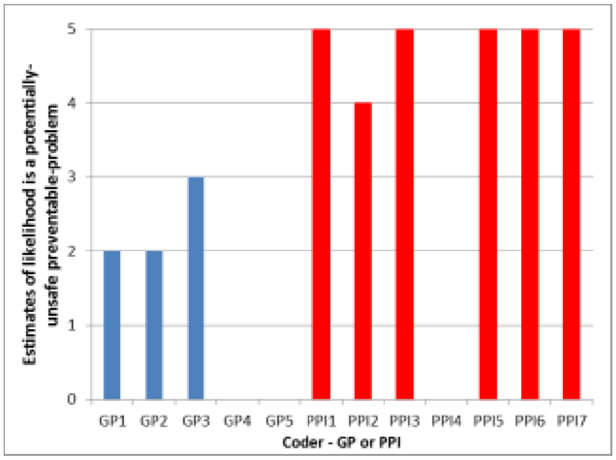

5=very likely or certain, 4=probably, 3=possibly, 2=unlikely, 1=definitely not, 0 = insufficient information or don't know

Patient-perspective problem-type code: B4. Investigation not thorough enough

Scenario58. GP Surgery

Briefly describe the mistake or problem and how it happened. *"review of drugs, GP indicated the high blood pressure, and decided to put me on blood pressure reducing tablets, which resulted in very bad side effects."*

Could the mistake or problem have been avoided? If so how? missing

Were you able to talk about the mistake or problem with anybody working in the primary care service? *"my daughter is GP, she advised me to stop taking the tablets, and monitor my own blood pressure which I did for a week and recorded it."*

Patient-reported prospect of harm: there was a problem or error that could have been prevented but it did not make your health worse

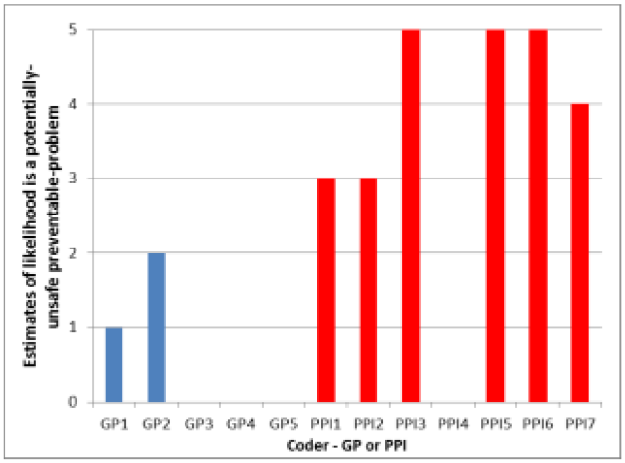

5=very likely or certain, 4=probably, 3=possibly, 2=unlikely, 1=definitely not, 0 = insufficient information or don't know

Patient-perspective problem-type code: C1 Medication error not otherwise specified /other problem

Scenario59. GP Surgery

Briefly describe the mistake or problem and how it happened. *"Complaining about severe pain in right shoulder then left shoulder for 3 years. I demanded to see a specialist. I saw a muscular skeletal specialist who diagnosed me with fibromyalgia, so I am no longer able to go to the gym now."*

Could the mistake or problem have been avoided? If so how? *"If the diagnosis had not have taken as long my overall health and fitness would not have deteriorated. It's affected my mental health and body image and I have paid over 2,000 pounds for private chiropractor"*

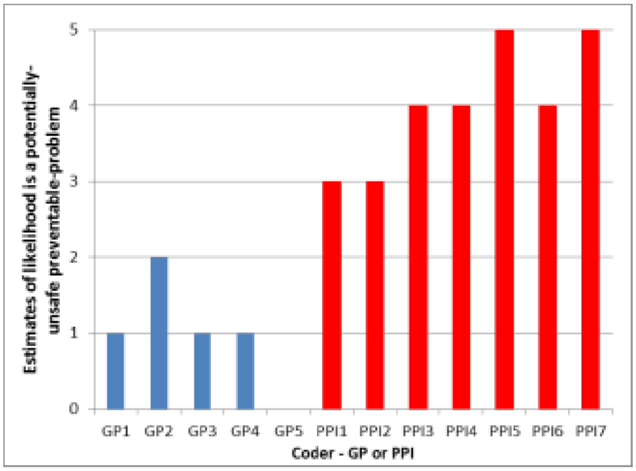

5=very likely or certain, 4=probably, 3=possibly, 2=unlikely, 1=definitely not, 0 = insufficient information or don't know

Were you able to talk about the mistake or problem with anybody working in the primary care service? *"the musculoskeletal specialist when referred listened to me and gave a diagnosis"*

Patient-reported prospect of harm: your health has been made worse by a problem or error that could have been prevented

Patient-perspective problem-type code: B5. Not referred when patient felt was needed

<u>Patient reported scenarios occurring during the past 12 months that PPIs scored as higher likelihood to be a potentially-unsafe preventable-problem in primary care compared with clinicians – pilot survey (reference 24)</u>

Scenario60. GP Surgery

Briefly describe the mistake or problem and how it happened. *"I had a severe reaction to Atorvastatin after a dose increase so much so that I was almost immobile and took 4 months to recover"*

Could the mistake or problem have been avoided? If so how? *"According to guidelines I should have been on the increased dose - it took a long time to convince the GP that I needed blood tests to find out why I couldn't walk. My GP was very hesitant to admit that I did have a reaction to statins."*

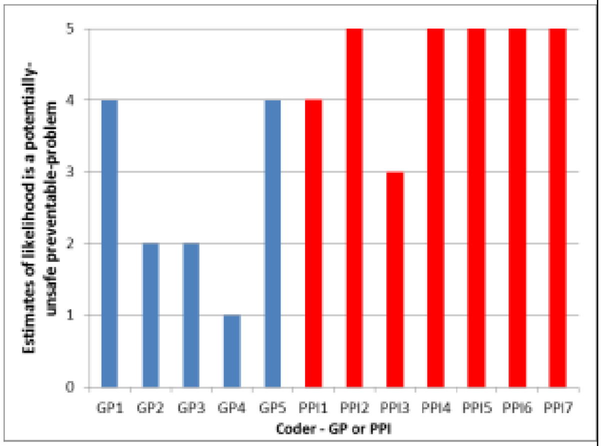

5=very likely or certain, 4=probably, 3=possibly, 2=unlikely, 1=definitely not, 0 = insufficient information or don't know

Were you able to talk about the mistake or problem with anybody working in the primary care service? *"No I could not find anybody with whom I could discuss the mistake or problem. It was not really the GPs fault per se, just took a lot of convincing that there was a problem"*

Patient-reported prospect of harm: health could have been made worse had someone not noticed a problem or error

Patient-perspective problem-type code: C1.1.3 Long term or continued prescribing without review or consideration of long term or side effects

Scenario61. GP Surgery

Briefly describe the mistake or problem and how it happened. *"Doctor kept saying I had vitamin deficiency B1, it turned out I had peripheral neuropathy which is very painful"*

Could the mistake or problem have been avoided? If so how? *"I just needed the proper medication to help"*

Were you able to talk about the mistake or problem with anybody working in the primary care service? *"Just saw another Doctor and she knew straight away what the problem was - she was experienced with Diabetic problems. Yes had the opportunity but did not feel comfortable to discuss the mistake or problem"*

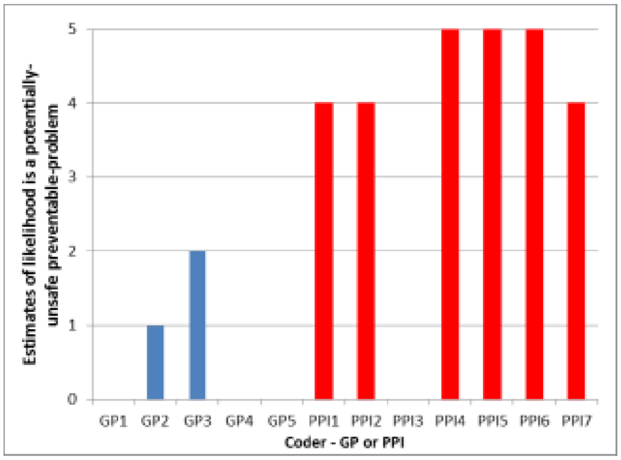

5=very likely or certain, 4=probably, 3=possibly, 2=unlikely, 1=definitely not, 0 = insufficient information or don't know

Patient-reported prospect of harm: prompted via Q10 (Box 1 main paper)

Patient-perspective problem-type code: F1. Wrong/late/missed/delayed diagnosis

Scenario62. GP Surgery

Briefly describe the mistake or problem and how it happened. *"Incapable diabetic doctor trying to take blood out the back of my hand haphazardly, not listening and resulting in me fitting and the student watching having to get help."*

Could the mistake or problem have been avoided? If so how? *"Yes. By listening to me"*

Were you able to talk about the mistake or problem with anybody working in the primary care service? *"No I could not find anybody with whom I could discuss the mistake or problem"*

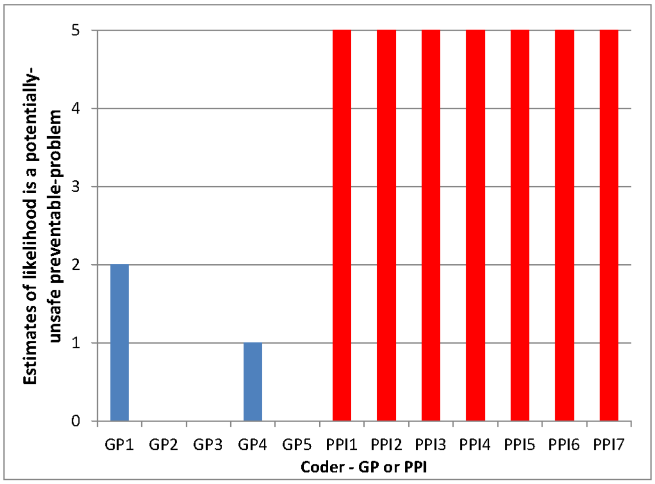

5=very likely or certain, 4=probably, 3=possibly, 2=unlikely, 1=definitely not, 0 = insufficient information or don't know

Patient-reported prospect of harm: prompted via Q10 (Box 1 main paper)

Patient-perspective problem-type code: E2. Procedure was not carried out correctly; D1. Clinician seemed to lack interest in the patient's health problem or did not listen carefully enough

Scenario63. Dental Surgery

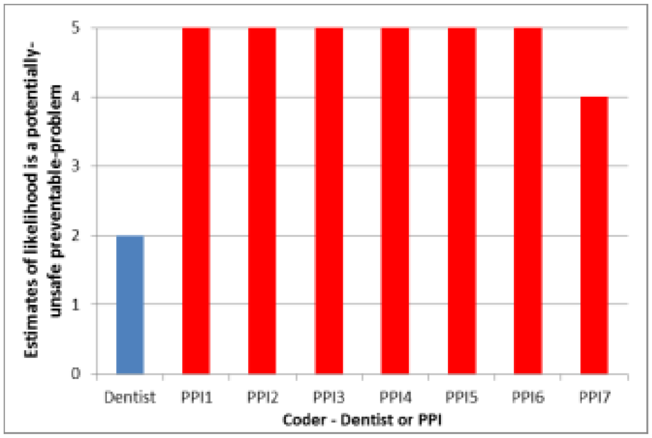

5=very likely or certain, 4=probably, 3=possibly, 2=unlikely, 1=definitely not, 0 = insufficient information or don't know

Briefly describe the mistake or problem and how it happened. *"I had an infection under my wisdom tooth. They agreed that the only way to solve the problem was to take the tooth out. They gave me an appointment to do this in 6 weeks. I am a type 1 diabetic and the infection was affecting my blood sugars and I was concerned that I would have to go to A&E if my blood sugars continued to rise due to the infection. It would have affected my health if I had not paid to go to a private dentist."*

Could the mistake or problem have been avoided? If so how? *"They could have taken out the tooth straight away. I was happy to wait at the emergency dentist for them to do this."*

Were you able to talk about the mistake or problem with anybody working in the primary care service? *"I explained but they said I would have to wait. They also asked if I needed a sugary drink when I said that my sugars were high so I was too scared to eat and had not eaten in 12hrs. It was clear they didn't understand diabetes."*

Patient-reported prospect of harm: health could have been made worse had someone not noticed a problem or error

Patient-perspective problem-type code: A5. Unable to get an appointment/other problems with making appointment

Scenario64. Dental Surgery

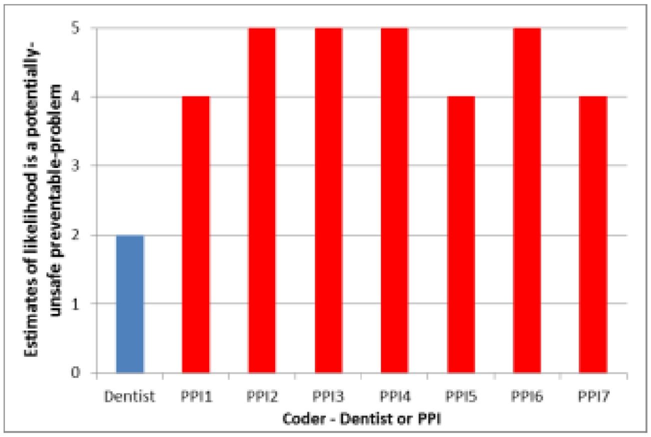

5=very likely or certain, 4=probably, 3=possibly, 2=unlikely, 1=definitely not, 0 = insufficient information or don't know

Briefly describe the mistake or problem and how it happened. *"Caries, cavities and problem with crown not diagnosed or treated"*

Could the mistake or problem have been avoided? If so how? *"Better dentist & not working to tight time-scale imposed by company owning dental surgery"*

Were you able to talk about the mistake or problem with anybody working in the primary care service? *"No I could not find anybody with whom I could discuss the mistake or problem"*

Patient-reported prospect of harm: prompted via Q10 (Box 1 main paper)

Patient-perspective problem-type code: C3. Problem with dental treatment or diagnosis

Scenario65. GP Surgery

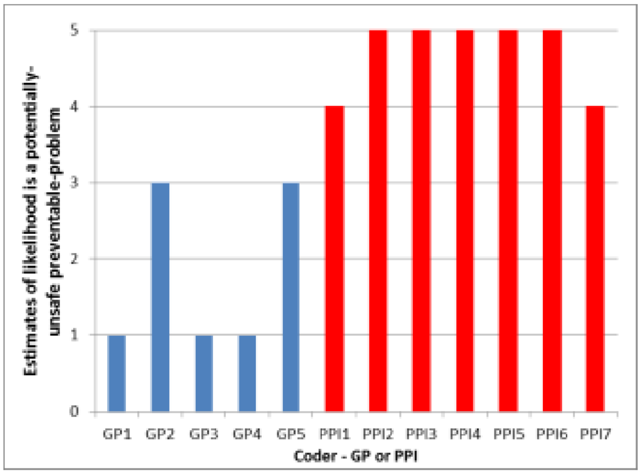

5=very likely or certain, 4=probably, 3=possibly, 2=unlikely, 1=definitely not, 0 = insufficient information or don't know

Briefly describe the mistake or problem and how it happened. *"Using the summary on discharge from hospital, one GP transcribed incorrectly on to my electronic notes ie size of ovarian cyst was 7.5cms and he put 7.5 mms. Another GP requested diagnostic bone density scan but either forgot or did not record it and she ended up questioning why I had it and who requested it. She also referred me for an orthopedic consultation then said I was not funded for the steroid injection put into my swollen elbows."*

Could the mistake or problem have been avoided? If so how? *"Yes"*

Were you able to talk about the mistake or problem with anybody working in the primary care service? *"I was too scared to discuss my concerns for fear of being labelled a trouble maker"*

Patient-reported prospect of harm: health could have been made worse had someone not noticed a problem or error

Patient-perspective problem-type code: A2. Incorrect notes/inadequate notes/notes not kept up to date

Scenario66. GP Surgery

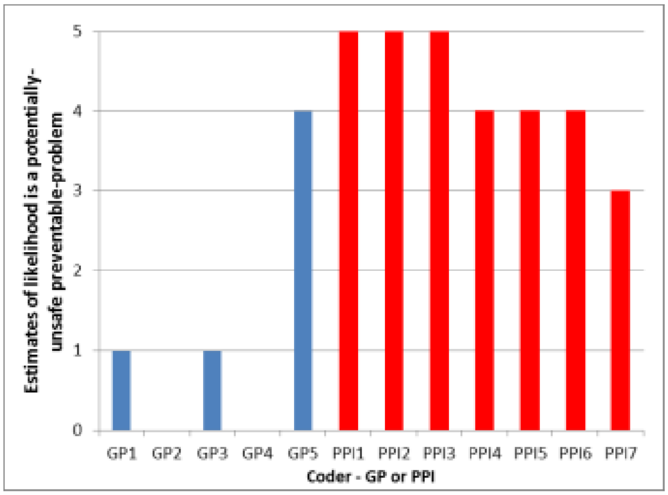

5=very likely or certain, 4=probably, 3=possibly, 2=unlikely, 1=definitely not, 0 = insufficient information or don't know

Briefly describe the mistake or problem and how it happened. *"GP prescribed pills, but then got phone call saying not to take them"*

Could the mistake or problem have been avoided? If so how? *"Not sure"*

Were you able to talk about the mistake or problem with anybody working in the primary care service? *"No I was not concerned about the problem"*

Patient-reported prospect of harm: prompted via Q10 (Box 1 main paper)

Patient-perspective problem-type code: C1. Medication problem

Scenario67. GP Surgery

Briefly describe the mistake or problem and how it happened. *"I had a burst appendix and peritonitis, something that even a scan couldn't detect adequately. My first visit to GP was when I said I think I have appendicitis, no other symptoms only the pain. It was ten days before seeing a consultant, a further 10 days to have a scan, then 2 weeks to be told that I had a lump on my colon which is what my GP had said 5 weeks previously. It was a further 2 weeks before I had surgery."*

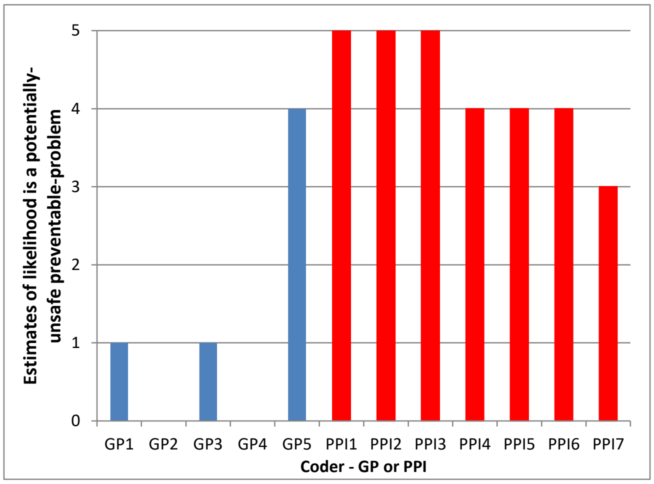

5=very likely or certain, 4=probably, 3=possibly, 2=unlikely, 1=definitely not, 0 = insufficient information or don't know

Could the mistake or problem have been avoided? If so how? *"If my GP had referred me for a scan immediately it would have saved 3 weeks out of the seven. It was two weeks from scan to results and I hear that is usual, but they're not looking at them for 2 weeks"*

Were you able to talk about the mistake or problem with anybody working in the primary care service? *"Had the outcome been different my widow might have pursued the matter further. The system is at fault rather than any individual."*

Patient-reported prospect of harm: your health has been made worse by a problem or error that could have been prevented

Patient-perspective problem-type code: B5. Not referred when patient felt was needed

<u>Patient reported scenarios occurring during the past 12 months that clinicians scored as definitely not a potentially-unsafe preventable-problem in primary care</u>

Scenario68: GP surgery
Description of event: Surgery arranged visits to cytology department at a local hospital; surgery did not ensure accurate visiting times came to patient
How could it be prevented: better communication between surgery and hospital
Were you able to talk about the problem or error with anybody working in the primary care service? deputy practice manager of GP surgery

Scenario69: GP surgery
Description of event: Given some medication that brought about a nervous breakdown and crisis team attended within 4 hours. Seeing mental health social worker each week now as a result. Hearing voices and seeing things which I didn't before this medication.
How could it be prevented: GP could have listened more carefully and not changed my medication.
Were you able to talk about the problem or error with anybody working in the primary care service? the crisis mental health team/the psychologist and social worker

For peer review only

Scenario70: Out of hours care

Description of event: Needed medication for vertigo but out of hours service sent me to A and E thinking I had had a stroke. Had all investigations for stroke over 4 hours, only for conclusion that it was indeed vertigo.

How could it be prevented: Could have ignored their pathway and had more clinical reasoning at the outset.

Were you able to talk about the problem or error with anybody working in the primary care service? No, once on the pathway you have to continue with it – no point in questioning

Scenario71: GP surgery

Description of event: mental health situation

How could it be prevented: doctor seemed unaware and worsened the condition

Were you able to talk about the problem or error with anybody working in the primary care service? attended A&E which got the doctor re-involved

Scenario72: GP surgery

Description of event: problem with process of obtaining blood test results. Lack of information and no communication

How could it be prevented: better communication

Were you able to talk about the problem or error with anybody working in the primary care service? I could not find anybody with whom I could discuss the problem or error

Scenario73: GP surgery

Description of event: I suspected I was told lies about what was on my record

How could it be prevented: My hunch is in the previous practice I belonged to someone was making up information to hit targets by saying I had test I hadn't had

Were you able to talk about the problem or error with anybody working in the primary care service? GP, it made me doubt my own sanity.

Scenario74: walk in clinic

Description of event: waiting time made the problem worse

How could it be prevented: shorter wait

Were you able to talk about the problem or error with anybody working in the primary care service? I was too distressed to discuss the problem or error

Scenario75: Dental/GP surgery

Description of event: A lump in the mouth resulted in me being referred to as out-patient at hospital. A biopsy was taken and then another was taken from the outside. Nothing has happened since then although I now have an indentation on my face. Referred back to my doctor still awaiting remedial treatment.

How could it be prevented: By my dentist who surely could have treated me properly.

Were you able to talk about the problem or error with anybody working in the primary care service? At the hospital I spoke to a consultant who kept referring to his team. The same thing happened at my doctors. It seems that no one will accept responsibility for the problem caused.

For peer review only

Scenario76: Dental surgery

Description of event: The dentist I was seeing had a plan for my treatment but the dentist who replaced her said the plan was "rubbish" and that I had to have private treatment. I had prepared myself for treatment according to the agreed plan but the new dentist tried to persuade me to spend £5000 on private treatment. As a result the dental treatment I need has not been done on the NHS and I have to find another dentist.

How could it be prevented: The problem was that my original dentist who I was happy with moved to the private sector within the same surgery

Were you able to talk about the problem or error with anybody working in the primary care service? I was too distressed to discuss the problem or error

Scenario77:GP surgery

Description of event: attempting to get routine screening and not being offered a convenient time as there is only a 2 week window

How could it be prevented: longer time scales and more choice over appointments

Were you able to talk about the problem or error with anybody working in the primary care service? it would require enormous effort and it was too time consuming to speak to someone

Scenario78: GP surgery

Description of event: Acne around eyes. Wanted dermatologist appointment which was not granted.

How could it be prevented: GP said only if the condition worsened.

Were you able to talk about the problem or error with anybody working in the primary care service? GP

Scenario79: GP surgery

Description of event: Doctor called me fat.

How could it be prevented: Yes, by better communication.

Were you able to talk about the problem or error with anybody working in the primary care service? I was too distressed to discuss the problem or error

Scenario80: GP surgery

Description of event: Six months ago I was referred by my GP to go for breast cancer screening for all women over 50. Since then I have not received the results of the test. I did not have any further contact so I called to check the result and was told it was with your GP. I called the GP and was told they had sent results to my home but I have not received it and six months on I have not heard.

How could it be prevented: I expected a sooner response or immediate response from the GP whatever the results but have had none I expect to call again tomorrow.

Were you able to talk about the problem or error with anybody working in the primary care service? I could not find anybody with whom I could discuss the problem or error

For peer review only

STROBE Statement—Checklist of items that should be included in reports of ***cross-sectional studies***

	Item No	Recommendation
**Title and abstract**	1	(*a*) Indicate the study's design with a commonly used term in the title or the abstract Yes p1
		(*b*) Provide in the abstract an informative and balanced summary of what was done and what was found yes p3
**Introduction**		
Background/rationale	2	Explain the scientific background and rationale for the investigation being reported Yes p4
Objectives	3	State specific objectives, including any prespecified hypotheses yes p4-5
**Methods**		
Study design	4	Present key elements of study design early in the paper yes p5
Setting	5	Describe the setting, locations, and relevant dates, including periods of recruitment, exposure, follow-up, and data collection yes p5
Participants	6	(*a*) Give the eligibility criteria, and the sources and methods of selection of participants yes p5
Variables	7	Clearly define all outcomes, exposures, predictors, potential confounders, and effect modifiers. Give diagnostic criteria, if applicable yes box1, online appendix 1
Data sources/ measurement	8*	For each variable of interest, give sources of data and details of methods of assessment (measurement). Describe comparability of assessment methods if there is more than one group yes p5, online appendix 1
Bias	9	Describe any efforts to address potential sources of bias yes p5 and reference 23
Study size	10	Explain how the study size was arrived at n/a power calculation described in protocol in terms of confidence intervals for generalisability to UK population but sample size was determined for practical reasons as is a descriptive analysis.
Quantitative variables	11	Explain how quantitative variables were handled in the analyses. If applicable, describe which groupings were chosen and why yes p6-7
Statistical methods	12	(*a*) Describe all statistical methods, including those used to control for confounding yes p5
		(*b*) Describe any methods used to examine subgroups and interactions, yes just chi2 tests p5
		(*c*) Explain how missing data were addressed all missing data is listed in the tables so it is completely transparent how this was dealt with, there were few missing data
		(*d*) If applicable, describe analytical methods taking account of sampling strategy the unweighted sample was used. This is not discussed as the difference was very small and adds much complexity without adding important information.
		(*e*) Describe any sensitivity analyses none done
**Results**		
Participants	13*	(a) Report numbers of individuals at each stage of study—eg numbers potentially eligible, examined for eligibility, confirmed eligible, included in the study, completing follow-up, and analysed yes online appendix 3
		(b) Give reasons for non-participation at each stage yes online appendix 3
		(c) Consider use of a flow diagram yes online appendix 3
Descriptive data	14*	(a) Give characteristics of study participants (eg demographic, clinical, social) and information on exposures and potential confounders yes table 1
		(b) Indicate number of participants with missing data for each variable of interest yes

For peer review only - http://bmjopen.bmj.com/site/about/guidelines.xhtml

		all tables
Outcome data	15*	Report numbers of outcome events or summary measures yes all tables
Main results	16	(*a*) Give unadjusted estimates and, if applicable, confounder-adjusted estimates and their precision (eg, 95% confidence interval). Make clear which confounders were adjusted for and why they were included yes table 3
		(*b*) Report category boundaries when continuous variables were categorized yes all tables
		(*c*) If relevant, consider translating estimates of relative risk into absolute risk for a meaningful time period yes p9
Other analyses	17	Report other analyses done—eg analyses of subgroups and interactions, and sensitivity analyses table 6 considers demographics for problems more likely to be a potentially harmful.
**Discussion**		
Key results	18	Summarise key results with reference to study objectives yes p9
Limitations	19	Discuss limitations of the study, taking into account sources of potential bias or imprecision. Discuss both direction and magnitude of any potential bias yes p11
Interpretation	20	Give a cautious overall interpretation of results considering objectives, limitations, multiplicity of analyses, results from similar studies, and other relevant evidence yes p11-12
Generalisability	21	Discuss the generalisability (external validity) of the study results yes p10
**Other information**		
Funding	22	Give the source of funding and the role of the funders for the present study and, if applicable, for the original study on which the present article is based yes p13

*Give information separately for exposed and unexposed groups.

Note: An Explanation and Elaboration article discusses each checklist item and gives methodological background and published examples of transparent reporting. The STROBE checklist is best used in conjunction with this article (freely available on the Web sites of PLoS Medicine at http://www.plosmedicine.org/, Annals of Internal Medicine at http://www.annals.org/, and Epidemiology at http://www.epidem.com/). Information on the STROBE Initiative is available at www.strobe-statement.org.

For peer review only
